# Chemoenzymatic Synthesis of Original Stilbene Dimers Possessing Wnt Inhibition Activity in Triple-Negative Breast Cancer Cells Using the Enzymatic Secretome of *Botrytis cinerea* Pers.

**DOI:** 10.3389/fchem.2022.881298

**Published:** 2022-04-19

**Authors:** Robin Huber, Alexey Koval, Laurence Marcourt, Margaux Héritier, Sylvain Schnee, Emilie Michellod, Leonardo Scapozza, Vladimir L. Katanaev, Jean-Luc Wolfender, Katia Gindro, Emerson Ferreira Queiroz

**Affiliations:** ^1^ School of Pharmaceutical Sciences, University of Geneva, CMU, Geneva, Switzerland; ^2^ Institute of Pharmaceutical Sciences of Western Switzerland, University of Geneva, CMU, Geneva, Switzerland; ^3^ Department of Cell Physiology and Metabolism, Translational Research Centre in Oncohaematology, Faculty of Medicine, University of Geneva, CMU, Geneva, Switzerland; ^4^ Mycology Group, Research Department Plant Protection, Agroscope, Nyon, Switzerland; ^5^ Institute of Life Sciences and Biomedicine, Far Eastern Federal University, Vladivostok, Russia

**Keywords:** chemoenzymatic synthesis, *Botrytis cinerea*, enzymatic secretome, stilbene dimers, high-resolution semi-preparative HPLC, dry load introduction, Wnt inhibition, triple negative breast cancer cells

## Abstract

The Wnt signaling pathway controls multiple events during embryonic development of multicellular animals and is carcinogenic when aberrantly activated in adults. Breast cancer and triple-negative breast cancer (TNBC) in particular depend upon Wnt pathway overactivation. Despite this importance, no Wnt pathway-targeting drugs are currently available, which necessitates novel approaches to search for therapeutically relevant compounds targeting this oncogenic pathway. Stilbene analogs represent an under-explored field of therapeutic natural products research. In the present work, a library of complex stilbene derivatives was obtained through biotransformation of a mixture of resveratrol and pterostilbene using the enzymatic secretome of *Botrytis cinerea*. To improve the chemodiversity, the reactions were performed using *i*-PrOH, *n*-BuOH, *i*-BuOH, EtOH, or MeOH as cosolvents. Using this strategy, a series of 73 unusual derivatives was generated distributed among 6 scaffolds; 55 derivatives represent novel compounds. The structure of each compound isolated was determined by nuclear magnetic resonance and high-resolution mass spectrometry. The inhibitory activity of the isolated compounds against the oncogenic Wnt pathway was comprehensively quantified and correlated with their capacity to inhibit the growth of the cancer cells, leading to insights into structure-activity relationships of the derivatives. Finally, we have dissected mechanistic details of the stilbene derivatives activity within the pathway.

## Introduction

In developed countries, breast cancer is the most frequent malignancy and consequently the first cause of cancer-related death among women. The development of new targeted therapies, such as aromatase inhibitors, monoclonal antibodies (trastuzumab, pertuzumab, T-DM1), and lapatinib, has improved remarkably the outcome for endocrine-sensitive or HER2-positive breast cancer subtypes (Giordano et al., 2004). Some interesting developments resulted in further improvement of these agents, such as creation of clinically approved trastuzumab-based antibody-drug conjugates (Ado-Trastuzumab Emtansine and Fam-Trastuzumab Deruxtecan) (Mukohara et al., 2021). Additionally, HER2-positive tumors respond well to several clinically approved tyrosine kinase inhibitors (Schlam and Swain, 2021). However, the prognosis of patients with tumors negative for these factors, defined as triple-negative breast cancer subtype (TNBC), remains poor since this form of breast cancer elicits an aggressive behavior with early recurrence and a higher rate of visceral and brain metastases. Targeted therapies are virtually non-existent for TNBC with a notable exception of recent approval of sacituzumab govitecan—an anti-TROP2 antibody drug conjugate shown to be effective as a last resort against resistant and metastatic disease (Seligson et al., 2020). The treatment options are thus generally limited to cytotoxic chemotherapies, to which the disease becomes rapidly resistant (Kassam et al., 2009; Bianchini et al., 2021). There is therefore an urgent need to develop specific therapies for this subgroup of patients.

The Wnt signaling pathway controls multiple events during embryonic development of multicellular animals of almost all taxa starting from sponges, and, when aberrantly activated in the adult, is carcinogenic (Barker and Clevers, 2006; Blagodatski et al., 2014). The pathway is triggered by Wnt lipoglycoproteins binding the co-receptor pair, Frizzled (FZD) and LRP5/6; the signal is subsequently transmitted by Dishevelled (Dvl) and heterotrimeric G proteins. Their coordinate action dissociates the Axin-APC-GSK3β-casein kinase complex, which in its active state destabilizes β-catenin. Thus, when the pathway is “on,” β-catenin accumulates in the cytoplasm and consequently in the nucleus where it acts as a co-factor for transcription of the target genes through interaction with the TCF/LEF transcription factor.

Breast cancers are dependent on Wnt pathway overactivation mostly through aberrations in the expression of the pathway components rather than through mutations (Turashvili et al., 2006; Khramtsov et al., 2010; Dey et al., 2013; Koval and Katanaev, 2018). Genetic targeting of the Wnt pathway components identified the role of this pathway in TNBC proliferation and metastasis (Yang et al., 2011; Gurney et al., 2012; Wend et al., 2013; Simmons et al., 2014; Yang et al., 2014; Le et al., 2015) as well as breast cancer stem cells (CSC) proliferation and maintenance (Jang et al., 2015; Wang et al., 2016) that contribute to the development of tumor resistance to conventional chemotherapies (Abdullah and Chow, 2013; O’toole et al., 2013; O’reilly et al., 2015), with a specific role belonging to the receptor proteins in the pathway, Frizzleds (Larasati et al., 2022). Collectively, these findings identify the Wnt pathway as an excellent target to develop novel selective therapies and co-therapies, both for the primary TNBC and the prevention of tumor metastasis and relapse.

In context of these findings and inspired by our recent success on the subject, such as repositioning of the lichen-sourced compound clofazimine and its derivatives against Wnt signaling (Xu et al., 2020; Koval et al., 2021) we have embarked on a discovery of the novel anti-Wnt therapies using our platform for targeted screening of the such compounds (Shaw et al., 2019; Katanaev et al., 2021). Since there are multiple attractive targets within pathway which might mediate pharmacologically relevant anti-cancer effect, we have created a high-throughput system based on the triple-negative breast cancer cells stably transfected with TopFlash reporter, thus accurately representing the entire repertoire of pathway aberrations driving the disease (Veeman et al., 2003). In this adapted system, the activation of the pathway by Wnt3a ligand results in complete engagement of all the downstream components since it measures firefly luciferase expression levels induced by Wnt-specific transcription factors of TCF/LEF family, and thus the compound engaging any of the intermediate target within the pathway will be identified (Veeman et al., 2003). Furthermore, employment of a counter-screen using constitutively expressed *Renilla* luciferase allows us to immediately distinguish compounds inhibiting TopFlash unspecifically, usually by induction of toxicity, though other mechanisms are possible as well (Shaw et al., 2019). Moreover, using pharmacological approach, such as use of GSK3β inhibitor CHIR99021, we can rapidly dissect a more exact mode of action of active compounds (An et al., 2010).

Natural products have been one of the main historical sources of anticancer drugs (Newman and Cragg, 2020). Stilbenes are phenolic natural products produced by the phenylpropanoid pathway present in numerous families of plants (Shen et al., 2009). Some of them, such as resveratrol and pterostilbene, have been ascribed to a wide range of biological activities against cancer. Most notable of them are associated with effects on oxidative homeostasis, membrane structure and integrity, as well as a broad scope of intracellular pathways, ultimately activating responses resulting in tumor cell inhibition and chemosensitization—these activities are summarized in the recent excellent reviews (Obrador et al., 2021; Quarta et al., 2021; Ren et al., 2021). It has been further reported that resveratrol and pterostilbene have a potential Wnt-inhibition activity in several models and tissues (Blagodatski et al., 2020). However, this dependency on the promiscuous interactions of these molecules with multiple targets, has a backlash in being strongly context-dependent and poorly reproducible *in vivo* (Baell and Walters, 2014). Despite that, resveratrol and pterostilbene possess interesting properties as the starting point for the production of more complex derivatives with potentially specific and targeted activities.

Recently, we successfully used an enriched fraction of enzymes from fungi (“fungal secretome”) for the biotransformation of natural products to create complex derivative compounds with improved biological activity (Gindro et al., 2017; Righi et al., 2020; Huber et al., 2021). The enzymatic diversity produced by fungi used for the degradation and/or transformation of various substrates has the potential to catalyze different chemical reactions (Gindro et al., 2017). The use of such fungal secretomes for biotransformation thus has great potential to transform various types of substrates with less specificity than pure enzymes (Lopes et al., 2014) and is probably well suited to generate high chemodiversity by the transformation of related rather simple initial natural compound substrates. In addition, the use of a fungal secretome rather than whole-cell incubation facilitates the isolation of biotransformed compounds due to the absence of fungal metabolites in the final mixture.

It was also demonstrated that biotransformation reactions can be performed in the presence of high amounts of organic solvents (Righi et al., 2020). These solvents can improve the solubility of substrates in aqueous media and also allow a relatively easy product recovery (Klibanov, 2001). Moreover, the use of MeOH as a cosolvent was shown to generate new compounds by unexpected solvolysis reactions (Righi et al., 2020).

In this context, we extended this approach to 4 other cosolvents (*i*-PrOH, *n*-BuOH, *i*-BuOH and EtOH) in order to increase the chemodiversity of the stilbene dimers library obtained by the biotransformations of a mixture of resveratrol and pterostilbene using the *Botrytis cinerea* Pers. enzymatic secretome. This library was generated in view of a Wnt inhibition screening; the generation of close analogs allowing a study of the structure-activity relationship with the aim of determining a possible pharmacophore.

## Results and Discussion

### Generation of Stilbene Analogs by Biotransformation Using Organic Solvents and the Enzymatic Secretome of *Botrytis cinerea*

In previous studies, biotransformation reactions with the *B. cinerea* secretome allowed us to obtain 6 families of complex dimers with several stereochemical centers starting from simple stilbenes without asymmetric carbon (Gindro et al., 2017). To overcome solubility issues, a series of biotransformation reactions were performed with a mixture of resveratrol and pterostilbene in presence of different amounts of acetone or methanol as a cosolvent with water (Righi et al., 2020). The reactions occurred even at a high organic solvent ratio, and methoxylated derivatives with interesting biological activities were successfully obtained when the reaction was performed with methanol (Righi et al., 2020).

The study of the reactions at the origin of these unexpected compounds indicated that it was a nucleophilic attack of the solvent (solvolysis) on a *sp* ^*2*^ carbon of an intermediate structure that generated them (Righi et al., 2020). Taking advantage of this observation, we designed a series of biotransformation reactions with a mixture of resveratrol and pterostilbene in presence of different alcohols to obtain a series of analogs. This ability to generate a variety of close analogs combined with the existence of results concerning the ability of resveratrol to inhibit the Wnt pathway in several models and tissues led us to generate a library of compounds based on this pharmacophore to evaluate their activity.

**FIGURE 1:**
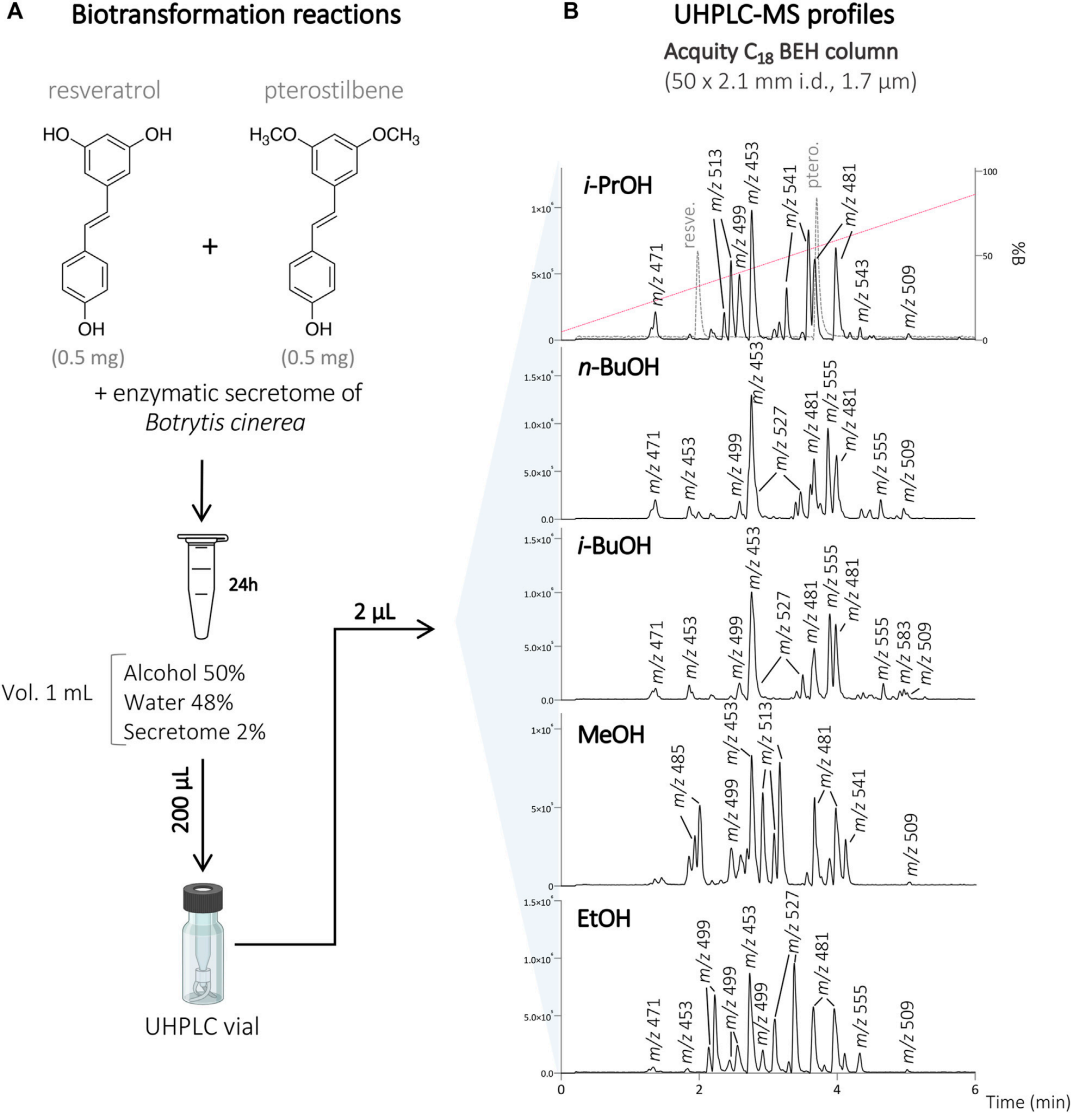
**(A)** Biotransformation reactions of the mixture of resveratrol and pterostilbene with the secretome of *Botrytis cinerea* in the presence of 50% of different organic solvents (*i*-PrOH, *n*-BuOH, *i*-BuOH, MeOH, EtOH). **(B)** UHPLC-MS profile of the different reactions at the analytical scale using an Acquity C_18_ BEH column 50 × 2.1 mm i.d., 1.7 µm. The retention times of the starting compounds resveratrol and pterostilbene are indicated by the dashed line on the reaction with *i*-PrOH. The molecular ion species for all the main compounds detected are indicated with their corresponding *m/z* values.

To this end, resveratrol and pterostilbene (total concentration of 1 mg/ml; 0.5 mg/ml of each compound) were diluted in the organic solvent (*i*-PrOH, *n*-BuOH, *i*-BuOH, EtOH, or MeOH) and H_2_O, before incubation with the secretome of *B. cinerea* over 24 h. Each reaction was monitored by UHPLC-PDA-ELSD-MS (data not shown). The biotransformation reactions were performed with 50% of organic solvents according to our previous study (Righi et al., 2020). With this amount of organic solvent, the compounds were soluble, and based on the LC-MS monitoring, a series of molecular ion species corresponding to the presence of new dimeric analogs were observed (Figure 1).

To isolate these compounds, the biotransformation reactions with each cosolvent were scaled-up keeping the same conditions, except the amount of substrate which was increased to 200–400 mg (see Experimental section). After 24 h, the reactions were monitored by UHPLC-PDA-ELSD-MS. As expected, resveratrol and pterostilbene were fully consumed, demonstrating a good enzymatic activity under such optimized conditions.

**FIGURE 2:**
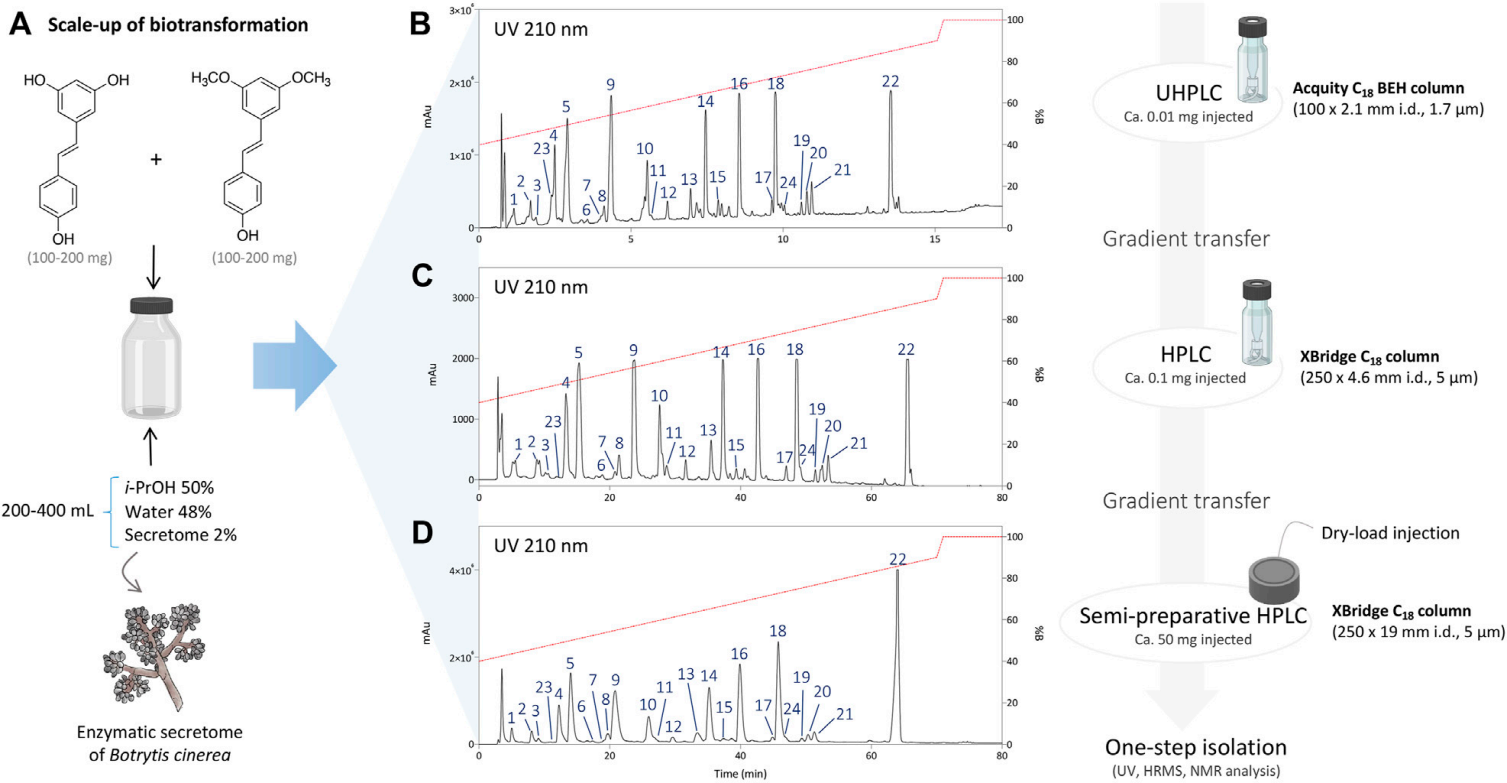
**(A)** Scale-up conditions of the biotransformation reaction of resveratrol and pterostilbene with the secretome of *Botrytis cinerea* with 50% of *i*-PrOH, 2% of enzymatic secretome and 48% of water (total volume 200–400 ml). **(B)** Metabolite profile of the reaction after 24 h using UHPLC-PDA with optimized chromatographic conditions. **(C)** Analytical HPLC-PDA chromatogram after gradient transfer of the UHPLC-optimized conditions. **(D)** Semi-preparative HPLC-PDA chromatogram after gradient transfer of the UHPLC-optimized conditions.

**FIGURE 3:**
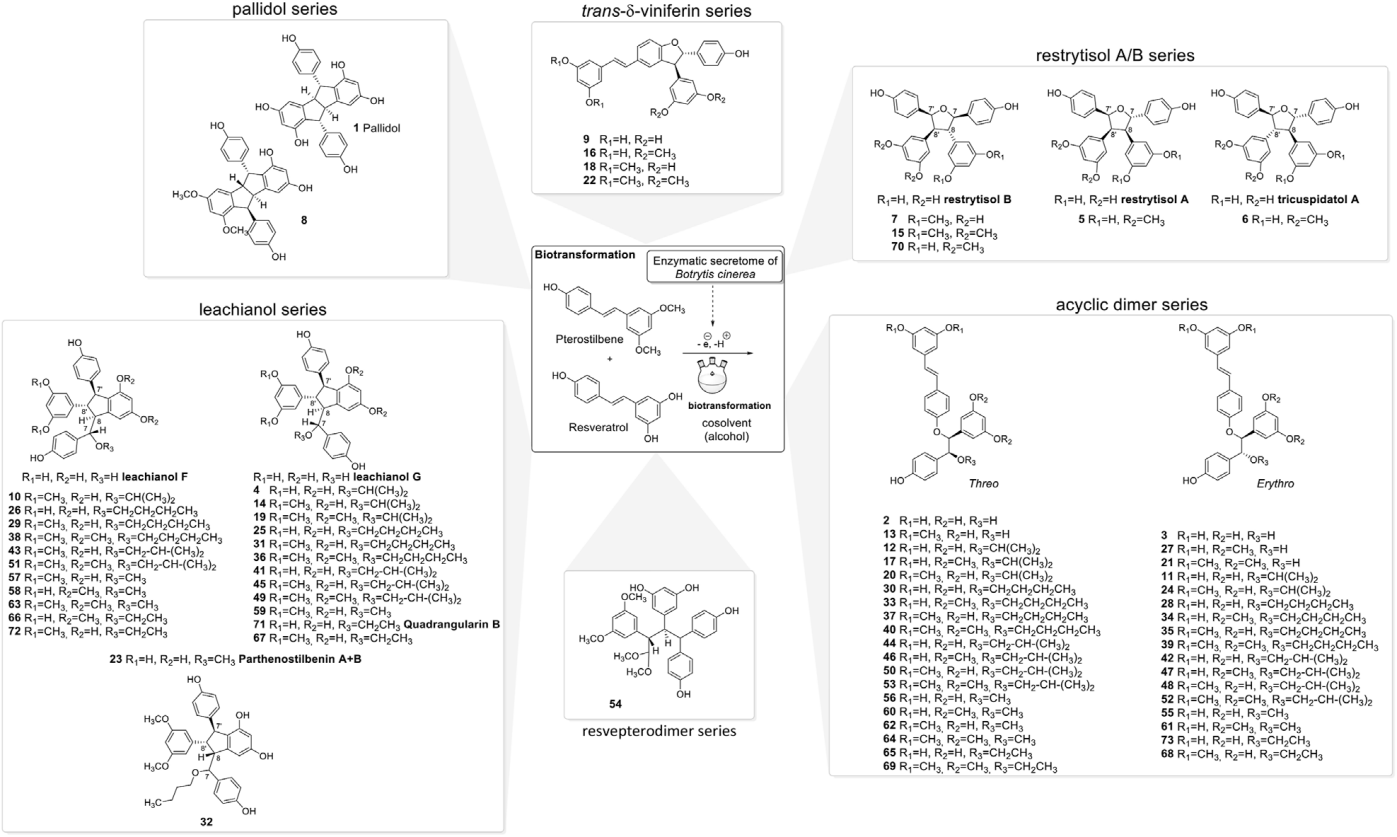
Compounds isolated from the biotransformation reactions of the mixture of resveratrol and pterostilbene with the secretome of *Botrytis cinerea* in the presence of 50% of different organic solvents (*i*-PrOH, *n*-BuOH, *i*-BuOH, EtOH, or MeOH).

After initial reaction monitoring, separation conditions were optimized at the UHPLC scale, allowing for rapid and low solvent consumption runs. These conditions were transferred to the analytical (as a control) and semi-preparative HPLC scale using geometric gradient transfer. The same column chemistry was used on each instrument to maintain the same chromatographic selectivity (Figure 2; Supplementary Figures S1–S4) (Guillarme et al., 2007; Guillarme et al., 2008). At the semi-preparative scale, the samples were injected by dry-load instead of a conventional loop injection, following a protocol recently developed in our laboratory (Queiroz et al., 2019). This approach avoids the loss of resolution caused by the organic solvent during the sample injection. Thanks to this approach, the separations obtained at the semi-preparative HPLC scale perfectly matched those obtained at the analytical scale, allowing the isolation of a majority of compounds in one step. Using this approach, the crude mixtures of the five biotransformation reactions were efficiently purified affording a total of 73 pure compounds; compounds **1**–**24** from the reaction with *i*-PrOH, **25**–**40** from *n*-BuOH, **41**–**53** from *i*-BuOH, **54**–**64** from MeOH and **65**–**73** from EtOH (Figure 3).

The NMR analysis of the compounds generated by the biotransformation reactions of resveratrol and pterostilbene indicates that 6 main scaffolds are formed following the dimerization of the 2 starting entities (Figure 3).

**FIGURE 4:**
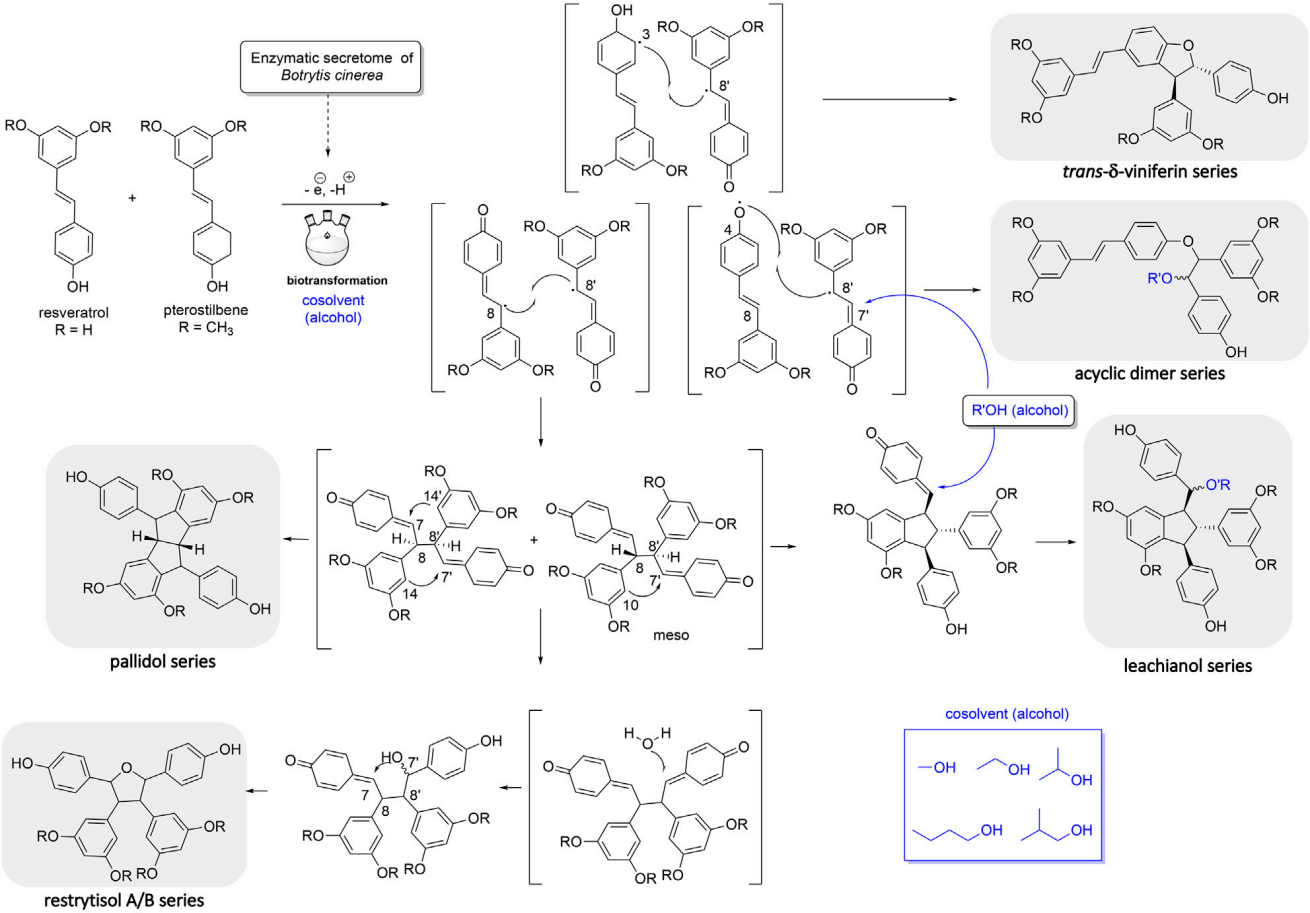
Mechanism of reaction explaining the generation of the 5 main scaffolds (trans-δ-viniferin, acyclic dimer, leachianol, pallidol, restrytisol A/B) of stilbene dimers obtained by the biotransformation reaction of resveratrol and pterostilbene using the enzymatic secretome of *Botrytis cinerea* in the presence of alcohol as cosolvents.

**FIGURE 5:**
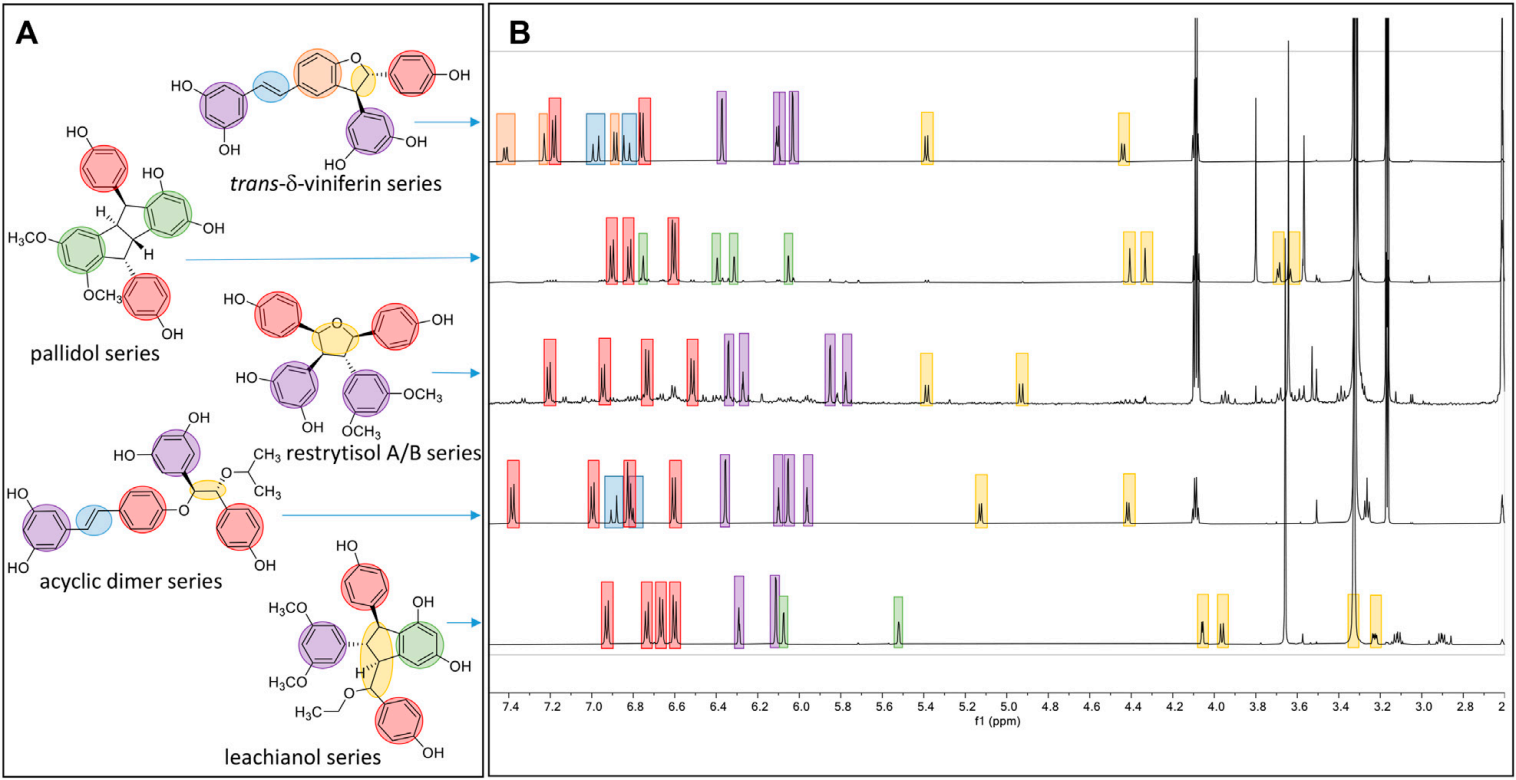
Overview of the typical ^1^H-NMR shifts of the different scaffolds generated. **(A)** Five compounds representing the five main scaffolds obtained by the biotransformations reactions. **(B)** The typical ^1^H-NMR spectra of each scaffold is highlighted in blue for the *trans* double-bound; orange for the 1,3,4-trisubstituted aromatic cycle; red for a para-disubstituted system; purple for the 1,3,5-trisubstituted aromatic cycle; green for the two meta coupled proton; and yellow for the methine protons.

The first one is the well-known *trans*-δ-viniferin series, resulting from a 3-8′ coupling (Figure 4). This scaffold includes only 4 compounds: *trans*-δ-viniferin (**9**), 11′,13′-di-*O*-methyl-*trans*-δ-viniferin (**16**), 11,13-di-*O*-methyl-*trans*-δ-viniferin (**18**), and pterostilbene-*trans*-dehydromer (**22**). These compounds have already been isolated from our previous biotransformations (Gindro et al., 2017). The characteristic ^1^H NMR signals of these compounds are 2 doublets (^3^J_HH_ = 16.3–16.4 Hz) at δ_H_ 6.97–7.22 for H-7 and δ_H_ 6.82–6.96 for H-8 of the *trans* double bond and the H-2, H-5, and H-6 protons from the 1,3,4-trisubstituted aromatic cycle at δ_H_ 7.12–7.24 (1H, d, *J* = 1.9 Hz, H-2), 6.85–6.90 (1H, d, *J* = 8.3 Hz, H-5) and 7.38–7.44 (1H, dd, *J* = 8.3, 1.9 Hz, H-6) (Figure 5).

The second one is the pallidol series, resulting from an 8-8′ coupling followed by a 7-14′ and 14-7′ cyclization (Figure 4). This scaffold is represented by pallidol (**1**) and 11, 13-di-*O*-methylisopallidol (**8**). The double cyclization (7-14′ and 14-7′) leads to the disappearance of the 2 systems consisting of a triplet integrating for one proton and a doublet integrating for two protons (*J* = 2.1–2.3 Hz) and the appearance of 2 pairs of doublets (*J* = 2.1–2.3 Hz) (Figure 5). Due to the symmetrical structure of pallidol (**1**), only the signals of one monomer are visible which is not the case for 11,13-di-*O*-methylisopallidol (**8**). These 2 compounds have already been isolated from our previous works (Gindro et al., 2017).

The third one is the restrytisol A/B series, resulting from an 8-8′ coupling followed by water addition in 7 or 7′ and O-7 or 7′ cyclization (Figure 4). 5 compounds belong to this scaffold. In this case, every aromatic signal of the resveratrol is preserved, i.e., the 2 doublets (2H each) with ortho coupling (*J* = 8.3–8.6 Hz) from the para-disubstituted system and the triplet (1H)/doublet (2H) with meta coupling (*J* = 2.1–2.3 Hz) from the trisubstituted aromatic (Figure 5). One di-*O*-methyl derivative (**5**) has the same configuration as restrytisol A as indicated by the ROE correlations from H-7 to H-10/H-14 and H-10′/H-14′, from CH_3_O-11′/CH_3_O-13′ to H-10′/H-14′ and from H-7′ to H-2/H-6 and from H-8. This compound is a new derivative named 11′,13′-*O*-dimethylrestrytisol A (**5**). A second di-*O*-methyl derivative (**6**) is also a new derivative. It has the same configuration as tricuspidatol A as confirmed by the ROE correlations from H-7 to H-8′ and H-10/H-14 and from H-7′ to H-8 and H-10′/H-14′. It was thus named 11′,13′-*O*-dimethyl tricuspidatol A (**6**). The three remaining compounds belong to the restrytisol B configuration and correspond to the two possible di-*O*-methyl derivatives (**7** already isolated from previous biotransformations and one new compound **70** identified as 11′,13′-di-*O*-methylrestrytisol B) and the tetra-*O*-methyl derivative **15** also isolated from previous biotransformations (Gindro et al., 2017). The following ROE correlations were key to confirm the restrytisol B isomerism: from H-7 to H-8′, H-7′ and H-10/H-14 and from H-8 to H-2/H-6, H-2′/H-6′ and H-10′/H-14′. In summary for this restrytisol A/B series, 5 compounds were synthesized and 3 are described here for the first time.

The compounds belonging to these first three series were not able to incorporate solvent in their structure unlike those of the fourth one, named the acyclic dimer series and which results from an 8′-O-4 coupling (Figure 4). The compounds of this series were recognized by ^1^H-NMR by the presence of 2 doublets (^3^ *J*_HH_ = 16.3–16.6 Hz) at δ_H_ 6.86–7.14 for H-7 and δ_H_ 6.79–6.96 for H-8 from the *trans* double bound, 4 doublets (2H each) with ortho coupling (*J* = 8.3–8.6 Hz) from the 2 para-disubstituted ring and 2 triplets (1H each)/2 doublets (2H each) with meta coupling (*J* = 2.1–2.3 Hz) from the 2 trisubstituted rings, as well as 2 oxymethine between δ_H_ 4.29–4.73 for H-7′ and δ_H_ 5.00–5.34 for H-8′ (Figure 5). This series contains 36 compounds because a nucleophilic addition of the alcohol used as cosolvent happens in C-7′ after the 8′-O-4 coupling between the 2 monomer radicals (Figure 4). Moreover, 2 diastereoisomers in C-7′ and C-8′ are possible and are defined as *erythro* and *threo*. Compounds **2**, **55**, and **56** were already prepared by oxidative coupling reaction using AgOAc as oxidant by Zhang et al. (2014). They identified each diastereoisomer based on the larger ^3^J_H-7′-H-8′_ coupling constant in the *threo* form compared to the *erythro* and from the weak NOE effect between H-7′ and H-8′ and from H-7′ to H-2′/H-6′ and H-10′/H-14′. They also indicated that the chemical shift values of C-7′ and C-8′ were discriminant: in the *threo* form, these signals were shifted to a lower field than those in the *erythro* form. This was also described by Braga et al. (1984) in their conformational analysis of 8-O-4′ neolignans. In our series of data, both the coupling constant between H-7′ and H-8′ and the ROE correlations were the same for each diastereoisomer. Only the ^13^C chemical shifts values were slightly different, for example in the case of a hydroxy group in C-7′ the values were [δ_C_: 75.6–75.7 (C-7′), 83.1–83.7 (C-8′)] in the *erythro* form and [δ_C_: 76.1 (C-7′), 84.0–84.1 (C-8′)] in the *threo* form or in the case of an isobutanol group in C-7′ the values were [δ_C_: 83.5–84.0 (C-7′), 81.6–81.9 (C-8′)] in the *erythro* form and [δ_C_: 84.4–84.6 (C-7′), 82.6–82.9 (C-8′)] in the *threo* form. Due to the small difference of C-7′ and C-8′ ^13^C chemical shift in DMSO-*d*_6_ between the *threo* and *erythro* form and the lack of difference of the ^3^ *J*_H-7′-H-8′_ coupling constant and ROE correlations, the 2 hydroxylated diastereoisomer **2** and **3** were analyzed again in acetone-*d*_6_. Compound **2** displayed the same chemical shift than *threo*-5-[1-[4-[(1*E*)-2-(3,5-dihydroxyphenyl)ethenyl]phenoxy]-2-hydroxy-2-(4-hydroxyphenyl)ethyl]-1,3-benzenediol described by Zhang et al. (2014) and the ^3^ *J*_H-7′-H-8′_ coupling constant was 7.0 Hz for **2** (*threo*) and 5.8 Hz for **3** (*erythro*). These data confirmed the *threo*/*erythro* assignment made in DMSO-*d*_6_. A more probable free rotation along the C-7′-C-8′ bond in DMSO-*d*_6_ giving an average value of coupling constant compared to acetone-*d*_6_ could explain the differences observed between these two solvents. Among the 36 compounds of this acyclic series, **2** and one of the two diastereoisomers **55** or **56** were already described by Zhang et al. (2014), **21** by Ponzoni et al. (2007), **65** and **73** by Shingai et al. (2011) as well as **60** and **62** from our previous work (Righi et al., 2020). The remaining 29 derivatives are therefore compounds that are described here for the first time.

**FIGURE 6:**
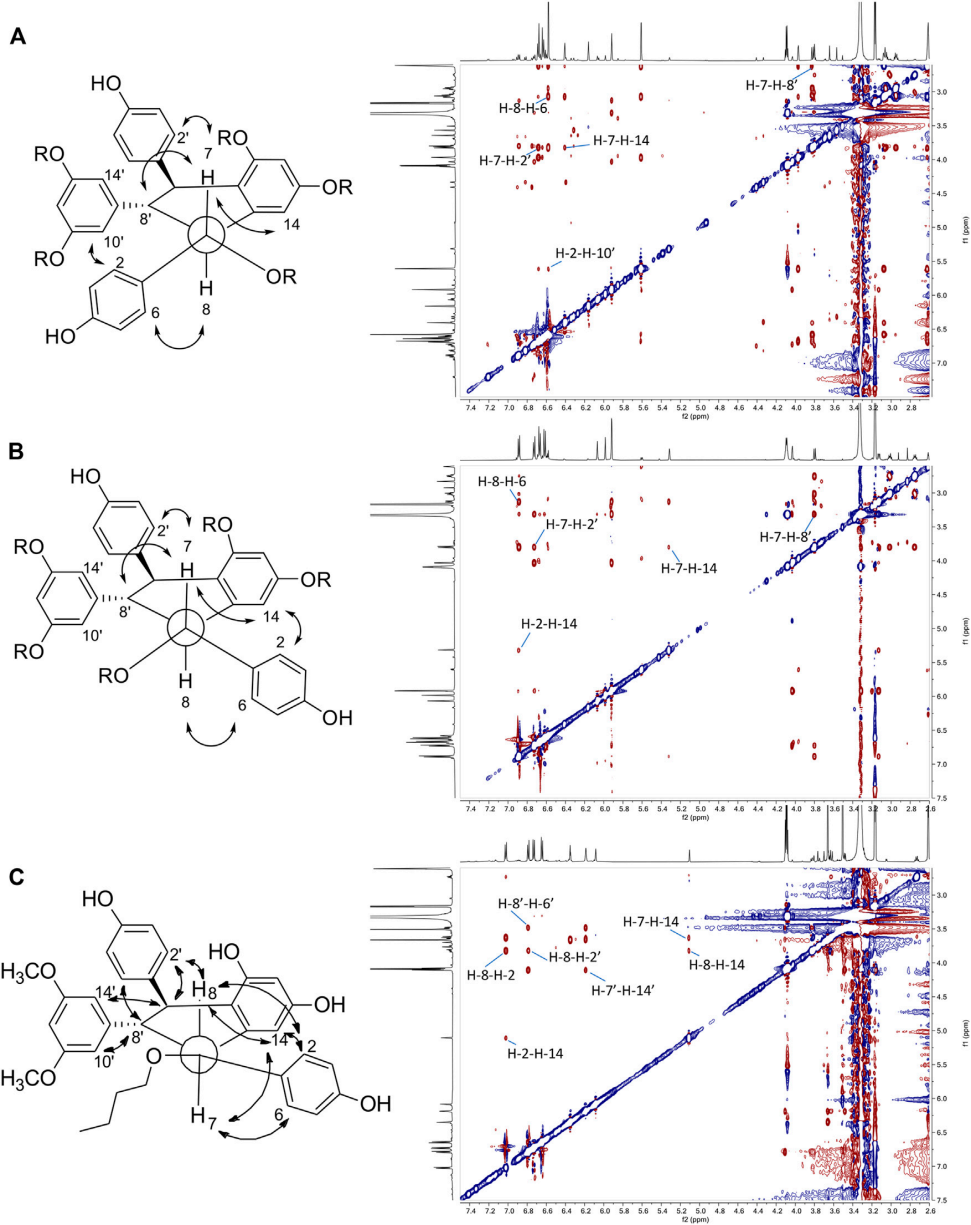
Key ROESY correlations observed for **(A)** leachianol F series **(B)** leachianol G series and **(C)** for the different isomer (compound **32**).

The fifth series is called leachianol, resulting from a 8-8′ coupling followed by a 10-7′ cyclization and a nucleophilic addition of the alcohol in C-7 (Figure 4). The ^1^H NMR spectrum of this series was recognized by the lack of doublets corresponding to the *trans* double bond, the presence of 4 doublets (2H each) with ortho coupling (*J* = 8.3–8.6 Hz) from the 2 para-disubstituted rings, and a triplet (1H)/doublet (2H) with meta coupling (*J* = 2.1–2.3 Hz) from the trisubstituted ring and especially the presence of four methines signals from H-7, H-8, H-7′ and H-8′ (Figure 5). Two diastereoisomers can be formed during the dimerization process and were called F and G regarding the configuration of leachianol F and G isolated first by Ohyama et al. (1995) from the roots of *Sophora leachiana*. The distinction between these two diastereoisomers can be made by NMR first by comparing the ^1^H chemical shifts of the aromatics H-14 and H-10′/H-14′ and the methine H-8′. Due to the proximity of the phenol in C-7 in the case of the F diastereoisomer, H-8′ and H-10′/H-14′ are shifted to a higher field (δ_H_ 2.59–2.76 for H-8′ and 5.59–5.73 for H-10′/H-14′) than that in the G diastereoisomer (δ_H_ 3.20–3.46 for H-8′ and 5.92–6.12 for H-10′/H-14′) (Figure 6). In the case of the G diastereoisomer, the phenol group is on the side of H-14 which is shielded compared to F diastereoisomer (δ_H_ 5.17–5.57 and 6.41–6.75 for G and F diastereoisomer, respectively). The ROE correlation from H-2/H-6 to H-10′/H14′ in the case of F diastereoisomer and to H-14 in the case of G diastereoisomer confirmed this assignment (Figure 6). Among the 26 compounds of this leachianol series, parthenostilbenin A and B (**23**), already isolated by Kim et al. (2005) from *Parthenocissus tricuspidata* were isolated as a mixture and quadrangulain B already isolated from *Cissus quadrangularis* by Adesanya et al. (1999) was also produced from this biotransformation reaction (**71**). Compound **32** could be considered apart as this was the only compound with a different configuration of the cyclopentane ring. The ROE correlations from H-7′ to H-10′/H14′ and from H8′ to H-2′/H-6′ indicated a *trans* configuration of H-7′ and H-8′ protons, the correlation from H-8 to H-2′/H-6′ indicated a *cis* configuration of H-8 and H-8′ protons and the correlation from H-2/H-6 to H-14 allowed to define the C-7 configuration (Figure 6). Finally, 23 compounds from this series are described here for the first time.

Compound **54** belongs to a new scaffold (sixth series) already produced and described from previous biotransformation reactions and named resvepterodimer A (Righi et al., 2020). No other derivatives belonging to this series have been isolated, probably due to steric hindrance caused by the longer carbon chains of solvents other than methanol.

In summary, the biotransformation reactions performed with resveratrol and pterostilbene in five different alcoholic solvents allowed the isolation of 73 derivatives distributed among 6 scaffolds, 55 being described here for the first time (Figure 3).

**FIGURE 7:**
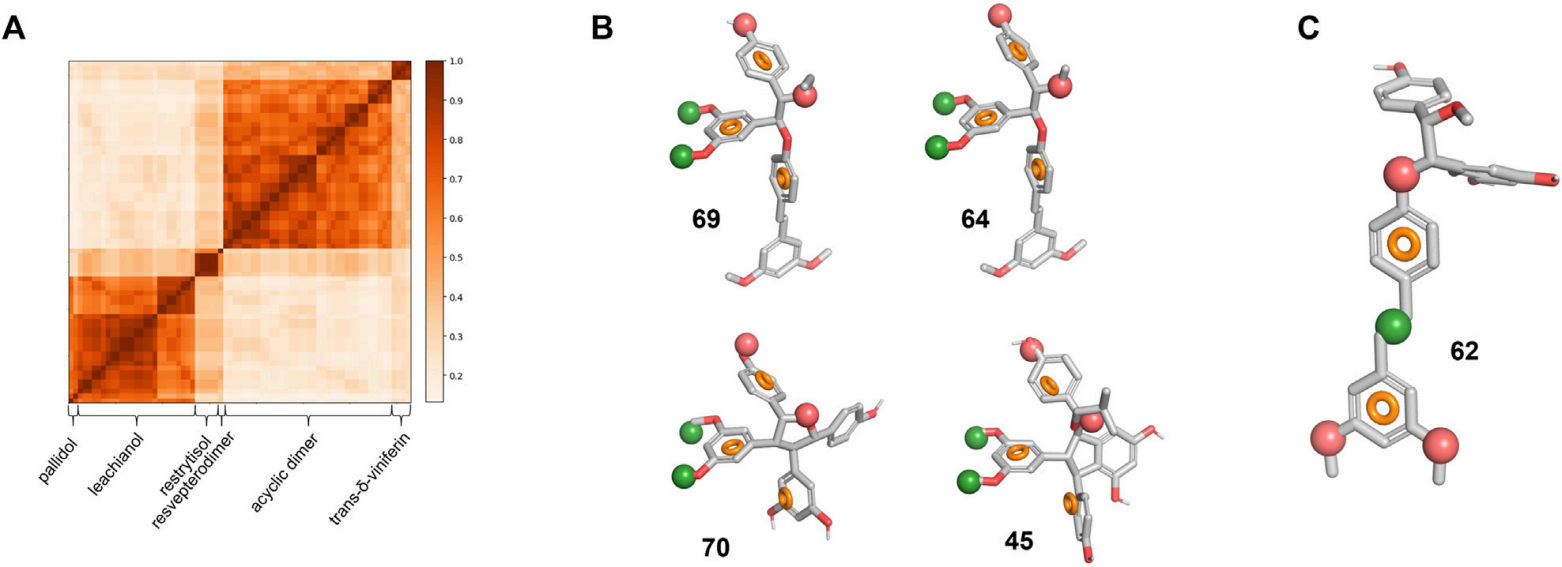
Library chemical similarity and pharmacophore models. **(A)** Similarity matrix for all six series. A value of 1 corresponds to the identity. **(B)** Pharmacophore model for specific actives overlapped on the four compounds with an SI > 10. **(C)** Pharmacophore model for non-specific actives overlapped on **62**. The red and green spheres represent respectively an H-bond acceptor and a hydrophobic region. The orange circle represents the ideal 3D positioning of aromatic rings.

To understand further how different or similar compounds are, we measured the Tanimoto coefficient and represented it in the form of a similarity matrix (Figure 7A). The analysis resulted in defined clusters corresponding to each series of compounds. Most clusters exhibit coefficient value superior than 0.7, showing the similarity within the clusters and the presence of a common scaffold. In contrast, when comparing the scaffolds of the different clusters to each other, the analysis reveals diversity with value lower than 0.5 indicating that the biotransformation methodology is able to deliver chemically diverse scaffolds starting from the same material.

### Biotransformation Leads to Novel Derivatives With Specific Activity Against the Wnt Pathway in Triple-Negative Breast Cancer

Our main building blocks resveratrol and pterostilbene have previously been reported to inhibit Wnt signaling in breast cancer (Wang et al., 2012) and inhibit or activate the pathway in many other cancers or contexts (Ashrafizadeh et al., 2020). On the other hand, both compounds are well known to be highly promiscuous and are defined as pan-assay interfering compounds (PAINS) (Baell and Walters, 2014). Thus, we first investigated if these principal building blocks might show specific anti-Wnt activity in our assay and thus confer such activity to the various derivatives generated. We analyzed activation of the TopFlash Wnt-specific reporter in the TNBC cell line BT-20 by purified Wnt3a simultaneously measuring the constitutively expressed *Renilla* luciferase activity as a readout of unspecific and/or cytotoxic action of compounds. Our results (Supplementary Figure S5) indicate that resveratrol possesses only certain unspecific cytotoxic effects at concentrations exceeding 100 μM, while pterostilbene did not show any activity.

**FIGURE 8:**
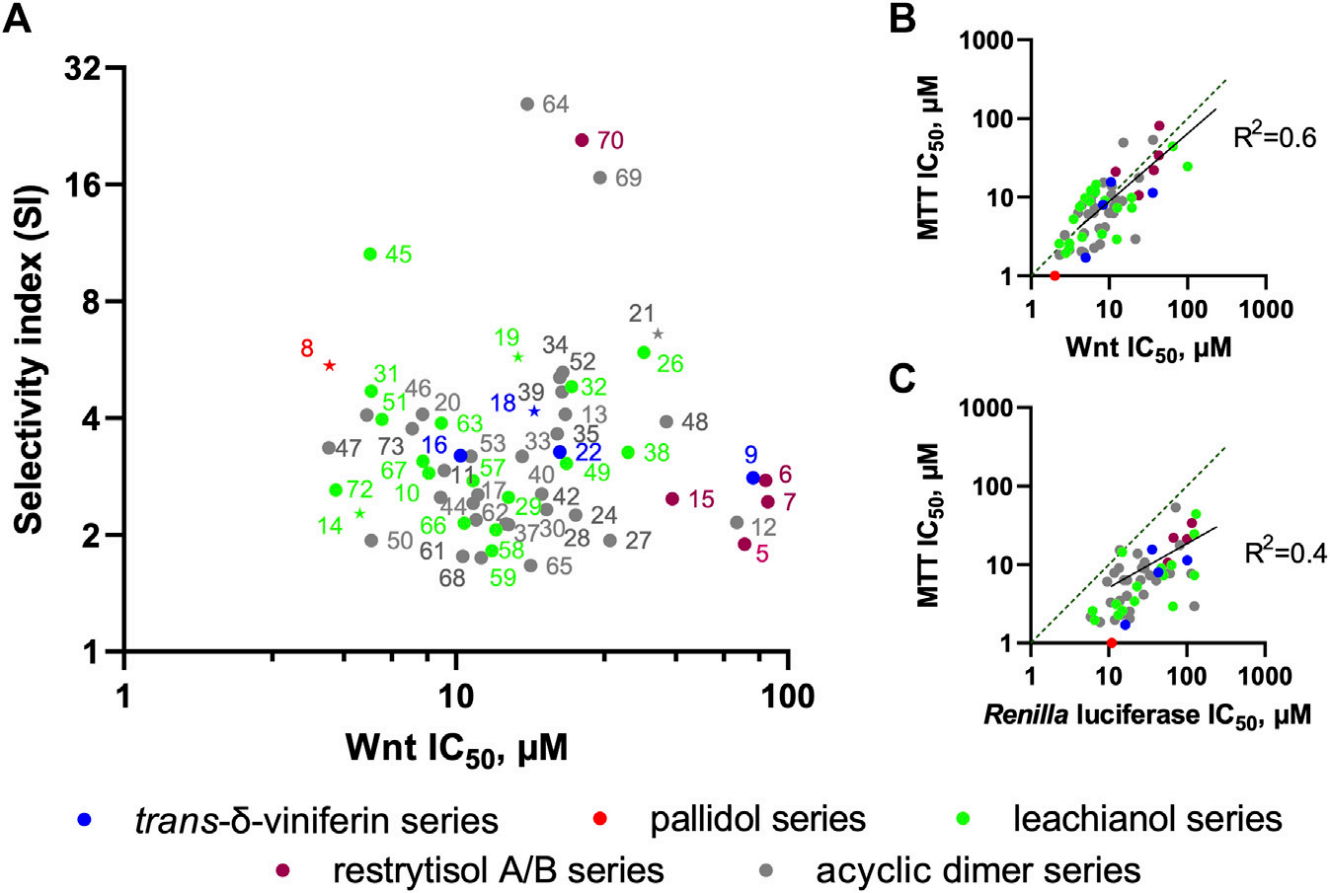
Relations between derivative scaffolds and anti-Wnt activity. **(A)** Distribution between the selectivity index (SI) and anti-Wnt potency of the investigated compounds. The compounds selected for further biochemical analysis are highlighted as stars **(B)** Correlation between anti-Wnt IC_50_ and IC_50_ of long-term (3 days) inhibition of the cell growth measured by MTT assay **(C)** Correlation between anti-Wnt IC_50_ and IC_50_ of acute cytotoxicity.

We further tested all the derivatives **1–73** in the same assay, discovering that the biotransformation of resveratrol/pterostilbene results in a large family of compounds with specific anti-Wnt properties (Table 1, columns “Wnt” and “*Renilla*”). The most interesting derivatives are highlighted by plotting the selectivity index (SI), expressed as a ratio of *Renilla* and Wnt IC_50_s, against the compound potency for the Wnt pathway inhibition (Figure 8A). We saw a general loose trend for increasing pathway inhibition selectivity with increased potency (i.e., with the decreased IC_50_). The three most selective compounds (**64**, **69,** and **70**), two belonging to the *threo* acyclic dimers series (**64** and **69**) both resulting from two pterostilbene units and one being the 11′,13′-di-*O*-methylrestrytisol B (**70**), demonstrate only average potency (18–27 µM Wnt IC_50_). It should be noted, that this analysis also showed the absence of a well-defined dependency of the compound potency or selectivity on a particular scaffold, with most of their representatives demonstrating activity within a broad range of values. However, some trends were observed by representing the data from Figure 8A by scaffold and mapping information such as the nature of the monomers (Supplementary Figures S6A–D). The restrytisol series shows little potency (>20 µM for the best compound). The leachianol, pallidol and viniferin series have broad activity values, spanning from high (<5 µM) to no activity. A general trend for these scaffolds is a lower activity for the dimers resulting from the coupling of two resveratrol units. The presence of methoxy groups (coming from the pterostilbene unit) therefore seems to impact positively the ability to inhibit the Wnt activity. Moreover, mixed dimers resulting from the coupling of a resveratrol and pterostilbene unit generally have the highest activities in the leachianol series. For this series with a clear dependence on the nature of the monomers, the influence of the side-chain coming from the solvolysis was also investigated (Supplementary Figure S6E) but did not show any clear tendency. For the acyclic dimer series, no visible trend between structure and activity was observed, probably due to the high flexibility of this scaffold. For all the scaffolds, no clear difference in potency or selectivity was observed between the stereoisomers (leachianol F or G; acyclic dimer *erythro* or *threo*; restrytisol A or B).

**TABLE 1:** IC_50_s of the investigated compounds against Wnt pathway and breast cancer cell survival.

Group	Compound ID	IC_50_, µM						
		Pathway specificity			3-day survival (MTT)			
		Wnt	*Renilla*	SI	BT-20	HCC1395	MDA-MB-231	MDA-MB-468
*trans*-δ-viniferin	**16**	10 ± 1	33 ± 17	3.2	3.7 ± 0.4	3.3 ± 0.4	3.1 ± 0.6	3.9 ± 0.2
	**18**	18 ± 2	77 ± 19	4.2	17 ± 2	16 ± 4	13 ± 2	19 ± 2
	**22**	22 ± 6	71 ± 6	3.3	31 ± 4	33 ± 8	29 ± 6	27 ± 6
	**9**	79 ± 13	∼222^a^	2.8	26 ± 2	29 ± 4	22 ± 2	24 ± 4
Pallidol	**8**	4.6 ± 0.6	25 ± 2	5.5	2.5 ± 0.2	1.5 ± 0	2.3 ± 0.2	2.1 ± 0.2
	**1**	Inactive^b^	Inactive^b^		172 ± 51	222 ± 97	165 ± 55	224 ± 114
leachianol F/G	**72** (F)	4.4 ± 1.1	11 ± 2	2.6	5.7 ± 0.8	4.9 ± 0.8	5.7 ± 0.8	4.7 ± 0.8
	**14** (G)	5.5 ± 0.6	13 ± 1	2.3	3.9 ± 0.2	3.3 ± 0.4	4.4 ± 0.2	2.8 ± 0.4
	**31** (G)	5.7 ± 0.4	27 ± 7	4.7	5.6 ± 0.2	4.1 ± 0.4	4.7 ± 0.5	4.3 ± 0.4
	**67** (G)	7.9 ± 1.1	25 ± 2	3.1	17 ± 2	13 ± 2	16 ± 2	14 ± 2
	**10** (F)	8.3 ± 0.7	24 ± 4	2.9	7 ± 0.7	4.4 ± 0.2	5.9 ± 0.6	5.7 ± 0.6
	**63** (F)	9 ± 3.1	35 ± 6	3.9	28 ± 4	14 ± 2	14 ± 3	15 ± 2
	**45** (G)	9.3 ± 0.7	99 ± 14	10.6	4.7 ± 0.4	3.8 ± 0.2	4 ± 0.4	4 ± 0.2
	**51** (F)	9.9 ± 1	39 ± 9	4.0	11 ± 1	8.6 ± 0.3	10 ± 0	6.5 ± 0.9
	**57** (F)	11 ± 2	31 ± 4	2.8	25 ± 4	14 ± 2	21 ± 2	16 ± 2
	**66** (F)	11 ± 2	23 ± 2	2.1	19 ± 2	16 ± 2	18 ± 2	14 ± 2
	**58** (F)	13 ± 2	27 ± 4	2.1	37 ± 4	21 ± 2	21 ± 2	21 ± 2
	**59** (G)	13 ± 2	23 ± 4	1.8	23 ± 4	15 ± 3	19 ± 4	12 ± 3
	**29** (F)	14 ± 1	36 ± 4	2.5	5.7 ± 0.4	5.2 ± 0.9	7.5 ± 0.4	5.9 ± 0.7
	**19** (G)	14 ± 1	82 ± 11	5.7	14 ± 1	12 ± 1	13 ± 1	23 ± 4
	**32** (G)	20 ± 4	95 ± 20	4.8	5.7 ± 0.9	4.5 ± 0.5	5.4 ± 0.7	5.4 ± 0.7
	**49** (G)	29 ± 22	89 ± 65	3.1	14 ± 1	11 ± 1	11 ± 1	15 ± 1
	**38** (F)	32 ± 5	106 ± 34	3.3	27 ± 3	14 ± 2	13 ± 2	13 ± 1
	**26** (F)	40 ± 11	∼235	5.9	17 ± 4	10 ± 2	14 ± 3	13 ± 2
	**36** (G)	115 ± 48	∼227^a^	2.0	87 ± 15	63 ± 12	91 ± 19	63 ± 9
	**41** (G)	>236^b^	non-toxic^b^		>473^a^	>473^a^	>473^a^	>473^a^
	**25** (G)	inactive^b^	non-toxic^b^		>473	>473	>473	>473
	**71** (G)	inactive^b^	non-toxic^b^		Inactive^b^	Inactive^b^	Inactive^b^	Inactive^b^
	**23** (F + G)	inactive^b^	non-toxic^b^		>513	>513	>513	>513
	**4** (G)	Toxic^c^	>243		51 ± 12	45 ± 16	52 ± 17	43 ± 14
	**43** (F)	Toxic^c^	6.1 ± 0.4		1.8 ± 0.4	1.1 ± 0.2	1.4 ± 0.2	1.4 ± 0.2
resvepterodimer	**54**	Inactive^b^	non-toxic^b^		Inactive^b^	Inactive^b^	Inactive^b^	Inactive^b^
acyclic dimers	**47** (*erythro*)	4.1 ± 0.2	14 ± 2	3.3	3.8 ± 0.4	3.2 ± 0.4	3.2 ± 0.5	3.1 ± 0.5
	**73** (*erythro*)	5.4 ± 1	22 ± 2	4.1	7 ± 1	6.8 ± 1	8.2 ± 1.2	6.8 ± 1
	**50** (*threo*)	5.6 ± 0.5	11 ± 1	1.9	3.8 ± 0.2	3.4 ± 0.2	4.9 ± 0.4	3.4 ± 0.2
	**20** (*threo*)	7.4 ± 0.6	28 ± 4	3.8	9.6 ± 0.4	11 ± 2	16 ± 1	9.6 ± 0.7
	**46** (*threo*)	7.9 ± 0.7	32 ± 9	4.1	4.1 ± 0.4	3.2 ± 0.2	3.2 ± 0.4	4.1 ± 0.4
	**44** (*threo*)	9.1 ± 0.6	23 ± 4	2.5	4.2 ± 0.4	3.4 ± 0.2	3.8 ± 0.4	3.6 ± 0.4
	**61** (*erythro*)	10 ± 3	18 ± 2	1.8	17 ± 2	13 ± 2	8.6 ± 1.2	12 ± 1
	**62** (*threo*)	11 ± 2	27 ± 2	2.4	25 ± 2	15 ± 3	15 ± 2	18 ± 3
	**37** (*threo*)	11 ± 1	25 ± 5	2.2	4.5 ± 0.4	3.1 ± 0.2	4.9 ± 0.4	4 ± 0.2
	**11** (*erythro*)	11 ± 1	33 ± 10	2.9	8.2 ± 1	4.5 ± 0.4	7.2 ± 1	7 ± 0.4
	**17** (*threo*)	12 ± 1	29 ± 6	2.5	10 ± 1	9.2 ± 0.9	14 ± 3	13 ± 1
	**68** (*erythro*)	12 ± 3	21 ± 2	1.7	28 ± 4	14 ± 2	19 ± 2	19 ± 2
	**30** (*threo*)	14 ± 1	30 ± 4	2.1	8.7 ± 1.7	6.1 ± 0.9	11 ± 2	4.7 ± 0.6
	**28** (*erythro*)	15 ± 2	32 ± 6	2.1	5.7 ± 0.8	4.7 ± 0.8	4.5 ± 0.8	4.2 ± 0.4
	**33** (*threo*)	16 ± 1	50 ± 9	3.2	7.2 ± 0.9	7.7 ± 0.5	6.1 ± 1.4	8.6 ± 0.5
	**65** (*threo*)	17 ± 3	28 ± 4	1.7	46 ± 12	16 ± 3	26 ± 4	20 ± 4
	**64** (*threo*)	18 ± 5	>460	25.8	Inactive^b^	inactive^b^	inactive^b^	inactive^b^
	**53** (*threo*)	19 ± 3	60 ± 9	3.2	15 ± 2	8.9 ± 2.1	14 ± 3	12 ± 2
	**40** (*threo*)	19 ± 2	48 ± 10	2.5	19 ± 2	21 ± 3	17 ± 3	16 ± 3
	**42** (*erythro*)	19 ± 1	44 ± 9	2.3	13 ± 2	12 ± 2	11 ± 2	11 ± 2
	**35** (*erythro*)	20 ± 2	72 ± 13	3.6	15 ± 3	5.6 ± 0.7	13 ± 2	11 ± 1
	**39** (*erythro*)	21 ± 2	96 ± 68	4.7	15 ± 3	12 ± 1	11 ± 2	14 ± 2
	**13** (*threo*)	22 ± 2	90 ± 12	4.1	36 ± 6	20 ± 4	36 ± 6	20 ± 4
	**24** (*erythro*)	22 ± 2	50 ± 7	2.3	22 ± 4	12 ± 1	15 ± 2	18 ± 2
	**34** (*erythro*)	22 ± 4	110 ± 23	5.1	16 ± 2	12 ± 1	14 ± 2	14 ± 1
	**52** (*erythro*)	25 ± 8	130 ± 91	5.2	12 ± 2	12 ± 2	15 ± 4	13 ± 3
	**69** (*threo*)	27 ± 9	>449	16.7	Inactive^b^	104 ± 142	74 ± 54	Inactive^b^
	**27** (*erythro*)	30 ± 4	58 ± 58	1.9	20 ± 2	13 ± 1	20 ± 2	18 ± 2
	**21** (*erythro*)	36 ± 13	∼189^a^	6.6	7.4 ± 1.3	4.5 ± 0.4	5.1 ± 0.6	5.3 ± 0.8
	**48** (*erythro*)	43 ± 7	∼169^a^	3.9	41 ± 9	23 ± 13	38 ± 11	25 ± 13
	**12** (*threo*)	76 ± 23	∼163	2.2	266 ± 138	43 ± 17	60 ± 27	49 ± 17
	**2** (*threo*)	Inactive^b^	non-toxic^b^		233 ± 55	186 ± 68	195 ± 68	231 ± 102
	**55** (*erythro*)	Inactive^b^	non-toxic^b^		68 ± 12	47 ± 6	43 ± 6	39 ± 6
	**56** (*threo*)	toxic^c^	33 ± 4		43 ± 6	27 ± 4	33 ± 4	29 ± 4
	**60** (*threo*)	toxic^c^	19 ± 10		21 ± 4	14 ± 3	17 ± 3	21 ± 4
	**3** (*erythro*)	Toxic^c^	∼212^a^		174 ± 40	157 ± 49	131 ± 28	142 ± 34
restrytisol A/B	**70**	24 ± 4	>500^a^	20.8	48 ± 6	40 ± 6	34 ± 6	36 ± 6
	**15**	44 ± 8	108 ± 26	2.5	28 ± 4	21 ± 4	19 ± 2	14 ± 1
	**5**	74 ± 8	140 ± 60	1.9	64 ± 18	18 ± 4	48 ± 14	46 ± 4
	**6**	84 ± 20	∼231^a^	2.8	72 ± 18	44 ± 16	102 ± 48	50 ± 14
	**7**	88 ± 18	∼214^a^	2.4	194 ± 34	128 ± 20	142 ± 28	182 ± 54

a The value was estimated approximately due to its proximity to the limits of the concentration range.

b Compounds are considered as inactive and non-toxic if if the highest concentration tested does not show a 20% reduction compared to the control.

c Compound is considered “toxic” if the IC_50_ value against *Renilla* luciferase is less than 1.7-fold of estimated TopFlash one (i.e., SI < 1.7), indicating that the decrease observed in TopFlash signal is due to or strongly affected by toxic effect.

To identify key features that distinguish actives from inactive compounds in 3D space, we developed a pharmacophore model hypothesis. Pharmacophore models consider common electronic and shape features that would otherwise be difficult to assess in 2D space. The compounds were first split in three groups based on their SI and IC_50_ (Supplementary Figure S7A): we defined the “specific-active” group for compounds with a high selectivity index (>10) and a low IC_50_ (<35 μM, 4 compounds in total), the “low specificity-active” group (SI < 10, IC_50_ < 35 μM, 46 compounds in total) and the “low specificity-low activity” group (SI < 10, IC_50_ > 35 μM, 18 compounds in total). As the stereochemistry of these compounds is relative and not absolute, the enantiomers for each compound were considered to build the model. The pharmacophore analysis of the “specific-activity” group lead to a seven-features model that overlap with the asymmetric centers part of **64** and **69** from the acyclic dimer series (Figure 7B). This model describes also key features of **70** from the restrytisol series and **45** from the leachianol series (Figure 7B). To validate and assess the predictiveness of this model, we analyzed how the whole library fits to the model. Besides the true positives, false positive compounds from the acyclic dimer series appeared (Supplementary Figure S7B). The overlap of the model on false positives as well as the absence of excluded volumes in the model suggests additional requirements for a specific activity: the acyclic dimer must be in a *threo* and not in an *erythro* configuration, and the substituent in R3 must be smaller than an ethyl group and not branched.

The model derived from the “low specificity-active” compounds describes the pterostilbene moiety when overlapped with the acyclic dimer series such as compound **62** (Figure 7C). This model has to be considered as descriptive but non-predictive as shown by the poor correlation between the IC_50_ and the fitness (Supplementary Figure S7C). The two “specific-activity” compounds from this series (**64** and **69**) fit to this model, leading to the hypothesis that their IC_50_ might be the result of both unspecific and specific activity (Supplementary Figure S7C). Finally, the analysis of the “low specificity-low activity” group did not generate any model. This suggests that their low activity is not linked to a particular structure but rather a lack of the key features depicted in the model built on the “specific-active” compounds (Figure 7B) or the presence of substituents in hypothetical excluded volumes. It should be noted that some “low specificity-low activity” compounds still display features from the “low specificity-active” model.

Although these pharmacophore models suffer from limitations such as lack of excluded volumes, they rationally explain how different backbones can display the same activity and emphasize the crucial role of stereochemistry in natural products. The models built on defined groups identified discriminative key features for the specificity and descriptive common features for the activity. They lay the ground for analog synthesis or virtual screening.

We next analyzed whether these compounds are capable of inhibiting the proliferation of TNBC cell lines (BT-20, HCC1395, MDA-MB-231, and MDA-MB-468). Indeed, as reported in the corresponding columns of Table 1, the majority of the compounds efficient against the Wnt pathway were also excellent inhibitors of cancer cell proliferation. However, since the majority of the compounds demonstrated an unspecific inhibition of *Renilla* luciferase, which might be both manifestation of direct cytotoxicity or other interference with the cell well-being leading to the decrease in proliferation as well, we wanted to test independently whether this activity is indeed attributable to Wnt signaling.

To this end, we analyzed the relationships between the observed effect in MTT assay (taken as an average value of IC_50_s across all 4 TNBC cell lines, which are very close) and the potency of the compounds against the Wnt pathway (Figure 8B) and the *Renilla* luciferase reporter (Figure 8C). It is clearly evident that the long-term proliferation IC_50_s correlate much better (linear fit R^2^ = 0.6) with these against the Wnt signaling than with the *Renilla* reporter (R^2^ = 0.4. Data distribution indicates a relatively good linear correlation between anti-Wnt activity and long-term inhibition activity, while this correlation for *Renilla* is strongly right-shifted, indicating that, in general, the IC_50_s obtained from the MTT assay are lower. It should be also noted, that IC_50_ values for some compounds are either lower or higher than those obtained against Wnt signaling, which can be attributed to the different lengths of the assays, and thus effects of the tested compounds stability or subcellular accumulation might influence these values.

**FIGURE 9:**
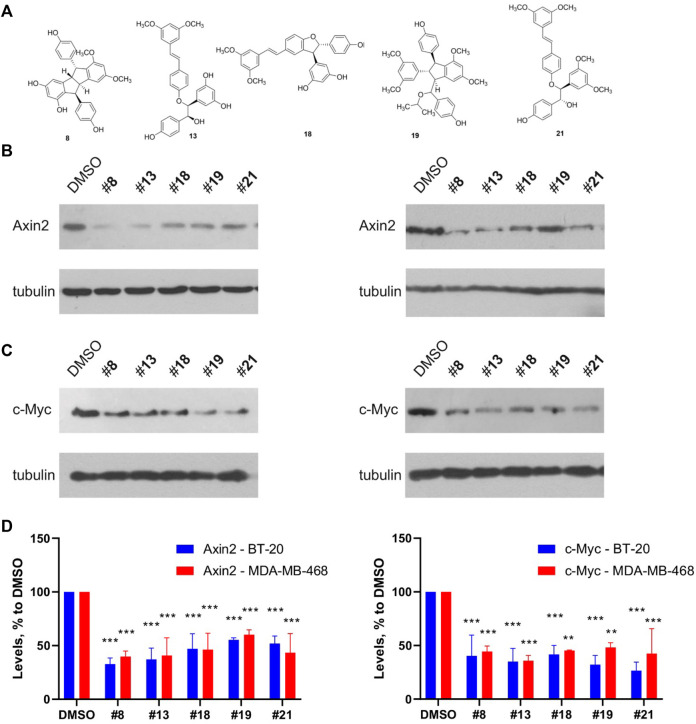
Representative compounds from the different structural groups of the derivatives **(A)** inhibit expression of the basal levels of Wnt target genes Axin2 **(B)** and c-Myc **(C)** in the TNBC cell lines BT-20 (left) and MDA-MB-468 (right). Quantification of the Western blots is presented on **(D)**. Statistical significance was assessed by two-way ANOVA followed by multiple comparisons with DMSO-treated values, *p* values are shown as **p < 0.01, ***p < 0.001.

We also confirmed the Wnt inhibitory activity of the compounds in a different assay from TopFlash. A set of five representative compounds with different scaffolds was chosen on the grounds of both addressing the most prominent structural families and capacity to produce a sufficient quantity of the compound. We have analyzed this set for its ability to inhibit basal levels of the Wnt target genes Axin2 and c-Myc (Figure 9) (Koval et al., 2018; Xu et al., 2020). All the tested compounds demonstrated the capacity to decrease both target genes expression with slightly different efficacies. These levels of the Wnt target genes inhibition are in line with our and other’s findings, e.g., for clofazimine (Ahmed et al., 2019; Xu et al., 2020), a compound capable of suppressing tumor growth *in vitro* and *in vivo*.

We next aimed at dissecting the potential mechanism of compounds’ activity within the Wnt pathway, analyzing whether the compounds are capable of inhibiting the signaling upstream or downstream of its symbolical “watershed”—the β-catenin destruction complex. For this purpose, we assessed the levels of β-catenin induced in L-cells (murine fibroblasts) by addition of Wnt3a (Koval et al., 2014; Koval et al., 2018). These cells have virtually no endogenous β-catenin, but it starts to accumulate rapidly upon the addition of the Wnt ligand Wnt3a. However, the compounds were not able to suppress this effect (Supplementary Figures S8A,B). At the same time, they were capable to suppress the Wnt pathway when it was stimulated by CHIR99021, a specific inhibitor of GSK3β kinase which can strongly stimulate the pathway downstream of Wnt3a ligand (Supplementary Figures S8C,D) (An et al., 2010). These results indicate that the target of the compound is located well downstream of the destruction complex.

We also assessed whether the compounds might affect two other important steps in Wnt signaling—translocation of β-catenin to the nucleus and its phosphorylation. To this end, we performed double staining of total and phosphorylated β-catenin in the BT-20 and MDA-MB-468 TNBC cell lines, stimulated or not by Wnt3a and treated by the compound **8** on the background of Wnt3a activation. Accumulation of the total β-catenin in the cytoplasm and nucleus of many cells is evident upon treatment with Wnt3a, but this effect was not reversed by addition of the compound **8** (Supplementary Figure S9). Similarly, in the MDA-MB-468 cells addition of Wnt3a result in a visible strong reduction of phosphorylated β-catenin, and this effect is again unsusceptible to the action of the compound **8**.

With the strong inhibition of the target genes that we observe, we can therefore conclude that the Wnt inhibitory activity of the compounds studied is probably due to interference with the nuclear effectors of the pathway and thus with Wnt-induced transcription. Considering that selected compounds represent all the main scaffolds produced by biotransformation, this mechanism of action can be extrapolated to the entire set of the active compounds reported here.

## Conclusion

Biotransformation reactions of the mixture of pterostilbene and resveratrol with the *B. cinerea* secretome generated a large diversity of 73 dimeric stilbene analogs organized into six scaffolds. All compounds were obtained by dimerization of the stilbene units by coupling of phenoxy radicals generated by oxidation with laccases (Langcake and Pryce, 1977; Keylor et al., 2015). The use of five different organic solvents in the biotransformation reactions enriched the chemodiversity of the library generated. These compounds (representing 78% of the library) were produced by a nucleophilic solvent attack (solvolysis) on a *sp* ^*2*^ carbon of an intermediate structure (Figure 4) (Righi et al., 2020).

The structural sources of these derivatives (resveratrol and pterostilbene) were reported to both inhibit or activate Wnt pathway in various contexts, including cancers, such as gastric (Aquilano et al., 2009), lung (Langcake and Pryce, 1977), colon (Hope et al., 2008; Khan et al., 2020), endometrial (Wang et al., 2012; Mineda et al., 2019), and breast (Wang et al., 2012). Typically for PAINS (Pan-Assay INterfering compoundS), no clear target was identified, and likely the observed effects on Wnt pathway are the results of interaction with multiple targets and overlapping of various mechanisms of action. Such effects obviously depend on the context and the exact way of stimulating the pathway, thus we first verified to which extent our source compounds might inhibit Wnt signaling induced in BT-20 TNBC cells by addition of ligand in order to take into account inheritance of the same unspecific activity trend by the derivatives. Our results demonstrate no specific interference of the resveratrol and pterostilbene with Wnt3a-induced activity in TNBC cells.

The generated derivatives obtained by the enzymatic biotransformation of these two stilbenoids, however, result from a significant modification of the structures of the original compounds. None of the classes of the identified derivatives are described as PAINS, and overall, one might expect that the structural rearrangements in the derived molecules will result in the loss of the main part of the PAINS features of the original compounds—such as high reactivity, low structure complexity and ability to interfere with the redox balance, features that lead to the promiscuity due to oxidation and etherification. We also identified that none of the classes of investigated derivatives was reported as Wnt inhibitor before.

Nevertheless, despite the extent of structural variation across the large set of compounds studied, we identified that the anti-Wnt activity of the derivatives is not restricted to any particular scaffold, although some trends can be observed. It should be stressed that certain chemical scaffolds, such as the pallidol, the viniferin and the restrytisol series are represented by only one or several derivatives with results highly divergent among them and thus provide us only very limited information about their potential as Wnt inhibitors. The leachianol and acyclic dimer series have many more representatives and are therefore more adapted to in-depth analysis.

The acyclic dimer series shows little or no structure-activity relationship. Indeed, neither the nature of the monomers nor the *erythto*/*threo* configuration properly correlates with the observed IC_50_s. Two compounds from this series stand out concerning their selectivity indexes (**64** and **69**). Both are pterostilbene dimers and have a *threo* configuration. However, other derivatives with the same characteristics (**40** and **53**) have much lower SI values. This lack of structure-activity relationship could be explained by the high flexibility of this scaffold compared to the other ones. The absence of cyclization during the dimerization process leads to a high number of free-rotating bonds and therefore to a higher diversity of conformers.

A clearer correlation between structure and activity can be observed for the leachianol series. In general, the resveratrol dimers show the lowest activities, whereas the resveratrol-pterostilbene dimers show the highest ones. This could indicate that the presence of one more apolar side (with methoxy groups) is favorable to the interaction with the target. We however couldn’t correlate the observed activities within this scaffold with the F or G configuration, neither with the type of side chain introduced by the solvent. The pharmacophore models did not give further insight for this series in particular.

The in-depth investigations of the potential mechanism of action of these derivatives suggest that the compounds act on the transcriptional machinery associated with the Wnt pathway. The determination of the exact target protein(s) is clearly a task for future work and is expected to explain why so structurally divergent derivatives are all capable of specific inhibition of the pathway. Currently, it can be speculated in light of the different pharmacophore models that this is due to the presence of highly similar structural determinants in these compounds, present in optimal positions on different backbones. A significant input in activity can be also expected from differential stability, permeability and compartmentalization of the compounds, which is especially affected by the solvent-derived moieties.

## Experimental Section

### General Experiment Procedures

The UV spectra were recorded on an Agilent Cary 60 UV-Vis (Santa-Clara, CA, United States) spectrometer in MeCN, using a 1 cm cell. ECD spectra were recorded on a JASCO J-815 spectrometer (Loveland, CO, United States) in MeCN, using a 1 cm cell. NMR data were recorded on a Bruker Avance Neo 600 MHz NMR spectrometer equipped with a QCI 5 mm cryoprobe and a SampleJet automated sample changer (Bruker BioSpin, Rheinstetten, Germany). 1D and 2D NMR experiments (^1^H, COSY, ROESY, HSQC, and HMBC) were recorded in DMSO-*d*_6_. Chemical shifts are reported in parts per million (δ) and coupling constants (*J*) in Hz. The residual DMSO-*d*_6_ signal (δ_H_ 2.50; δ_C_ 39.5) were used as internal standards for ^1^H and ^13^C NMR, respectively. Resveratrol (99% purity) and pterostilbene (99% purity) were purchased from Biopurify Phytochemicals Ltd. (Chengdu, China).

### Secretome Isolation From *Botrytis cinerea* Culture

The *B. cinerea* Pers., isolate K16, was obtained from naturally sporulated grape berries from the Changins Agroscope experimental vineyards in 2015. The strain was purified, determined phenotypically as well as by molecular tools (sequencing of the ITS regions), and maintained by regular transplanting. The fungus was grown on oatmeal agar medium (Difco), and conidia were sampled by vacuum aspiration and stored dry at −80°C until use. *B. cinerea* was cultivated in 1.5 L liquid medium (in 5 L bottles) at 22°C under alternating light and dark (12 h/12 h). The medium was filtrated through a double layer of folded filter paper (diameter 500 mm, Prat Dumas, France). The filtrate was brought to 80% saturation [(NH_4_)_2_SO_4_, 24 h at 4°C] and centrifuged (4,200 g, 4°C, 2 h). The supernatant was discarded, and the resulting pellet was solubilized in nanopure water (Evoqua Water Technologies, 4.21 μS cm^−1^) (the protein crude extract) and submitted to a dialysis (Spectra/Por 1 dialysis membrane, 6–8 kDa, diameter 14.6 mm) against nanopure water overnight at 4°C. The resulting protein extract was concentrated against polyethylene glycol beads (PEG 20,000) in the dialyzed tube. The protein content was determined by the method of Bradford, using a Bio-Rad protein assay kit with BSA as a standard. The volume of the extract was adjusted to obtain a final protein concentration of 2 μg/μl. The resulting extract (considered as secretome) was aliquoted to 1 ml and stored at −20°C until use.

### Biotransformation of the Resveratrol/Pterostilbene Mixture at Analytical Scale

The biotransformation reactions of the resveratrol/pterostilbene mixtures at analytical scale were performed in 2 ml Eppendorf tubes. The conditions were kept constant for each reaction as follows: 1 ml of total volume, 1 mg/ml of total substrate concentration (0.5 mg/ml of each compound), 50% of alcohol (*i*-PrOH, *n*-BuOH, *i*-BuOH, MeOH or EtOH, 500 µl), 48% of water (480 µl), and 2% of secretome (20 µl). Once prepared, the mixtures were incubated at room temperature in the dark under constant gentle agitation. Reactions were stopped after 24 h by removing the solvent under vacuum in a centrifugal evaporator. 1 ml of MeOH was then added on the dry deposit. The sample was centrifugated and the supernatant was analyzed by UHPLC-PDA-ELSD-MS.

### UHPLC-PDA-ELSD-MS Analysis

The crude reaction mixtures were analyzed on an Ultra-high-performance liquid chromatography system equipped with a photodiode array, an evaporative light-scattering detector, and a single quadrupole detector using heated electrospray ionization (UHPLC-PDA-ELSD-MS) (Waters, Milford, MA, United States). The ESI parameters were the following: capillary voltage 800 V, cone voltage 15 V, source temperature 120°C, and probe temperature 600°C. Acquisition was done in negative ionization mode (NI) with an *m/z* range of 150–1,000 Da. The chromatographic separation was performed on an Acquity UPLC BEH C18 column (50 × 2.1 mm i.d., 1.7 μm; Waters) at 0.6 ml/min, 40°C with H_2_O (A) and MeCN (B) both containing 0.1% formic acid as solvents. The gradient was carried out as follow: 5–100% B in 7 min, 1 min at 100% B, and a re-equilibration step at 5% B for 2 min. The ELSD temperature was fixed at 45°C, with a gain of 9. The PDA data were acquired from 190 to 500 nm, with a resolution of 1.2 nm. The sampling rate was set at 20 points/s. Every data was processed with the MassLynx software (Waters, Milford, MA, United States).

### UHPLC-PDA-CAD-HRMS Analysis

The pure compounds were analyzed on a Waters Acquity UHPLC system equipped with a Q-Exactive Focus mass spectrometer (Thermo Scientific, Bremen, Germany), using heated electrospray ionization source (HESI-II). The chromatographic separation was carried out on an Acquity UPLC BEH C18 column (50 × 2.1 mm i.d., 1.7 μm; Waters) at 0.6 ml/min, 40°C with H_2_O (A) and MeCN (B) both containing 0.1% formic acid as solvents. The gradient was carried out as follow: 5%–100% B in 7 min, 1 min at 100% B, and a re-equilibration step at 5% B in 2 min. The ionization parameters were the same as used in Rutz et al. (2019).

### Biotransformation of the Resveratrol/Pterostilbene Mixture at Large Scale

The biotransformation reactions at large scale were performed by keeping the same parameters as the analytical scale (substrate concentration, solvent and secretome percentages) but increasing the amount of starting materials. Typically, reactions were performed in Schott bottles of 200–500 ml using 200–400 mg of starting material (resveratrol + pterostilbene). Substrates were first dissolved in 100–200 ml of alcohol (*i*-PrOH, *n*-BuOH, *i*-BuOH, MeOH or EtOH), water was then added (50% of the total volume) and the secretome volume (2% of the total volume) was added last. This mixture was incubated for 24 h at room temperature in the dark under gentle magnetic stirring. After evaporation of the solvent with a rotatory evaporator, the dry deposit was resuspended in MeOH and filtered through 0.45 µm filters (13 mm Syringe filter, PVDF, BGB Analytik, Böckten, Switzerland). These crude reaction mixtures were analyzed by UHPLC-PDA-ELSD-MS for comparison with analytical scale reactions before moving to the gradient optimization and semi-preparative HPLC fractionation.

### Purification of the Crude Reaction Mixtures by Semi-preparative HPLC-UV

The separation conditions of the 5 crude reaction mixtures were optimized on the UHPLC-PDA-ELSD-MS system with an Acquity UPLC BEH C18 column (100 × 2.1 mm i.d., 1.7 μm; Waters) at 0.4 ml/min, 40°C with H_2_O (A) and MeOH (B) both containing 0.1% formic acid as solvents. The optimized gradient conditions for each reaction were geometrically transferred by gradient transfer to the analytical and semi-preparative HPLC scale (Supplementary Table S1, for each chromatographic conditions). The geometrically transferred gradients were first tested on a HP 1260 Agilent High-Performance liquid chromatography equipped with a photodiode array detector (HPLC-PDA) (Agilent technologies, Santa Clara, CA, United States). The chromatographic separation was performed on an XBridge C18 column (250 × 4.6 mm i.d., 5 μm; Waters) equipped with a C18 pre-column at 1 ml/min, with H_2_O (A) and MeOH (B) both containing 0.1% formic acid as solvents. The UV absorbance was measured at 210 and 254 nm and the UV-PDA spectra were recorded between 190 and 600 nm (step 2 nm). The geometrically transferred gradients were used at the semi-preparative scale on a Shimadzu system equipped with an LC-20 A module pumps, an SPD-20 A UV/VIS, a 7725I Rheodyne® valve, and an FRC-40 fraction collector (Shimadzu, Kyoto, Japan). The separation was performed on a XBridge C18 column (250 mm × 19 mm i.d., 5 μm; Waters) equipped with a C18 pre-column cartridge holder (10 mm × 19 mm i.d., 5 μm; Waters) at 17 ml/min, with H_2_O (A) and MeOH (B) both containing 0.1% formic acid as solvents. The UV detection was set at 210, 254 and 280 nm. The mixtures were injected on the semi-preparative HPLC column using a dry-load methodology developed in our laboratory (Queiroz et al., 2019).

### Biotransformation Reaction N°1 (With *i*-PrOH as Cosolvent)

Six semi-preparative HPLC dry-load injections of about 60 mg each yielded 60 fractions each. Fractions were analyzed by UHPLC-PDA-ELSD-MS and mixed together according to their composition. 24 compounds were obtained pure: **1** (7.3 mg, *t*_R_ 5.1 min), **23** (0.2 mg, *t*_R_ 6.9 min), **2** (7.7 mg, *t*_R_ 8.0 min), **3** (1.1 mg, *t*_R_ 9.2 min), **4** (6.6 mg, *t*_R_ 12.3 min), **5** (10.0 mg, *t*_R_ 14.7 min), **6** (1.1 mg, *t*_R_ 17.5 min), **7** (0.3 mg, *t*_R_ 19.0 min), **8** (1.4 mg, *t*_R_ 19.8 min), **9** (60.7 mg, *t*_R_ 21.0 min), **10** (0.7 mg, *t*_R_ 25.5 min), **11** (8.5 mg, *t*_R_ 27.0 min), **12** (4.6 mg, *t*_R_ 29.6 min), **13** (6.1 mg, *t*_R_ 33.5 min), **14** (21 mg, *t*_R_ 35.2 min), **15** (0.4 mg, *t*_R_ 37.7 min), **16** (38.4 mg, *t*_R_ 39.9 min), **17** (2.7 mg, *t*_R_ 44.9 min), **18** (49.9 mg, *t*_R_ 46.0 min), **24** (0.4 mg, *t*_R_ 47.1 min), **19** (1.2 mg, *t*_R_ 49.5 min), **20** (1.8 mg, *t*_R_ 50.3 min), **21** (5.4 mg, *t*_R_ 51.2 min), and **22** (28.3 mg, *t*_R_ 64.0 min).

### Biotransformation Reaction N°2 (With *n*-BuOH as Cosolvent)

16 compounds were purified based on 2 injections of 55 mg each: **25** (5.1 mg, *t*_R_ 13.2 min), **26** (0.5 mg, *t*_R_ 14.1 min), **27** (0.3 mg, *t*_R_ 20.5 min), **28** (0.4 mg, *t*_R_ 28.0 min), **29** (0.2 mg, *t*_R_ 29.0 min), **30** (1.8 mg, *t*_R_ 31.3 min), **31** (3.8 mg, *t*_R_ 36.9 min), **32** (0.1 mg, *t*_R_ 38.8 min), **33** (1.0 mg, *t*_R_ 46.7 min), **34** (0.7 mg, *t*_R_ 48.0 min), **35** (0.3 mg, *t*_R_ 48.8 min), **36** (0.2 mg, *t*_R_ 51.7 min), **37** (1.0 mg, *t*_R_ 52.8 min), **38** (0.4 mg, *t*_R_ 53.4 min), **39** (0.3 mg, *t*_R_ 65.8 min), and **40** (0.4 mg, *t*_R_ 66.5 min).

### Biotransformation Reaction N°3 (With i-BuOH as Cosolvent)

13 compounds purified based on 1 injection of 55 mg: **41** (2.1 mg, *t*_R_ 13.8 min), **42** (0.4 mg, *t*_R_ 29.0 min), **43** (0.7 mg, *t*_R_ 29.8 min), **44** (0.6 mg, *t*_R_ 32.8 min), **45** (1.9 mg, *t*_R_ 38.1 min), **46** (0.4 mg, *t*_R_ 48.1 min), **47** (0.1 mg, *t*_R_ 49.2 min), **48** (0.1 mg, *t*_R_ 49.7 min), **49** (0.1 mg, *t*_R_ 53.0 min), **50** (0.3 mg, *t*_R_ 53.9 min), **51** (0.2 mg, *t*_R_ 54.8 min), **52** (0.2 mg, *t*_R_ 66.5 min), and **53** (0.5 mg, *t*_R_ 67.5 min).

### Biotransformation Reaction N°4 (With MeOH as Cosolvent)

11 compounds were purified based on 2 injections of 50 mg: **54** (0.2 mg, *t*_R_ 22.2 min), **55** (0.1 mg, *t*_R_ 24.4 min), **56** (0.6 mg, *t*_R_ 24.6 min), **57** (0.6 mg, *t*_R_ 25.6 min), **58** (1.1 mg, *t*_R_ 30.2 min), **59** (6 mg, *t*_R_ 31.5 min), **60** (1.3 mg, *t*_R_ 37.9 min), **61** (0.2 mg, *t*_R_ 38.8 min), **62** (1 mg, *t*_R_ 42.6 min), **63** (2.7 mg, *t*_R_ 44.1 min), and **64** (0.4 mg, *t*_R_ 54.1 min).

### Biotransformation Reaction N°5 (With EtOH as Cosolvent)

6 compounds were purified based on 2 injections of 40 mg: **70** (0.5 mg, *t*_R_ 22.5 min), **65** (1.6 mg, *t*_R_ 35.8 min), **66** (0.5 mg, *t*_R_ 41.6 min), **67** (5.4 mg, *t*_R_ 42.5 min), **68** (0.4 mg, *t*_R_ 54.8 min), **69** (0.7 mg, *t*_R_ 70.6 min). The fractions collected from 17.5 to 19.0 min (3.6 mg) were further purified on a smaller XBridge C18 column (250 × 10 mm i.d., 5 μm; Waters) by using isocratic conditions at 30% MeOH to give compound **71** (0.7 mg, *t*_R_ 50 min). The fractions collected from 33.5 to 35.0 min (2.3 mg) were further purified on a smaller XBridge C18 column (250 × 10 mm i.d., 5 μm; Waters) by using isocratic conditions at 42% MeOH to give compound **72** (1.1 mg,, *t*_R_ 68 min) and **73** (0.2 mg, *t*_R_ 92.5 min).

### Description of the Generated Compounds

Pallidol (**1**) UV (MeCN) λ_max_ (log ε) 228 (sh) (4.29), 283 (3.58) nm. ^1^H NMR (DMSO, 600 MHz) δ 3.60 (2H, s, H-8, H-8′), 4.30 (2H, s, H-7, H-7′), 6.05 (2H, d, *J* = 2.0 Hz, H-12, H-12′), 6.38 (2H, d, *J* = 2.0 Hz, H-10, H-10′), 6.60 (4H, d, *J* = 8.5 Hz, H-3, H-3′, H-5, H-5′), 6.84 (4H, d, *J* = 8.5 Hz, H-2, H-2′, H-6, H-6′), 8.88 (2H, s), 9.01 (2H, s), 9.05 (2H, s). HR-ESI/MS analysis: *m/z* 453.1346 [M-H]^-^, (calcd for C_28_H_21_O_6_, 453.1338, ∆ = 1.7 ppm). MS/MS spectrum: CCMSLIB00009918856.

*Threo*-resveratrol acyclic dimer (**2**) UV (MeCN) λ_max_ (log ε) 228 (sh) (4.25), 285 (4.00), 306 (4.14), 323 (4.15), 340 (sh) (3.85) nm. ^1^H NMR (DMSO, 600 MHz) δ 4.66 (1H, dd, *J* = 6.4, 4.5 Hz, H-7′), 5.00 (1H, d, *J* = 6.4 Hz, H-8′), 5.41 (1H, d, *J* = 4.5 Hz, 7′OH), 5.97 (1H, t, *J* = 2.2 Hz, H-12′), 6.03 (2H, d, *J* = 2.2 Hz, H-10′, H-14′), 6.10 (1H, t, *J* = 2.1 Hz, H-12), 6.35 (2H, d, *J* = 2.1 Hz, H-10, H-14), 6.58 (2H, d, *J* = 8.6 Hz, H-3′, H-5′), 6.81 (1H, d, *J* = 16.2 Hz, H-8), 6.83 (2H, d, *J* = 8.6 Hz, H-3, H-5), 6.89 (1H, d, *J* = 16.2 Hz, H-7), 6.99 (2H, d, *J* = 8.5 Hz, H-2′, H-6′), 7.38 (2H, d, *J* = 8.5 Hz, H-2, H-6), 9.02 (2H, s, 11′OH, 13′OH), 9.18 (3H, m, 4′OH, 11OH, 13OH). HR-ESI/MS analysis: *m/z* 471.1451 [M-H]^−^, (calcd for C_28_H_23_O_7_, 471.1444, ∆ = 1.5 ppm). MS/MS spectrum: CCMSLIB00009918857.

*Erythro*-resveratrol acyclic dimer **(3)** UV (MeCN) λ_max_ (log ε) 229 (sh) (4.33), 285 (4.00), 308 (4.10), 324 (4.11) nm. ^1^H NMR (DMSO, 600 MHz) δ 4.63 (1H, t, *J* = 6.3, 4.8 Hz, H-7′), 5.00 (1H, d, *J* = 6.3 Hz, H-8′), 5.30 (1H, d, *J* = 4.8 Hz, 7′OH), 6.05 (1H, t, *J* = 2.2 Hz, H-12′), 6.09 (1H, t, *J* = 2.2 Hz, H-12), 6.18 (2H, d, *J* = 2.2 Hz, H-10′, H-14′), 6.34 (2H, d, *J* = 2.2 Hz, H-10, H-14), 6.65 (2H, d, *J* = 8.5 Hz, H-3′, H-5′), 6.71 (2H, d, *J* = 8.7 Hz, H-3, H-5), 6.79 (1H, d, *J* = 16.2 Hz, H-8), 6.87 (1H, d, *J* = 16.2 Hz, H-7), 7.13 (2H, d, *J* = 8.5 Hz, H-2′, H- 6′), 7.34 (2H, d, *J* = 8.7 Hz, H-2, H-6), 9.07 (2H, s, 11′OH, 13′OH), 9.17 (2H, s, 11OH, 13OH), 9.20 (1H, s, 4′OH). ^1^H NMR (Acetone, 600 MHz) δ 4.28 (1H, d, J = 4.7 Hz, 7′OH), 4.86 (1H, d, J = 5.8 Hz, H-7′), 5.13 (1H, d, J = 5.8 Hz, H-8′), 6.23 (1H, t, J = 2.2 Hz, H-12′), 6.25 (1H, t, J = 2.2 Hz, H-12), 6.34 (2H, d, J = 2.2 Hz, H-10′, H-14′), 6.50 (2H, d, J = 2.2 Hz, H-10, H-14), 6.74 (2H, d, J = 8.6 Hz, H-3′, H-5′), 6.80 (1H, d, J = 8.8 Hz, H-3, H-5), 6.84 (1H, d, J = 16.3 Hz, H-8), 6.95 (1H, d, J = 16.3 Hz, H-7), 7.23 (2H, d, J = 8.6 Hz, H-2′, H-6′), 7.35 (2H, d, J = 8.8 Hz, H-2, H-6); ^13^C NMR (Acetone, 151 MHz) δ 77.3 (C-7′), 84.9 (C-8′), 102.7 (C-12), 102.8 (C-12′), 105.7 (C-10, C-14), 107.1 (C-10′, C-14′), 115.2 (C-3′, C-5′), 117.0 (C-3, C-5), 127.5 (C-8), 128.3 (C-2, C-6), 128.8 (C-7), 129.5 (C-2′, C-6′), 131.0 (C-1), 133.7 (C-1′), 140.7 (C-9), 142.1 (C-9′), 157.5 (C-4′), 158.9 (C-4), 159.0 (C-11′, C-13′), 159.6 (C-11, C-13). HR-ESI/MS analysis: *m/z* 471.1451 [M-H]^-^, (calcd for C_28_H_23_O_7_, 471.1444, ∆ = 1.5 ppm). MS/MS spectrum: CCMSLIB00009918858.

7-*O*-Isopropylleachianol G (**4**) UV (MeCN) λ_max_ (log ε) 228 (sh) (4.36), 281 (3.60) nm. ^1^H NMR (DMSO, 600 MHz) δ 0.60 (3H, d, *J* = 6.0 Hz, CH_3_-7c), 0.83 (3H, d, *J* = 6.0 Hz, CH_3_-7b), 3.02 (1H, dd, *J* = 9.2, 2.1 Hz, H-8), 3.06 (1H, hept, *J* = 6.0 Hz, H-7a), 3.35 (1H, t, *J* = 2.1 Hz, H-8′), 3.89 (1H, d, *J* = 9.2 Hz, H-7), 4.06 (1H, d, *J* = 2.1 Hz, H-7′), 5.17 (1H, d, *J* = 2.1 Hz, H-14), 5.93 (2H, d, *J* = 2.2 Hz, H-10′, H-14′), 5.98 (1H, t, *J* = 2.2 Hz, H-12′), 6.07 (1H, d, *J* = 2.1 Hz, H-12), 6.64 (2H, d, *J* = 8.5 Hz, H-3′, H-5′), 6.67 (2H, d, *J* = 8.4 Hz, H-3, H-5), 6.78 (2H, d, *J* = 8.5 Hz, H-2′, H-6′), 6.88 (2H, d, *J* = 8.4 Hz, H-2, H-6), 8.74 (1H, s, 13OH), 8.77 (1H, s, 11OH), 8.99 (2H, s, 11′OH, 13′OH), 9.04 (1H, s, 4′OH), 9.29 (1H, s, 4OH); ^13^C NMR (DMSO, 151 MHz) δ 20.2 (CH_3_-7c), 23.4 (CH_3_-7b), 53.9 (C-7′), 57.6 (C-8′), 60.3 (C-8), 67.1 (C-7a), 81.1 (C-7), 100.0 (C-12′), 101.4 (C-12), 104.3 (C-14), 104.6 (C-10′, C-14′), 114.6 (C-3, C-3′, C-5, C-5′), 121.3 (C-10), 128.1 (C-2′, C-6′), 128.9 (C-2, C-6), 131.6 (C-1), 136.8 (C-1′), 145.6 (C-9), 150.5 (C-9′), 154.0 (C-11), 155.1 (C-4′), 156.6 (C-4), 157.1 (C-13), 158.2 (C-11′, C-13′). HR-ESI/MS analysis: *m/z* 513.1910 [M-H]^−^, (calcd for C_31_H_29_O_7_, 513.1913, ∆ = 0.6 ppm). MS/MS spectrum: CCMSLIB00009918859.

Di-*O*-methyl-restrytisol A (**5**) UV (MeCN) λ_max_ (log ε) 228 (sh) (4.15), 281 (3.40) nm. ^1^H NMR (DMSO, 600 MHz) δ 3.53 (6H, s, CH_3_O-11′, CH_3_O-13′), 3.90 (1H, t, *J* = 6.2, 4.3 Hz, H-8′), 3.96 (1H, dd, *J* = 10.3, 6.2 Hz, H-8), 5.39 (1H, d, *J* = 10.3 Hz, H-7), 5.76 (1H, d, *J* = 4.3 Hz, H-7′), 5.87 (1H, t, *J* = 2.1 Hz, H-12), 5.89 (2H, d, *J* = 2.2 Hz, H-10, H-14), 6.05 (1H, t, *J* = 2.3 Hz, H-12′), 6.09 (2H, d, *J* = 2.3 Hz, H-10′, H-14′), 6.54 (2H, d, *J* = 8.6 Hz, H-3′, H-5′), 6.70 (2H, d, *J* = 8.5 Hz, H-3, H-5), 7.04 (2H, d, *J* = 8.6 Hz, H-2′, H-6′), 7.26 (2H, d, *J* = 8.6 Hz, H-2, H-6), 8.88 (2H, s, 11OH, 13OH), 9.08 (1H, s, 4′OH), 9.33 (1H, s, 4OH); ^13^C NMR (DMSO, 151 MHz) δ 54.7 (CH_3_O-11′, CH_3_O-13′), 56.6 (C-8′), 58.6 (C-8), 81.2 (C-7), 83.3 (C-7′), 97.6 (C-12′), 100.4 (C-12), 106.7 (C-12, C-14), 108.5 (C-10′, C-14′), 114.3 (C-3′, C-5′), 115.0 (C-3, C-5), 126.8 (C-2′, C-6′), 127.8 (C-2, C-6), 130.1 (C-1′), 133.0 (C-1), 155.2 (C-4′), 156.6 (C-4), 157.5 (C-11, C-13), 159.1 (C-11′, C-13′). HR-ESI/MS analysis: *m/z* 499.1760 [M-H]^-^, (calcd for C_30_H_27_O_7_, 499.1757, ∆ = 0.6 ppm). MS/MS spectrum: CCMSLIB00009918860.

11′,13′-Di-*O*-methyltricuspidatol A (**6**) UV (MeCN) λ_max_ (log ε) 228 (sh) (4.35), 281 (3.74), 323 (3.44) nm. ^1^H NMR (DMSO, 600 MHz) δ 3.44 (2H, m, H-8, H-8′), 3.63 (6H, s, CH_3_O-11′, CH_3_O-13′), 5.06 (1H, m, H-7), 5.12 (1H, m, H-7′), 5.97 (1H, t, *J* = 2.2 Hz, H-12), 6.01 (2H, d, *J* = 2.1 Hz, H-10, H-14), 6.24 (2H, d, *J* = 2.3 Hz, H-10′, H-14′), 6.27 (1H, t, *J* = 2.2 Hz, H-12′), 6.69 (1H, d, *J* = 8.5 Hz, H-3′, H-5′), 6.70 (2H, d, *J* = 8.5 Hz, H-3, H-5), 7.13 (2H, d, *J* = 8.5 Hz, H-2′, H-6′), 7.16 (2H, d, *J* = 8.5 Hz, H-2, H-6), 9.01 (2H, s, 11OH, 13OH), 9.31 (1H, s, 4′OH), 9.32 (1H, s, 4OH); ^13^C NMR (DMSO, 151 MHz) δ 54.7 (CH_3_O-11′, CH_3_O-13′), 60.5 (C-8), 61.1 (C-8′), 86.0 (C-7′), 86.5 (C-7), 97.7 (C-12′), 100.9 (C-12), 105.8 (C-10, C-14), 106.0 (C-10′, C-14′), 114.6 (C-3, C-5, C-3′, C-5′), 127.8 (C-2, C-6, C-2′, C-6′), 131.5 (C-1, C-1′), 156.6 (C-4, C-4′), 157.8 (C-11, C-13), 160.0 (C-11′, C-13′). HR-ESI/MS analysis: *m/z* 499.1760 [M-H]^-^, (calcd for C_30_H_27_O_7_, 499.1757, ∆ = 0.6 ppm). MS/MS spectrum: CCMSLIB00009918861.

11,13-Di-*O*-methylrestrytisol B (**7**) UV (MeCN) λ_max_ (log ε) 228 (sh) (4.21), 281 (3.47) nm. ^1^H NMR (DMSO, 600 MHz) δ 3.39 (1H, t, *J* = 10.2, 8.9 Hz, H-8), 3.64 (6H, s, CH_3_O-13, CH_3_O-11), 3.95 (1H, t, *J* = 10.2, 9.7 Hz, H-8′), 4.93 (1H, d, *J* = 9.7 Hz, H-7), 5.39 (1H, d, *J* = 8.9 Hz, H-7′), 5.78 (1H, t, *J* = 2.2 Hz, H-12′), 5.85 (2H, d, *J* = 2.2 Hz, H-10′, H-14′), 6.27 (1H, t, *J* = 2.2 Hz, H-12), 6.34 (2H, d, *J* = 2.2 Hz, H-10, H-14), 6.51 (2H, d, *J* = 8.5 Hz, H-3′, H-5′), 6.73 (2H, d, *J* = 8.7 Hz, H-3, H-5), 6.95 (2H, d, *J* = 8.5 Hz, H-2′, H-6′), 7.21 (2H, d, *J* = 8.7 Hz, H-2, H-6), 8.78 (2H, s, 11′OH, 13′OH), 9.08 (1H, s, 4′OH), 9.38 (1H, s, 4OH); ^13^C NMR (DMSO, 151 MHz) δ 54.7 (CH_3_O-11, CH_3_O-13), 57.5 (C-8), 57.6 (C-8′), 82.4 (C-7′), 85.4 (C-7), 97.6 (C-12), 100.2 (C-12′), 106.2 (C-10, C-14), 107.1 (C-10′, C-14′), 113.9 (C-3′, C-5′), 114.8 (C-3, C-5), 127.6 (C-2′, C-6′), 128.0 (C-2, C-6), 129.7 (C-1), 130.6 (C-1′), 141.6 (C-9), 141.8 (C-9′), 155.5 (C-4′), 156.7 (C-4), 157.1 (C-11′, C-13′), 160.0 (C-11, C-13). HR-ESI/MS analysis: *m/z* 499.1758 [M-H]^−^, (calcd for C_30_H_27_O_7_, 499.1757, ∆ = 0.2 ppm). MS/MS spectrum: CCMSLIB00009918862.

11, 13-Di-*O*-methylisopallidol (**8**) UV (MeCN) λ_max_ (log ε) 229 (sh) (4.29), 282 (3.64) nm. ^1^H NMR (DMSO, 600 MHz) δ 3.57 (3H, s, CH_3_O-13), 3.64 (1H, d, *J* = 6.2 Hz, H-8′), 3.69 (1H, d, *J* = 6.2 Hz, H-8), 3.80 (3H, s, CH_3_O-11), 4.33 (1H, s, H-7′), 4.41 (1H, s, H-7), 6.05 (1H, d, *J* = 2.0 Hz, H-12′), 6.31 (1H, d, *J* = 2.1 Hz, H-12), 6.40 (1H, d, *J* = 2.0 Hz, H-10′), 6.60 (2H, d, *J* = 8.5 Hz, H-3, H-5), 6.61 (2H, d, *J* = 8.5 Hz, H-3′, H-5′), 6.75 (1H, d, *J* = 2.1 Hz, H-10), 6.82 (2H, d, *J* = 8.5 Hz, H-2′, H-6′), 6.90 (2H, d, *J* = 8.5 Hz, H-2, H-6), 8.92 (1H, s), 9.03 (1H, s), 9.06 (1H, s), 9.09 (1H, s). HR-ESI/MS analysis: *m/z* 481.1657 [M-H]^−^, (calcd for C_30_H_25_O_6_, 481.1651, ∆ = 1.2 ppm). MS/MS spectrum: CCMSLIB00009918863.

*Trans*-δ-viniferin (**9**) UV (MeCN) λ_max_ (log ε) 227 (sh) (4.46), 285 (4.08), 312 (4.32), 333 (4.127), 350 (sh) (3.99) nm. ^1^H NMR (DMSO, 600 MHz) δ 4.44 (1H, d, *J* = 7.8 Hz, H-8′), 5.39 (1H, d, *J* = 7.8 Hz, H-7′), 6.03 (2H, d, *J* = 2.1 Hz, H-10′, H-14′), 6.10 (1H, t, *J* = 2.1 Hz, H-12′), 6.11 (1H, t, *J* = 2.2 Hz, H-12), 6.37 (2H, d, *J* = 2.2 Hz, H-10, H-14), 6.76 (2H, d, *J* = 8.6 Hz, H-3′, H-5′), 6.83 (1H, d, *J* = 16.3 Hz, H-8), 6.89 (1H, d, *J* = 8.3 Hz, H-5), 6.98 (1H, d, *J* = 16.3 Hz, H-7), 7.18 (2H, d, *J* = 8.6 Hz, H-2′, H-6′), 7.23 (1H, d, *J* = 1.9 Hz, H-2), 7.42 (1H, dd, *J* = 8.3, 1.9 Hz, H-6), 9.17 (2H, s), 9.21 (2H, s), 9.53 (1H, s, 4′OH). HR-ESI/MS analysis: *m/z* 453.1348 [M-H]^-^, (calcd for C_28_H_21_O_6_, 453.1338, ∆ = 2.2 ppm). MS/MS spectrum: CCMSLIB00009918864.

7-*O*-Isopropyl-11′,13′-di-*O*-methylleachianol F (**10**) UV (MeCN) λ_max_ (log ε) 228 (sh) (4.30), 284 (3.76), 323 (3.70) nm. ^1^H NMR (DMSO, 600 MHz) δ 0.91 (6H, d, *J* = 6.1 Hz, CH_3_-7b, CH_3_-7c), 2.71 (1H, t, *J* = 3.4 Hz, H-8′), 3.05 (1H, dd, *J* = 9.1, 3.4 Hz, H-8), 3.21 (1H, hept, *J* = 6.1 Hz, H-7a), 3.57 (6H, s, CH_3_O-13′, CH_3_O-11′), 4.00 (1H, d, *J* = 9.1 Hz, H-7), 4.06 (1H, d, *J* = 3.1 Hz, H-7′), 5.73 (2H, d, *J* = 2.2 Hz, H-10′, H-14′), 6.16 (1H, d, *J* = 2.2 Hz, H-12), 6.20 (1H, t, *J* = 2.2 Hz, H-12′), 6.47 (1H, d, *J* = 2.2 Hz, H-14), 6.57 (2H, d, *J* = 8.5 Hz, H-3, H-5), 6.64 (1H, d, *J* = 8.5 Hz, H-3′, H-5′), 6.64 (2H, d, *J* = 8.5 Hz, H-2, H-6), 6.69 (2H, d, *J* = 8.5 Hz, H-2′, H-6′), 8.77 (1H, s, 11OH), 8.95 (1H, s, 13OH), 9.10 (1H, s, 4′OH), 9.25 (1H, s, 4OH); ^13^C NMR (DMSO, 151 MHz) δ 53.6 (C-7′), 54.7 (CH_3_O-11′, CH_3_O-13′), 57.5 (C-8′), 59.8 (C-8), 67.7 (C-7a), 81.2 (C-7), 96.9 (C-12′), 101.2 (C-12), 104.2 (C-10′, C-14′), 144.4 (C-3, C-5, C-3′, C-5′), 120.5 (C-10), 128.0 (C-2′, C-6′), 128.2 (C-2, C-6), 131.7 (C-1), 136.0 (C-1′), 147.0 (C-9), 149.2 (C-9′), 153.5 (C-11), 155.1 (C-4′), 156.2 (C-4), 157.3 (C-13), 159.8 (C-11′, C-13′). HR-ESI/MS analysis: *m/z* 541.2225 [M-H]^−^, (calcd for C_33_H_33_O_7_, 541.2226, ∆ = 0.2 ppm). MS/MS spectrum: CCMSLIB00009918865.

*Erythro*-7′-*O*-isopropylresveratrol acyclic dimer (**11**) UV (MeCN) λ_max_ (log ε) 229 (sh) (4.32), 284 (3.73), 325 (3.67) nm. ^1^H NMR (DMSO, 600 MHz) δ 0.87 (3H, d, *J* = 6.0 Hz, H-7′c), 0.91 (3H, d, *J* = 6.2 Hz, H-7′b), 3.33 (1H, overlapped, H-7′a), 4.43 (1H, d, *J* = 6.6 Hz, H-7′), 5.02 (1H, d, *J* = 6.6 Hz, H-8′), 6.06 (1H, t, *J* = 2.2 Hz, H-12′), 6.09 (1H, t, *J* = 2.1 Hz, H-12), 6.21 (2H, d, *J* = 2.2 Hz, H-10′, H-14′), 6.34 (2H, d, *J* = 2.1 Hz, H-10, H-14), 6.67 (1H, d, *J* = 8.6 Hz, H-3′, H5′), 6.70 (2H, d, *J* = 8.8 Hz, H-3, H-5), 6.78 (1H, d, *J* = 16.6 Hz, H-8), 6.86 (1H, d, *J* = 16.6 Hz, H-7), 7.16 (2H, d, *J* = 8.6 Hz, H-2′, H-6′), 7.33 (2H, d, *J* = 8.8 Hz, H-2, H-6), 9.08 (2H, s, 11′OH, 13′OH), 9.17 (2H, s, 11OH, 13OH), 9.26 (1H, s, 4′OH); ^13^C NMR (DMSO, 151 MHz) δ 21.2 (C-7′c), 23.0 (C-7’b), 69.4 (C-7’a), 81.4 (C-7′), 82.1 (C-8′), 101.8 (C-12, C-12′), 104.2 (C-10, C-14), 105.7 (C-10′, C-14′), 114.3 (C-3′, C-5′), 115.9 (C-3, C-5), 126.6 (C-8), 127.4 (C-2, C-6, C-7), 128.9 (C-2′, C-6′), 129.4 (C-1), 129.8 (C-1′), 138.8 (C-9), 141.1 (C-9′), 156.6 (C-4′), 157.0 (C-4), 157.7 (C-11′, C-13′), 158.4 (C-11, C-13). HR-ESI/MS analysis: *m/z* 513.1913 [M-H]^-^, (calcd for C_31_H_29_O_7_, 513.1913, ∆ = 0 ppm). MS/MS spectrum: CCMSLIB00009918866.

*Threo*-7′-*O*-isopropylresveratrol acyclic dimer (**12**) UV (MeCN) λ_max_ (log ε) 228 (sh) (4.29), 285 (4.01), 305 (4.14), 324 (4.15), 343 (sh) (3.84) nm. ^1^H NMR (DMSO, 600 MHz) δ 0.96 (3H, d, *J* = 6.2 Hz, H-7′c), 1.03 (3H, d, *J* = 6.2 Hz, H-7'b), 3.47 (1H, hept, *J* = 6.2 Hz, H-7′a), 4.51 (1H, d, *J* = 6.3 Hz, H-7′), 5.06 (1H, d, *J* = 6.3 Hz, H-8′), 5.97 (1H, t, *J* = 2.2 Hz, H-12′), 6.08 (2H, d, *J* = 2.2 Hz, H-10′, H-14′), 6.10 (1H, t, *J* = 2.1 Hz, H-12), 6.35 (2H, d, *J* = 2.1 Hz, H-10, H-14), 6.60 (2H, d, *J* = 8.5 Hz, H-3′, H-5′), 6.80 (2H, d, *J* = 8.9 Hz, H-3, H-5), 6.81 (1H, d, *J* = 16.3 Hz, H-8), 6.89 (1H, d, *J* = 16.3 Hz, H-7), 7.03 (2H, d, *J* = 8.5 Hz, H-2′, H-6′), 7.38 (2H, d, *J* = 8.9 Hz, H-2, H-6), 9.01 (2H, s, 11′OH, 13′OH), 9.17 (2H, s, 11OH, 13OH), 9.22 (1H, s, 4′OH). ^13^C NMR (DMSO, 151 MHz) δ 21.3 (C-7'c), 23.0 (C-7'b), 69.5 (C-7'a), 81.9 (C-7′), 82.9 (C-8′), 101.6 (C-12′), 101.7 (C-12), 104.2 (C-10, C-14), 105.5 (C-10′, C-14′), 114.3 (C-3′, C-5′), 115.7 (C-3, C-5), 126.5 (C-8), 127.3 (C-2, C-6, C-7), 128.6 (C-2′, C-6′), 129.3 (C-1), 129.8 (C-1′), 138.9 (C-9), 140.3 (C-9′), 156.4 (C-4′), 157.7 (C-11′, C-13′), 157.8 (C-4), 158.4 (C-11, C-13). HR-ESI/MS analysis: *m/z* 513.1918 [M-H]^-^, (calcd for C_31_H_29_O_7_, 513.1913, ∆ = 1.0 ppm). MS/MS spectrum: CCMSLIB00009918867.

*Threo*-resveptero acyclic dimer (**13**) UV (MeCN) λ_max_ (log ε) 228 (sh) (4.37), 285 (4.01), 305 (4.12), 326 (4.10), 343 (sh) (3.80) nm. ^1^H NMR (DMSO, 600 MHz) δ 3.75 (6H, s, CH_3_O-11, CH_3_O-13), 4.67 (1H, dd, *J* = 6.4, 2.9 Hz, H-7′), 5.02 (1H, d, *J* = 6.4 Hz, H-8′), 5.41 (1H, d, *J* = 2.9 Hz, 7′OH), 5.98 (1H, t, *J* = 2.2 Hz, H-12′), 6.04 (2H, d, *J* = 2.2 Hz, H-10′, H-14′), 6.36 (1H, t, *J* = 2.3 Hz, H-12), 6.59 (2H, d, *J* = 8.4 Hz, H-3′, H-5′), 6.69 (2H, d, *J* = 2.3 Hz, H-10, H-14), 6.85 (2H, d, *J* = 9.0 Hz, H-3, H-5), 6.95 (1H, d, *J* = 16.3 Hz, H-8), 7.00 (2H, d, *J* = 8.4 Hz, H-2′, H-6′), 7.13 (1H, d, *J* = 16.3 Hz, H-7), 7.41 (2H, d, *J* = 9.0 Hz, H-2, H-6), 9.02 (2H, s, 11′OH, 13′OH), 9.18 (1H, s, 4′OH); ^13^C NMR (DMSO, 151 MHz) δ 55.2 (CH_3_O-11, CH_3_O-13), 76.1 (C-7′), 84.0 (C-8′), 99.5 (C-12), 101.7 (C-12′), 104.1 (C-10, C-14), 105.6 (C-10′, C-14′), 114.3 (C-3′, C-5′), 116.0 (C-3, C-5), 126.1 (C-8), 127.5 (C-2, C-6), 128.3 (C-2′, C-6′), 128.5 (C-7), 129.3 (C-1), 131.9 (C-1′), 139.4 (C-9), 140.6 (C-9′), 156.2 (C-4′), 157.7 (C-11′, C-13′), 157.9 (C-4), 160.6 (C-11, C-13). HR-ESI/MS analysis: *m/z* 499.1763 [M-H]^−^, (calcd for C_30_H_27_O_7_, 499.1757, ∆ = 1.2 ppm). MS/MS spectrum: CCMSLIB00009918868.

7-*O*-Isopropyl-11′,13′-di-*O*-methylleachianol G (**14**) UV (MeCN) λ_max_ (log ε) 228 (sh) (4.42), 283 (3.75), 323 (3.44) nm. ^1^H NMR (DMSO, 600 MHz) δ 0.65 (3H, d, *J* = 6.1 Hz, CH_3_-7c), 0.85 (3H, d, *J* = 6.1 Hz, CH_3_-7b), 3.08 (1H, dd, *J* = 8.6, 2.6 Hz, H-8), 3.11 (1H, hept, *J* = 6.1 Hz, H-7a), 3.43 (1H, t, *J* = 2.6 Hz, H-8′), 3.66 (6H, s, CH_3_O-11′, CH_3_O-13′), 4.02 (1H, d, *J* = 8.6 Hz, H-7), 4.09 (6H, d, *J* = 2.6 Hz, H-7′), 5.31 (1H, d, *J* = 2.1 Hz, H-14), 6.08 (1H, d, *J* = 2.1 Hz, H-12), 6.12 (3H, d, *J* = 2.3 Hz, H-10′, H-14′), 6.30 (1H, t, *J* = 2.3 Hz, H-12′), 6.63 (2H, d, *J* = 8.5 Hz, H-3′, H-5′), 6.66 (2H, d, *J* = 8.4 Hz, H-3, H-5), 6.80 (2H, d, *J* = 8.5 Hz, H-2′, H-6′), 6.91 (2H, d, *J* = 8.4 Hz, H-2, H-6), 8.78 (1H, s, 11OH or 13OH), 8.79 (1H, s, 11OH or 13OH), 9.04 (1H, s, 4′OH), 9.28 (1H, s, 4OH); ^13^C NMR (DMSO, 151 MHz) δ 20.3 (CH_3_-7c), 23.4 (CH_3_-7b), 54.0 (C-7′), 54.9 (CH_3_O-11′, CH_3_O-13′), 57.5 (C-8′), 60.5 (C-8), 67.1 (C-7a), 80.6 (C-7), 96.9 (C-12′), 101.4 (C-12), 104.0 (C-14), 104.9 (C-10′, C-14′), 114.6 (C-3′, C-5′), 114.6 (C-3, C-5), 121.3 (C-10), 128.2 (C-2′, C-6′), 128.8 (C-2, C-6), 131.4 (C-1), 136.5 (C-1′), 145.4 (C-9), 150.4 (C-9′), 154.0 (C-11), 155.1 (C-4′), 156.6 (C-4), 157.3 (C-13), 160.3 (C-11′, C-13′). HR-ESI/MS analysis: *m/z* 541.2222 [M-H]^−^, (calcd for C_33_H_33_O_7_, 541.2226, ∆ = 0.7 ppm). MS/MS spectrum: CCMSLIB00009918869.

11,11′,13,13′-Tetra-*O*-methylrestrytisol B (**15**) UV (MeCN) λ_max_ (log ε) 228 (sh) (4.43), 283 (3.88), 323 (3.84) nm. ^1^H NMR (DMSO, 600 MHz) δ 3.53 (6H, s, CH_3_O-11′, CH_3_O-13′), 3.57 (1H, t, *J* = 10.6, 9.8 Hz, H-8), 3.64 (6H, s, CH_3_O-11, CH_3_O-13), 4.12 (1H, t, *J* = 10.6, 9.1 Hz, H-8′), 4.99 (1H, d, *J* = 9.8 Hz, H-7), 5.43 (1H, d, *J* = 9.1 Hz, H-7′), 6.04 (1H, t, *J* = 2.3 Hz, H-12′), 6.10 (2H, d, *J* = 2.3 Hz, H-10′, H-14′), 6.25 (1H, t, *J* = 2.3 Hz, H-12), 6.42 (2H, d, *J* = 2.3 Hz, H-10, H-14), 6.52 (2H, d, *J* = 8.5 Hz, H-3′, H-5′), 6.74 (2H, d, *J* = 8.4 Hz, H-3, H-5), 6.96 (2H, d, *J* = 8.5 Hz, H-2′, H-6′), 7.25 (2H, d, *J* = 8.4 Hz, H-2, H-6), 9.10 (1H, s, 4′OH), 9.37 (1H, s, 4OH); ^13^C NMR (DMSO, 151 MHz) δ 54.6 (CH_3_O-11′, CH_3_O-13′), 54.7 (CH_3_O-11, CH_3_O-13), 56.3 (C-8), 57.7 (C-8′), 82.3 (C-7′), 85.5 (C-7), 97.7 (C-12, C-12′), 106.2 (C-10, C-14), 107.0 (C-10′, C-14′), 113.9 (C-3′, C-5′), 114.8 (C-3, C-5), 127.7 (C-2′, C-6′), 128.0 (C-2, C-6), 129.4 (C-1), 130.7 (C-1′), 141.0 (C-9′), 141.2 (C-9), 155.5 (C-4′), 156.7 (C-4), 159.1 (C-11′), 159.9 (C-11). HR-ESI/MS analysis: *m/z* 527.2075 [M-H]^−^, (calcd for C_32_H_31_O_7_, 527.2070, ∆ = 0.9 ppm). MS/MS spectrum: CCMSLIB00009918870.

11′,13′-Di-*O*-methyl-*trans*-δ-viniferin (**16**) UV (MeCN) λ_max_ (log ε) 227 (sh) (4.45), 285 (4.06), 312 (4.30), 333 (4.25), 350 (sh) (3.97) nm. ^1^H NMR (DMSO, 600 MHz) δ 3.70 (6H, s, CH_3_O-11′, CH_3_O-13′), 4.58 (1H, d, *J* = 8.1 Hz, H-8′), 5.56 (1H, d, *J* = 8.1 Hz, H-7′), 6.11 (1H, t, *J* = 2.2 Hz, H-12), 6.36 (4H, 2d, *J* = 2.3 Hz, H-10, H-10′, H-14, H-14′), 6.43 (1H, t, *J* = 2.3 Hz, H-12′), 6.76 (2H, d, *J* = 8.7 Hz, H-3′, H-5′), 6.82 (1H, d, *J* = 16.3 Hz, H-8), 6.90 (1H, d, *J* = 8.3 Hz, H-5), 6.97 (1H, d, *J* = 16.3 Hz, H-7), 7.19 (2H, d, *J* = 8.7 Hz, H-2′, H-6′), 7.21 (1H, d, *J* = 1.9 Hz, H-2), 7.43 (1H, dd, *J* = 8.3, 1.9 Hz, H-6), 9.17 (2H, s, 11OH, 13OH), 9.53 (1H, s, 4′OH). HR-ESI/MS analysis: *m/z* 481.1652 [M-H]^-^, (calcd for C_30_H_25_O_6_, 481.1651, ∆ = 0.2 ppm). MS/MS spectrum: CCMSLIB00009918871.

*Threo*-7′-*O*-isopropylisoresveptero acyclic dimer (**17**) UV (MeCN) λ_max_ (log ε) 228 (sh) (4.37), 285 (4.05), 305 (4.18), 323 (4.19), 343 (sh) (3.89) nm. ^1^H NMR (DMSO, 600 MHz) δ 0.97 (3H, d, *J* = 6.2 Hz, CH_3_-7′c), 1.03 (3H, d, *J* = 6.1 Hz, CH_3_-7′b), 3.49 (2H, hept, *J* = 6.1 Hz, H-7′a), 3.61 (6H, s, CH_3_O-11′, CH_3_O-13′), 4.60 (1H, d, *J* = 6.2 Hz, H-7′), 5.24 (1H, d, *J* = 6.2 Hz, H-8′), 6.10 (1H, t, *J* = 2.2 Hz, H-12), 6.25 (1H, t, *J* = 2.3 Hz, H-12′), 6.34 (2H, d, *J* = 2.3 Hz, H-10′, H-14′), 6.35 (2H, d, *J* = 2.2 Hz, H-10, H-14), 6.60 (2H, d, *J* = 8.4 Hz, H-3′, H-5′), 6.82 (1H, d, *J* = 16.3 Hz, H-8), 6.84 (2H, d, *J* = 8.7 Hz, H-3, H-5), 6.89 (1H, d, *J* = 16.3 Hz, H-7), 7.04 (2H, d, *J* = 8.4 Hz, H-2′, H-6′), 7.37 (2H, d, *J* = 8.7 Hz, H-2, H-6), 9.17 (2H, s, 11OH, 13OH), 9.23 (1H, s, 4′OH); ^13^C NMR (DMSO, 151 MHz) δ 21.4 (CH_3_-7′b), 23.1 (CH_3_-7'c), 54.9 (CH_3_O-11′, CH_3_O-13′), 69.5 (C-7′a), 81.8 (C-7′), 82.9 (C-8′), 99.0 (C-12′), 101.8 (C-12), 104.3 (C-10, C-14), 105.6 (C-10′, C-14′), 114.4 (C-3′, C5′), 115.9 (C-3, C-5), 126.6 (C-8), 127.3 (C-7), 127.5 (C-2, C-6), 128.8 (C-2′, C-6′), 129.5 (C-1), 129.6 (C-1′), 138.9 (C-9), 140.7 (C-9′), 156.5 (C-4′), 158.4 (C-11, C-13), 159.7 (C-11′, C-13′). HR-ESI/MS analysis: *m/z* 541.2231 [M-H]^−^, (calcd for C_33_H_33_O_7_, 541.2226, ∆ = 0.9 ppm). MS/MS spectrum: CCMSLIB00009918872.

11,13-Di-*O*-methyl-*trans*-δ-viniferin (**18**) UV (MeCN) λ_max_ (log ε) 227 (sh) (4.48), 285 (4.10), 312 (4.34), 333 (4.30), 350 (sh) (4.00) nm. ^1^H NMR (DMSO, 600 MHz) δ 3.75 (6H, s, CH_3_O-11, CH_3_O-13), 4.46 (1H, d, *J* = 8.3 Hz, H-8′), 5.41 (1H, d, *J* = 8.3 Hz, H-7′), 6.05 (2H, d, *J* = 2.1 Hz, H-10′, H-14′), 6.11 (1H, t, *J* = 2.2 Hz, H-12′), 6.35 (1H, t, *J* = 2.2 Hz, H-12), 6.73 (2H, d, *J* = 2.2 Hz, H-10, H-14), 6.76 (2H, d, *J* = 8.7 Hz, H-3′, H-5′), 6.90 (1H, d, *J* = 8.3 Hz, H-5), 6.96 (1H, d, *J* = 16.4 Hz, H-8), 7.20 (2H, d, *J* = 8.7 Hz, H-2′, H-6′), 7.22 (1H, d, *J* = 16.4 Hz, H-7), 7.24 (1H, d, *J* = 1.8 Hz, H-2), 7.44 (1H, dd, *J* = 8.3, 1.8 Hz, H-6), 9.22 (2H, s, 11′OH, 13′OH), 9.53 (1H, s, 4′OH). HR-ESI/MS analysis: *m/z* 481.1654 [M-H]^−^, (calcd for C_30_H_25_O_6_, 481.1651, ∆ = 0.6 ppm). MS/MS spectrum: CCMSLIB00009918873.

7-*O*-Isopropyl-11,11′,13,13′-tetra-*O*-methylleachianol G (**19**) UV (MeCN) λ_max_ (log ε) 228 (sh) (4.48), 283 (3.91), 324 (3.89) nm. ^1^H NMR (DMSO, 600 MHz) δ 0.67 (3H, d, *J* = 6.1 Hz, CH_3_-7c), 0.88 (3H, d, *J* = 6.1 Hz, CH_3_-7b), 3.13 (1H, dd, *J* = 8.8, 2.4 Hz, H-8), 3.16 (1H, overlapped, H-7a), 3.46 (1H, t, *J* = 2.4 Hz, H-8′), 3.48 (3H, s, CH_3_O-13), 3.56 (3H, s, CH_3_O-11), 3.66 (6H, s, CH_3_O-13′, CH_3_O-11′), 4.06 (1H, d, *J* = 8.8 Hz, H-7), 4.14 (1H, d, *J* = 2.4 Hz, H-7′), 5.41 (1H, d, *J* = 2.2 Hz, H-14), 6.10 (2H, d, *J* = 2.3 Hz, H-10′, H-14′), 6.31 (1H, t, *J* = 2.3 Hz, H-12′), 6.34 (1H, d, *J* = 2.2 Hz, H-12), 6.65 (2H, d, *J* = 8.5 Hz, H-3′, H-5′), 6.69 (2H, d, *J* = 8.4 Hz, H-3, H-5), 6.78 (2H, d, *J* = 8.5 Hz, H-2′, H-6′), 6.92 (2H, d, *J* = 8.4 Hz, H-2, H-6), 9.09 (1H, s, 4′OH), 9.32 (1H, s, 4OH); ^13^C NMR (DMSO, 151 MHz) δ 20.0 (CH_3_-7c), 23.2 (CH_3_-7b), 48.3 (C-7a), 54.1 (C-7′), 54.5 (CH_3_O-13), 54.7 (CH_3_O-11′, CH_3_O-13′), 54.8 (CH_3_O-11), 57.3 (C-8′), 60.6 (C-8), 80.5 (C-7), 96.7 (C-12′), 97.4 (C-12), 101.8 (C-14), 104.6 (C-10′, C-14′), 114.4 (C-3, C-5), 114.6 (C-3′, C-5′), 124.1 (C-10), 127.8 (C-2′, C-6′), 128.7 (C-2, C-6), 131.0 (C-1), 136.0 (C-1′), 145.0 (C-9), 149.9 (C-9′), 154.8 (C-4′), 156.0 (C-11), 156.6 (C-4), 159.6 (C-13), 160.2 (C-11′, C-13′). HR-ESI/MS analysis: *m/z* 569.2543 [M-H]^−^, (calcd for C_35_H_37_O_7_, 569.2539, ∆ = 0.7 ppm). MS/MS spectrum: CCMSLIB00009918874.

*Threo*-7′-*O*-isopropylresvepterol acyclic dimer (**20**) UV (MeCN) λ_max_ (log ε) 228 (sh) (4.42), 285 (4.04), 306 (4.15), 326 (4.12), 343 (sh) (3.87) nm. ^1^H NMR (DMSO, 600 MHz) δ 0.96 (3H, d, *J* = 6.1 Hz, CH_3_-7'c), 1.03 (3H, d, *J* = 6.1 Hz, CH_3_-7'b), 3.47 (1H, hept, *J* = 6.1 Hz, H-7'a), 3.75 (6H, s, CH_3_O-11, CH_3_O-13), 4.51 (1H, d, *J* = 6.3 Hz, H-7′), 5.07 (1H, d, *J* = 6.3 Hz, H-8′), 5.98 (1H, t, *J* = 2.2 Hz, H-12′), 6.08 (2H, d, *J* = 2.2 Hz, H-10′, H-14′), 6.36 (1H, t, *J* = 2.3 Hz, H-12), 6.60 (2H, d, *J* = 8.5 Hz, H-3′, H-5′), 6.69 (3H, d, *J* = 2.3 Hz, H-10, H-14), 6.82 (2H, d, *J* = 8.8 Hz, H-3, H-5), 6.95 (1H, d, *J* = 16.4 Hz, H-8), 7.03 (2H, d, *J* = 8.5 Hz, H-2′, H-6′), 7.13 (1H, d, *J* = 16.4 Hz, H-7), 7.40 (2H, d, *J* = 8.8 Hz, H-2, H-6), 9.02 (2H, s, 11′OH, 13′OH), 9.23 (1H, s, 4′OH); ^13^C NMR (DMSO, 151 MHz) δ 21.2 (CH_3_-7′b), 22.8 (CH_3_-7′c), 54.8 (CH_3_O-11, CH_3_O-13), 69.4 (C-7′a), 81.8 (C-7′), 82.8 (C-8′), 99.3 (C-12), 101.4 (C-12′), 103.9 (C-10, C-14), 105.3 (C-10′, C-14′), 114.3 (C-3′, C-5′), 115.7 (C-3, C-5), 125.9 (C-8), 127.3 (C-2, C-6), 128.3 (C-7), 128.5 (C-2′, C-6′), 129.0 (C-1), 129.7 (C-1′), 139.1 (C-9), 140.2 (C-9′), 156.2 (C-4′), 157.5 (C-11′, C-13′), 157.8 (C-4), 160.5 (C-11,C-13). HR-ESI/MS analysis: *m/z* 541.2224 [M-H]^−^, (calcd for C_33_H_33_O_7_, 541.2226, ∆ = 0.4 ppm). MS/MS spectrum: CCMSLIB00009918875.

*Erythro*-pterostilbene acyclic dimer (**21**) UV (MeCN) λ_max_ (log ε) 228 (sh) (4.34), 285 (4.09), 306 (4.25), 326 (4.22), 344 (sh) (3.81) nm. ^1^H NMR (DMSO, 600 MHz) δ 3.60 (6H, s, CH_3_O-11′, CH_3_O-13′), 3.75 (6H, s, CH_3_O-11, CH_3_O-13), 4.73 (1H, t, *J* = 6.2, 4.6 Hz, H-7′), 5.20 (1H, d, *J* = 6.2 Hz, H-8′), 5.48 (1H, d, *J* = 4.6 Hz, 7′OH), 6.26 (1H, t, *J* = 2.3 Hz, H-12′), 6.29 (2H, d, *J* = 2.3 Hz, H-10′, H-14′), 6.37 (2H, t, *J* = 2.3 Hz, H-12), 6.59 (2H, d, *J* = 8.5 Hz, H-3′, H-5′), 6.69 (2H, d, *J* = 2.3 Hz, H-10, H-14), 6.89 (2H, d, *J* = 8.8 Hz, H-3, H-5), 6.95 (1H, d, *J* = 16.4 Hz, H-8), 7.01 (2H, d, *J* = 8.5 Hz, H-2′, H-6′), 7.13 (2H, d, *J* = 16.4 Hz, H-7), 7.41 (2H, d, *J* = 8.8 Hz, H-2, H-6), 9.19 (1H, s, 4′OH); ^13^C NMR (DMSO, 126 MHz) δ 54.6 (CH_3_O-11′, CH_3_O-13′), 54.8 (CH_3_O-11, CH_3_O-13), 75.7 (C-7′), 83.5 (C-8′), 98.6 (C-12′), 99.2 (C-12), 103.8 (C-10, C-14), 105.4 (C-10′, C-14′), 113.9 (C-3′, C-5′), 115.7 (C-3, C-5), 125.8 (C-8), 127.2 (C-2, C-6), 128.0 (C-2′, C-6′), 128.1 (C-7), 129.3 (C-1), 131.5 (C-1′), 139.1 (C-9), 156.1 (C-4′), 157.5 (C-4), 159.5 (C-11′, C-13′), 160.3 (C-11, C-13). HR-ESI/MS analysis: *m/z* 527.2078 [M-H]^−^, (calcd for C_32_H_31_O_7_, 527.2070, ∆ = 1.5 ppm). MS/MS spectrum: CCMSLIB00009918876.

11,11′,13,13′-Tetra-*O*-methyl-*trans*-δ-viniferin (**22**) UV (MeCN) λ_max_ (log ε) 227 (sh) (4.56), 285 (4.17), 311 (4.41), 333 (4.37), 350 (sh) (4.12) nm. ^1^H NMR (DMSO, 600 MHz) δ 3.63 (6H, s, CH_3_O-11′, CH_3_O-13′), 3.67 (6H, s, CH_3_O-11, CH_3_O-13), 4.54 (1H, d, *J* = 8.5 Hz, H-8′), 5.52 (1H, d, *J* = 8.5 Hz, H-7′), 6.28 (1H, t, *J* = 2.3 Hz, H-12), 6.31 (2H, d, *J* = 2.3 Hz, H-10′, H-14′), 6.36 (1H, t, *J* = 2.3 Hz, H-12′), 6.65 (2H, d, *J* = 2.3 Hz, H-10, H-14), 6.69 (2H, d, *J* = 8.6 Hz, H-3′, H-5′), 6.85 (1H, d, *J* = 8.3 Hz, H-5), 6.88 (1H, d, *J* = 16.4 Hz, H-8), 7.12 (1H, d, *J* = 1.8 Hz, H-2), 7.13 (2H, d, *J* = 8.6 Hz, H-2′, H-6′), 7.14 (1H, d, *J* = 16.4 Hz, H-7), 7.38 (1H, dd, *J* = 8.3, 1.8 Hz, H-6), 9.49 (1H, s, 4′OH). HR-ESI/MS analysis: *m/z* 509.1968 [M-H]^−^, (calcd for C_32_H_29_O_6_, 509.1964, ∆ = 0.8 ppm). MS/MS spectrum: CCMSLIB00009918877.

Parthenostilbenin A+B (**23**) Parthenostilbenin A (7S) ^1^H NMR (DMSO, 600 MHz) δ 2.59 (1H, t, *J* = 2.6 Hz, H-8′), 2.92 (3H, s, CH_3_O-7), 3.10 (1H, dd, *J* = 9.1, 2.6 Hz, H-8), 3.75 (1H, d, *J* = 9.1 Hz, H-7), 3.96 (1H, d, *J* = 2.6 Hz, H-7′), 5.60 (2H, d, *J* = 2.2 Hz, H-10′, H-14′), 5.91 (1H, t, *J* = 2.2 Hz, H-12′), 6.17 (1H, d, *J* = 2.1 Hz, H-12), 6.41 (1H, d, *J* = 2.1 Hz, H-14), 6.59–6.64 (8H, m, H-2, H-3, H-5, H-6, H-2′, H-3′, H-5′, H-6′), 8.80 (1H, s, 11OH), 8.92 (2H, s, 11′OH, 13′OH), 9.00 (1H, s, 13OH), 9.10 (1H, s, 4′OH), 9.30 (1H, s, 4OH); ^13^C NMR (DMSO, 151 MHz) δ 54.1 (C-7′), 55.5 (CH_3_O-7), 57.1 (C-8′), 58.9 (C-8), 85.9 (C-7), 99.7 (C-12′), 101.1 (C-12), 103.9 (C-10′, C-14′), 104.2 (C-14), 114.2 (C-3, C-5, C-3′, C-5′), 120.5 (C-10), 128.2 (C-2, C-6), 135.8 (C-1′), 152.6 (C-13), 153.7 (C-11), 156.2 (C-4), 157.8 (C-11′, C-13′). Parthenostilbenin B (7R) UV (MeCN) λ_max_ (log ε) 227 (sh) (4.46), 283 (3.74) nm. ^1^H NMR (DMSO, 600 MHz) δ 2.83 (3H, s, CH_3_O-7), 3.20 (2H, m, H-8, H-8′), 3.72 (1H, d, *J* = 8.9 Hz, H-7), 4.01 (1H, d, *J* = 2.7 Hz, H-7′), 5.42 (1H, d, *J* = 2.1 Hz, H-14), 5.92 (2H, d, *J* = 2.2 Hz, H-10′, H-14′), 5.99 (1H, t, *J* = 2.2 Hz, H-12′), 6.06 (1H, d, *J* = 2.1 Hz, H-12), 6.60 (2H, d, *J* = 8.6 Hz, H-3′, H-5′), 6.68 (4H, 2xd, *J* = 8.5 Hz, H-2′, H-3, H-5, H-6′), 6.90 (2H, d, *J* = 8.5 Hz, H-2, H-6), 8.74 (1H, s, 11OH), 8.79 (1H, s, 13OH), 9.00 (2H, s, 11′OH, 13′OH), 9.04 (1H, s, 4′OH), 9.35 (1H, s, 4OH); ^13^C NMR (DMSO, 151 MHz) δ 54.1 (C-7′), 55.4 (CH_3_O-7), 57.3 (C-8′), 59.4 (C-8), 85.7 (C-7), 99.7 (C-12′), 101.1 (C-12), 103.5 (C-14), 104.4 (C-10′, C-14′), 114.2 (C-3, C-5, C-3′, C-5′), 121.2 (C-10), 127.6 (C-2′, C-6′), 128.8 (C-2, C-6), 129.5 (C-1), 136.3 (C-1′), 153.7 (C-11), 154.7 (C-4′), 156.4 (C-4), 156.9 (C-13), 157.8 (C-11′, C-13′). HR-ESI/MS analysis: *m/z* 485.1606 [M-H]^−^, (calcd for C_29_H_25_O_7_, 485.1600, ∆ = 1.2 ppm). MS/MS spectrum: CCMSLIB00009918878.

*Erythro*-7′-*O*-isopropylresvepterol acyclic dimer (**24**) UV (MeCN) λ_max_ (log ε) 229 (sh) (4.08), 285 (3.75), 306 (3.92), 327 (3.91), 345 (sh) (3.62) nm. ^1^H NMR (DMSO, 600 MHz) δ 0.87 (3H, d, *J* = 6.0 Hz, CH_3_-7′c), 0.91 (3H, d, *J* = 6.2 Hz, CH_3_-7′b), 3.33 (1H, overlapped, H-7a), 3.75 (6H, s, CH_3_O-11, CH_3_O-13), 4.43 (1H, d, *J* = 6.5 Hz, H-7′), 5.04 (1H, d, *J* = 6.5 Hz, H-8′), 6.06 (1H, t, *J* = 2.2 Hz, H-12′), 6.22 (2H, d, *J* = 2.2 Hz, H-10′, H-14′), 6.36 (1H, t, *J* = 2.3 Hz, H-12), 6.67 (2H, d, *J* = 8.5 Hz, H-3′, H-5′), 6.68 (2H, d, *J* = 2.3 Hz, H-10, H-14), 6.73 (2H, d, *J* = 8.7 Hz, H-3, H-5), 6.92 (1H, d, *J* = 16.3 Hz, H-8), 7.10 (1H, d, *J* = 16.3 Hz, H-7), 7.16 (2H, d, *J* = 8.5 Hz, H-2′, H-6′), 7.36 (2H, d, *J* = 8.7 Hz, H-2, H-6), 9.09 (2H, s, 11′OH, 13′OH), 9.27 (1H, s, 4′OH); ^13^C NMR (DMSO, 151 MHz) δ 21.1 (CH_3_-7′c), 22.8 (CH_3_-7′b), 54.8 (CH_3_O-11, CH_3_O-13), 69.2 (C-7'a), 81.4, (C-7′), 81.7 (C-8′), 99.3 (C-12), 101.6 (C-12′), 103.9 (C-10, C-14), 105.5 (C-10′, C-14′), 114.2 (C-3′, C-5′), 115.7 (C-3, C-5), 125.9 (C-8), 127.3 (C-2, C-6), 128.3 (C-7), 128.7 (C-2′, C-6′), 129.2 (C-1), 130.1 (C-1′), 139.1 (C-9), 156.4 (C-4′), 157.1 (C-4), 157.5 (C-11′, C-13′), 160.5 (C-11, C-13). HR-ESI/MS analysis: *m/z* 541.2229 [M-H]^−^, (calcd for C_33_H_33_O_7_, 541.2226, ∆ = 0.6 ppm). MS/MS spectrum: CCMSLIB00009918879.

7-*O*-Butylleachianol G (**25**) UV (MeCN) λ_max_ (log ε) 229 (sh) (4.39), 280 (3.64) nm. ^1^H NMR (DMSO, 600 MHz) δ 0.74 (3H, t, *J* = 7.2 Hz, CH_3_-7d), 1.18 (4H, m, H-7b, H-7c), 2.75 (1H, dt, *J* = 9.8, 5.9 Hz, H-7a″), 3.01 (1H, dt, *J* = 9.8, 6.0 Hz, H-7a′), 3.13 (1H, dd, *J* = 8.8, 2.8 Hz, H-8), 3.32 (1H, overlapped, H-8′), 3.80 (1H, d, *J* = 8.8 Hz, H-7), 4.03 (1H, d, *J* = 2.5 Hz, H-7′), 5.31 (1H, d, *J* = 2.1 Hz, H-14), 5.92 (2H, d, *J* = 2.2 Hz, H-10′, H-14′), 5.98 (1H, t, *J* = 2.2 Hz, H-12′), 6.07 (1H, d, *J* = 2.1 Hz, H-12), 6.62 (2H, d, *J* = 8.6 Hz, H-3′, H-5′), 6.67 (2H, d, *J* = 8.5 Hz, H-3, H-5), 6.73 (2H, d, *J* = 8.6 Hz, H-2′, H-6′), 6.89 (2H, d, *J* = 8.5 Hz, H-2, H-6), 8.76 (1H, s, 11OH), 8.78 (1H, s, 13OH), 9.00 (2H, s, 11′OH, 13′OH), 9.04 (1H, s, 4′OH), 9.32 (1H, s, 4OH); ^13^C NMR (DMSO, 151 MHz) δ 13.6 (CH_3_-7d), 18.9 (C-7c), 31.0 (C-7b), 54.3 (C-7′), 57.5 (C-8′), 60.1 (C-8), 66.8 (C-7a), 83.9 (C-7), 100.1 (C-12′), 101.4 (C-12), 104.1 (C-14), 104.6 (C-10′, C-14′), 114.6 (C-3′, C-5′), 114.7 (C-3, C-5), 121.5 (C-10), 128.0 (C-2′, C-6′), 129.0 (C-2, C-6), 130.8 (C-1), 136.7 (C-1′), 145.5 (C-9), 150.2 (C-9′), 154.0 (C-11), 155.1 (C-4′), 156.7 (C-4), 157.2 (C-13), 158.2 (C-11′, C-13′). HR-ESI/MS analysis: *m/z* 527.2071 [M-H]^−^, (calcd for C_32_H_31_O_7_, 527.2070, ∆ = 0.2 ppm). MS/MS spectrum: CCMSLIB00009918880.

7-*O*-Butylleachianol F (**26**) UV (MeCN) λ_max_ (log ε) 229 (sh) (4.33), 282 (3.56) nm. ^1^H NMR (DMSO, 600 MHz) δ 0.78 (3H, t, *J* = 7.3 Hz, CH_3_-7d), 1.22 (2H, m, H-7c), 1.35 (2H, m, H-7b), 2.63 (1H, overlapped, H-8′), 2.95 (1H, dt, *J* = 9.5, 6.2 Hz, H-7a″), 3.05 (1H, m, H-7a′), 3.07 (1H, dd, *J* = 9.1, 2.7 Hz, H-8), 3.82 (1H, d, *J* = 9.1 Hz, H-7), 3.97 (1H, d, *J* = 2.4 Hz, H-7′), 5.61 (2H, d, *J* = 2.1 Hz, H-10′, H-14′), 5.92 (1H, t, *J* = 2.1 Hz, H-12′), 6.16 (1H, d, *J* = 2.1 Hz, H-12), 6.41 (1H, d, *J* = 2.1 Hz, H-14), 6.58 (4H, s, H-2, H-3, H-5, H-6), 6.63 (2H, d, *J* = 8.6 Hz, H-3′, H-5′), 6.68 (2H, d, *J* = 8.6 Hz, H-2′, H-6′), 8.78 (1H, s, 11OH), 8.92 (2H, s, 11′OH, 13′OH), 8.95 (1H, s, 13OH), 9.10 (1H, s, 4′OH), 9.27 (1H, s, 4OH); ^13^C NMR (DMSO, 151 MHz) δ 13.5 (CH_3_-7d), 18.6 (C-7c), 31.1 (C-7b), 54.1 (C-7′), 57.1 (C-8′), 59.2 (C-8), 67.1 (C-7a), 83.8 (C-7), 99.8 (C-12′), 101.1 (C-12), 104.1 (C-10′, C-14′), 104.6 (C-14), 114.4 (C-3′, C-5′), 114.5 (C-3, C-5), 120.5 (C-10), 127.8 (C-2′, C-6′), 128.0 (C-2, C-6), 131.1 (C-1), 136.0 (C-1′), 147.5 (C-9), 149.5 (C-9′), 153.8 (C-11), 154.9 (C-4′), 156.2 (C-4), 157.3 (C-13), 157.8 (C-11′, C-13′). HR-ESI/MS analysis: *m/z* 527.2074 [M-H]^−^, (calcd for C_32_H_31_O_7_, 527.2070, ∆ = 0.8 ppm). MS/MS spectrum: CCMSLIB00009918881.

*Erythro*-isoresveptero acyclic dimer (**27**) UV (MeCN) λ_max_ (log ε) 228 (sh) (4.21), 285 (3.95), 307 (4.08), 322 (4.08), 340 (sh) (3.81) nm. ^1^H NMR (DMSO, 600 MHz) δ 3.59 (6H, s, CH_3_O-11′, CH_3_O-13′), 4.73 (1H, dd, *J* = 6.3, 4.5 Hz, H-7′), 5.19 (1H, d, *J* = 6.3 Hz, H-8′), 5.48 (1H, d, *J* = 4.5 Hz, 7′OH), 6.10 (1H, t, *J* = 2.2 Hz, H-12), 6.25 (1H, t, *J* = 2.3 Hz, H-12′), 6.28 (2H, d, *J* = 2.3 Hz, H-10′, H-14′), 6.35 (2H, d, *J* = 2.2 Hz, H-10, H-14), 6.59 (2H, d, *J* = 8.6 Hz, H-3′, H-5′), 6.82 (1H, d, *J* = 16.3 Hz, H-8), 6.86 (2H, d, *J* = 8.9 Hz, H-3, H-5), 6.89 (1H, d, *J* = 16.3 Hz, H-7), 7.01 (2H, d, *J* = 8.6 Hz, H-2′, H-6′), 7.38 (2H, d, *J* = 8.9 Hz, H-2, H-6), 9.18 (2H, s, 11OH, 13OH), 9.20 (1H, s, 4′OH); ^13^C NMR (DMSO, 126 MHz) δ 54.7 (CH_3_O-11′, CH_3_O-13′), 75.7 (C-7′), 83.7 (C-8′), 98.6 (C-12′), 101.6 (C-12), 104.1 (C-10, C-14), 105.5 (C-10′, C-14′), 114.1 (C-3′, C-5′), 115.8 (C-3, C-5), 126.5 (C-8), 127.1 (C-7), 127.3 (C-2, C-6), 128.1 (C-2′, C-6′), 129.5 (C-1), 131.7 (C-1′), 138.9 (C-9), 156.3 (C-4′), 157.4 (C-4), 158.3 (C-11, C-13), 159.6 (C-11′, C-13′). HR-ESI/MS analysis: *m/z* 499.1769 [M-H]^−^, (calcd for C_30_H_27_O_7_, 499.1757, ∆ = 2.4 ppm). MS/MS spectrum: CCMSLIB00009918882.

*Erythro*-7′-*O*-butylresveratrol acyclic dimer (**28**) UV (MeCN) λ_max_ (log ε) 228 (sh) (4.29), 285 (4.02), 308 (4.18), 324 (4.18), 340 (sh) (3.90) nm. ^1^H NMR (DMSO, 600 MHz) δ 0.74 (3H, t, *J* = 7.4 Hz, CH_3_-7′d), 1.15 (2H, m, H-7′c), 1.32 (2H, m, H-7′b), 3.10 (1H, dt, *J* = 9.5, 6.3 Hz, H-7′a″), 3.21 (1H, dt, *J* = 9.5, 6.3 Hz, H-7′a′), 4.34 (1H, d, *J* = 6.4 Hz, H-7′), 5.10 (1H, d, *J* = 6.4 Hz, H-8′), 6.06 (1H, t, *J* = 2.2 Hz, H-12′), 6.09 (1H, t, *J* = 2.2 Hz, H-12), 6.20 (2H, d, *J* = 2.2 Hz, H-10′, H-14′), 6.34 (2H, d, *J* = 2.2 Hz, H-10, H-14), 6.68 (2H, d, *J* = 8.6 Hz, H-3′, H-5′), 6.71 (2H, d, *J* = 8.9 Hz, H-3, H-5), 6.79 (1H, d, *J* = 16.2 Hz, H-8), 6.87 (1H, d, *J* = 16.2 Hz, H-7), 7.13 (2H, d, *J* = 8.6 Hz, H-2′, H-6′), 7.33 (2H, d, *J* = 8.9 Hz, H-2, H-6), 9.08 (2H, s, 11′OH, 13′OH), 9.17 (2H, s, 11OH, 13OH), 9.29 (1H, s, 4′OH); ^13^C NMR (DMSO, 126 MHz) δ 13.2 (CH_3_-7'd), 18.3 (C-7'c), 30.8 (C-7'b), 67.5 (C-7'a), 81.4 (C-8′), 83.5 (C-7′), 101.4 (C-12, C-12′), 103.9 (C-10, C-14), 105.3 (C-10′, C-14′), 114.1 (C-3′, C-5′), 115.5 (C-3, C-5), 126.3 (C-8), 127.0 (C-7), 127.1 (C-2, C-6), 128.7 (C-2′, C-6′), 129.0 (C-1′), 129.2 (C-1), 138.5 (C-9), 156.2 (C-4′), 156.7 (C-4), 157.3 (C-11′, C-13′), 158.0 (C-11, C-13). HR-ESI/MS analysis: *m/z* 527.2076 [M-H]^−^, (calcd for C_32_H_31_O_7_, 527.2070, ∆ = 1.1 ppm). MS/MS spectrum: CCMSLIB00009918883.

7-*O*-Butyl-11′,13′-di-*O*-methylleachianol F (**29**) UV (MeCN) λ_max_ (log ε) 229 (sh) (4.16), 283 (3.51), 322 (3.33) nm. ^1^H NMR (DMSO, 600 MHz) δ 0.79 (3H, t, *J* = 7.4 Hz, CH_3_-7d), 1.24 (2H, m, *J* = 6.7 Hz, H-7c), 1.38 (2H, m, H-7b), 2.70 (1H, t, *J* = 3.6 Hz, H-8′), 3.01 (1H, dt, *J* = 9.3, 6.2 Hz, H-7a″), 3.08 (1H, dt, *J* = 9.3, 6.7 Hz, H-7a′), 3.12 (1H, dd, *J* = 9.1, 3.6 Hz, H-8), 3.91 (1H, d, *J* = 9.1 Hz, H-7), 4.05 (1H, d, *J* = 3.6 Hz, H-7′), 5.73 (2H, d, *J* = 2.3 Hz, H-10′, H-14′), 6.16 (1H, d, *J* = 2.1 Hz, H-12), 6.20 (1H, t, *J* = 2.3 Hz, H-12′), 6.45 (1H, d, *J* = 2.1 Hz, H-14), 6.58 (2H, d, *J* = 8.6 Hz, H-3, H-5), 6.62 (2H, d, *J* = 8.5 Hz, H-3′, H-5′), 6.65 (2H, d, *J* = 8.6 Hz, H-2, H-6), 6.67 (2H, d, *J* = 8.5 Hz, H-2′, H-6′), 8.77 (1H, s, 11OH), 8.97 (1H, s, 13OH), 9.09 (1H, s, 4′OH), 9.28 (1H, s, 4OH); ^13^C NMR (DMSO, 151 MHz) δ 13.5 (CH_3_-7d), 18.6 (C-7c), 31.1 (C-7b), 53.8 (C-7′), 54.6 (CH_3_O-11′, CH_3_O-13′), 57.5 (C-8′), 59.6 (C-8), 67.1 (C-7a), 84.0 (C-7), 97.0 (C-12′), 107.1 (C-12), 104.2 (C-10′, C-14′), 104.4 (C-14), 114.4 (C-3, C-5, C-3′, C-5′), 120.6 (C-10), 128.0 (C-2′, C-6′), 128.3 (C-2, C-6), 130.7 (C-1), 135.9 (C-1′), 146.7 (C-9), 148.9 (C-9′), 154.8 (C-4′), 156.3 (C-4), 157.5 (C-13), 159.7 (C-11′, C-13′). HR-ESI/MS analysis: *m/z* 555.2392 [M-H]^−^, (calcd for C_34_H_35_O_7_, 555.2383, ∆ = 1.6 ppm). MS/MS spectrum: CCMSLIB00009918884.

*Threo*-7′-*O*-butylresveratrol acyclic dimer (**30**) UV (MeCN) λ_max_ (log ε) 228 (sh) (4.34), 285 (4.12), 307 (4.30), 325 (4.28), 341 (sh) (4.03) nm. ^1^H NMR (DMSO, 600 MHz) δ 0.80 (3H, t, *J* = 7.4 Hz, CH_3_-7′d), 1.26 (2H, m, H-7′c), 1.41 (2H, m, H-7′b), 3.26 (2H, t, *J* = 6.4 Hz, H-7′a), 4.42 (1H, d, *J* = 6.6 Hz, H-7′), 5.13 (1H, d, *J* = 6.6 Hz, H-8′), 5.96 (1H, t, *J* = 2.2 Hz, H-12′), 6.05 (2H, d, *J* = 2.2 Hz, H-10′, H-14′), 6.10 (1H, t, *J* = 2.2 Hz, H-12), 6.36 (2H, d, *J* = 2.2 Hz, H-10, H-14), 6.60 (2H, d, *J* = 8.6 Hz, H-3′, H-5′), 6.81 (2H, d, *J* = 16.3 Hz, H-8), 6.82 (2H, d, *J* = 8.9 Hz, H-3, H-5), 6.89 (1H, d, *J* = 16.3 Hz, H-7), 7.00 (2H, d, *J* = 8.6 Hz, H-2′, H-6′), 7.38 (2H, d, *J* = 8.9 Hz, H-2, H-6), 9.02 (2H, s, 11′OH, 13′OH), 9.18 (2H, s, 11OH, 13OH), 9.26 (1H, s, 4′OH); ^13^C NMR (DMSO, 151 MHz) δ 13.7 (CH_3_-7′d), 18.8 (C-7′c), 31.4 (C-7′b), 68.1 (C-7′a), 82.8 (C-8′), 84.4 (C-7′), 101.7 (C-12′), 101.9 (C-12), 104.3 (C-10, C-14), 105.7 (C-10′, C-14′), 114.6 (C-3′, C-5′), 115.9 (C-3, C-5), 126.6 (C-8), 127.4 (C-7), 127.5 (C-2, C-6), 128.8 (C-1′), 128.9 (C-2′, C-6′), 129.5 (C-1), 139.0 (C-9), 140.2 (C-9′), 156.6 (C-4′), 157.7 (C-11′, C-13′), 157.8 (C-4), 158.5 (C-11, C-13). HR-ESI/MS analysis: *m/z* 527.2076 [M-H]^−^, (calcd for C_32_H_31_O_7_, 527.2070, ∆ = 1.3 ppm). MS/MS spectrum: CCMSLIB00009918885.

7-*O*-Butyl-11′,13′-di-*O*-methylleachianol G (**31**) UV (MeCN) λ_max_ (log ε) 229 (sh) (4.44), 282 (3.69) nm. ^1^H NMR (DMSO, 600 MHz) δ 0.75 (3H, t, *J* = 7.2 Hz, CH_3_-7d), 1.18 (2H, m, H-7c), 1.24 (2H, m, H-7b), 2.81 (1H, dt, *J* = 9.7, 5.9 Hz, H-7a″), 3.05 (1H, dt, *J* = 9.7, 6.1 Hz, H-7a′), 3.19 (1H, dd, *J* = 8.2, 3.3 Hz, H-8), 3.38 (1H, t, *J* = 3.3 Hz, H-8′), 3.65 (6H, s, CH_3_O-13′, CH_3_O-11′), 3.92 (1H, d, *J* = 8.2 Hz, H-7), 4.07 (1H, d, *J* = 3.3 Hz, H-7′), 5.45 (1H, d, *J* = 2.1 Hz, H-14), 6.08 (1H, d, *J* = 2.1 Hz, H-12), 6.10 (2H, d, *J* = 2.3 Hz, H-10′, H-14′), 6.29 (1H, t, *J* = 2.3 Hz, H-12′), 6.61 (2H, d, *J* = 8.5 Hz, H-3′, H-5′), 6.66 (2H, d, *J* = 8.5 Hz, H-3, H-5), 6.75 (2H, d, *J* = 8.5 Hz, H-2′, H-6′), 6.91 (2H, d, *J* = 8.5 Hz, H-2, H-6), 8.77 (1H, s, 11OH), 8.82 (1H, s, 13OH), 9.05 (1H, s, 4′OH), 9.31 (1H, s, 4OH); ^13^C NMR (DMSO, 151 MHz) δ 13.6 (CH_3_-7d), 18.9 (C-7c), 31.1 (C-7b), 54.3 (C-7′), 54.9 (CH_3_O-11′, CH_3_O-13′), 57.6 (C-8′), 60.2 (C-8), 67.0 (C-7a), 83.6 (C-7), 97.0 (C-12′), 101.5 (C-12), 103.8 (C-14), 104.9 (C-10′, C-14′), 114.6 (C-3′, C-5′), 114.7 (C-3, C-5), 121.4 (C-10), 128.1 (C-2′, C-6′), 128.9 (C-2, C-6), 130.7 (C-1), 136.5 (C-1′), 145.4 (C-9), 150.1 (C-9′), 154.0 (C-11), 155.1 (C-4′), 156.6 (C-4), 157.3 (C-13), 160.3 (C-11′, C-13′). HR-ESI/MS analysis: *m/z* 555.2390 [M-H]^−^, (calcd for C_34_H_35_O_7_, 555.2383, ∆ = 1.3 ppm). MS/MS spectrum: CCMSLIB00009918886.

7-*O*-Butyl-11′,13′-di-*O*-methylisoleachianol G (**32**) UV (MeCN) λ_max_ (log ε) 229 (sh) (4.41), 283 (3.80), 322 (3.66) nm. ^1^H NMR (DMSO, 600 MHz) δ 0.67 (3H, t, *J* = 7.3 Hz, CH_3_-7d), 1.03 (1H, m, H-7c″), 1.09 (1H, m, H-7c′), 1.19 (2H, m, H-7b), 2.39 (1H, overlapped, H-7a″), 2.73 (1H, dt, *J* = 8.3, 7.0 Hz, H-7a′), 3.48 (1H, d, *J* = 6.8 Hz, H-8′), 3.62 (1H, d, *J* = 10.4 Hz, H-7), 3.66 (6H, s, CH_3_O-13′, CH_3_O-11′), 3.82 (1H, dd, *J* = 10.4, 6.8 Hz, H-8), 4.11 (1H, overlapped, H-7′), 5.11 (1H, d, *J* = 2.0 Hz, H-14), 6.08 (1H, d, *J* = 2.0 Hz, H-12), 6.19 (2H, d, *J* = 2.3 Hz, H-10′, H-14′), 6.35 (1H, t, *J* = 2.3 Hz, H-12′), 6.65 (2H, d, *J* = 8.5 Hz, H-3′, H-5′), 6.73 (2H, d, *J* = 8.5 Hz, H-3, H-5), 6.79 (2H, d, *J* = 8.5 Hz, H-2′, H-6′), 7.02 (2H, d, *J* = 8.5 Hz, H-2, H-6), 8.84 (1H, s, 11OH), 8.85 (1H, s, 13OH), 9.13 (1H, s, 4′OH), 9.43 (1H, s, 4OH); ^13^C NMR (DMSO, 151 MHz) δ 13.3 (CH_3_-7d), 18.6 (C-7c), 31.1 (C-7b), 51.8 (C-7′), 52.2 (C-8), 54.6 (CH_3_O-11′, CH_3_O-13′), 58.7 (C-8′), 65.6 (C-7a), 80.1 (C-7), 96.9 (C-12′), 100.7 (C-12), 103.4 (C-14), 106.3 (C-10′, C-14′), 114.6 (C-3′, C-5′), 114.9 (C-3, C-5), 122.2 (C-10), 127.8 (C-2′, C-6′), 129.2 (C-2, C-6), 130.8 (C-1), 134.4 (C-1′), 144.9 (C-9), 146.2 (C-9′), 153.5 (C-11 or C-13), 155.3 (C-4′), 156.8 (C-4), 159.5 (C-11′, C-13′). HR-ESI/MS analysis: *m/z* 555.2395 [M-H]^−^, (calcd for C_34_H_35_O_7_, 555.2383, ∆ = 2.2 ppm). MS/MS spectrum: CCMSLIB00009918887.

*Threo*-7′-*O*-butylisoresveptero acyclic dimer (**33**) UV (MeCN) λ_max_ (log ε) 228 (sh) (4.13), 287 (3.95), 309 (4.09), 325 (4.08), 340 (sh) (3.84) nm. ^1^H NMR (DMSO, 600 MHz) δ 0.80 (3H, t, *J* = 7.4 Hz, CH_3_-7′d), 1.26 (2H, m, H-7′c), 1.42 (2H, m, H-7′b), 3.28 (2H, t, *J* = 6.4 Hz, H-7'a), 3.60 (6H, s, CH_3_O-11′, CH_3_O-13′), 4.50 (1H, d, *J* = 6.5 Hz, H-7′), 5.30 (1H, d, *J* = 6.5 Hz, H-8′), 6.10 (1H, t, *J* = 2.2 Hz, H-12), 6.24 (1H, t, *J* = 2.3 Hz, H-12′), 6.32 (2H, d, *J* = 2.3 Hz, H-10′, H-14′), 6.36 (2H, d, *J* = 2.2 Hz, H-10, H-14), 6.61 (2H, d, *J* = 8.5 Hz, H-3′, H-5′), 6.82 (1H, d, *J* = 16.3 Hz, H-8), 6.86 (2H, d, *J* = 8.8 Hz, H-3, H-5), 6.89 (1H, d, *J* = 16.3 Hz, H-7), 7.01 (2H, d, *J* = 8.5 Hz, H-2′, H-6′), 7.38 (2H, d, *J* = 8.8 Hz, H-2, H-6), 9.18 (2H, s, 11OH, 13OH), 9.27 (1H, s, 4′OH); ^13^C NMR (DMSO, 151 MHz) δ 13.5 (CH_3_-7′d), 18.6 (C-7′c), 31.1 (C-7'b), 54.7 (CH_3_O-11′, CH_3_O-13′), 67.8 (C-7′a), 82.4 (C-8′), 84.0 (C-7′), 98.8 (C-12′), 101.6 (C-12), 104.1 (C-10, C-14), 105.5 (C-10′, C-14′), 114.3 (C-3′, C-5′), 115.7 (C-3, C-5), 126.6 (C-8), 127.1 (C-7), 127.3 (C-2, C-6), 128.6 (C-1′), 128.7 (C-2′, C-6′), 129.5 (C-1), 138.7 (C-9), 140.5 (C-9′), 156.4 (C-4′), 157.3 (C-4), 158.2 (C-11, C-13), 159.6 (C-11′, C-13′). HR-ESI/MS analysis: *m/z* 555.2401 [M-H]^−^, (calcd for C_34_H_35_O_7_, 555.2383, ∆ = 3.2 ppm). MS/MS spectrum: CCMSLIB00009918888.

*Erythro*-7′-*O*-butylisoresveptero acyclic dimer (**34**) UV (MeCN) λ_max_ (log ε) 228 (sh) (3.99), 288 (3.75), 314 (3.90), 340 (sh) (3.63) nm. ^1^H NMR (DMSO, 600 MHz) δ 0.73 (3H, t, *J* = 7.4 Hz, CH_3_-7′d), 1.14 (2H, h, *J* = 7.3 Hz, H-7′c), 1.32 (2H, m, H-7′b), 3.12 (1H, dt, *J* = 9.5, 6.2 Hz, H-7′a″), 3.23 (1H, dt, *J* = 9.5, 6.2 Hz, H-7′a′), 3.67 (6H, s, CH_3_O-11′, CH_3_O-13′), 4.42 (1H, d, *J* = 6.5 Hz, H-7′), 5.28 (1H, d, *J* = 6.5 Hz, H-8′), 6.10 (1H, t, *J* = 2.2 Hz, H-12), 6.34 (2H, d, *J* = 2.2 Hz, H-10, H-14), 6.35 (1H, t, *J* = 2.2 Hz, H-12′), 6.48 (2H, d, *J* = 2.2 Hz, H-10′, H-14′), 6.69 (2H, d, *J* = 8.5 Hz, H-3′, H-5′), 6.77 (2H, d, *J* = 8.8 Hz, H-3, H-5), 6.80 (1H, d, *J* = 16.6 Hz, H-8), 6.87 (1H, d, *J* = 16.6 Hz, H-7), 7.15 (2H, d, *J* = 8.5 Hz, H-2′, H-6′), 7.34 (2H, d, *J* = 8.8 Hz, H-2, H-6), 9.17 (2H, s, 11OH, 13OH), 9.31 (1H, s, 4′OH). HR-ESI/MS analysis: *m/z* 555.2403 [M-H]^−^, (calcd for C_34_H_35_O_7_, 555.2383, ∆ = 3.6 ppm). MS/MS spectrum: CCMSLIB00009918889.

*Erythro*-7′-*O*-butylresveptero acyclic dimer (**35**) UV (MeCN) λ_max_ (log ε) 228 (sh) (4.46), 288 (4.23), 308 (4.38), 325 (4.38), 340 (sh) (4.12) nm. ^1^H NMR (DMSO, 600 MHz) δ 0.74 (3H, t, *J* = 7.4 Hz, CH_3_-7′d), 1.15 (2H, m, H-7′c), 1.32 (2H, m, H-7′b), 3.10 (1H, dt, *J* = 9.4, 6.3 Hz, H-7′a″), 3.21 (1H, dt, *J* = 9.7, 6.4 Hz, H-7′a′), 3.75 (6H, s, CH_3_O-11, CH_3_O-13), 4.33 (1H, d, *J* = 6.5 Hz, H-7′), 5.12 (1H, d, *J* = 6.5 Hz, H-8′), 6.06 (1H, t, *J* = 2.2 Hz, H-12′), 6.21 (2H, d, *J* = 2.2 Hz, H-10′, H-14′), 6.36 (1H, t, *J* = 2.2 Hz, H-12), 6.68 (2H, d, *J* = 8.3 Hz, H-3′, H-5′), 6.68 (2H, d, *J* = 2.2 Hz, H-10, H-14), 6.74 (2H, d, *J* = 8.8 Hz, H-3, H-5), 6.92 (1H, d, *J* = 16.3 Hz, H-8), 7.10 (1H, d, *J* = 16.3 Hz, H-7), 7.13 (2H, d, *J* = 8.3 Hz, H-2′, H-6′), 7.36 (2H, d, *J* = 8.8 Hz, H-2, H-6), 9.09 (2H, s, 11′OH, 13′OH), 9.30 (1H, s, 4′OH); ^13^C NMR (DMSO, 126 MHz) δ 13.3 (CH_3_-7′d), 18.4 (C-7′c), 30.9 (C-7′b), 55.0 (CH_3_O-11, CH_3_O-13), 67.7 (C-7'a), 81.6 (C-8′), 83.7 (C-7′), 99.1 (C-12), 101.6 (C-12′), 103.9 (C-10, C-14), 105.3 (C-10′, C-14′), 114.3 (C-3′, C-5′), 115.7 (C-3, C-5), 125.9 (C-8), 127.3 (C-2, C-6), 128.3 (C-7), 128.9 (C-2′, C-6′), 129.2 (C-1, C-1′), 139.1 (C-9), 156.4 (C-4′), 157.1 (C-4), 157.5 (C-11′, C-13′), 160.4 (C-11, C-13). HR-ESI/MS analysis: *m/z* 555.2400 [M-H]−, (calcd for C_34_H_35_O_7_, 555.2383, ∆ = 3.1 ppm). MS/MS spectrum: CCMSLIB00009918891.

7-*O*-Butyl-11,11′,13,13′-tetra-*O*-methylleachianol G (**36**) UV (MeCN) λ_max_ (log ε) 229 (sh) (3.87), 253 (3.56), 322 (3.49) nm. ^1^H NMR (DMSO, 600 MHz) δ 0.75 (3H, t, *J* = 7.2 Hz, CH_3_-7d), 1.19 (2H, m, H-7c), 1.25 (2H, m, H-7b), 2.86 (1H, dt, *J* = 9.8, 5.8 Hz, H-7a″), 3.10 (1H, dt, *J* = 9.6, 6.1 Hz, H-7a′), 3.24 (1H, dd, *J* = 8.4, 3.0 Hz, H-8), 3.40 (1H, t, *J* = 3.0 Hz, H-8′), 3.51 (3H, s, CH_3_O-13), 3.55 (3H, s, CH_3_O-11), 3.66 (6H, s, CH_3_O-11′, CH_3_O-13′), 3.96 (1H, d, *J* = 8.5 Hz, H-7), 4.12 (1H, d, *J* = 3.0 Hz, H-7′), 5.53 (1H, d, *J* = 2.2 Hz, H-14), 6.09 (2H, d, *J* = 2.3 Hz, H-10′, H-14′), 6.31 (1H, t, *J* = 2.3 Hz, H-12′), 6.34 (1H, d, *J* = 2.2 Hz, H-12), 6.63 (2H, d, *J* = 8.5 Hz, H-3′, H-5′), 6.70 (2H, d, *J* = 8.4 Hz, H-3, H-5), 6.73 (2H, d, *J* = 8.5 Hz, H-2′, H-6′), 6.93 (2H, d, *J* = 8.4 Hz, H-2, H-6), 9.10 (1H, s, 4′OH), 9.35 (1H, s, 4OH); ^13^C NMR (DMSO, 151 MHz) δ 13.3 (CH_3_-7d), 18.6 (C-7c), 30.7 (C-7b), 54.5 (C-7′), 54.5 (CH_3_O-13), 54.6 (CH_3_O-11′, CH_3_O-13′), 54.8 (CH_3_O-11), 57.5 (C-8′), 60.3 (C-8), 66.8 (C-7a), 83.5 (C-7), 96.8 (C-12′), 97.4 (C-12), 101.8 (C-14), 104.6 (C-10′, C-14′), 114.4 (C-3, C-5), 114.6 (C-3′, C-5′), 127.6 (C-2′, C-6′), 128.7 (C-2, C-6). HR-ESI/MS analysis: *m/z* 583.2706 [M-H]^−^, (calcd for C_36_H_39_O_7_, 583.2696, ∆ = 1.7 ppm). MS/MS spectrum: CCMSLIB00009918890.

*Threo*-7′-*O*-butylresveptero acyclic dimer (**37**) UV (MeCN) λ_max_ (log ε) 228 (sh) (4.32), 286 (4.12), 308 (4.28), 326 (4.27), 343 (sh) (3.97) nm. ^1^H NMR (DMSO, 600 MHz) δ 0.80 (3H, t, *J* = 7.4 Hz, CH_3_-7′d), 1.26 (2H, m, H-7'c), 1.41 (2H, m, H-7′b), 3.26 (2H, t, *J* = 6.4 Hz, H-7'a), 3.75 (6H, s, CH_3_O-11, CH_3_O-13), 4.42 (1H, d, *J* = 6.6 Hz, H-7′), 5.14 (1H, d, *J* = 6.6 Hz, H-8′), 5.97 (1H, t, *J* = 2.2 Hz, H-12′), 6.06 (2H, d, *J* = 2.2 Hz, H-10′, H-14′), 6.36 (1H, t, *J* = 2.3 Hz, H-12), 6.61 (2H, d, *J* = 8.5 Hz, H-3′, H-5′), 6.70 (2H, d, *J* = 2.3 Hz, H-10, H-14), 6.84 (2H, d, *J* = 8.8 Hz, H-3, H-5), 6.95 (1H, d, *J* = 16.3 Hz, H-8), 7.00 (2H, d, *J* = 8.5 Hz, H-2′, H-6′), 7.13 (1H, d, *J* = 16.3 Hz, H-7), 7.41 (2H, d, *J* = 8.8 Hz, H-2, H-6), 9.03 (2H, s, 11′OH, 13′OH), 9.27 (1H, s, 4′OH); ^13^C NMR (DMSO, 151 MHz) δ 13.5 (CH_3_-7′d), 18.6 (C-7′c), 31.1 (C-7′b), 55.0 (CH_3_O-11, CH_3_O-13), 68.0 (C-7′a), 82.6 (C-8′), 84.2 (C-7′), 99.3 (C-12), 101.4 (C-12′), 103.9 (C-10, C-14), 105.5 (C-10′, C-14′), 114.3 (C-3′, C-5′), 115.7 (C-3, C-5), 125.9 (C-8), 127.3 (C-2, C-6), 128.3 (C-7), 128.5 (C-1′), 128.7 (C-2′, C-6′), 129.1 (C-1), 139.1 (C-9), 140.0 (C-9′), 156.4 (C-4′), 157.5 (C-11′, C-13′), 157.8 (C-4), 160.5 (C-11, C-13). HR-ESI/MS analysis: *m/z* 555.2394 [M-H]^−^, (calcd for C_34_H_35_O_7_, 555.2383, ∆ = 2.0 ppm). MS/MS spectrum: CCMSLIB00009918892.

7-*O*-Butyl-11,11′,13,13′-tetra-*O*-methylleachianol F (**38**) UV (MeCN) λ_max_ (log ε) 229 (sh) (4.02), 285 (3.46), 320 (3.39) nm. ^1^H NMR (DMSO, 600 MHz) δ 0.79 (3H, t, *J* = 7.4 Hz, CH_3_-7d), 1.26 (2H, m, H-7c), 1.39 (2H, m, H-7b), 2.76 (1H, t, *J* = 3.7 Hz, H-8′), 3.02 (1H, dt, *J* = 9.4, 6.8 Hz, H-7a″), 3.12 (1H, dt, *J* = 9.5, 6.4 Hz, H-7a′), 3.24 (1H, dd, *J* = 9.1, 3.7 Hz, H-8), 3.57 (3H, s, CH_3_O-11), 3.57 (6H, s, CH_3_O-11′, CH_3_O-13′), 3.78 (3H, s, CH_3_O-13), 3.97 (1H, d, *J* = 9.1 Hz, H-7), 4.09 (1H, overlapped, H-7′), 5.71 (2H, d, *J* = 2.2 Hz, H-10′, H-14′), 6.21 (1H, t, *J* = 2.3 Hz, H-12′), 6.45 (1H, d, *J* = 2.1 Hz, H-12), 6.60 (2H, d, *J* = 8.5 Hz, H-3, H-5), 6.62 (2H, d, *J* = 8.7 Hz, H-3′, H-5′), 6.65 (2H, d, *J* = 8.7 Hz, H-2′, H-6′), 6.69 (2H, d, *J* = 8.5 Hz, H-2, H-6), 6.72 (1H, d, *J* = 2.1 Hz, H-14), 9.13 (1H, s, 4′OH), 9.30 (1H, s, 4OH); ^13^C NMR (DMSO, 151 MHz) δ 13.5 (CH_3_-7d), 18.6 (C-7c), 31.1 (C-7b), 54.1 (C-7′), 54.6 (CH_3_O-11, CH_3_O-11′, CH_3_O-13′), 54.8 (CH_3_O-13), 57.5 (C-8′), 59.6 (C-8), 67.1 (C-7a), 84.2 (C-7), 97.0 (C-12′), 97.5 (C-12), 102.5 (C-14), 104.2 (C-10′, C-14′), 114.4 (C-3, C-5), 114.7 (C-3′, C-5′), 127.6 (C-2′, C-6′), 128.3 (C-2, C-6). HR-ESI/MS analysis: *m/z* 583.2699 [M-H]^−^, (calcd for C_36_H_39_O_7_, 583.2696, ∆ = 0.5 ppm). MS/MS spectrum: CCMSLIB00009918893.

*Erythro*-7′-*O*-butylpterostilbene acyclic dimer (**39**) UV (MeCN) λ_max_ (log ε) 228 (sh) (3.98), 285 (3.68), 314 (3.87), 344 (sh) (3.46) nm. ^1^H NMR (DMSO, 600 MHz) δ 0.73 (3H, t, *J* = 7.4 Hz, CH_3_-7′d), 1.14 (2H, h, *J* = 7.3 Hz, H-7′c), 1.32 (2H, dq, *J* = 8.2, 6.3 Hz, H-7′b), 3.12 (1H, dt, *J* = 9.5, 6.3 Hz, H-7′a″), 3.23 (1H, dt, *J* = 9.5, 6.3 Hz, H-7′a′), 3.67 (6H, s, CH_3_O-11′, CH_3_O-13′), 3.75 (6H, s, CH_3_O-11, CH_3_O-13), 4.42 (1H, d, *J* = 6.4 Hz, H-7′), 5.30 (1H, d, *J* = 6.4 Hz, H-8′), 6.36 (1H, t, *J* = 2.3 Hz, H-12′), 6.36 (1H, t, *J* = 2.4 Hz, H-12), 6.48 (2H, d, *J* = 2.3 Hz, H-10′, H-14′), 6.68 (2H, d, *J* = 2.4 Hz, H-10, H-14), 6.69 (2H, d, *J* = 8.6 Hz, H-3′, H-5′), 6.79 (2H, d, *J* = 8.8 Hz, H-3, H-5), 6.93 (1H, d, *J* = 16.3 Hz, H-8), 7.10 (1H, d, *J* = 16.3 Hz, H-7), 7.15 (2H, d, *J* = 8.6 Hz, H-2′, H-6′), 7.36 (2H, d, *J* = 8.8 Hz, H-2, H-6), 9.31 (1H, s, 4′OH); ^13^C NMR (DMSO, 126 MHz) δ 13.3 (CH_3_-7′d), 18.4 (C-7′c), 31.1 (C-7′b), 54.8 (CH_3_O-11, CH_3_O-13, CH_3_O-11′, CH_3_O-13′), 67.7 (C-7'a), 81.4 (C-8′), 83.3 (C-7′), 99.0 (C-12′), 99.2 (C-12), 103.9 (C-10, C-14), 105.6 (C-10′, C-14′), 114.3 (C-3′, C-5′), 115.8 (C-3, C-5), 126.0 (C-8), 127.3 (C-2, C-6), 128.2 (C-7), 128.9 (C-2′, C-6′). HR-ESI/MS analysis: *m/z* 583.2711 [M-H]^−^, (calcd for C_36_H_39_O_7_, 583.2696, ∆ = 2.6 ppm). MS/MS spectrum: CCMSLIB00009918894.

*Threo*-7′-*O*-butylpterostilbene acyclic dimer (**40**) UV (MeCN) λ_max_ (log ε) 228 (sh) (4.56), 285 (4.35), 308 (4.53), 324 (4.53), 341 (sh) (4.27) nm. ^1^H NMR (DMSO, 600 MHz) δ 0.79 (3H, t, *J* = 7.4 Hz, CH_3_-7′d), 1.25 (2H, m, H-7′c), 1.42 (2H, m, H-7′b), 3.28 (2H, t, *J* = 6.4 Hz, H-7′a), 3.60 (6H, s, CH_3_O-11′, CH_3_O-13′), 3.75 (6H, s, CH_3_O-11, CH_3_O-13), 4.50 (1H, d, *J* = 6.5 Hz, H-7′), 5.31 (1H, d, *J* = 6.5 Hz, H-8′), 6.25 (1H, t, *J* = 2.3 Hz, H-12′), 6.33 (2H, d, *J* = 2.3 Hz, H-10′, H-14′), 6.37 (1H, t, *J* = 2.3 Hz, H-12), 6.61 (2H, d, *J* = 8.5 Hz, H-3′, H-5′), 6.70 (2H, d, *J* = 2.3 Hz, H-10, H-14), 6.89 (2H, d, *J* = 8.8 Hz, H-3, H-5), 6.96 (1H, d, *J* = 16.3 Hz, H-8), 7.01 (2H, d, *J* = 8.5 Hz, H-2′, H-6′), 7.13 (1H, d, *J* = 16.3 Hz, H-7), 7.41 (2H, d, *J* = 8.8 Hz, H-2, H-6), 9.27 (1H, s, 4′OH); ^13^C NMR (DMSO, 151 MHz) δ 13.5 (CH_3_-7′d), 18.6 (C-7′c), 31.1 (C-7′b), 54.6 (CH_3_O-11′, CH_3_O-13′), 55.0 (CH_3_O-11, CH_3_O-13), 67.8 (C-7′a), 82.4 (C-8′), 84.0 (C-7′), 98.8 (C-12′), 99.3 (C-12), 103.9 (C-10, C-14), 105.5 (C-10′, C-14′), 114.3 (C-3′, C-5′), 115.8 (C-3, C-5), 126.0 (C-8), 127.4 (C-2, C-6), 128.3 (C-7), 128.5 (C-1′), 128.7 (C-2′, C-6′), 129.2 (C-1), 139.1 (C-9), 140.4 (C-9′), 156.4 (C-4′), 157.5 (C-4), 159.5 (C-11′, C-13′), 160.4 (C-11, C-13). HR-ESI/MS analysis: *m/z* 583.2713 [M-H]^−^, (calcd for C_36_H_39_O_7_, 583.2696, ∆ = 2.9 ppm). MS/MS spectrum: CCMSLIB00009918895.

7-*O*-Isobutylleachianol G (**41**) UV (MeCN) λ_max_ (log ε) 229 (sh) (4.37), 282 (3.62) nm. ^1^H NMR (DMSO, 600 MHz) δ 0.70 (3H, d, *J* = 6.6 Hz, CH_3_-7d), 0.72 (3H, d, *J* = 6.6 Hz, CH_3_-7c), 1.52 (1H, hept, *J* = 6.6 Hz, H-7b), 2.77 (1H, dd, *J* = 9.1, 6.5 Hz, H-7a′), 3.15 (1H, dd, *J* = 8.9, 2.7 Hz, H-8), 3.36 (1H, t, *J* = 2.7 Hz, H-8′), 3.77 (1H, d, *J* = 8.9 Hz, H-7), 4.03 (1H, d, *J* = 2.7 Hz, H-7′), 5.33 (1H, d, *J* = 2.1 Hz, H-14), 5.93 (2H, d, *J* = 2.2 Hz, H-10′, H-14′), 5.99 (1H, t, *J* = 2.2 Hz, H-12′), 6.07 (1H, d, *J* = 2.1 Hz, H-12), 6.61 (2H, d, *J* = 8.4 Hz, H-3′, H-5′), 6.67 (2H, d, *J* = 8.5 Hz, H-3, H-5), 6.72 (2H, d, *J* = 8.4 Hz, H-2′, H-6′), 6.88 (2H, d, *J* = 8.5 Hz, H-2, H-6), 8.76 (1H, s, 11OH), 8.78 (1H, s, 13OH), 9.00 (2H, s, 11′OH, 13′OH), 9.04 (1H, s, 4′OH), 9.32 (1H, s, 4OH); ^13^C NMR (DMSO, 151 MHz) δ 19.4 (C-7d), 19.4 (C-7c), 27.8 (C-7b), 54.2 (C-7′), 57.4 (C-8′), 60.2 (C-8), 74.6 (C-7a), 84.3 (C-7), 100.1 (C-12′), 101.4 (C-12), 104.1 (C-14), 104.6 (C-10′, C-14′), 114.6 (C-3′, C-5′), 114.7 (C-3, C-5), 121.5 (C-10), 128.0 (C-2′, C-6′), 129.0 (C-2, C-6), 130.7 (C-1), 136.7 (C-1′), 145.5 (C-9), 150.2 (C-9′), 154.0 (C-11), 155.1 (C-4′), 156.7 (C-4), 157.2 (C-13), 158.2 (C-11′, C-13′). HR-ESI/MS analysis: *m/z* 527.2072 [M-H]^-^, (calcd for C_32_H_31_O_7_, 527.2070, ∆ = 0.4 ppm). MS/MS spectrum: CCMSLIB00009918896.

*Erythro*-7′-*O*-isobutylresveratrol acyclic dimer (**42**) UV (MeCN) λ_max_ (log ε) 227 (sh) (4.26), 286 (3.95), 308 (4.08), 325 (4.06), 341 (sh) (3.81) nm. ^1^H NMR (DMSO, 600 MHz) δ 0.69 (3H, d, *J* = 6.6 Hz, CH_3_-7′c), 0.69 (3H, d, *J* = 6.6 Hz, CH_3_-7′d), 1.63 (1H, hept, *J* = 6.6 Hz, H-7′b), 2.85 (1H, dd, *J* = 9.1, 6.6 Hz, H-7′a″), 2.99 (1H, dd, *J* = 9.1, 6.6 Hz, H-7′a′), 4.33 (1H, d, *J* = 6.6 Hz, H-7′), 5.09 (1H, d, *J* = 6.6 Hz, H-8′), 6.06 (1H, t, *J* = 2.2 Hz, H-12′), 6.09 (1H, t, *J* = 2.1 Hz, H-12), 6.22 (2H, d, *J* = 2.2 Hz, H-10′, H-14′), 6.34 (2H, d, *J* = 2.1 Hz, H-10, H-14), 6.69 (2H, d, *J* = 8.5 Hz, H-3′, H-5′), 6.72 (2H, d, *J* = 8.8 Hz, H-3, H-5), 6.79 (1H, d, *J* = 16.3 Hz, H-8), 6.86 (1H, d, *J* = 16.3 Hz, H-7), 7.14 (2H, d, *J* = 8.5 Hz, H-2′, H-6′), 7.33 (2H, d, *J* = 8.8 Hz, H-2, H-6), 9.07 (2H, s, 11′OH, 13′OH), 9.17 (2H, s, 11OH, 13OH), 9.30 (1H, s, 4′OH); ^13^C NMR (DMSO, 151 MHz) δ 19.1 (CH_3_-7′d), 19.1 (CH_3_-7′c), 27.9 (C-7′b), 75.1 (C-7'a), 81.9 (C-8′), 84.0 (C-7′), 101.8 (C-12′), 101.9 (C-12), 104.3 (C-10, C-14), 105.7 (C-10′, C-14′), 114.6 (C-3′, C-5′), 115.9 (C-3, C-5), 126.7 (C-8), 127.3 (C-7), 127.4 (C-2, C-6), 129.0 (C-2′, C-6′), 129.4 (C-1′), 129.6 (C-1), 139.0 (C-9), 141.0 (C-9′), 156.8 (C-4′), 157.2 (C-4), 157.8 (C-11′, C-13′), 158.5 (C-11, C-13). HR-ESI/MS analysis: *m/z* 527.2076 [M-H]^−^, (calcd for C_32_H_31_O_7_, 527.2070, ∆ = 1.1 ppm). MS/MS spectrum: CCMSLIB00009918897.

7-*O*-Isobutyl-11′,13′-di-*O*-methylleachianol F (**43**) UV (MeCN) λ_max_ (log ε) 229 (sh) (4.44), 285 (3.81), 320 (3.62) nm. ^1^H NMR (DMSO, 600 MHz) δ 0.77 (3H, d, *J* = 6.7 Hz, CH_3_-7d), 0.78 (3H, d, *J* = 6.7 Hz, CH_3_-7c), 1.70 (1H, dp, *J* = 13.3, 6.6 Hz, H-7b), 2.70 (1H, t, *J* = 3.4 Hz, H-8′), 2.82 (2H, m, H-7a), 3.12 (1H, dd, *J* = 9.1, 3.4 Hz, H-8), 3.57 (5H, s, CH_3_O-13′, CH_3_O-11′), 3.90 (1H, d, *J* = 9.1 Hz, H-7), 4.06 (1H, d, *J* = 3.4 Hz, H-7′), 5.73 (2H, d, *J* = 2.3 Hz, H-10′, H-14′), 6.17 (1H, d, *J* = 2.1 Hz, H-12), 6.20 (1H, t, *J* = 2.3 Hz, H-12′), 6.47 (1H, d, *J* = 2.1 Hz, H-14), 6.59 (2H, d, *J* = 8.5 Hz, H-3, H-5), 6.62 (2H, d, *J* = 8.5 Hz, H-3′, H-5′), 6.65 (1H, d, *J* = 8.5 Hz, H-2, H-6), 6.67 (2H, d, *J* = 8.5 Hz, H-2′, H-6′), 8.79 (1H, s, 11OH), 8.97 (1H, s, 13OH), 9.10 (1H, s, 4′OH), 9.29 (1H, s, 4OH); ^13^C NMR (DMSO, 151 MHz) δ 19.1 (CH_3_-7d), 19.1 (CH_3_-7c), 27.7 (C-7b), 53.8 (C-7′), 54.6 (CH_3_O-11′, CH_3_O-13′), 57.3 (C-8′), 59.7 (C-8), 74.5 (C-7a), 84.2 (C-7), 97.0 (C-12′), 101.2 (C-12), 104.2 (C-10′, C-14′), 104.6 (C-14), 114.4 (C-3, C-5, C-3′, C-5′), 120.7 (C-10), 128.1 (C-2, C-6, C-2′, C-6′), 130.8 (C-1), 135.7 (C-1′), 146.7 (C-9), 149.2 (C-9′), 153.7 (C-11), 154.8 (C-4′), 156.4 (C-4), 157.5 (C-13), 159.8 (C-11′, C-13′). HR-ESI/MS analysis: *m/z* 555.2393 [M-H]^−^, (calcd for C_34_H_35_O_7_, 555.2383, ∆ = 1.8 ppm). MS/MS spectrum: CCMSLIB00009918898.

*Threo*-7′-*O*-isobutylresveratrol acyclic dimer (**44**) UV (MeCN) λ_max_ (log ε) 229 (sh) (4.36), 285 (4.15), 307 (4.31), 326 (4.30), 341 (sh) (4.05) nm. ^1^H NMR (DMSO, 600 MHz) δ 0.79 (3H, d, *J* = 6.6 Hz, CH_3_-7′d), 0.79 (3H, d, *J* = 6.6 Hz, CH_3_-7'c), 1.73 (1H, hept, *J* = 6.6 Hz, H-7′b), 3.03 (2H, d, *J* = 6.6 Hz, H-7'a), 4.41 (1H, d, *J* = 6.6 Hz, H-7′), 5.13 (1H, d, *J* = 6.6 Hz, H-8′), 5.97 (1H, t, *J* = 2.2 Hz, H-12′), 6.06 (2H, d, *J* = 2.2 Hz, H-10′, H-14′), 6.10 (1H, t, *J* = 2.1 Hz, H-12), 6.36 (2H, d, *J* = 2.1 Hz, H-10, H-14), 6.61 (2H, d, *J* = 8.6 Hz, H-3′, H-5′), 6.81 (2H, d, *J* = 16.3 Hz, H-8), 6.82 (2H, d, *J* = 8.8 Hz, H-3, H-5), 6.89 (1H, d, *J* = 16.3 Hz, H-7), 6.99 (2H, d, *J* = 8.6 Hz, H-2′, H-6′), 7.39 (2H, d, *J* = 8.8 Hz, H-2, H-6), 9.02 (2H, s, 11′OH, 13′OH), 9.18 (2H, s, 11OH, 13OH), 9.26 (1H, s, 4′OH); ^13^C NMR (DMSO, 151 MHz) δ 19.2 (CH_3_-7′d), 19.3 (CH_3_-7′c), 28.0 (C-7'b), 75.4 (C-7′a), 82.9 (C-8′), 84.6 (C-7′), 101.7 (C-12′), 101.9 (C-12), 104.3 (C-10, C-14), 105.6 (C-10′, C-14′), 114.6 (C-3′, C-5′), 115.8 (C-3, C-5), 126.6 (C-8), 127.4 (C-7), 127.5 (C-2, C-6), 128.7 (C-1′), 128.9 (C-2′, C-6′), 129.4 (C-1), 139.0 (C-9), 140.2 (C-9′), 156.6 (C-4′), 157.7 (C-11′, C-13′), 157.8 (C-4), 158.5 (C-11, C-13). HR-ESI/MS analysis: *m/z* 527.2077 [M-H]^−^, (calcd for C_32_H_31_O_7_, 527.2070, ∆ = 1.3 ppm). MS/MS spectrum: CCMSLIB00009918899.

7-*O*-Isobutyl-11′,13′-di-*O*-methylleachianol G (**45**) UV (MeCN) λ_max_ (log ε) 228 (sh) (4.43), 285 (3.65) nm. ^1^H NMR (DMSO, 600 MHz) δ 0.71 (3H, d, *J* = 6.7 Hz, CH_3_-7d), 0.73 (3H, d, *J* = 6.7 Hz, CH_3_-7c), 1.56 (1H, hept, *J* = 6.7 Hz, H-7b), 2.57 (1H, dd, *J* = 9.0, 6.7 Hz, H-7a″), 2.81 (1H, dd, *J* = 9.0, 6.7 Hz, H-7a′), 3.21 (1H, dd, *J* = 8.3, 3.3 Hz, H-8), 3.42 (1H, t, *J* = 3.3 Hz, H-8′), 3.65 (6H, s, CH_3_O-11′, CH_3_O-13′), 3.89 (1H, d, *J* = 8.3 Hz, H-7), 4.08 (1H, d, *J* = 3.3 Hz, H-7′), 5.46 (1H, d, *J* = 2.1 Hz, H-14), 6.08 (1H, d, *J* = 2.1 Hz, H-12), 6.11 (2H, d, *J* = 2.3 Hz, H-10′, H-14′), 6.30 (1H, t, *J* = 2.3 Hz, H-12′), 6.61 (2H, d, *J* = 8.5 Hz, H-3′, H-5′), 6.66 (2H, d, *J* = 8.5 Hz, H-3, H-5), 6.74 (2H, d, *J* = 8.5 Hz, H-2′, H-6′), 6.91 (2H, d, *J* = 8.5 Hz, H-2, H-6), 8.77 (1H, s, 11OH), 8.82 (1H, s, 13OH), 9.04 (1H, s, 4′OH), 9.30 (1H, s, 4OH); ^13^C NMR (DMSO, 151 MHz) δ 19.3 (CH_3_-7d), 19.4 (CH_3_-7c), 27.9 (C-7b), 54.2 (C-7′), 54.9 (CH_3_O-11′, CH_3_O-13′), 57.5 (C-8′), 60.3 (C-8), 74.7 (C-7a), 84.0 (C-7), 97.0 (C-12′), 101.5 (C-12), 103.9 (C-14), 104.9 (C-10′, C-14′), 114.6 (C-3′, C-5′), 114.7 (C-3, C-5), 121.5 (C-10), 128.1 (C-2′, C-6′), 128.9 (C-2, C-6), 130.6 (C-1), 136.4 (C-1′), 145.3 (C-9), 150.1 (C-9′), 154.0 (C-11), 155.1 (C-4′), 156.7 (C-4), 157.3 (C-13), 160.3 (C-11′, C-13′). HR-ESI/MS analysis: *m/z* 555.2390 [M-H]^−^, (calcd for C_34_H_35_O_7_, 555.2383, ∆ = 1.3 ppm). MS/MS spectrum: CCMSLIB00009918900.

*Threo*-7′-*O*-isobutylisoresveptero acyclic dimer (**46**) UV (MeCN) λ_max_ (log ε) 229 (sh) (4.36), 285 (4.15), 307 (4.33), 326 (4.30), 341 (sh) (4.08) nm. ^1^H NMR (DMSO, 600 MHz) δ 0.79 (3H, d, *J* = 6.6 Hz, CH_3_-7′d), 0.80 (3H, d, *J* = 6.6 Hz, CH_3_-7′c), 1.74 (1H, hept, *J* = 6.6 Hz, H-7′b), 3.06 (2H, d, *J* = 6.6 Hz, H-7′a), 3.60 (6H, s, CH_3_O-11′, CH_3_O-13′), 4.50 (1H, d, *J* = 6.5 Hz, H-7′), 5.30 (1H, d, *J* = 6.5 Hz, H-8′), 6.10 (1H, t, *J* = 2.1 Hz, H-12), 6.25 (1H, t, *J* = 2.3 Hz, H-12′), 6.32 (2H, d, *J* = 2.3 Hz, H-10′, H-14′), 6.36 (2H, d, *J* = 2.1 Hz, H-10, H-14), 6.61 (2H, d, *J* = 8.7 Hz, H-3′, H-5′), 6.82 (1H, d, *J* = 16.3 Hz, H-8), 6.87 (2H, d, *J* = 8.8 Hz, H-3, H-5), 6.90 (1H, d, *J* = 16.3 Hz, H-7), 7.00 (2H, d, *J* = 8.4 Hz, H-2′, H- 6′), 7.39 (2H, d, *J* = 8.7 Hz, H-2, H-6), 9.18 (2H, s, 11OH, 13OH), 9.28 (1H, s, 4′OH); ^13^C NMR (DMSO, 151 MHz) δ 19.2 (CH_3_-7′d), 19.2 (CH_3_-7′c), 28.1 (C-7′b), 55.0 (CH_3_O-11′, CH_3_O-13′), 75.3 (C-7′a), 82.7 (C-8′), 84.4 (C-7′), 99.1 (C-12′), 101.9 (C-12), 104.4 (C-10, C-14), 105.6 (C-10′, C-14′), 114.5 (C-3′, C-5′), 115.9 (C-3, C-5), 126.7 (C-8), 127.4 (C-7), 127.5 (C-2, C-6), 128.6 (C-1′), 129.0 (C-2′, C-6′), 129.6 (C-1), 139.0 (C-9), 140.6 (C-9′), 156.7 (C-4′), 157.7 (C-4), 158.5 (C-11, C-13), 159.8 (C-11′, C-13′). HR-ESI/MS analysis: *m/z* 555.2402 [M-H]^−^, (calcd for C_34_H_35_O_7_, 555.2383, ∆ = 3.4 ppm). MS/MS spectrum: CCMSLIB00009918901.

*Erythro*-7′-*O*-isobutylisoresveptero acyclic dimer (**47**) UV (MeCN) λ_max_ (log ε) 229 (sh) (4.41), 285 (4.16), 307 (4.32), 327 (4.27), 342 (sh) (4.01) nm. ^1^H NMR (DMSO, 600 MHz) δ 0.69 (3H, d, *J* = 6.6 Hz, CH_3_-7′d), 0.70 (3H, d, *J* = 6.6 Hz, CH_3_-7′c), 1.64 (1H, hept, *J* = 6.6 Hz, CH_3_-7′b), 2.88 (1H, dd, *J* = 9.0, 6.3 Hz, CH_3_-7′a″), 3.01 (1H, dd, *J* = 9.1, 6.4 Hz, CH_3_-7′a′), 3.67 (6H, s, CH_3_O-11′, CH_3_O-13′), 4.42 (1H, d, *J* = 6.5 Hz, H-7′), 5.27 (1H, d, *J* = 6.5 Hz, H-8′), 6.10 (1H, t, *J* = 2.2 Hz, H-12), 6.34 (2H, d, *J* = 2.2 Hz, H-10, H-14), 6.35 (1H, t, *J* = 2.3 Hz, H-12′), 6.50 (2H, d, *J* = 2.3 Hz, H-10′, H-14′), 6.70 (2H, d, *J* = 8.5 Hz, H-3′, H-5′), 6.77 (2H, d, *J* = 8.9 Hz, H-3, H-5), 6.80 (1H, d, *J* = 16.2 Hz, H-8), 6.87 (1H, d, *J* = 16.2 Hz, H-7), 7.15 (2H, d, *J* = 8.6 Hz, H-2′, H-6′), 7.34 (2H, d, *J* = 8.9 Hz, H-2, H-6), 9.17 (2H, s, 11OH, 13OH), 9.32 (1H, s, 4′OH); ^13^C NMR (DMSO, 151 MHz) δ 18.9 (CH_3_-7′d, CH_3_-7′c), 27.7 (C-7′b), 74.9 (C-7′a), 81.6 (C-8′), 83.5 (C-7′), 99.0 (C-12′), 101.6 (C-12), 104.1 (C-10, C-14), 105.6 (C-10′, C-14′), 114.3 (C-3′, C-5′), 115.8 (C-3, C-5), 126.7 (C-8), 127.1 (C-7), 127.3 (C-2, C-6), 128.9 (C-2′, C-6′), 129.0 (C-1′), 129.5 (C-1), 138.7 (C-9), 156.7 (C-4′), 156.9 (C-4), 158.3 (C-11, C-13), 159.6 (C-11′, C-13′). HR-ESI/MS analysis: *m/z* 555.2402 [M-H]^−^, (calcd for C_34_H_35_O_7_, 555.2383, ∆ = 3.4 ppm). MS/MS spectrum: CCMSLIB00009918902.

*Erythro*-7′-*O*-isobutylresveptero acyclic dimer (**48**) UV (MeCN) λ_max_ (log ε) 229 (sh) (4.63), 286 (4.36), 307 (4.51), 326 (4.51), 343 (sh) (4.19) nm. ^1^H NMR (DMSO, 600 MHz) δ 0.69 (4H, d, *J* = 6.6 Hz, CH_3_-7′d), 0.70 (4H, d, *J* = 6.6 Hz, CH_3_-7′c), 1.63 (1H, m, H-7′b), 2.86 (1H, dd, *J* = 9.1, 6.4 Hz, H-7′a″), 2.99 (1H, dd, *J* = 9.1, 6.5 Hz, H-7′a′), 3.75 (6H, s, CH_3_O-11, CH_3_O-13), 4.32 (1H, d, *J* = 6.7 Hz, H-7′), 5.10 (1H, d, *J* = 6.7 Hz, H-8′), 6.06 (1H, t, *J* = 2.2 Hz, H-12′), 6.23 (2H, d, *J* = 2.2 Hz, H-10′, H-14′), 6.36 (1H, t, *J* = 2.3 Hz, H-12), 6.68 (2H, d, *J* = 2. Hz, H-10, H-14), 6.69 (2H, d, *J* = 8.6 Hz, H-3′, H-5′), 6.74 (2H, d, *J* = 8.8 Hz, H-3, H-5), 6.92 (1H, d, *J* = 16.4 Hz, H-8), 7.10 (1H, d, *J* = 16.4 Hz, H-7), 7.14 (2H, d, *J* = 8.6 Hz, H-2′, H-6′), 7.36 (2H, d, *J* = 8.8 Hz, H-2, H-6), 9.08 (2H, s, 11′OH, 13′OH), 9.30 (1H, s, 4′OH); ^13^C NMR (DMSO, 151 MHz) δ 19.0 (CH_3_-7′d, CH_3_-7′c), 27.7 (C-7′b), 54.8 (CH_3_O-11, CH_3_O-13), 74.9 (C-7′a), 81.6 (C-8′), 83.9 (C-7′), 99.3 (C-12), 101.6 (C-12′), 103.9 (C-10, C-14), 105.5 (C-10′, C-14′), 114.3 (C-3′, C-5′), 115.7 (C-3, C-5), 125.9 (C-8), 127.3 (C-2, C-6), 128.1 (C-7), 128.9 (C-2′, C-6′), 129.3 (C-1, C-1′), 139.1 (C-9), 140.9 (C-9′), 156.4 (C-4′), 157.1 (C-4), 157.6 (C-11′, C-13′), 160.5 (C-11, C-13). HR-ESI/MS analysis: *m/z* 555.2398 [M-H]^−^, (calcd for C_34_H_35_O_7_, 555.2383, ∆ = 2.7 ppm). MS/MS spectrum: CCMSLIB00009918903.

7-*O*-Isobutyl-11,11′,13,13′-tetra-*O*-methylleachianol G (**49**) UV (MeCN) λ_max_ (log ε) 229 (sh) (4.30), 285 (3.70), 321 (3.56) nm. ^1^H NMR (DMSO, 600 MHz) δ 0.72 (3H, d, *J* = 6.3 Hz, CH_3_-7d), 0.73 (3H, d, *J* = 6.3 Hz, CH_3_-7c), 1.58 (1H, m, *J* = 6.6 Hz, H-7b), 2.63 (1H, dd, *J* = 9.1, 6.6 Hz, H-7a″), 2.87 (1H, dd, *J* = 9.1, 6.6 Hz, H-7a′), 3.26 (1H, dd, *J* = 8.4, 3.0 Hz, H-8), 3.43 (1H, t, *J* = 3.0 Hz, H-8′), 3.51 (3H, s, CH_3_O-13), 3.55 (3H, s, CH_3_O-11), 3.66 (6H, s, CH_3_O-13′, CH_3_O-11′), 3.94 (1H, d, *J* = 8.4 Hz, H-7), 4.13 (1H, d, *J* = 3.0 Hz, H-7′), 5.57 (1H, d, *J* = 2.1 Hz, H-14), 6.10 (2H, d, *J* = 2.3 Hz, H-10′, H-14′), 6.31 (1H, t, *J* = 2.3 Hz, H-12′), 6.35 (1H, d, *J* = 2.1 Hz, H-12), 6.62 (2H, d, *J* = 8.5 Hz, H-3′, H-5′), 6.69 (3H, d, *J* = 8.4 Hz, H-3, H-5), 6.71 (3H, d, *J* = 8.5 Hz, H-2′, H-6′), 6.92 (2H, d, *J* = 8.4 Hz, H-2, H-6), 9.09 (1H, s, 4′OH), 9.35 (1H, s, 4OH); ^13^C NMR (DMSO, 151 MHz) δ 19.2 (CH_3_-7d), 19.3 (CH_3_-7c), 27.9 (C-7b), 54.5 (C-7′), 54.7 (CH_3_O-13), 54.9 (CH_3_O-11′, CH_3_O-13′), 55.0 (CH_3_O-11), 57.7 (C-8′), 60.6 (C-8), 74.7 (C-7a), 84.1 (C-7), 97.1 (C-12′), 97.6 (C-12), 102.0 (C-14), 104.8 (C-10′, C-14′), 114.7 (C-3, C-5), 114.8 (C-3′, C-5′), 124.4 (C-10), 127.9 (C-2′, C-6′), 129.0 (C-2, C-6), 130.4 (C-1), 136.0 (C-1′), 145.1 (C-9), 149.8 (C-9′), 155.2 (C-4′), 156.3 (C-11), 156.9 (C-4), 159.8 (C-13), 160.4 (C-11′, C-13′). HR-ESI/MS analysis: *m/z* 583.2704 [M-H]^−^, (calcd for C_36_H_39_O_7_, 583.2696, ∆ = 1.4 ppm). MS/MS spectrum: CCMSLIB00009918904.

*Threo*-7′-*O*-isobutylresveptero acyclic dimer (**50**) UV (MeCN) λ_max_ (log ε) 228 (sh) (4.35), 286 (4.13), 307 (4.28), 326 (4.27), 343 (sh) (3.96) nm. ^1^H NMR (DMSO, 600 MHz) δ 0.78 (3H, d, *J* = 6.7 Hz, CH_3_-7′c), 0.79 (3H, d, *J* = 6.7 Hz, CH_3_-7′d), 1.73 (1H, hept, *J* = 6.6 Hz, H-7′b), 3.03 (2H, d, *J* = 6.6 Hz, H-7′a), 3.76 (6H, s, CH_3_O-11, CH_3_O-13), 4.41 (1H, d, *J* = 6.6 Hz, H-7′), 5.14 (1H, d, *J* = 6.6 Hz, H-8′), 5.97 (1H, t, *J* = 2.2 Hz, H-12′), 6.07 (2H, d, *J* = 2.2 Hz, H-10′, H-14′), 6.36 (1H, t, *J* = 2.2 Hz, H-12), 6.61 (2H, d, *J* = 8.5 Hz, H-3′, H-5′), 6.70 (2H, d, *J* = 2.2 Hz, H-10, H-14), 6.85 (2H, d, *J* = 8.8 Hz, H-3, H-5), 6.95 (1H, d, *J* = 16.4 Hz, H-8), 7.00 (2H, d, *J* = 8.5 Hz, H-2′, H-6′), 7.13 (1H, d, *J* = 16.4 Hz, H-7), 7.41 (2H, d, *J* = 8.8 Hz, H-2, H-6), 9.02 (2H, s, 11′OH, 13′OH), 9.26 (1H, s, 4′OH); ^13^C NMR (DMSO, 151 MHz) δ 19.2 (CH_3_-7′d), 19.3 (CH_3_-7′c), 28.0 (C-7′b), 55.2 (CH_3_O-11, CH_3_O-13), 75.4 (C-7'a), 82.8 (C-8′), 84.6 (C-7′), 99.5 (C-12), 101.7 (C-12′), 104.1 (C-10, C-14), 105.6 (C-10′, C-14′), 114.6 (C-3′, C-5′), 115.8 (C-3, C-5), 126.1 (C-8), 127.6 (C-2, C-6), 128.5 (C-7), 128.7 (C-1′), 128.9 (C-2′, C-6′), 129.3 (C-1), 139.4 (C-9), 140.2 (C-9′), 156.6 (C-4′), 157.7 (C-11′, C-13′), 158.0 (C-4), 160.6 (C-11, C-13). HR-ESI/MS analysis: *m/z* 555.2395 [M-H]^−^, (calcd for C_34_H_35_O_7_, 555.2383, ∆ = 2.2 ppm). MS/MS spectrum: CCMSLIB00009918905.

7-*O*-Isobutyl-11,11′,13,13′-tetra-*O*-methylleachianol F (**51**) UV (MeCN) λ_max_ (log ε) 228 (sh) (4.43), 285 (3.75), 321 (3.56) nm. ^1^H NMR (DMSO, 600 MHz) δ 0.78 (3H, d, *J* = 6.7 Hz, CH_3_-7c), 0.80 (3H, d, *J* = 6.7 Hz, CH_3_-7d), 1.70 (1H, m, H-7b), 2.76 (1H, t, *J* = 3.7 Hz, H-8′), 2.82 (1H, dd, *J* = 9.0, 6.2 Hz, H-7a″), 2.87 (1H, dd, *J* = 9.0, 6.5 Hz, H-7a′), 3.23 (1H, dd, *J* = 9.2, 3.7 Hz, H-8), 3.57 (6H, s, CH_3_O-13′, CH_3_O-11′), 3.58 (3H, s, CH_3_O-11), 3.78 (3H, s, CH_3_O-13), 3.96 (1H, d, *J* = 9.2 Hz, H-7), 4.10 (1H, d, *J* = 3.7 Hz, H-7′), 5.70 (2H, d, *J* = 2.3 Hz, H-10′, H-14′), 6.20 (1H, t, *J* = 2.3 Hz, H-12′), 6.45 (1H, d, *J* = 2.1 Hz, H-12), 6.60 (2H, d, *J* = 8.4 Hz, H-3, H-5), 6.62 (2H, d, *J* = 8.7 Hz, H-3′, H-5′), 6.65 (2H, d, *J* = 8.7 Hz, H-2′, H-6′), 6.68 (2H, d, *J* = 8.4 Hz, H-2, H-6), 6.75 (1H, d, *J* = 2.1 Hz, H-14), 9.14 (1H, s, 4′OH), 9.31 (1H, s, 4OH); ^13^C NMR (DMSO, 151 MHz) δ 19.1 (CH_3_-7c, CH_3_-7d), 27.9 (C-7b), 53.9 (C-7′), 54.6 (CH_3_O-11, CH_3_O-11′, CH_3_O-13′), 54.8 (CH_3_O-13), 57.3 (C-8′), 59.7 (C-8), 74.3 (C-7a), 84.4 (C-7), 97.0 (C-12′), 97.6 (C-12), 102.7 (C-14), 104.2 (C-10′, C-14′), 114.4 (C-3, C-5), 114.6 (C-3′, C-5′), 123.6 (C-10), 127.6 (C-2′, C-6′), 128.2 (C-2, C-6), 130.3 (C-1), 135.5 (C-1′), 146.7 (C-9), 148.8 (C-9′), 155.1 (C-4′), 156.0 (C-11), 156.4 (C-4), 159.8 (C-11′, C-13′), 160.0 (C-13). HR-ESI/MS analysis: *m/z* 583.2704 [M-H]^−^, (calcd for C_36_H_39_O_7_, 583.2696, ∆ = 1.4 ppm). MS/MS spectrum: CCMSLIB00009918906.

*Erythro*-7′-*O*-isobutylpterostilbene acyclic dimer (**52**) UV (MeCN) λ_max_ (log ε) 227 (sh) (4.32), 285 (4.04), 307 (4.22), 324 (4.23), 344 (sh) (3.82) nm. ^1^H NMR (DMSO, 600 MHz) δ 0.69 (3H, d, *J* = 6.7 Hz, CH_3_-7′d), 0.70 (3H, d, *J* = 6.6 Hz, CH_3_-7′c), 1.64 (1H, m, H-7'b), 2.88 (1H, dd, *J* = 9.1, 6.4 Hz, H-7′a″), 3.01 (1H, dd, *J* = 9.1, 6.4 Hz, H-7′a′), 3.67 (6H, s, CH_3_O-11′, CH_3_O-13′), 3.75 (6H, s, CH_3_O-11, CH_3_O-13), 4.42 (1H, d, *J* = 6.5 Hz, H-7′), 5.29 (1H, d, *J* = 6.5 Hz, H-8′), 6.36 (1H, t, *J* = 2.3 Hz, H-12′), 6.36 (1H, t, *J* = 2.3 Hz, H-12), 6.50 (2H, d, *J* = 2.3 Hz, H-10′, H-14′), 6.68 (2H, d, *J* = 2.3 Hz, H-10, H-14), 6.70 (2H, d, *J* = 8.6 Hz, H-3′, H-5′), 6.80 (2H, d, *J* = 8.8 Hz, H-3, H-5), 6.93 (1H, d, *J* = 16.3 Hz, H-8), 7.10 (1H, d, *J* = 16.3 Hz, H-7), 7.15 (2H, d, *J* = 8.6 Hz, H-2′, H-6′), 7.36 (2H, d, *J* = 8.8 Hz, H-2, H-6), 9.32 (1H, s, 4′OH); ^13^C NMR (DMSO, 151 MHz) δ 19.1 (CH_3_-7′c, CH_3_-7′d), 55.0 (CH_3_O-11′, CH_3_O-13′), 55.2 (CH_3_O-11, CH_3_O-13), 75.1 (C-7′a), 81.7 (C-8′), 83.7 (C-7′), 99.1 (C-12′), 99.5 (C-12), 104.1 (C-10, C-14), 105.9 (C-10′, C-14′), 114.6 (C-3′, C-5′), 116.0 (C-3, C-5), 126.3 (C-8), 127.6 (C-2, C-6), 128.4 (C-7), 129.0 (C-2′, C-6′), 129.2 (C-1′), 129.7 (C-1), 139.3 (C-9), 141.3 (C-9′), 156.8 (C-4′), 157.2 (C-4), 159.8 (C-11′, C-13′), 160.6 (C-11, C-13). HR-ESI/MS analysis: *m/z* 583.2715 [M-H]^−^, (calcd for C_36_H_39_O_7_, 583.2696, ∆ = 3.3 ppm). MS/MS spectrum: CCMSLIB00009918907.

*Threo*-7′-*O*-isobutylpterostilbene acyclic dimer (**53**) UV (MeCN) λ_max_ (log ε) 227 (sh) (4.34), 286 (4.13), 307 (4.29), 327 (4.28), 342 (sh) (3.99) nm. ^1^H NMR (DMSO, 600 MHz) δ 0.79 (3H, d, *J* = 6.7 Hz, CH_3_-7′d), 0.80 (3H, d, *J* = 6.7 Hz, CH_3_-7′c), 1.74 (1H, m, *J* = 6.6 Hz, H-7′b), 3.05 (2H, d, *J* = 6.5 Hz, H-7′a), 3.60 (6H, s, CH_3_O-11′, CH_3_O-13′), 3.75 (6H, s, CH_3_O-11, CH_3_O-13), 4.50 (1H, d, *J* = 6.5 Hz, H-7′), 5.32 (1H, d, *J* = 6.5 Hz, H-8′), 6.25 (1H, t, *J* = 2.3 Hz, H-12′), 6.33 (2H, d, *J* = 2.3 Hz, H-10′, H-14′), 6.37 (1H, t, *J* = 2.2 Hz, H-12), 6.61 (2H, d, *J* = 8.6 Hz, H-3′, H-5′), 6.70 (2H, d, *J* = 2.2 Hz, H-10, H-14), 6.89 (2H, d, *J* = 8.9 Hz, H-3, H-5), 6.96 (1H, d, *J* = 16.3 Hz, H-8), 7.01 (2H, d, *J* = 8.6 Hz, H-2′, H-6′), 7.13 (1H, d, *J* = 16.3 Hz, H-7), 7.41 (2H, d, *J* = 8.9 Hz, H-2, H-6), 9.27 (1H, s, 4′OH); ^13^C NMR (DMSO, 151 MHz) δ 19.2 (CH_3_-7′c, CH_3_-7′d), 28.1 (C-7′b), 55.0 (CH_3_O-11′, CH_3_O-13′), 55.2 (CH_3_O-11, CH_3_O-13), 75.3 (C-7′a), 82.6 (C-8′), 84.4 (C-7′), 99.1 (C-12′), 99.5 (C-12), 104.1 (C-10, C-14), 105.6 (C-10′, C-14′), 114.5 (C-3′, C-5′), 116.0 (C-3, C-5), 126.2 (C-8), 127.6 (C-2, C-6), 128.5 (C-7), 128.6 (C-1′), 129.0 (C-2′, C-6′), 129.5 (C-1), 139.4 (C-9), 140.6 (C-9′), 156.7 (C-4′), 157.9 (C-4), 159.8 (C-11′, C-13′), 160.6 (C-11, C-13). HR-ESI/MS analysis: *m/z* 583.2715 [M-H]^−^, (calcd for C_36_H_39_O_7_, 583.2696, ∆ = 3.3 ppm). MS/MS spectrum: CCMSLIB00009918908.

Resvepterodimer A (**54**) UV (MeCN) λ_max_ (log ε) 228 (sh) (4.14), 282 (3.52) nm. ^1^H NMR (DMSO, 600 MHz) δ 2.84 (3H, s, CH_3_O-7′a), 3.00 (1H, dd, *J* = 9.5, 2.5 Hz, H-8′), 3.36 (3H, s, CH_3_O-7′b), 3.57 (6H, s, CH_3_O-11′, CH_3_O-13′), 3.74 (1H, d, *J* = 12.7 Hz, H-7), 4.06 (2H, dd, *J* = 12.7, 2.5 Hz, H-8), 4.43 (1H, d, *J* = 9.5 Hz, H-7′), 5.38 (2H,br s, H-10, H-14), 5.60 (2H, brs, H-10′, H-14′), 5.88 (1H, t, *J* = 2.2 Hz, H-12), 6.28 (1H, t, *J* = 2.3 Hz, H-12′), 6.37 (2H, d, *J* = 8.5 Hz, H-3, H-5), 6.74 (2H, d, *J* = 8.4 Hz, H-3′, H-5′), 6.88 (2H, d, *J* = 8.5 Hz, H-2, H-6), 7.20 (2H, d, *J* = 8.4 Hz, H-2′, H-6′). HR-ESI/MS analysis: *m/z* 545.2160 [M-H]^−^, (calcd for C_32_H_33_O_8_, 545.2175, ∆ = 2.8 ppm). MS/MS spectrum: CCMSLIB00009918909.

*Erythro*-7′-*O*-methylresveratrol acyclic dimer (**55**) UV (MeCN) λ_max_ (log ε) 228 (sh) (4.46), 286 (4.13), 324 (4.19), 341 (sh) (3.96) nm. ^1^H NMR (DMSO, 600 MHz) δ 3.03 (3H, s, CH_3_O-7′), 4.29 (1H, d, *J* = 6.2 Hz, H-7′), 5.15 (1H, d, *J* = 6.2 Hz, H-8′), 6.06 (1H, t, *J* = 2.2 Hz, H-12′), 6.09 (1H, t, *J* = 2.2 Hz, H-12), 6.18 (2H, d, *J* = 2.2 Hz, H-10′, H-14′), 6.34 (2H, d, *J* = 2.2 Hz, H-10, H-14), 6.69 (2H, d, *J* = 8.6 Hz, H-3′, H-5′), 6.72 (2H, d, *J* = 8.8 Hz, H-3, H-5), 6.79 (1H, d, *J* = 16.3 Hz, H-8), 6.87 (1H, d, *J* = 16.3 Hz, H-7), 7.12 (2H, d, *J* = 8.6 Hz, H-2′, H-6′), 7.34 (2H, d, *J* = 8.8 Hz, H-2, H-6), 9.11 (2H, s, 11′OH, 13′OH), 9.18 (2H, s, 11OH, 13OH), 9.33 (1H, s, 4′OH); ^13^C NMR (DMSO, 151 MHz) δ 56.1 (CH_3_O-7′), 81.6 (C-8′), 85.6 (C-7′), 101.9 (C-12, C-12′), 104.2 (C-10, C-14), 105.7 (C-10′, C-14′), 114.6 (C-3′, C-5′), 115.9 (C-3, C-5), 126.8 (C-8), 127.3 (C-7), 127.5 (C-2, C-6), 128.4 (C-1′), 129.2 (C-2′, C-6′), 129.6 (C-1), 138.8 (C-9), 156.8 (C-4′), 157.0 (C-4), 157.7 (C-11′, C-13′), 158.3 (C-11, C-13). HR-ESI/MS analysis: *m/z* 485.1593 [M-H]^−^, (calcd for C_29_H_25_O_7_, 485.1600, ∆ = 1.4 ppm). MS/MS spectrum: CCMSLIB00009918910.

*Threo*-7′-*O*-methylresveratrol acyclic dimer (**56**) UV (MeCN) λ_max_ (log ε) 228 (sh) (4.32), 286 (4.05), 308 (4.14), 325 (4.17), 342 (sh) (3.92) nm. ^1^H NMR (DMSO, 600 MHz) δ 3.12 (3H, s, CH_3_O-7′), 4.35 (1H, d, *J* = 6.6 Hz, H-7′), 5.16 (1H, d, *J* = 6.6 Hz, H-8′), 5.96 (1H, t, *J* = 2.2 Hz, H-12′), 6.04 (2H, d, *J* = 2.2 Hz, H-10′, H-14′), 6.10 (1H, t, *J* = 2.3 Hz, H-12), 6.36 (2H, d, *J* = 2.3 Hz, H-10, H-14), 6.62 (2H, d, *J* = 8.5 Hz, H-3′, H-5′), 6.81 (1H, d, *J* = 16.3 Hz, H-8), 6.83 (2H, d, *J* = 8.7 Hz, H-3, H-5), 6.89 (1H, d, *J* = 16.3 Hz, H-7), 6.99 (2H, d, *J* = 8.5 Hz, H-2′, H-6′), 7.38 (2H, d, *J* = 8.7 Hz, H-2, H-6), 9.03 (2H, s, 11′OH, 13′OH), 9.18 (2H, s, 11OH, 13OH), 9.30 (1H, s, 4′OH); ^13^C NMR (DMSO, 151 MHz) δ 56.1 (CH_3_O-7′), 82.3 (C-8′), 85.8 (C-7′), 101.6 (C-12, C-12′), 104.0 (C-10, C-14), 105.5 (C-10′, C-14′), 114.5 (C-3′, C-5′), 115.7 (C-3, C-5), 126.5 (C-8), 127.2 (C-7), 127.3 (C-2, C-6), 127.8 (C-1′), 128.9 (C-2′, C-6′), 129.2 (C-1), 138.8 (C-9), 139.8 (C-9′), 156.4 (C-4′), 157.3 (C-4), 157.5 (C-11′, C-13′), 158.2 (C-11, C-13). HR-ESI/MS analysis: *m/z* 485.1593 [M-H]^−^, (calcd for C_29_H_25_O_7_, 485.1600, ∆ = 1.4 ppm). MS/MS spectrum: CCMSLIB00009918911.

7,11′,13′-Tri-*O*-methylleachianol F (**57**) UV (MeCN) λ_max_ (log ε) 228 (sh) (4.46), 285 (3.71) nm. ^1^H NMR (DMSO, 600 MHz) δ 2.67 (1H, t, *J* = 4.2, 3.4 Hz, H-8′), 2.96 (3H, s, CH_3_O-7), 3.15 (1H, dd, *J* = 9.5, 4.2 Hz, H-8), 3.57 (6H, s, CH_3_O-11′, CH_3_O-13′), 3.81 (1H, d, *J* = 9.5 Hz, H-7), 4.04 (1H, d, *J* = 3.4 Hz, H-7′), 5.71 (2H, d, *J* = 2.3 Hz, H-10′, H-14′), 6.17 (1H, d, *J* = 2.1 Hz, H-12), 6.20 (1H, t, *J* = 2.3 Hz, H-12′), 6.45 (1H, d, *J* = 2.1 Hz, H-14), 6.59 (3H, d, *J* = 8.6 Hz, H-3, H-5), 6.62 (2H, d, *J* = 8.6 Hz, H-3′, H-5′), 6.66 (2H, d, *J* = 8.6 Hz, H-2, H-6), 6.66 (2H, d, *J* = 8.6 Hz, H-2′, H-6′), 8.80 (1H, s, OH), 9.01 (1H, s, OH), 9.09 (1H, s, OH), 9.31 (1H, s, OH); ^13^C NMR (DMSO, 151 MHz) δ 54.2 (C-7′), 54.8 (CH_3_O-11′, CH_3_O-13′), 55.9 (CH_3_O-7), 57.8 (C-8′), 59.6 (C-8), 86.6 (C-7), 97.2 (C-12′), 101.5 (C-12), 104.4 (C-14), 104.5 (C-10′, C-14′), 114.7 (C-3′, C-5′), 114.7 (C-3, C-5), 120.9 (C-10), 128.1 (C-2′, C-6′), 128.7 (C-2, C-6), 130.3 (C-1), 136.0 (C-1′), 147.1 (C-9), 149.3 (C-9′), 154.0 (C-11), 155.2 (C-4′), 156.8 (C-4), 157.8 (C-13), 160.1 (C-11′, C-13′). HR-ESI/MS analysis: *m/z* 513.1899 [M-H]^−^, (calcd for C_31_H_29_O_7_, 513.1913, ∆ = 2.7 ppm). MS/MS spectrum: CCMSLIB00009918912.

7,11,13-Tri-*O*-methylleachianol F (**58**) UV (MeCN) λ_max_ (log ε) 228 (sh) (4.43), 284 (3.78), 322 (3.39) nm. ^1^H NMR (DMSO, 600 MHz) δ 2.67 (1H, t, *J* = 2.8 Hz, H-8′), 2.96 (3H, s, CH_3_O-7), 3.21 (1H, dd, *J* = 8.9, 2.8 Hz, H-8), 3.58 (3H, s, CH_3_O-11), 3.79 (3H, s, CH_3_O-13), 3.83 (1H, d, *J* = 8.9 Hz, H-7), 4.00 (1H, d, *J* = 2.8 Hz, H-7′), 5.59 (2H, d, *J* = 2.1 Hz, H-10′, H-14′), 5.93 (1H, t, *J* = 2.2 Hz, H-12′), 6.45 (1H, d, *J* = 2.1 Hz, H-12), 6.62 (9H, m, H-2, H-2′, H-3, H-3′, H-5, H-5′, H-6, H-6′, H-14), 8.95 (2H, s, 11′OH, 13′OH), 9.13 (1H, s, OH), 9.33 (1H, s, OH); ^13^C NMR (DMSO, 151 MHz) δ 54.8 (C-7′), 55.1 (CH_3_O-11), 55.2 (CH_3_O-13), 56.1 (CH_3_O-7), 57.3 (C-8′), 59.5 (C-8), 86.2 (C-7), 97.3 (C-12), 100.2 (C-12′), 103.0 (C-14), 104.2 (C-10′, C-14′), 114.8 (C-3, C-5, C-3′, C-5′), 123.9 (C-10), 127.8 (C-2′, C-6′), 128.4 (C-2, C-6), 130.1 (C-1), 135.8 (C-1′), 147.3 (C-9), 149.4 (C-9′), 155.2 (C-4′), 156.4 (C-11), 156.7 (C-4), 158.1 (C-11′, C-13′), 160.3 (C-13). HR-ESI/MS analysis: *m/z* 513.1901 [M-H]^−^, (calcd for C_31_H_29_O_7_, 513.1913, ∆ = 2.3 ppm). MS/MS spectrum: CCMSLIB00009918913.

7,11′,13′-Tri-*O*-methylleachianol G (**59**) UV (MeCN) λ_max_ (log ε) 227 (sh) (4.45), 285 (3.68) nm. ^1^H NMR (DMSO, 600 MHz) δ 2.86 (3H, s, CH_3_O-7), 3.30 (2H, m, H-8, H-8′), 3.66 (6H, s, CH_3_O-11′, CH_3_O-13′), 3.83 (1H, d, *J* = 7.2 Hz, H-7), 4.03 (1H, d, *J* = 3.3 Hz, H-7′), 5.57 (1H, d, *J* = 2.1 Hz, H-14), 6.07 (1H, d, *J* = 2.1 Hz, H-12), 6.12 (2H, d, *J* = 2.3 Hz, H-10′, H-14′), 6.29 (1H, t, *J* = 2.3 Hz, H-12′), 6.60 (2H, d, *J* = 8.5 Hz, H-3′, H-5′), 6.66 (2H, d, *J* = 8.5 Hz, H-3, H-5), 6.70 (2H, d, *J* = 8.5 Hz, H-2′, H-6′), 6.92 (2H, d, *J* = 8.5 Hz, H-2, H-6), 8.75 (1H, s, OH), 8.85 (1H, s, OH), 9.06 (1H, s, OH), 9.34 (1H, s, OH); ^13^C NMR (DMSO, 151 MHz) δ 54.7 (C-7′), 55.0 (CH_3_O-11′, CH_3_O-13′), 55.8 (CH_3_O-7), 57.7 (C-8′), 59.4 (C-8), 85.6 (C-7), 97.2 (C-12′), 101.5 (C-12), 103.8 (C-14), 105.0 (C-10′, C-14′), 114.6 (C-3′, C-5′), 114.7 (C-3, C-5), 121.4 (C-10), 128.1 (C-2′, C-6′), 129.1 (C-2, C-6), 129.8 (C-1), 136.3 (C-1′), 145.3 (C-9), 149.6 (C-9′), 154.1 (C-11), 155.1 (C-4′), 156.8 (C-4), 157.4 (C-13), 160.3 (C-11′, C-13′). HR-ESI/MS analysis: *m/z* 513.1897 [M-H]^−^, (calcd for C_31_H_29_O_7_, 513.1913, ∆ = 3.1 ppm). MS/MS spectrum: CCMSLIB00009918914.

*Threo*-7′-*O*-methylisoresveptero acyclic dimer (**60**) UV (MeCN) λ_max_ (log ε) 228 (sh) (4.37), 285 (4.09), 308 (4.21), 325 (4.19), 342 (sh) (3.91) nm. ^1^H NMR (DMSO, 600 MHz) δ 3.15 (3H, s, CH_3_O-7′), 3.59 (6H, s, CH_3_O-11′, CH_3_O-13′), 4.43 (1H, d, *J* = 6.9 Hz, H-7′), 5.33 (1H, d, *J* = 6.9 Hz, H-8′), 6.10 (1H, t, *J* = 2.1 Hz, H-12), 6.23 (1H, t, *J* = 2.3 Hz, H-12′), 6.29 (2H, d, *J* = 2.3 Hz, H-10′, H-14′), 6.36 (2H, d, *J* = 2.1 Hz, H-10, H-14), 6.62 (2H, d, *J* = 8.5 Hz, H-3′, H-5′), 6.82 (1H, d, *J* = 16.3 Hz, H-8), 6.88 (2H, d, *J* = 8.8 Hz, H-3, H-5), 6.89 (1H, d, *J* = 16.3 Hz, H-7), 7.00 (2H, d, *J* = 8.5 Hz, H-2′, H-6′), 7.38 (2H, d, *J* = 8.8 Hz, H-2, H-6), 9.18 (2H, s, 11OH, 13OH), 9.31 (1H, s, 4′OH); ^13^C NMR (DMSO, 151 MHz) δ 55.0 (CH_3_O-11′, CH_3_O-13′), 56.3 (CH_3_O-7′), 82.3 (C-8′), 85.9 (C-7′), 99.0 (C-12′), 101.9 (C-12), 104.4 (C-10, C-14), 105.7 (C-10′, C-14′), 114.6 (C-3′, C-5′), 116.0 (C-3, C-5), 126.7 (C-8), 127.4 (C-7), 127.5 (C-2, C-6), 127.9 (C-1′), 129.1 (C-2′, C-6′), 129.7 (C-1), 139.0 (C-9), 140.4 (C-9′), 156.8 (C-4′), 157.4 (C-4), 158.5 (C-11, C-13), 159.8 (C-11′, C-13′). HR-ESI/MS analysis: *m/z* 513.1905 [M-H]^−^, (calcd for C_31_H_29_O_7_, 513.1913, ∆ = 1.6 ppm). MS/MS spectrum: CCMSLIB00009918915.

*Erythro*-7′-*O*-methylisoresveptero acyclic dimer (**61**) UV (MeCN) λ_max_ (log ε) 228 (sh) (4.31), 285 (3.91), 308 (3.99), 324 (3.96), 342 (sh) (3.67) nm. ^1^H NMR (DMSO, 600 MHz) δ 3.05 (3H, s, CH_3_O-7′), 3.66 (6H, s, CH_3_O-11′, CH_3_O-13′), 4.37 (1H, d, *J* = 6.1 Hz, H-7′), 5.34 (1H, d, *J* = 6.1 Hz, H-8′), 6.10 (1H, t, *J* = 2.2 Hz, H-12), 6.34 (2H, d, *J* = 2.2 Hz, H-10, H-14), 6.35 (1H, t, *J* = 2.3 Hz, H-12′), 6.44 (2H, d, *J* = 2.3 Hz, H-10′, H-14′), 6.70 (2H, d, *J* = 8.6 Hz, H-3′, H-5′), 6.77 (2H, d, *J* = 8.8 Hz, H-3, H-5), 6.80 (1H, d, *J* = 16.2 Hz, H-8), 6.87 (1H, d, *J* = 16.2 Hz, H-7), 7.13 (2H, d, *J* = 8.6 Hz, H-2′, H-6′), 7.34 (2H, d, *J* = 8.8 Hz, H-2, H-6), 9.18 (2H, s, 11OH, 13OH), 9.35 (1H, s, 4′OH); ^13^C NMR (DMSO, 151 MHz) δ 54.9 (CH_3_O-11′, CH_3_O-13′), 55.9 (CH_3_O-7′), 81.2 (C-8′), 85.1 (C-7′), 98.8 (C-12′), 101.6 (C-12), 104.1 (C-10, C-14), 105.7 (C-10′, C-14′), 114.5 (C-3′, C-5′), 115.7 (C-3, C-5), 126.7 (C-8), 127.1 (C-7), 127.3 (C-2, C-6), 128.3 (C-1′), 129.1 (C-2′, C-6′), 129.6 (C-1), 138.8 (C-9), 156.8 (C-4′), 157.0 (C-4), 158.4 (C-11, C-13), 159.8 (C-11′, C-13′). HR-ESI/MS analysis: *m/z* 513.1905 [M-H]^−^, (calcd for C_31_H_29_O_7_, 513.1913, ∆ = 1.6 ppm). MS/MS spectrum: CCMSLIB00009918916.

*Threo*-7′-*O*-methylresveptero acyclic dimer (**62**) UV (MeOH) λ_max_ (log ε) 226 (sh) (4.24), 285 (3.74), 305 (3.80), 322 (3.79) nm. ^1^H NMR (DMSO, 600 MHz) δ 3.13 (3H, s, CH_3_O-7′), 3.75 (6H, s, CH_3_O-11, CH_3_O-13), 4.35 (1H, d, *J* = 6.6 Hz, H-7′), 5.17 (1H, d, *J* = 6.6 Hz, H-8′), 5.96 (1H, t, *J* = 2.2 Hz, H-12′), 6.05 (2H, d, *J* = 2.2 Hz, H-10′, H-14′), 6.36 (1H, t, *J* = 2.3 Hz, H-12), 6.62 (2H, d, *J* = 8.5 Hz, H-3′, H-5′), 6.69 (2H, d, *J* = 2.3 Hz, H-10, H-14), 6.85 (2H, d, *J* = 8.8 Hz, H-3, H-5), 6.95 (1H, d, *J* = 16.4 Hz, H-8), 7.00 (2H, d, *J* = 8.5 Hz, H-2′, H-6′), 7.13 (1H, d, *J* = 16.4 Hz, H-7), 7.41 (2H, d, *J* = 8.8 Hz, H-2, H-6); ^13^C NMR (DMSO, 151 MHz) δ 55.2 (CH_3_O-11, CH_3_O-13), 56.4 (CH_3_O-7′), 82.4 (C-8′), 86.0 (C-7′), 99.5 (C-12), 101.8 (C-12′), 104.1 (C-10, C-14), 105.7 (C-10′, C-14′), 114.6 (C-3′, C-5′), 115.9 (C-3, C-5), 126.1 (C-8), 127.6 (C-2, C-6), 128.0 (C-1′), 128.5 (C-7), 129.1 (C-2′, C-6′), 129.4 (C-1), 139.4 (C-9), 140.1 (C-9′), 156.7 (C-4′), 157.7 (C-4, C-11′, C-13′), 160.6 (C-11, C-13). HR-ESI/MS analysis: *m/z* 513.1897 [M-H]^−^, (calcd for C_31_H_29_O_7_, 513.1913, ∆ = 3.1 ppm). MS/MS spectrum: CCMSLIB00009918917.

7,11,11′,13,13′-Penta-*O*-methylleachianol F (**63**) UV (MeCN) λ_max_ (log ε) 228 (sh) (4.55), 284 (3.79) nm. ^1^H NMR (DMSO, 600 MHz) δ 2.74 (1H, t, *J* = 3.9 Hz, H-8′), 3.00 (3H, s, CH_3_O-7), 3.26 (1H, dd, *J* = 9.0, 4.1 Hz, H-8), 3.56 (3H, s, CH_3_O-11), 3.58 (6H, s, CH_3_O-11′, CH_3_O-13′), 3.78 (3H, s, CH_3_O-13), 3.90 (1H, d, *J* = 8.9 Hz, H-7), 4.09 (1H, d, *J* = 3.7 Hz, H-7′), 5.72 (2H, d, *J* = 2.3 Hz, H-10′, H-14′), 6.21 (1H, t, *J* = 2.3 Hz, H-12′), 6.46 (1H, d, *J* = 2.1 Hz, H-12), 6.62 (6H, m, H-2′, H-3, H-3′, H-5, H-5′, H-6′), 6.69 (1H, d, *J* = 2.2 Hz, H-14), 6.70 (2H, m, H-2, H-6); ^13^C NMR (DMSO, 151 MHz) δ 54.6 (C-7′), 54.8 (CH_3_O-11′, CH_3_O-13′), 55.1 (CH_3_O-11), 55.2 (CH_3_O-13), 56.0 (CH_3_O-7), 57.8 (C-8′), 59.6 (C-8), 86.3 (C-7), 97.3 (C-12′), 97.5 (C-12), 102.9 (C-14), 104.5 (C-10′, C-14′), 114.8 (C-3′, C-5′), 114.8 (C-3, C-5), 123.9 (C-10), 127.9 (C-2′, C-6′), 128.7 (C-2, C-6), 130.0 (C-1), 135.7 (C-1′), 146.9 (C-9), 148.9 (C-9′), 155.3 (C-4′), 156.3 (C-13), 156.9 (C-4), 160.1 (C-11′, C-13′), 160.4 (C-11). HR-ESI/MS analysis: *m/z* 541.2210 [M-H]^−^, (calcd for C_33_H_33_O_7_, 541.2226, ∆ = 3.0 ppm). MS/MS spectrum: CCMSLIB00009918918.

*Threo*-7′-*O*-methylpterostilbene acyclic dimer (**64**) UV (MeCN) λ_max_ (log ε) 227 (sh) (4.51), 285 (4.28), 308 (4.44), 324 (4.43), 341 (sh) (4.13) nm. ^1^H NMR (DMSO, 600 MHz) δ 3.15 (3H, s, CH_3_O-7′), 3.59 (6H, s, CH_3_O-11′, CH_3_O-13′), 3.75 (6H, s, CH_3_O-11, CH_3_O-13), 4.44 (1H, d, *J* = 6.8 Hz, H-7′), 5.34 (1H, d, *J* = 6.8 Hz, H-8′), 6.24 (1H, t, *J* = 2.3 Hz, H-12′), 6.31 (2H, d, *J* = 2.3 Hz, H-10′, H-14′), 6.37 (1H, t, *J* = 2.3 Hz, H-12), 6.62 (2H, d, *J* = 8.5 Hz, H-3′, H-5′), 6.69 (2H, d, *J* = 2.3 Hz, H-10, H-14), 6.91 (2H, d, *J* = 8.8 Hz, H-3, H-5), 6.96 (1H, d, *J* = 16.3 Hz, H-8), 7.01 (2H, d, *J* = 8.5 Hz, H-2′, H-6′), 7.13 (1H, d, *J* = 16.3 Hz, H-7), 7.41 (2H, d, *J* = 8.8 Hz, H-2, H-6); ^13^C NMR (DMSO, 151 MHz) δ 54.6 (CH_3_O-11′, CH_3_O-13′), 55.0 (CH_3_O-11, CH_3_O-13), 56.0 (CH_3_O-7′), 82.1 (C-8′), 85.6 (C-7′), 98.6 (C-12′), 99.1 (C-12), 103.9 (C-10, C-14), 105.5 (C-10′, C-14′), 114.4 (C-3′, C-5′), 115.7 (C-3, C-5), 126.0 (C-8), 127.4 (C-2, C-6), 127.6 (C-1′), 128.3 (C-7), 128.8 (C-2′, C-6′), 129.2 (C-1), 139.1 (C-9), 156.6 (C-4′), 157.2 (C-4), 159.5 (C-11′, C-13′), 160.4 (C-11, C-13). HR-ESI/MS analysis: *m/z* 541.2218 [M-H]^−^, (calcd for C_33_H_33_O_7_, 541.2226, ∆ = 1.8 ppm). MS/MS spectrum: CCMSLIB00009918919.

*Threo*-7′-*O*-ethylresveratrol acyclic dimer (**65**) UV (MeCN) λ_max_ (log ε) 230 (sh) (4.40), 285 (4.16), 306 (4.27), 322 (4.27), 340 (sh) (4.00) nm. ^1^H NMR (DMSO, 600 MHz) δ 1.04 (3H, t, *J* = 7.0 Hz, CH_3_-7′b), 3.32 (2H, overlapped, H-7′a), 4.44 (1H, d, *J* = 6.6 Hz, H-7′), 5.14 (1H, d, *J* = 6.6 Hz, H-8′), 5.96 (1H, t, *J* = 2.2 Hz, H-12′), 6.05 (2H, d, *J* = 2.2 Hz, H-10′, H-14′), 6.10 (1H, t, *J* = 2.2 Hz, H-12), 6.36 (2H, d, *J* = 2.2 Hz, H-10, H-14), 6.61 (2H, d, *J* = 8.4 Hz, H-3′, H-5′), 6.81 (1H, d, *J* = 16.3 Hz, H-8), 6.83 (2H, d, *J* = 8.8 Hz, H-3, H-5), 6.89 (1H, d, *J* = 16.3 Hz, H-7), 7.00 (2H, d, *J* = 8.4 Hz, H-2′, H-6′), 7.38 (2H, d, *J* = 8.8 Hz, H-2, H-6), 9.04 (2H, s, 11′OH, 13′OH), 9.20 (2H, s, 11OH, 13OH), 9.28 (1H, s, 4′OH); ^13^C NMR (DMSO, 151 MHz) δ 15.3 (CH_3_-7′b), 63.9 (C-7'a), 82.7 (C-8′), 84.2 (C-7′), 101.8 (C-12′), 101.9 (C-12), 104.4 (C-10, C-14), 105.7 (C-10′, C-14′), 114.6 (C-3′, C-5′), 115.9 (C-3, C-5), 126.6 (C-8), 127.4 (C-7), 127.5 (C-2, C-6), 128.7 (C-1′), 128.9 (C-2′, C-6′), 129.5 (C-1), 139.1 (C-9), 140.2 (C-9′), 156.6 (C-4′), 157.7 (C-4, C-11′, C-13′), 158.5 (C-11, C-13). HR-ESI/MS analysis: *m/z* 499.1788 [M-H]^−^, (calcd for C_30_H_27_O_7_, 499.1757, ∆ = 6.2 ppm). MS/MS spectrum: CCMSLIB00009918920.

7-*O*-Ethyl-11,13-di-*O*-methylleachianol F (**66**) UV (MeCN) λ_max_ (log ε) 228 (sh) (4.42), 283 (3.79), 323 (3.48) nm. ^1^H NMR (DMSO, 600 MHz) δ 1.01 (3H, t, *J* = 6.9 Hz, CH_3_-7b), 2.67 (1H, t, *J* = 2.7 Hz, H-8′), 3.02 (1H, dq, *J* = 9.5, 7.0 Hz, H-7a″), 3.17 (2H, m, H-7a′, H-8), 3.58 (3H, s, CH_3_O-11), 3.78 (3H, s, CH_3_O-13), 3.90 (1H, d, *J* = 9.0 Hz, H-7), 4.01 (1H, d, *J* = 2.7 Hz, H-7′), 5.59 (2H, d, *J* = 2.2 Hz, H-10′, H-14′), 5.93 (1H, t, *J* = 2.2 Hz, H-12′), 6.45 (1H, d, *J* = 2.1 Hz, H-12), 6.60 (2H, d, *J* = 8.7 Hz, H-3, H-5), 6.63 (4H, m, H-2, H-3′, H-5′, H-6), 6.65 (1H, overlapped, H-14), 6.66 (2H, d, *J* = 8.8 Hz, H-2′, H-6′), 8.94 (2H, s, 11′OH, 13′OH), 9.14 (1H, s, 4′OH), 9.31 (1H, s, 4OH); ^13^C NMR (DMSO, 151 MHz) δ 15.1 (CH_3_-7b), 54.7 (C-7′), 55.1 (CH_3_O-11, CH_3_O-13), 57.3 (C-8′), 59.5 (C-8), 63.4 (C-7a), 84.2 (C-7), 97.5 (C-12), 100.2 (C-12′), 102.8 (C-14), 104.1 (C-10′, C-14′), 114.8 (C-3, C-5, C-3′, C-5′), 123.9 (C-10), 127.8 (C-2′, C-6′), 128.3 (C-2, C-6), 130.9 (C-1), 135.9 (C-1′), 147.4 (C-9), 149.4 (C-9′), 155.2 (C-4′), 156.3 (C-11), 156.6 (C-4), 158.1 (C-11′, C-13′), 160.2 (C-13). HR-ESI/MS analysis: *m/z* 527.2099 [M-H]^−^, (calcd for C_32_H_31_O_7_, 527.2070, ∆ = 5.5 ppm). MS/MS spectrum: CCMSLIB00009918921.

7-*O*-Ethyl-11′,13′-di-*O*-methylleachianol G (**67**) UV (MeCN) λ_max_ (log ε) 228 (sh) (4.43), 282 (3.71) nm. ^1^H NMR (DMSO, 600 MHz) δ 0.89 (3H, t, *J* = 7.0 Hz, CH_3_-7b), 2.90 (1H, dq, *J* = 9.6, 7.0 Hz, H-7a″), 3.12 (1H, dq, *J* = 9.7, 7.0 Hz, H-7a′), 3.23 (1H, dd, *J* = 8.0, 3.7 Hz, H-8), 3.33 (1H, overlapped, H-8′), 3.66 (6H, s, CH_3_O-13′, CH_3_O-11′), 3.96 (1H, d, *J* = 8.0 Hz, H-7), 4.06 (1H, d, *J* = 3.4 Hz, H-7′), 5.52 (1H, d, *J* = 2.1 Hz, H-14), 6.08 (1H, d, *J* = 2.1 Hz, H-12), 6.11 (2H, d, *J* = 2.3 Hz, H-10′, H-14′), 6.29 (1H, t, *J* = 2.3 Hz, H-12′), 6.60 (2H, d, *J* = 8.5 Hz, H-3′, H-5′), 6.67 (2H, d, *J* = 8.5 Hz, H-3, H-5), 6.73 (2H, d, *J* = 8.5 Hz, H-2′, H-6′), 6.93 (2H, d, *J* = 8.5 Hz, H-2, H-6), 8.75 (1H, s, 11OH), 8.83 (1H, s, 13OH), 9.05 (1H, s, 4′OH), 9.31 (1H, s, 4OH); ^13^C NMR (DMSO, 151 MHz) δ 14.9 (CH_3_-7b), 54.5 (C-7′), 54.9 (CH_3_O-13′, CH_3_O-11′), 57.7 (C-8′), 59.9 (C-8), 62.8 (C-7a), 83.3 (C-7), 97.1 (C-12′), 101.5 (C-12), 103.8 (C-14), 105.0 (C-10′, C-14′), 114.6 (C-3′, C-5′), 114.7 (C-3, C-5), 121.5 (C-10), 128.2 (C-2′, C-6′), 128.9 (C-2, C-6), 130.6 (C-1), 136.5 (C-1′), 145.3 (C-9), 150.0 (C-9′), 154.0 (C-11), 155.1 (C-4′), 156.7 (C-4), 157.4 (C-13), 160.3 (C-11′, C-13′). HR-ESI/MS analysis: *m/z* 527.2098 [M-H]^−^, (calcd for C_32_H_31_O_7_, 527.2070, ∆ = 5.3 ppm). MS/MS spectrum: CCMSLIB00009918922.

*Erythro*-7′-*O*-ethylresveptero acyclic dimer (**68**) UV (MeCN) λ_max_ (log ε) 228 (sh) (4.47), 285 (4.16), 306 (4.26), 325 (4.26), 340 (sh) (4.00) nm. ^1^H NMR (DMSO, 600 MHz) δ 0.96 (3H, t, *J* = 7.0 Hz, CH_3_-7′b), 3.18 (1H, m, H-7′a″), 3.27 (1H, m, H-7′a′), 3.75 (6H, s, CH_3_O-11, CH_3_O-13), 4.37 (1H, d, *J* = 6.1 Hz, H-7′), 5.15 (1H, d, *J* = 6.1 Hz, H-8′), 6.06 (1H, t, *J* = 2.2 Hz, H-12′), 6.18 (2H, d, *J* = 2.2 Hz, H-10′, H-14′), 6.36 (1H, t, *J* = 2.3 Hz, H-12), 6.68 (2H, d, *J* = 8.5 Hz, H-3′, H-5′), 6.68 (3H, d, *J* = 2.3 Hz, H-10, H-14), 6.74 (2H, d, *J* = 8.9 Hz, H-3, H-5), 6.93 (1H, d, *J* = 16.4 Hz, H-8), 7.11 (1H, d, *J* = 16.4 Hz, H-7), 7.12 (2H, d, *J* = 8.5 Hz, H-2′, H-6′), 7.37 (2H, d, *J* = 8.9 Hz, H-2, H-6), 9.11 (2H, s, 11′OH, 13′OH), 9.30 (1H, s, 4′OH); ^13^C NMR (DMSO, 151 MHz) δ 15.1 (CH_3_-7'b), 55.2 (CH_3_O-11, CH_3_O-13), 63.7 (C-7′a), 81.6 (C-8′), 83.7 (C7′), 99.5 (C-12), 101.8 (C-12′), 104.1 (C-10, C-14), 105.6 (C-10′, C-14′), 114.5 (C-3′, C-5′), 115.9 (C-3, C-5), 126.2 (C-8), 127.5 (C-2, C-6), 128.4 (C-7), 129.1 (C-2′, C-6′), 129.2 (C-1′), 129.5 (C-1), 139.4 (C-9), 140.9 (C-9′), 156.7 (C-4′), 157.3 (C-4), 157.8 (C-11′, C-13′), 160.6 (C-11, C-13). HR-ESI/MS analysis: *m/z* 527.2103 [M-H]^−^, (calcd for C_32_H_31_O_7_, 527.2070, ∆ = 6.3 ppm). MS/MS spectrum: CCMSLIB00009918923.

*Threo*-7′-*O*-ethylpterostilbene acyclic dimer (**69**) UV (MeCN) λ_max_ (log ε) 228 (sh) (4.51), 285 (4.29), 306 (4.43), 325 (4.42), 340 (sh) (4.14) nm. ^1^H NMR (DMSO, 600 MHz) δ 1.05 (3H, t, *J* = 7.0 Hz, CH_3_-7′b), 3.35 (2H, t, *J* = 7.0 Hz, H-7′a), 3.60 (6H, s, CH_3_O-11′, CH_3_O-13′), 3.75 (6H, s, CH_3_O-11, CH_3_O-13), 4.52 (1H, d, *J* = 6.7 Hz, H-7′), 5.32 (1H, d, *J* = 6.7 Hz, H-8′), 6.24 (1H, t, *J* = 2.3 Hz, H-12′), 6.33 (2H, d, *J* = 2.3 Hz, H-10′, H-14′), 6.37 (1H, t, *J* = 2.3 Hz, H-12), 6.61 (2H, d, *J* = 8.6 Hz, H-3′, H-5′), 6.70 (2H, d, *J* = 2.3 Hz, H-10, H-14), 6.90 (2H, d, *J* = 8.8 Hz, H-3, H-5), 6.96 (1H, d, *J* = 16.3 Hz, H-8), 7.01 (2H, d, *J* = 8.6 Hz, H-2′, H-6′), 7.13 (1H, d, *J* = 16.3 Hz, H-7), 7.41 (2H, d, *J* = 8.8 Hz, H-2, H-6), 9.29 (1H, s, 4′OH); ^13^C NMR (DMSO, 151 MHz) δ 15.3 (CH_3_-7′b), 55.0 (CH_3_O-11′, CH_3_O-13′), 55.2 (CH_3_O-11, CH_3_O-13), 63.9 (C-7′a), 82.5 (C-8′), 84.0 (C-7′), 99.0 (C-12′), 99.5 (C-12), 104.1 (C-10, C-14), 105.7 (C-10′, C-14′), 114.5 (C-3′, C-5′), 116.1 (C-3, C-5), 126.2 (C-8), 127.6 (C-2, C-6), 128.5 (C-7), 128.7 (C-1′), 128.9 (C-2′, C-6′), 129.6 (C-1), 139.4 (C-9), 140.6 (C-9′), 156.7 (C-4′), 157.7 (C-4), 159.8 (C-11′, C-13′), 160.6 (C-11, C-13). HR-ESI/MS analysis: *m/z* 555.2426 [M-H]^−^, (calcd for C_34_H_35_O_7_, 555.2383, ∆ = 7.7 ppm). MS/MS spectrum: CCMSLIB00009918924.

11′,13′-Di-*O*-methylrestrytisol B (**70**) UV (MeCN) λ_max_ (log ε) 228 (sh) (4.30), 282 (3.65) nm. ^1^H NMR (DMSO, 600 MHz) δ 3.38 (1H, overlapped, H-8), 3.54 (6H, s, CH_3_O-11′, CH_3_O-13′), 3.98 (1H, t, *J* = 9.0 Hz, H-8′), 4.90 (1H, d, *J* = 9.7 Hz, H-7), 5.41 (1H, d, *J* = 9.0 Hz, H-7′), 5.98 (1H, t, *J* = 2.2 Hz, H-12), 6.05 (1H, t, *J* = 2.3 Hz, H-12′), 6.07 (1H, d, *J* = 2.3 Hz, H-10′, H-14′), 6.08 (2H, d, *J* = 2.2 Hz, H-10, H-14), 6.51 (2H, d, *J* = 8.5 Hz, H-3′, H-5′), 6.73 (1H, d, *J* = 8.5 Hz, H-3, H-5), 6.95 (2H, d, *J* = 8.5 Hz, H-2′, H-6′), 7.22 (1H, d, *J* = 8.5 Hz, H-2, H-6), 9.07 (2H, s, 11OH, 13OH), 9.12 (1H, s, 4′OH), 9.40 (1H, s, 4OH); ^13^C NMR (DMSO, 151 MHz) δ 54.7 (CH_3_O-11′, CH_3_O-13′), 57.1 (C-8), 58.3 (C-8′), 82.5 (C-7′), 85.7 (C-7), 97.7 (C-12′), 100.9 (C-12), 106.1 (C-10, C-14), 107.1 (C-10′, C-14′), 114.1 (C-3′, C-5′), 114.9 (C-3, C-5), 127.8 (C-2′, C-6′), 128.0 (C-2, C-6), 129.9 (C-1), 130.9 (C-1′), 141.0 (C-9′), 141.7 (C-9), 155.7 (C-4′), 156.9 (C-4), 158.1 (C-11, C-13), 159.3 (C-11′, C-13′). HR-ESI/MS analysis: *m/z* 499.1789 [M-H]^−^, (calcd for C_30_H_27_O_7_, 499.1757, ∆ = 6.4 ppm). MS/MS spectrum: CCMSLIB00009918925.

7-*O*-Ethylleachianol G (**71**) UV (MeCN) λ_max_ (log ε) 228 (sh) (4.45), 282 (3.74) nm. ^1^H NMR (DMSO, 600 MHz) δ 0.86 (3H, t, *J* = 7.0 Hz, CH_3_-7b), 2.84 (1H, dq, *J* = 9.8, 6.9 Hz, H-7a″), 3.09 (1H, m, H-7a′), 3.15 (1H, dd, *J* = 8.5, 3.2 Hz, H-8), 3.25 (1H, t, *J* = 3.2 Hz, H-8′), 3.84 (1H, d, *J* = 8.5 Hz, H-7), 4.03 (1H, d, *J* = 3.2 Hz, H-7′), 5.38 (1H, d, *J* = 2.1 Hz, H-14), 5.92 (2H, d, *J* = 2.2 Hz, H-10′, H-14′), 5.98 (1H, t, *J* = 2.2 Hz, H-12′), 6.06 (1H, d, *J* = 2.1 Hz, H-12), 6.61 (2H, d, *J* = 8.3 Hz, H-3′, H-5′), 6.67 (2H, d, *J* = 8.2 Hz, H-3, H-5), 6.71 (2H, d, *J* = 8.1 Hz, H-2′, H-6′), 6.90 (2H, d, *J* = 8.1 Hz, H-2, H-6); ^13^C NMR (DMSO, 151 MHz) δ 14.6 (CH_3_-7b), 54.1 (C-7′), 57.5 (C-8′), 59.7 (C-8), 62.4 (C-7a), 83.4 (C-7), 99.8 (C-12′), 101.2 (C-12), 103.9 (C-14), 104.4 (C-10′, C-14′), 114.4 (C-3, C-5, C-3′, C-5′), 121.3 (C-10), 127.7 (C-2′, C-6′), 128.8 (C-2, C-6), 130.3 (C-1), 136.6 (C-1′), 145.1 (C-9), 149.8 (C-9′), 153.7 (C-11), 154.8 (C-4′), 156.4 (C-4), 156.8 (C-13), 157.9 (C-11′, C-13′). HR-ESI/MS analysis: *m/z* 499.1788 [M-H]^−^, (calcd for C_30_H_27_O_7_, 499.1757, ∆ = 6.3 ppm). MS/MS spectrum: CCMSLIB00009918926.

7-*O*-Ethyl-11′,13′-di-*O*-methylleachianol F (**72**) UV (MeCN) λ_max_ (log ε) 228 (sh) (4.40), 282 (3.70) nm. ^1^H NMR (DMSO, 600 MHz) δ 1.02 (3H, t, *J* = 7.0 Hz, CH_3_-7b), 2.68 (1H, t, *J* = 4.0, 3.4 Hz, H-8′), 3.07 (1H, dq, *J* = 9.5, 7.0 Hz, H-7a″), 3.13 (1H, dd, *J* = 9.1, 4.0 Hz, H-8), 3.16 (1H, m, H-7a′), 3.57 (6H, s, CH_3_O-11′, CH_3_O-13′), 3.92 (1H, d, *J* = 9.1 Hz, H-7), 4.05 (1H, d, *J* = 3.4 Hz, H-7′), 5.72 (2H, d, *J* = 2.2 Hz, H-10′, H-14′), 6.16 (1H, d, *J* = 2.2 Hz, H-12), 6.20 (1H, t, *J* = 2.3 Hz, H-12′), 6.48 (1H, d, *J* = 2.1 Hz, H-14), 6.57 (2H, d, *J* = 8.5 Hz, H-3, H-5), 6.62 (2H, d, *J* = 8.7 Hz, H-3′, H-5′), 6.66 (2H, d, *J* = 8.5 Hz, H-2, H-6), 6.67 (2H, d, *J* = 8.7 Hz, H-2′ H-6′); ^13^C NMR (DMSO, 151 MHz) δ 15.1 (CH_3_-7b), 54.2 (C-7′), 54.8 (CH_3_O-11′, CH_3_O-13′), 57.8 (C-8′), 59.5 (C-8), 63.2 (C-7a), 84.5 (C-7), 97.2 (C-12′), 101.5 (C-12), 104.5 (C-14), 104.5 (C-10′, C-14′), 114.6 (C-3, C-3′, C-5, C-5′), 120.9 (C-10), 128.1 (C-2′, C-6′), 128.5 (C-2, C-6), 131.0 (C-1), 136.1 (C-1′), 147.2 (C-9), 149.3 (C-9′), 153.9 (C-11), 155.2 (C-4′), 156.7 (C-4), 157.8 (C-13), 160.1 (C-11′, C-13′). HR-ESI/MS analysis: *m/z* 527.2100 [M-H]^−^, (calcd for C_32_H_31_O_7_, 527.2070, ∆ = 5.7 ppm). MS/MS spectrum: CCMSLIB00009918927.

*Erythro*-7′-*O*-ethylresveratrol acyclic dimer (**73**) UV (MeCN) λ_max_ (log ε) 226 (sh) (4.15), 285 (3.88), 306 (3.94), 325 (3.92) nm. ^1^H NMR (DMSO, 600 MHz) δ 0.96 (3H, t, *J* = 7.0 Hz, CH_3_-7'b), 3.18 (1H, m, H-7′a″), 3.27 (1H, m, H-7′a′), 4.37 (1H, d, *J* = 6.2 Hz, H-7′), 5.14 (1H, d, *J* = 6.2 Hz, H-8′), 6.06 (1H, t, *J* = 2.2 Hz, H-12′), 6.10 (1H, t, *J* = 2.1 Hz, H-12), 6.18 (2H, d, *J* = 2.2 Hz, H-10′, H-14′), 6.34 (2H, d, *J* = 2.1 Hz, H-10, H-14), 6.68 (2H, d, *J* = 8.6 Hz, H-3′, H-5′), 6.72 (2H, d, *J* = 8.8 Hz, H-3, H-5), 6.79 (1H, d, *J* = 16.3 Hz, H-8), 6.87 (1H, d, *J* = 16.3 Hz, H-7), 7.12 (2H, d, *J* = 8.6 Hz, H-2′, H-6′), 7.34 (2H, d, *J* = 8.8 Hz, H-2, H-6); ^13^C NMR (DMSO, 151 MHz) δ 14.9 (CH_3_-7′b), 63.4 (C-7′a), 81.4 (C-8′), 83.4 (C7′), 101.6 (C-12, C-12′), 104.1 (C-10, C-14), 105.3 (C-10′, C-14′), 114.3 (C-3′, C-5′), 115.7 (C-3, C-5), 126.4 (C-8), 127.1 (C-7), 127.3 (C-2, C-6), 128.8 (C-2′, C-6′), 129.2 (C-1), 156.4 (C-4′), 156.9 (C-4), 157.5 (C-11′, C-13′), 158.2 (C-11, C-13). HR-ESI/MS analysis: *m/z* 499.1791[M-H]^−^, (calcd for C_30_H_27_O_7_, 499.1757, ∆ = 6.8 ppm). MS/MS spectrum: CCMSLIB00009918928.

### Wnt Activity Measurements

The Wnt-induced luciferase activity was analyzed as described. (Koval et al., 2014; Shaw et al., 2019) Briefly, 30 μl of BT-20 cells stably transfected with the TopFlash luciferase reporter (Koval et al., 2014) at 150,000 cells/ml were distributed in a white opaque 384-well plate. The cells were maintained in DMEM containing 10% FBS and incubated at 37°C, 5% CO_2_ overnight for attachment. Afterwards, the BT-20 cells were additionally transfected with the plasmid constitutively (under the CMV promoter) expressing *Renilla* luciferase (Addgene, Cambridge, MA, United States). Transfection was carried out as described in manufacturer’s protocol using 12 µg/ml of DNA and 40 µl/ml XtremeGENE 9 reagent (Roche). Next day, the medium of each well was replaced by 30 μL fresh medium containing Wnt3a (500 ng/ml) [purified as described (Koval et al., 2011] or CHIR99021 (1 µM) and the compounds at 6-8 different concentrations (following 1h of pre-incubation with the compound). After overnight incubation, the supernatant in each well was removed and the cells were harvested and analyzed for activities of both firefly and *Renilla* luciferase as described (Koval et al., 2014).

### MTT Assay

50 μl of indicated TNBC cell lines were added into each well of a transparent 384-well plate at density 1,500 cells/well. The cells were maintained in DMEM containing 10% FBS and incubated at 37°C, 5% CO_2_ overnight. The next day, the medium of each well was replaced by 50 μl fresh medium containing indicated concentrations of compounds. After incubation for 3–4 days, the medium in each well was replaced by 50 μl of 0.5 mg/ml Thiazolyl blue (Carl Roth) solution in 1xPBS. The plates were incubated for 3 h at 37°C. Then the solution was removed, and 25 μl DMSO was added into each well. Absorbance at 570 nm was measured in the Tecan Infinite 200 Pro reader.

### Western Blotting

TNBC cell lines and L-cells were seeded at 100,000 cells/well in 24 well plates. The next day, the medium was replaced with fresh medium pre-warmed at 37°C containing the indicated compounds. After further 24h incubation, the medium was removed, followed by washing with 500 μl of 1x PBS twice per well. The cells were lysed in the well by addition of 30 μl of ice-cold RIPA buffer (1x TBS, 4 mM EDTA, 1% Triton, 0.1% SDS, 1x Protease inhibitor cocktail (Roche)) and incubated on ice for 10 min. The samples were resuspended and then centrifuged at 18,000 g at 4°C to remove debris. 15 μl of the supernatants each were further analyzed by Western blot with antibodies against active β-catenin (Millipore), Axin2 (Abcam), c-Myc (Abcam) and α-Tubulin (Sigma) at 1:1,000 dilutions.

### Immunofluorescent Analysis

The indicated cell lines were seeded at 200,000 cells/well in the 6-well plates with poly-L-lysine-coated coverslips on the bottom. After 5 h of treatment with or without Wnt3a in presence or absence of **8**, the cells were washed 2x by PBS, fixed by 4%PFA at room temperature and stained and visualized as described above for tumor samples. The antibodies used for staining were against β-catenin (BD Biosciences) and pS33/37/T41-β-catenin (CST) at 1:200 and 1:100 dilution respectively.

### Similarity Analysis

The similarity matrix was calculated with all 73 compounds with the “Similarity and clustering” utility of Canvas (Schrödinger, LLC, New York, NY, release 2021-1) (Duan et al., 2010). A dendritic fingerprint of 64-bit was calculated with the daylight invariant atom types and bonds distinguished by bond order. The Tanimoto metric was chosen with the average linkage method to compute the matrix.

### Pharmacophore Model Hypothesis Development

The model was built with Phase and Maestro (Schrödinger, LLC, New York, NY, release 2021-1). The compounds were prepared with the LigPrep utility at pH 7.0 and the OPLS4 force field (Lu et al., 2021), then split into 3 groups: the “specific-activity” group (SI > 10, IC_50_ <35 μM), the “low specificity-active” group (SI < 10, IC_50_ < 35 μM) and the “low specificity-low activity” group (SI < 10, IC_50_ > 35 μM). The enantiomer for each compound was also generated. The pharmacophore method was to find the best alignment and common features, with a number of features in the hypothesis between 5 and 7, and a match of 100% of the input compounds. For the non-specific actives, matching 100% of the input compounds failed to produce any model, the matching percentage was then reduced to 75%. The output conformers were minimized. All other settings were the default ones. The model with the highest Phase Hypo Score was used for subsequent hypothesis validation with “Phase Ligand Screening” tab. The fitness score was used to assess the quality of the modes. Model images were rendered with PyMOL Molecular Graphics System (Schrödinger, LLC, New York, NY).

## Data Availability

The raw data files for the UHPLC-PDA-ELSD-MS analysis of the biotransformation reactions and NMR and HRMS data of the isolated compounds are available at the following link: https://doi.org/10.26037/yareta:zla4rtnowzfcdo6fqhqfec47jq. The MS/MS spectrum of each isolated compound has its own accession number from CCMSLIB00009918856 to CCMSLIB00009918928 on the Global Natural Product Social Molecular Networking (GNPS) (accessed via: https://gnps.ucsd.edu/ProteoSAFe/static/gnps-splash.jsp).

## Supplementary Material

The Supplementary Material for this article can be found online at: https://www.frontiersin.org/articles/10.3389/fchem.2022.881298/full#supplementary-material. NMR spectra of compounds 1 to 25 can be found in data sheet 2, those of compounds 26 to 47 in data sheet 3 and those of compounds 48 to 73 in data sheet 4.
