# Supplementary material for: Chemoenzymatic Synthesis of Original Stilbene Dimers Possessing Wnt Inhibition Activity in Triple-Negative Breast Cancer Cells Using the Enzymatic Secretome of Botrytis cinerea Pers
Source: Front Chem. 2022 Apr 19;10:881298. doi: 10.3389/fchem.2022.881298 (PMC9062038; doi:10.3389/fchem.2022.881298)



^{1}H NMR spectrum of compound **1** in DMSO-d_6

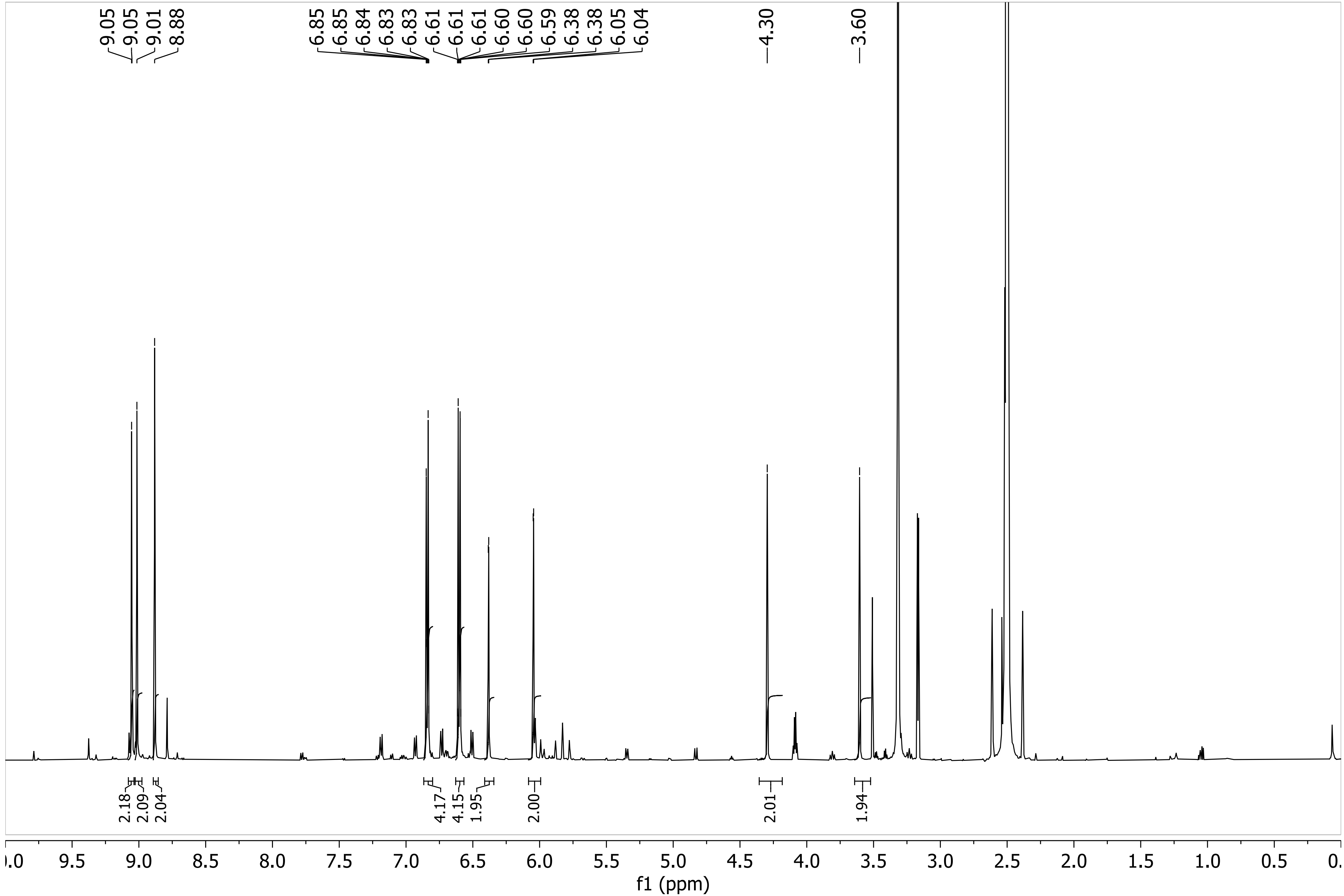

^{1}H NMR spectrum of compound **2** in $(CD_3)_2$-CO

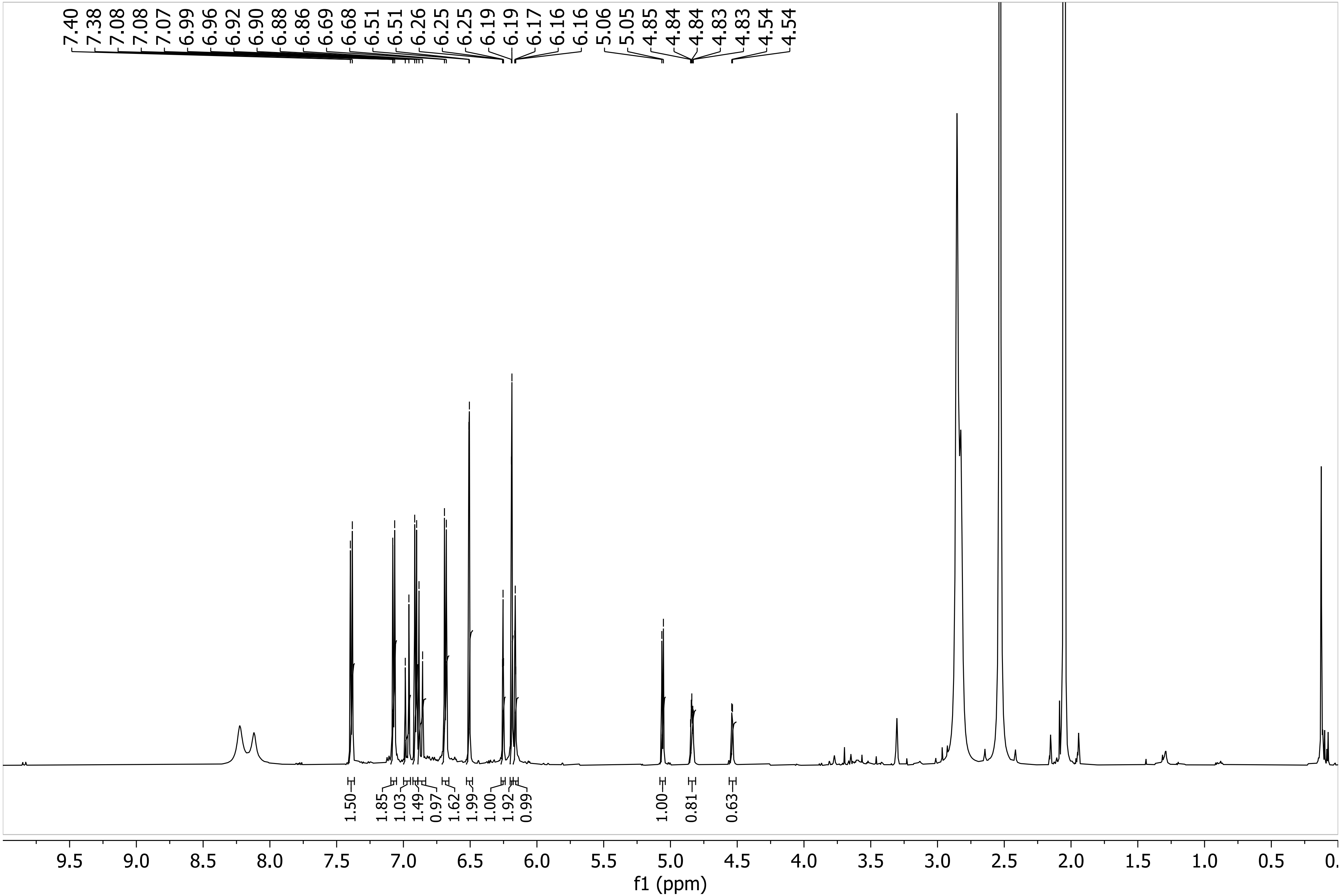

COSY NMR spectrum of compound **2** in $(CD_3)_2$-CO

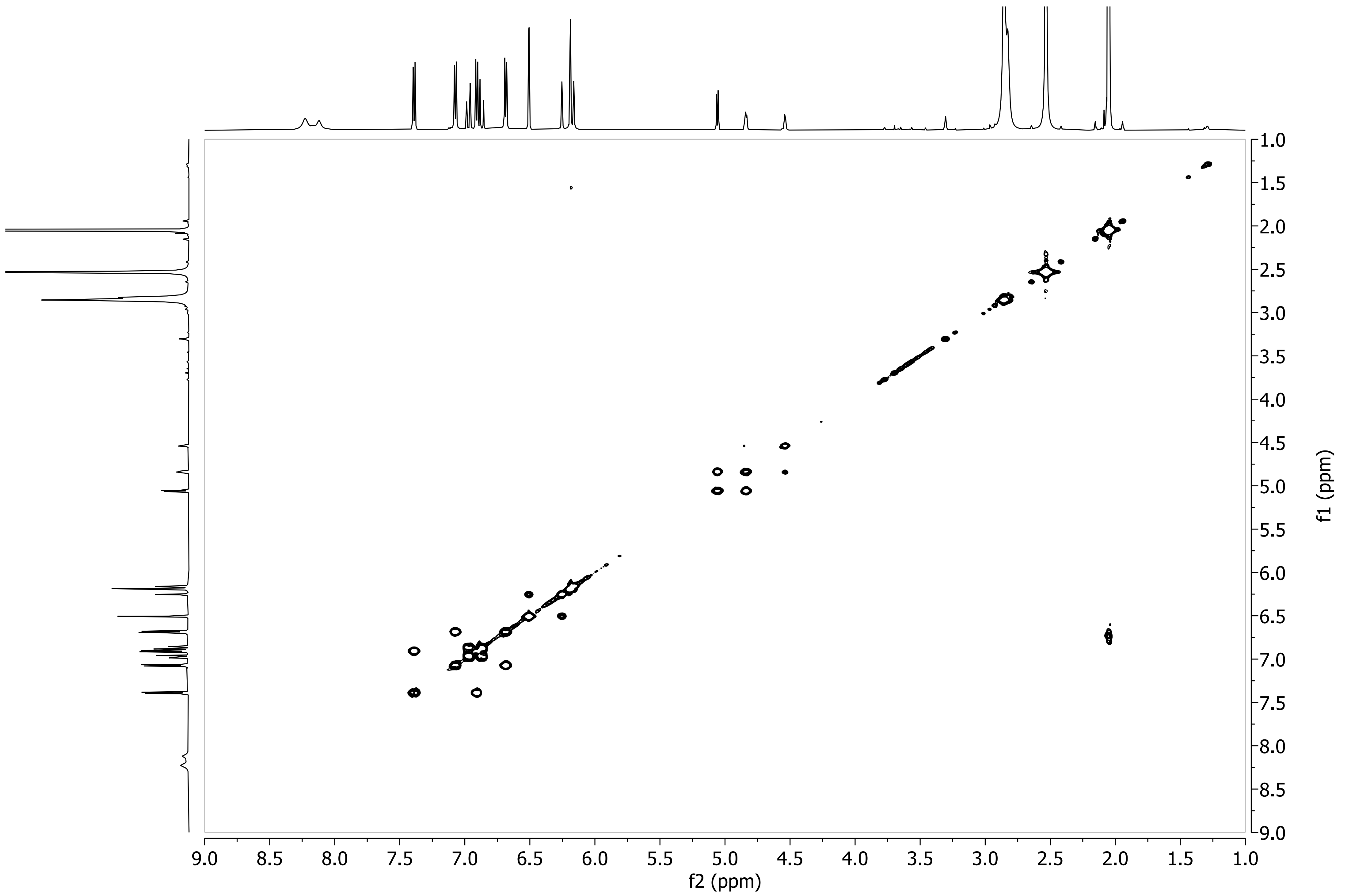

^{13}C-DEPTQ NMR spectrum of compound **2** in $(CD_3)_2$-CO

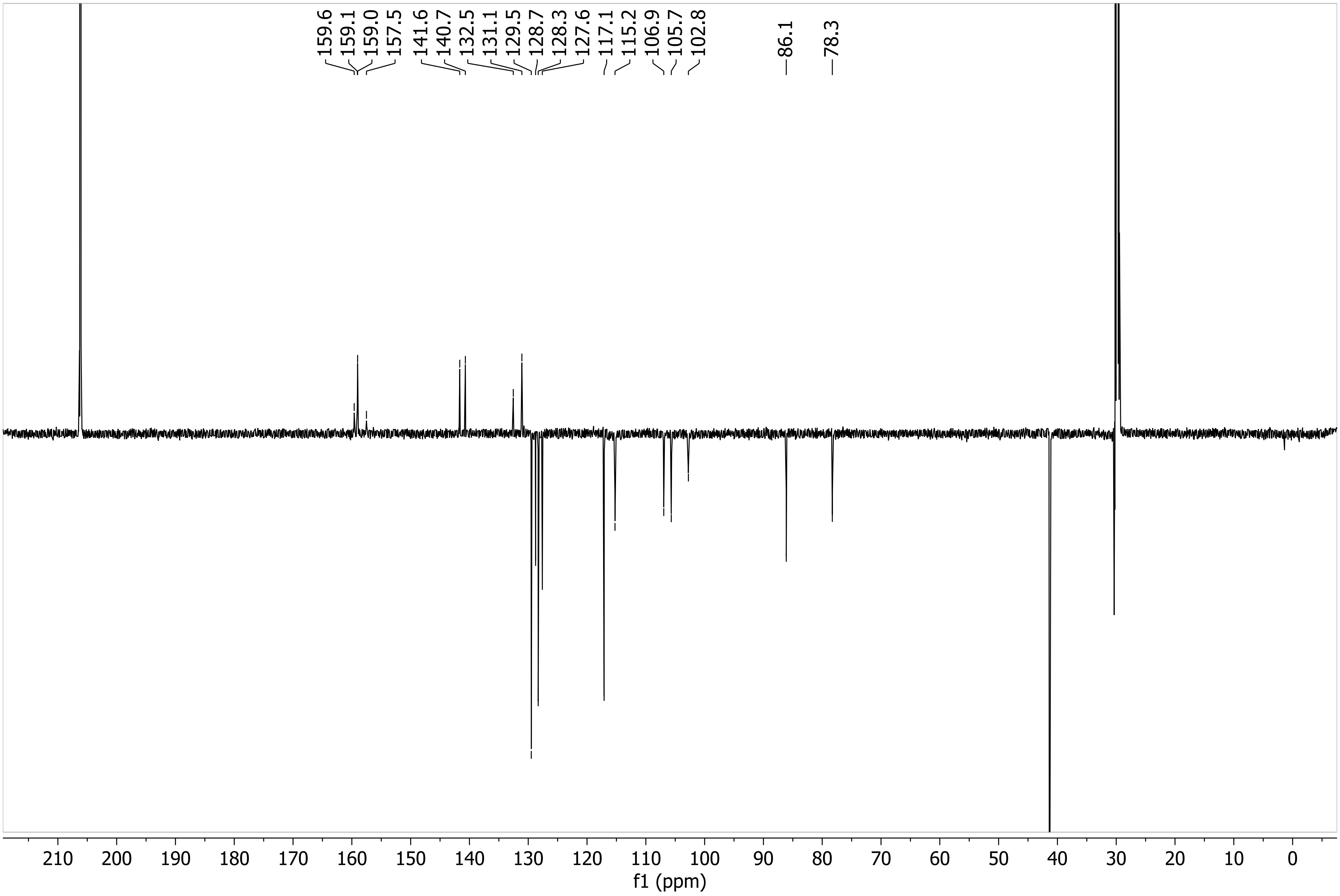

Edited-HSQC NMR spectrum of compound **2** in $(CD_3)_2$-CO

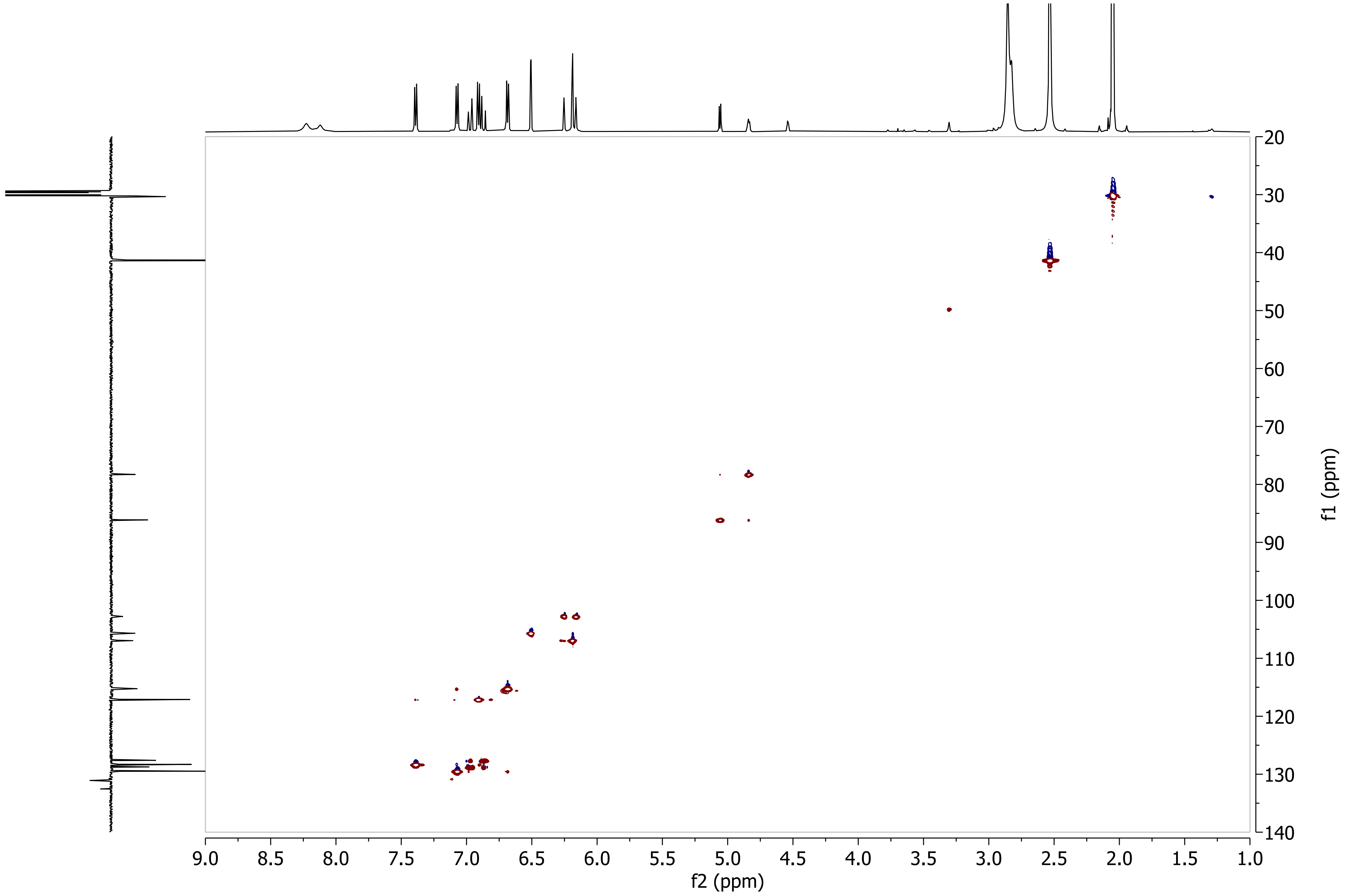

HMBC NMR spectrum of compound **2** in $(CD_3)_2$-CO

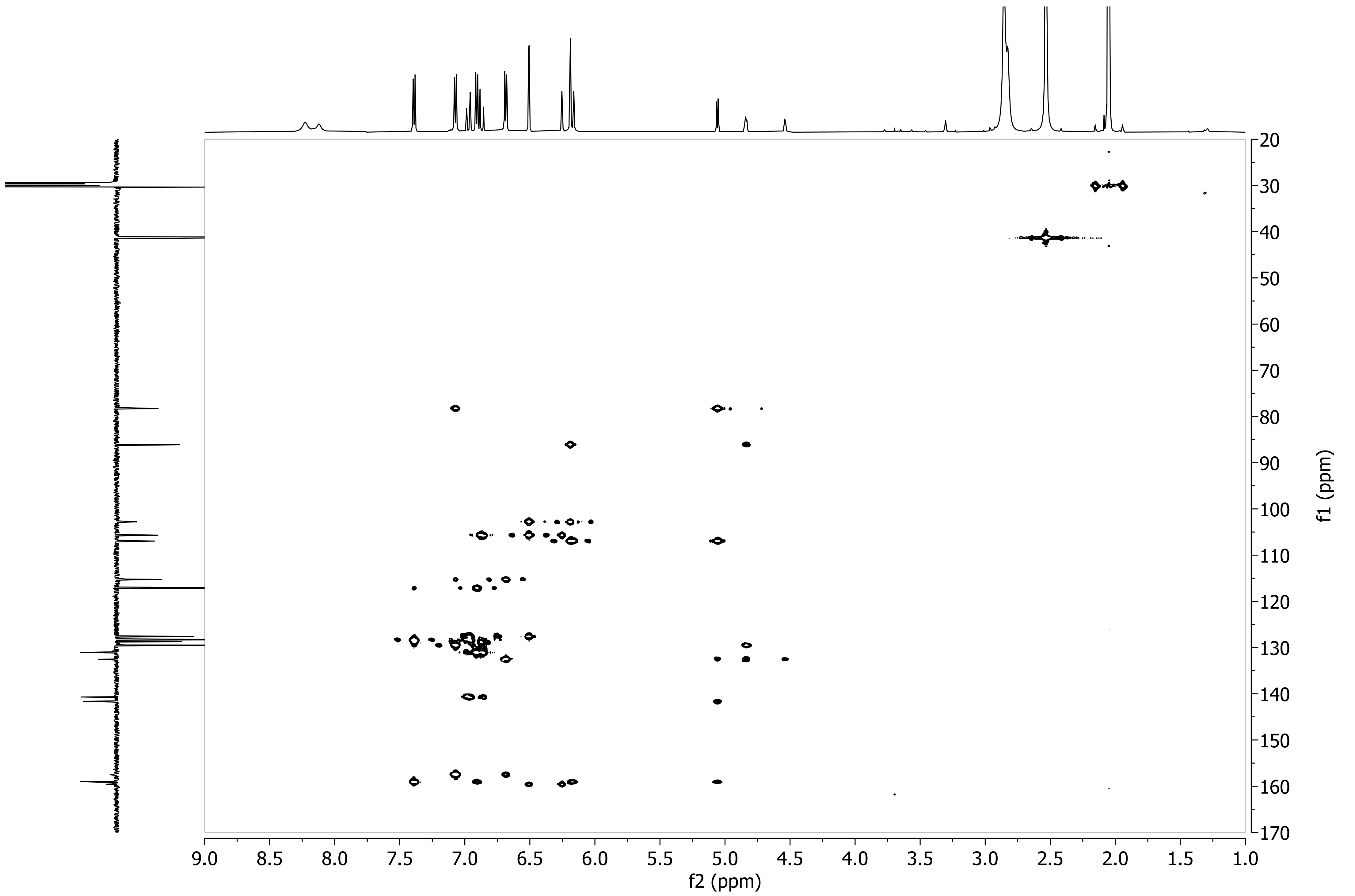

ROESY NMR spectrum of compound **2** in $(CD_3)_2$-CO

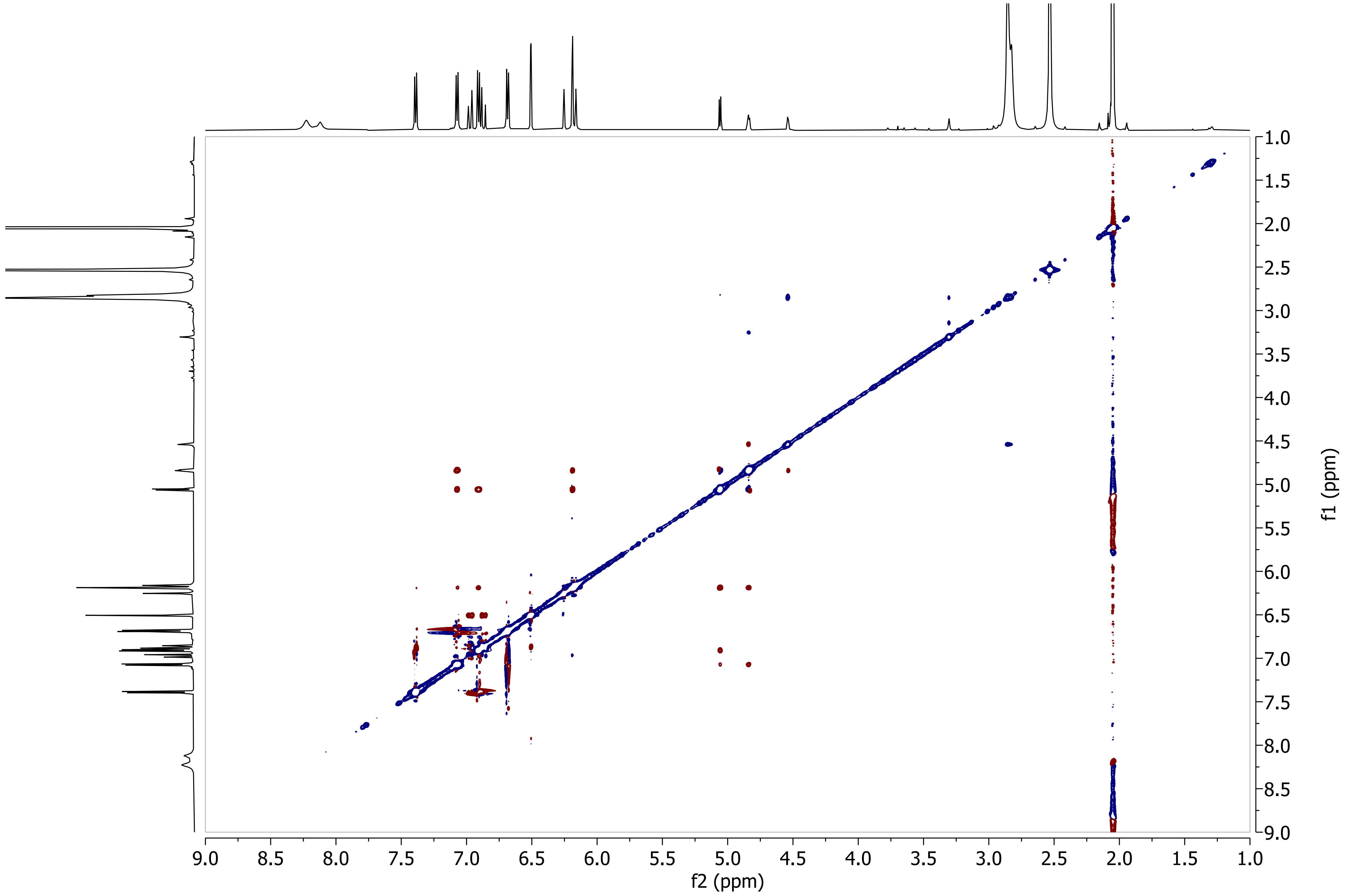

^{1}H NMR spectrum of compound **3** in $(CD_3)_2$-CO

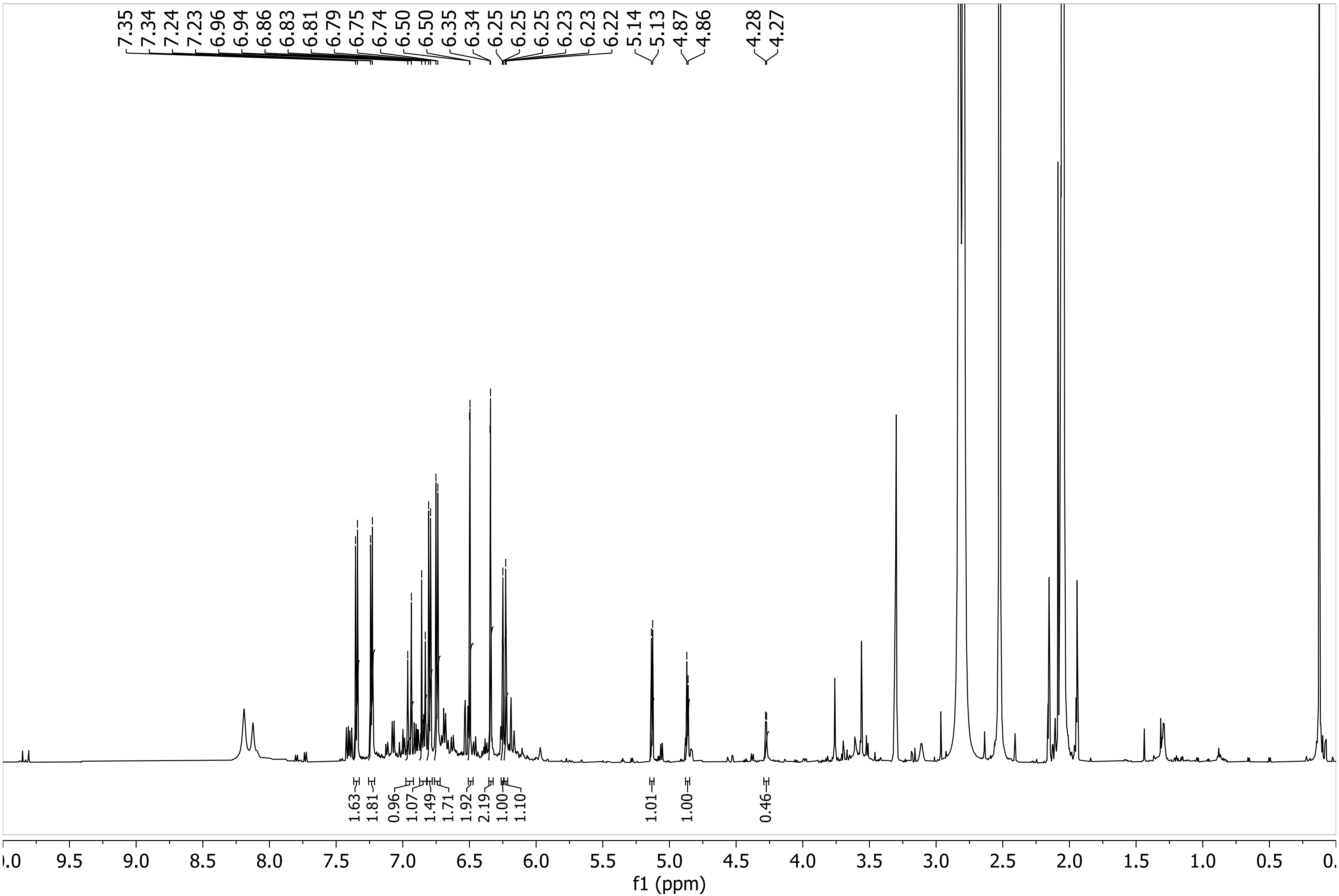

COSY NMR spectrum of compound **3** in $(CD_3)_2$-CO

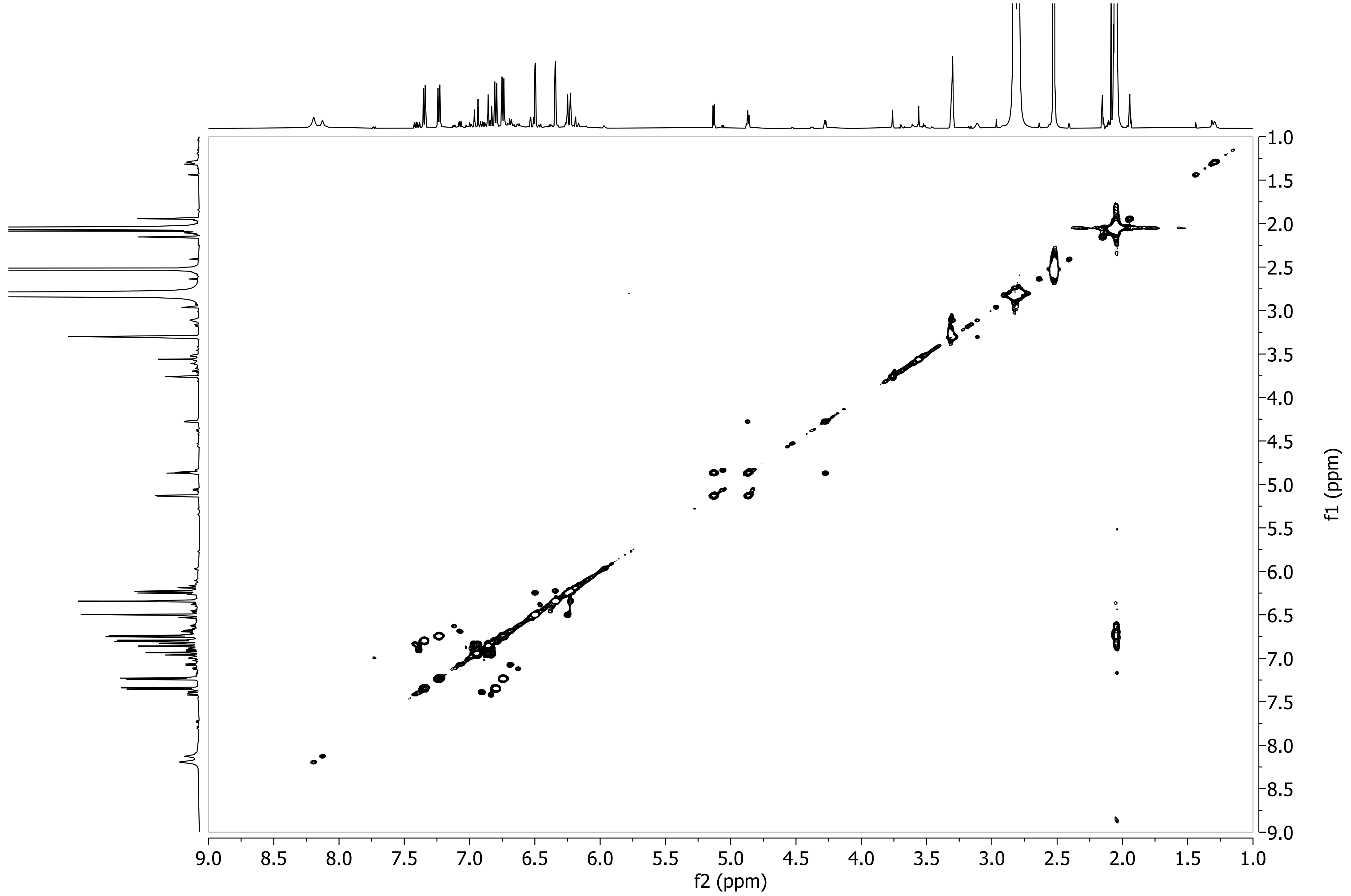

^{13}C-DEPTQ NMR spectrum of compound **3** in $(CD_3)_2$-CO

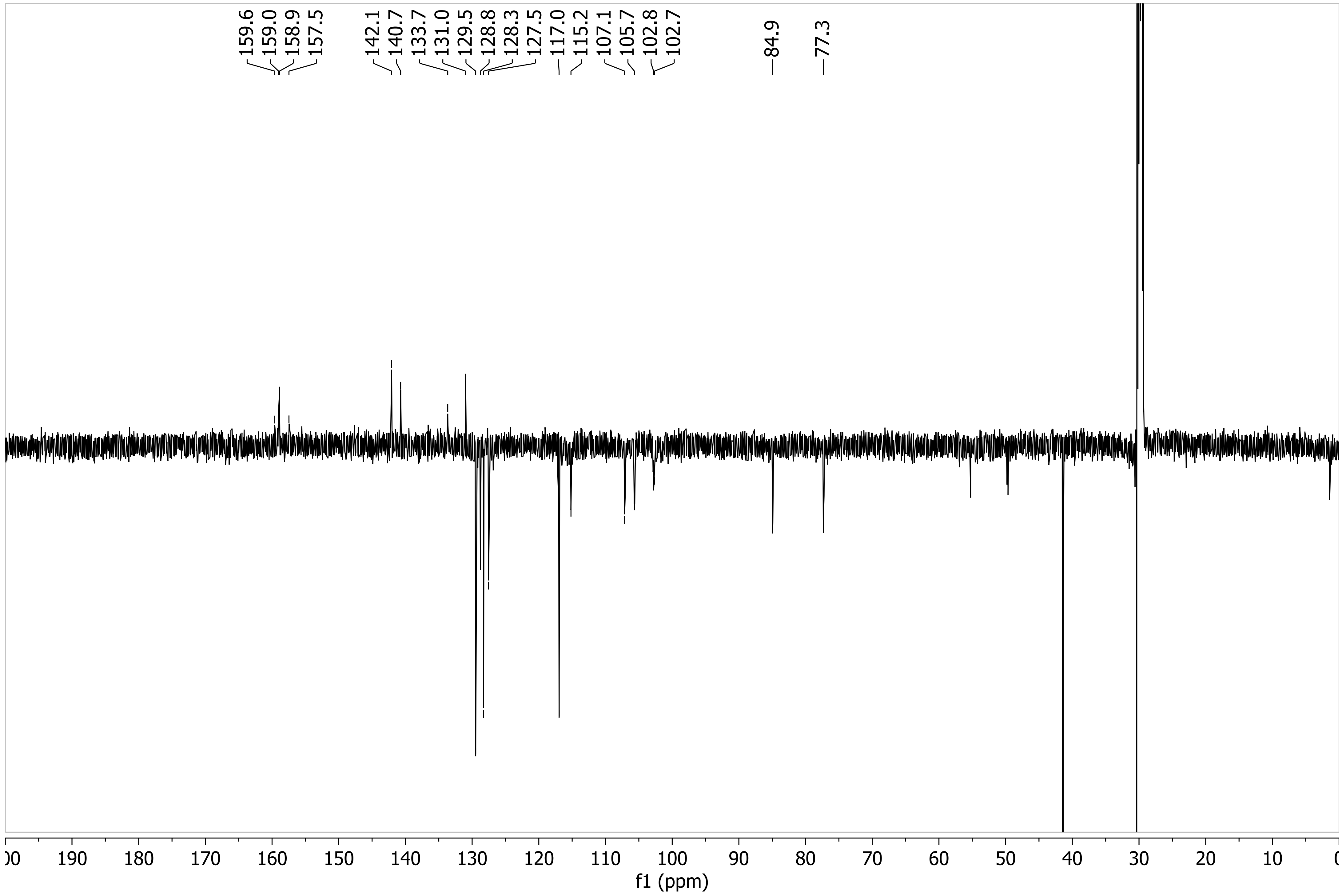

Edited-HSQC NMR spectrum of compound **3** in $(CD_3)_2$-CO

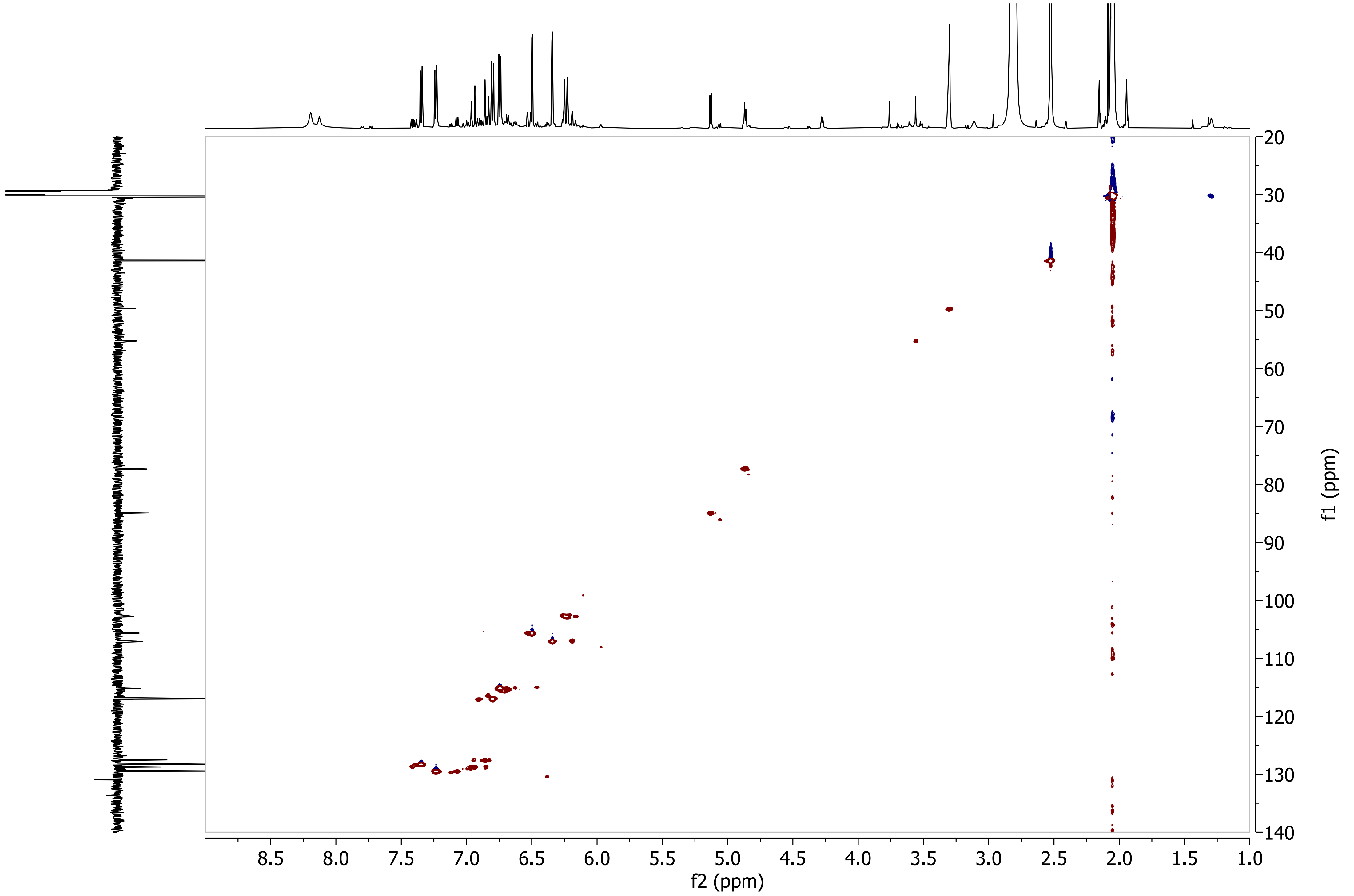

HMBC NMR spectrum of compound **3** in $(CD_3)_2$-CO

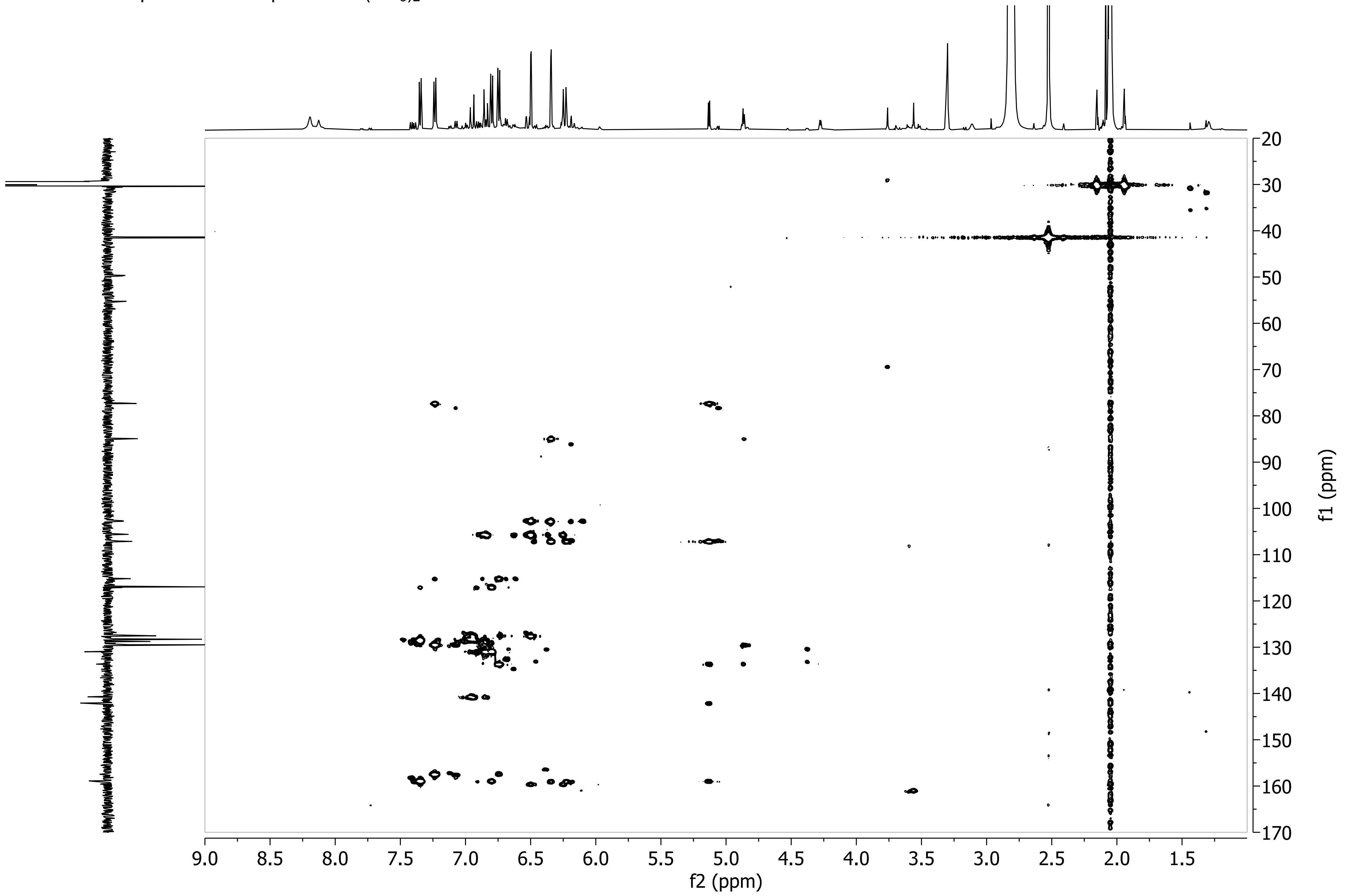

ROESY NMR spectrum of compound **3** in $(CD_3)_2$-CO

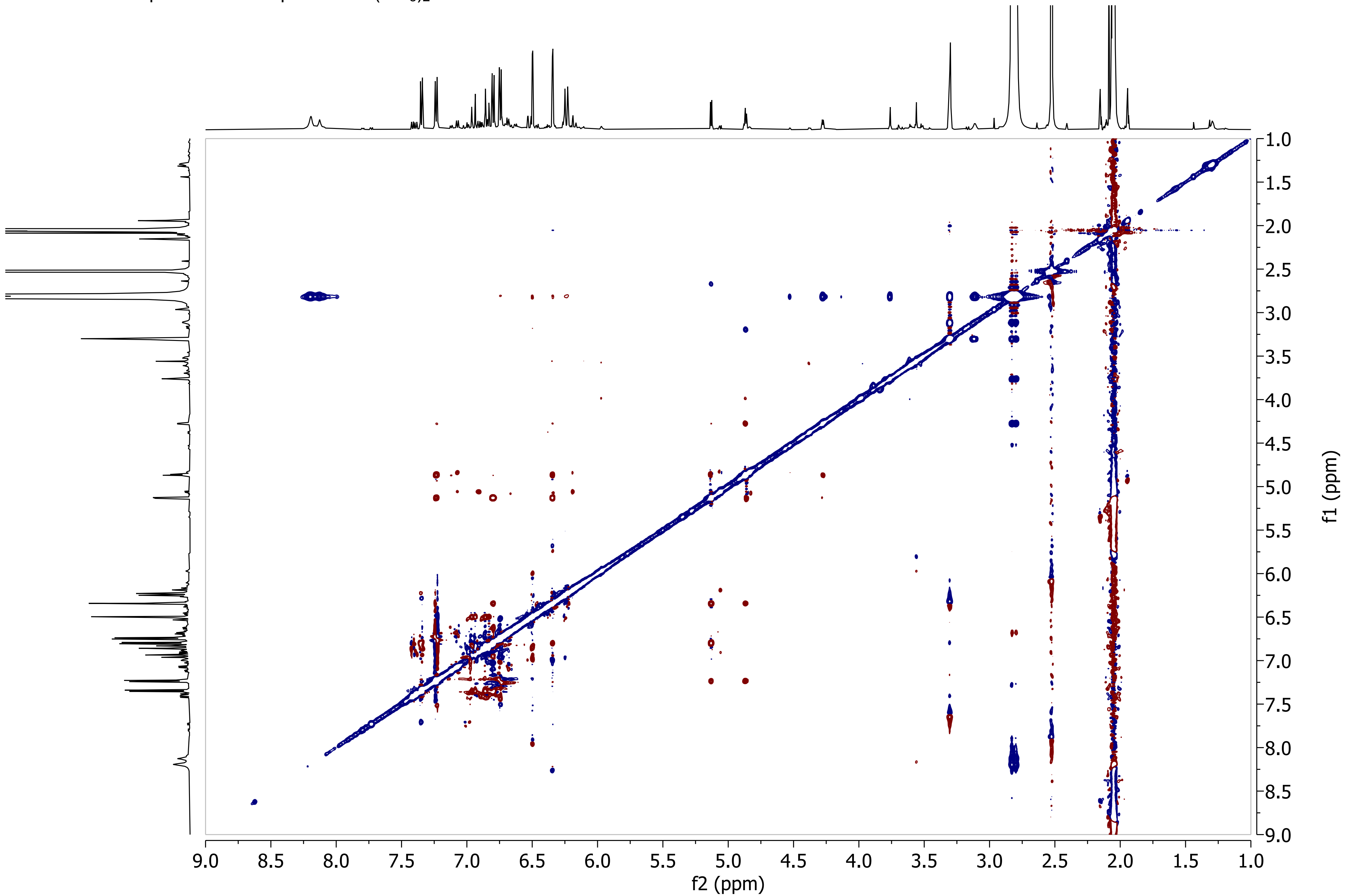

^{1}H NMR spectrum of compound **4** in DMSO-d_6

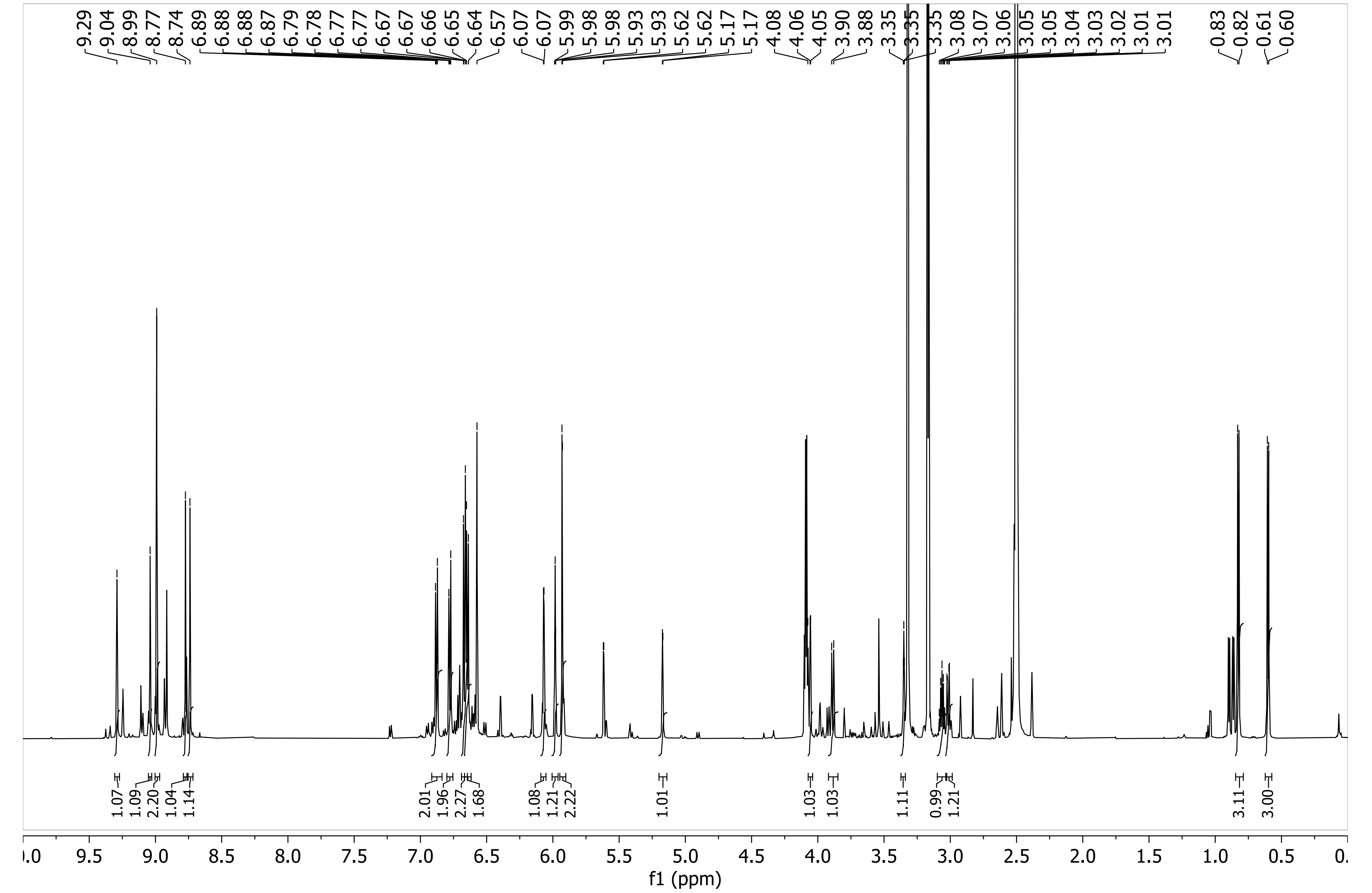

^{1}H NMR spectrum of compound **4** in DMSO-d_6

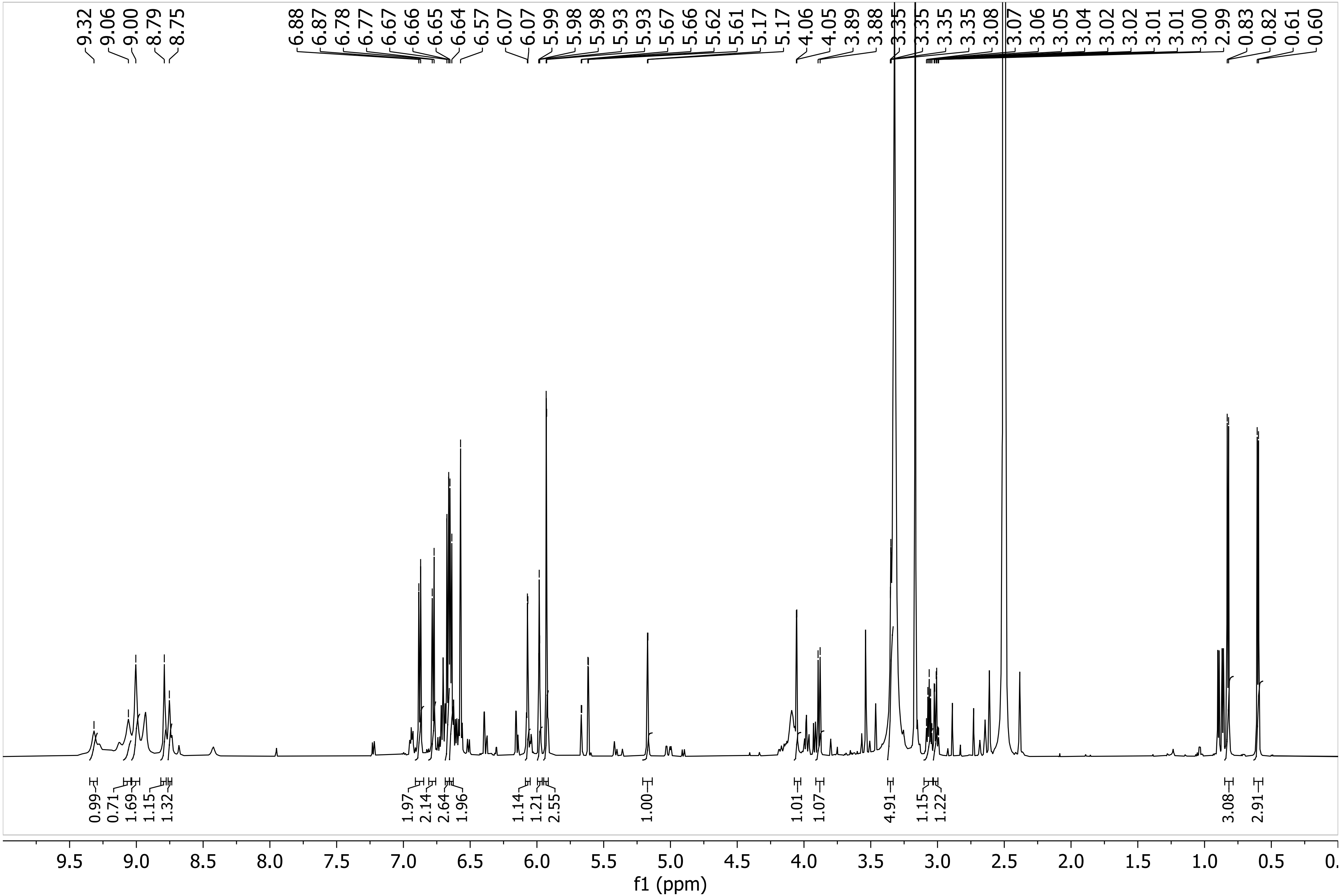

COSY NMR spectrum of compound **4** in DMSO-d_6

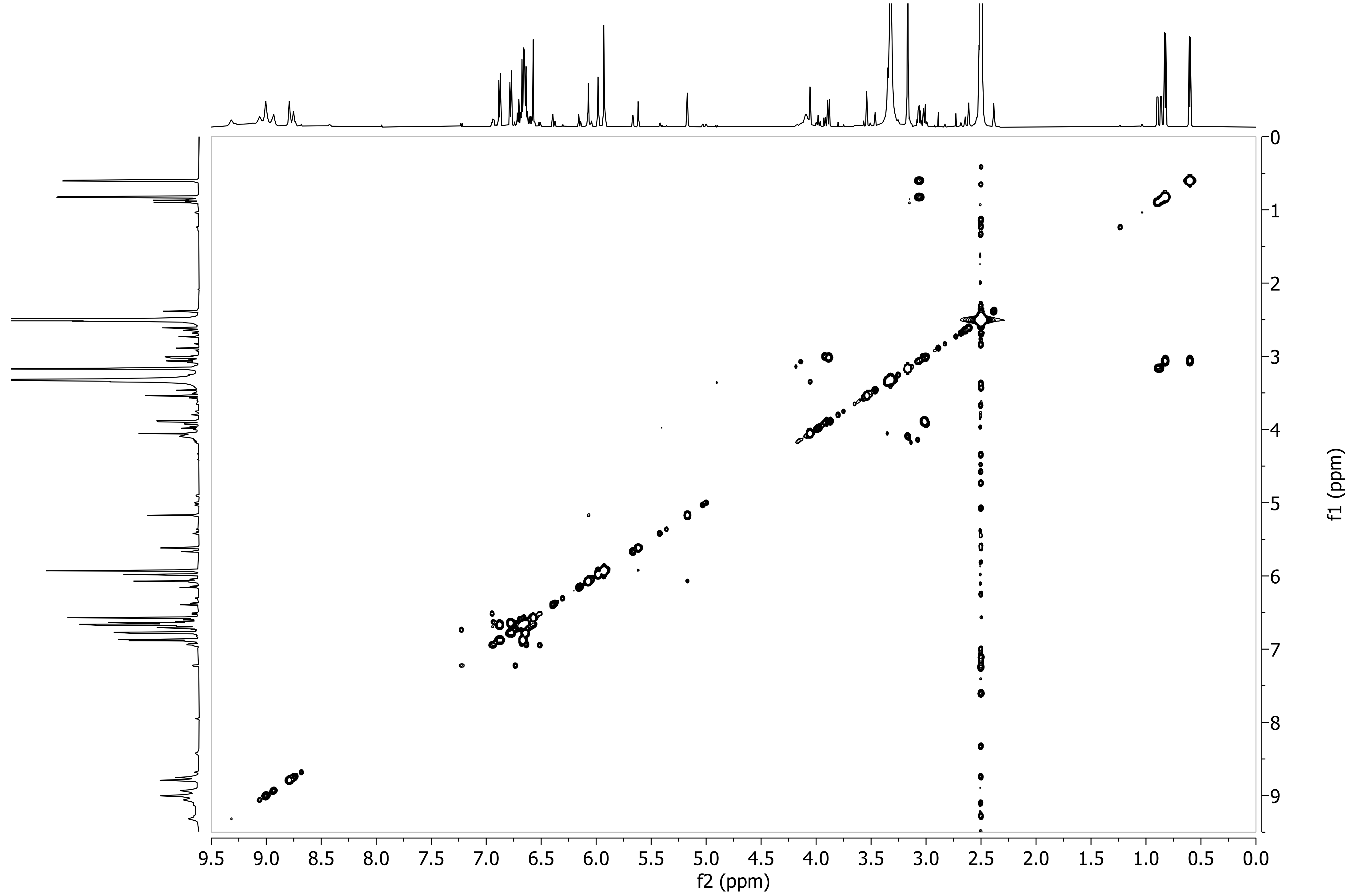

^{13}C-DEPTQ NMR spectrum of compound **4** in DMSO-d_6

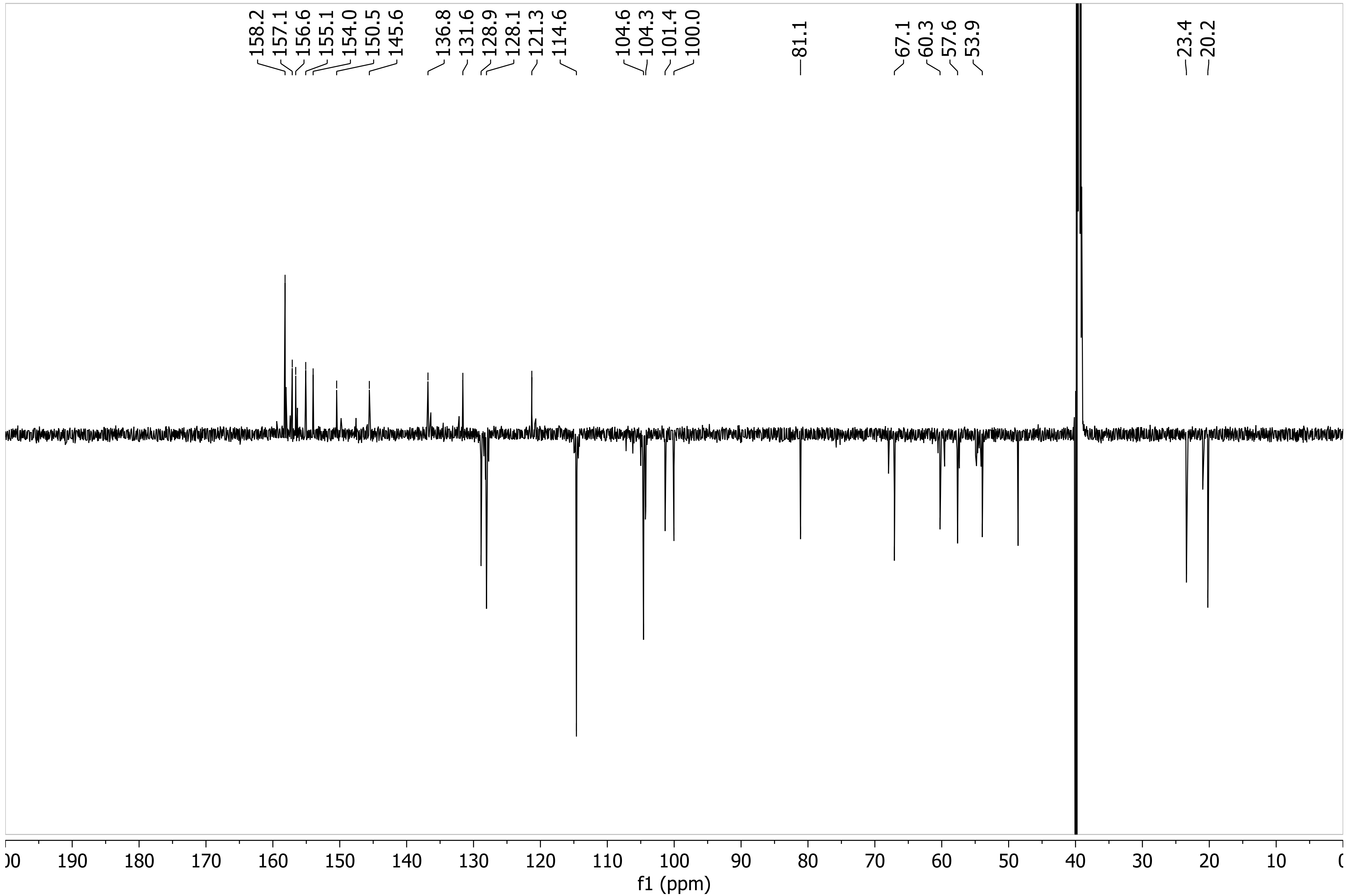

Edited-HSQC NMR spectrum of compound **4** in DMSO-d_6

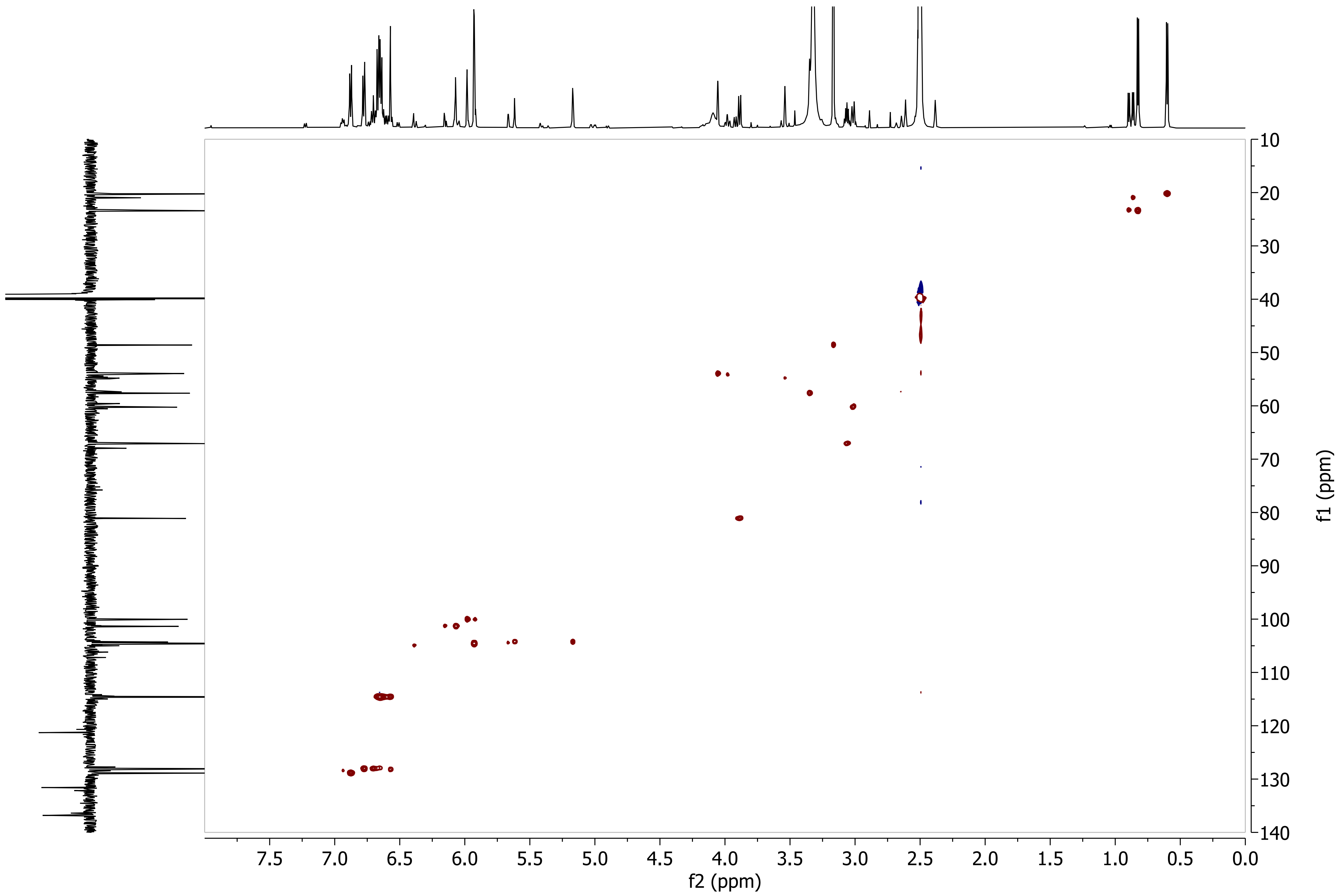

HMBC NMR spectrum of compound **4** in DMSO-d_6

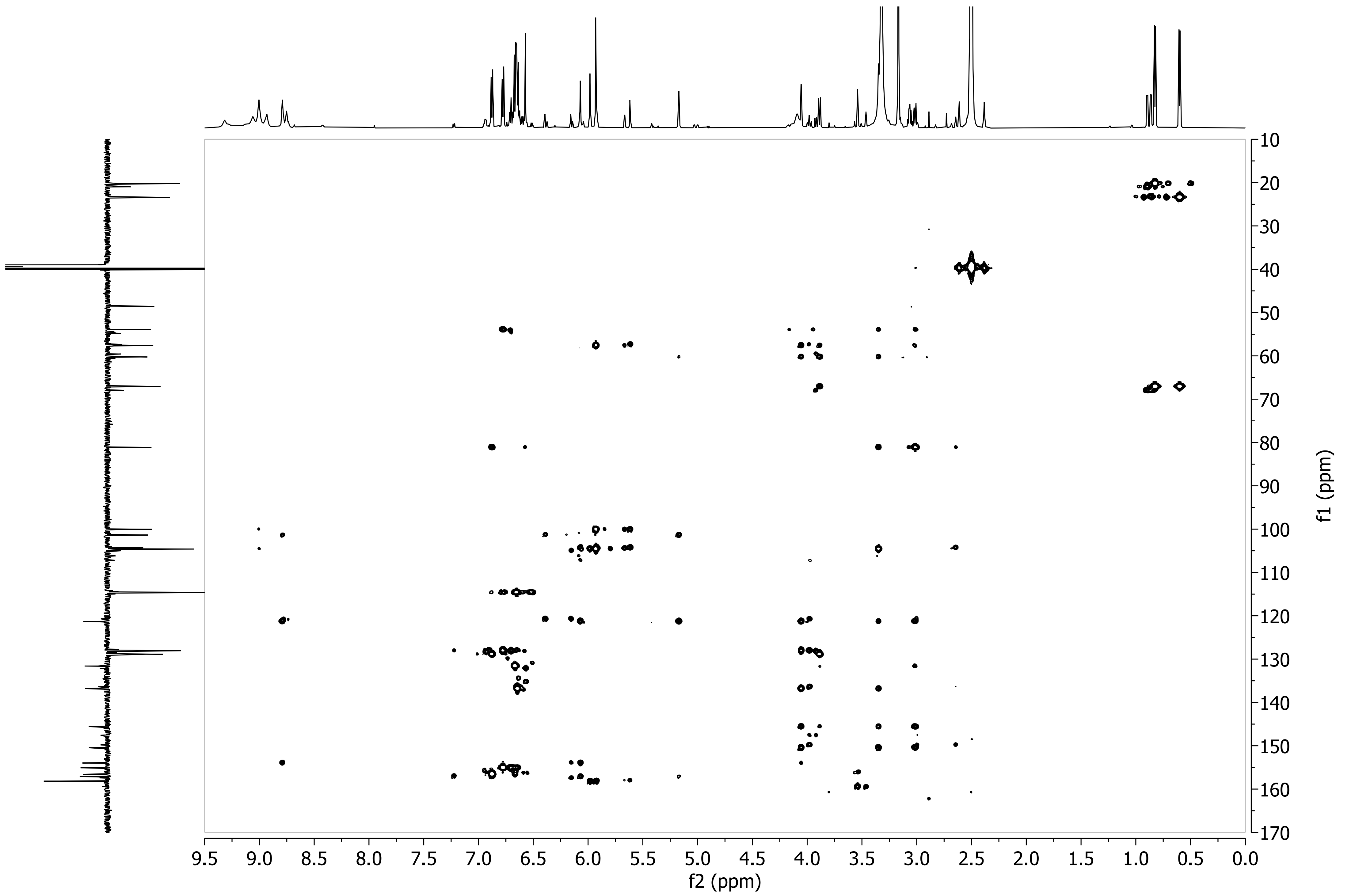

ROESY NMR spectrum of compound **4** in DMSO-d_6

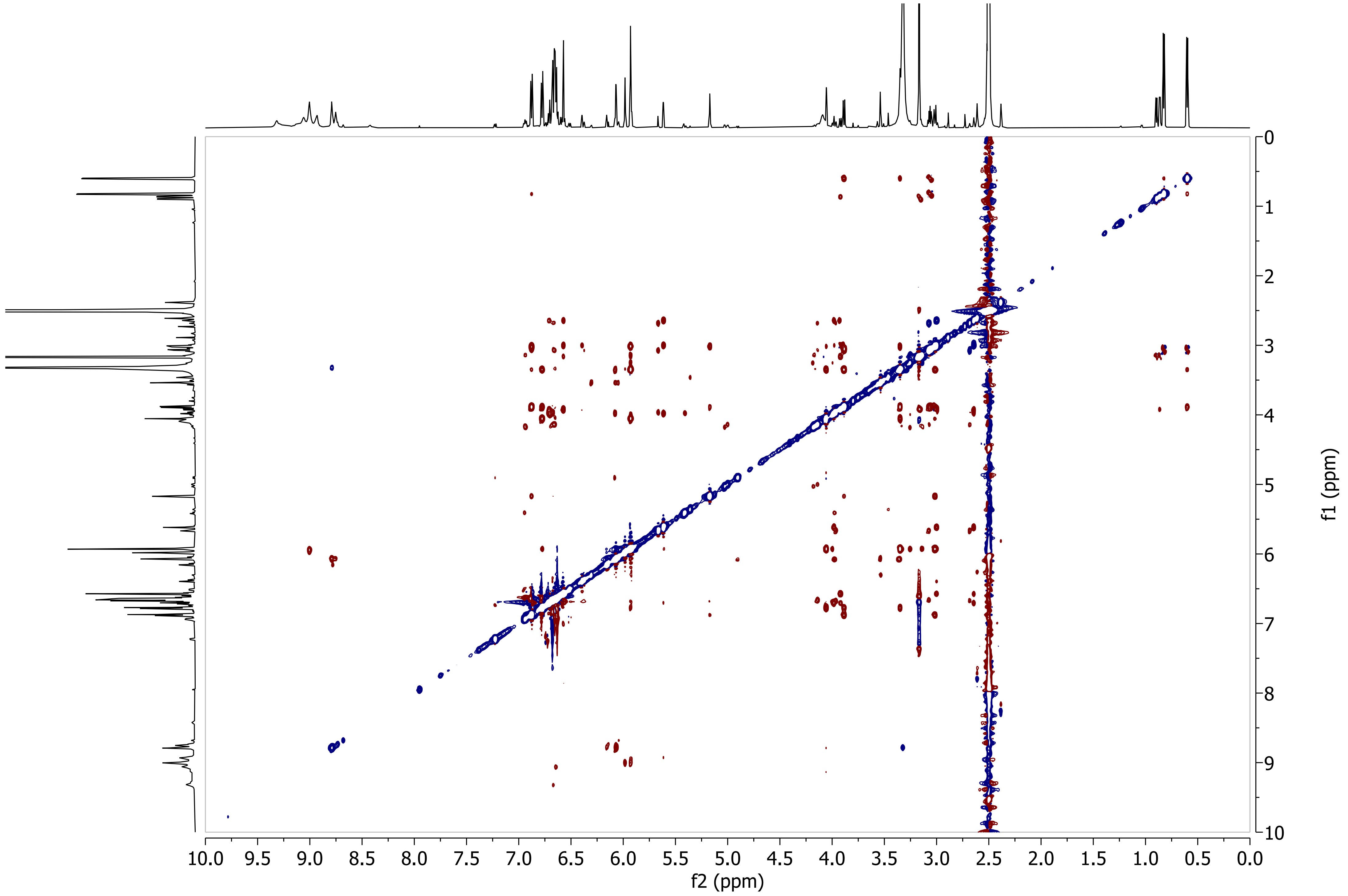

^{1}H NMR spectrum of compound **5** in DMSO-d_6

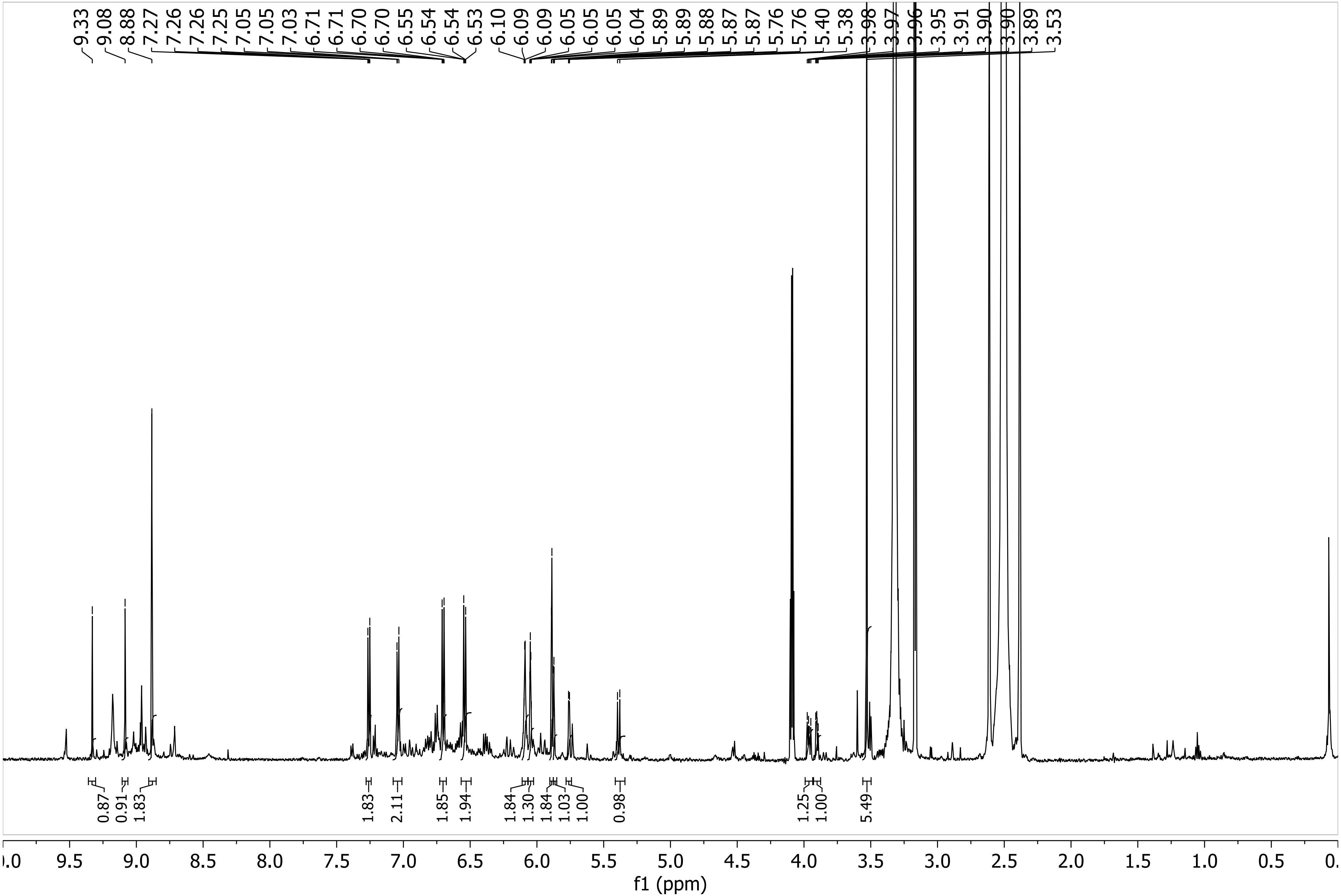

COSY NMR spectrum of compound **5** in DMSO-d_6

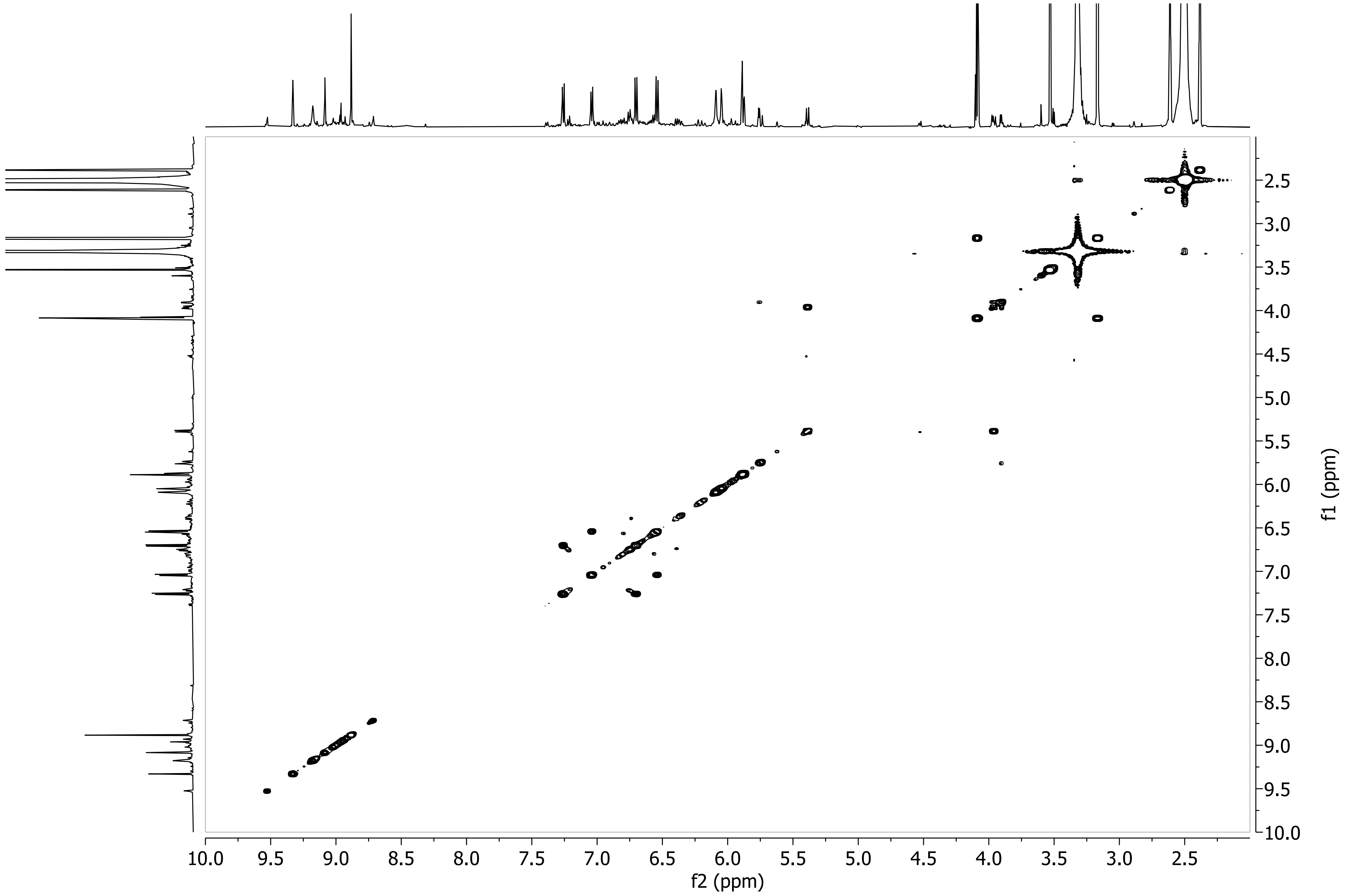

Edited-HSQC NMR spectrum of compound **5** in DMSO-d_6

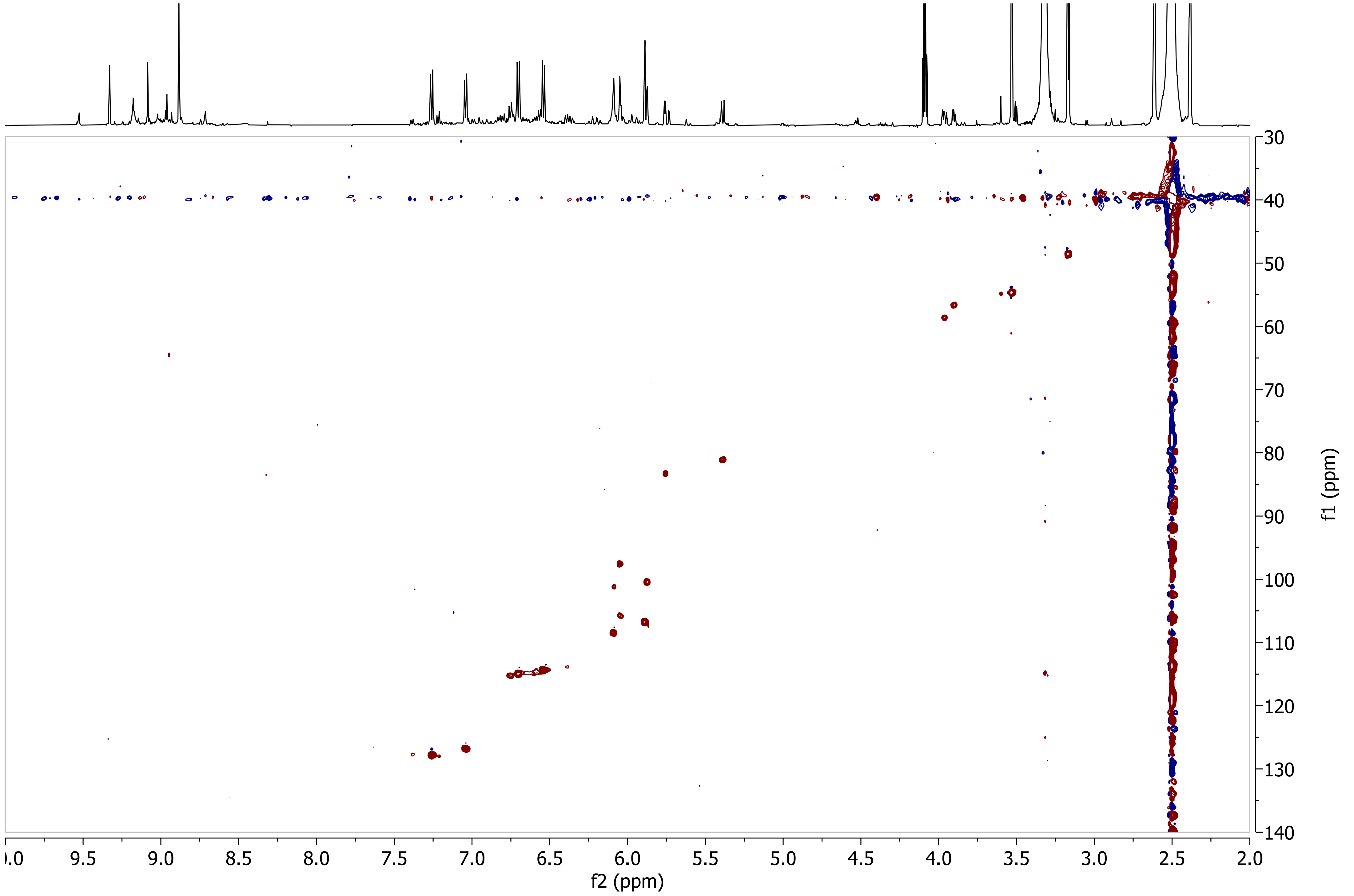

HMBC NMR spectrum of compound **5** in DMSO-d_6

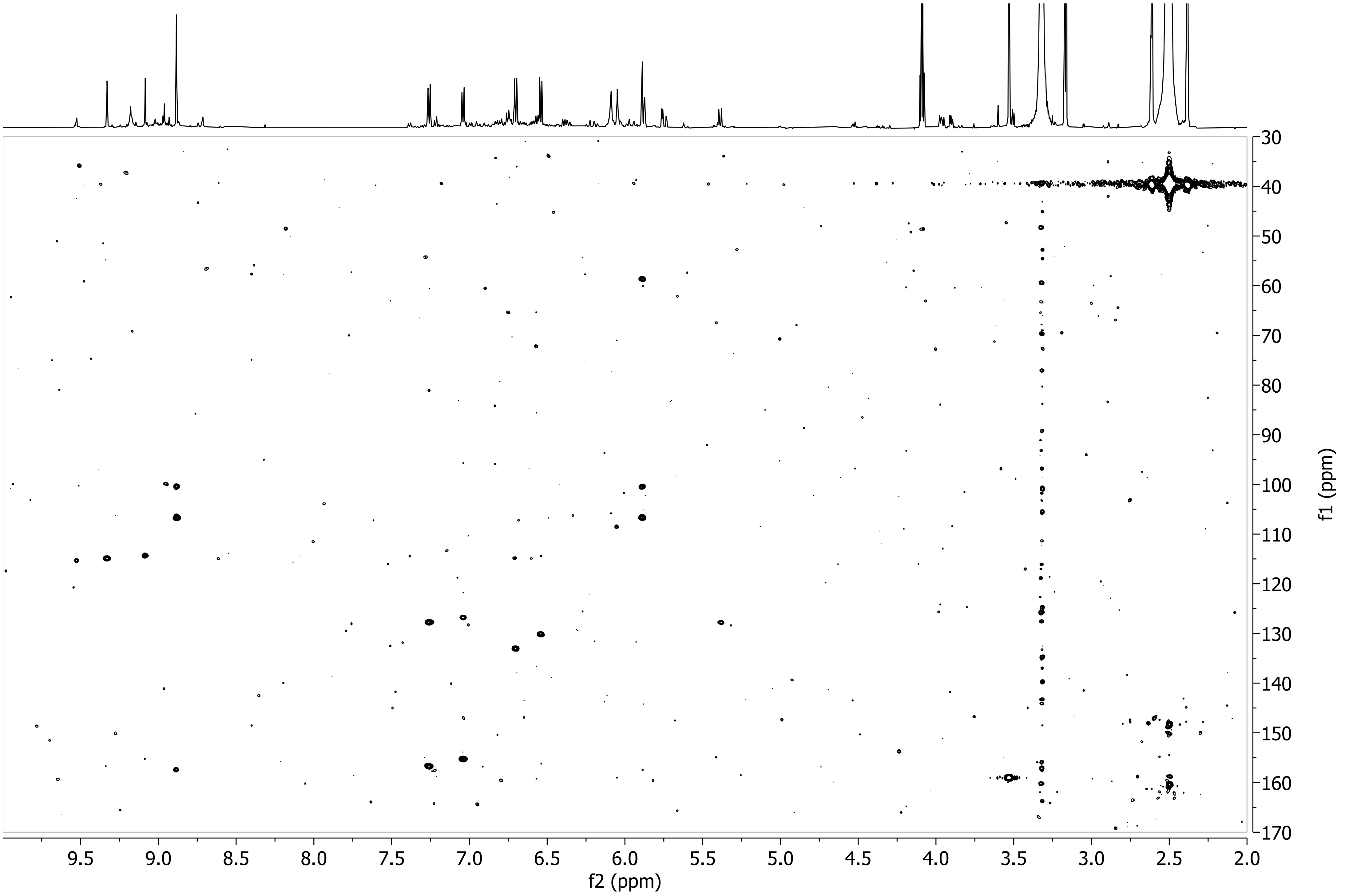

ROESY NMR spectrum of compound **5** in DMSO-d_6

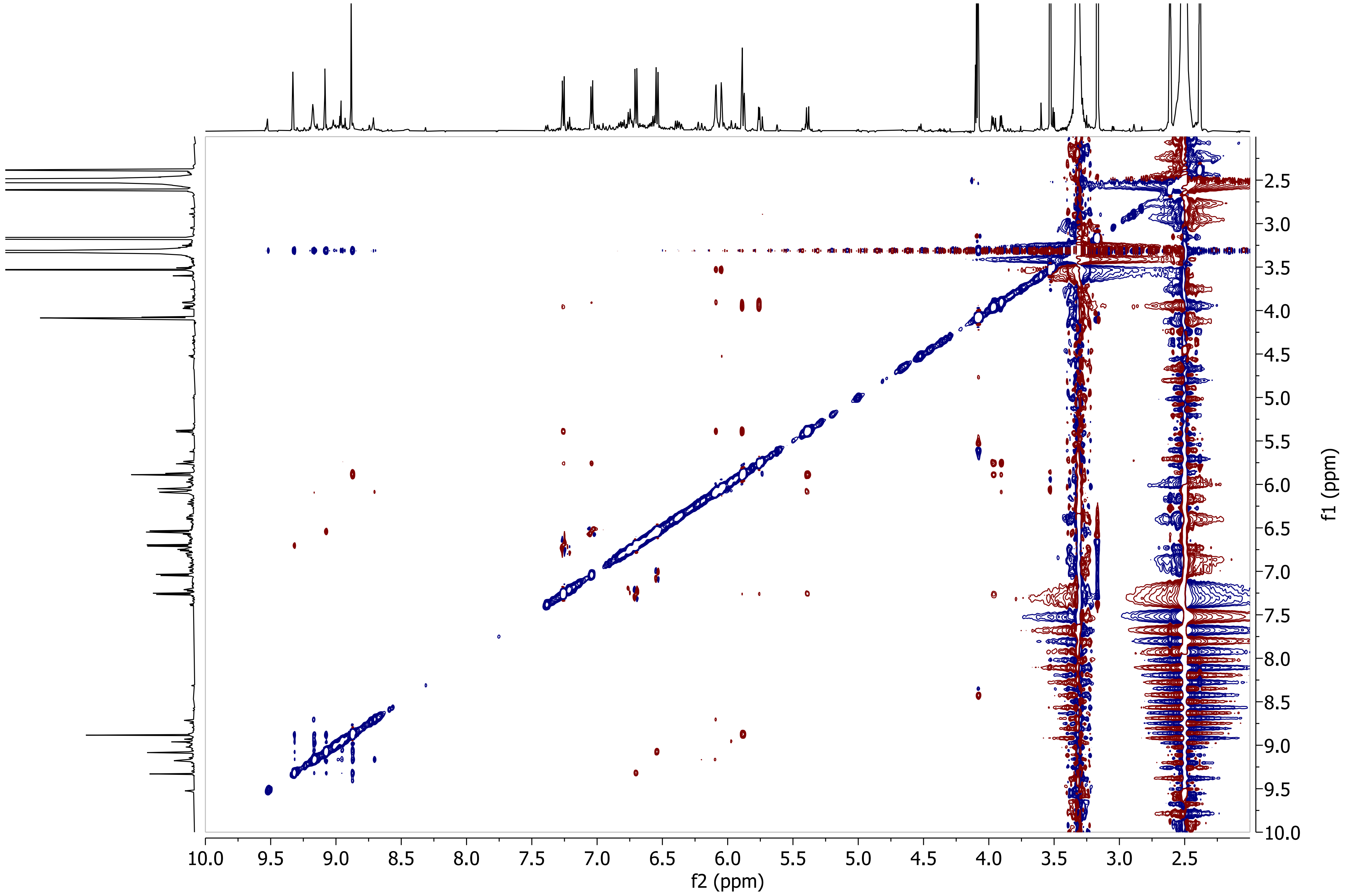

^1H NMR spectrum of compound **6** in DMSO-d_6

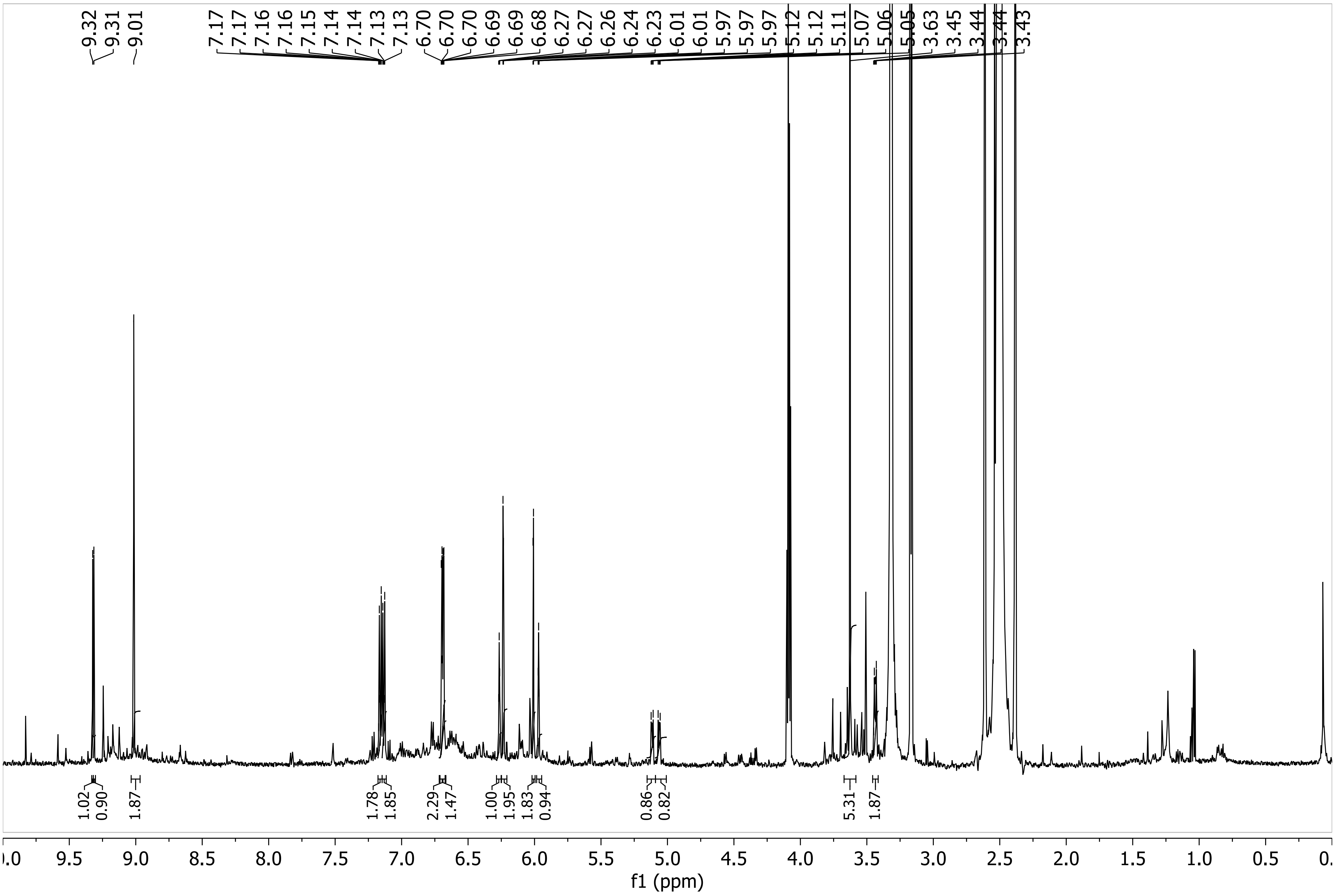

COSY NMR spectrum of compound **6** in DMSO-d_6

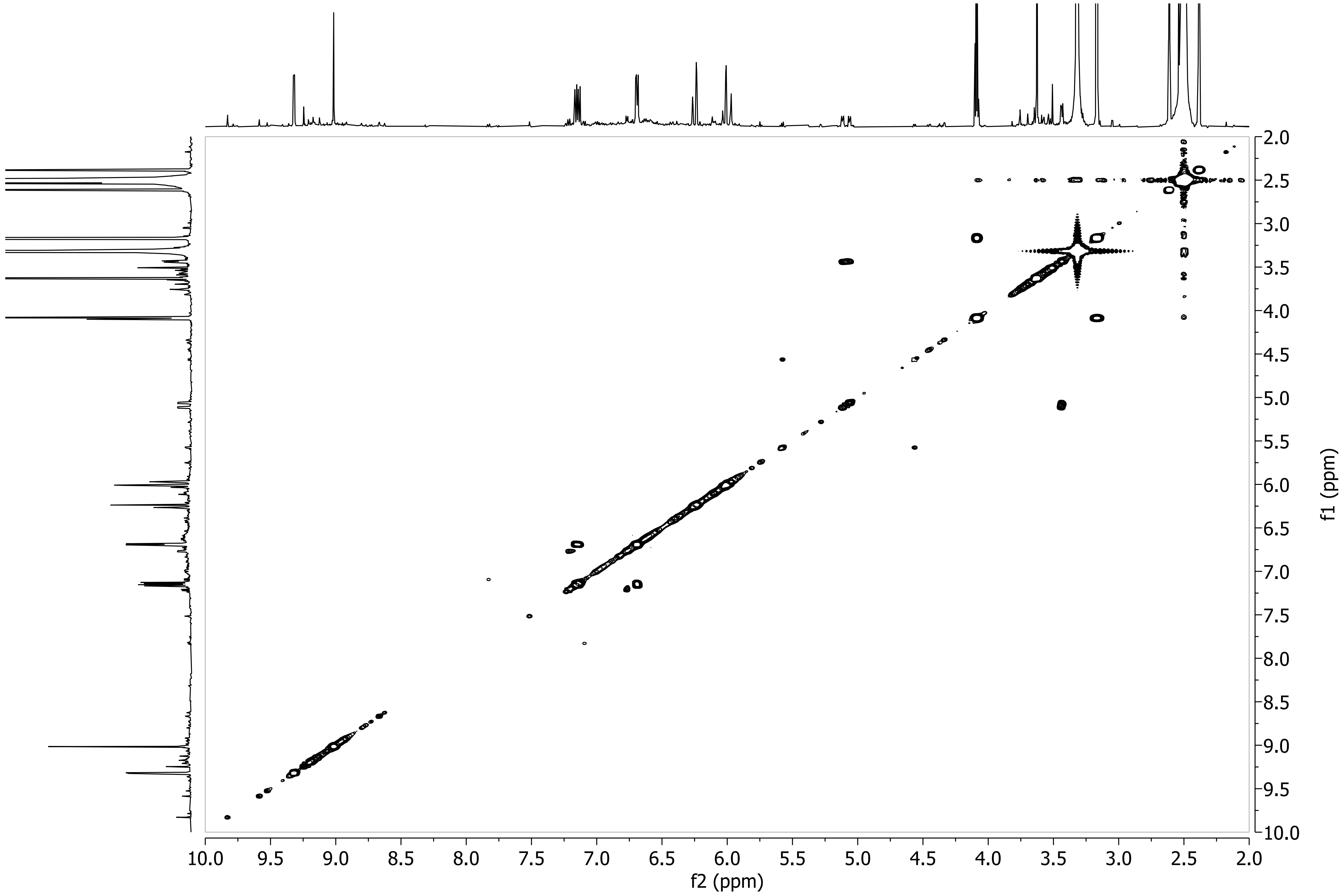

Edited-HSQC NMR spectrum of compound **6** in DMSO-d_6

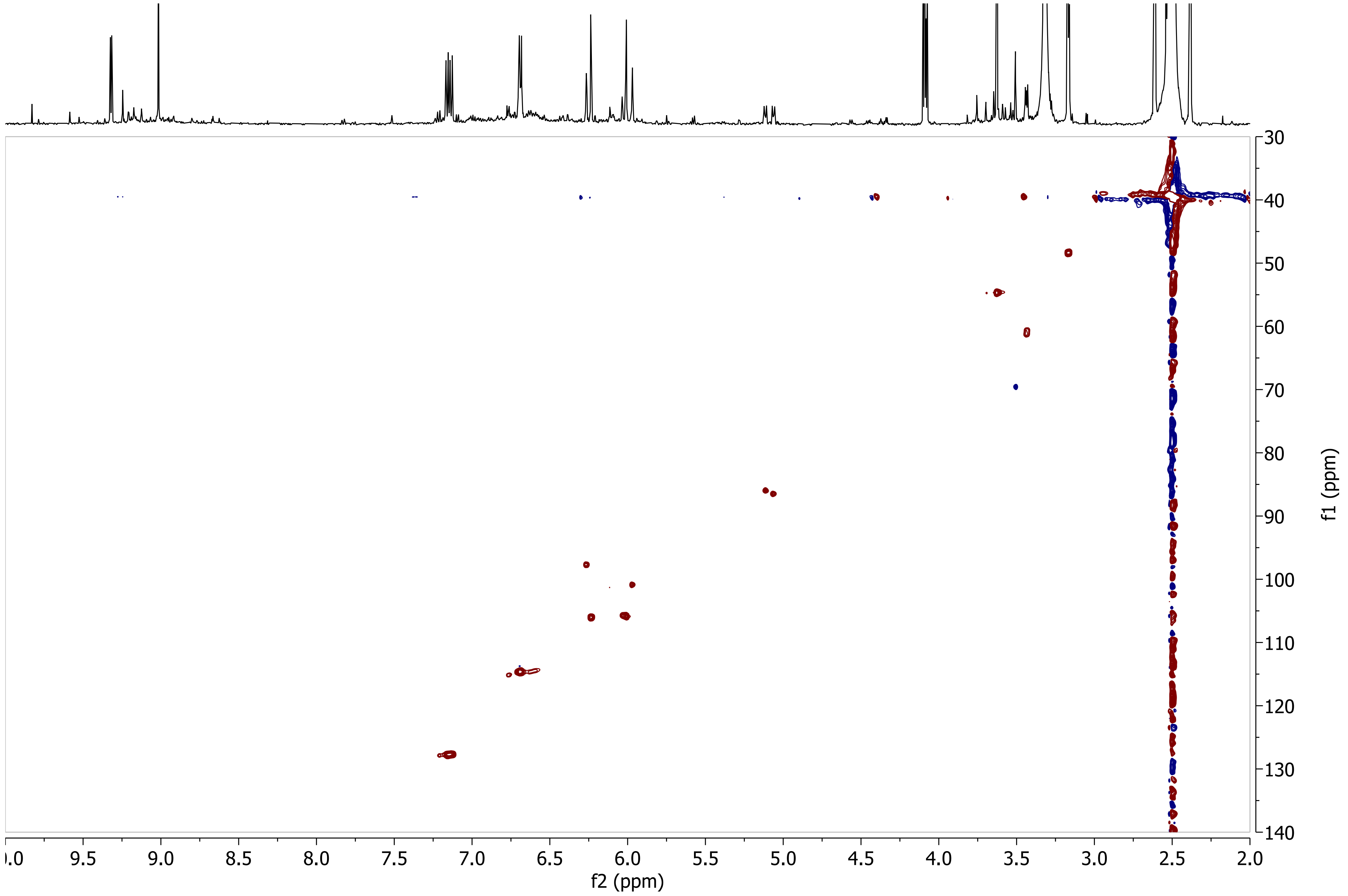

HMBC NMR spectrum of compound **6** in DMSO-d_6

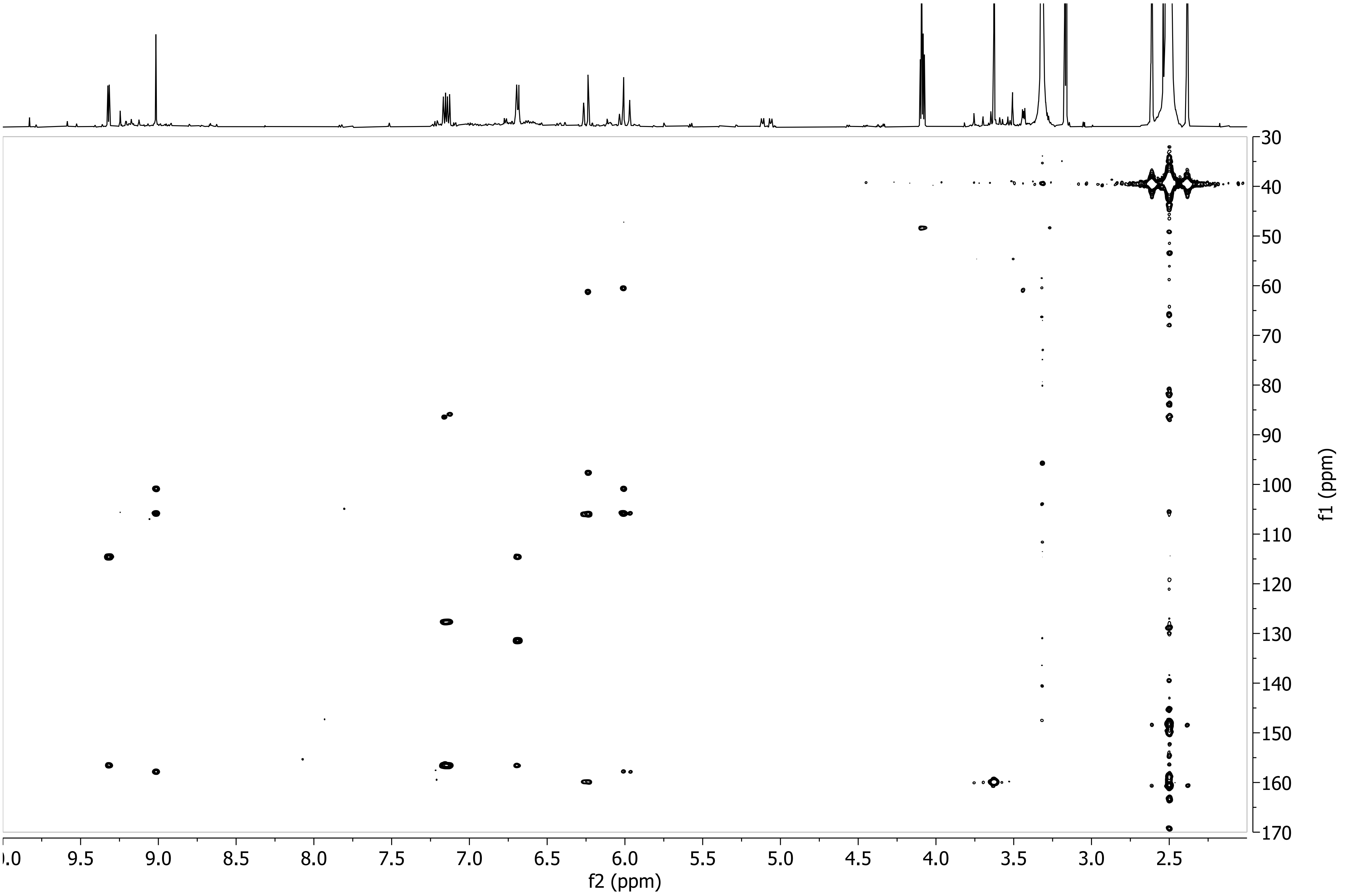

ROESY NMR spectrum of compound **6** in DMSO-d_6

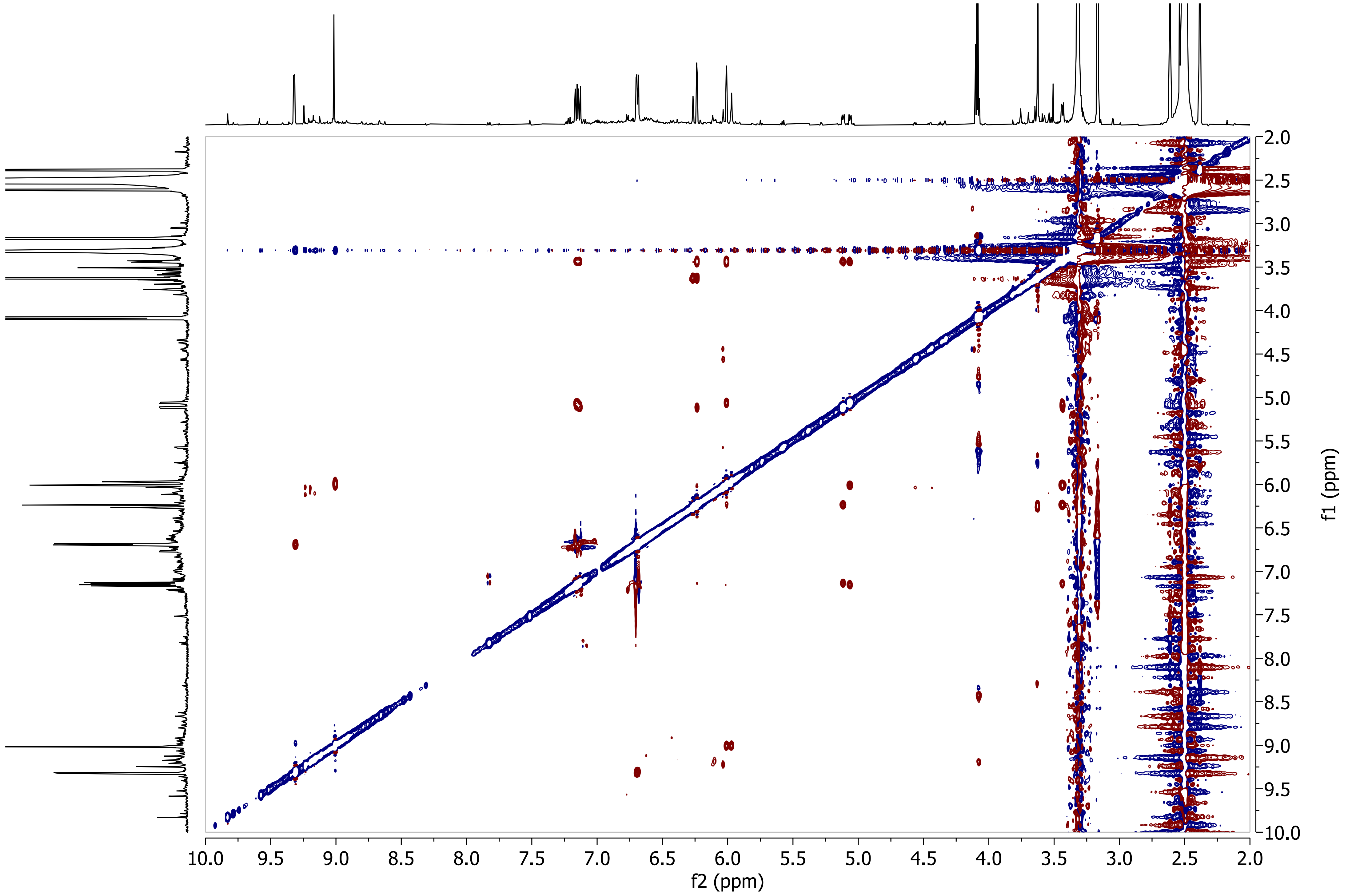

^{1}H NMR spectrum of compound **7** in DMSO-d_6

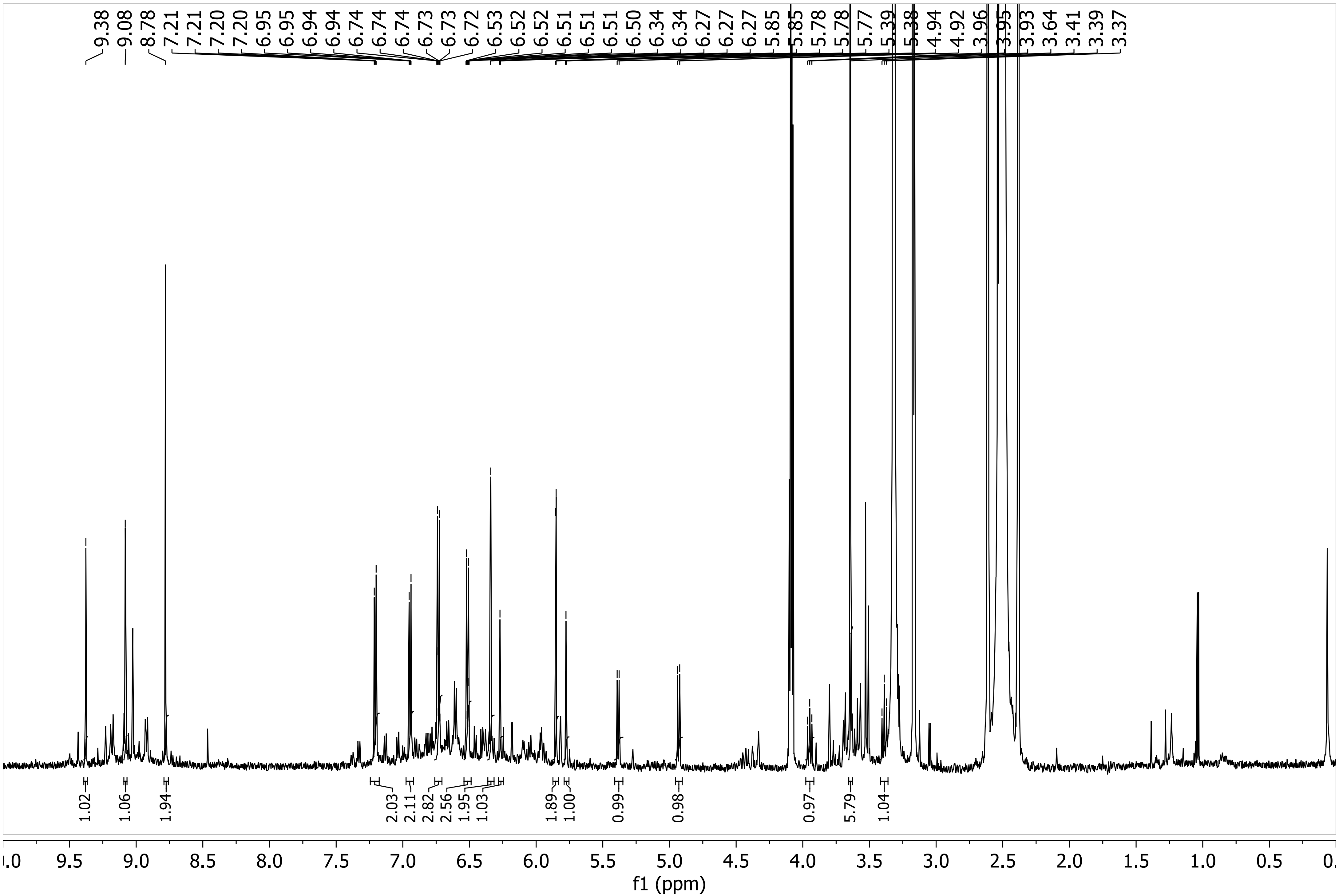

COSY NMR spectrum of compound **7** in DMSO-d_6

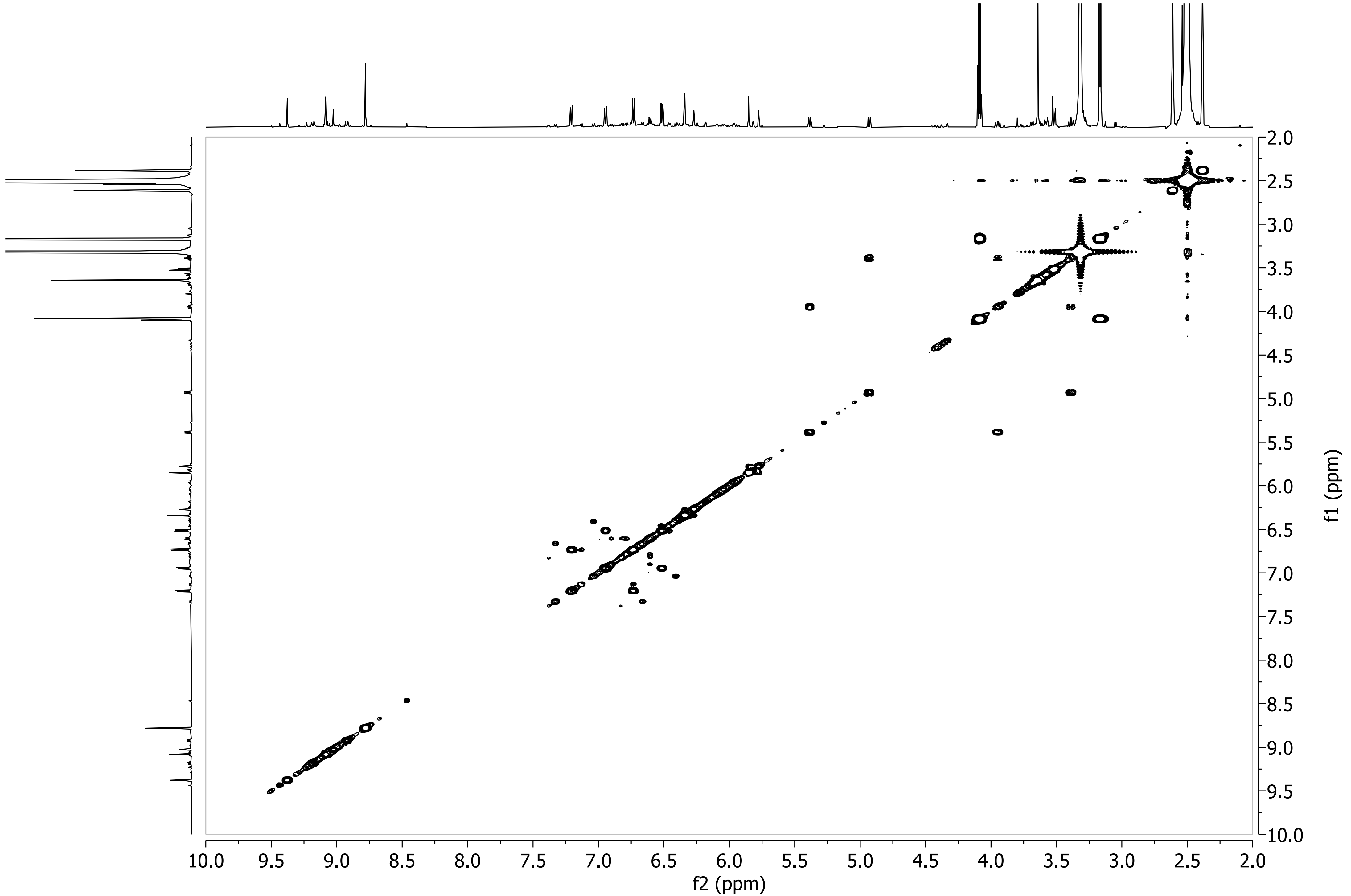

Edited-HSQC NMR spectrum of compound **7** in DMSO-d_6

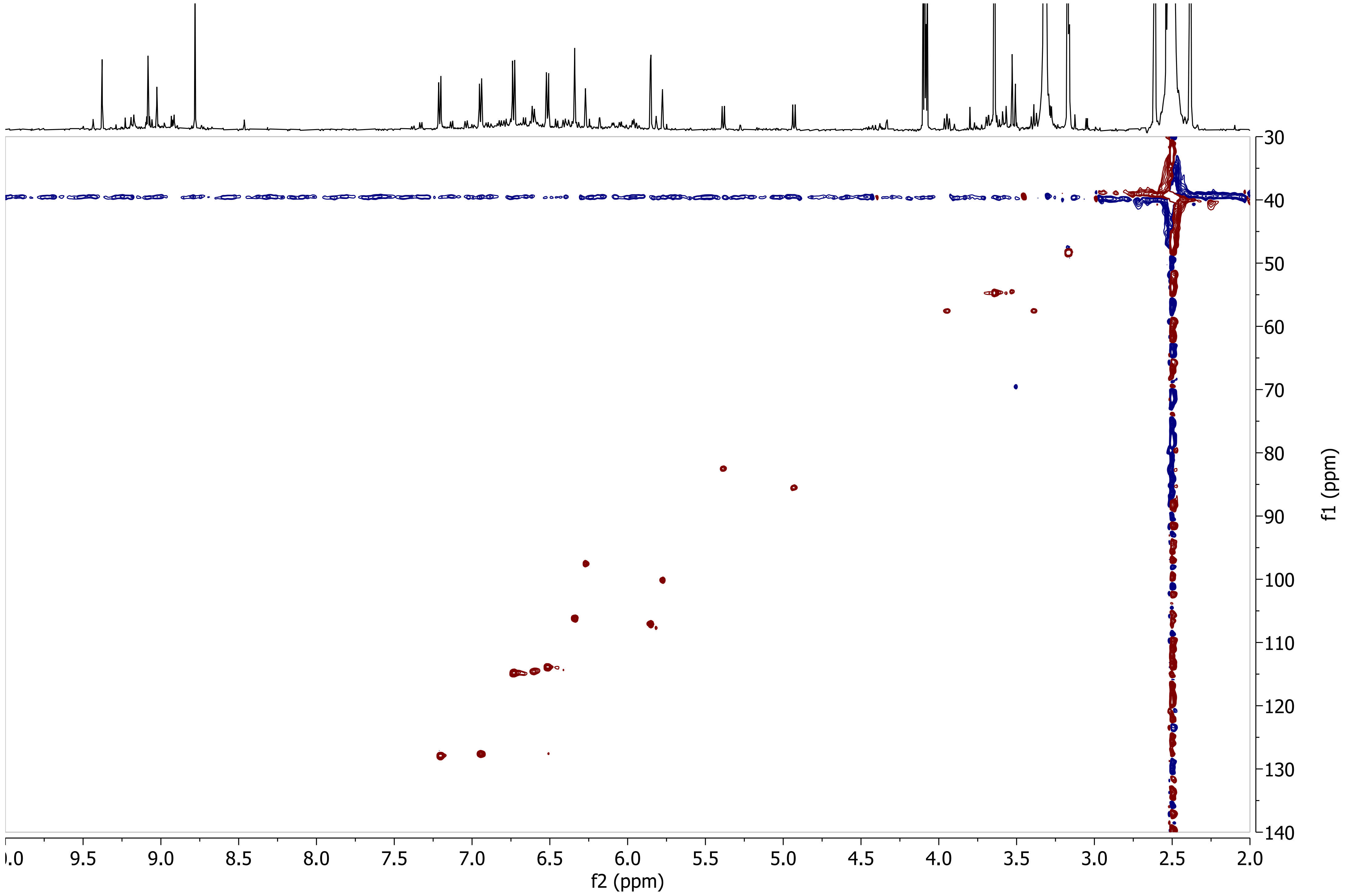

HMBC NMR spectrum of compound **7** in DMSO-d_6

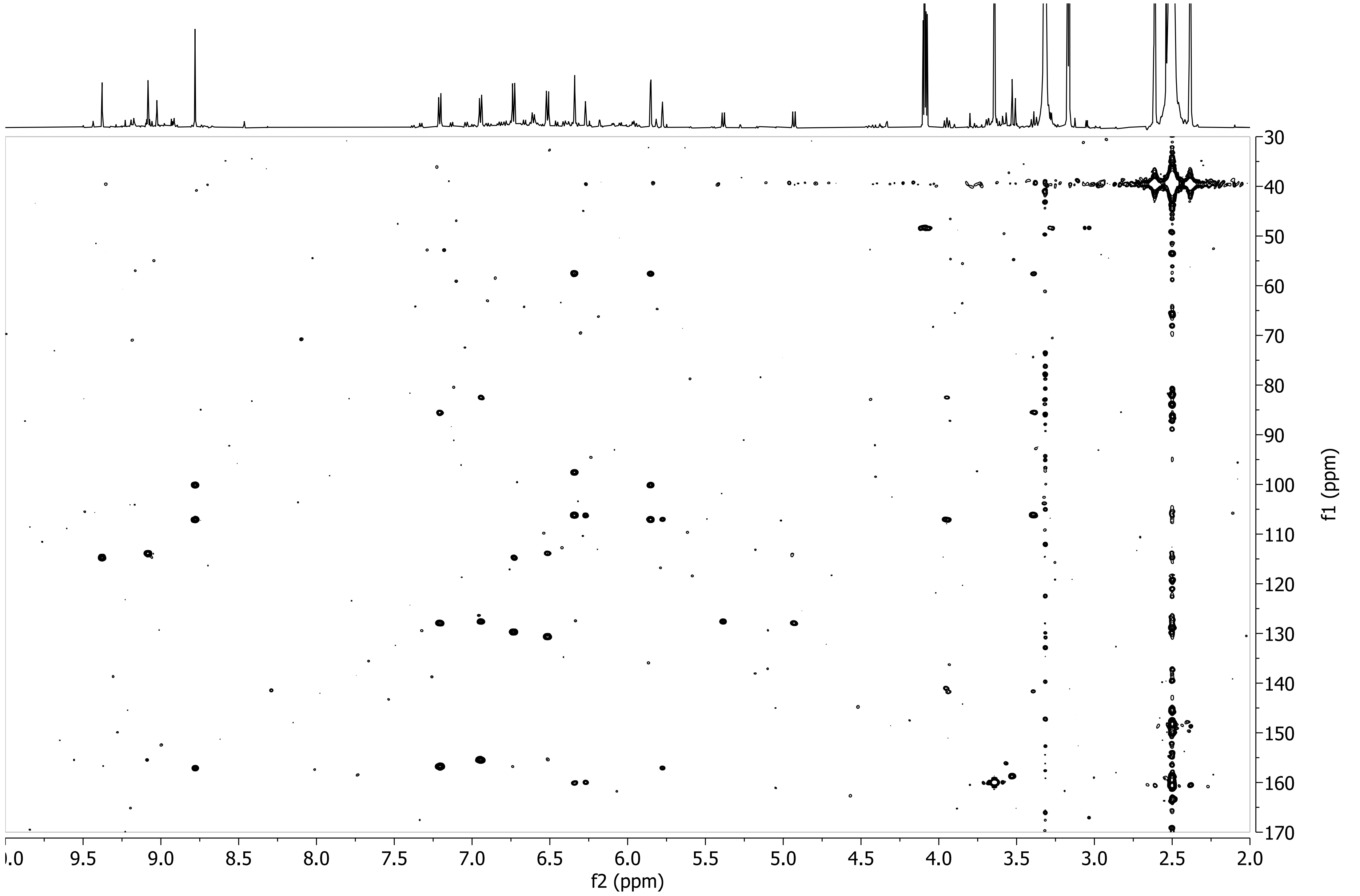

ROESY NMR spectrum of compound **7** in DMSO-d_6

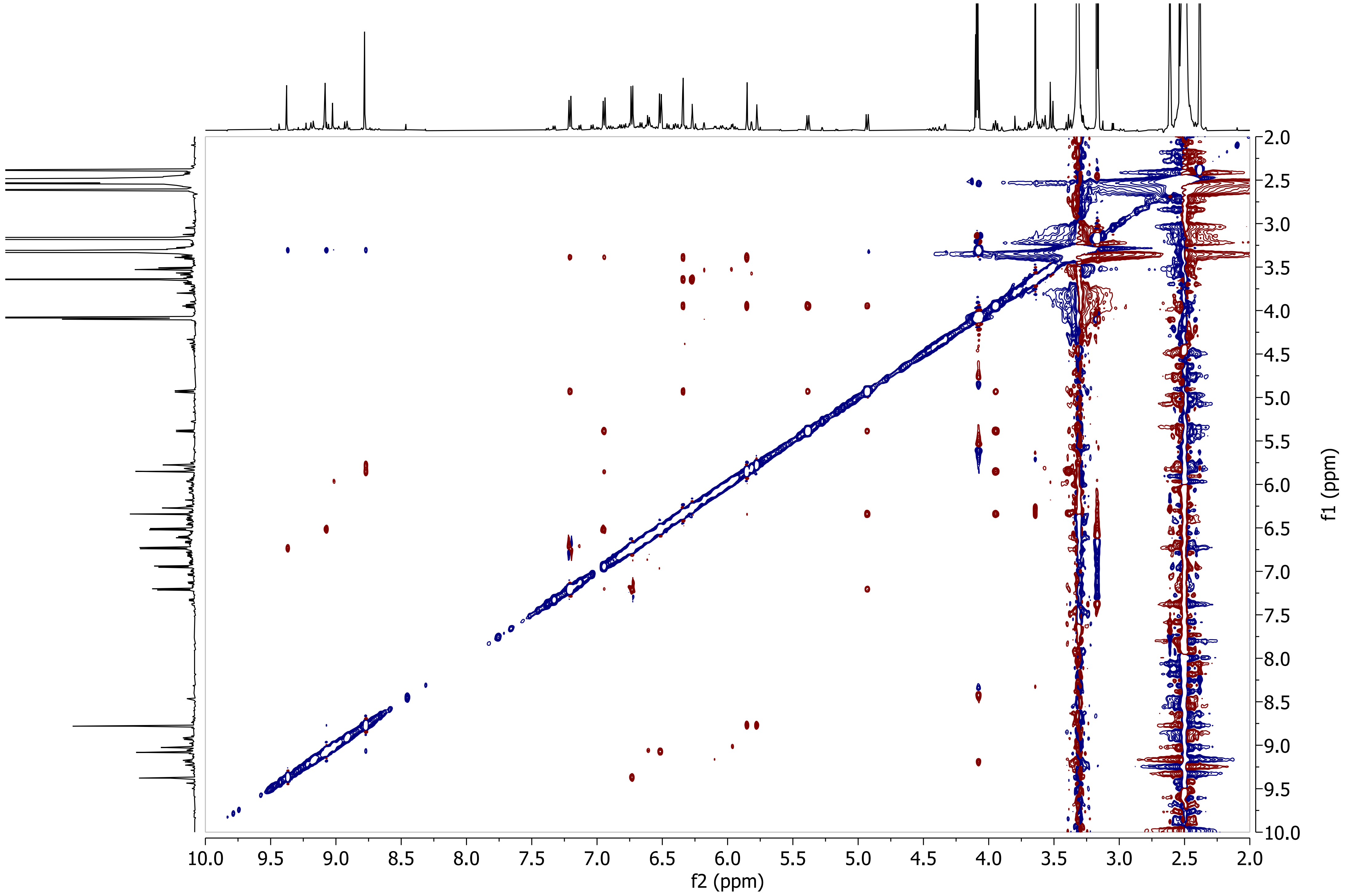

^{1}H NMR spectrum of compound **8** in DMSO-d_6

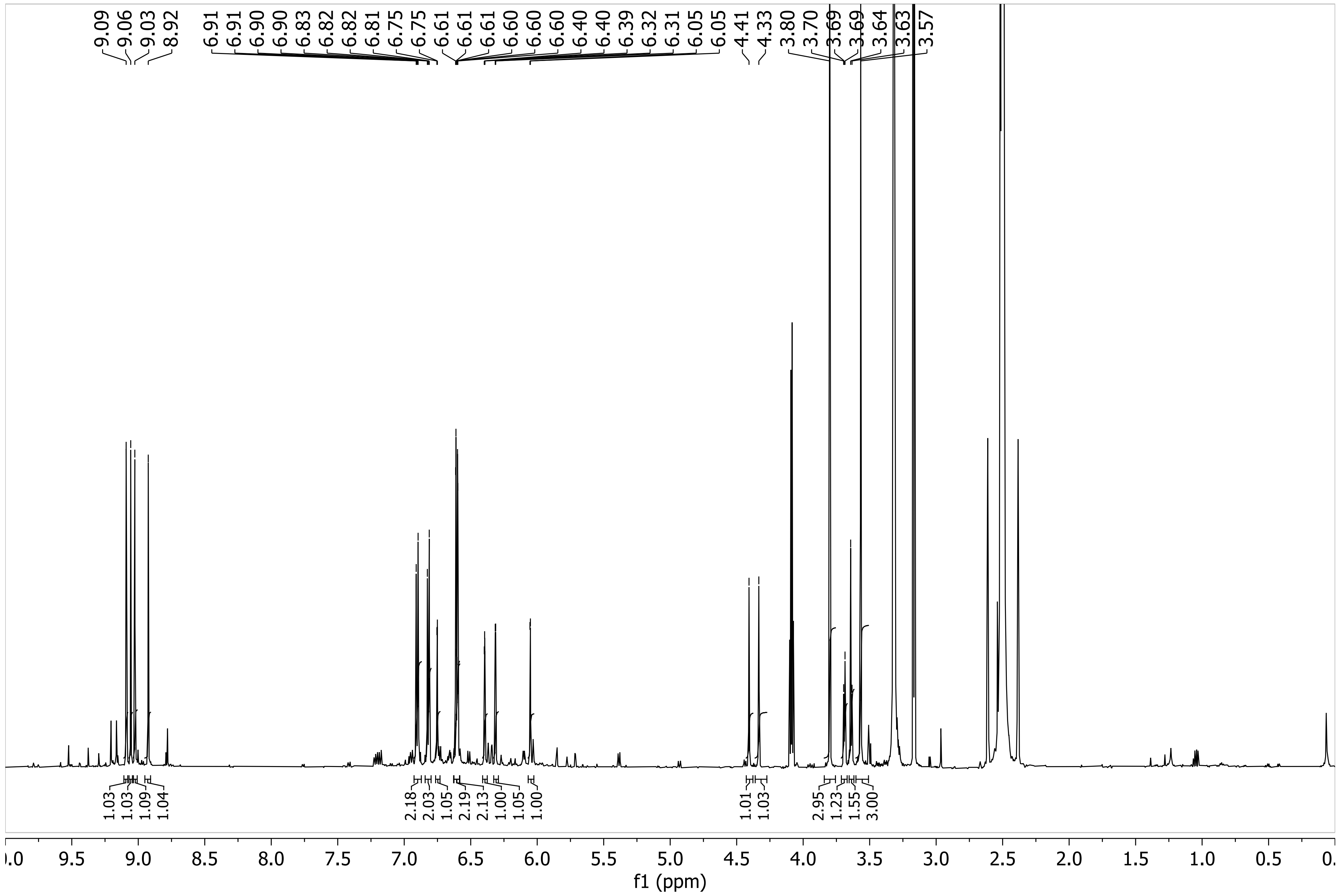

^{1}H NMR spectrum of compound **9** in DMSO-d_6

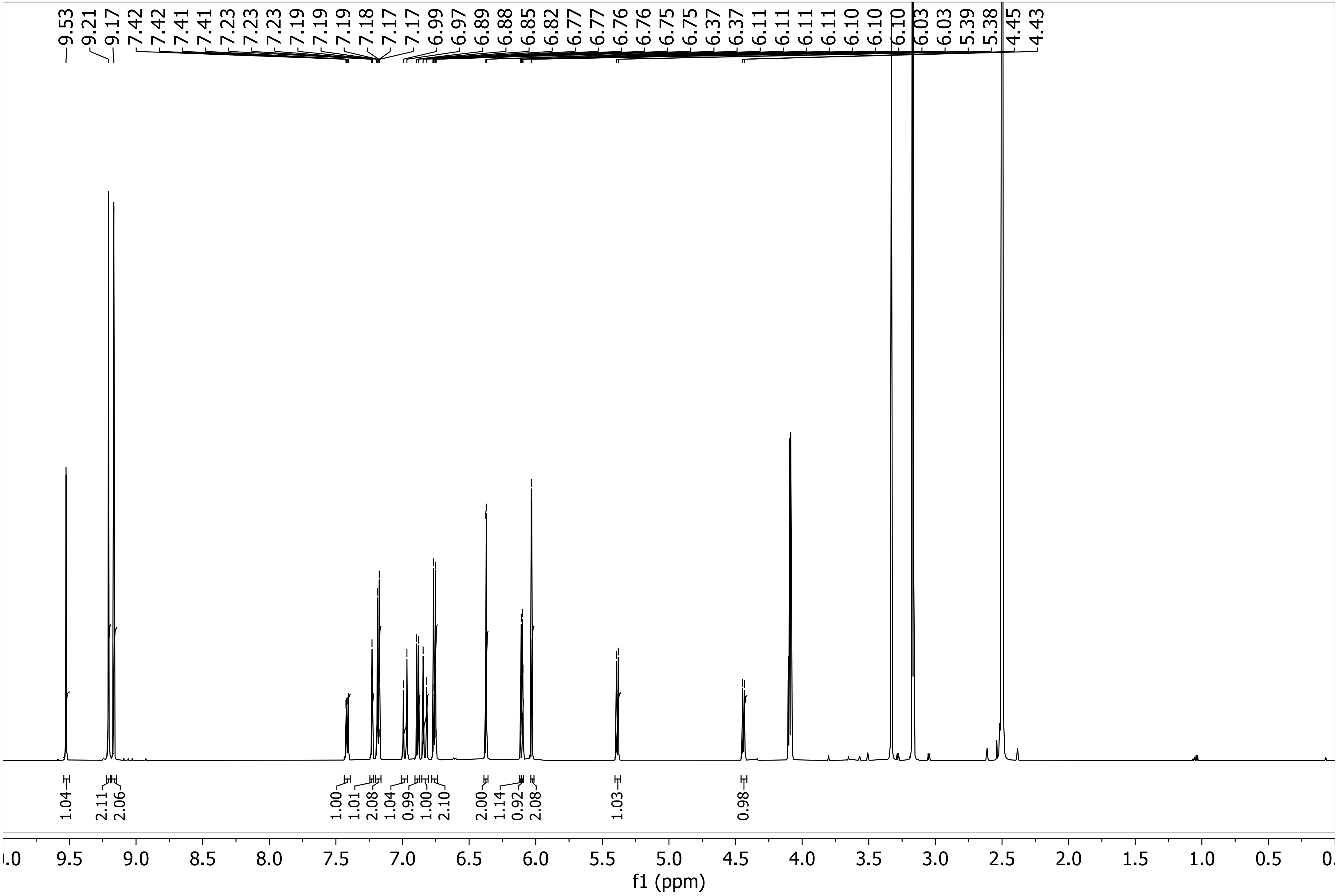

^{1}H NMR spectrum of compound **10** in DMSO-d_6

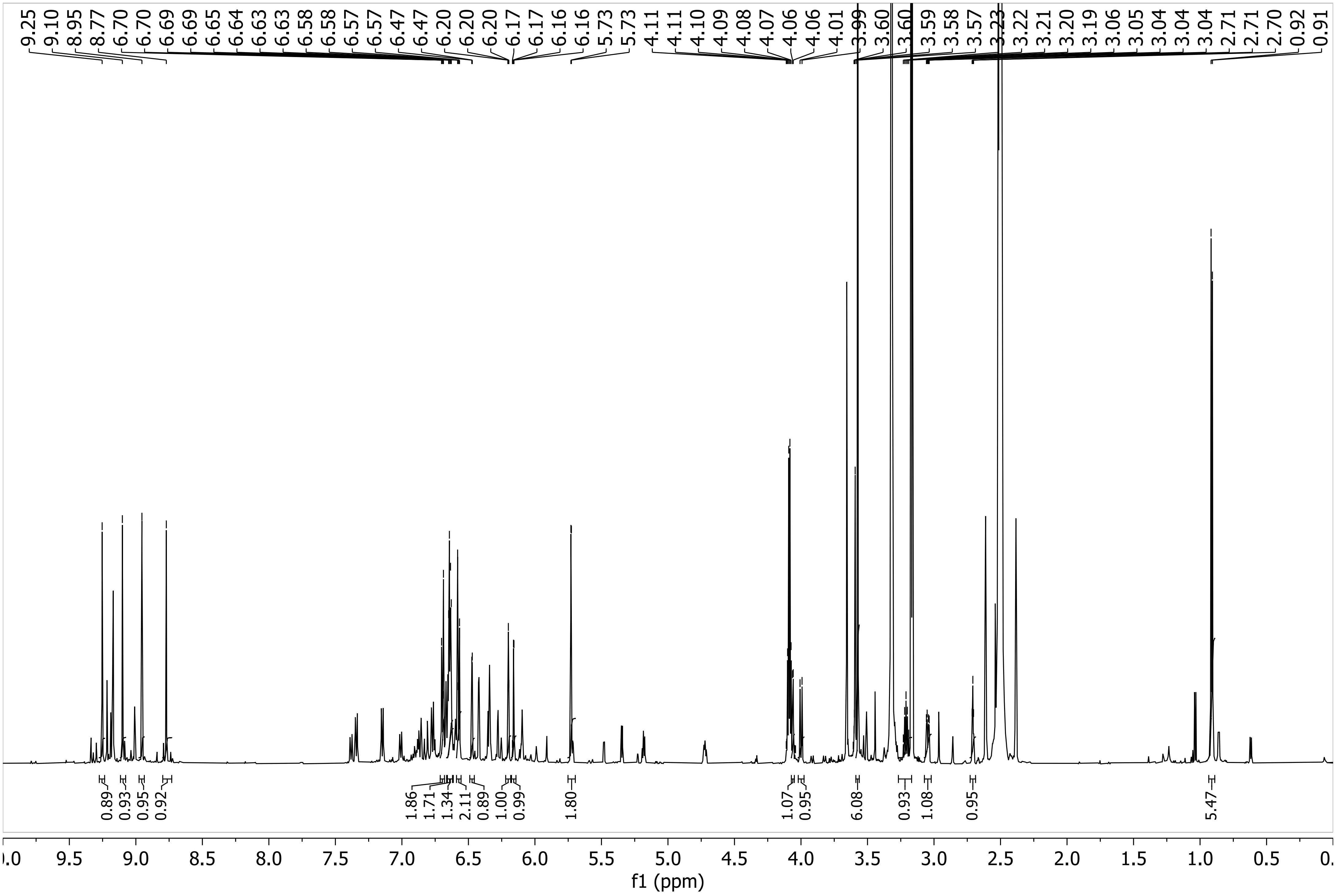

COSY NMR spectrum of compound **10** in DMSO-d_6

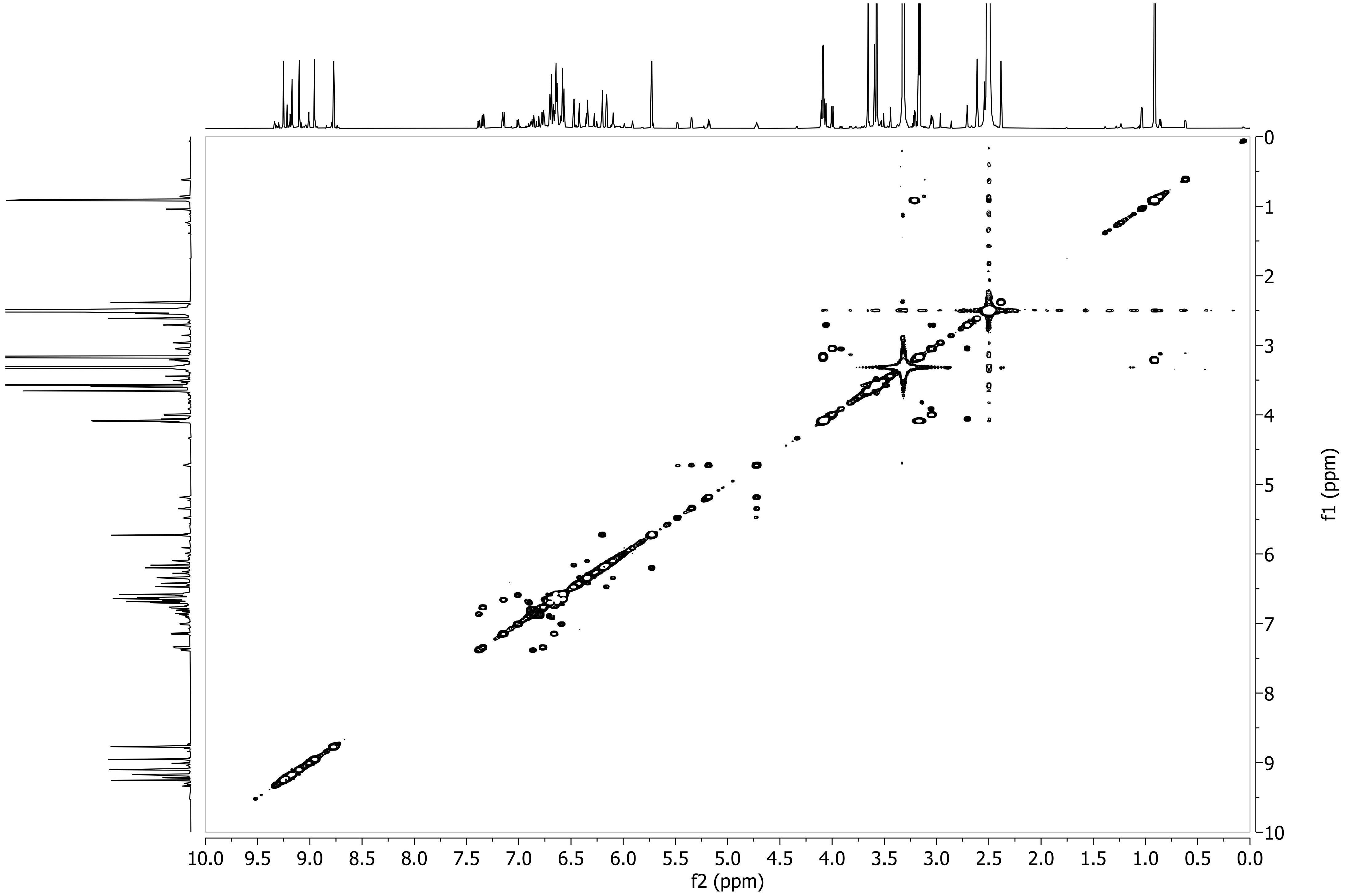

Edited-HSQC NMR spectrum of compound **10** in DMSO-d_6

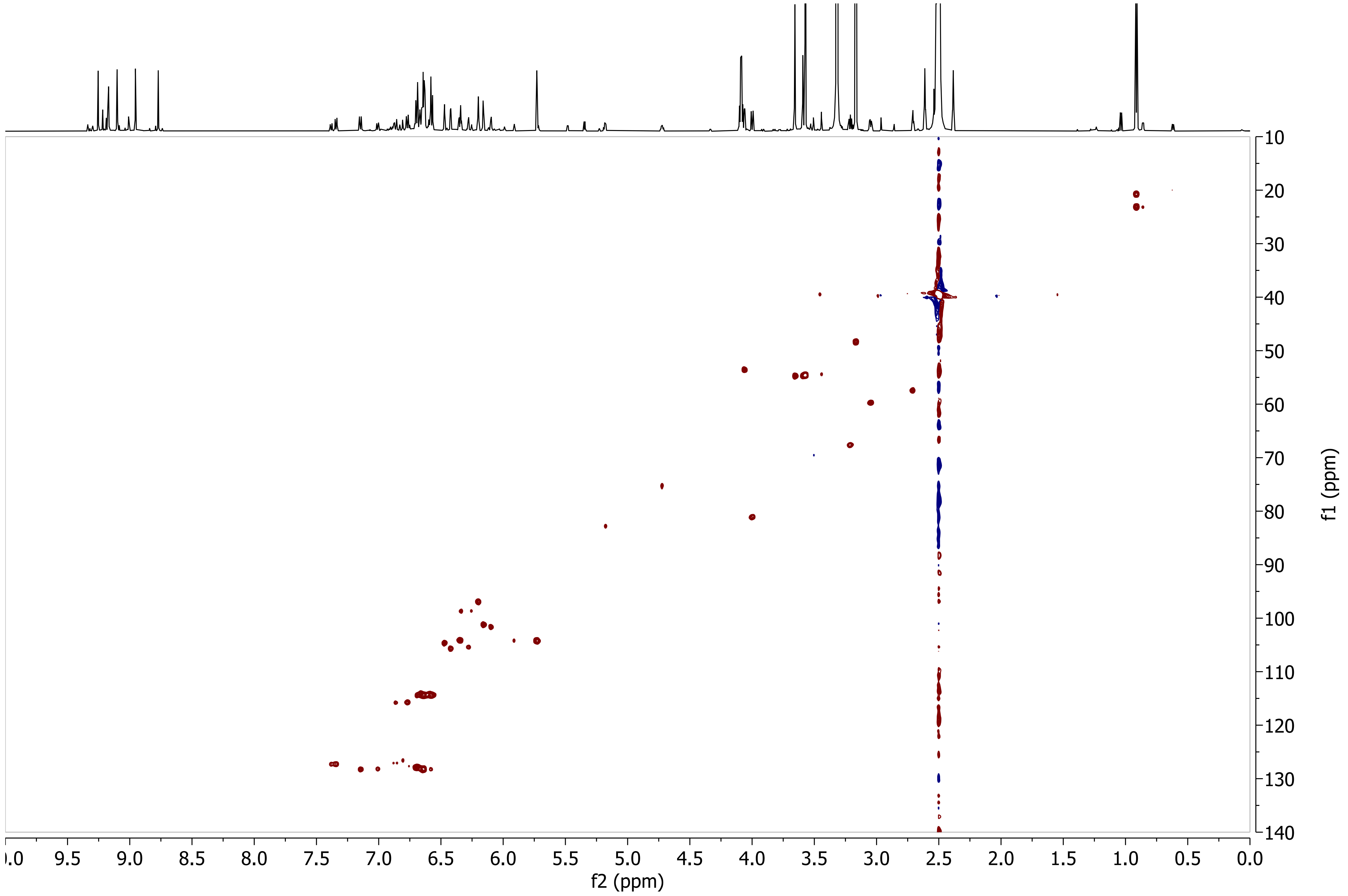

HMBC NMR spectrum of compound **10** in DMSO-d_6

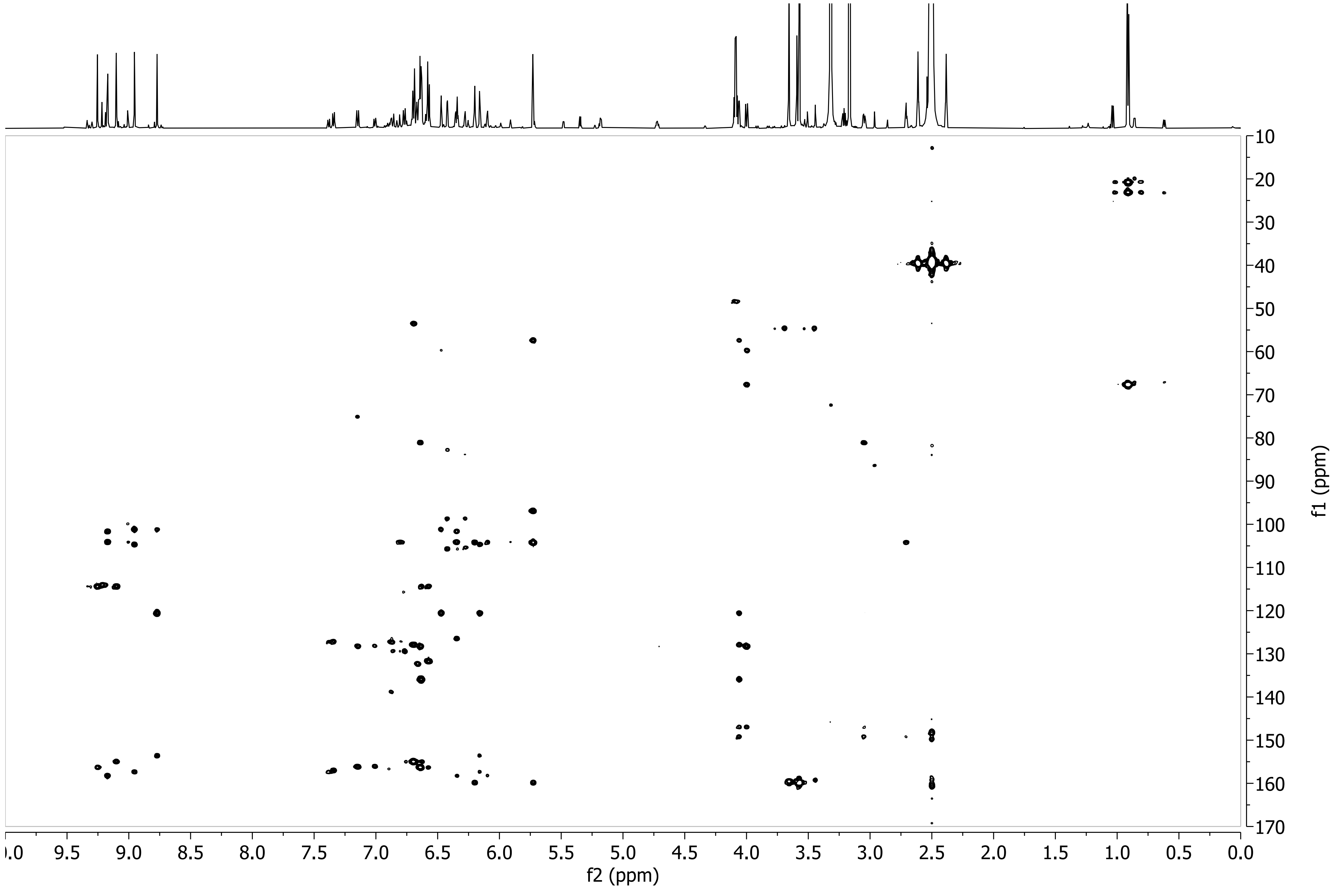

ROESY NMR spectrum of compound **10** in DMSO-d_6

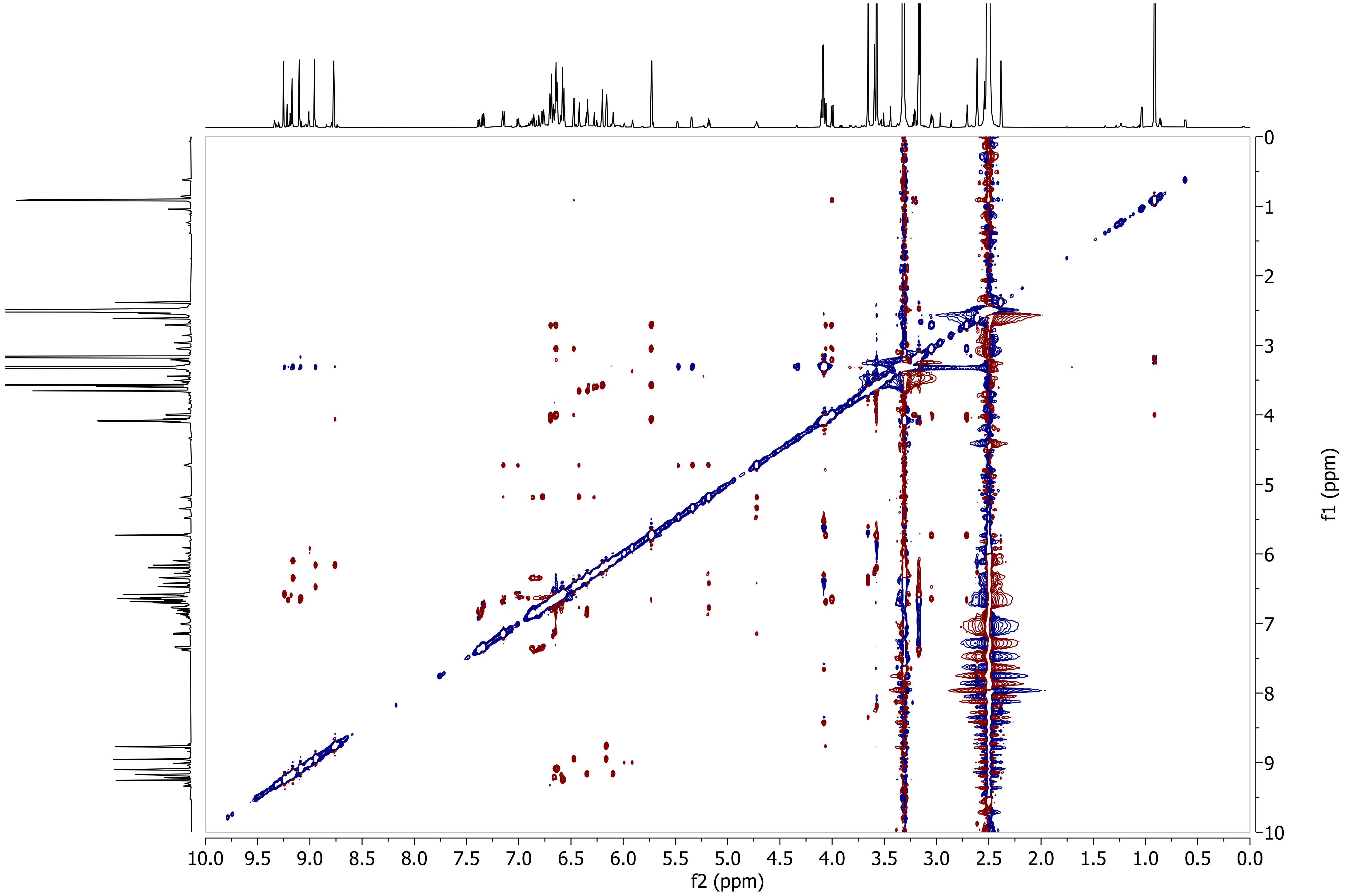

^{1}H NMR spectrum of compound **11** in DMSO-d_6

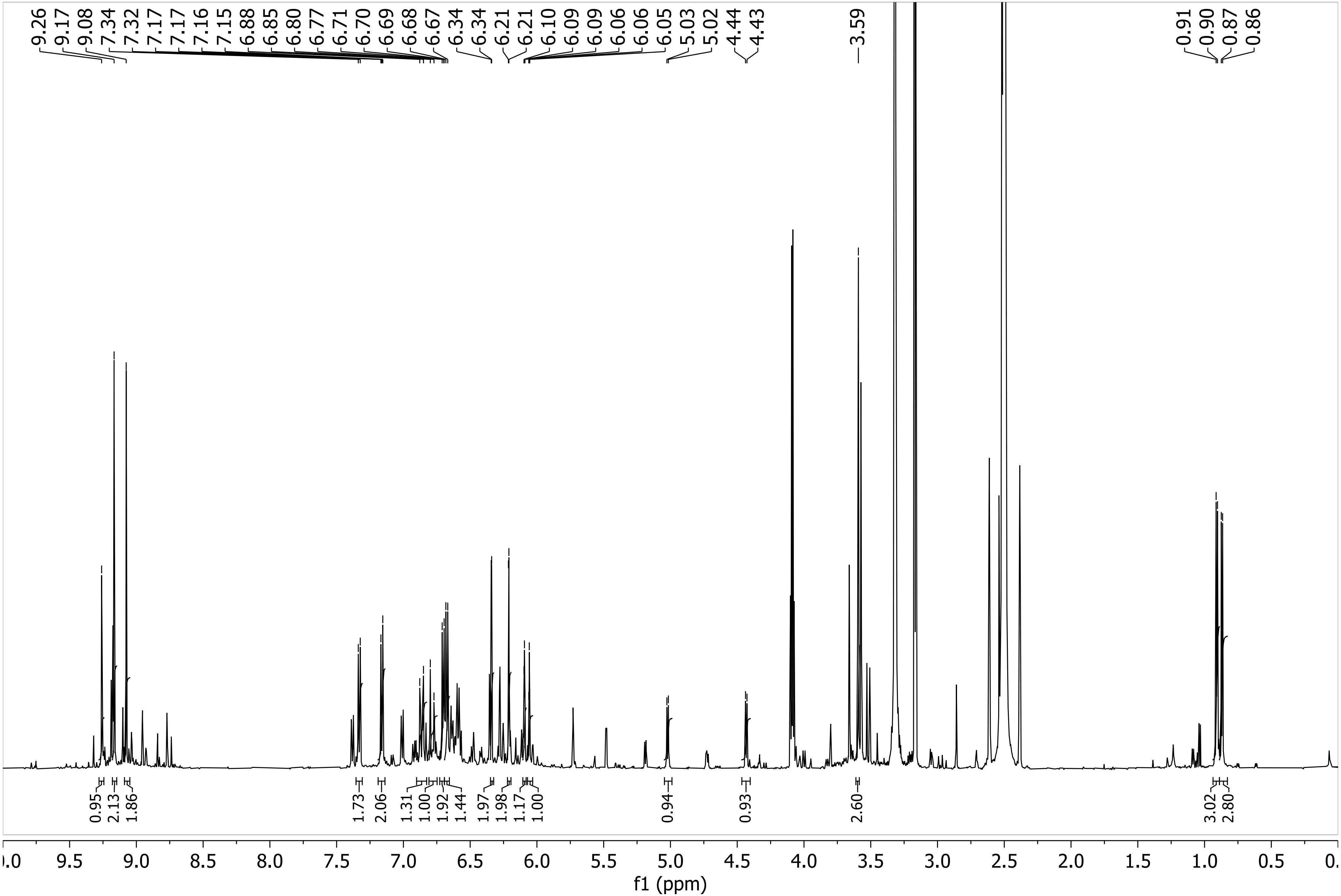

COSY NMR spectrum of compound **11** in DMSO-d_6

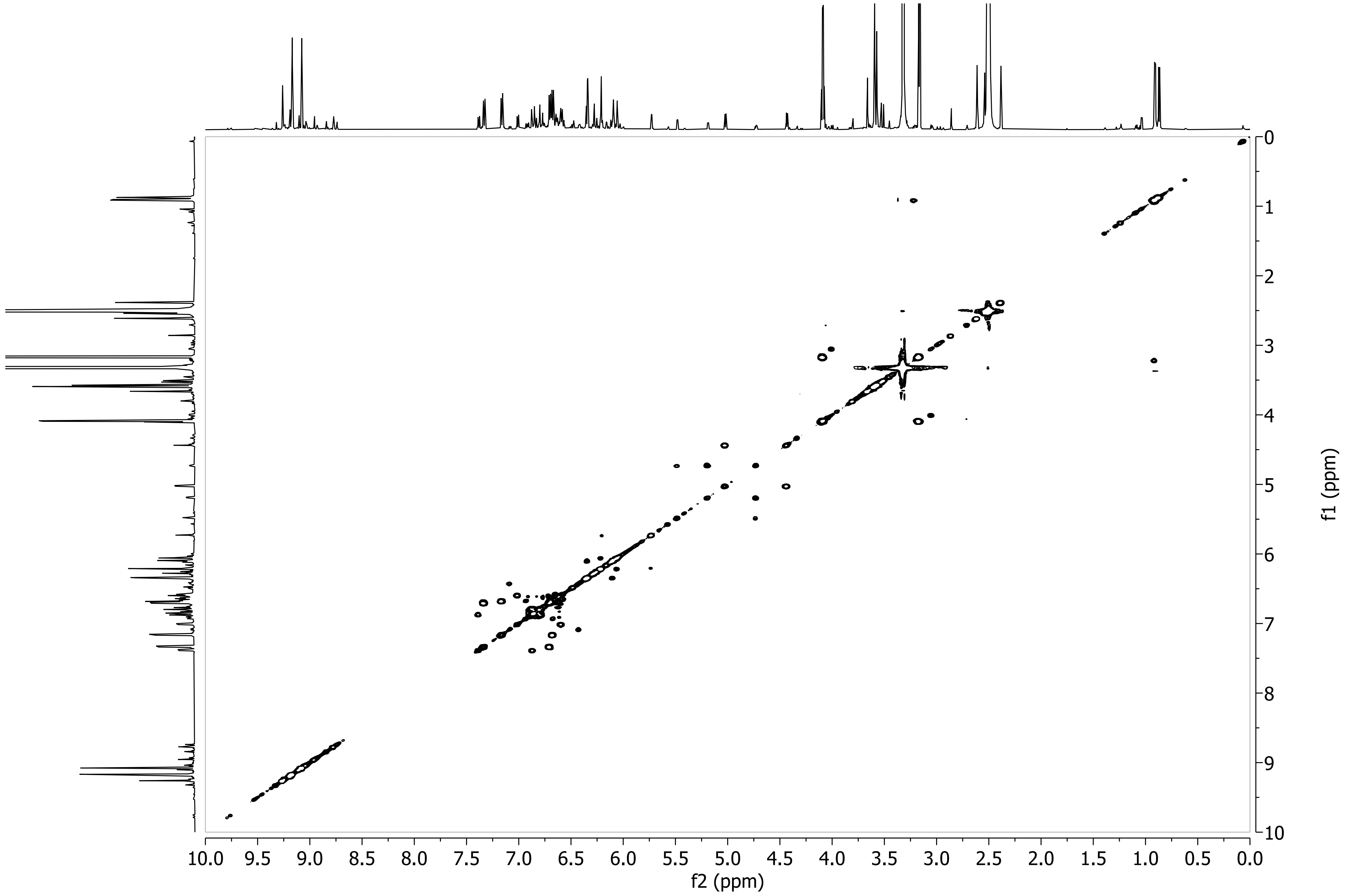

Edited-HSQC NMR spectrum of compound **11** in DMSO-d_6

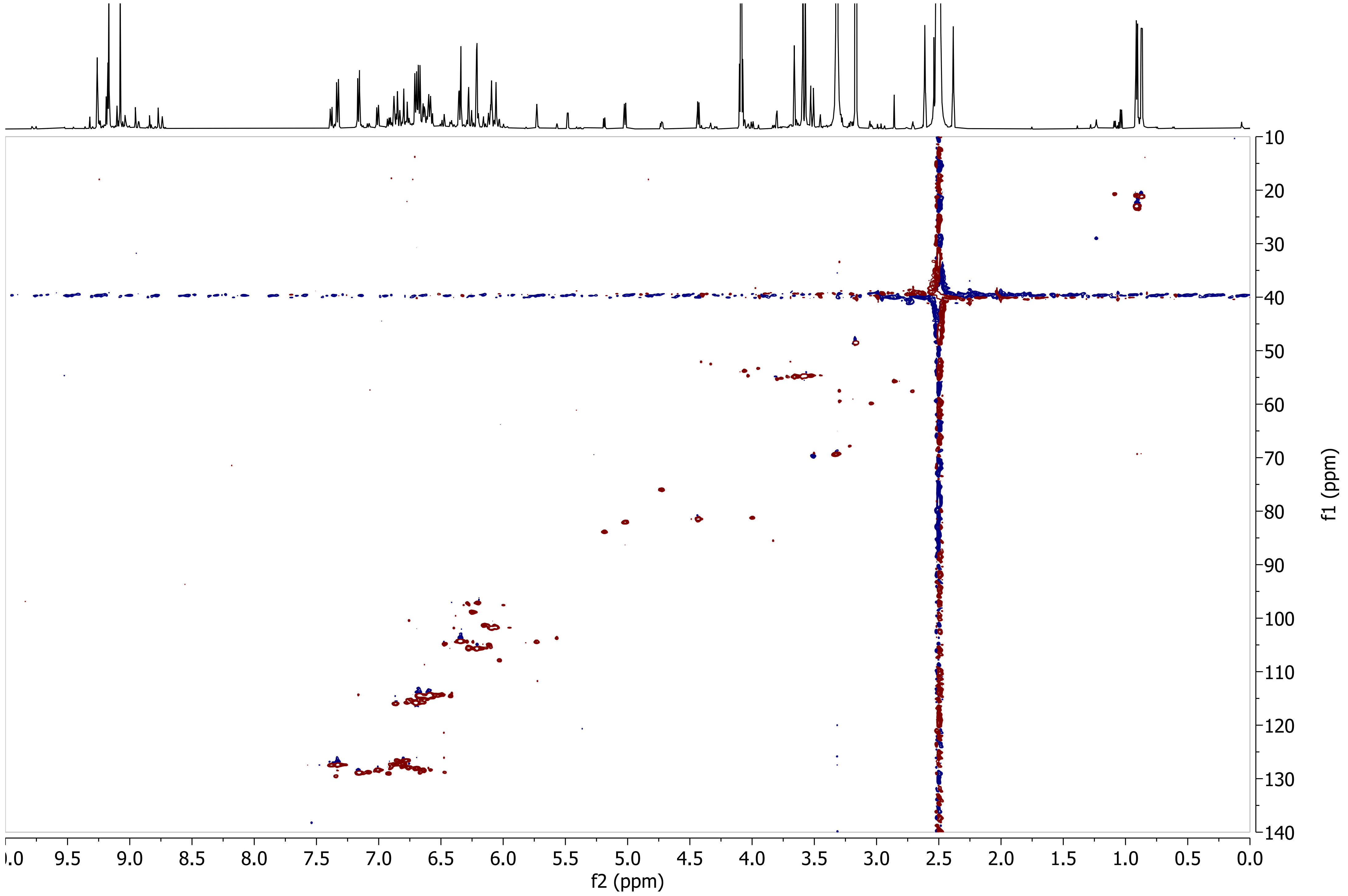

HMBC NMR spectrum of compound **11** in DMSO-d_6

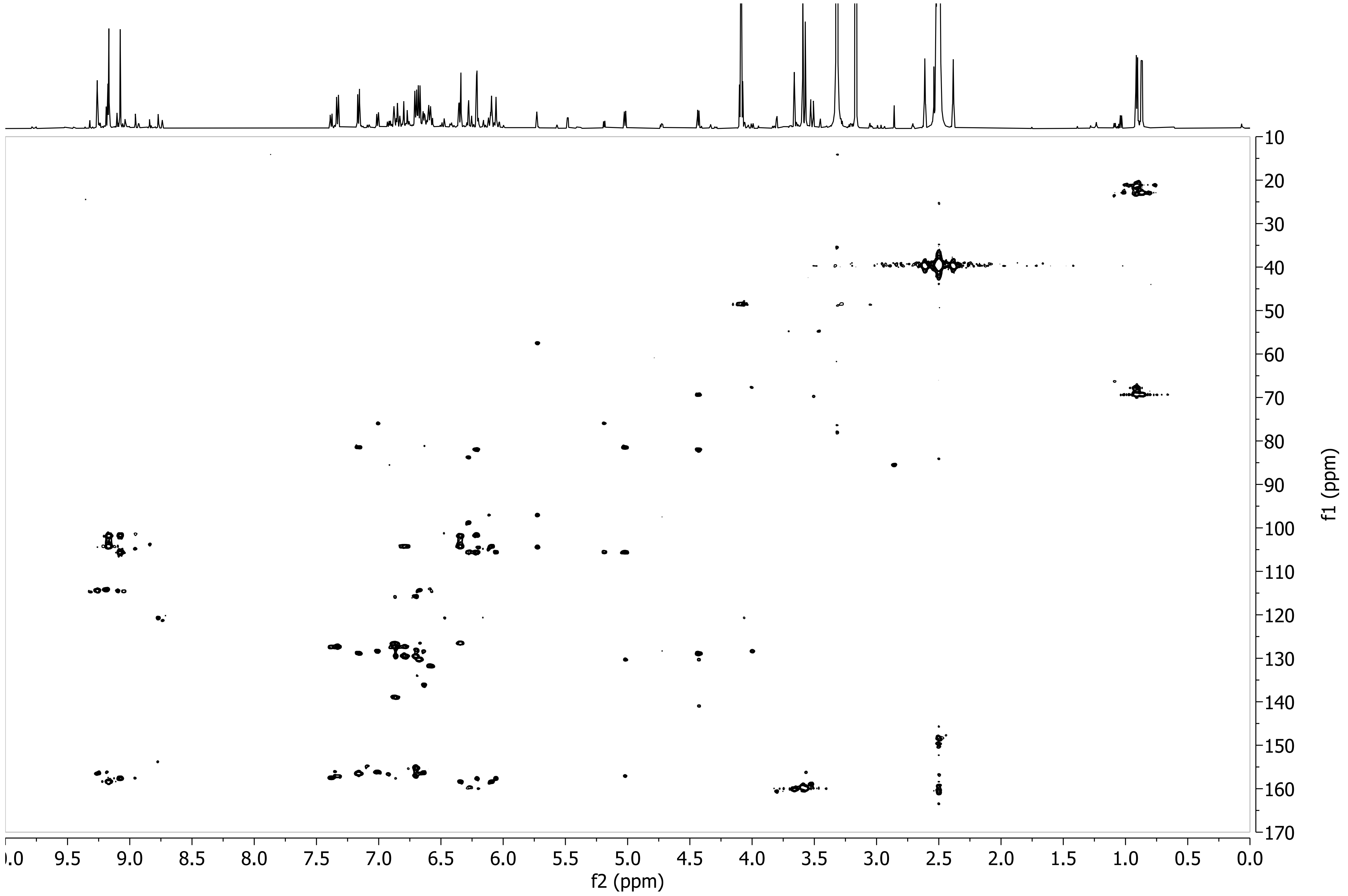

ROESY NMR spectrum of compound **11** in DMSO-d_6

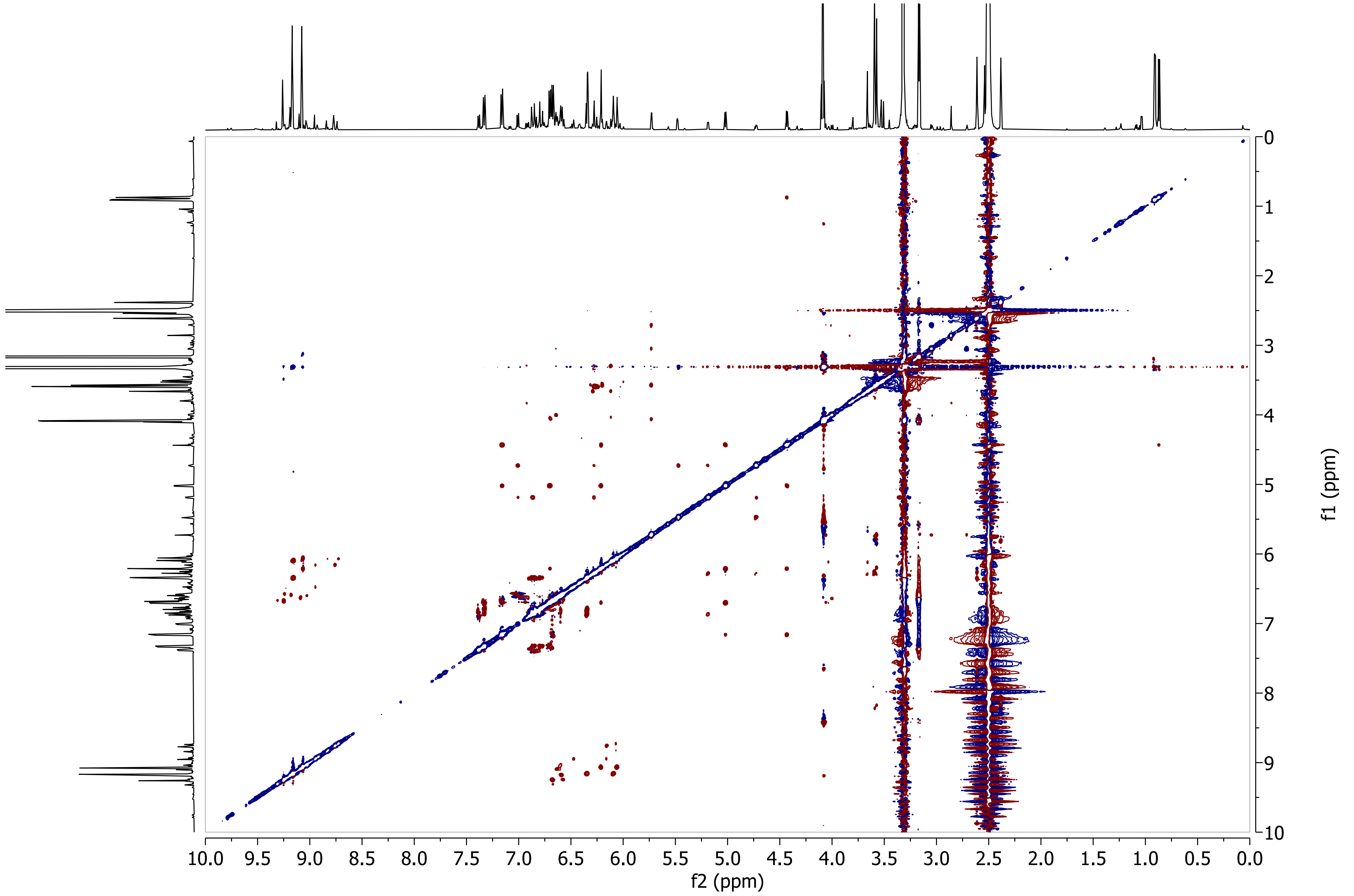

^{1}H NMR spectrum of compound **12** in DMSO-d_6

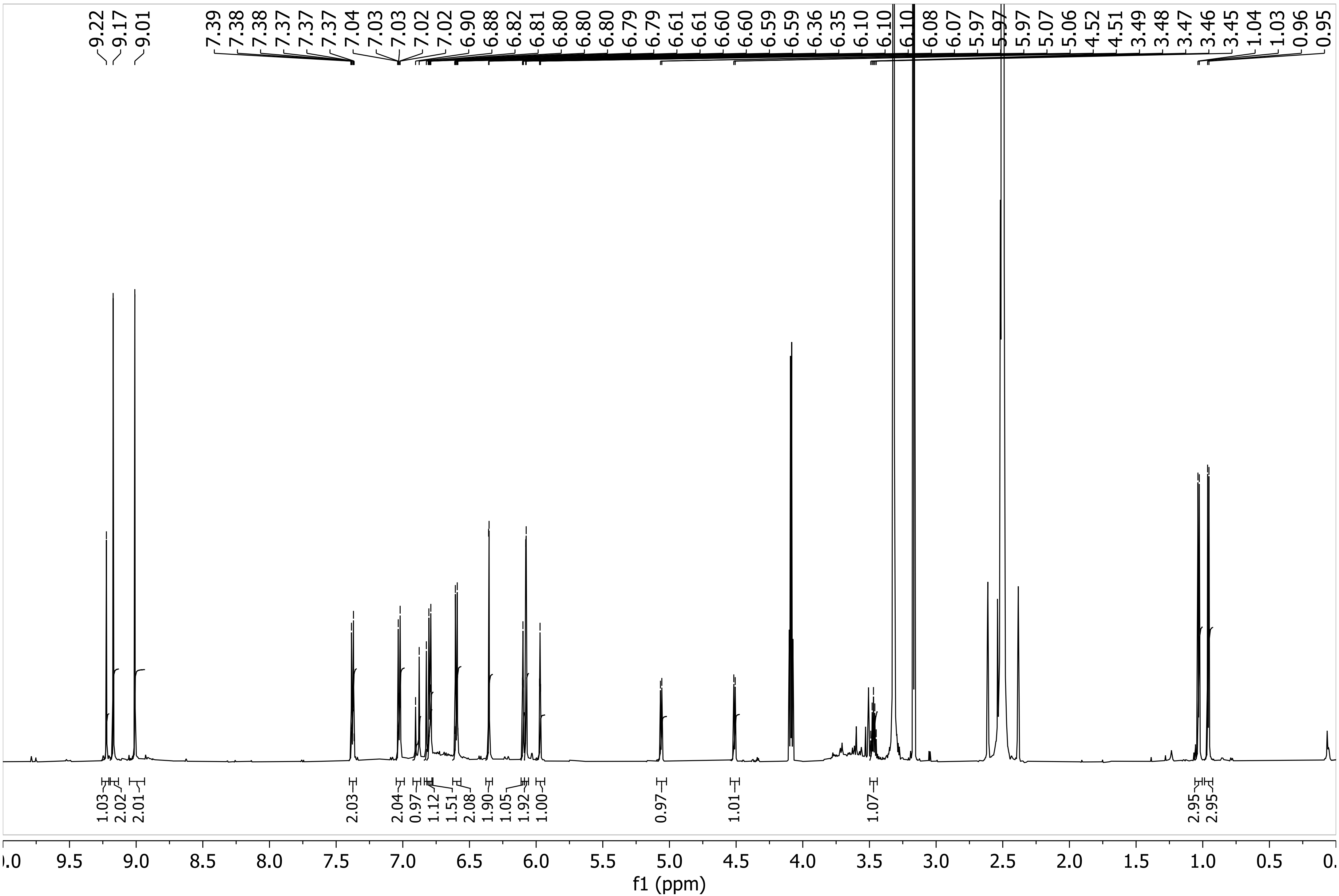

^{1}H NMR spectrum of compound **12** in DMSO-d_6

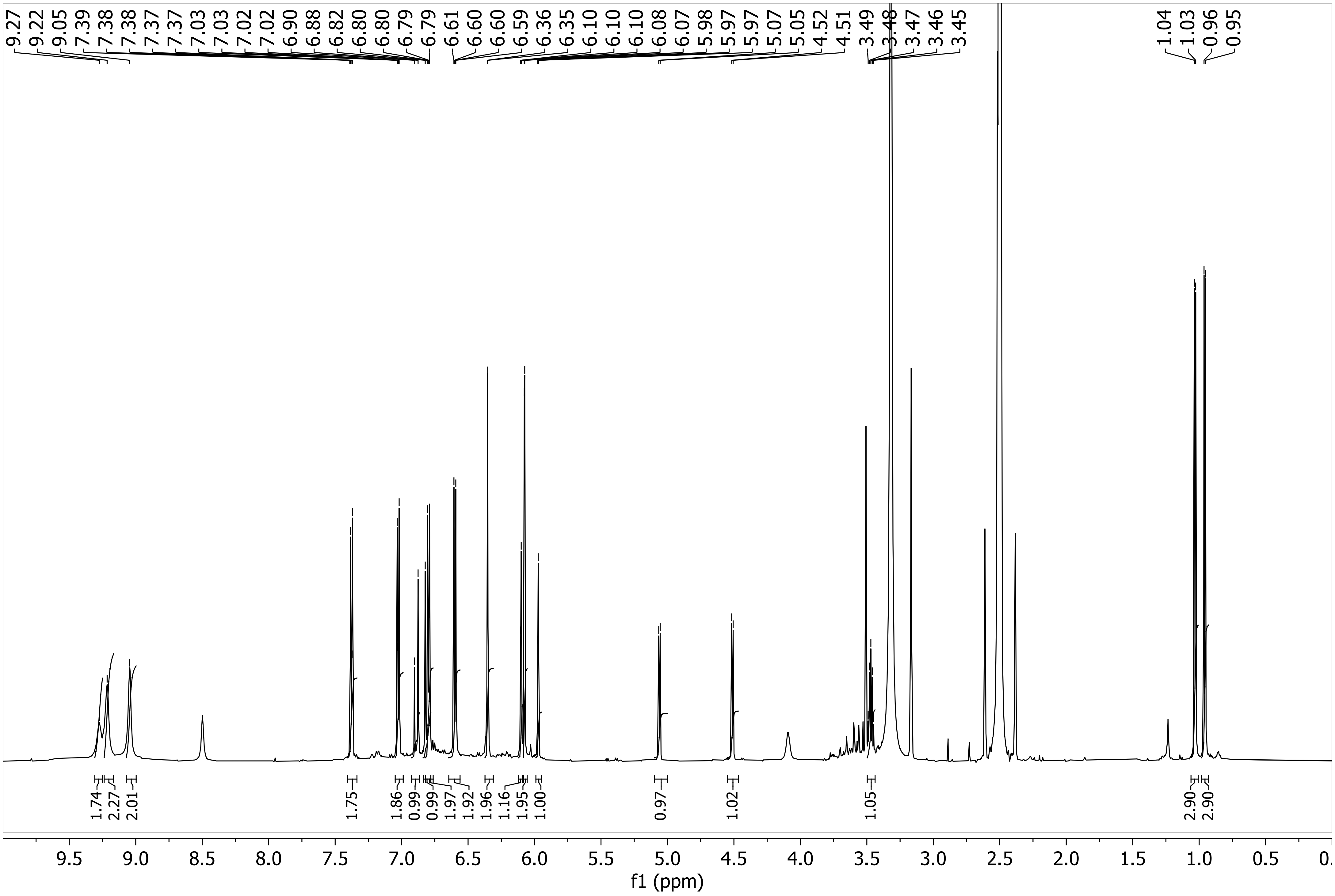

COSY NMR spectrum of compound **12** in DMSO-d_6

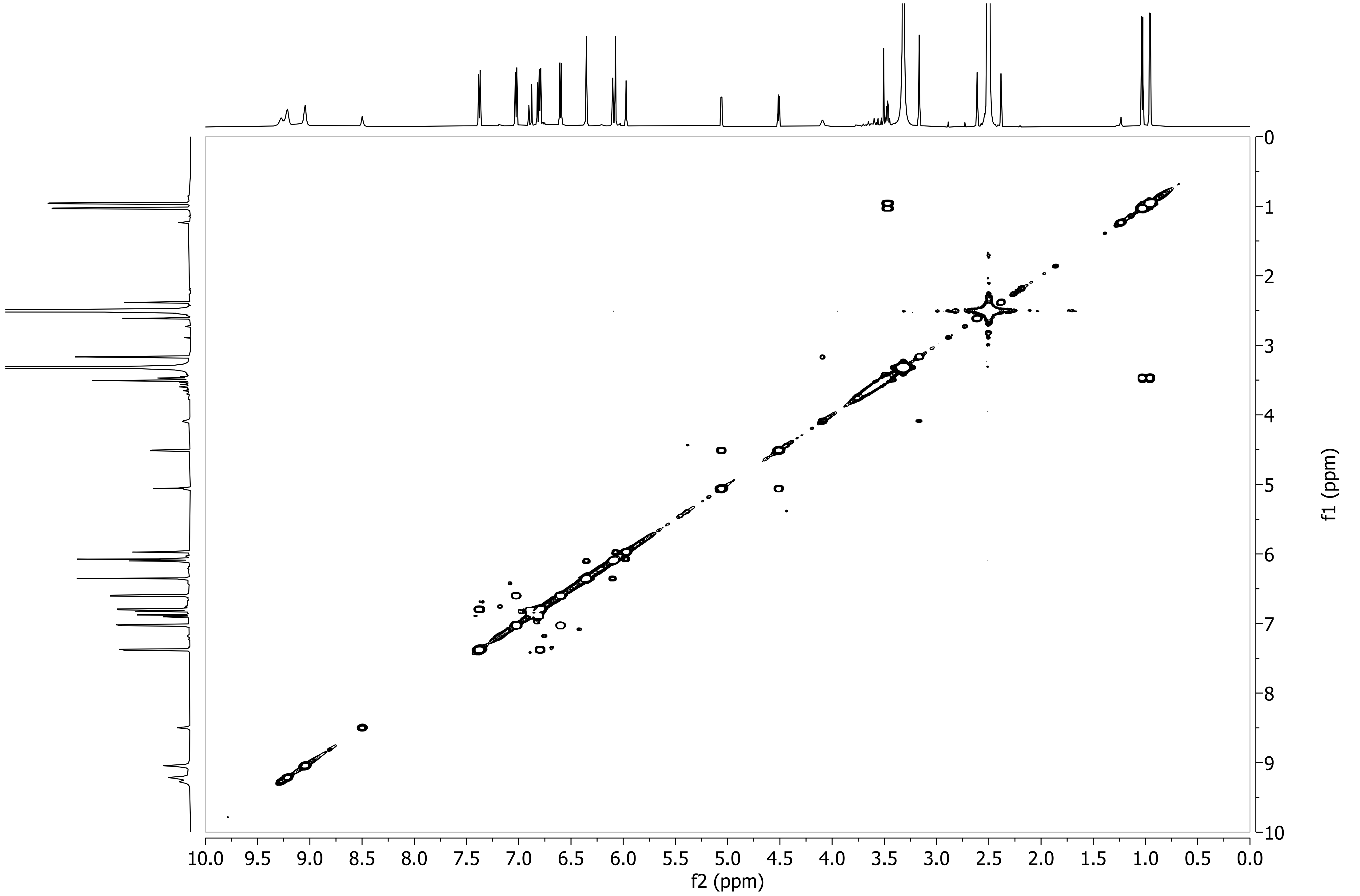

Edited-HSQC NMR spectrum of compound **12** in DMSO-d_6

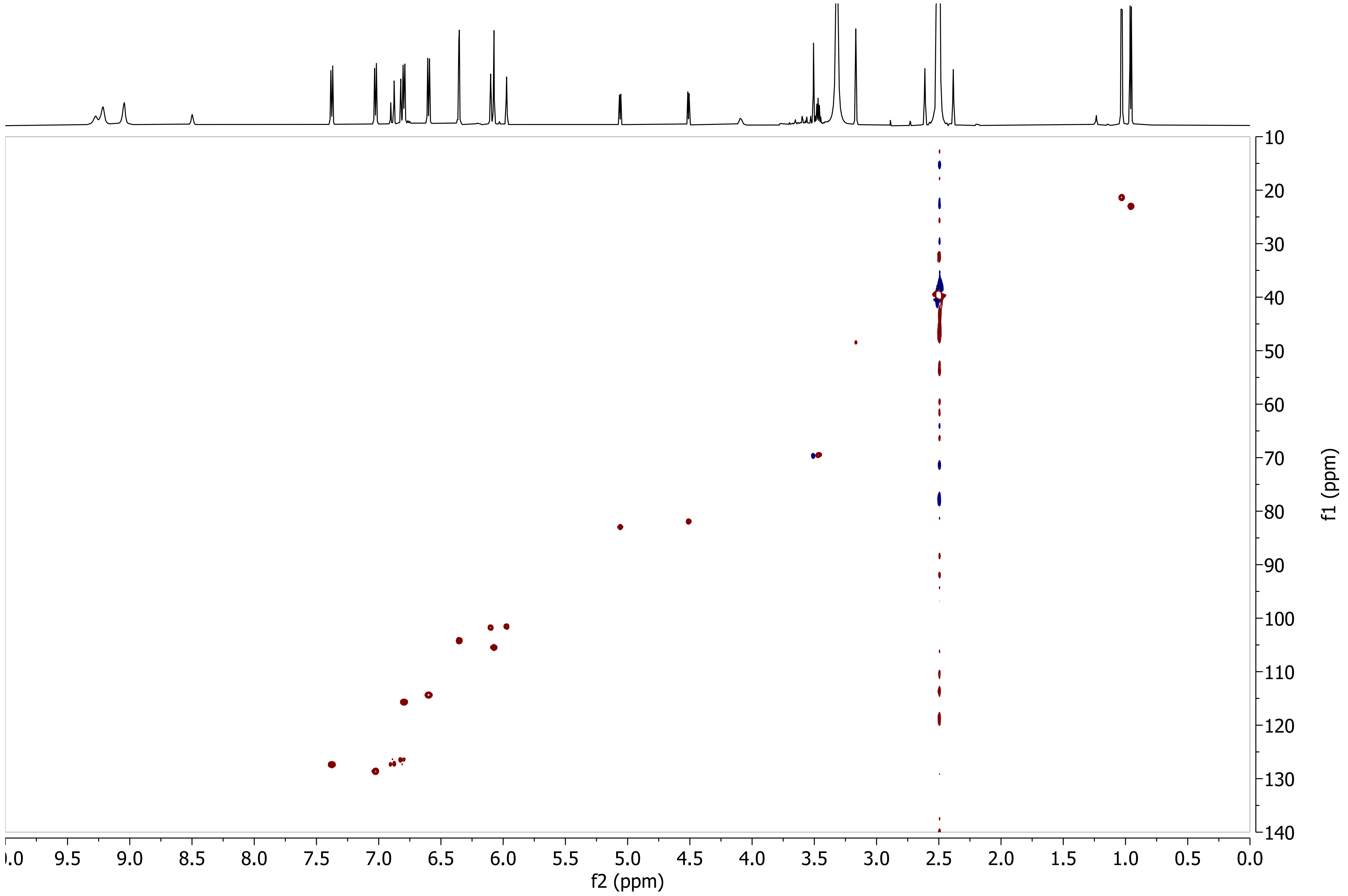

HMBC NMR spectrum of compound **12** in DMSO-d_6

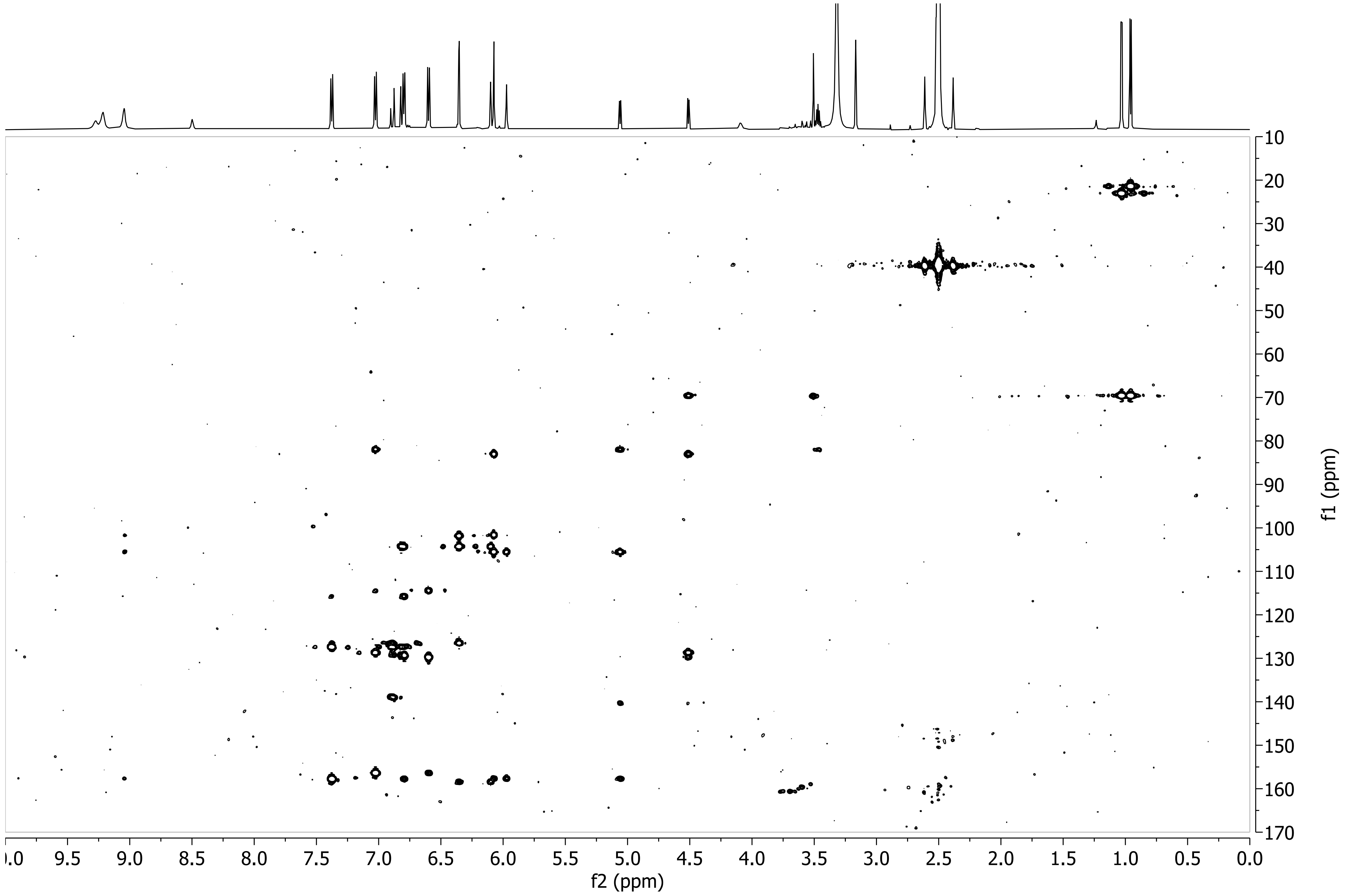

ROESY NMR spectrum of compound **12** in DMSO-d_6

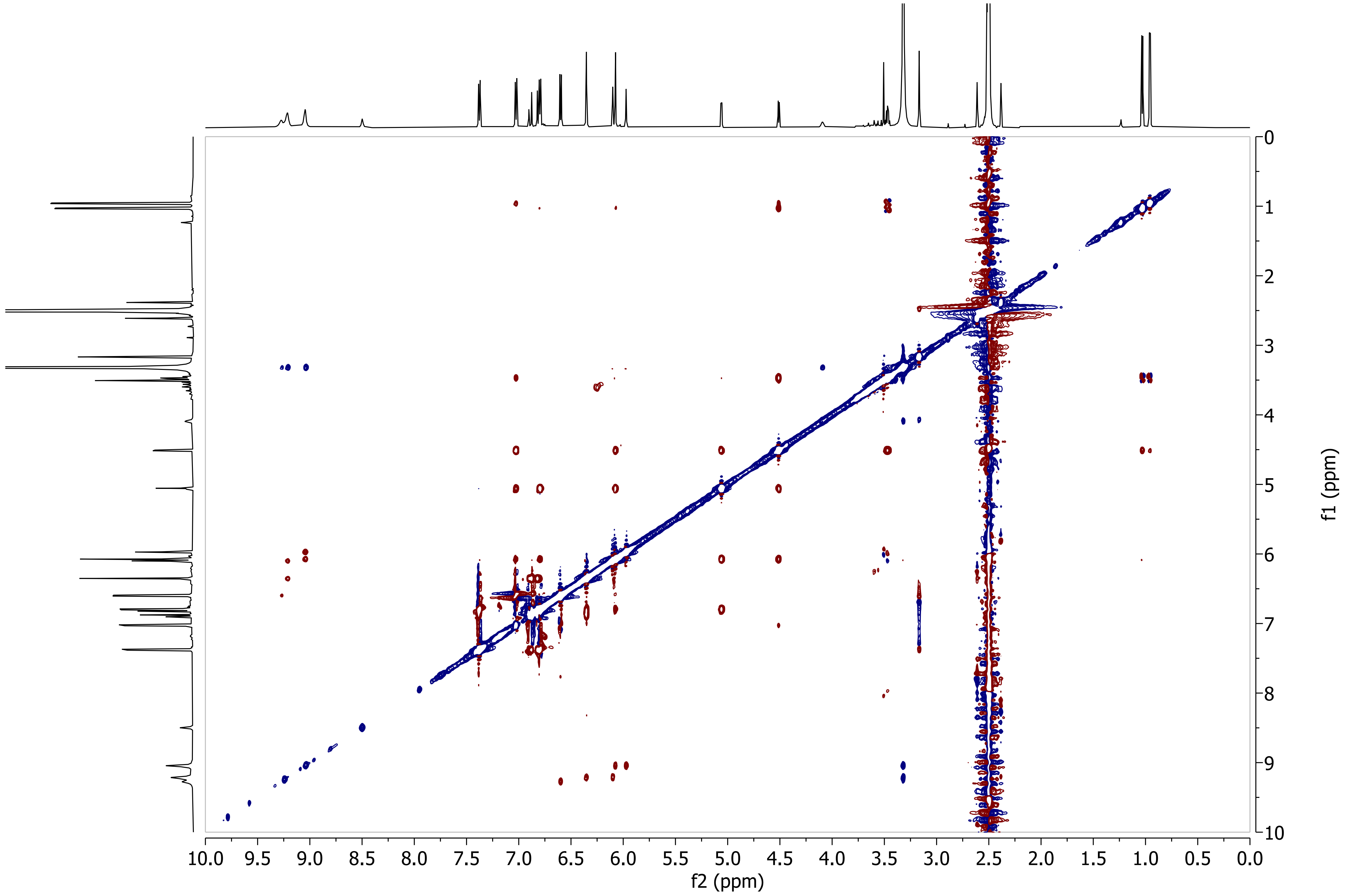

^{1}H NMR spectrum of compound **13** in DMSO-d_6

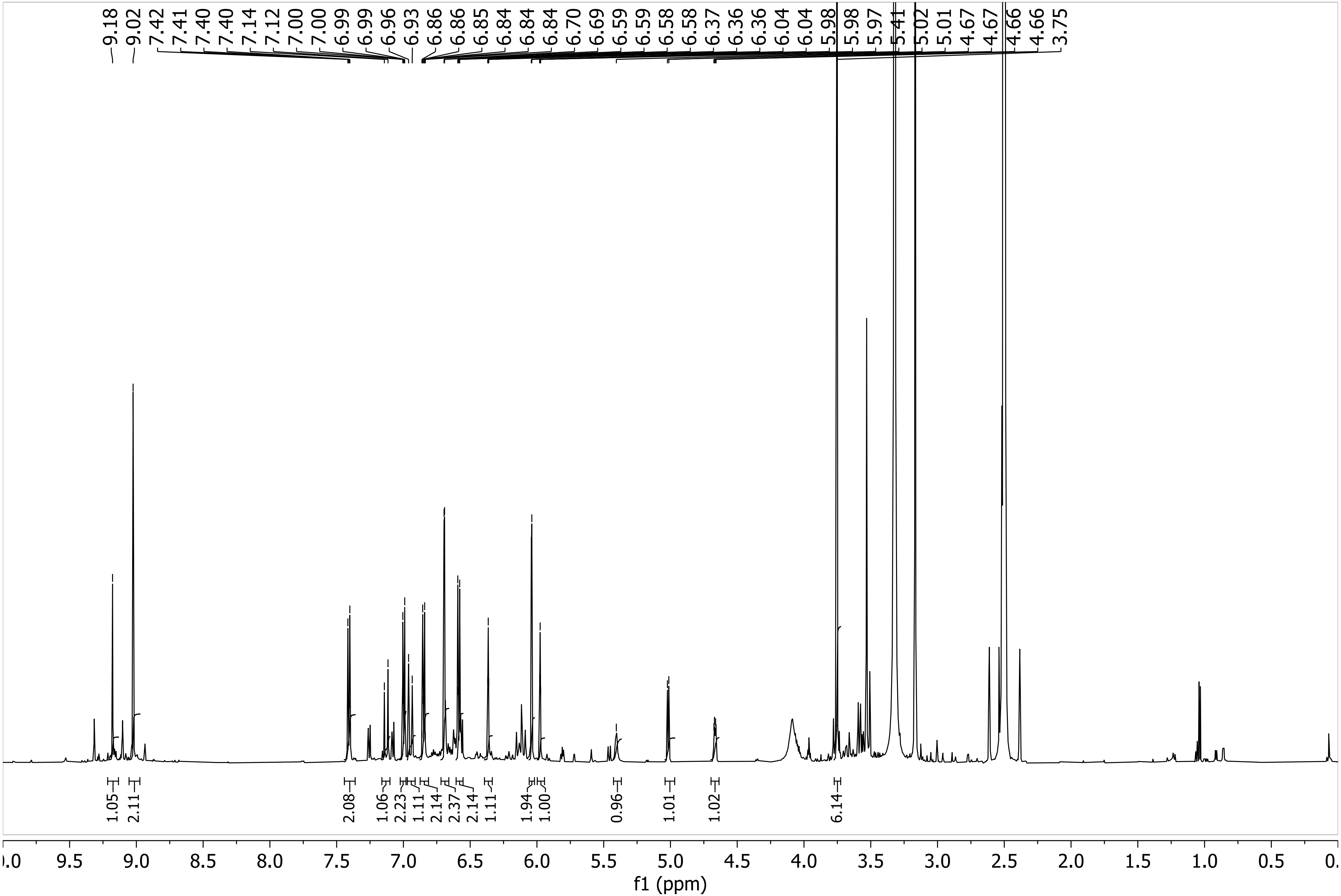

^{1}H NMR spectrum of compound **13** in DMSO-d_6

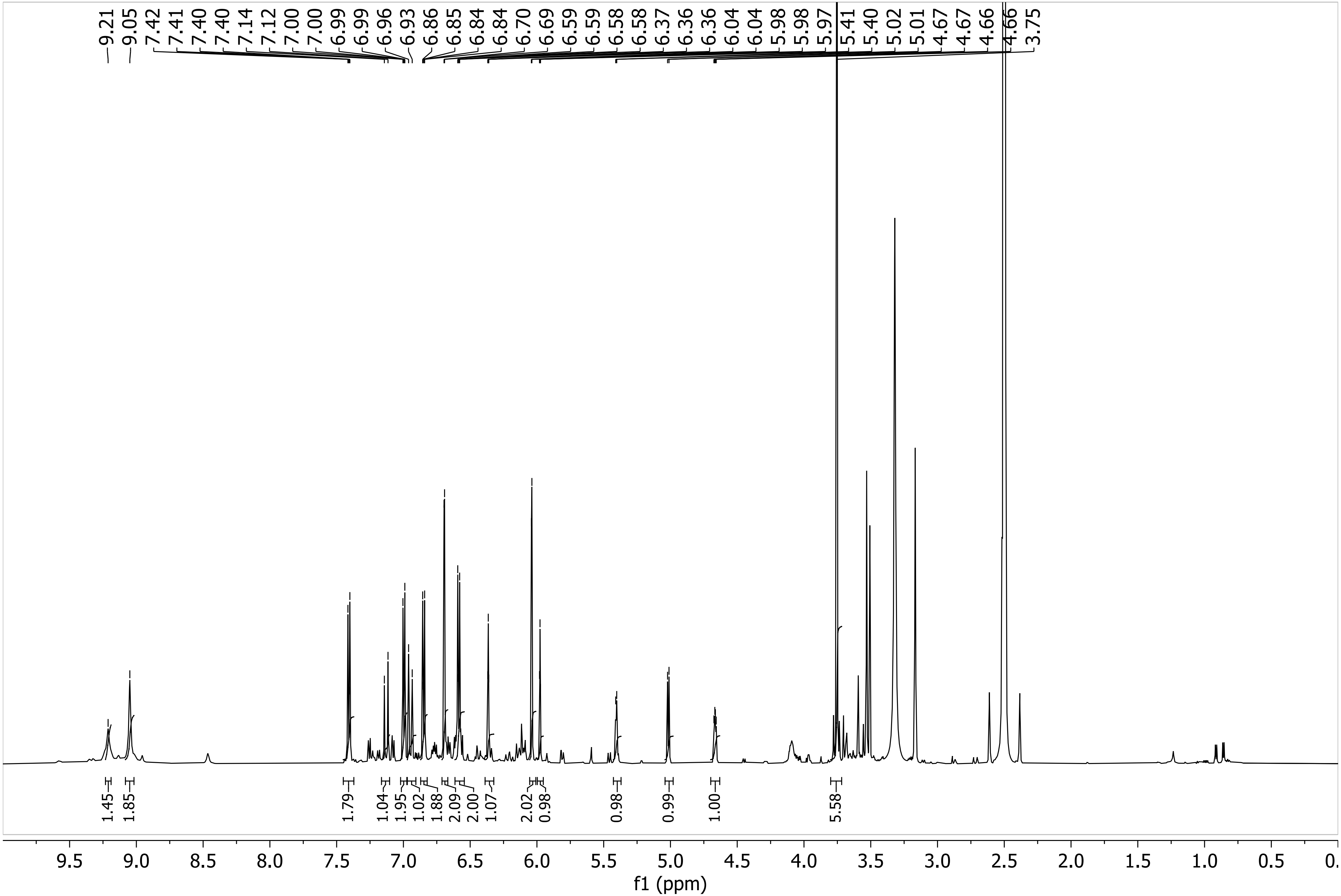

COSY NMR spectrum of compound **13** in DMSO-d_6

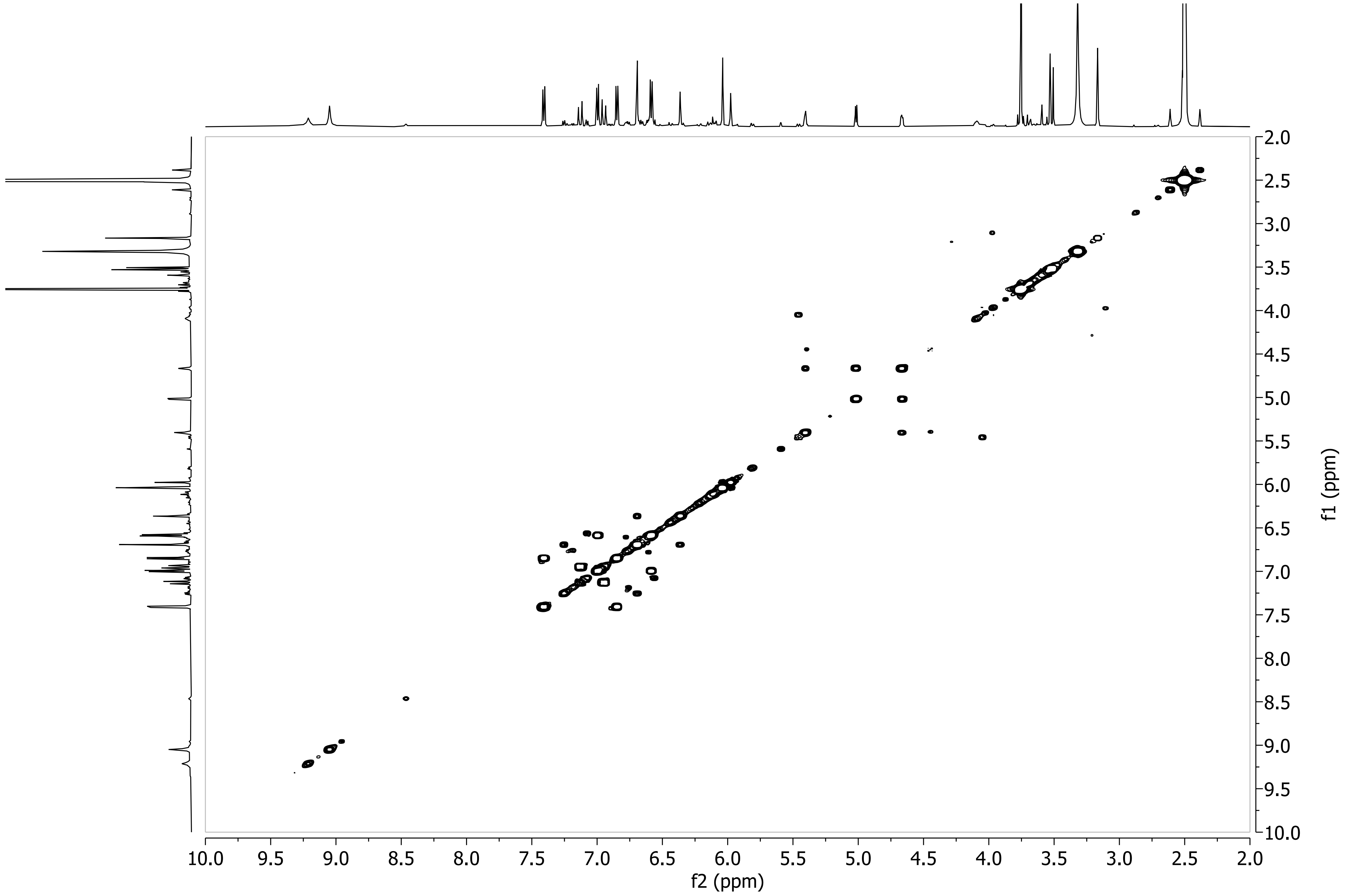

^{13}C-DEPTQ NMR spectrum of compound **13** in DMSO-d_6

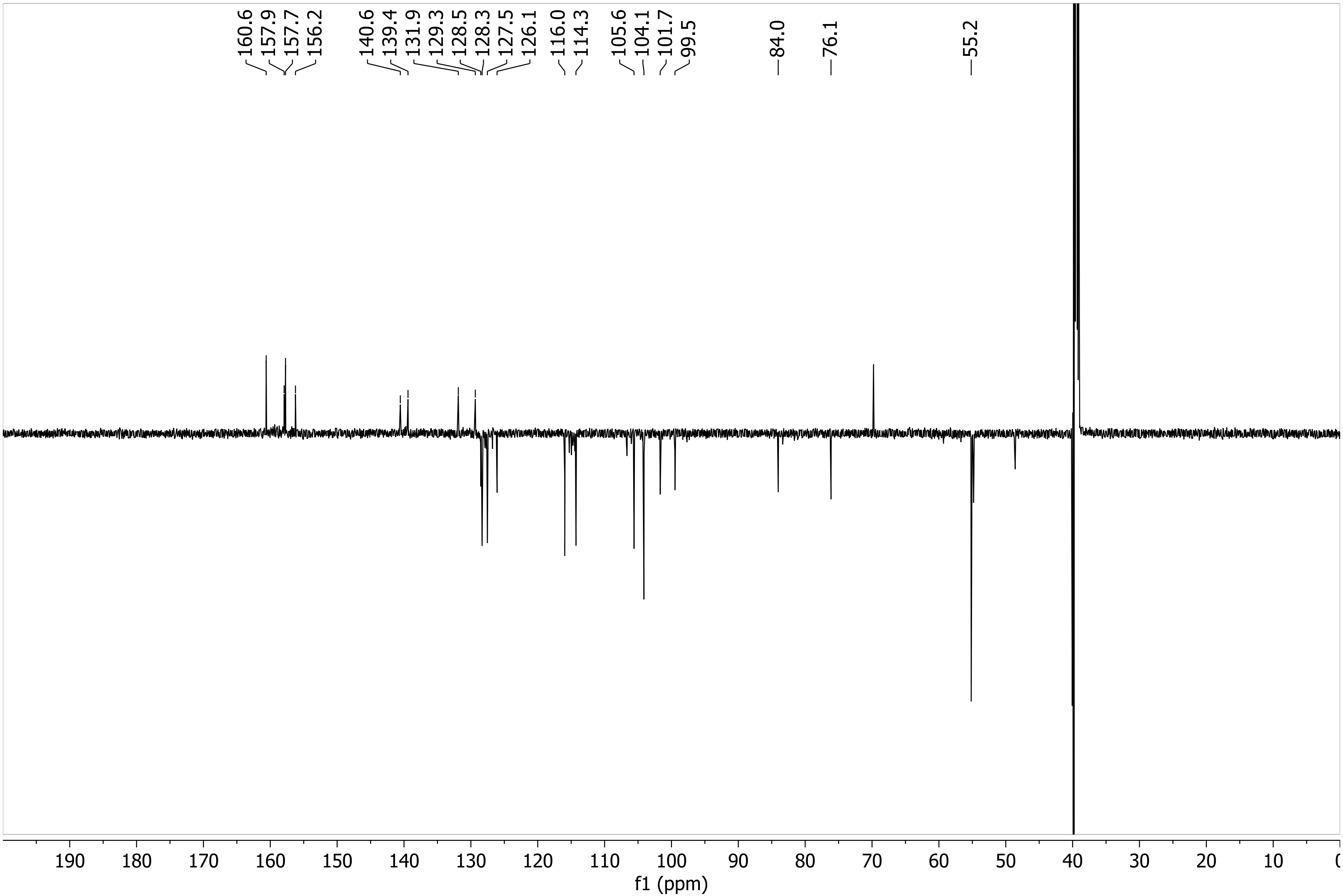

Edited-HSQC NMR spectrum of compound **13** in DMSO-d_6

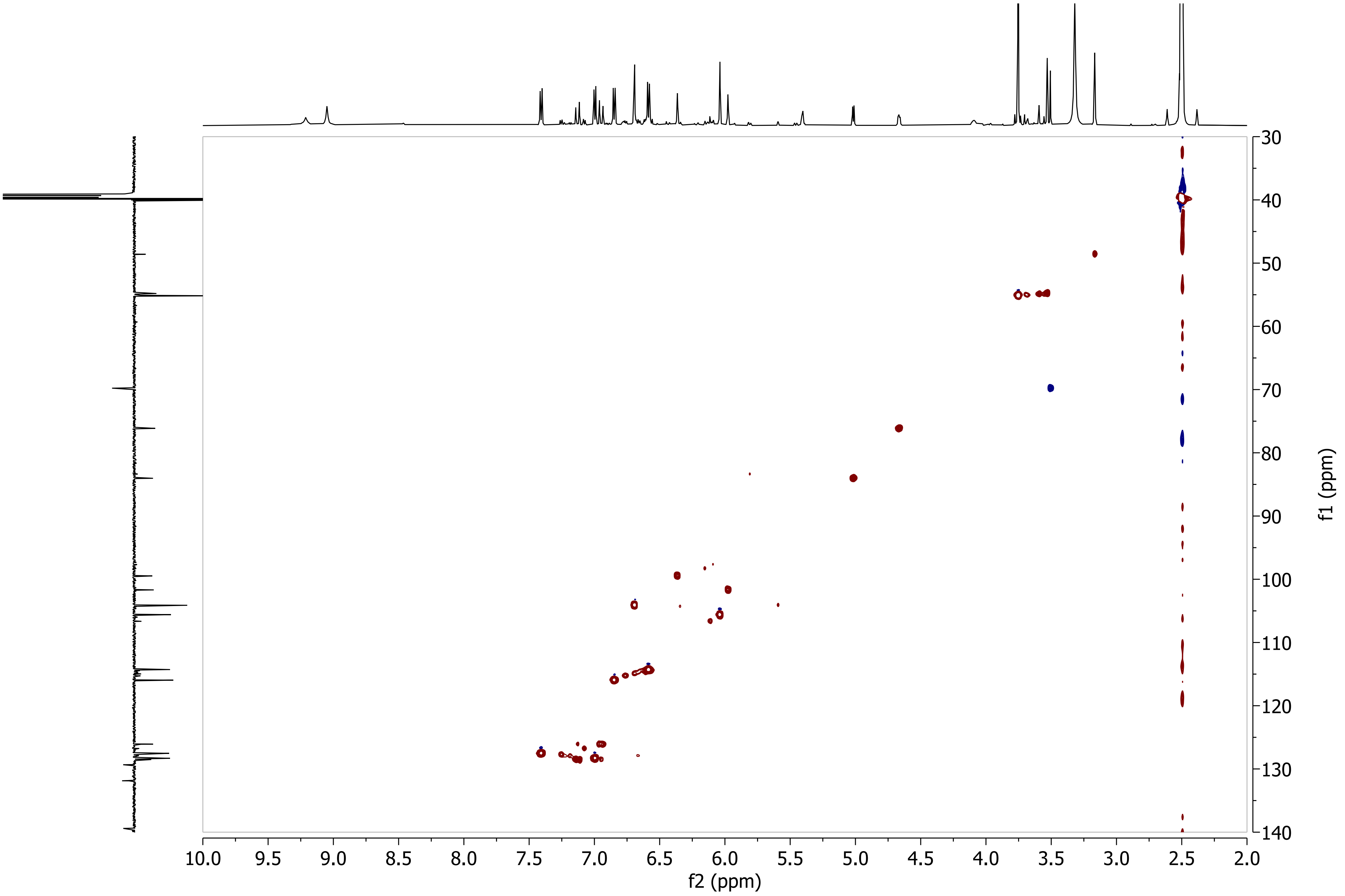

HMBC NMR spectrum of compound **13** in DMSO-d_6

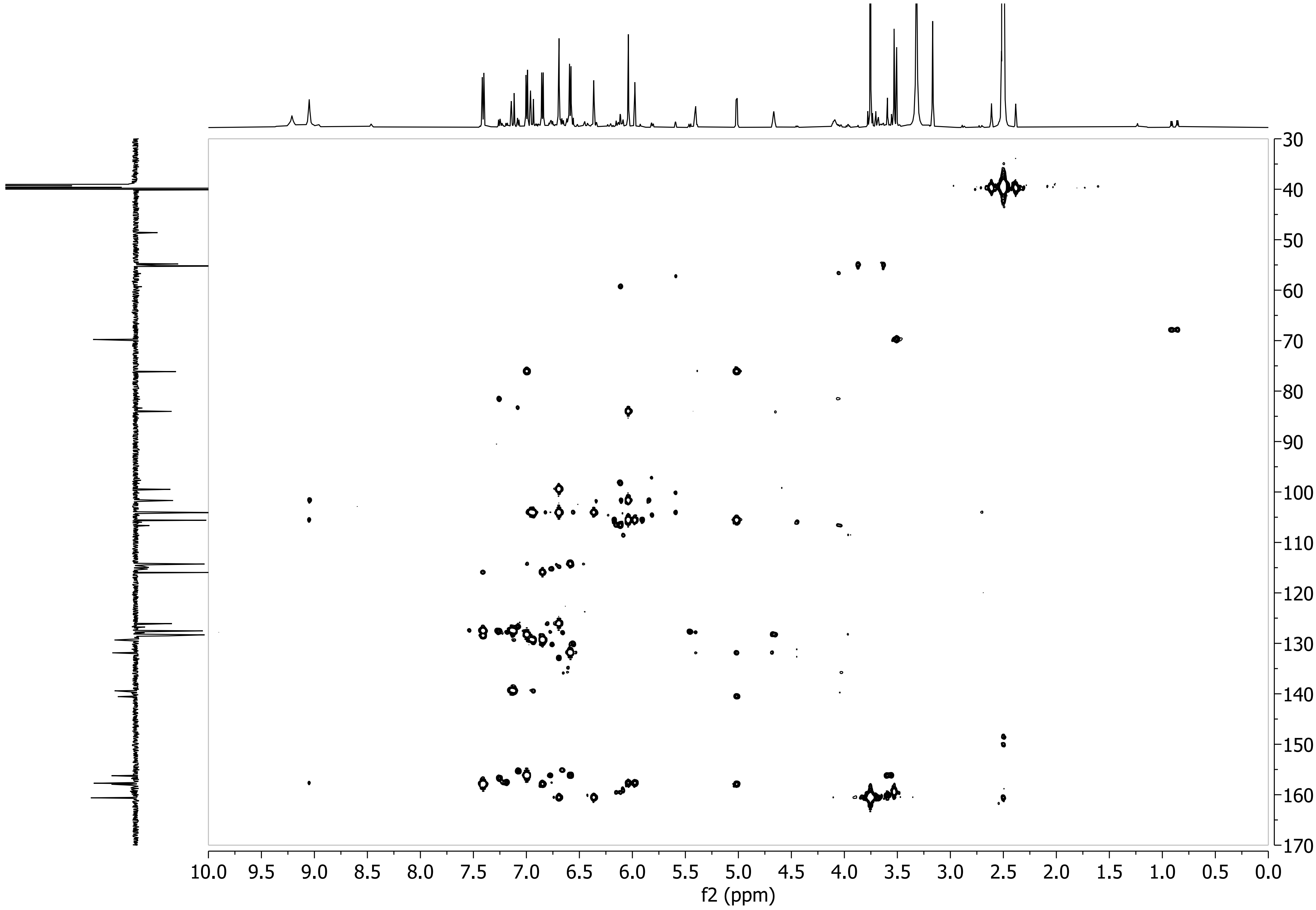

ROESY NMR spectrum of compound **13** in DMSO-d_6

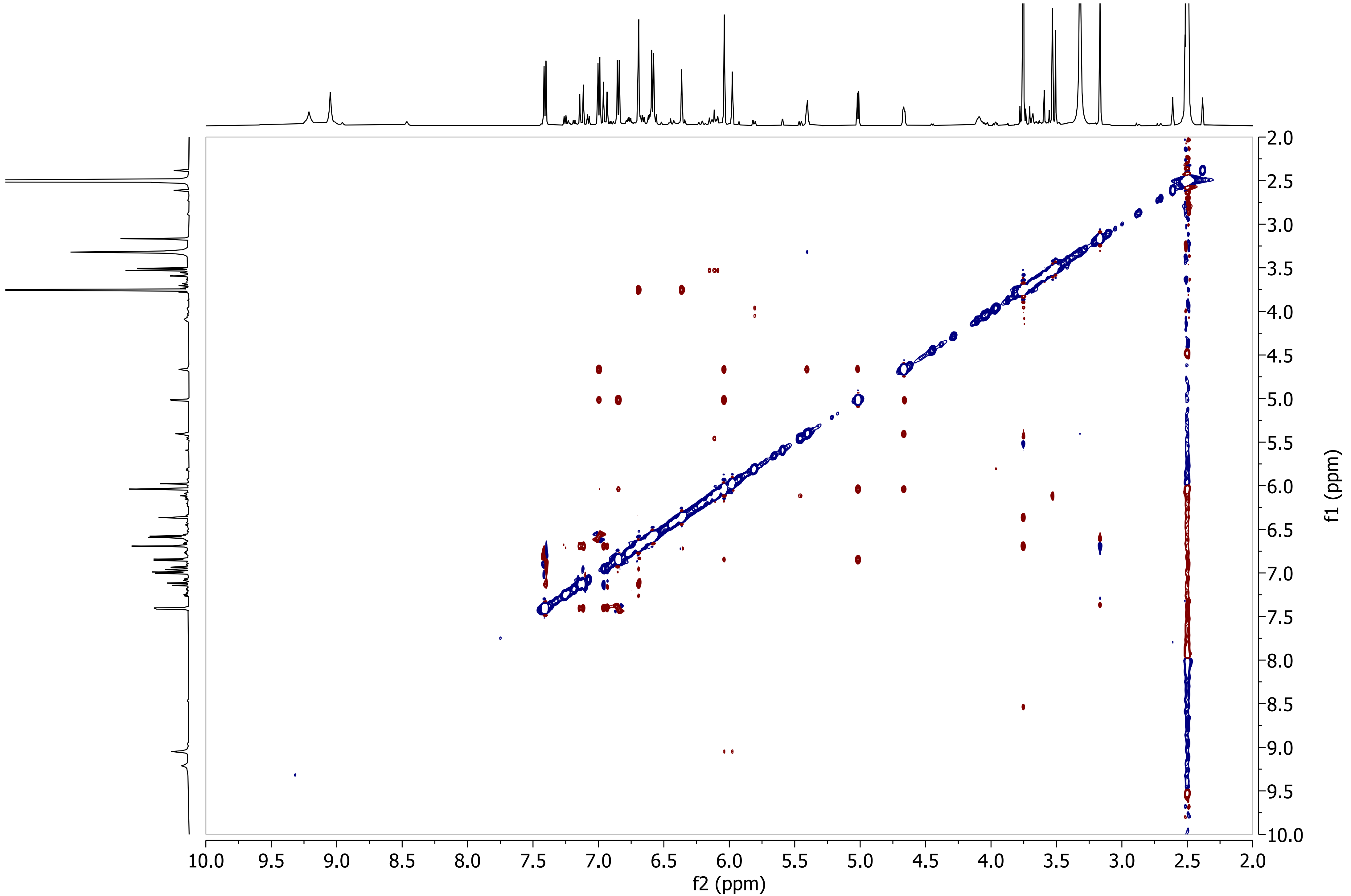

^{1}H NMR spectrum of compound **14** in DMSO-d_6

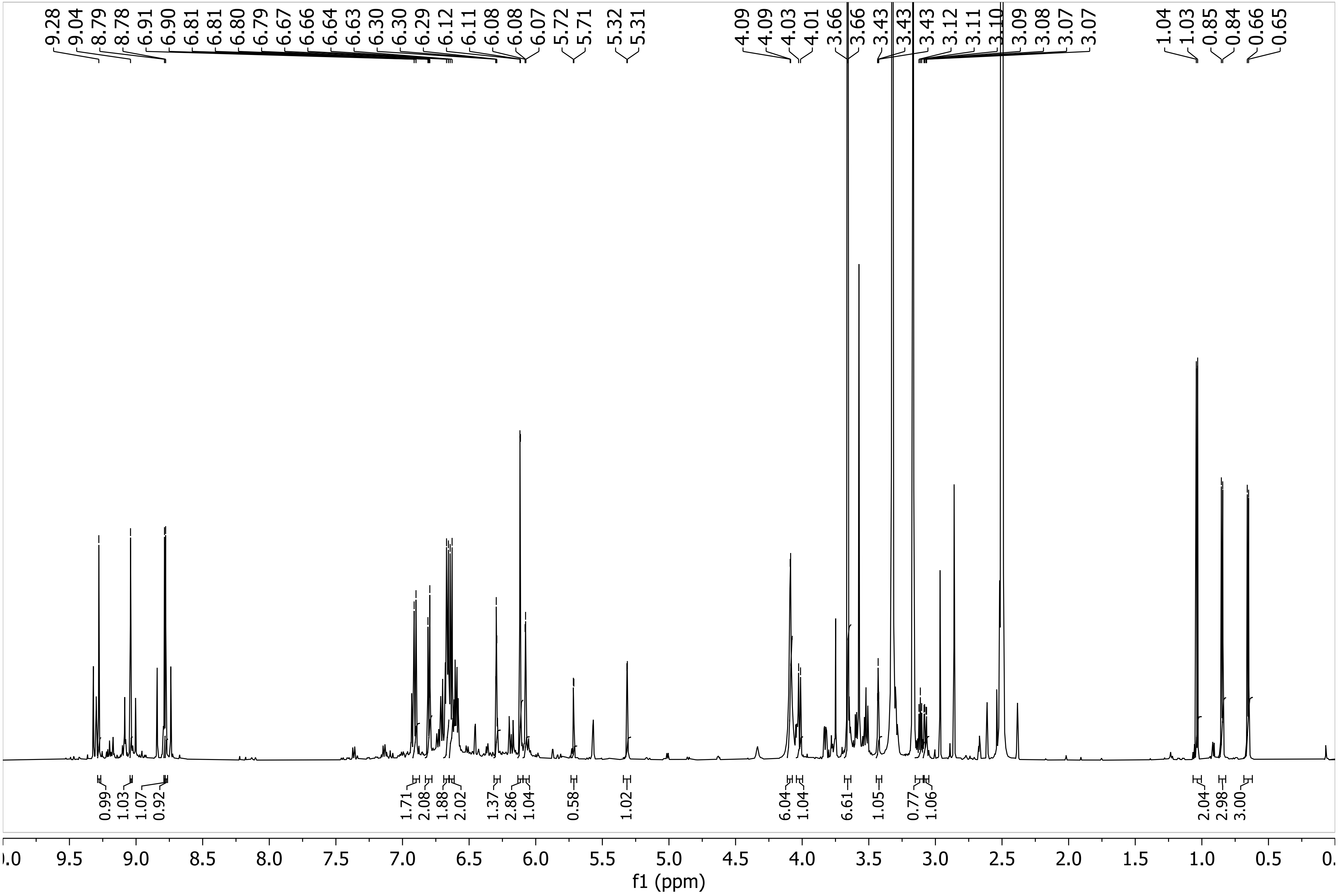

^{1}H NMR spectrum of compound **14** in DMSO-d_6

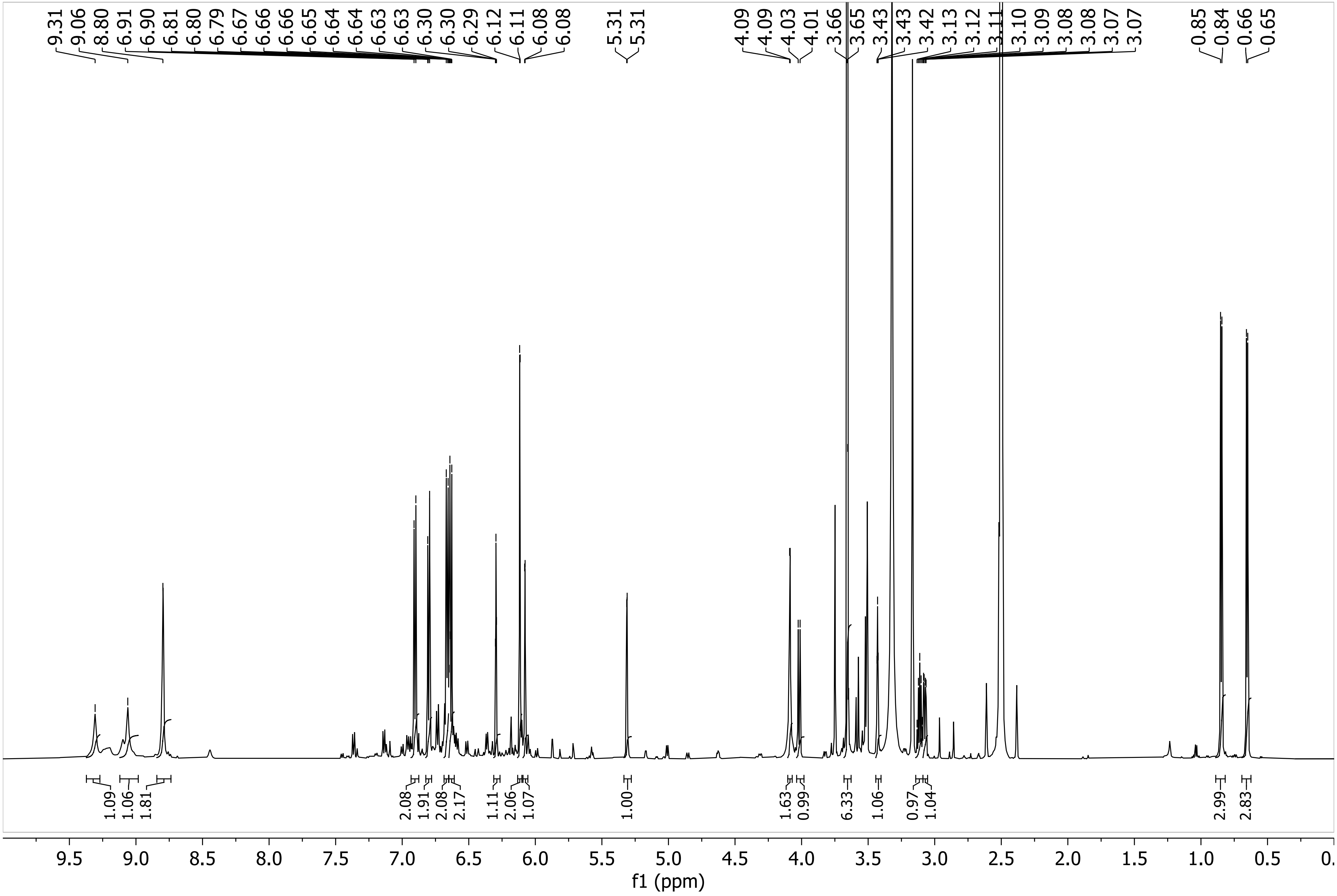

COSY NMR spectrum of compound **14** in DMSO-d_6

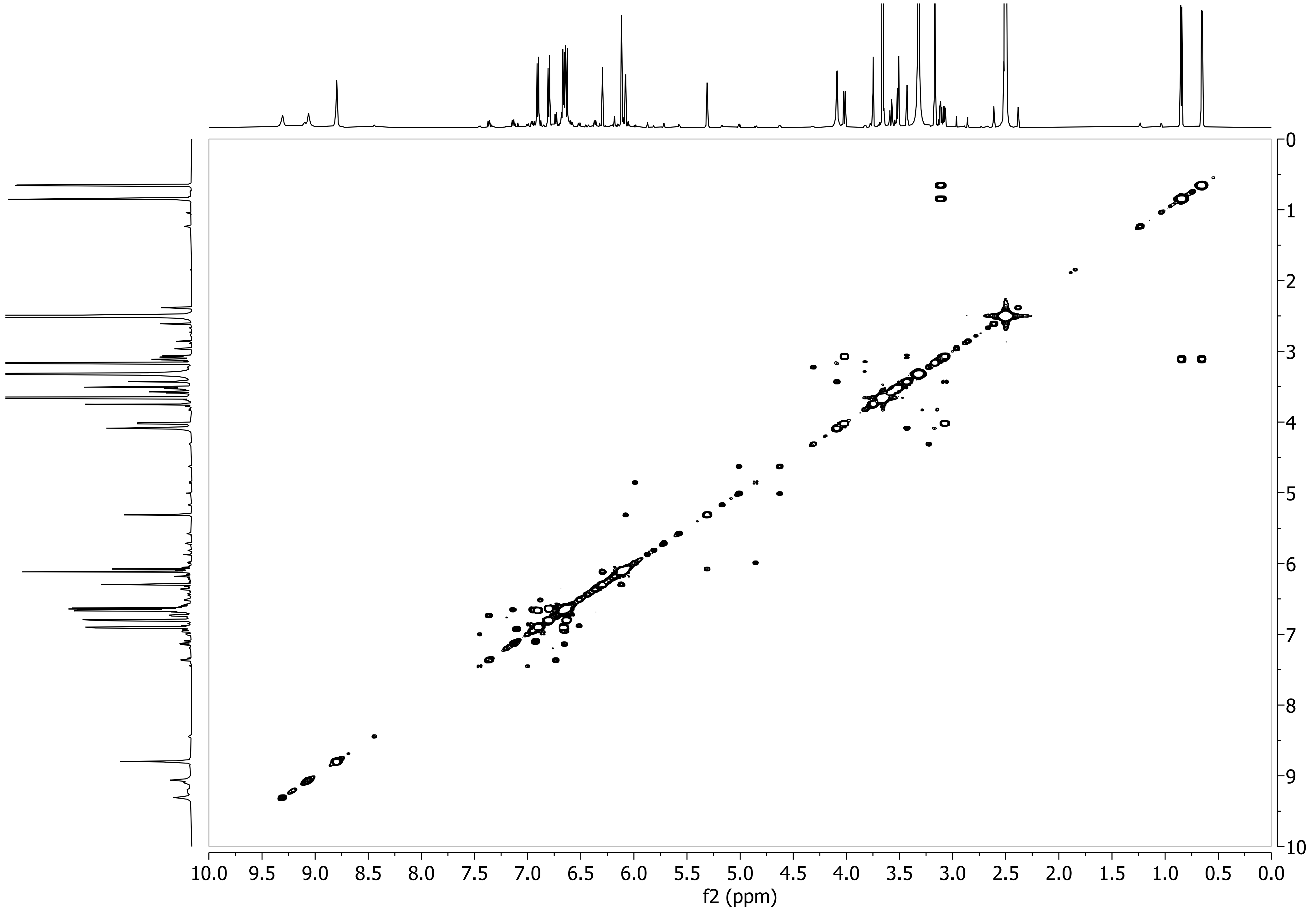

^{13}C-DEPTQ NMR spectrum of compound **14** in DMSO-d_6

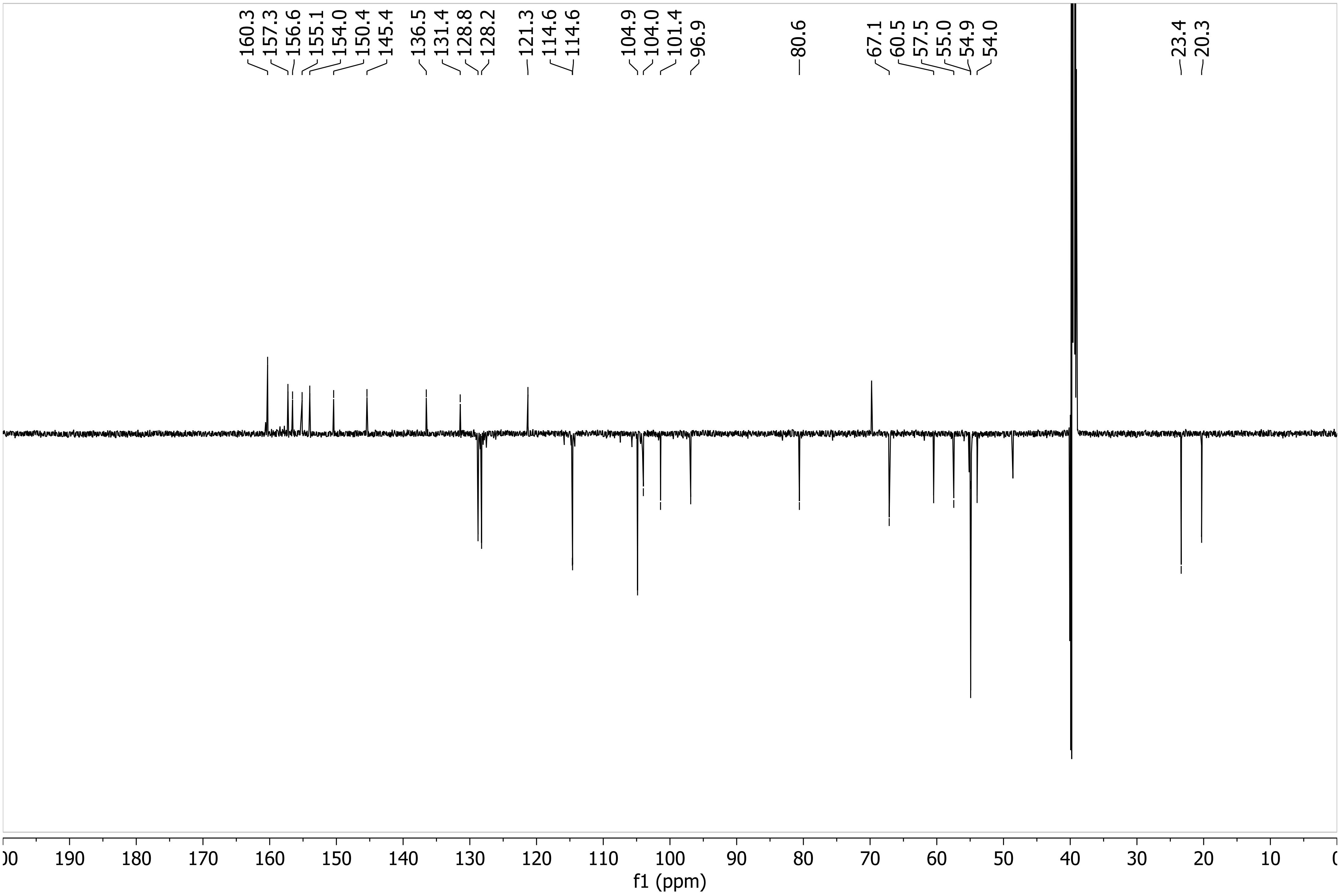

Edited-HSQC NMR spectrum of compound **14** in DMSO-d_6

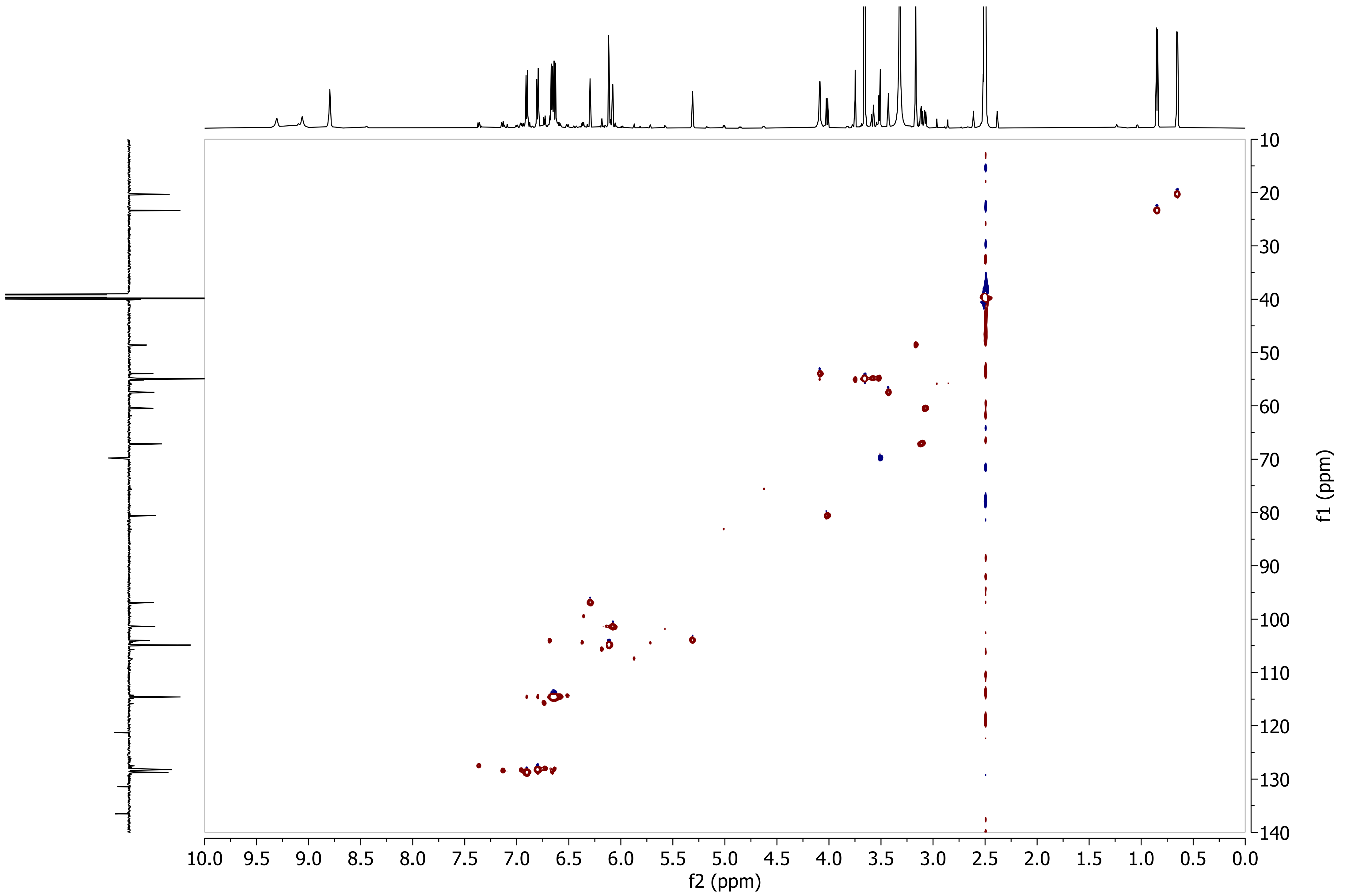

HMBC NMR spectrum of compound **14** in DMSO-d_6

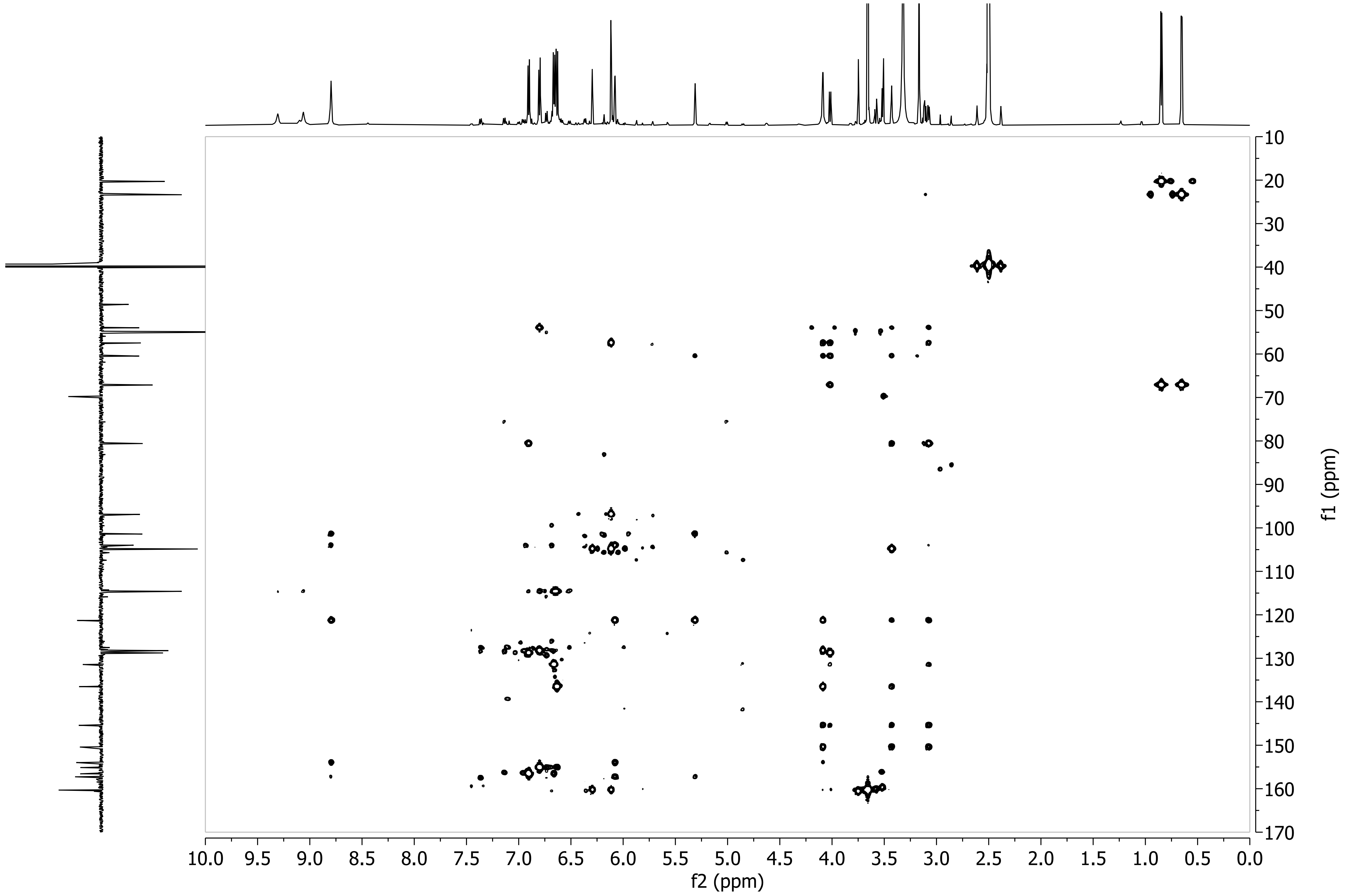

ROESY NMR spectrum of compound **14** in DMSO-d_6

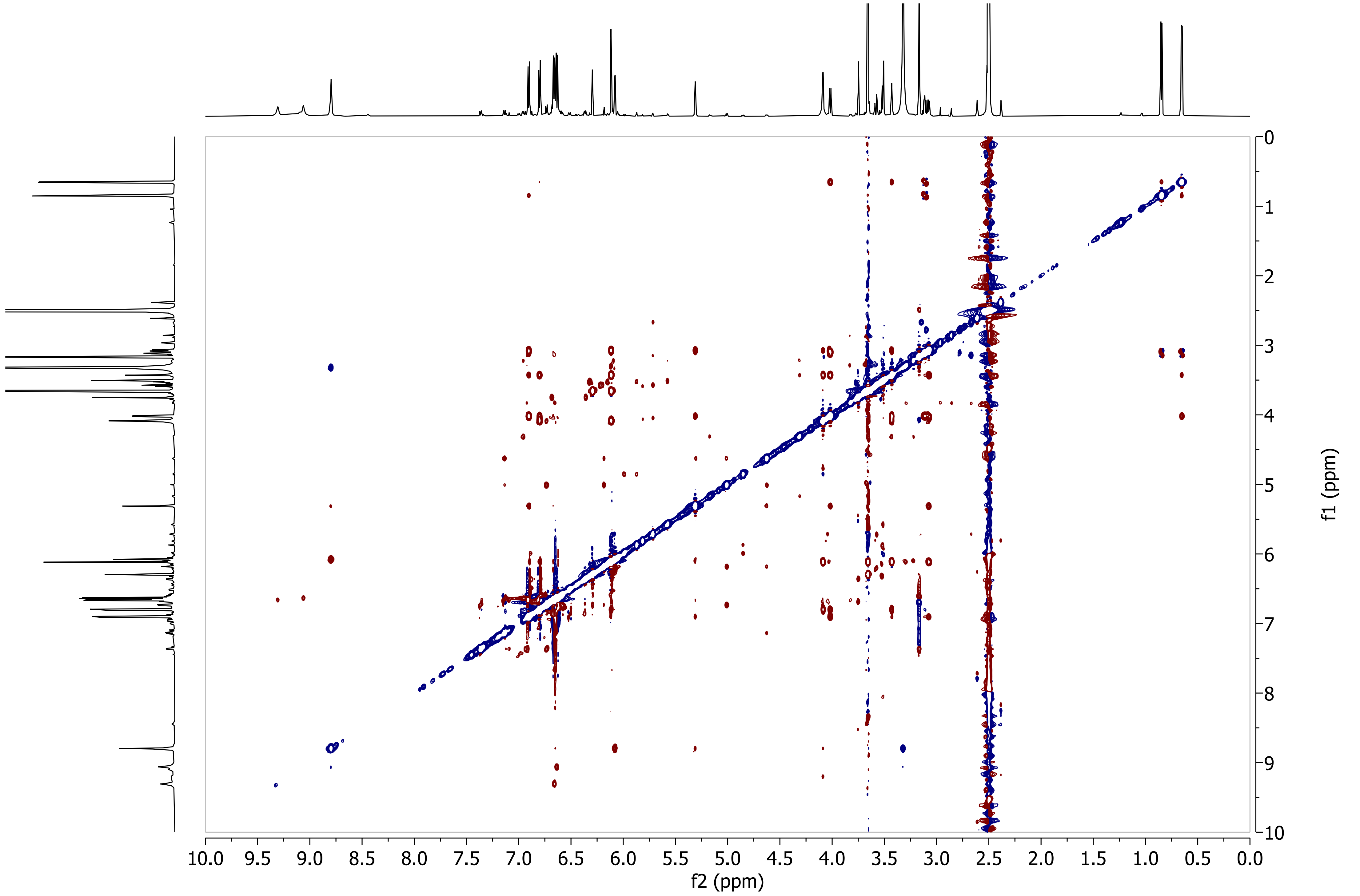

^{1}H NMR spectrum of compound **15** in DMSO-d_6

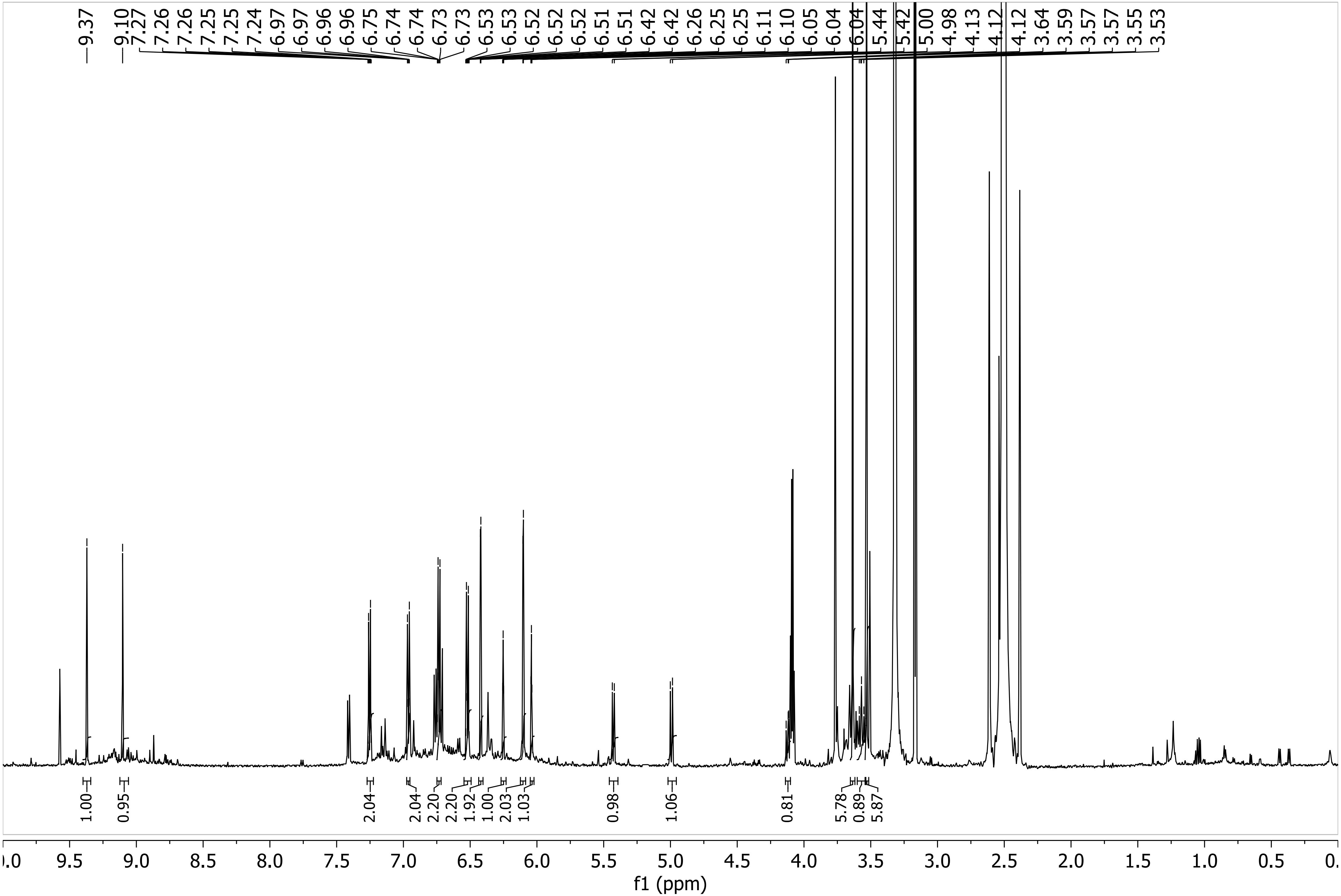

COSY NMR spectrum of compound **15** in DMSO-d_6

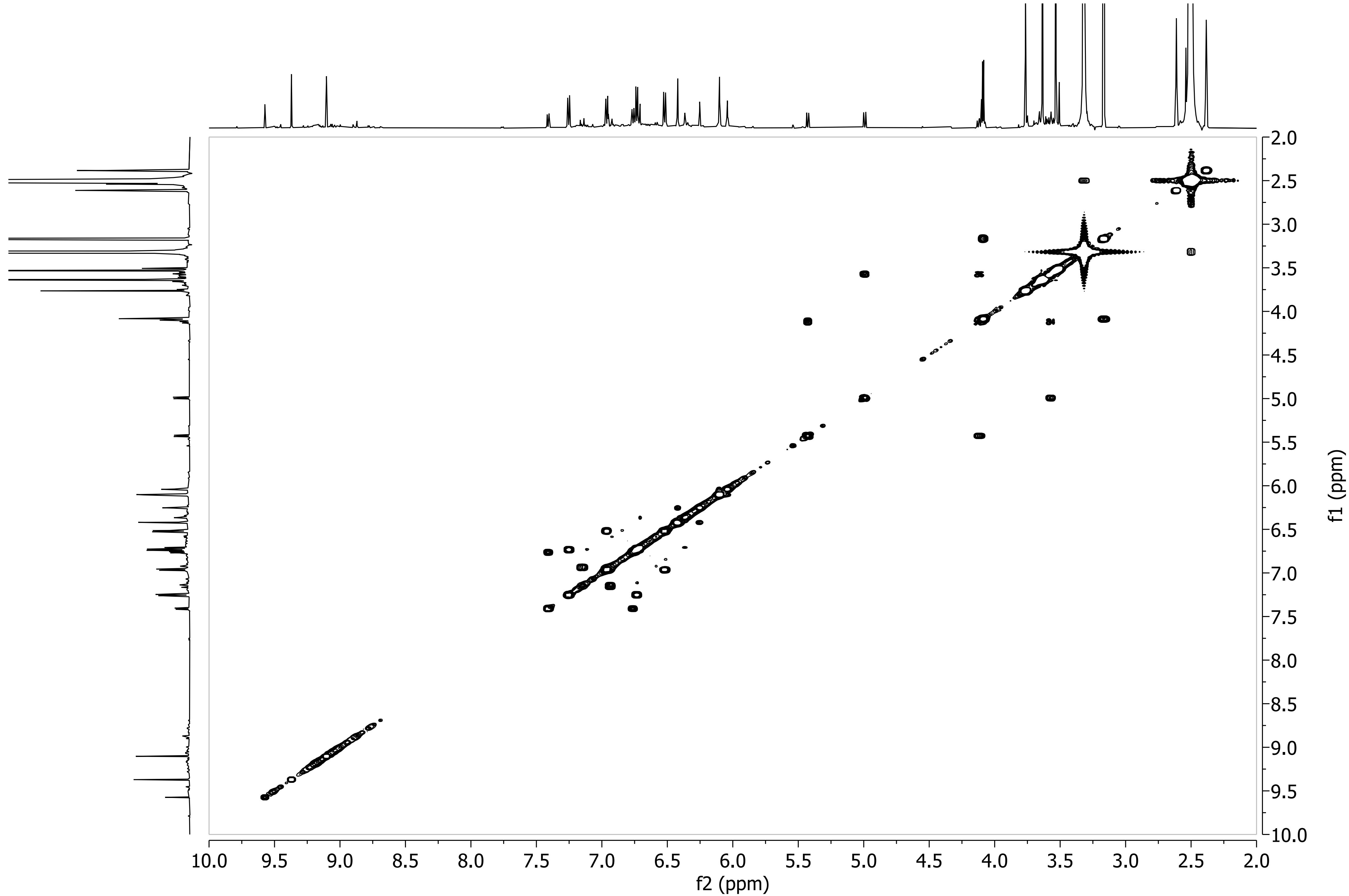

Edited-HSQC NMR spectrum of compound **15** in DMSO-d_6

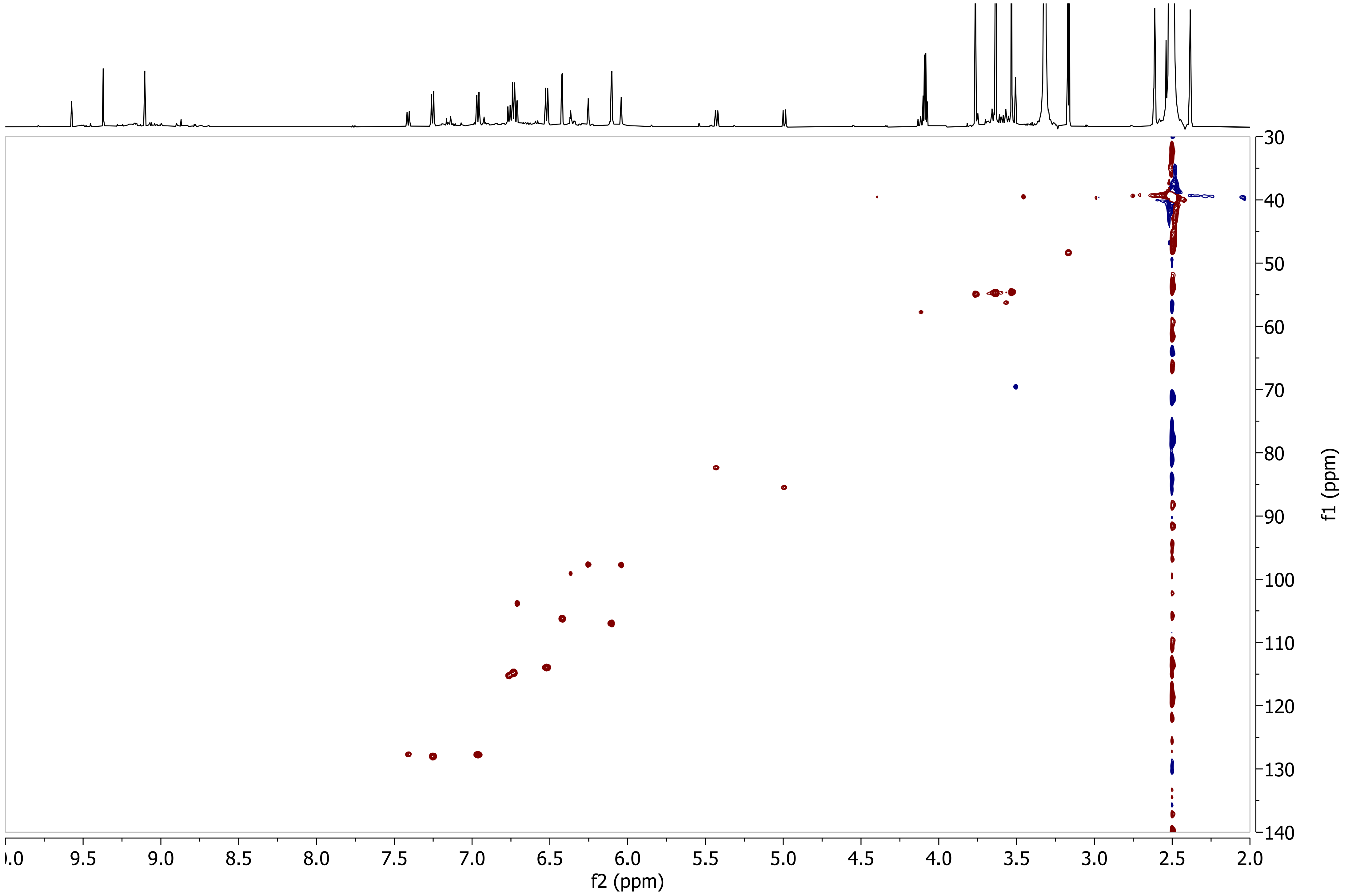

HMBC NMR spectrum of compound **15** in DMSO-d_6

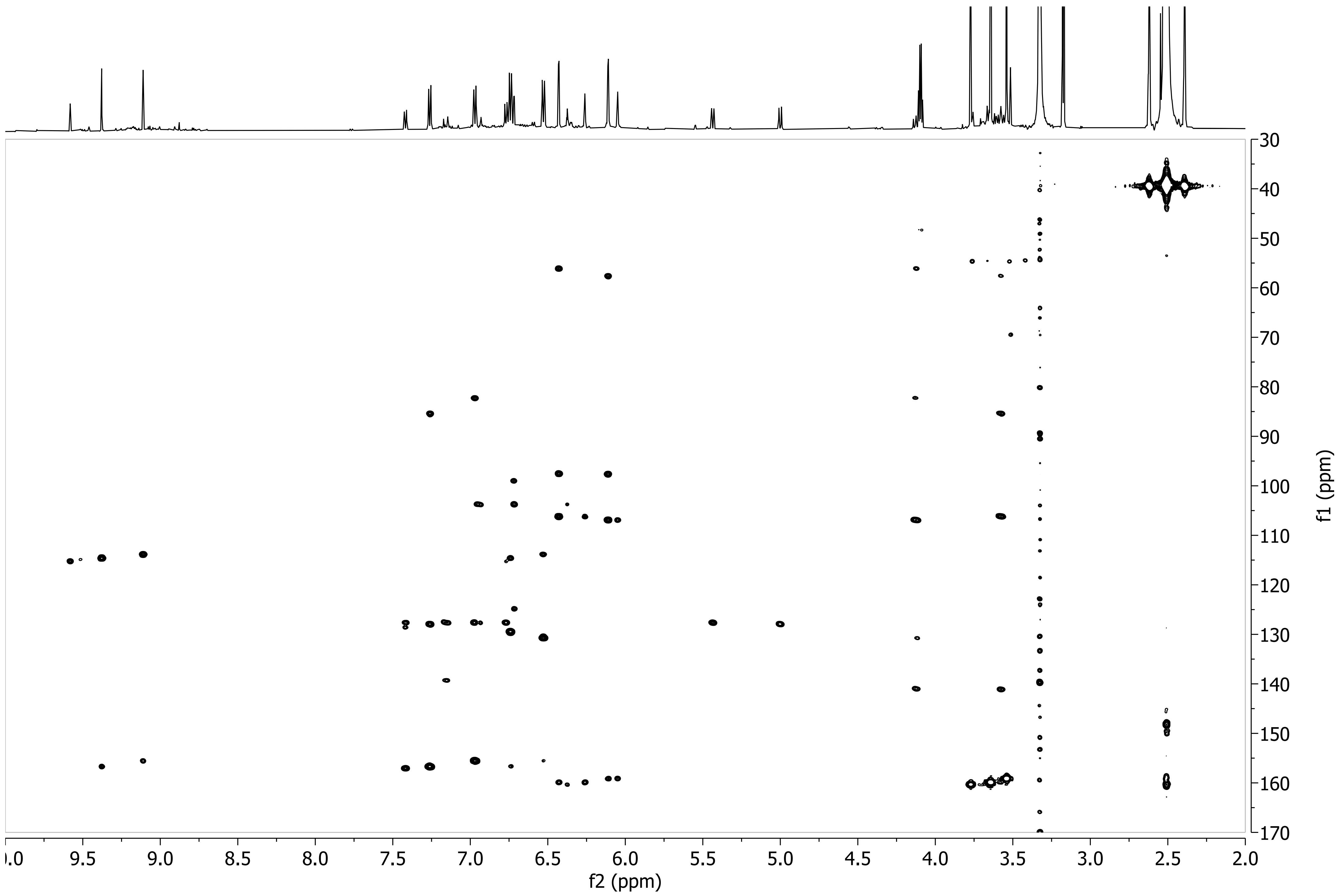

ROESY NMR spectrum of compound **15** in DMSO-d_6

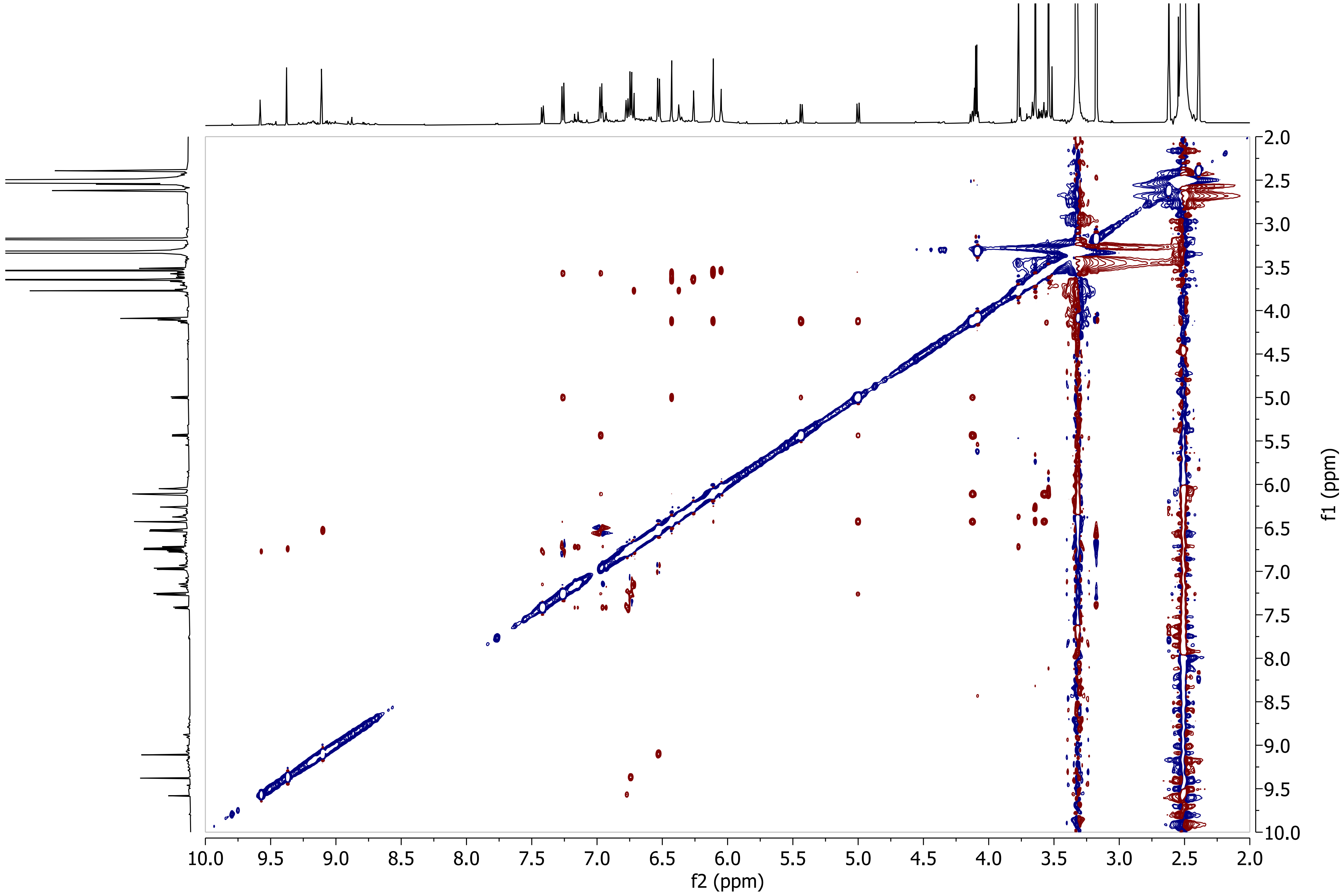

^{1}H NMR spectrum of compound **16** in DMSO-d_6

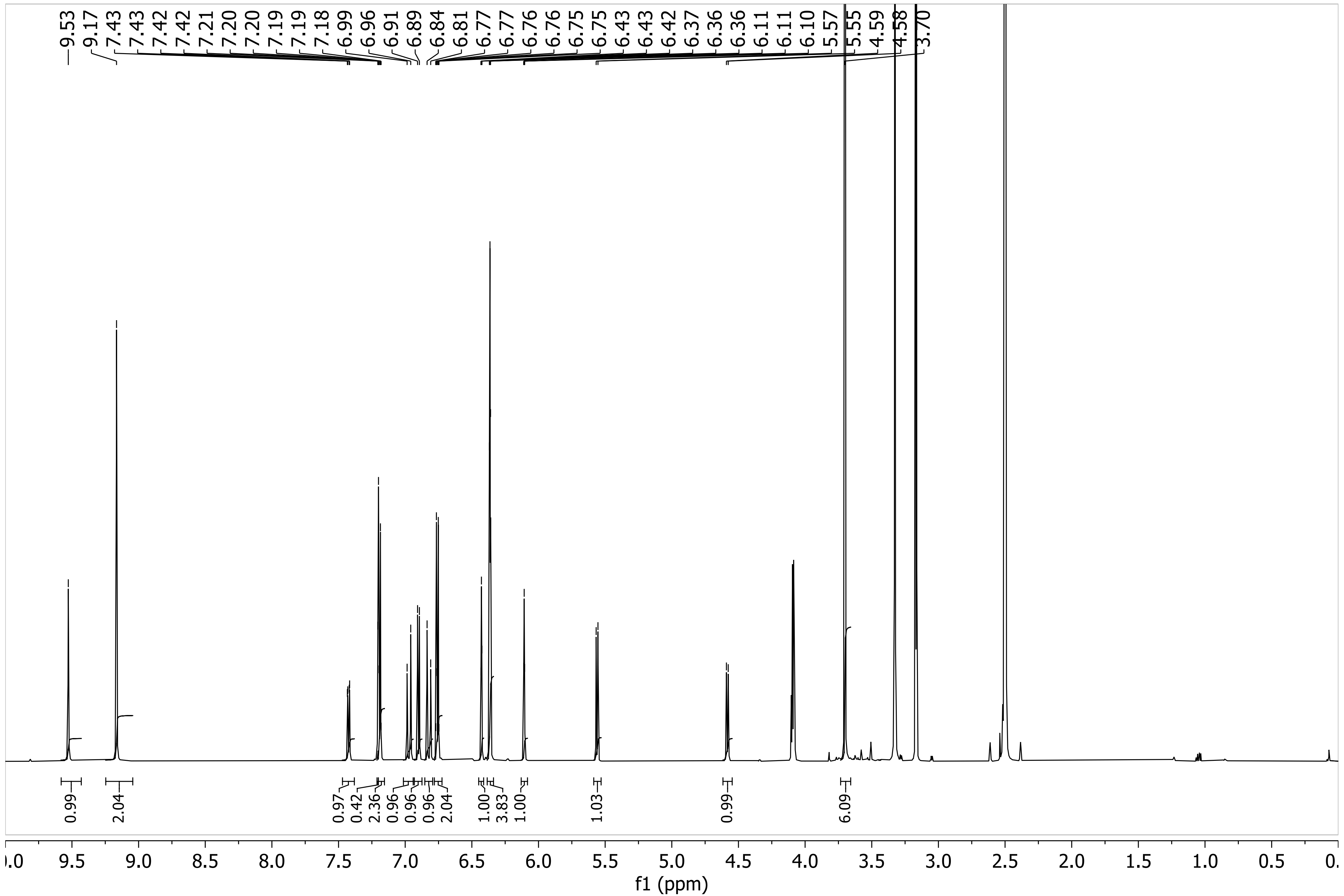

^{1}H NMR spectrum of compound **17** in DMSO-d_6

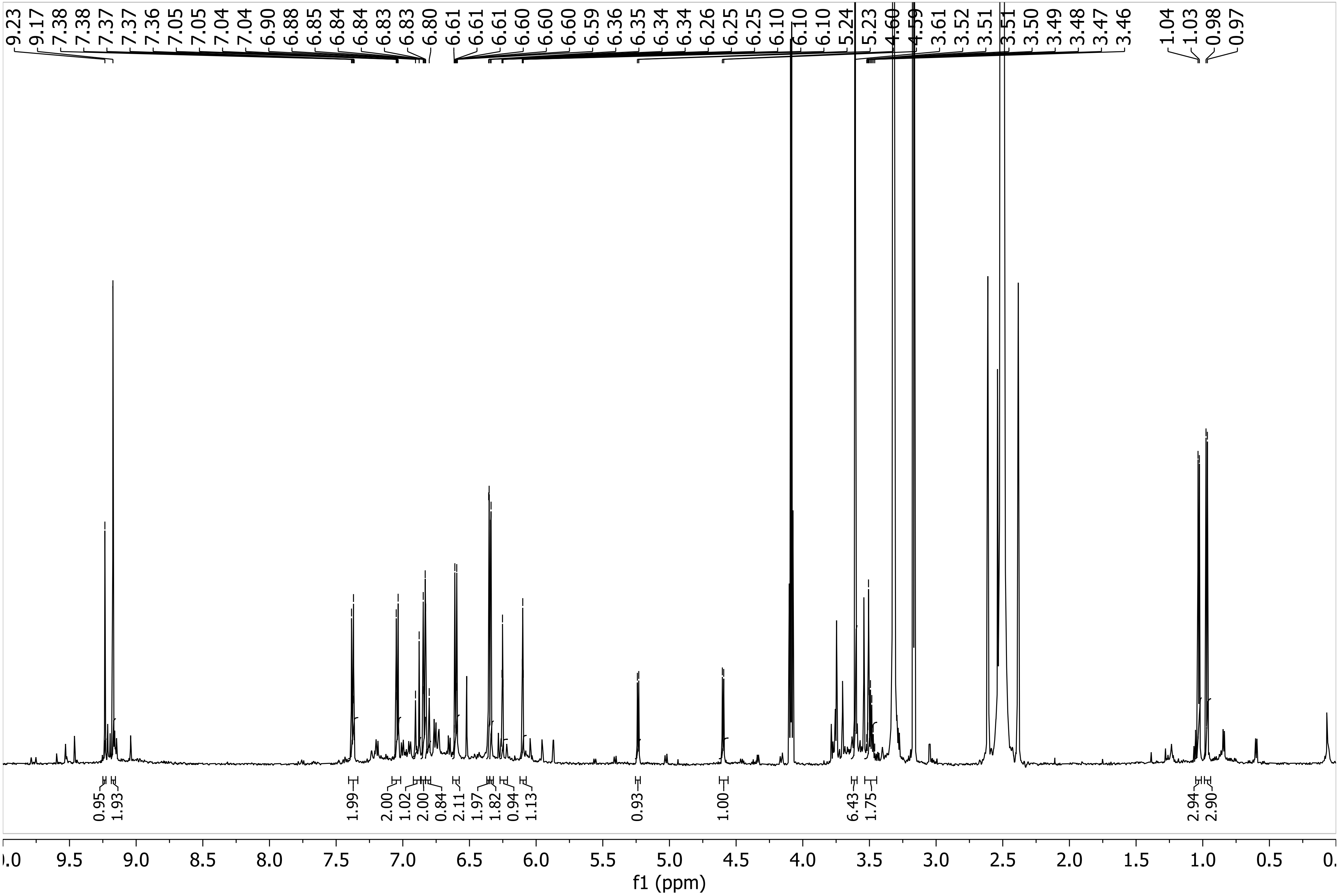

^{1}H NMR spectrum of compound **17** in DMSO-d_6

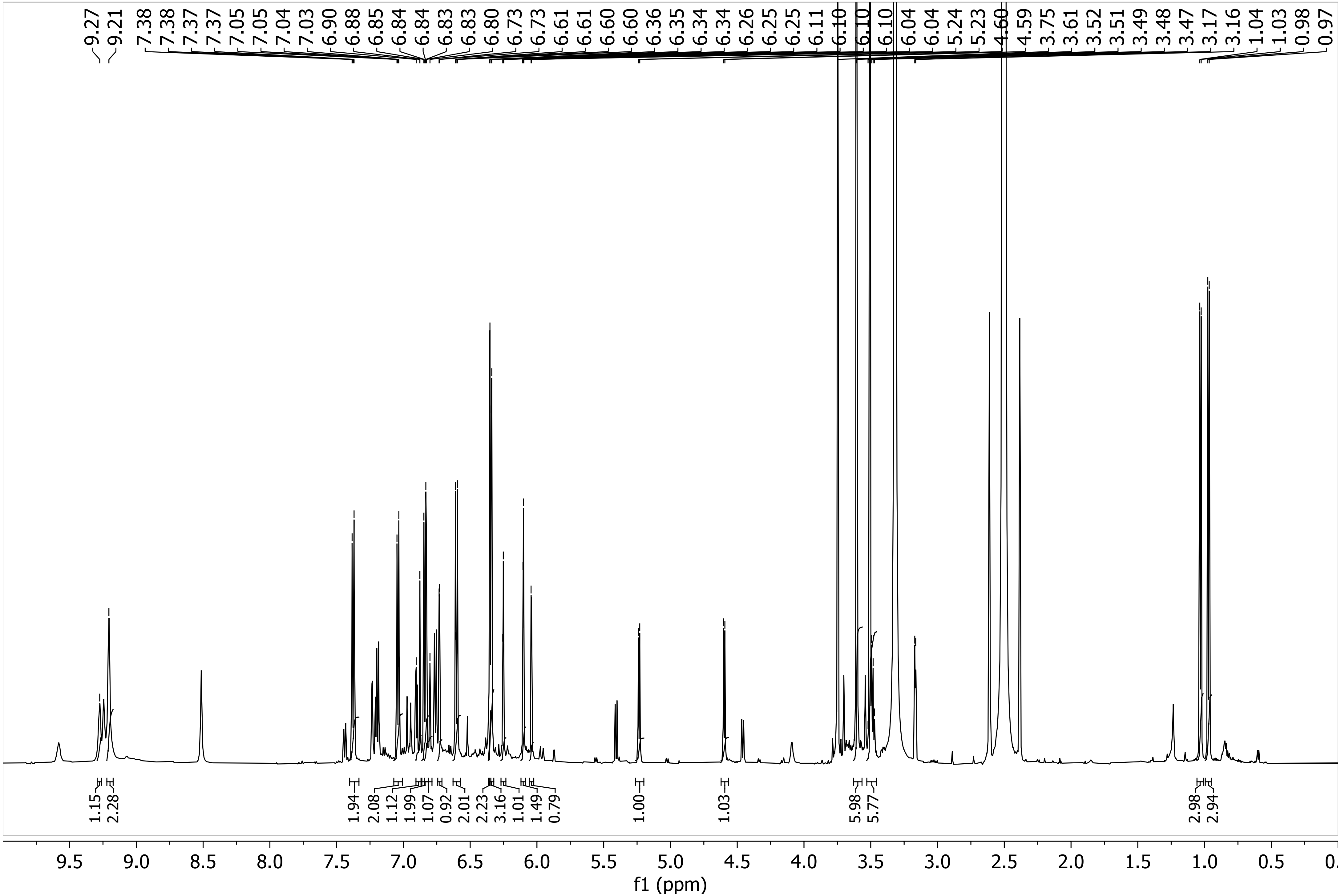

COSY NMR spectrum of compound **17** in DMSO-d_6

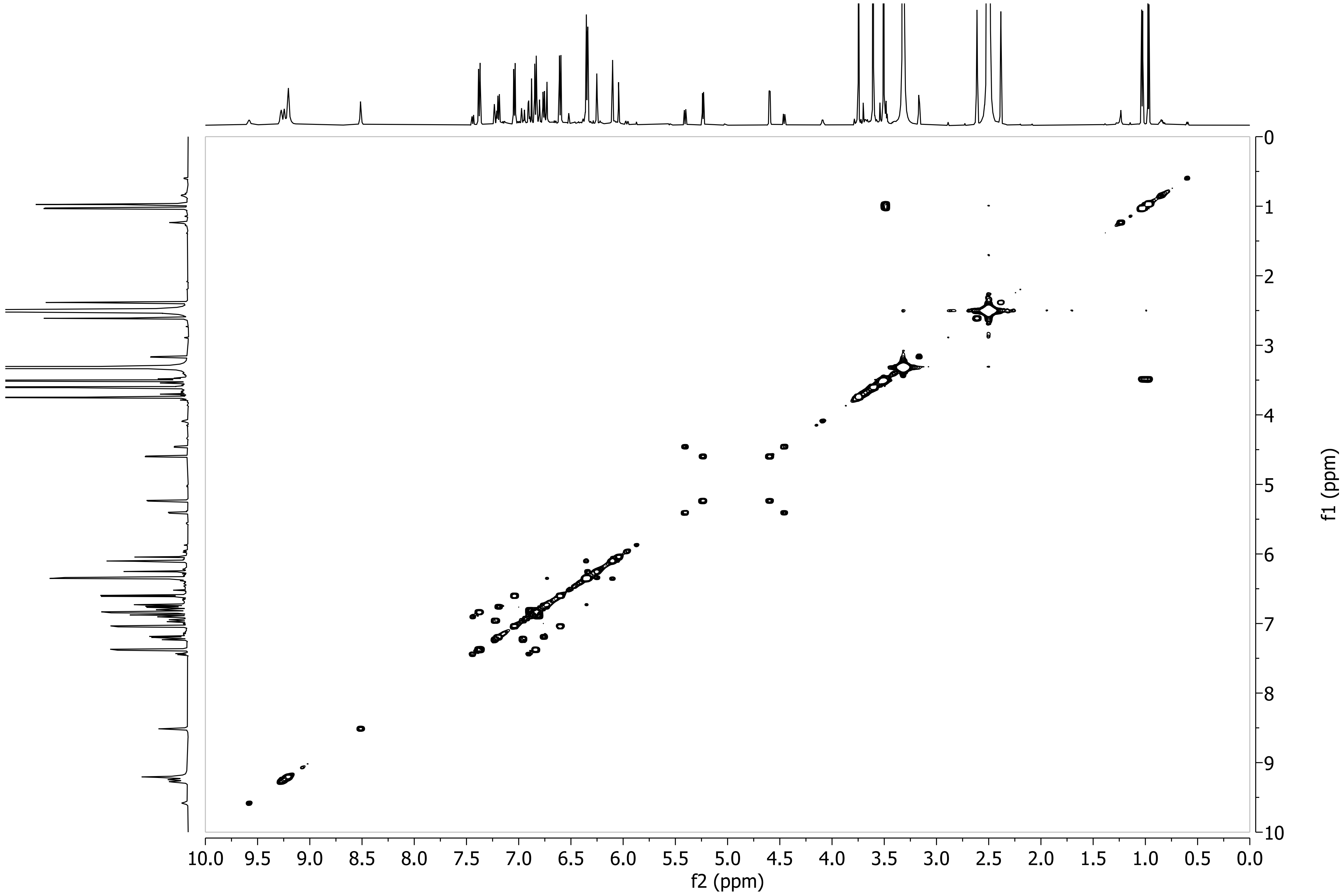

Edited-HSQC NMR spectrum of compound **17** in DMSO-d_6

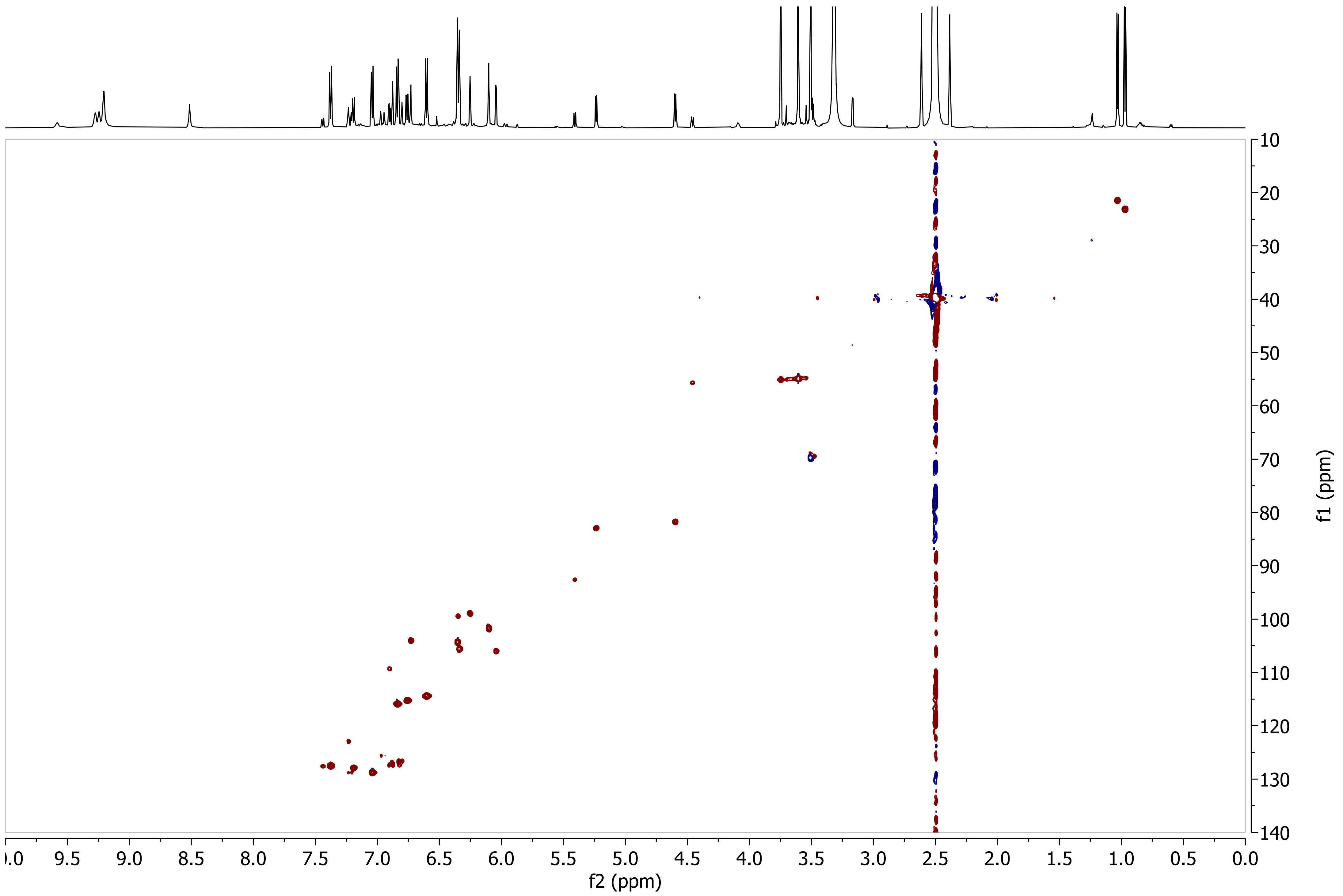

HMBC NMR spectrum of compound **17** in DMSO-d_6

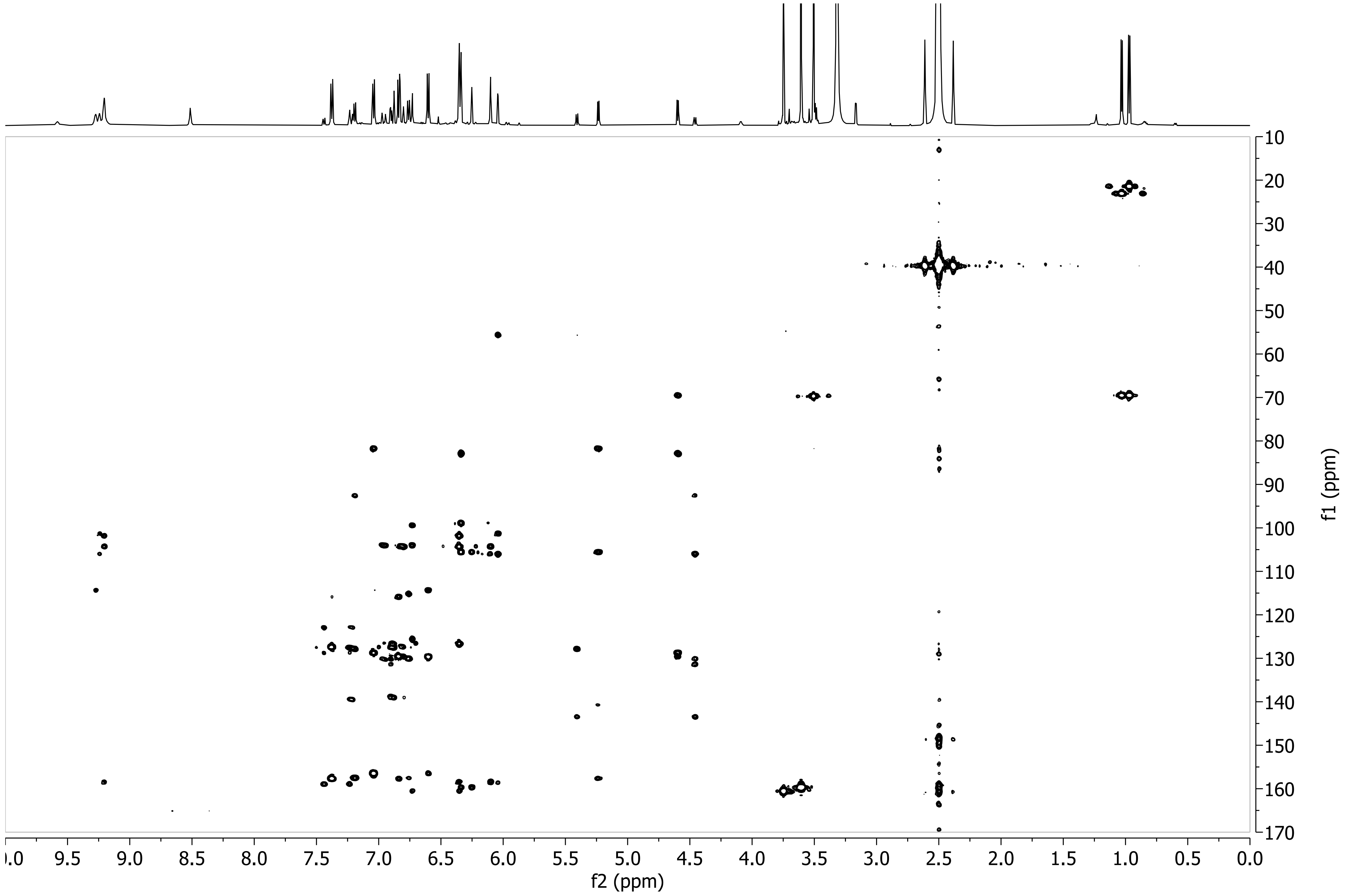

ROESY NMR spectrum of compound **17** in DMSO-d_6

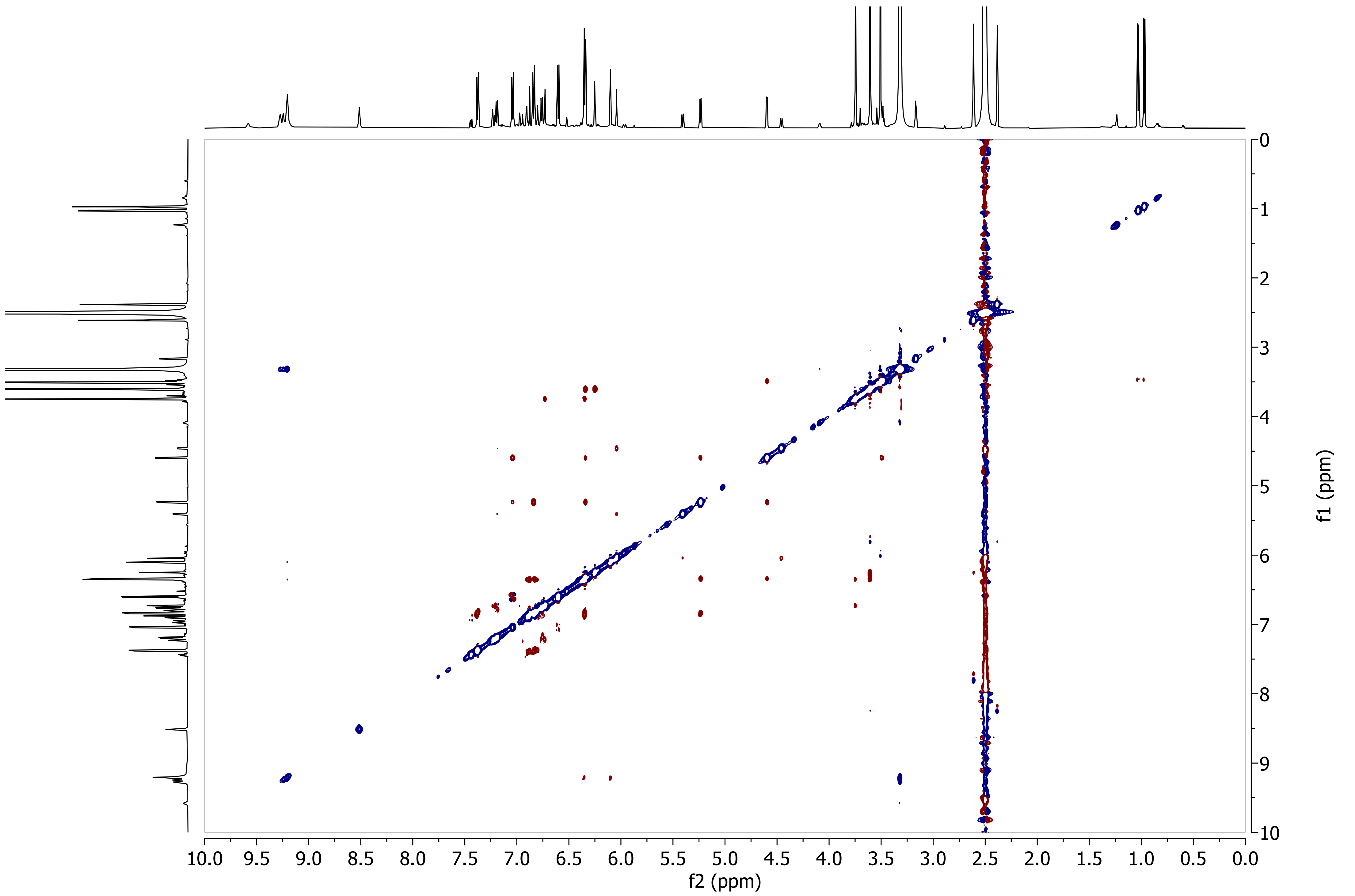

^1H NMR spectrum of compound **18** in DMSO-d_6

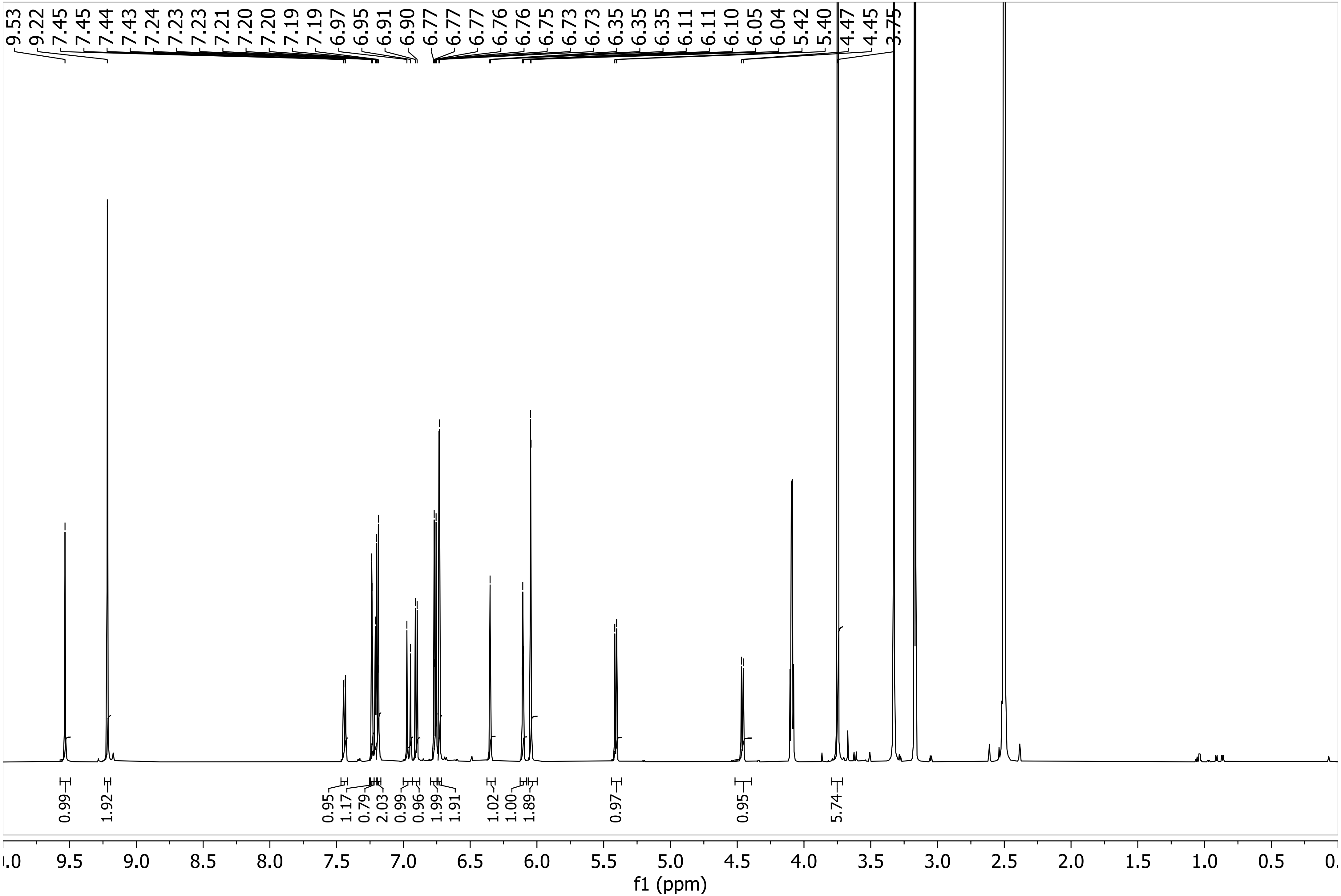

^{1}H NMR spectrum of compound **19** in DMSO-d_6

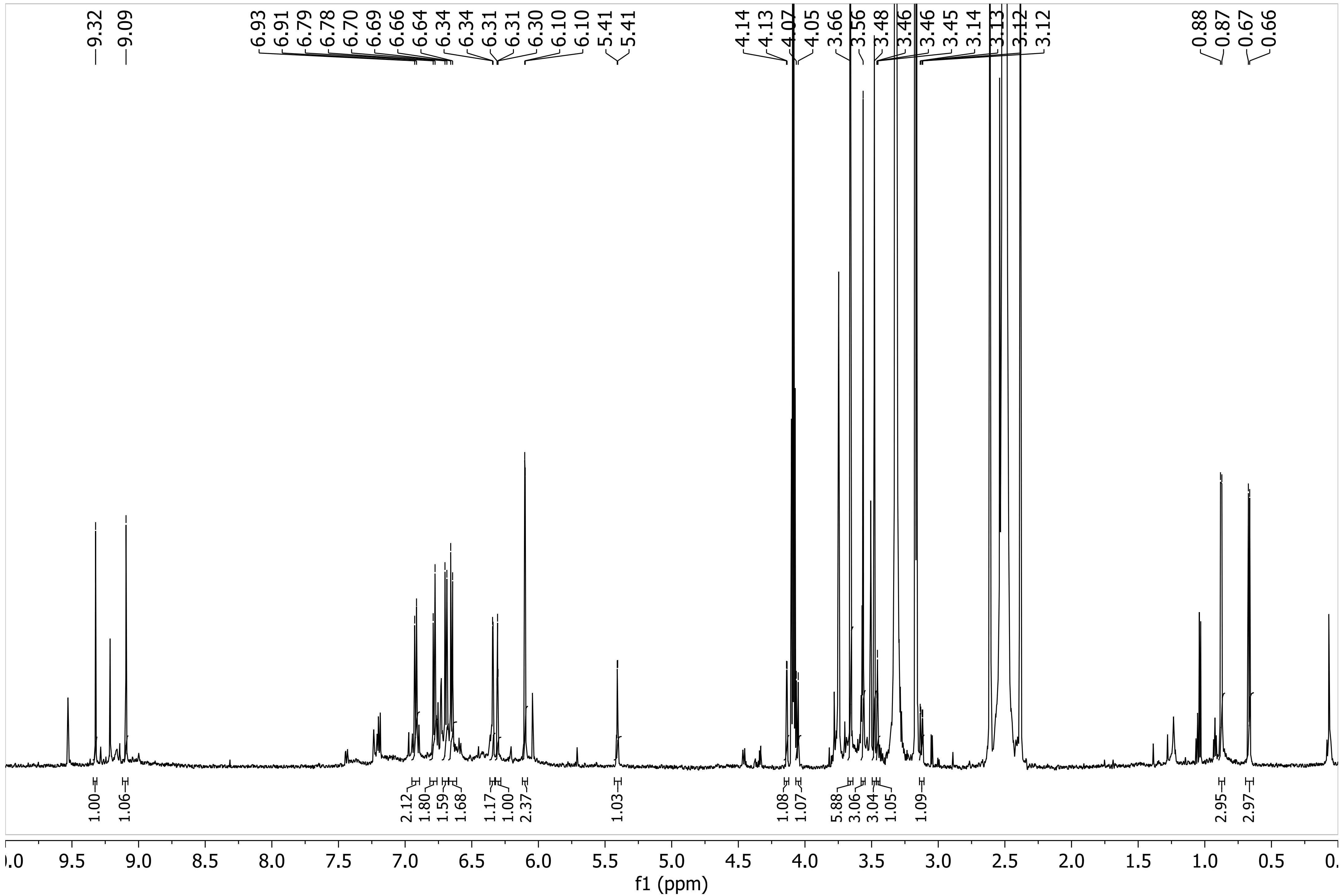

COSY NMR spectrum of compound **19** in DMSO-d_6

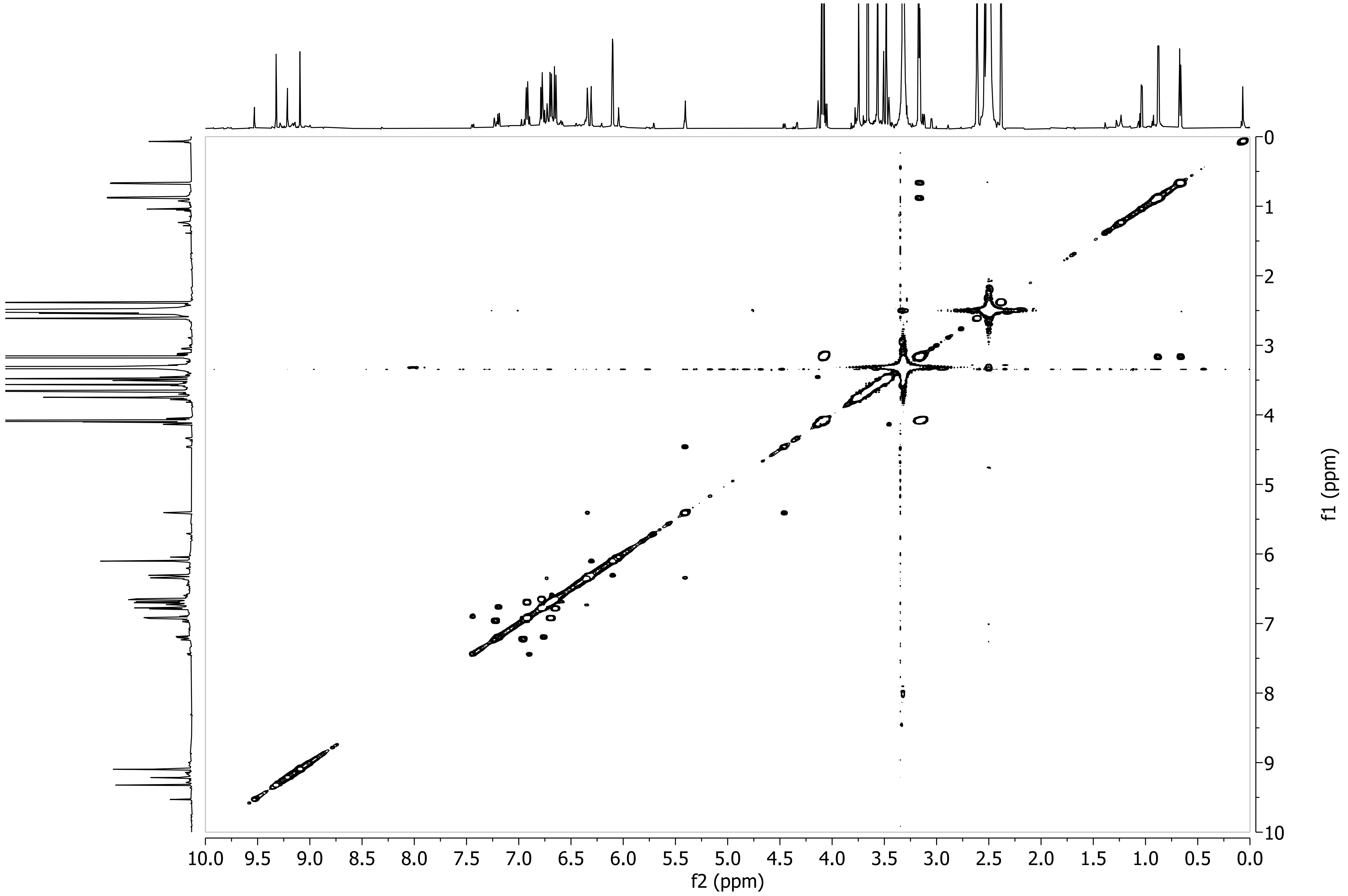

Edited-HSQC NMR spectrum of compound **19** in DMSO-d_6

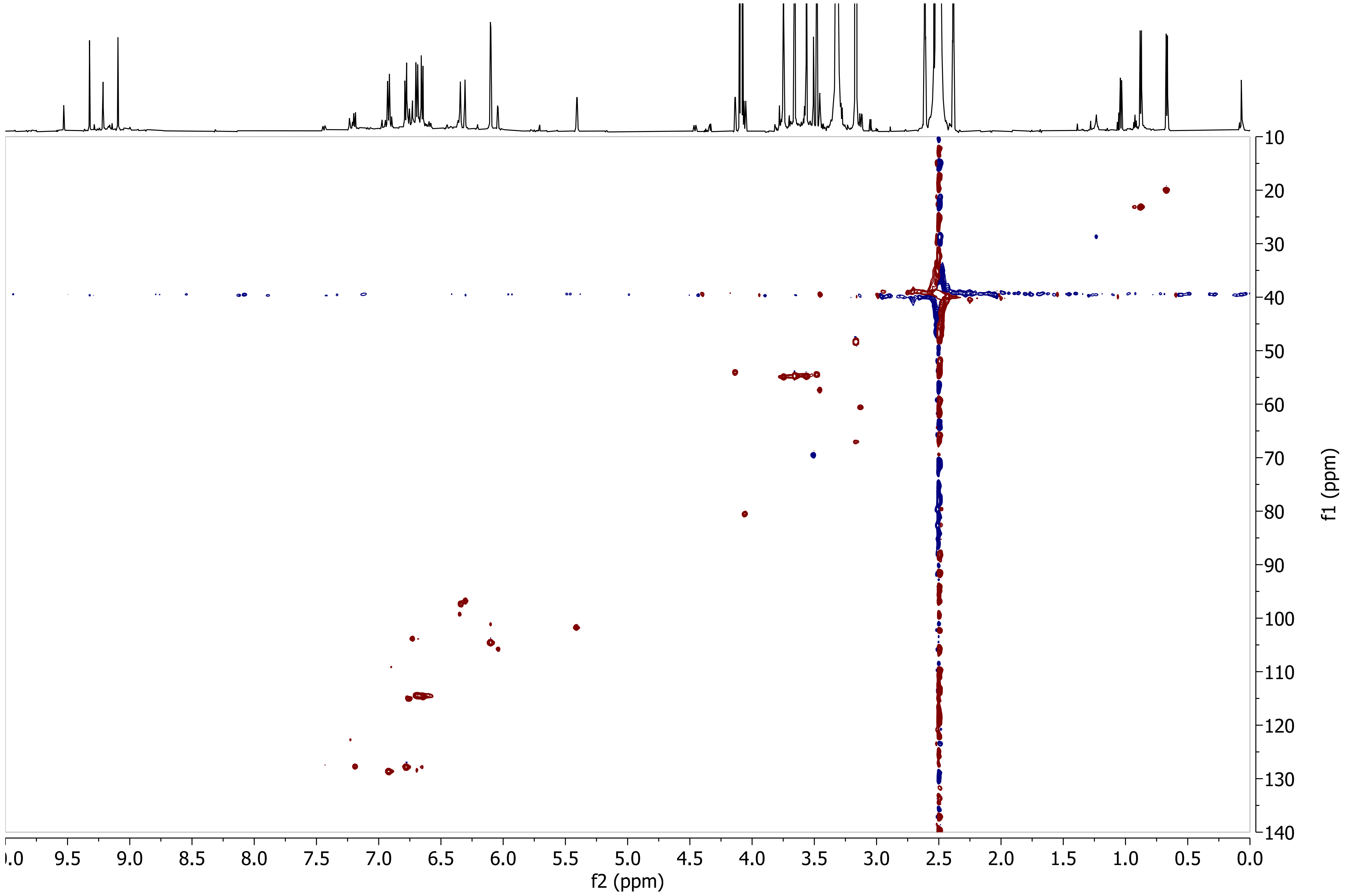

HMBC NMR spectrum of compound **19** in DMSO-d_6

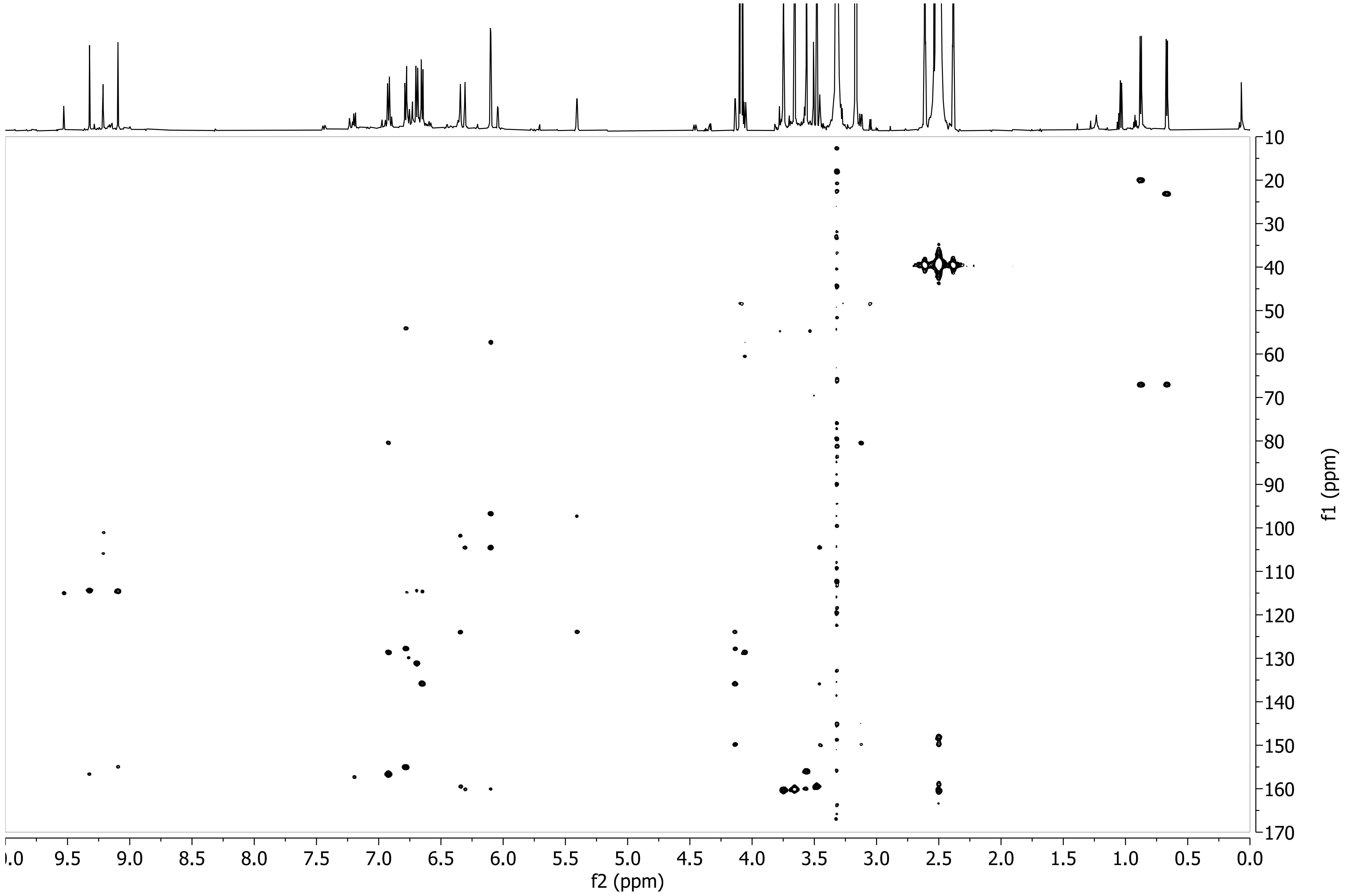

ROESY NMR spectrum of compound **19** in DMSO-d_6

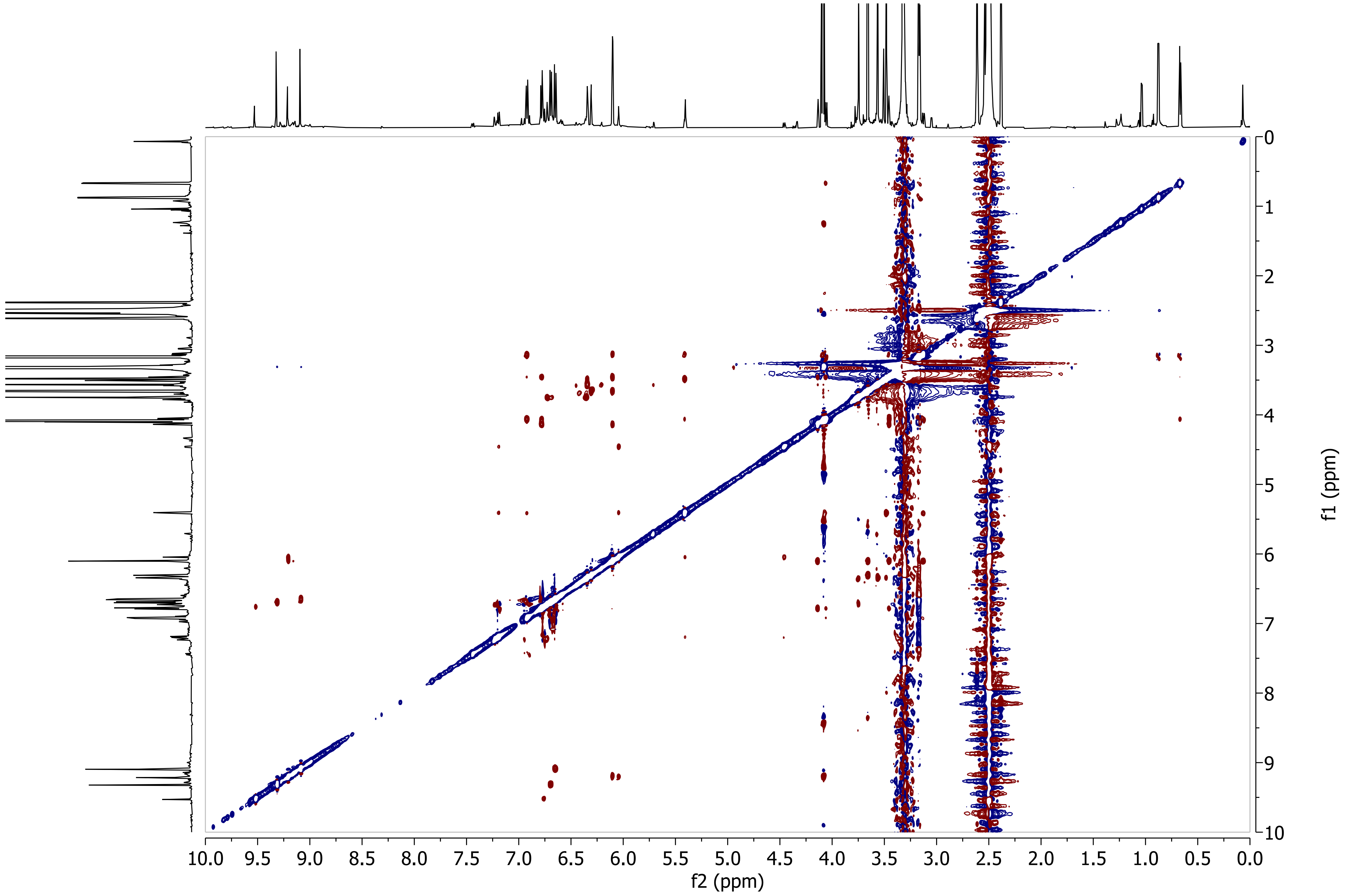

^{1}H NMR spectrum of compound **20** in DMSO-d_6

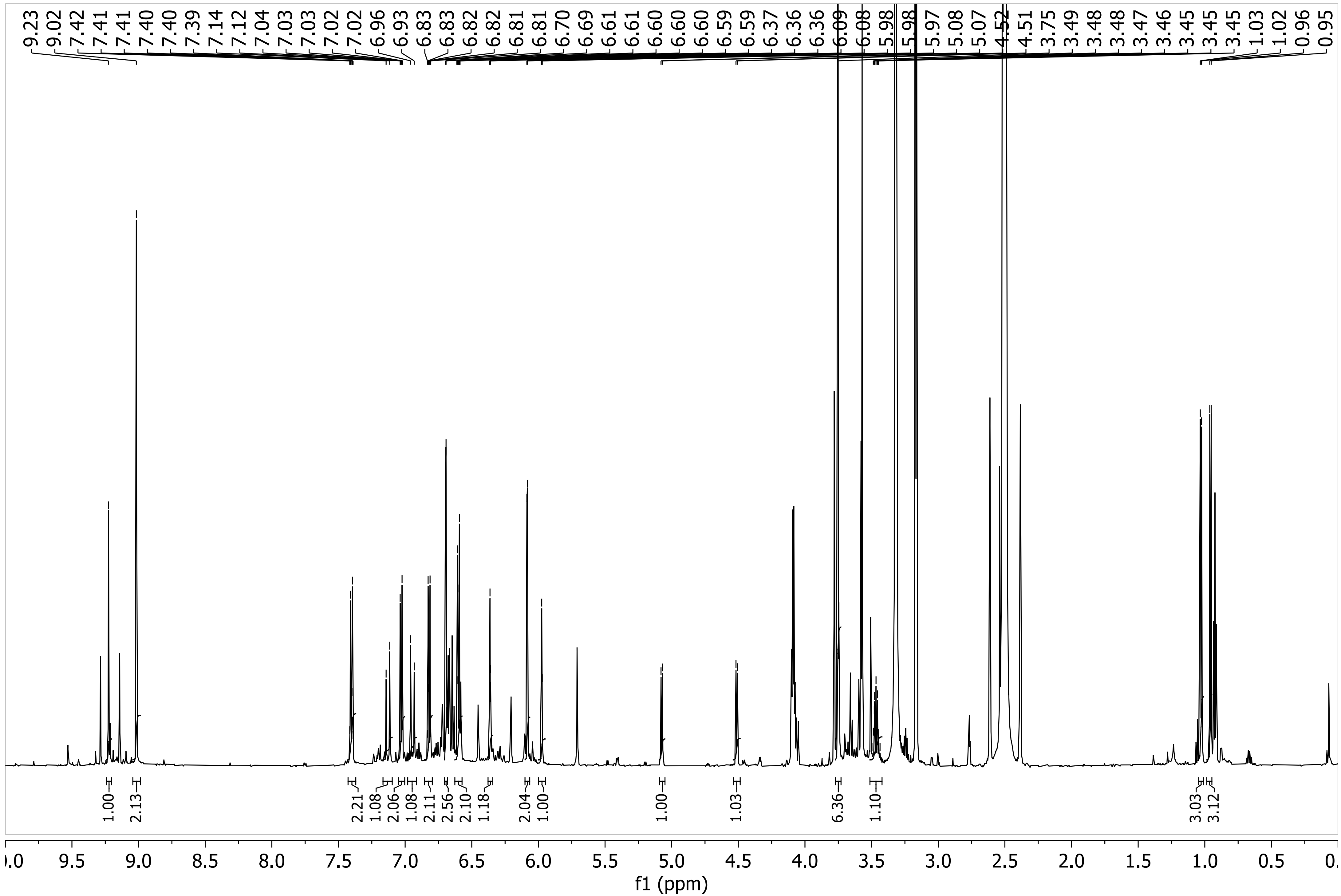

COSY NMR spectrum of compound **20** in DMSO-d_6

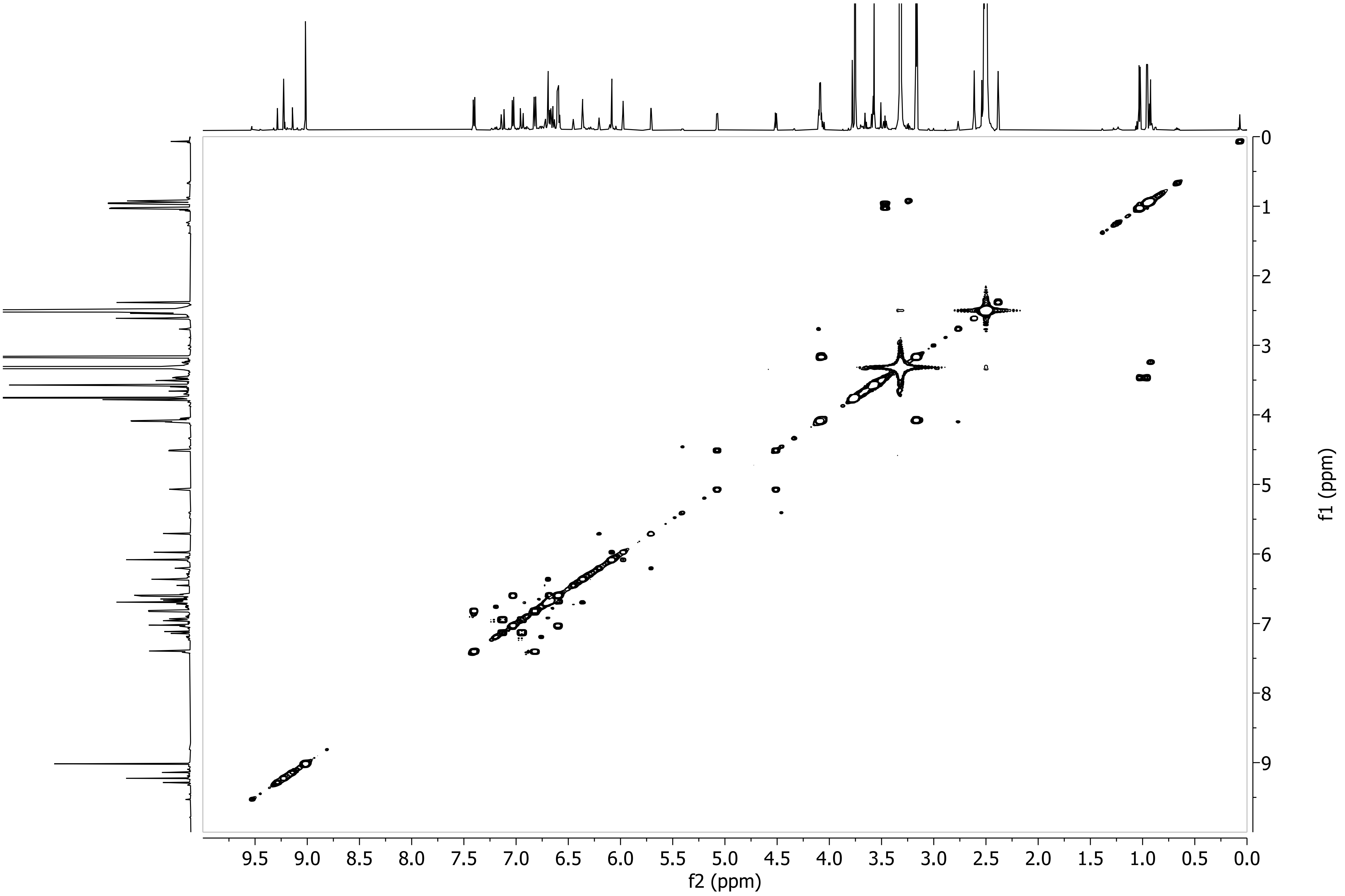

Edited-HSQC NMR spectrum of compound **20** in DMSO-d_6

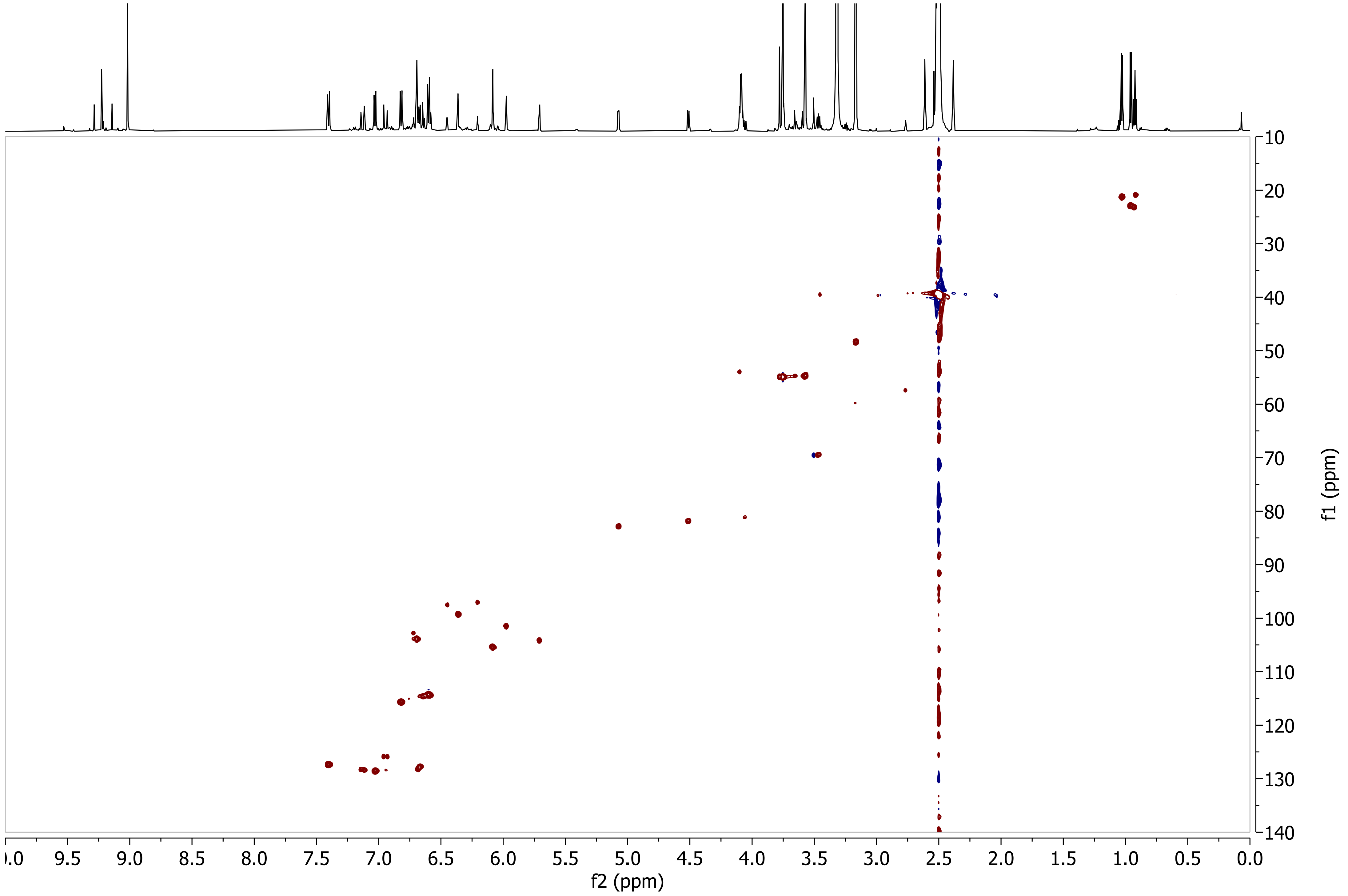

HMBC NMR spectrum of compound **20** in DMSO-d_6

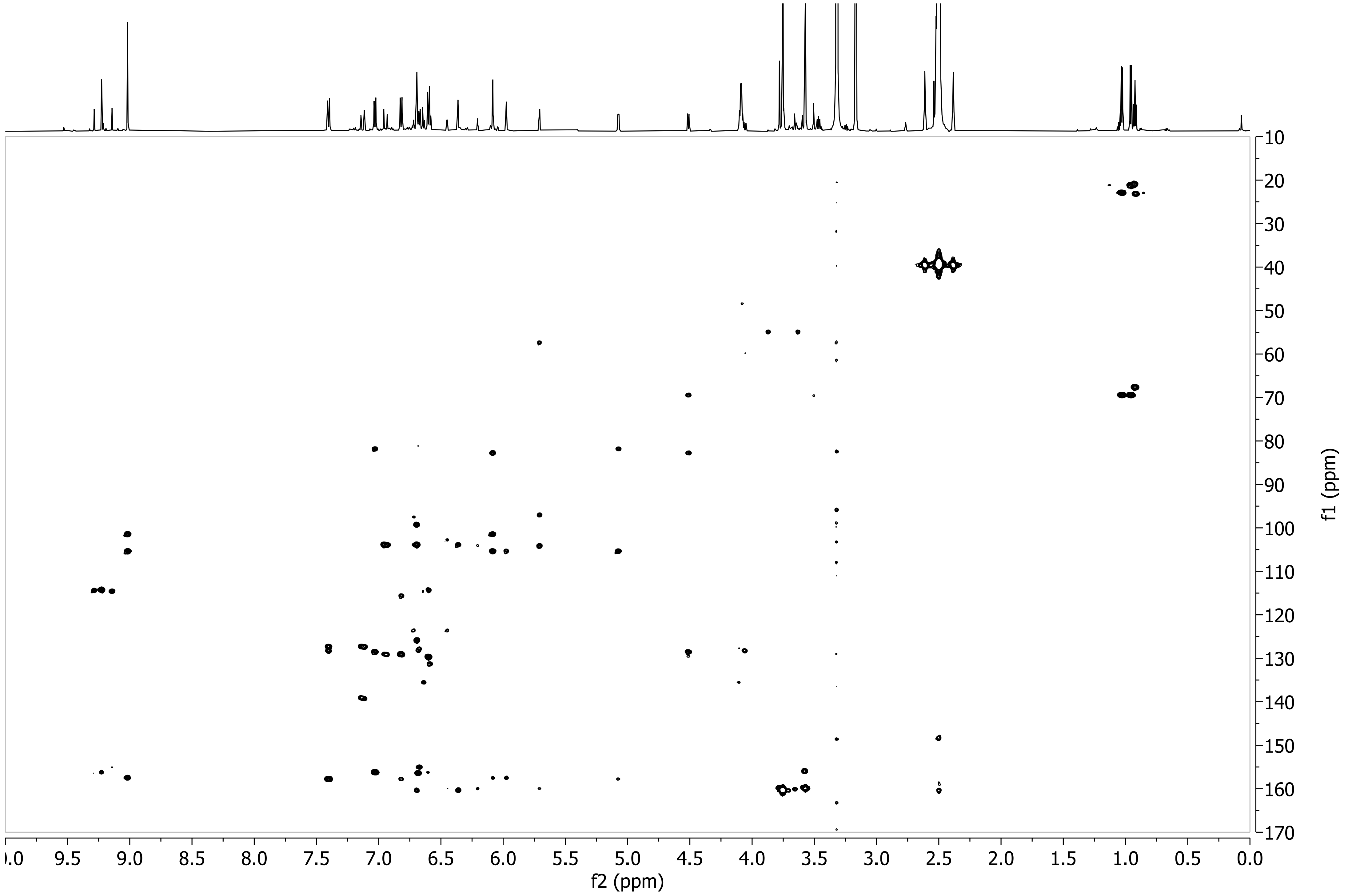

ROESY NMR spectrum of compound **20** in DMSO-d_6

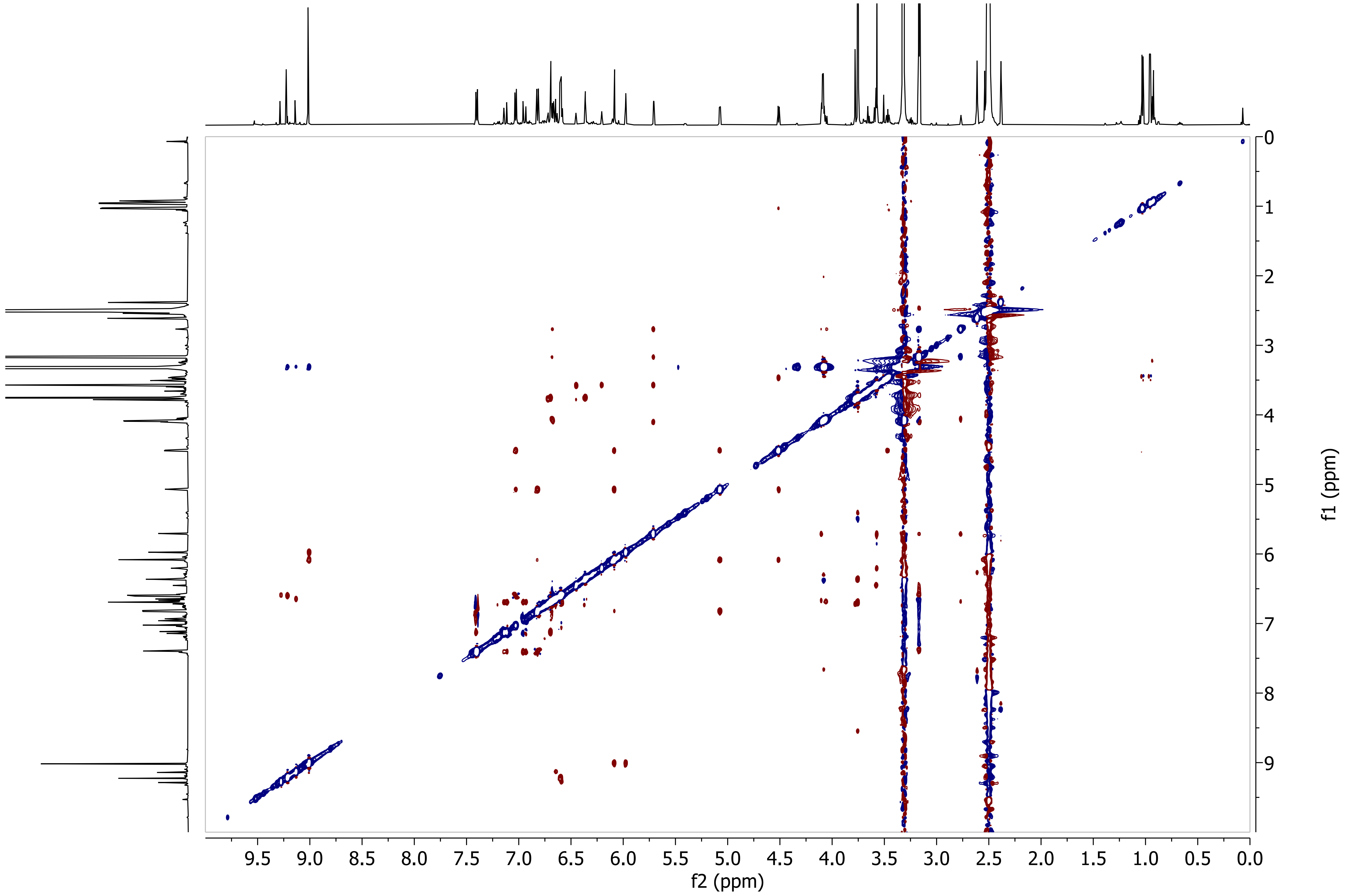

^{1}H NMR spectrum of compound **21** in DMSO-d_6

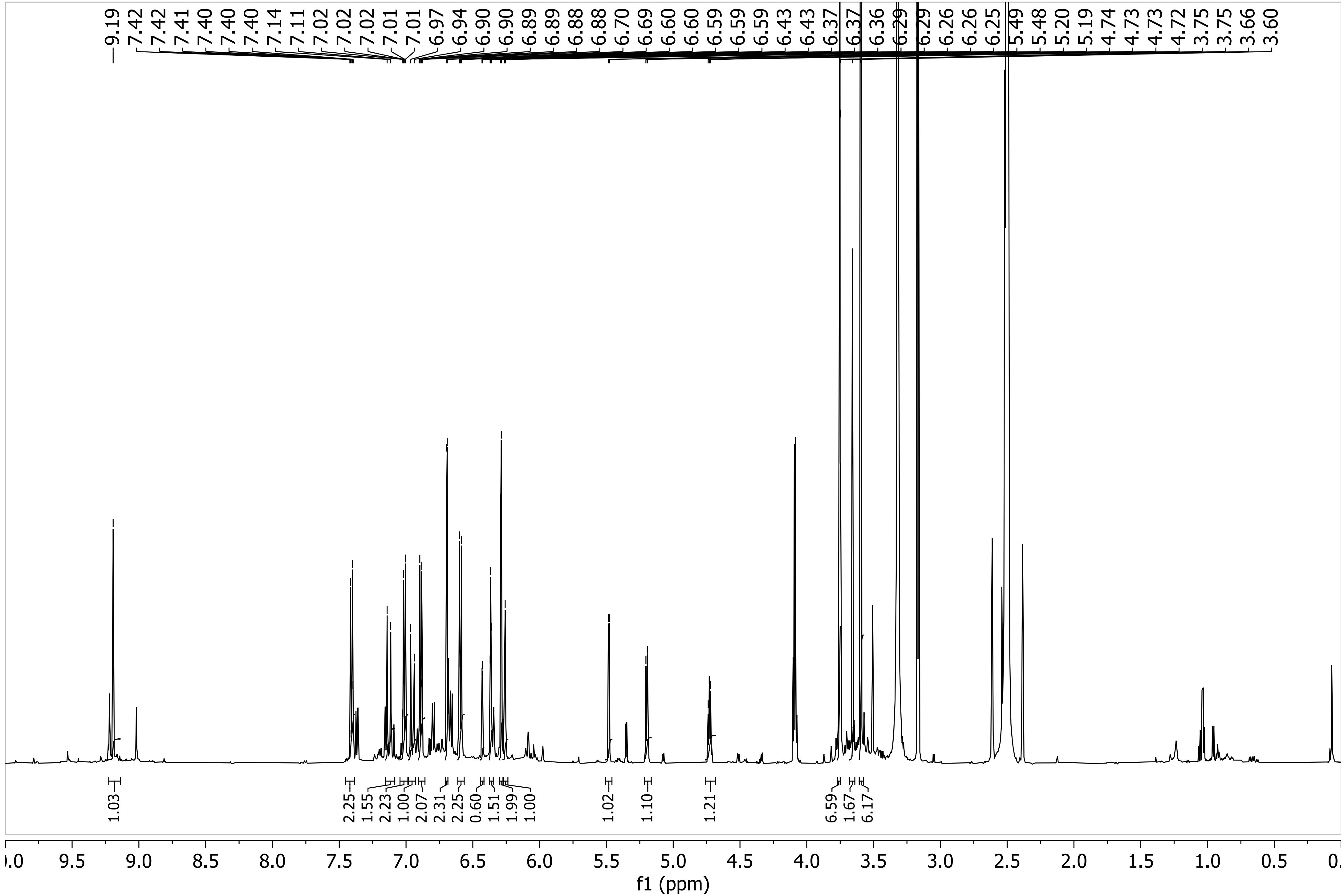

^{1}H NMR spectrum of compound **21** in DMSO-d_6

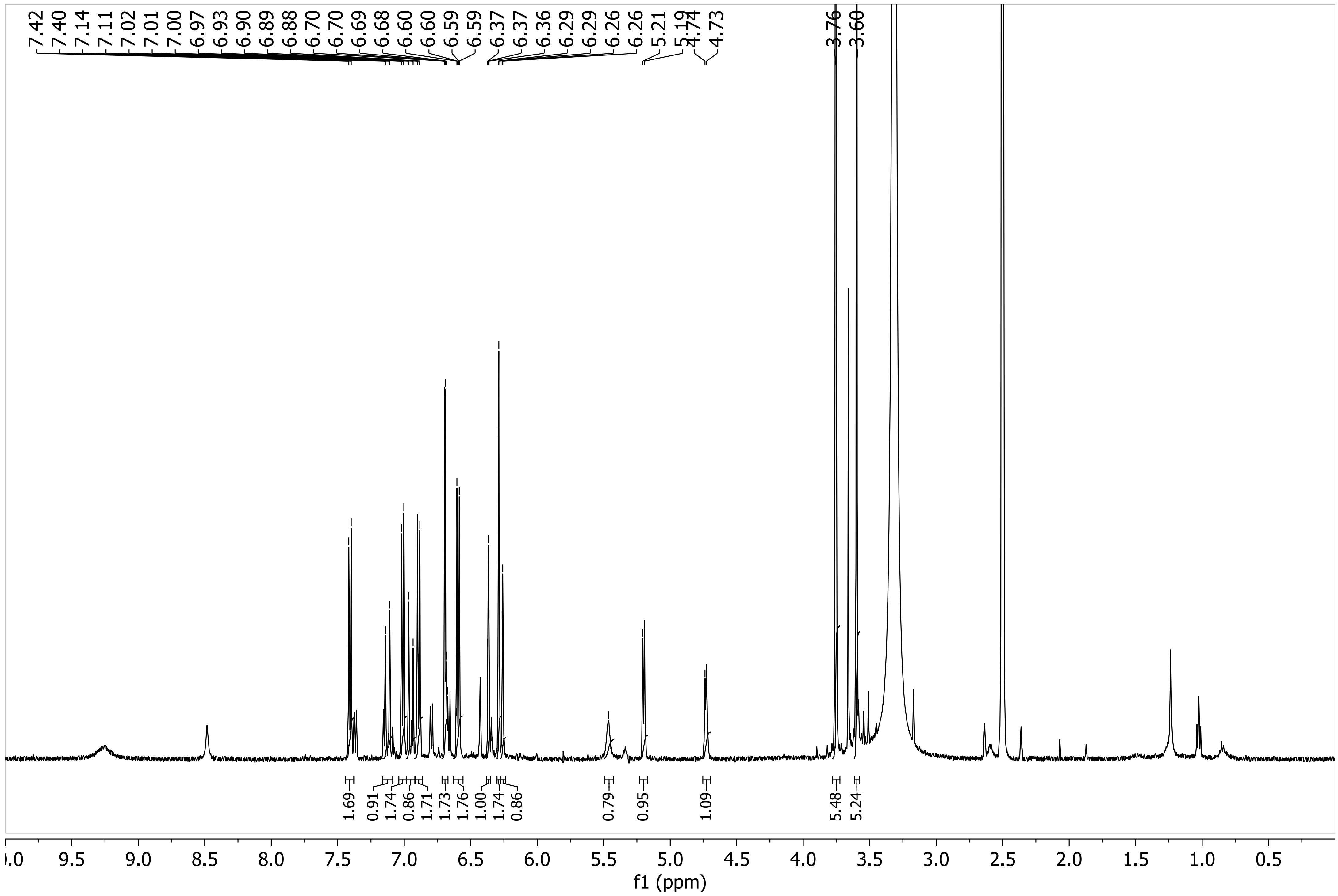

COSY NMR spectrum of compound **21** in DMSO-d_6

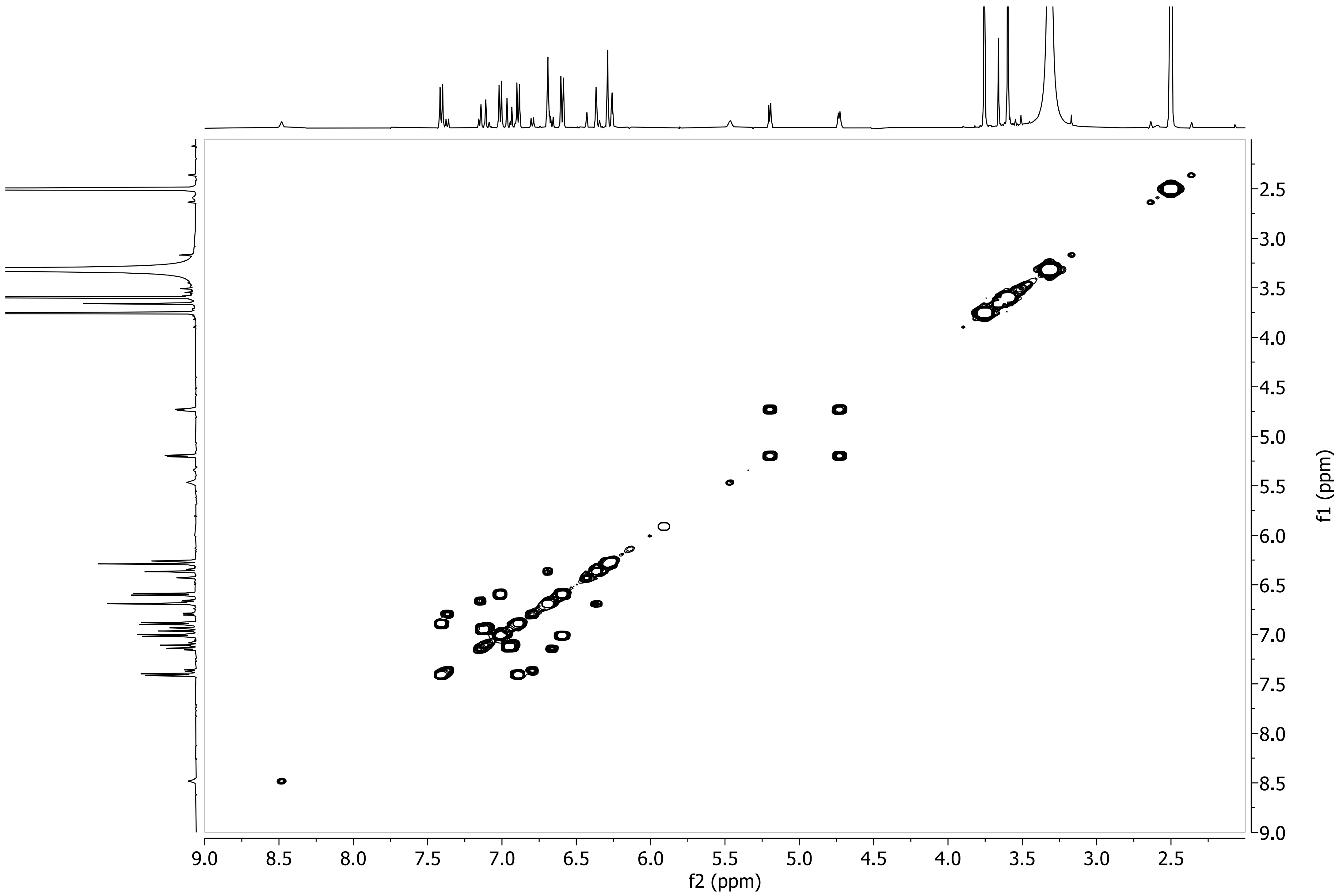

Edited-HSQC NMR spectrum of compound **21** in DMSO-d_6

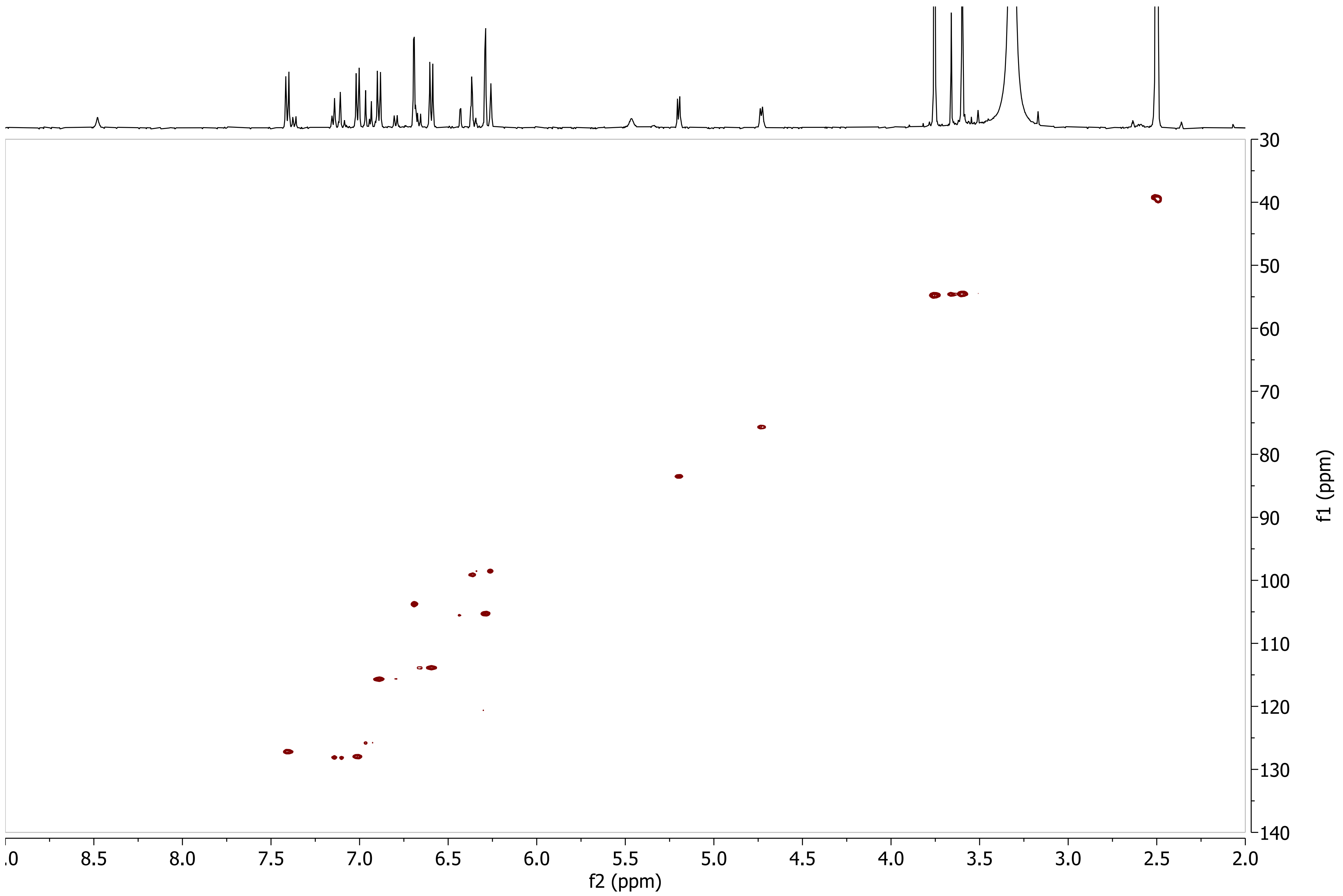

HMBC NMR spectrum of compound **21** in DMSO-d_6

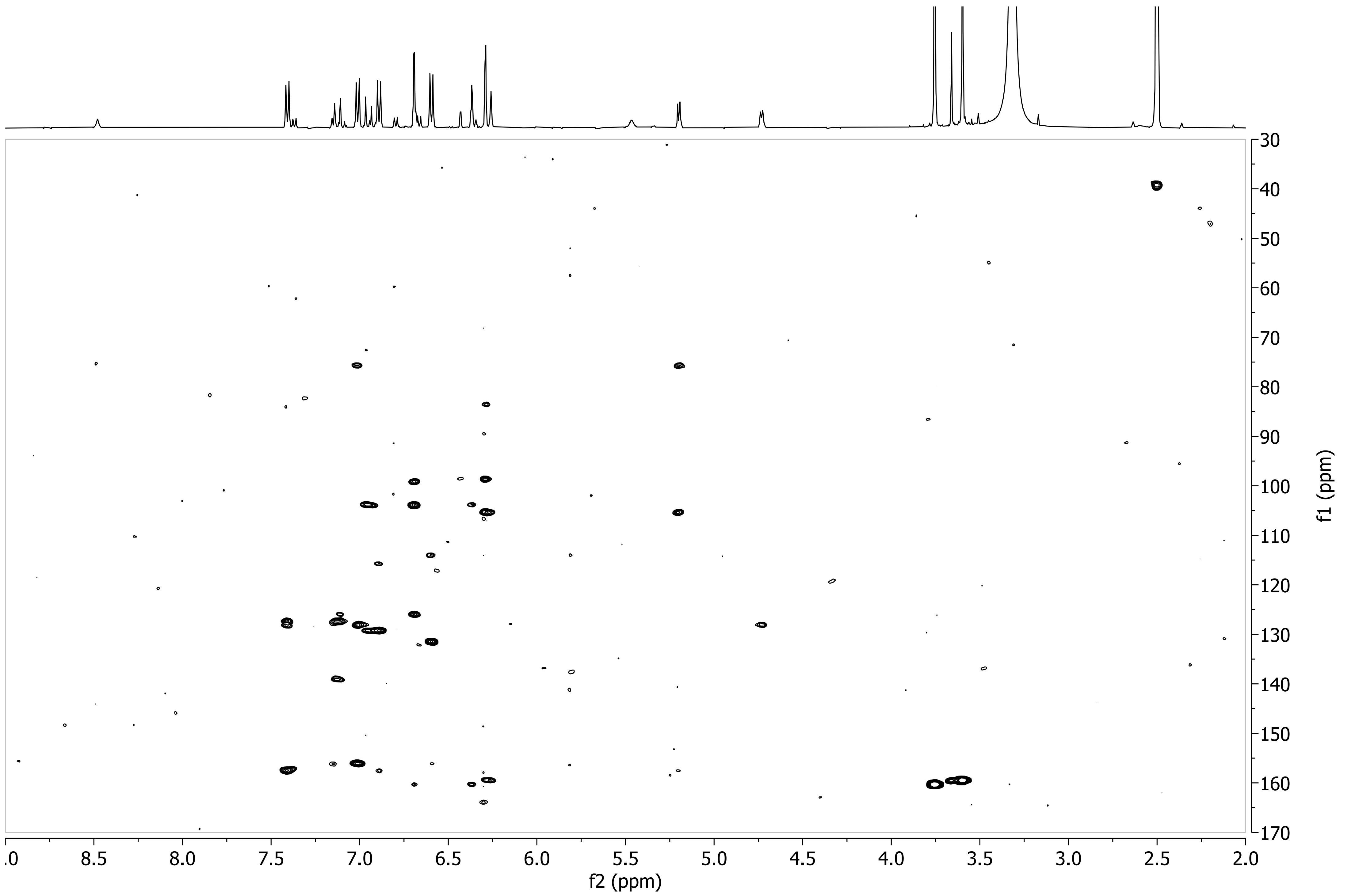

NOESY NMR spectrum of compound **21** in DMSO-d_6

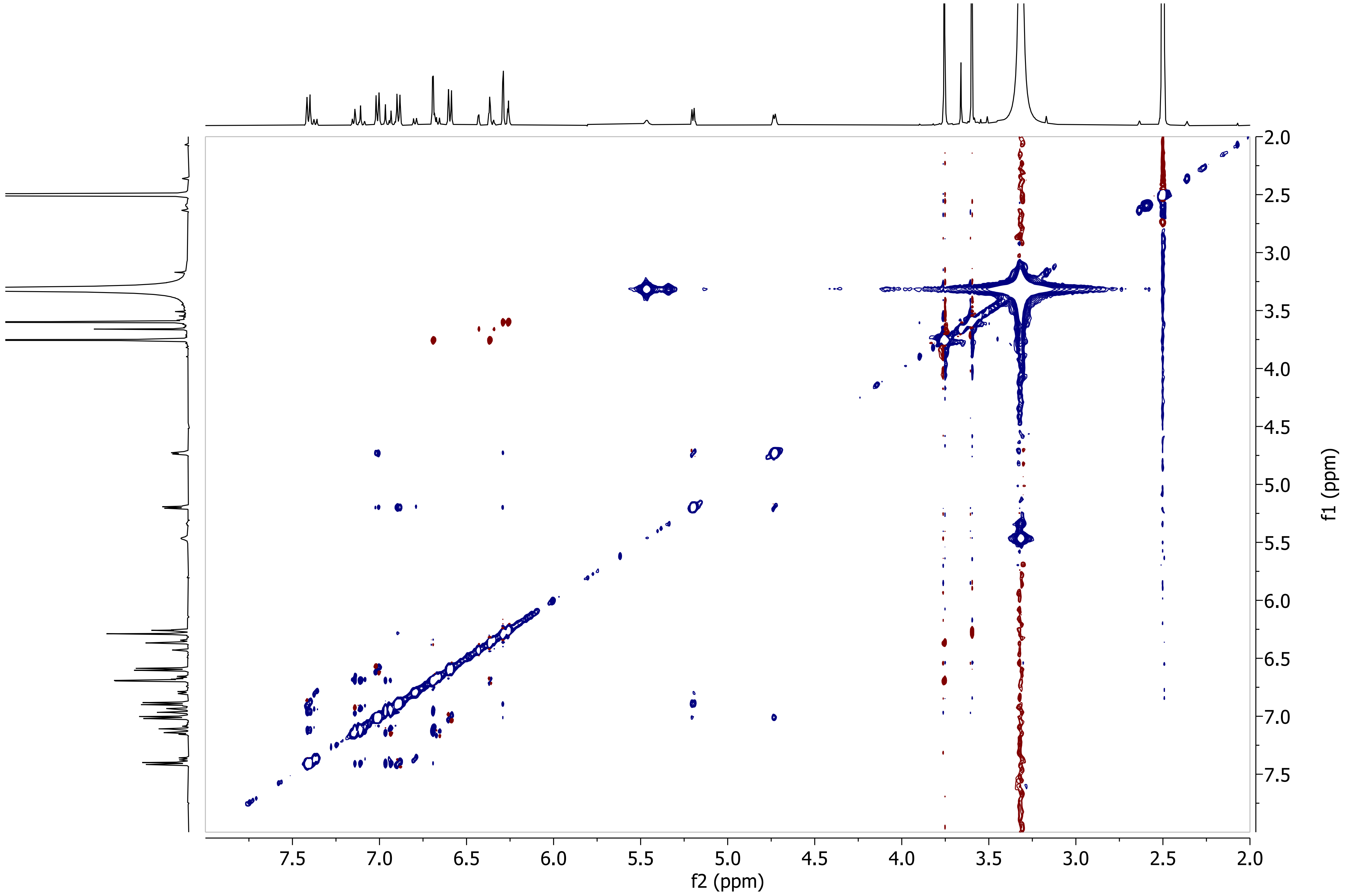

^{1}H NMR spectrum of compound **22** in DMSO-d_6

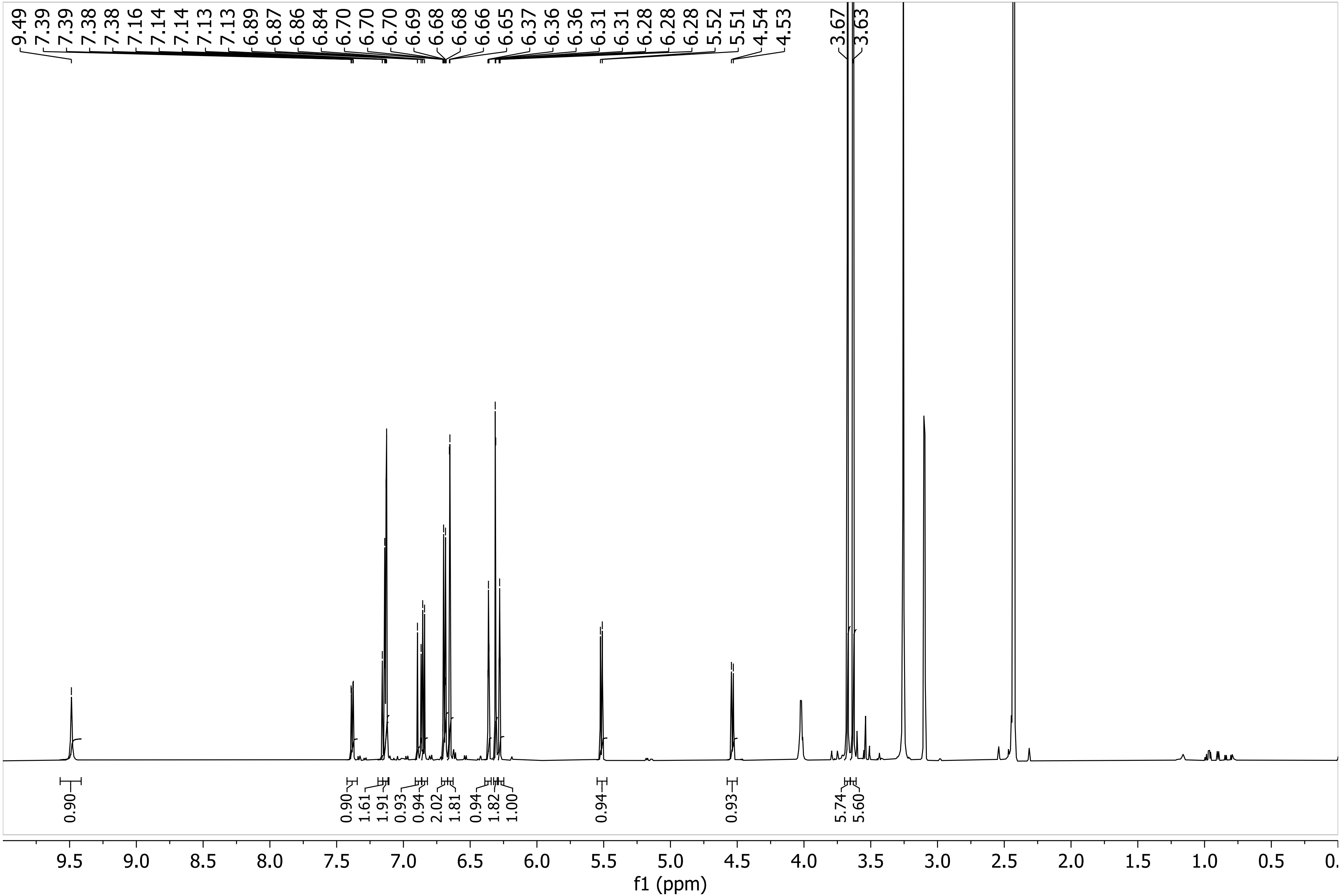

^{1}H NMR spectrum of compound **23** in DMSO-d_6

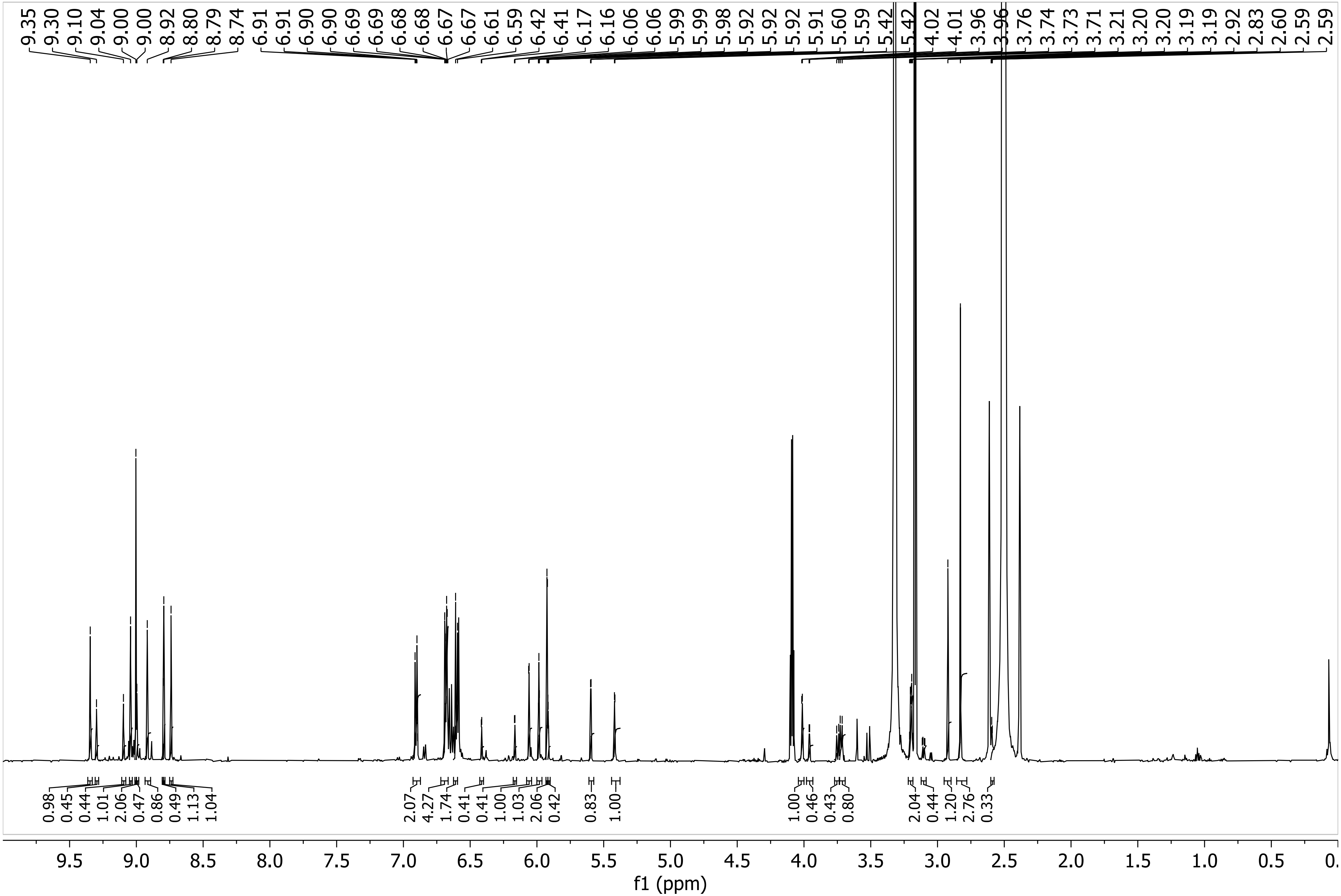

COSY NMR spectrum of compound **23** in DMSO-d_6

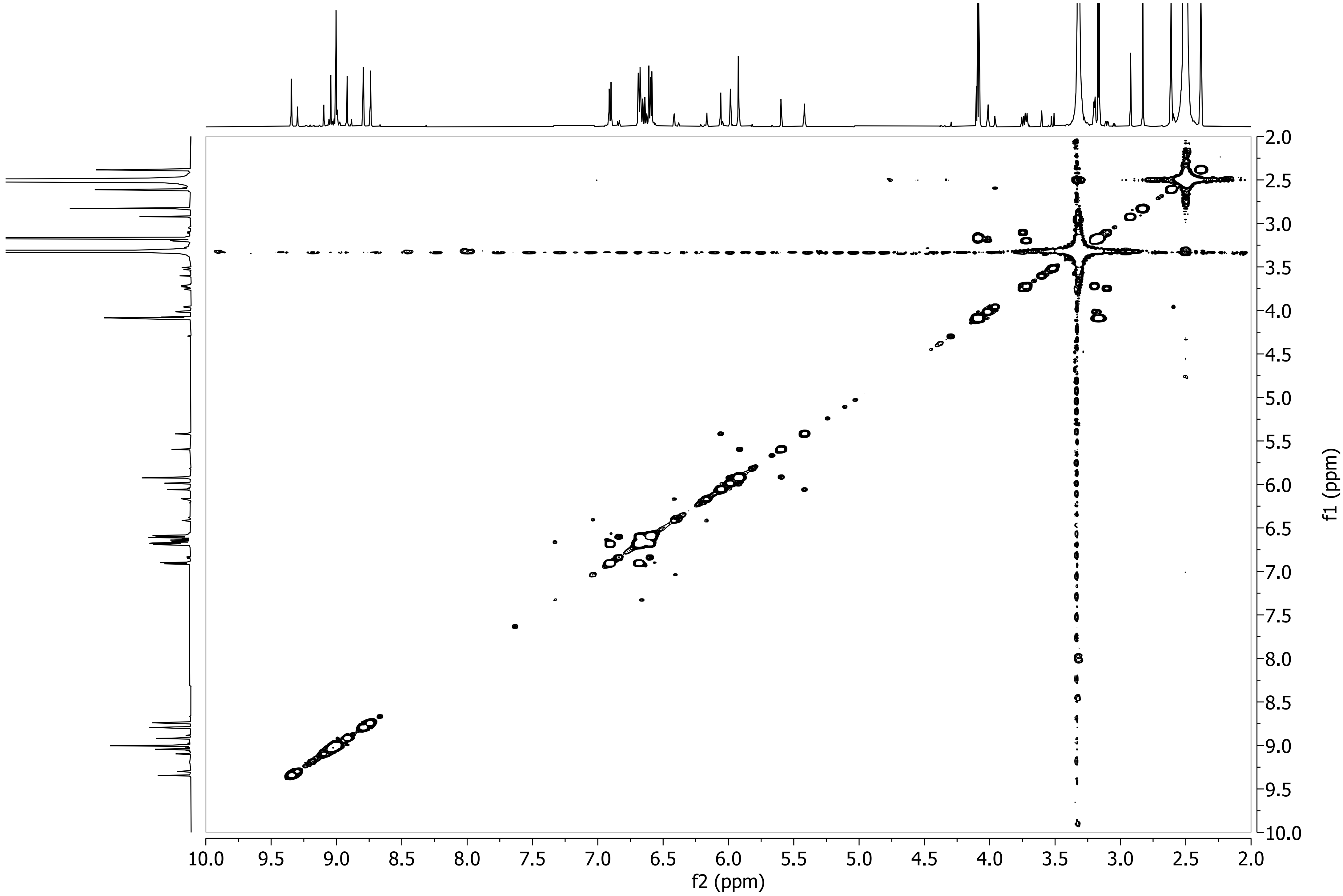

Edited-HSQC NMR spectrum of compound **23** in DMSO-d_6

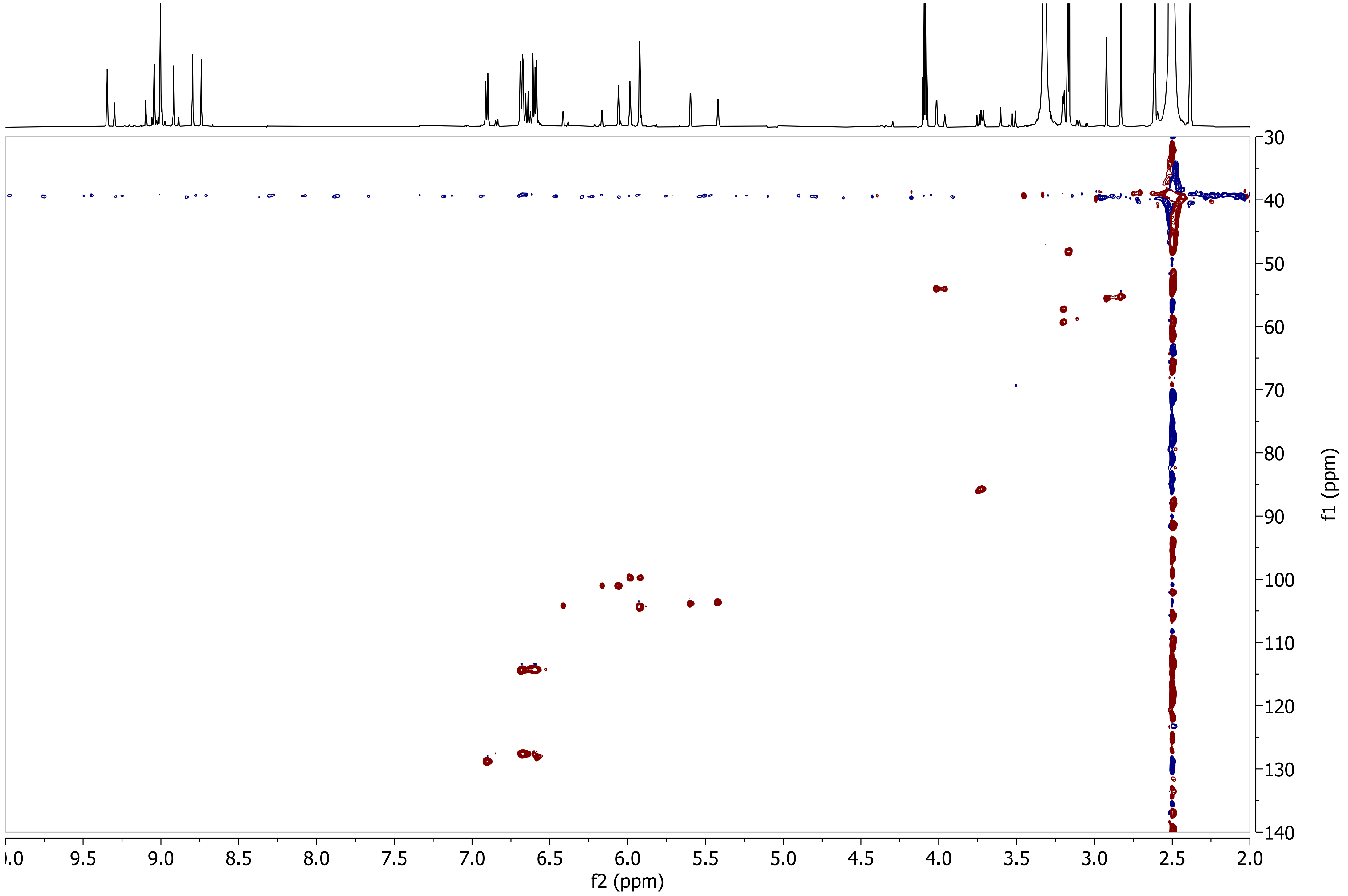

HMBC NMR spectrum of compound **23** in DMSO-d_6

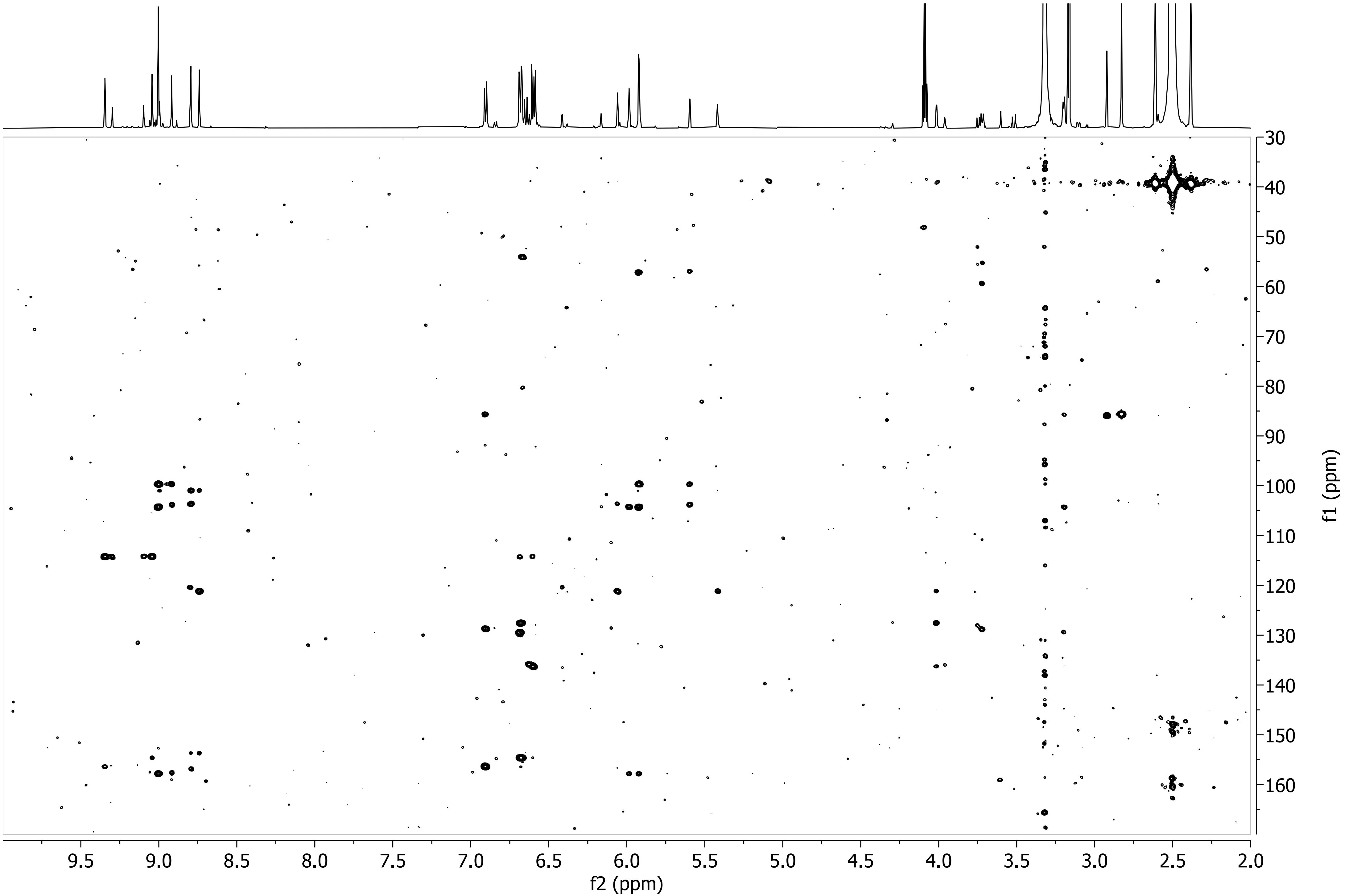

ROESY NMR spectrum of compound **23** in DMSO-d_6

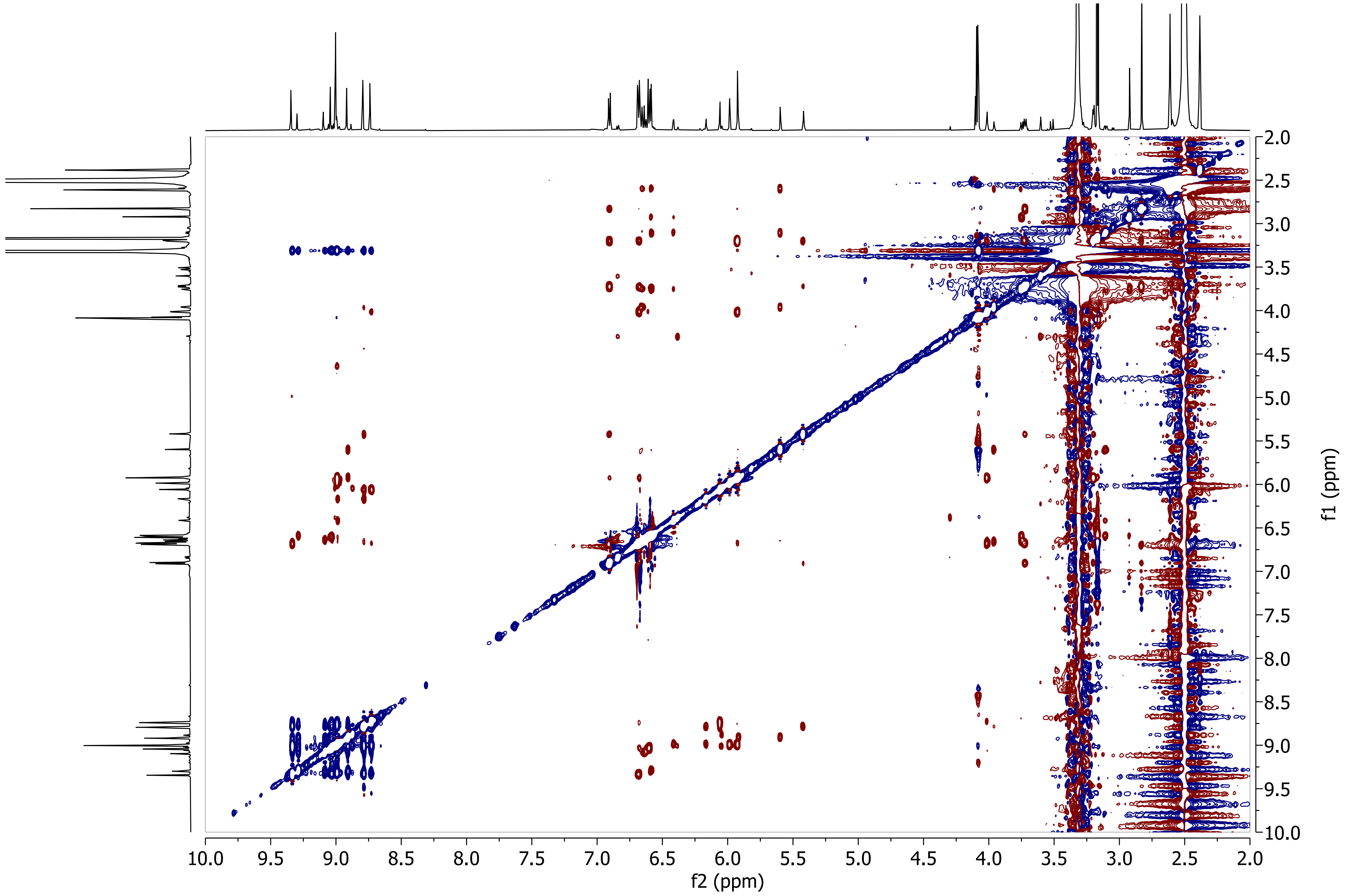

^{1}H NMR spectrum of compound **24** in DMSO-d_6

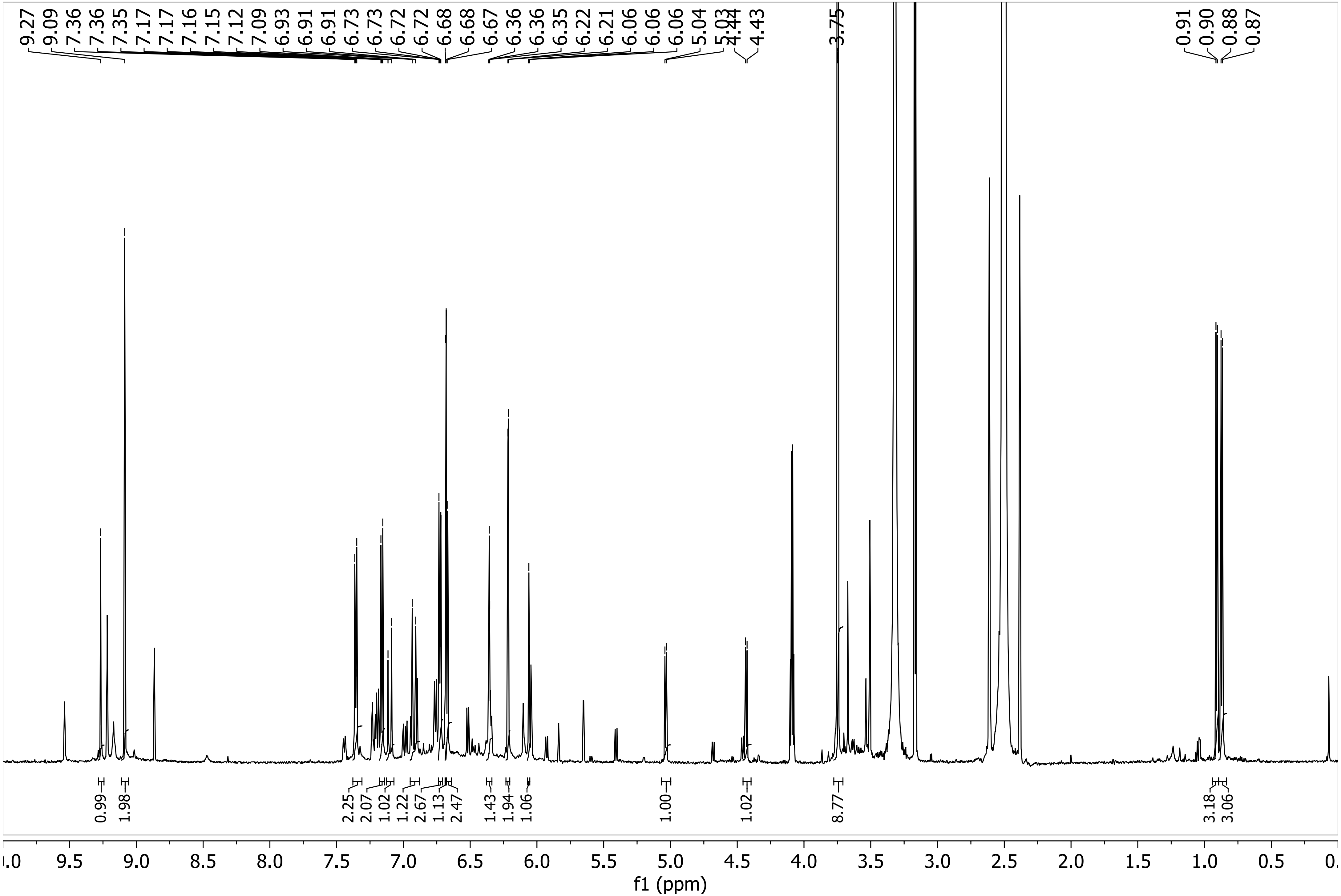

COSY NMR spectrum of compound **24** in DMSO-d_6

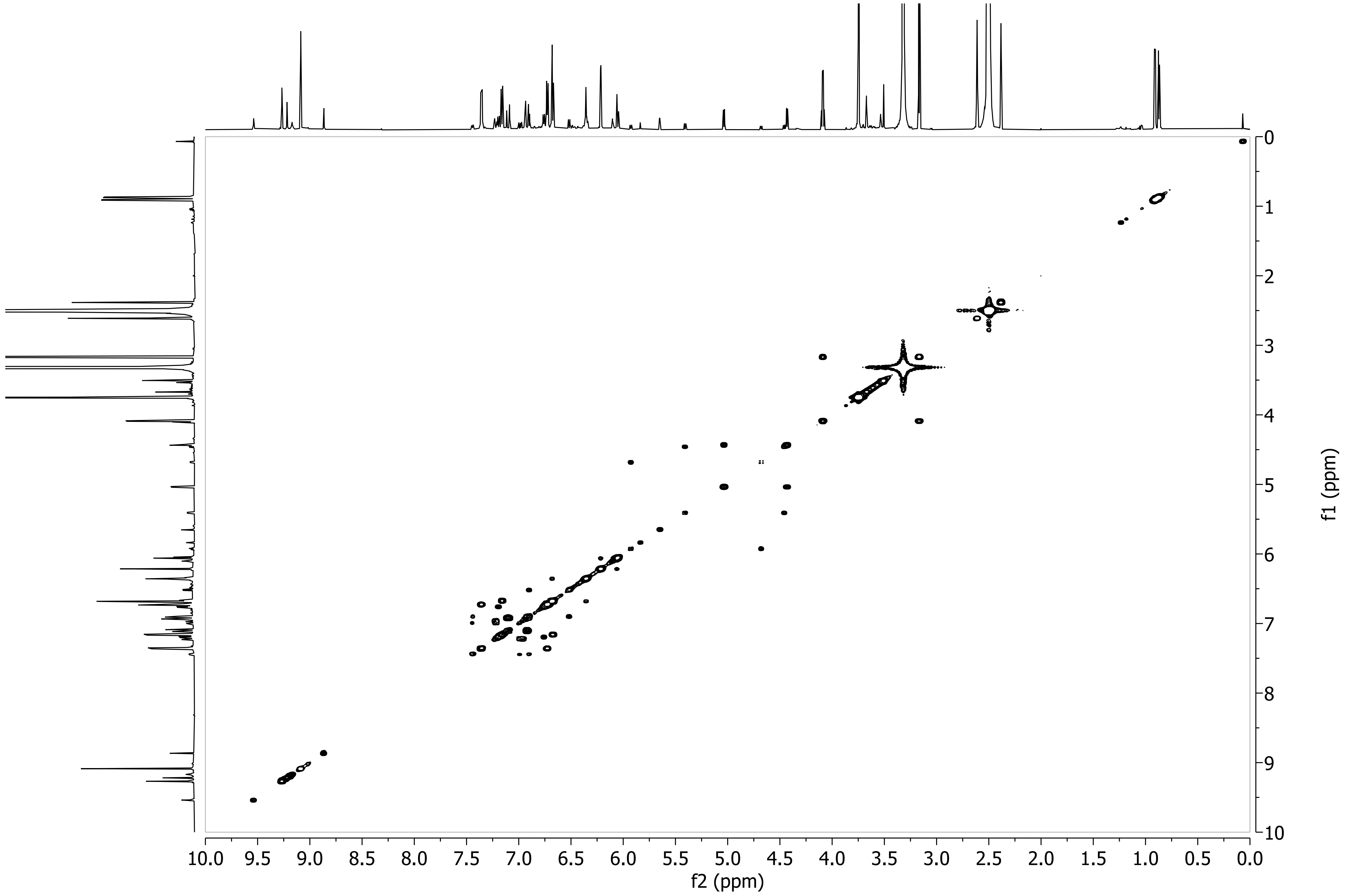

Edited-HSQC NMR spectrum of compound **24** in DMSO-d_6

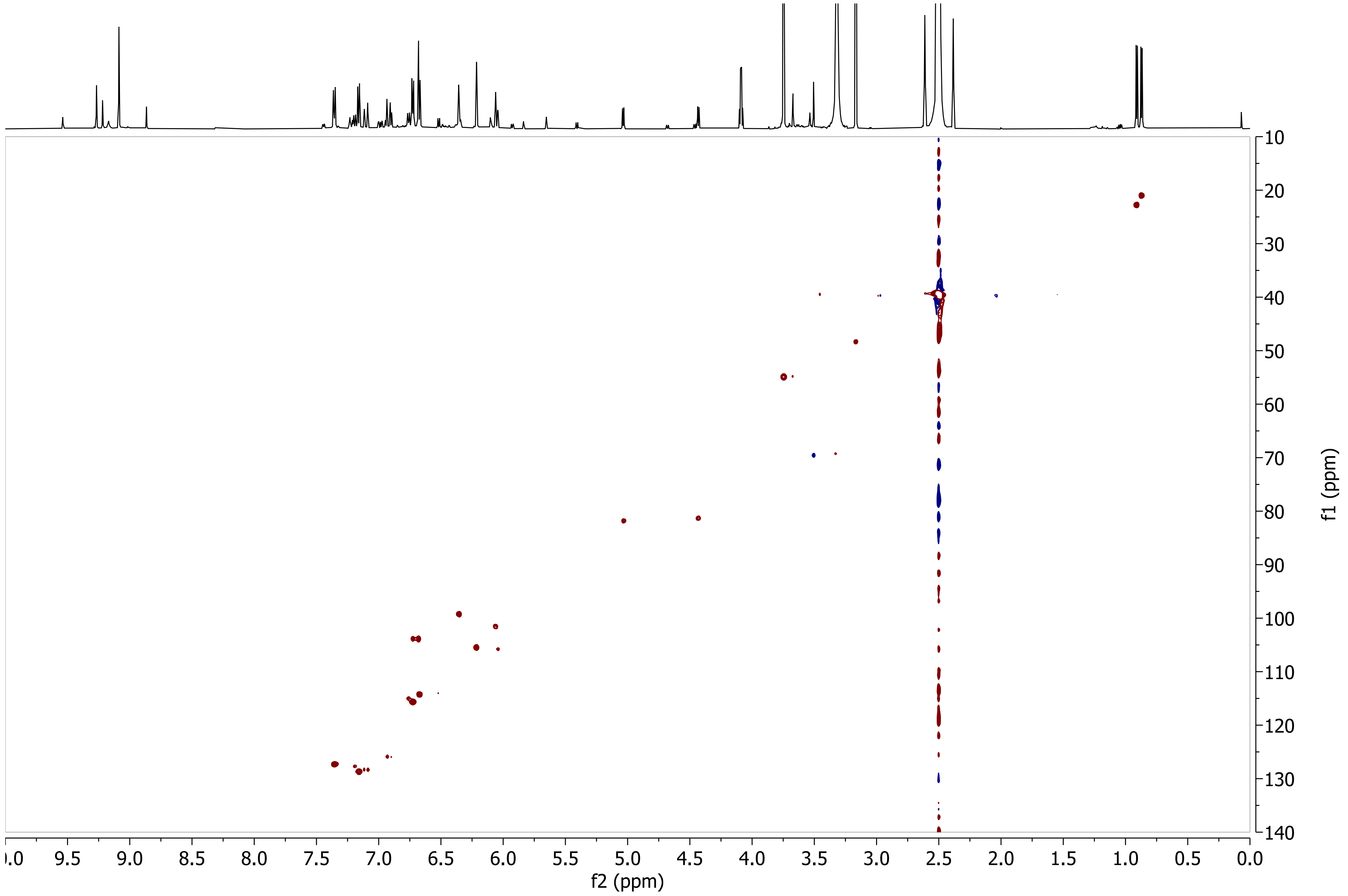

HMBC NMR spectrum of compound **24** in DMSO-d_6

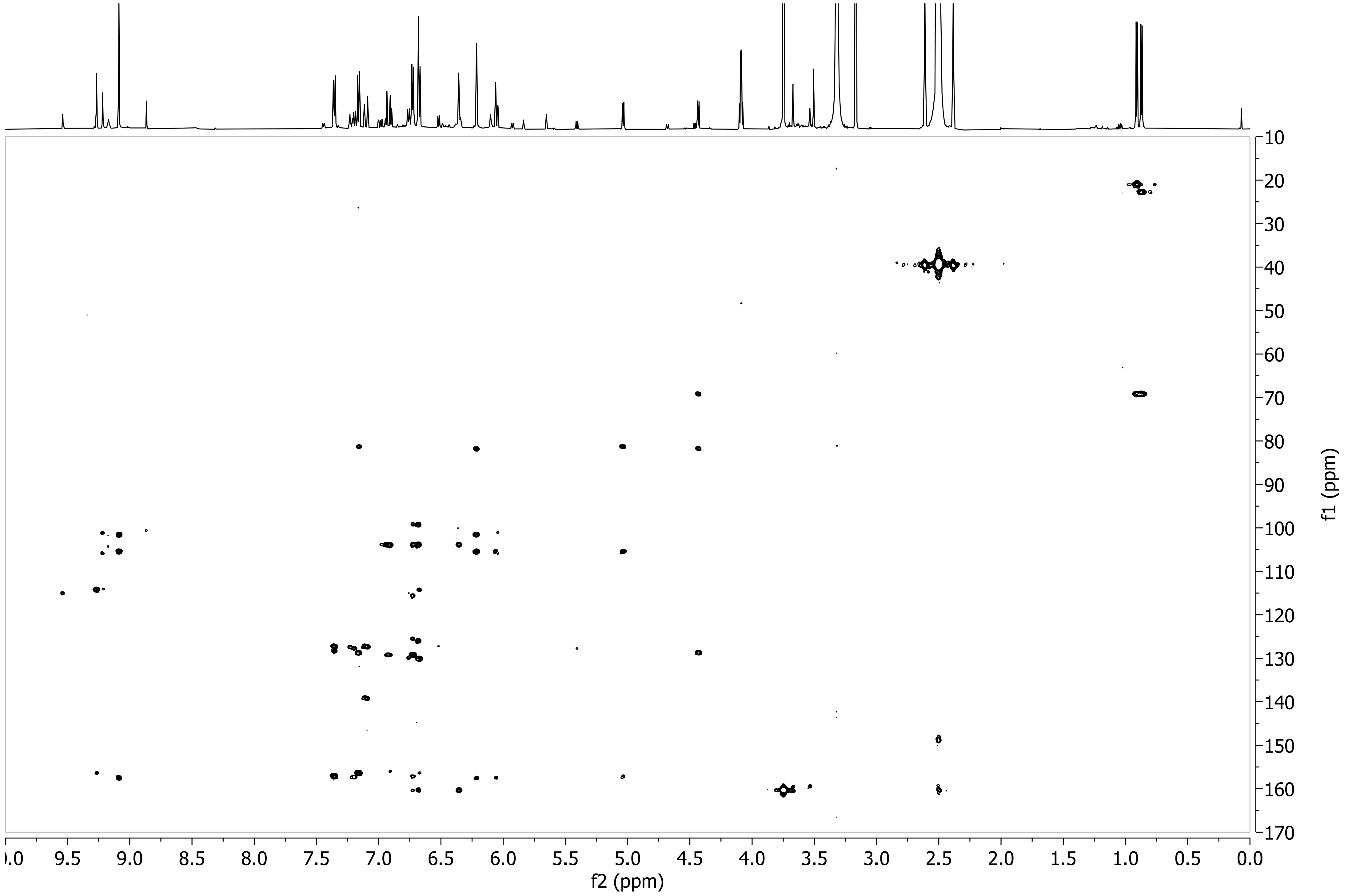

ROESY NMR spectrum of compound **24** in DMSO-d_6

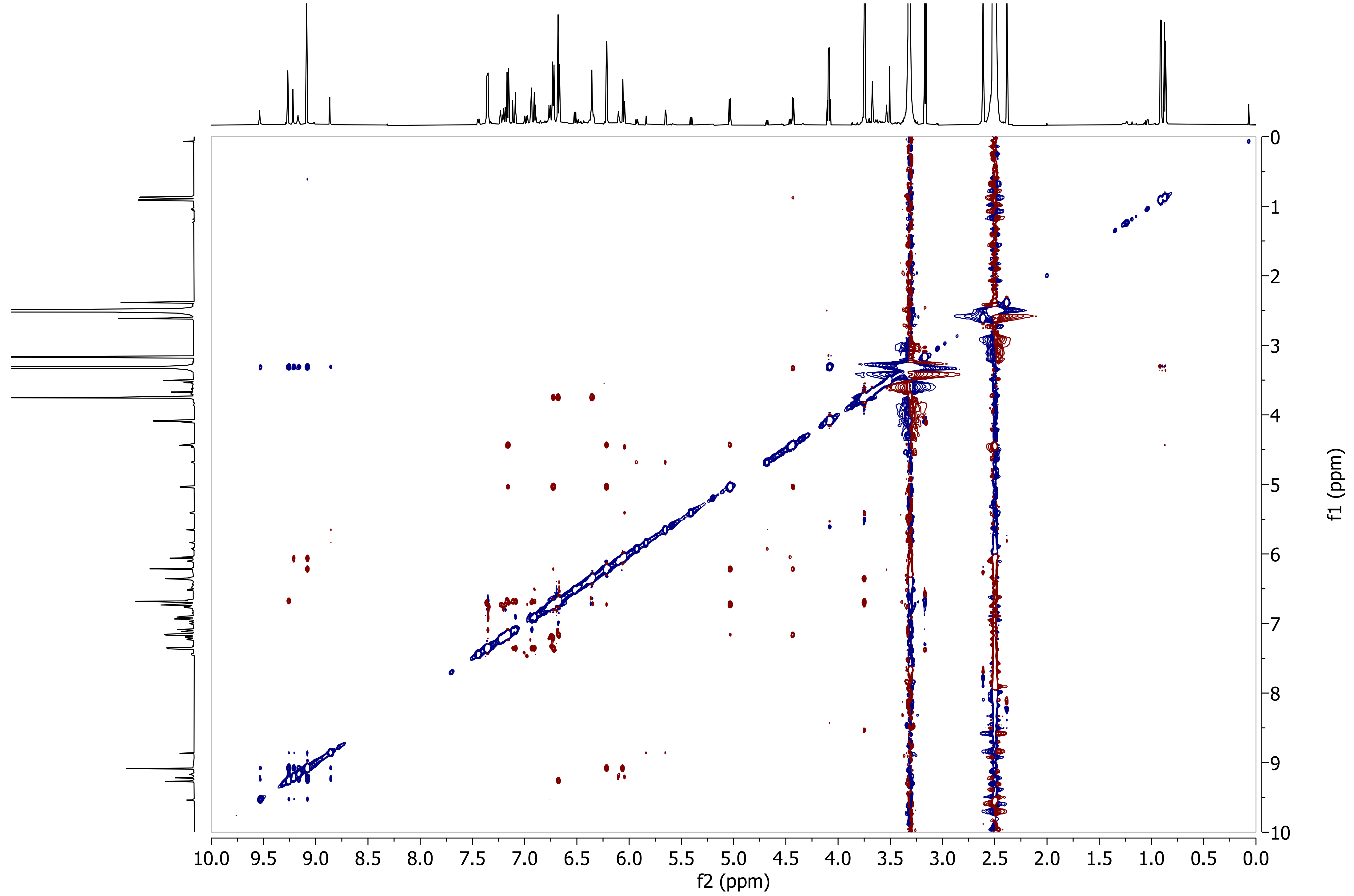

^{1}H NMR spectrum of compound **25** in DMSO-d_6

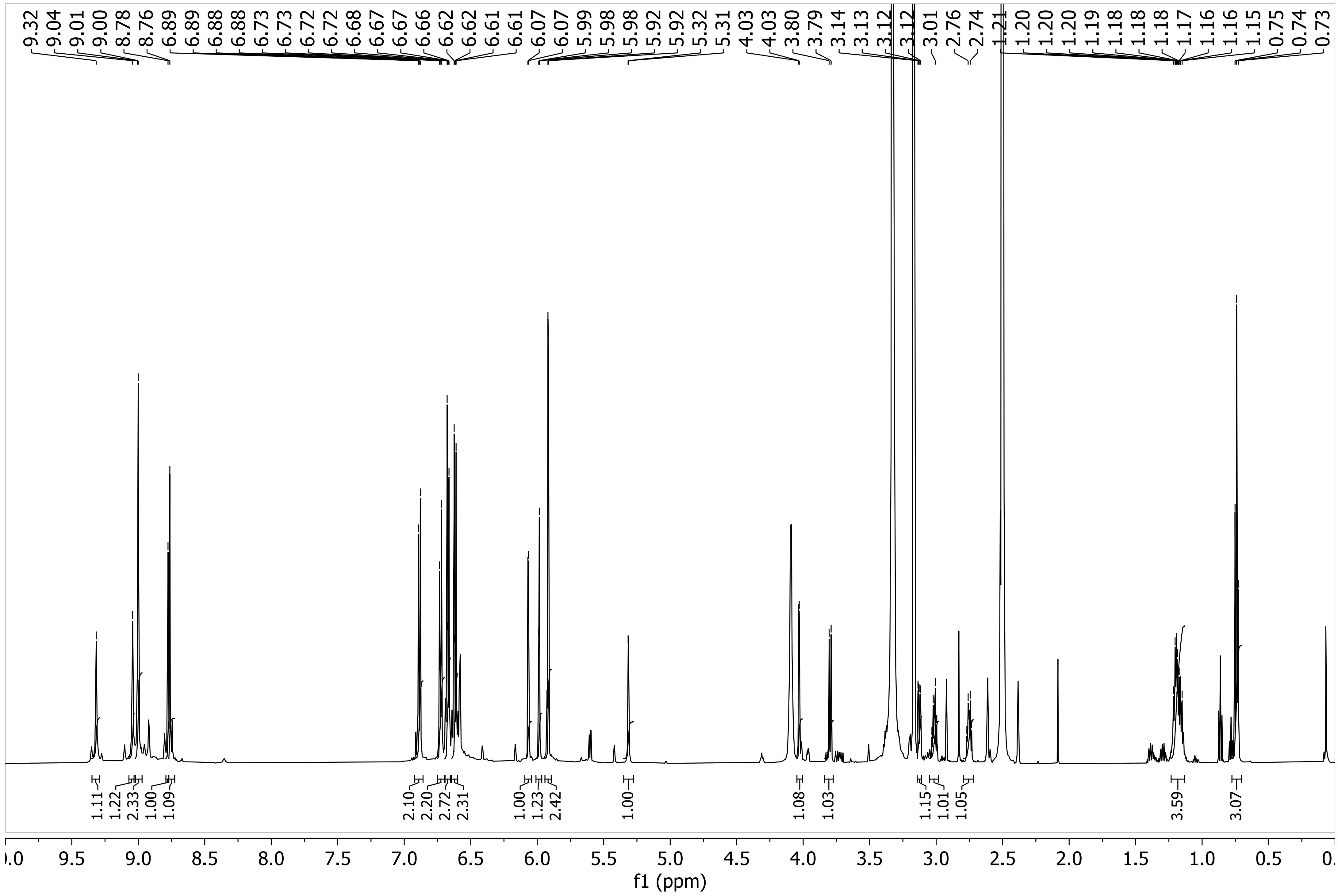

COSY NMR spectrum of compound **25** in DMSO-d_6

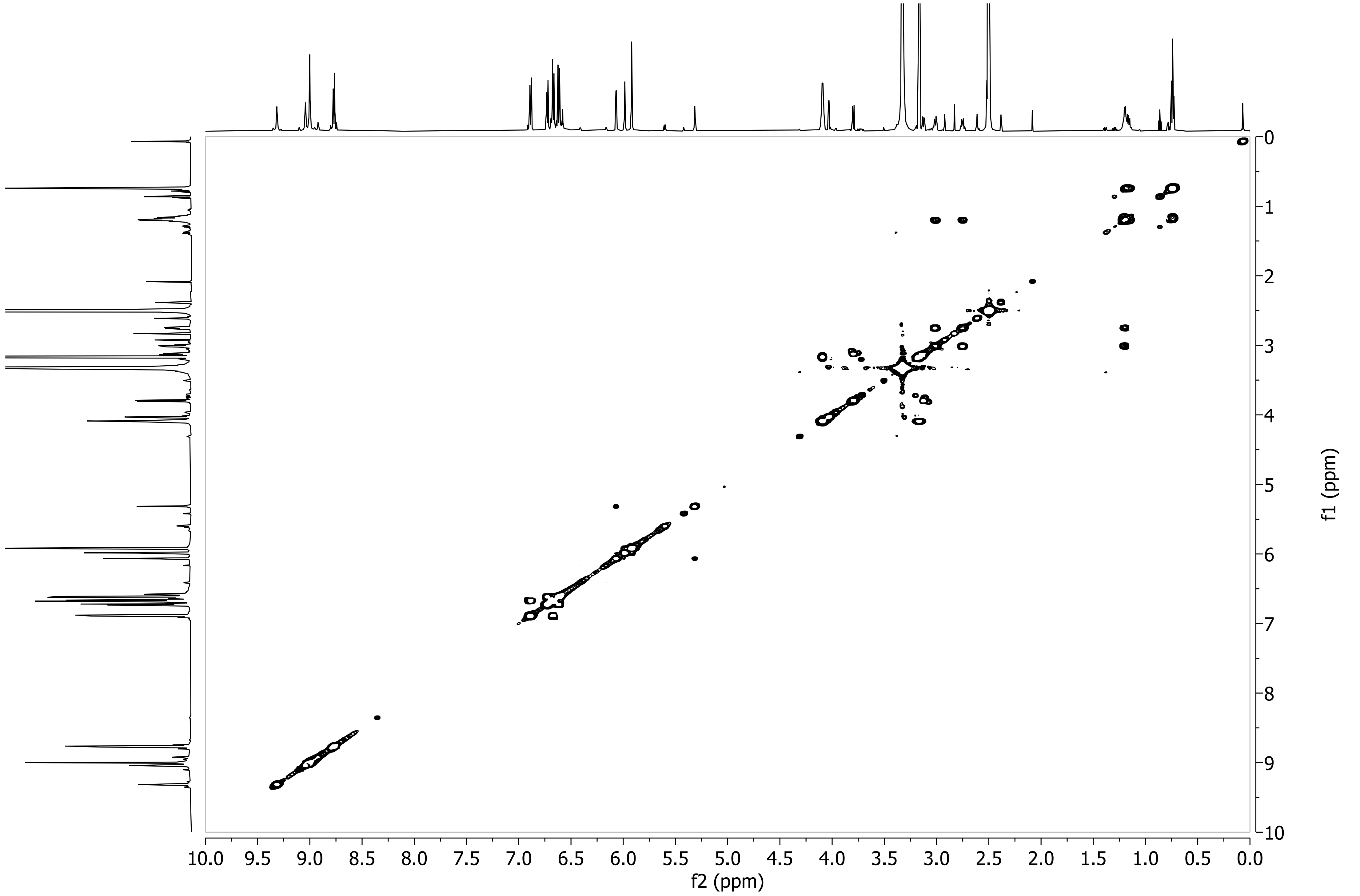

^{13}C-DEPTQ NMR spectrum of compound **25** in DMSO-d_6

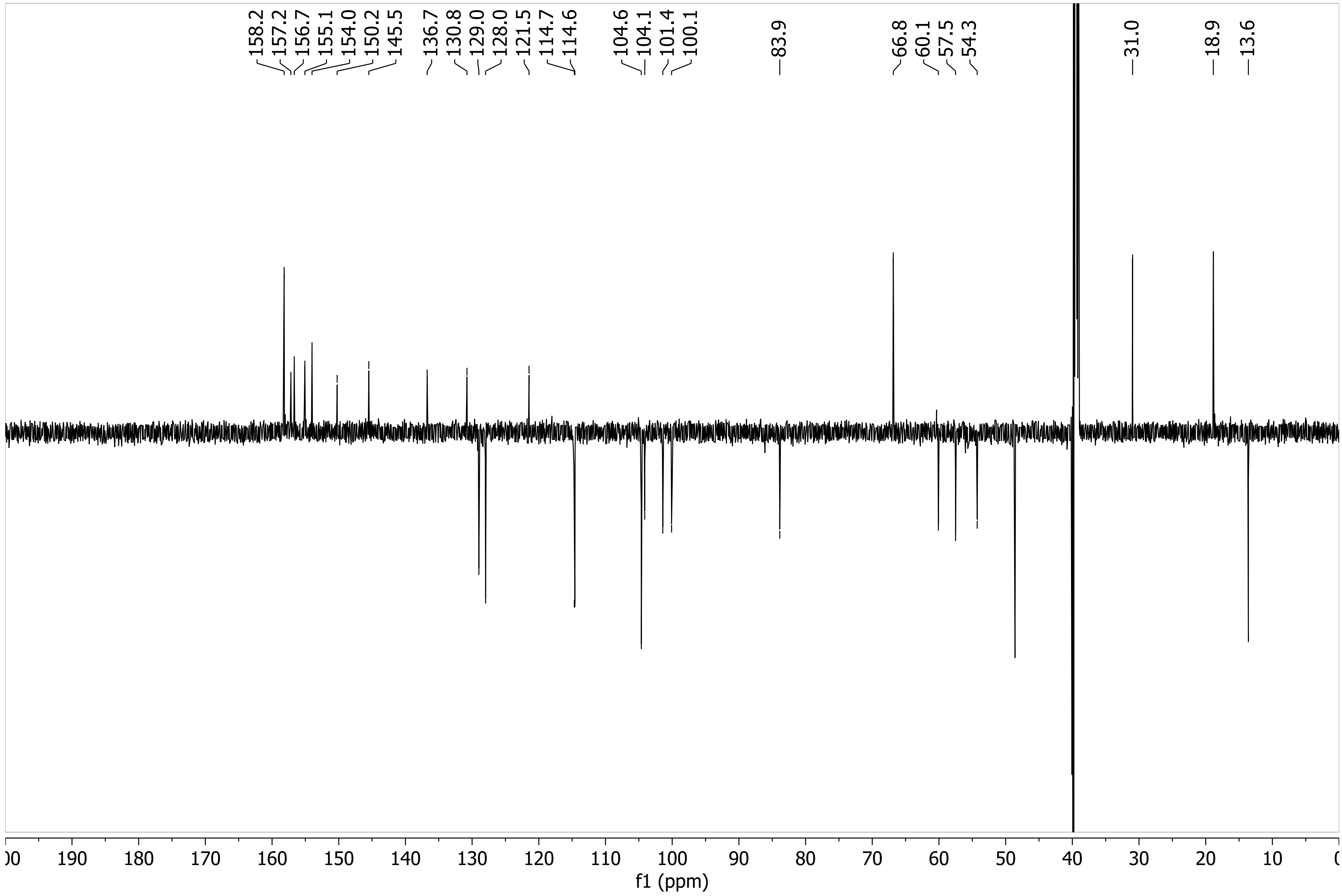

Edited-HSQC NMR spectrum of compound **25** in DMSO-d_6

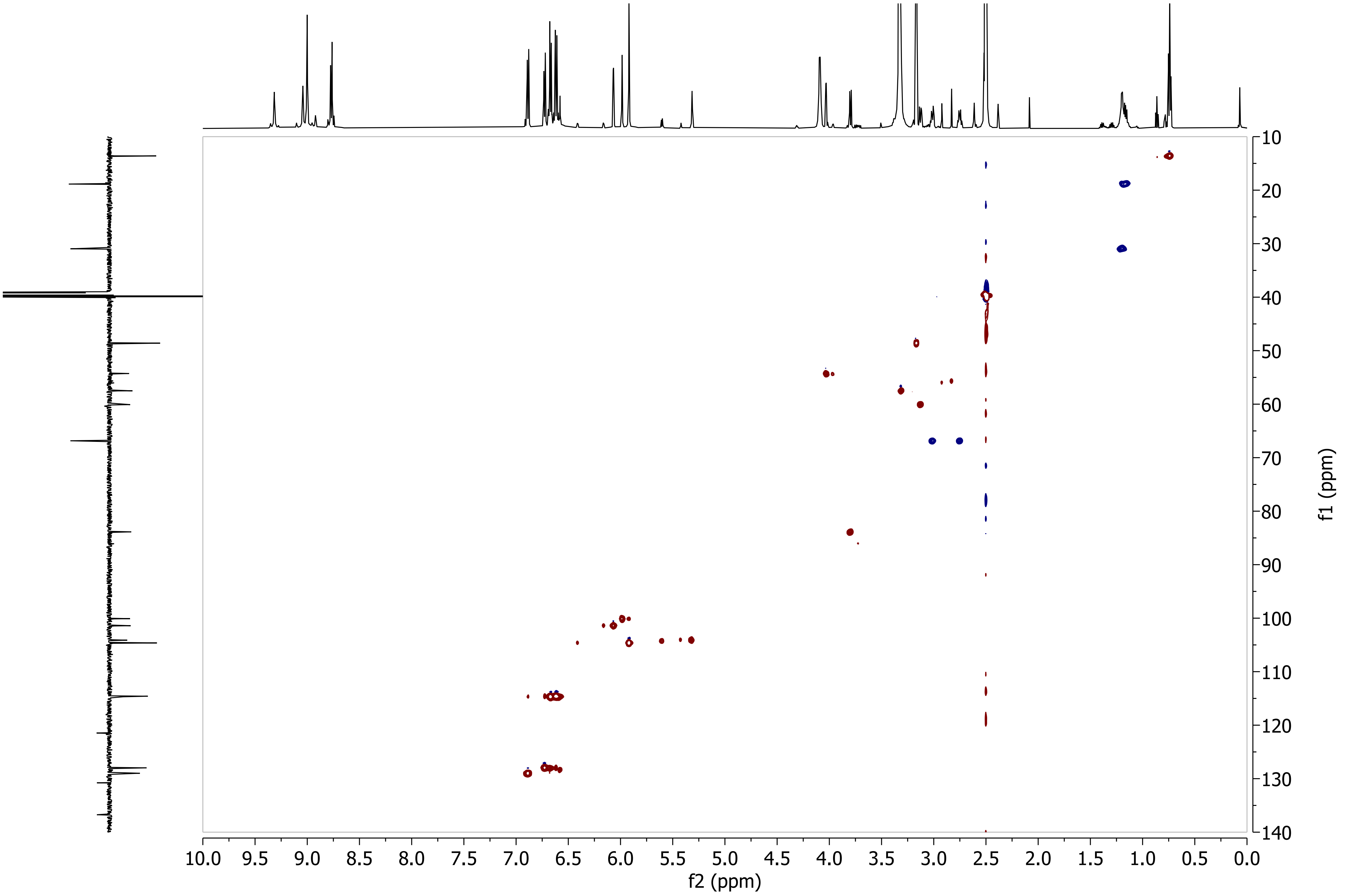

HMBC NMR spectrum of compound **25** in DMSO-d_6

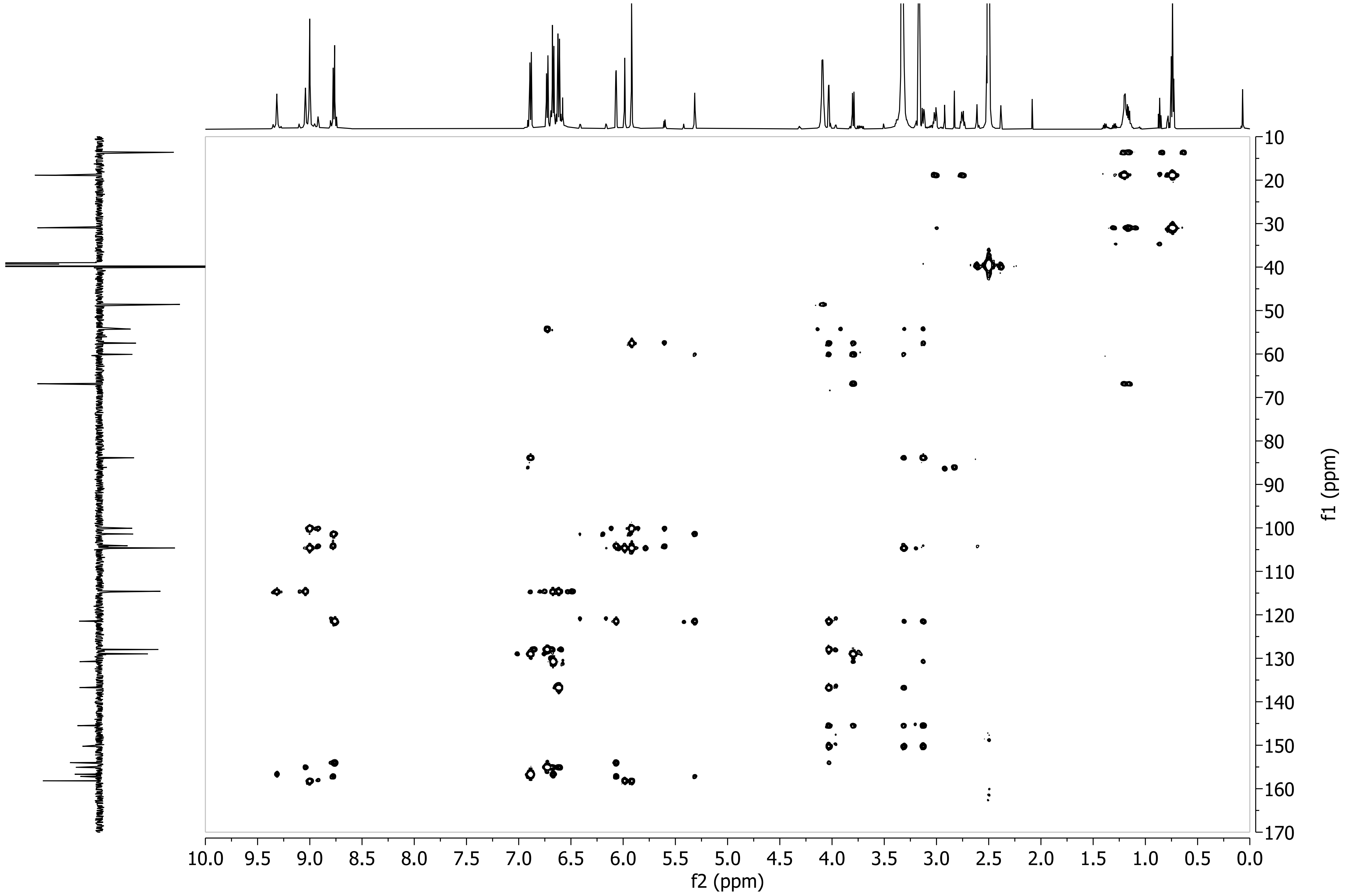

ROESY NMR spectrum of compound **25** in DMSO-d_6

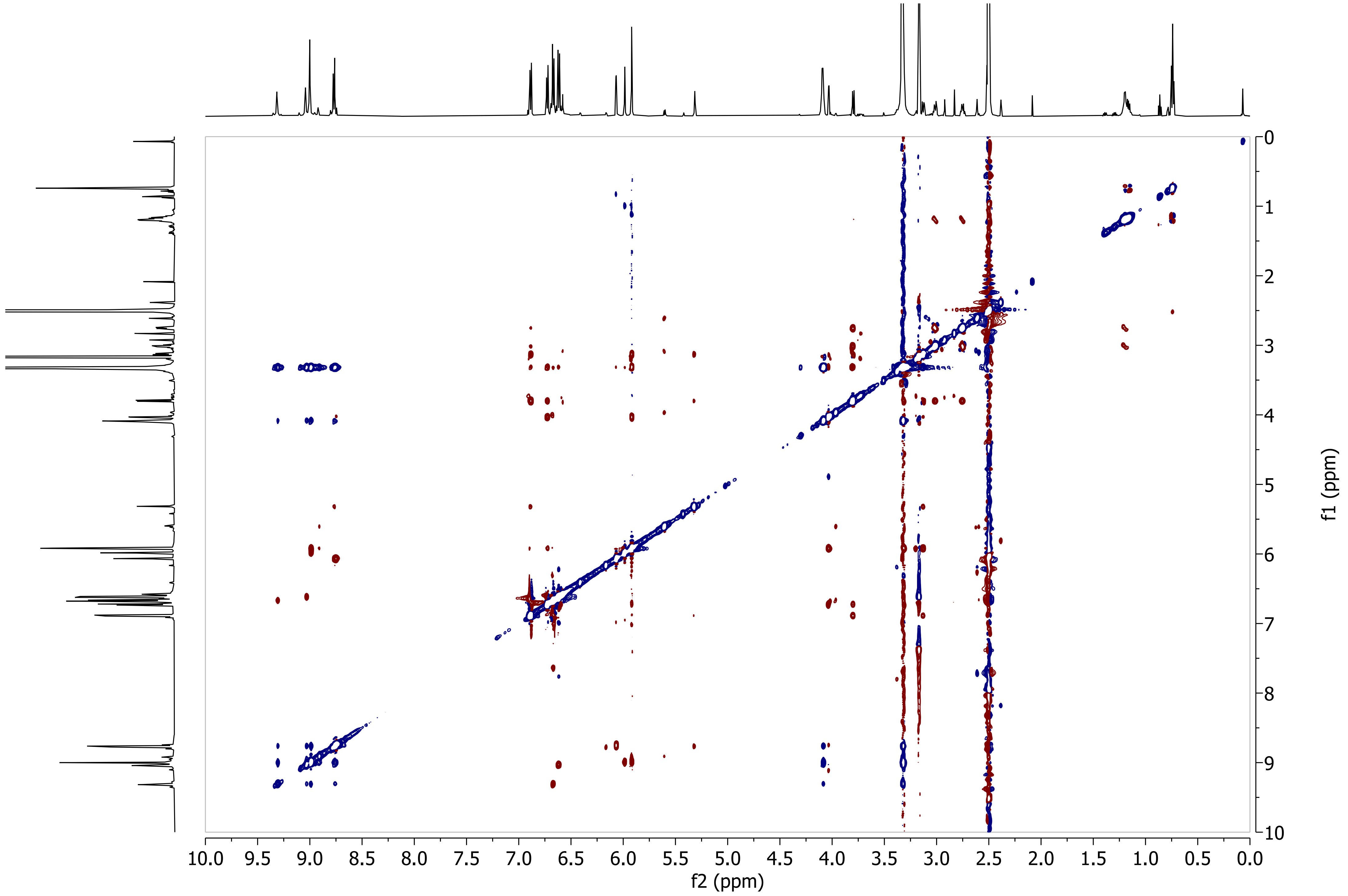

Supplement: Supplementary file 1 [file DataSheet2.pdf]
