# Supplementary material for: Chemoenzymatic Synthesis of Original Stilbene Dimers Possessing Wnt Inhibition Activity in Triple-Negative Breast Cancer Cells Using the Enzymatic Secretome of Botrytis cinerea Pers
Source: Front Chem. 2022 Apr 19;10:881298. doi: 10.3389/fchem.2022.881298 (PMC9062038; doi:10.3389/fchem.2022.881298)

^{1}H NMR spectrum of compound **48** in DMSO-d_6

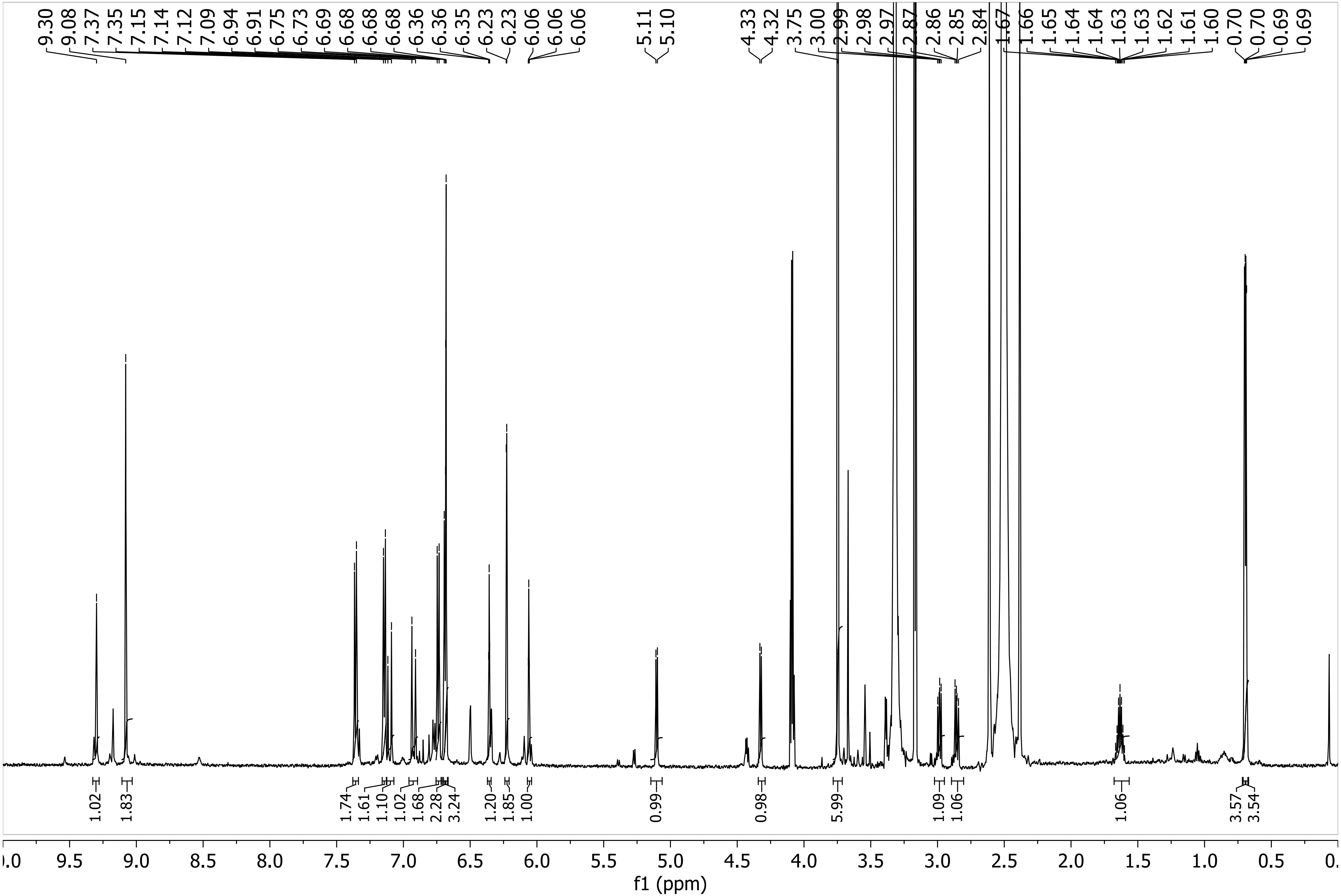

COSY NMR spectrum of compound **48** in DMSO-d_6

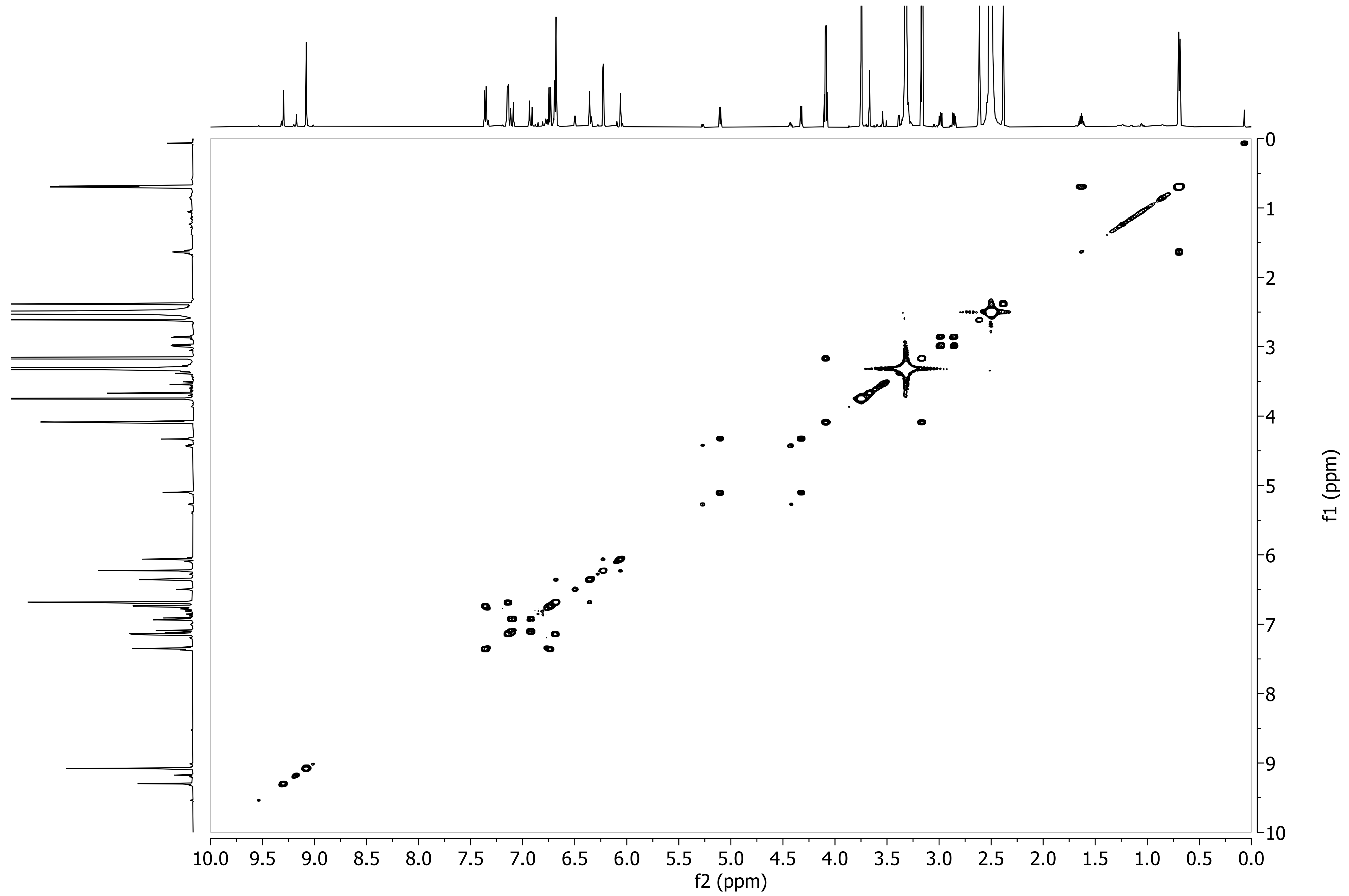

Edited-HSQC NMR spectrum of compound **48** in DMSO-d_6

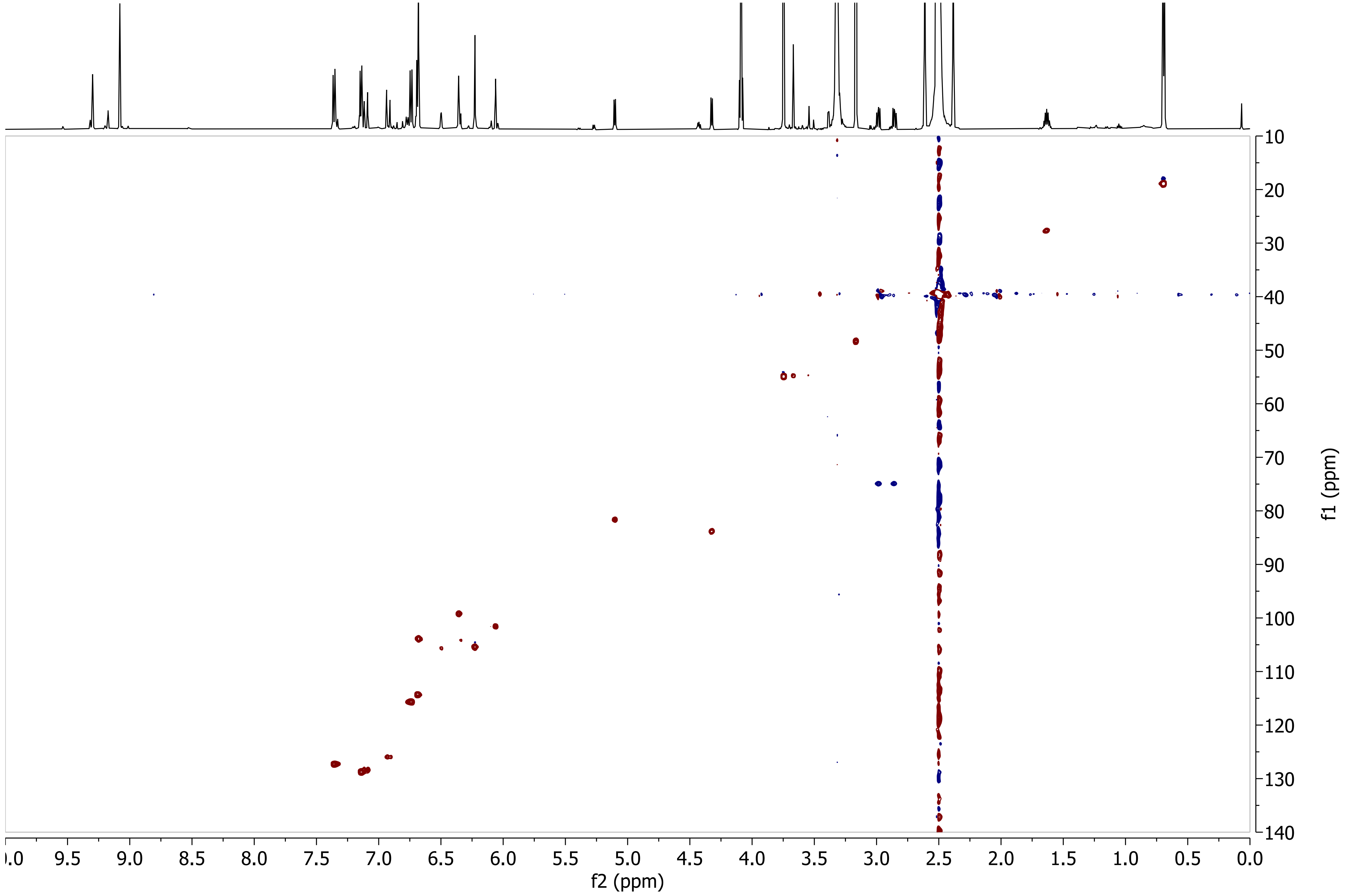

HMBC NMR spectrum of compound **48** in DMSO-d_6

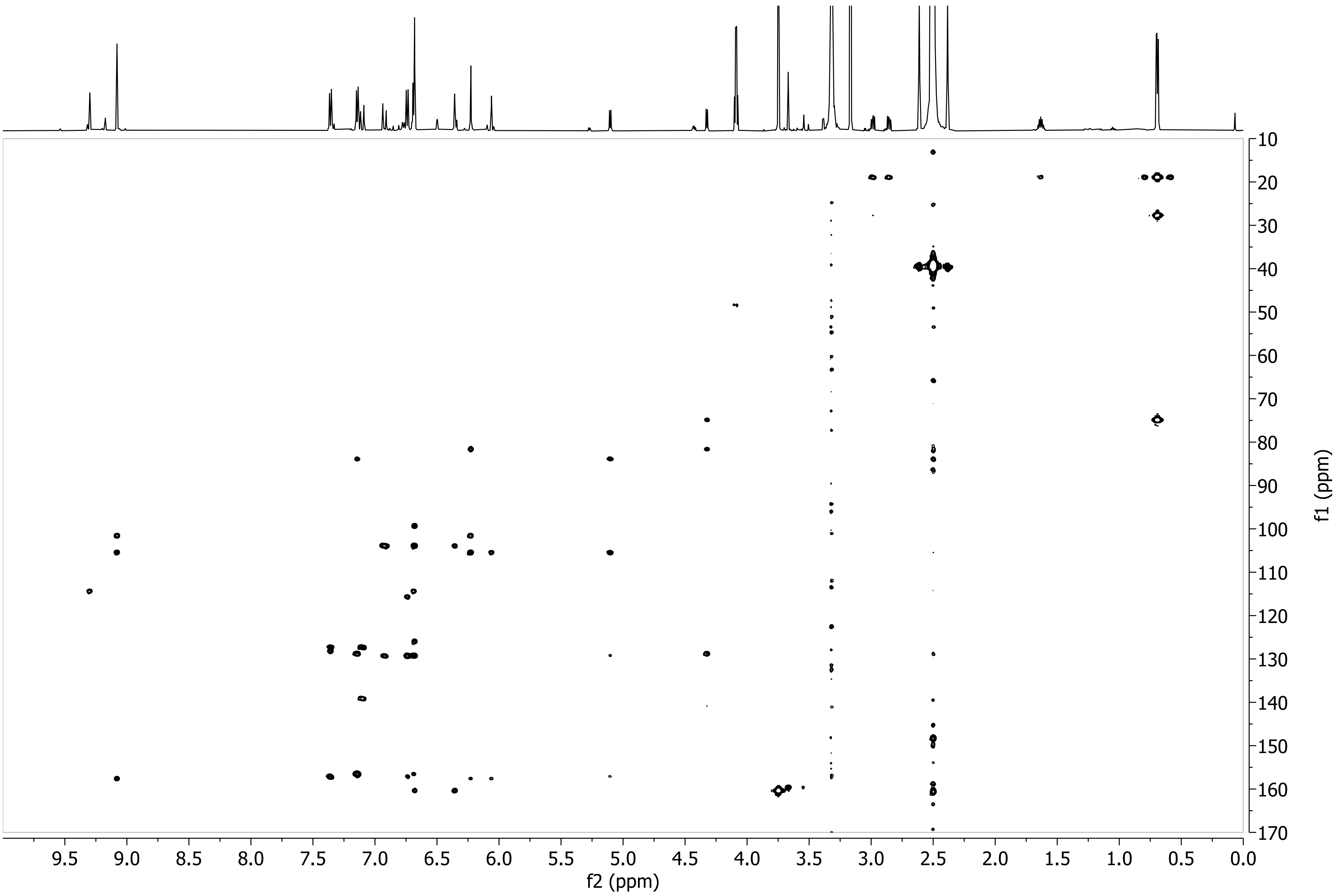

ROESY NMR spectrum of compound **48** in DMSO-d_6

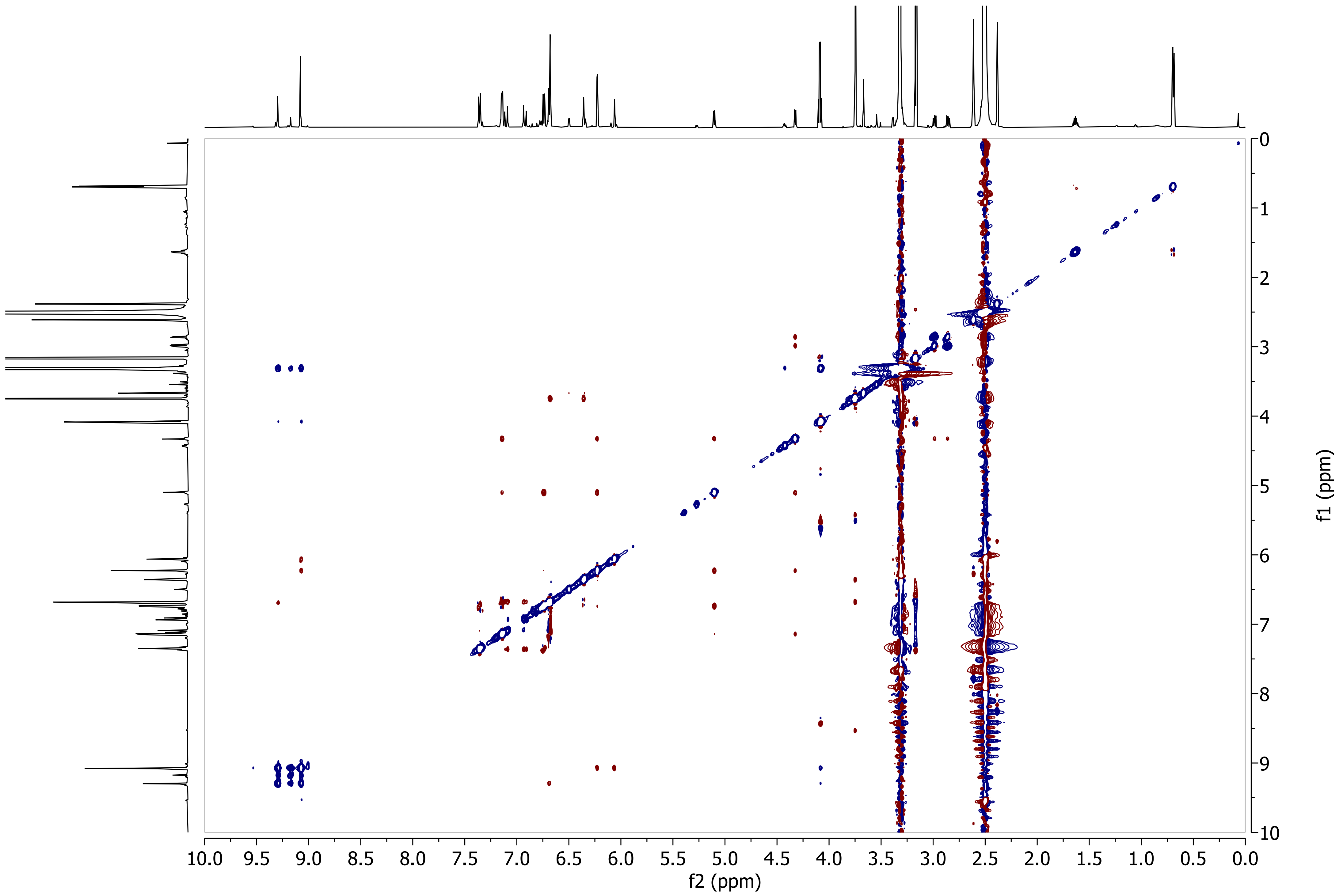

^{1}H NMR spectrum of compound **49** in DMSO-d_6

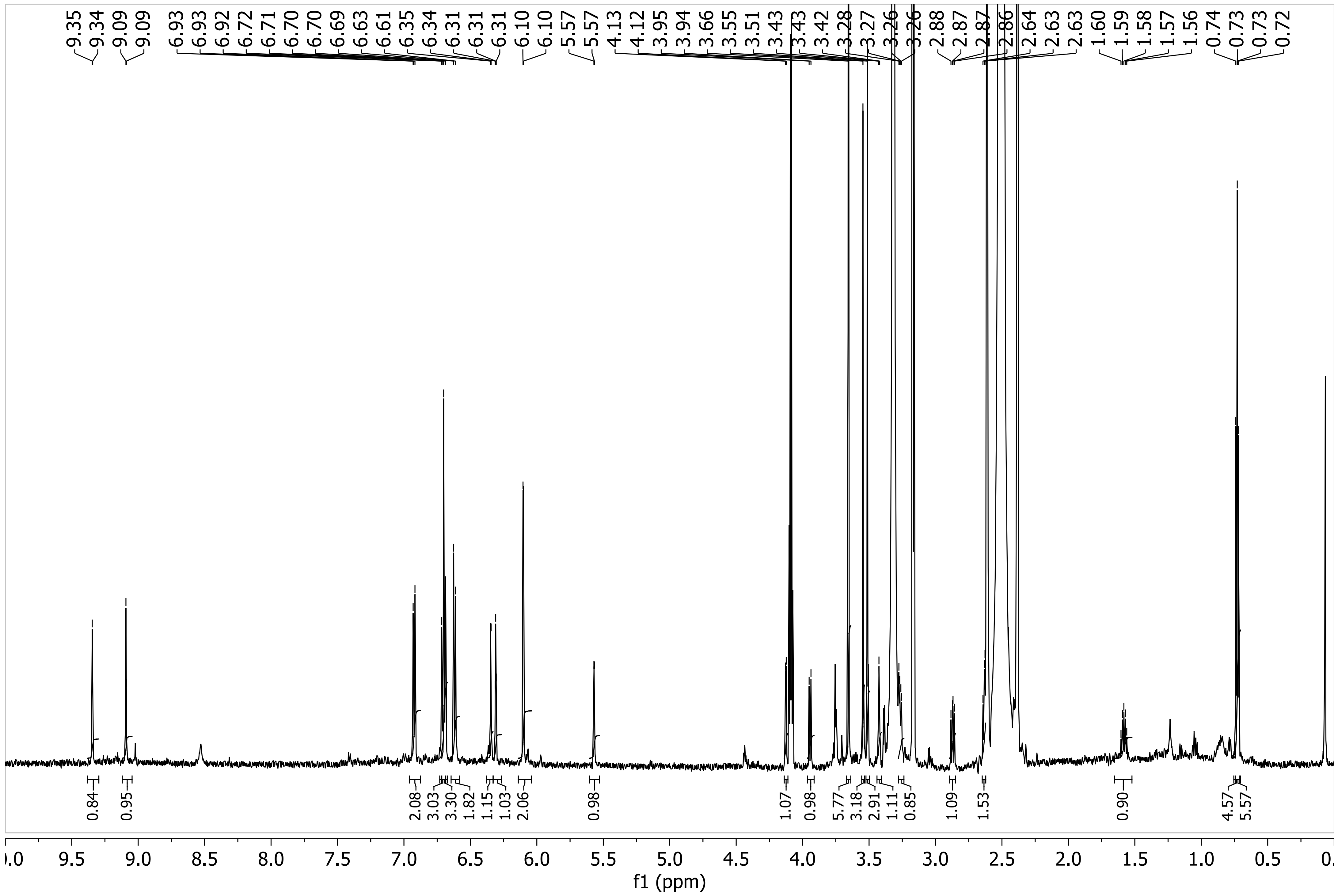

^1H NMR spectrum of compound **49** in DMSO-d_6

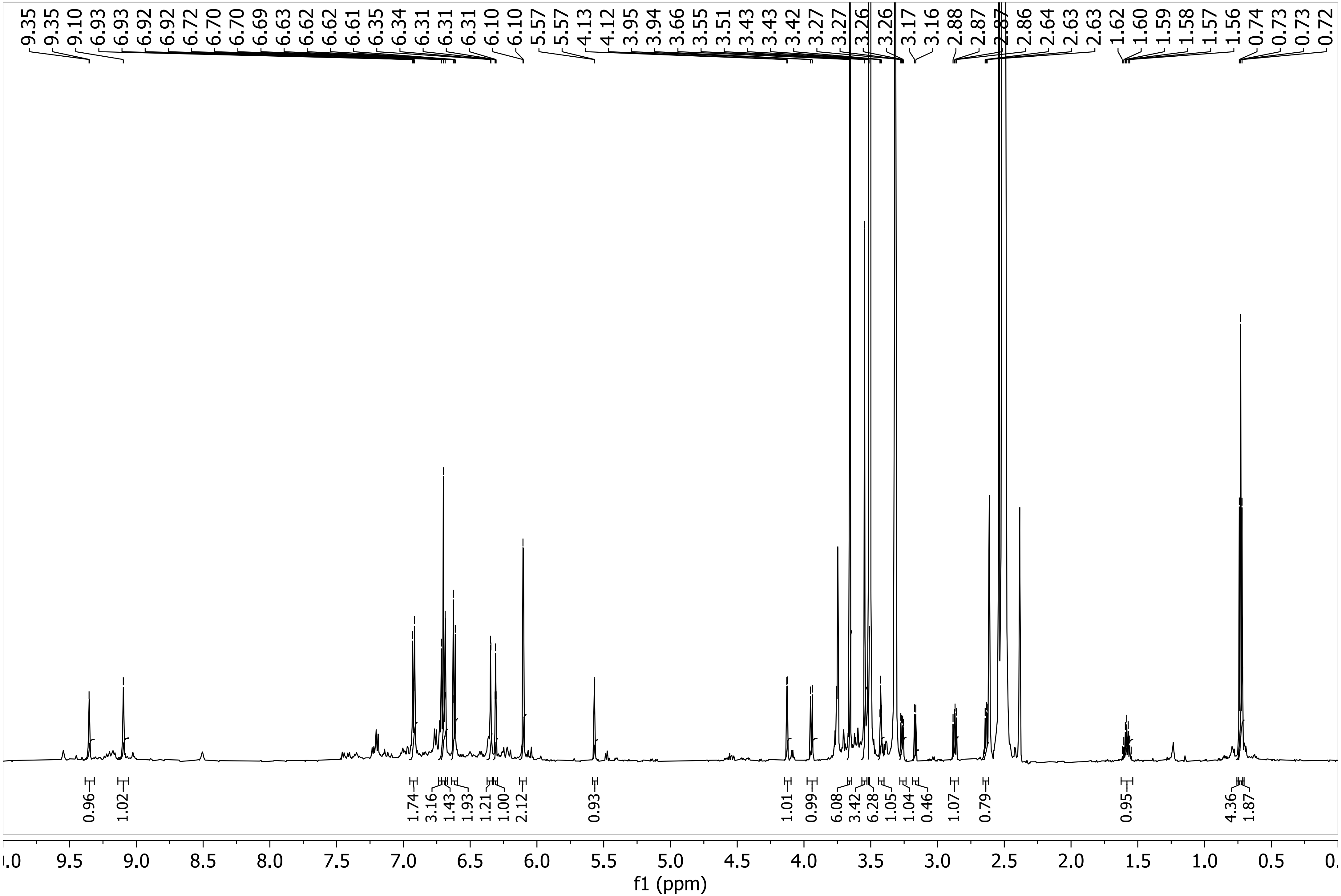

COSY NMR spectrum of compound **49** in DMSO-d_6

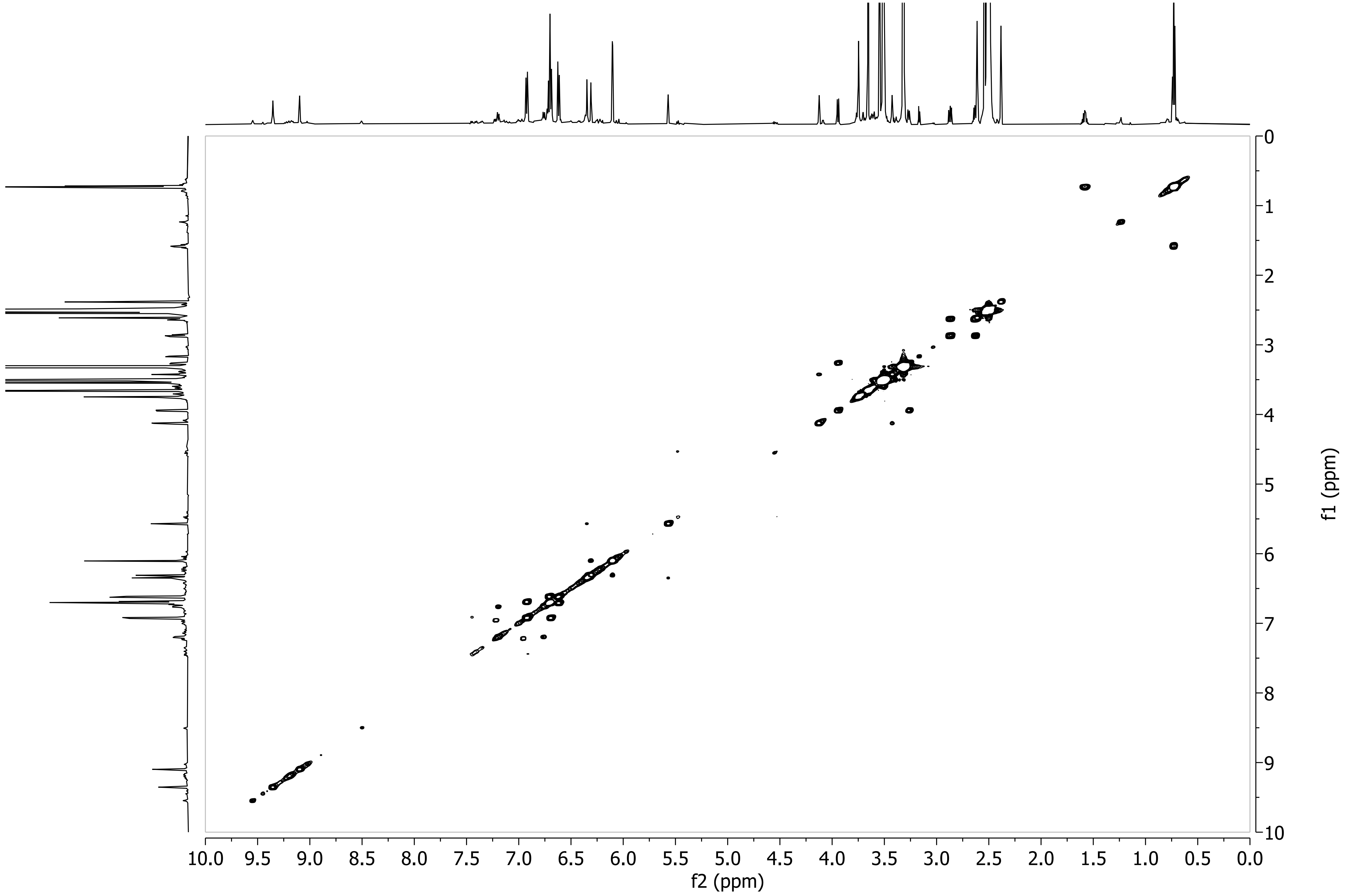

^{13}C-DEPTQ NMR spectrum of compound **49** in DMSO-d_6

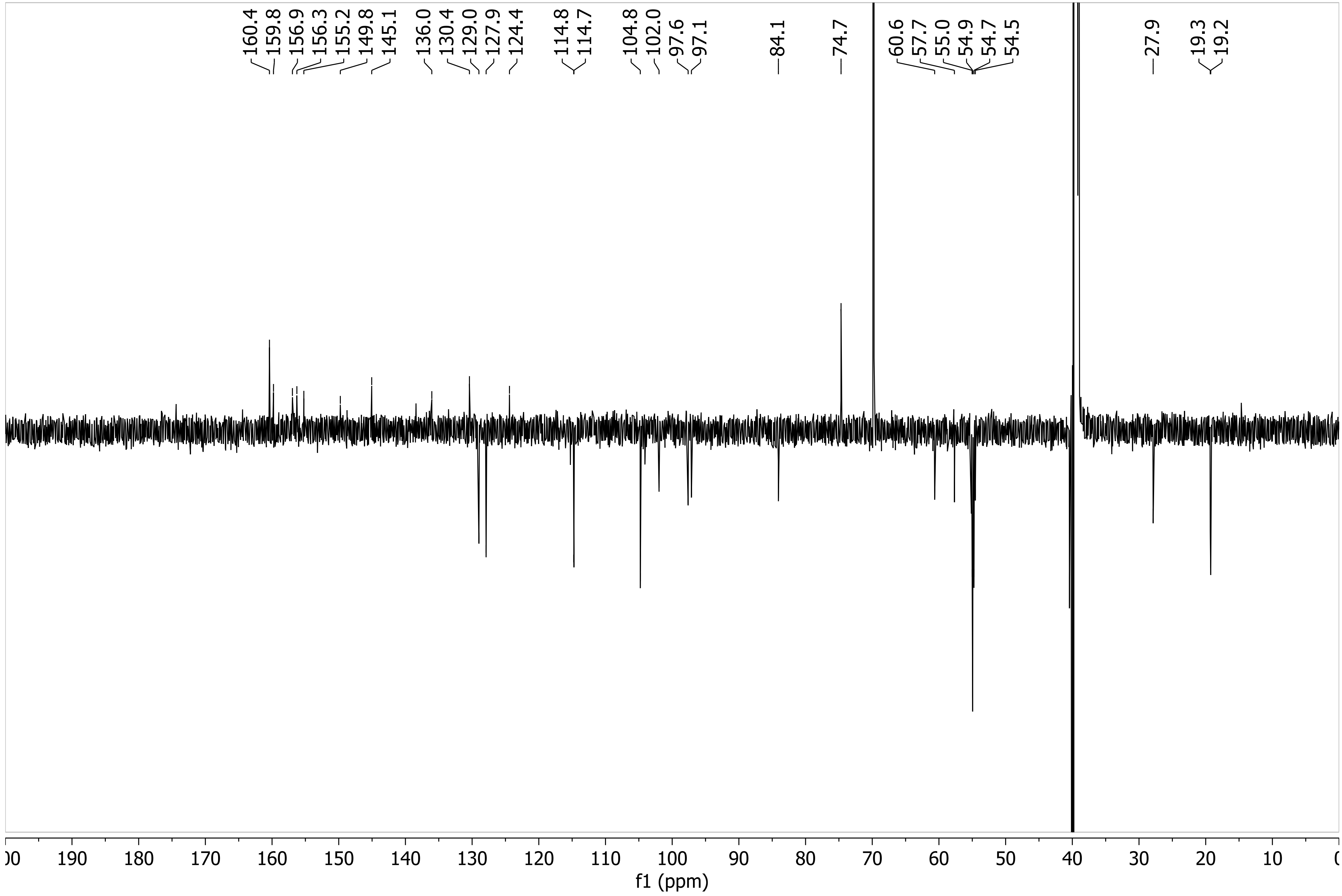

Edited-HSQC NMR spectrum of compound **49** in DMSO-d_6

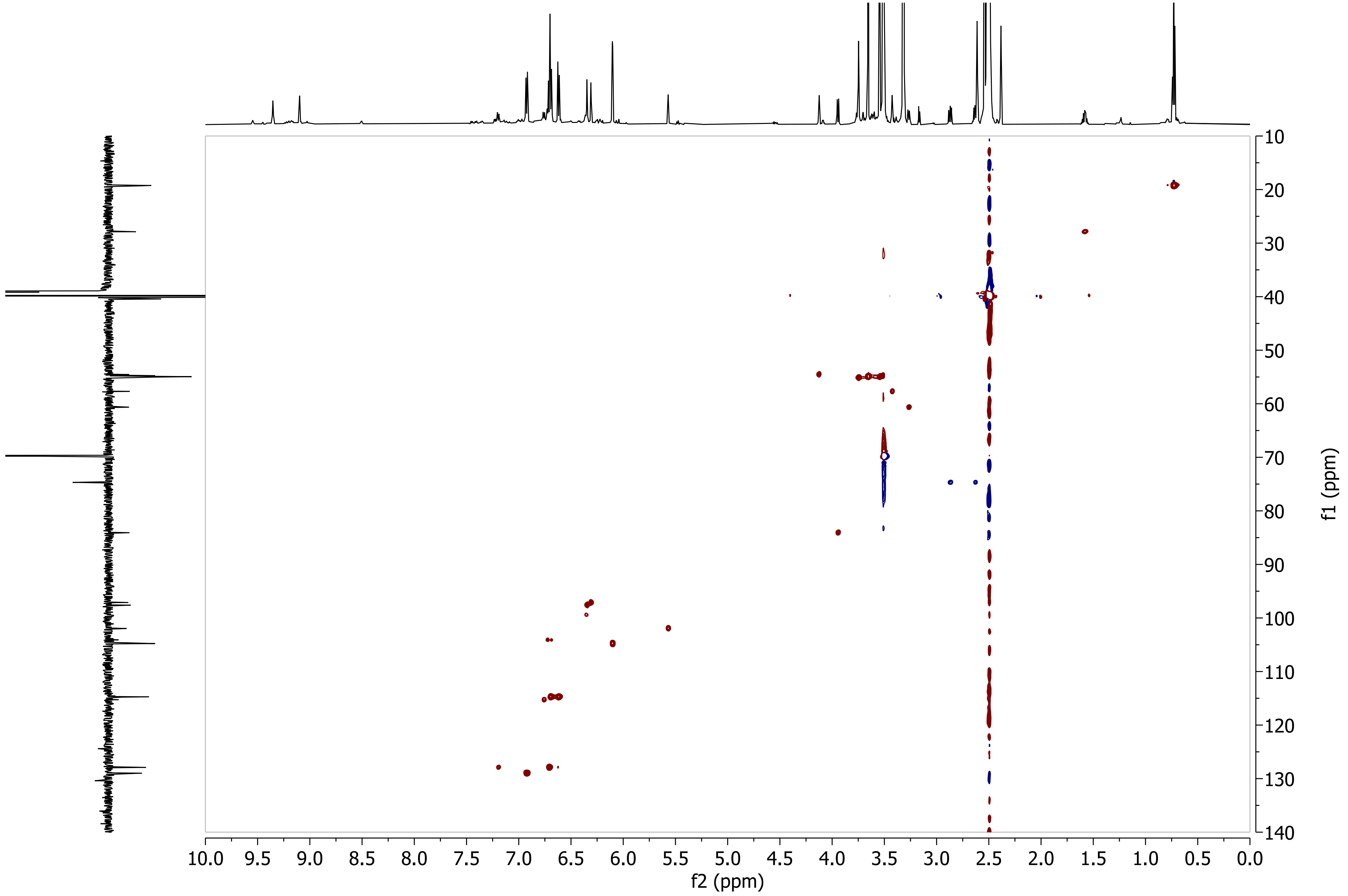

HMBC NMR spectrum of compound **49** in DMSO-d_6

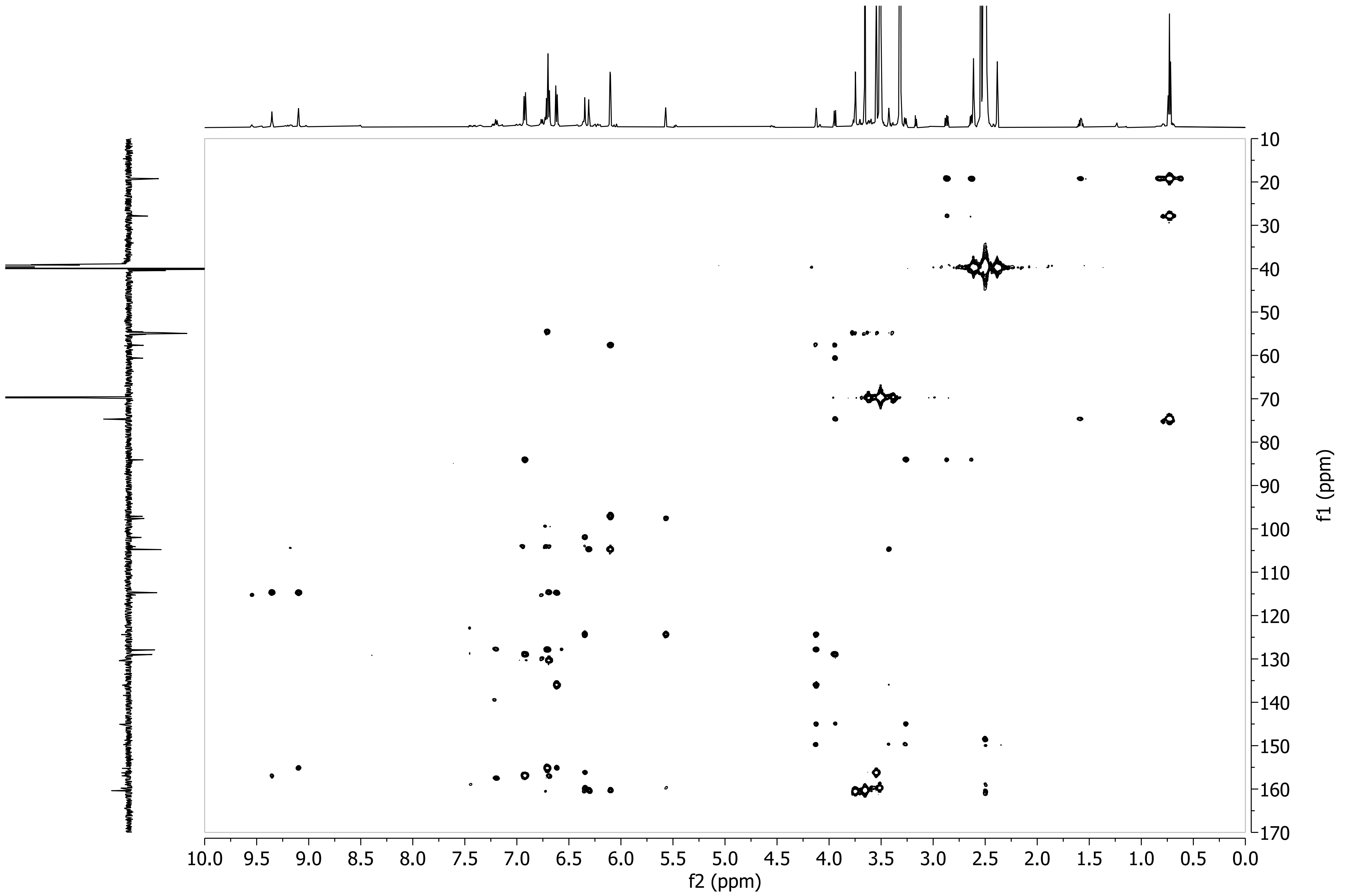

ROESY NMR spectrum of compound **49** in DMSO-d_6

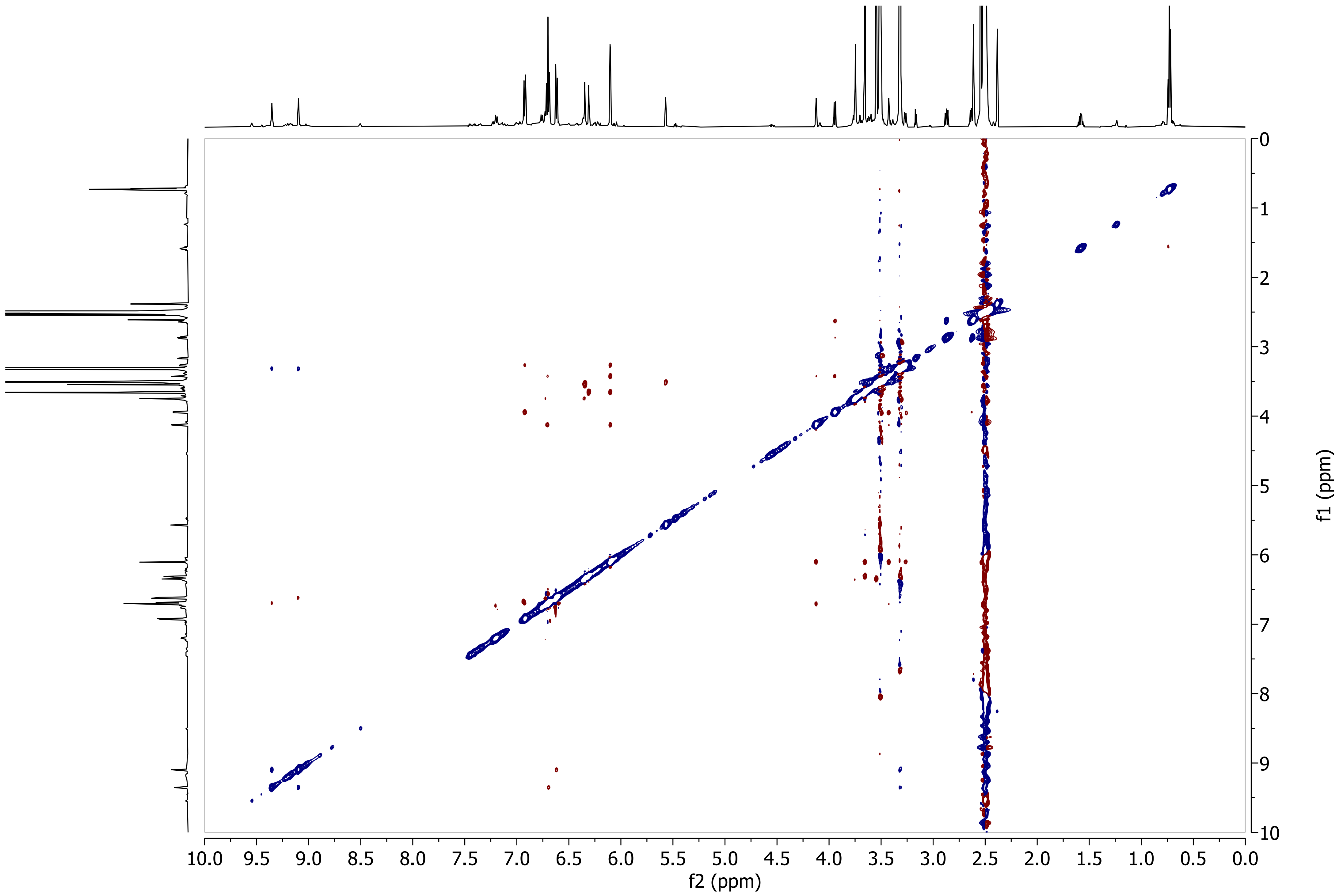

^1H NMR spectrum of compound **50** in DMSO-d_6

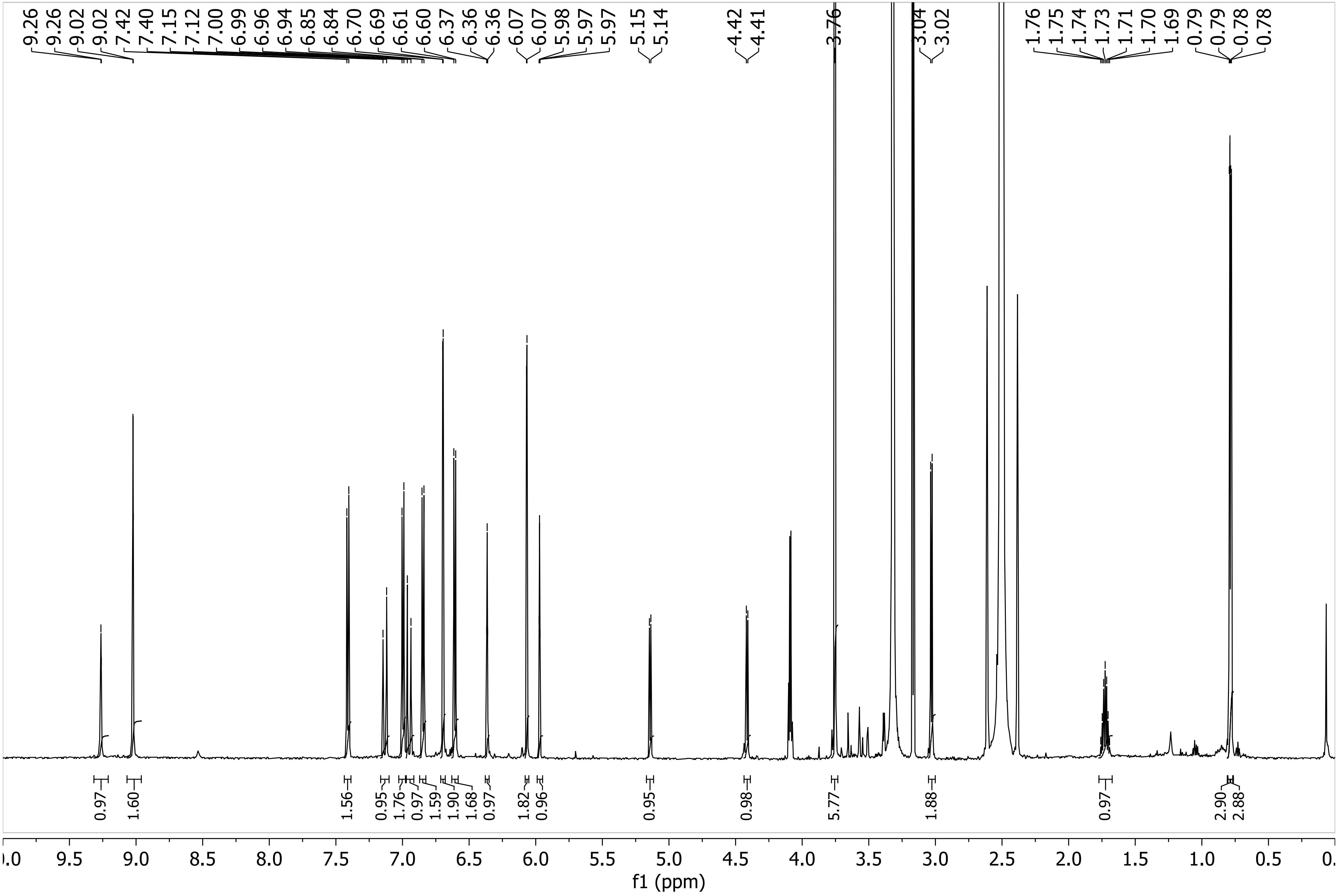

^{1}H NMR spectrum of compound **50** in DMSO-d_6

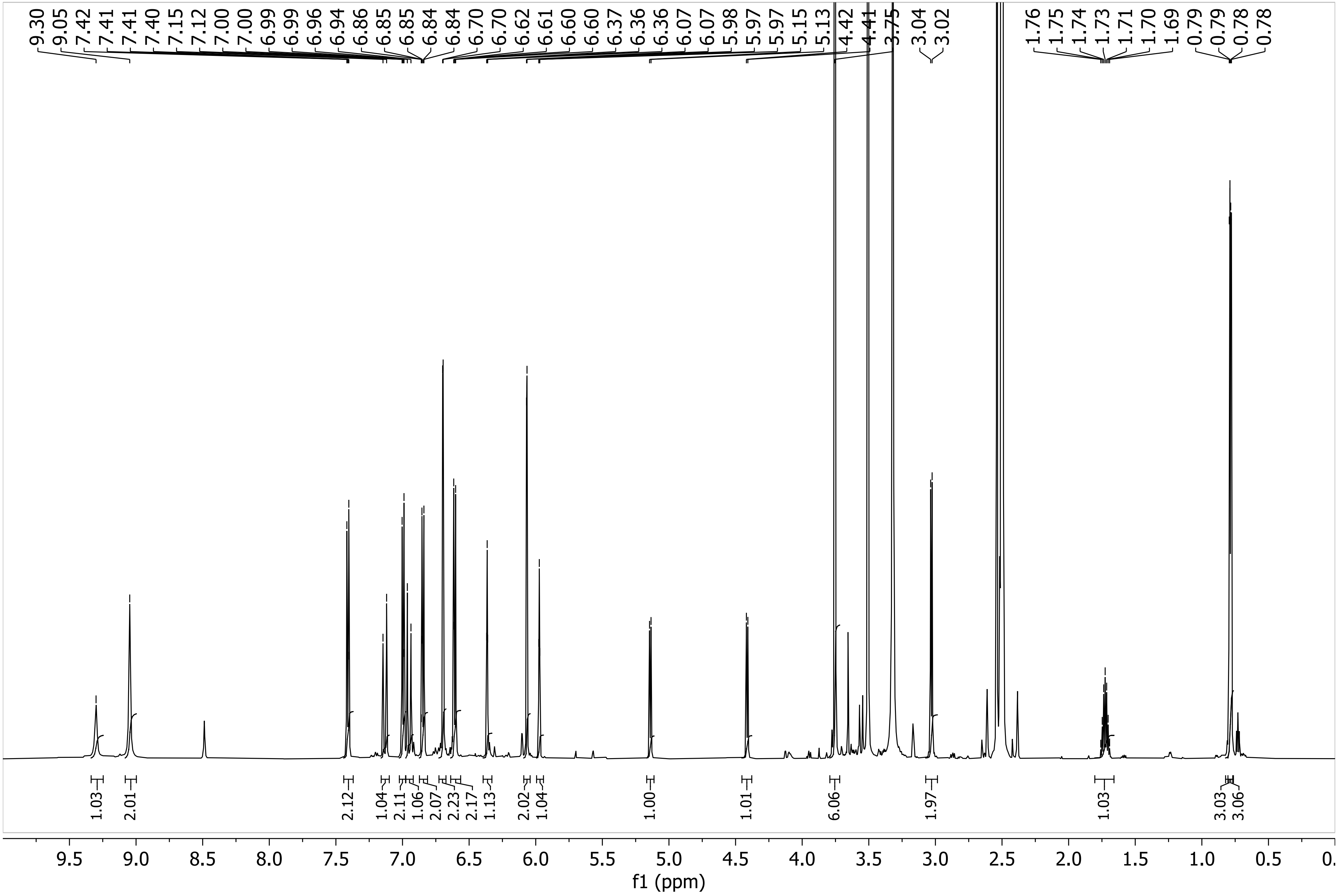

COSY NMR spectrum of compound **50** in DMSO-d_6

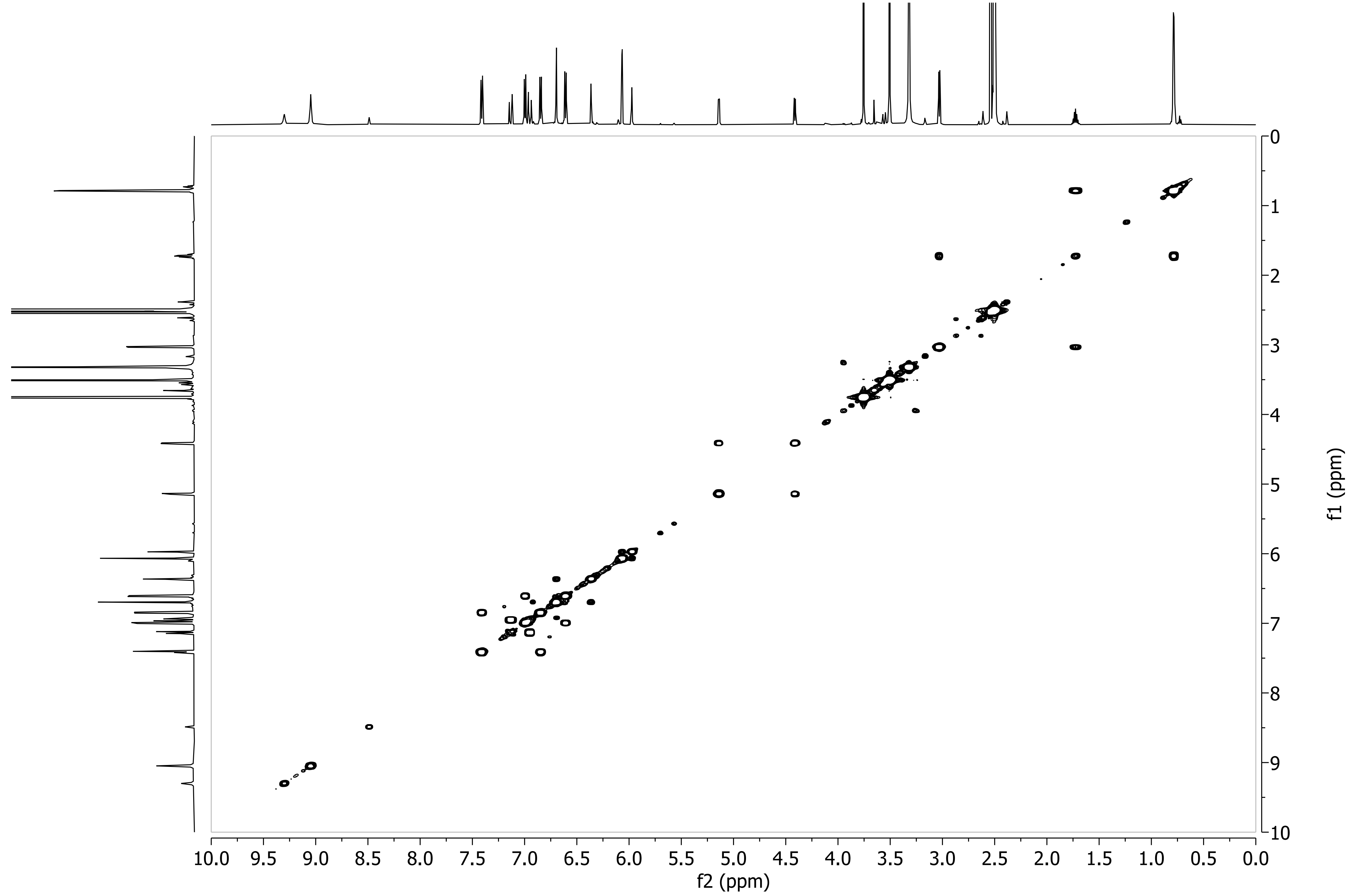

^{13}C-DEPTQ NMR spectrum of compound **50** in DMSO-d_6

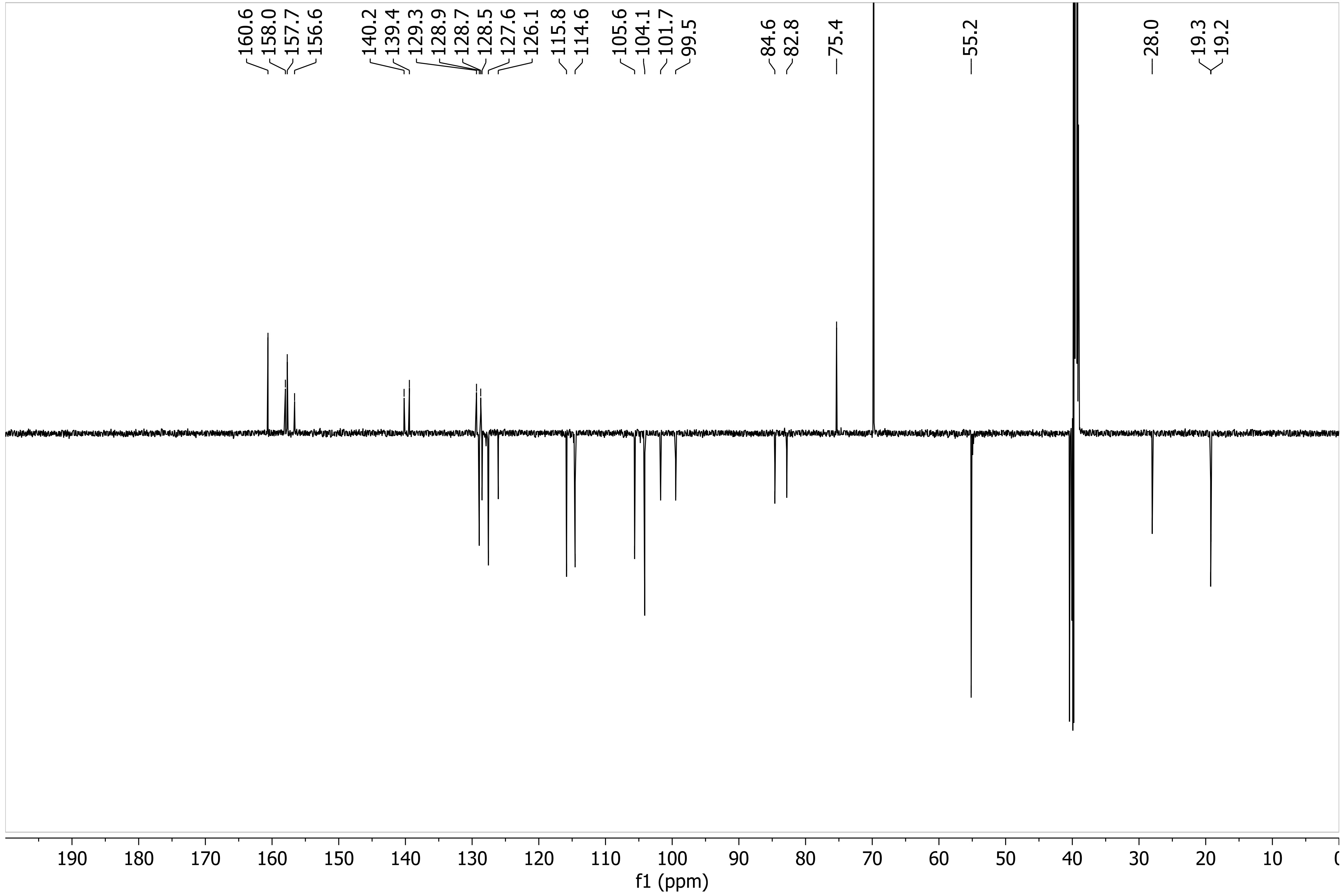

Edited-HSQC NMR spectrum of compound **50** in DMSO-d_6

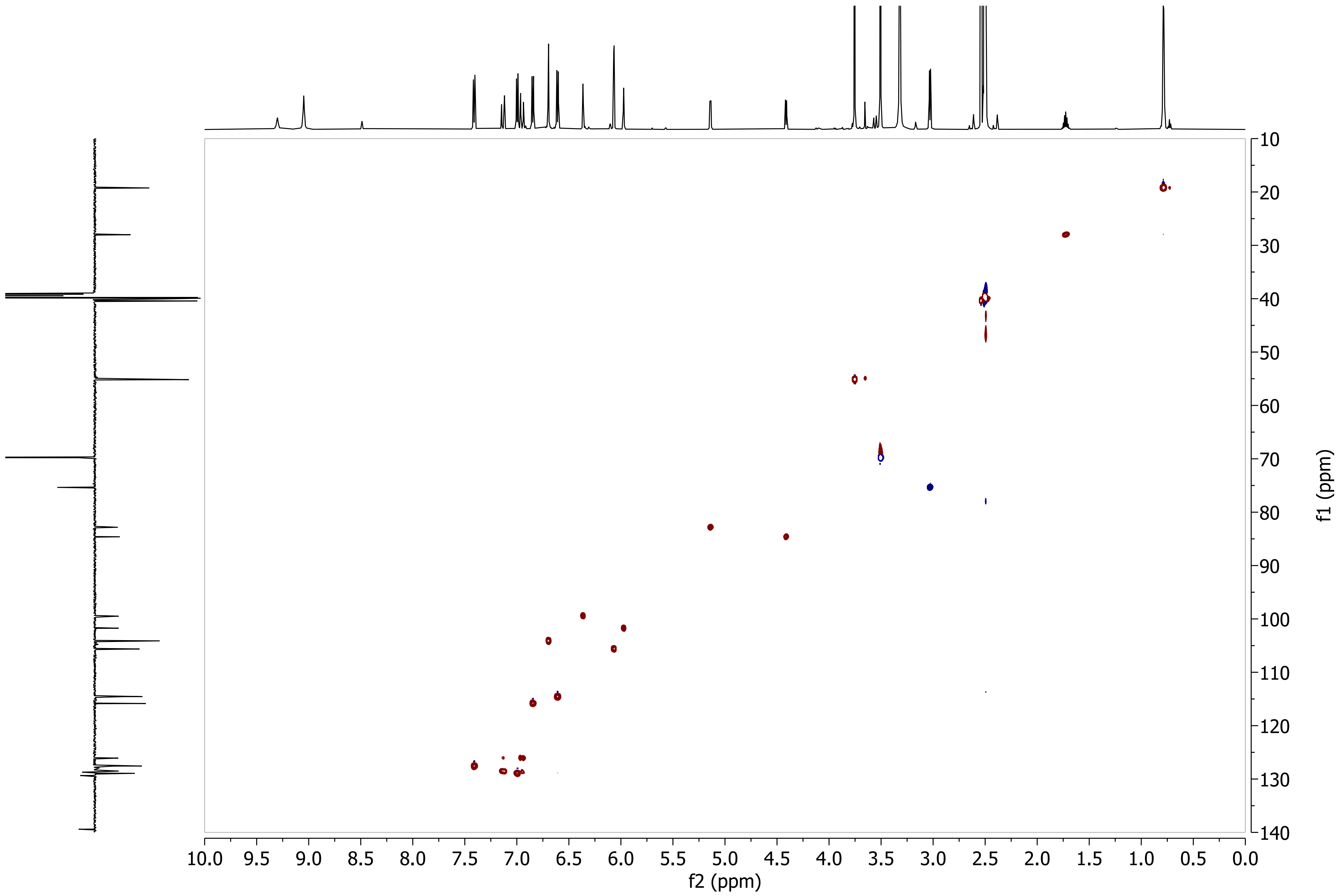

HMBC NMR spectrum of compound **50** in DMSO-d_6

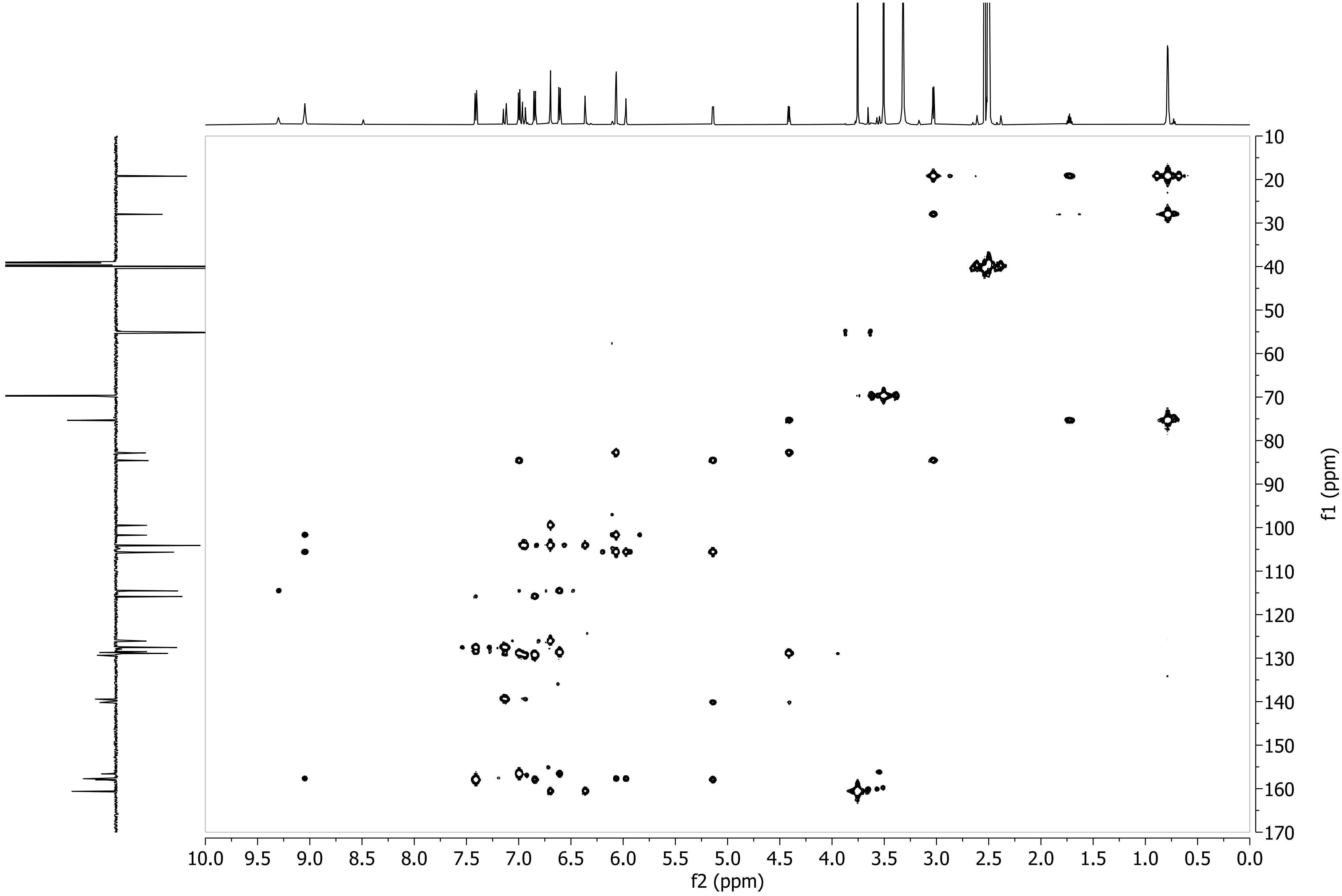

ROESY NMR spectrum of compound **50** in DMSO-d_6

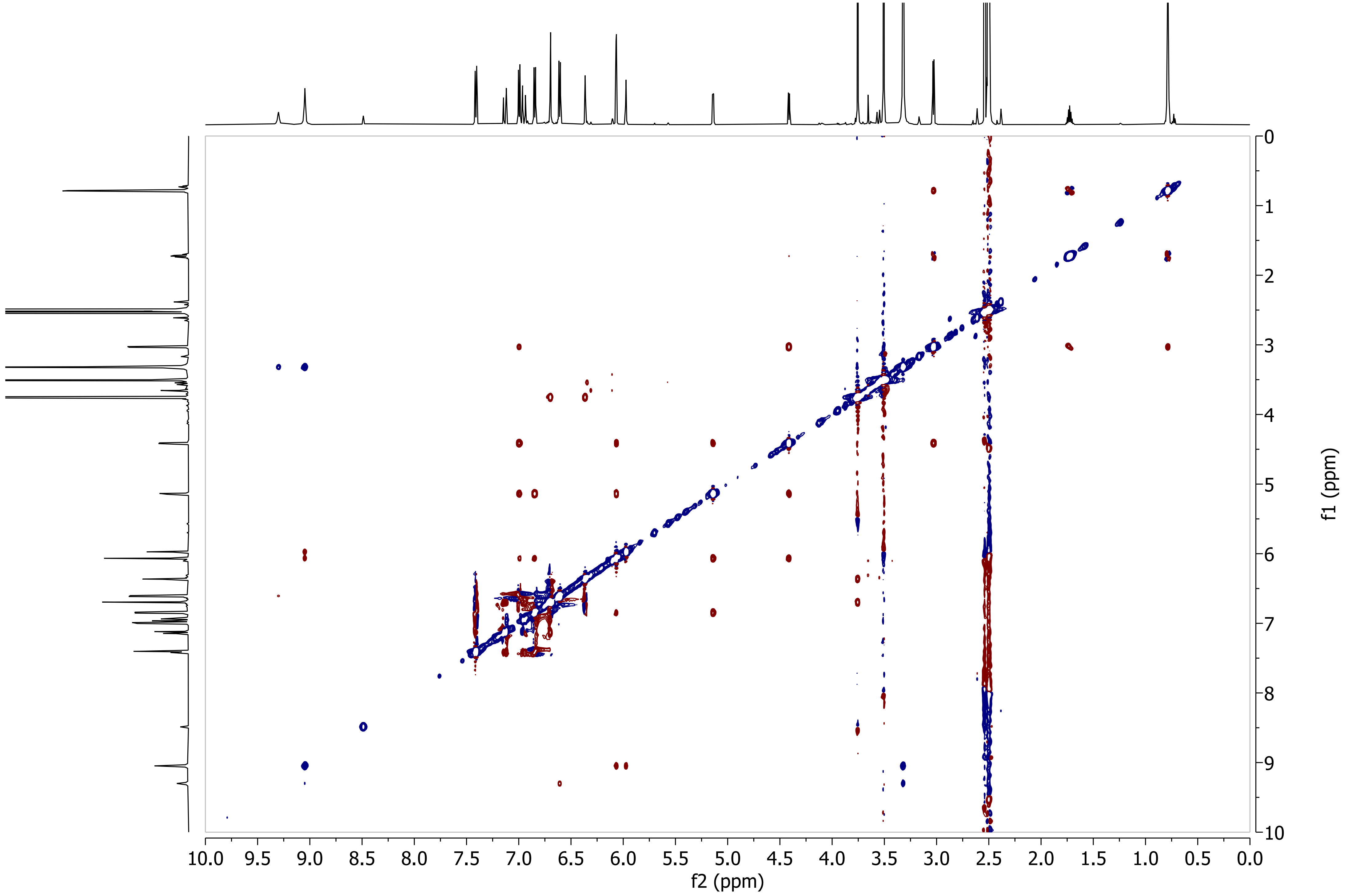

^{1}H NMR spectrum of compound **51** in DMSO-d_6

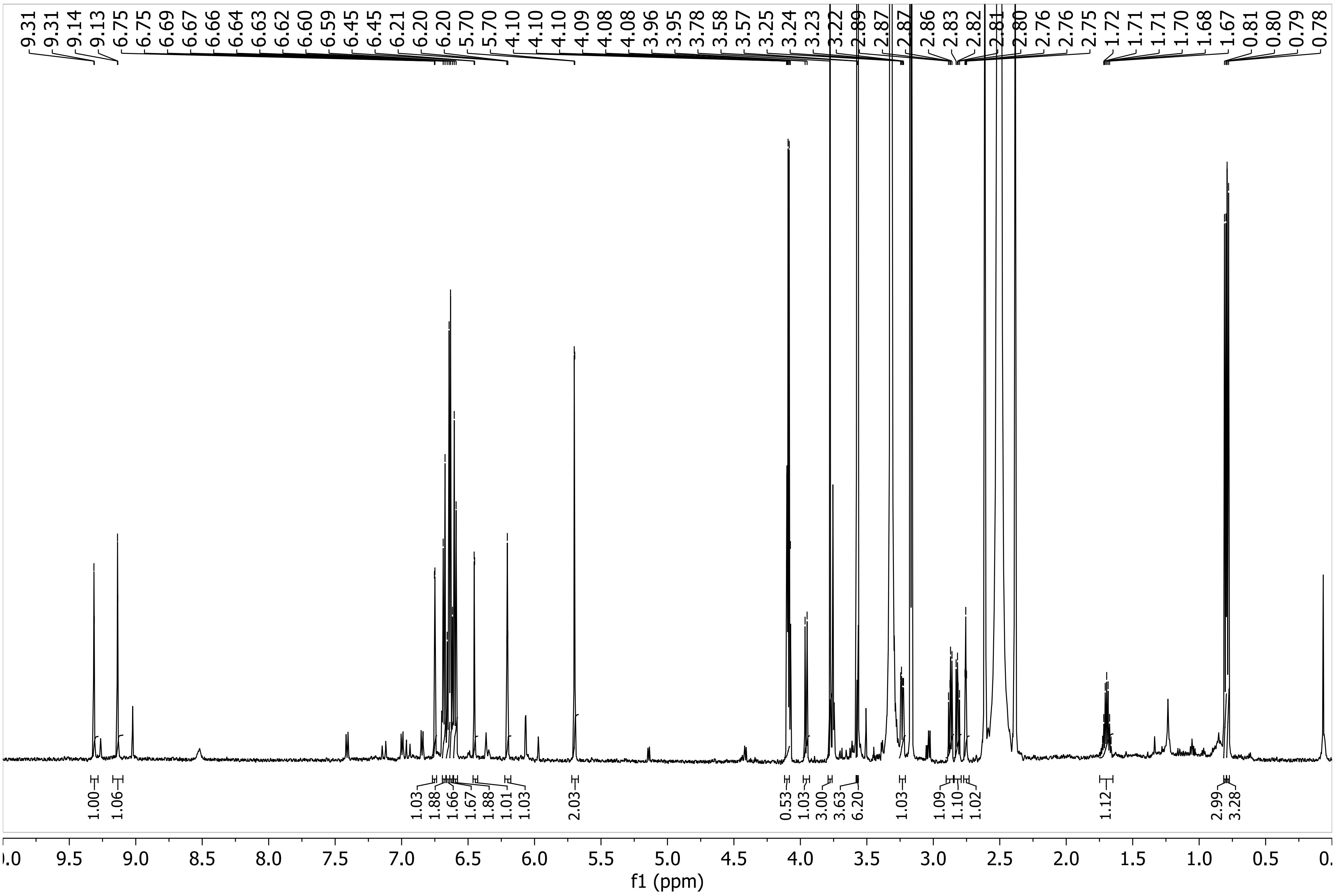

COSY NMR spectrum of compound **51** in DMSO-d_6

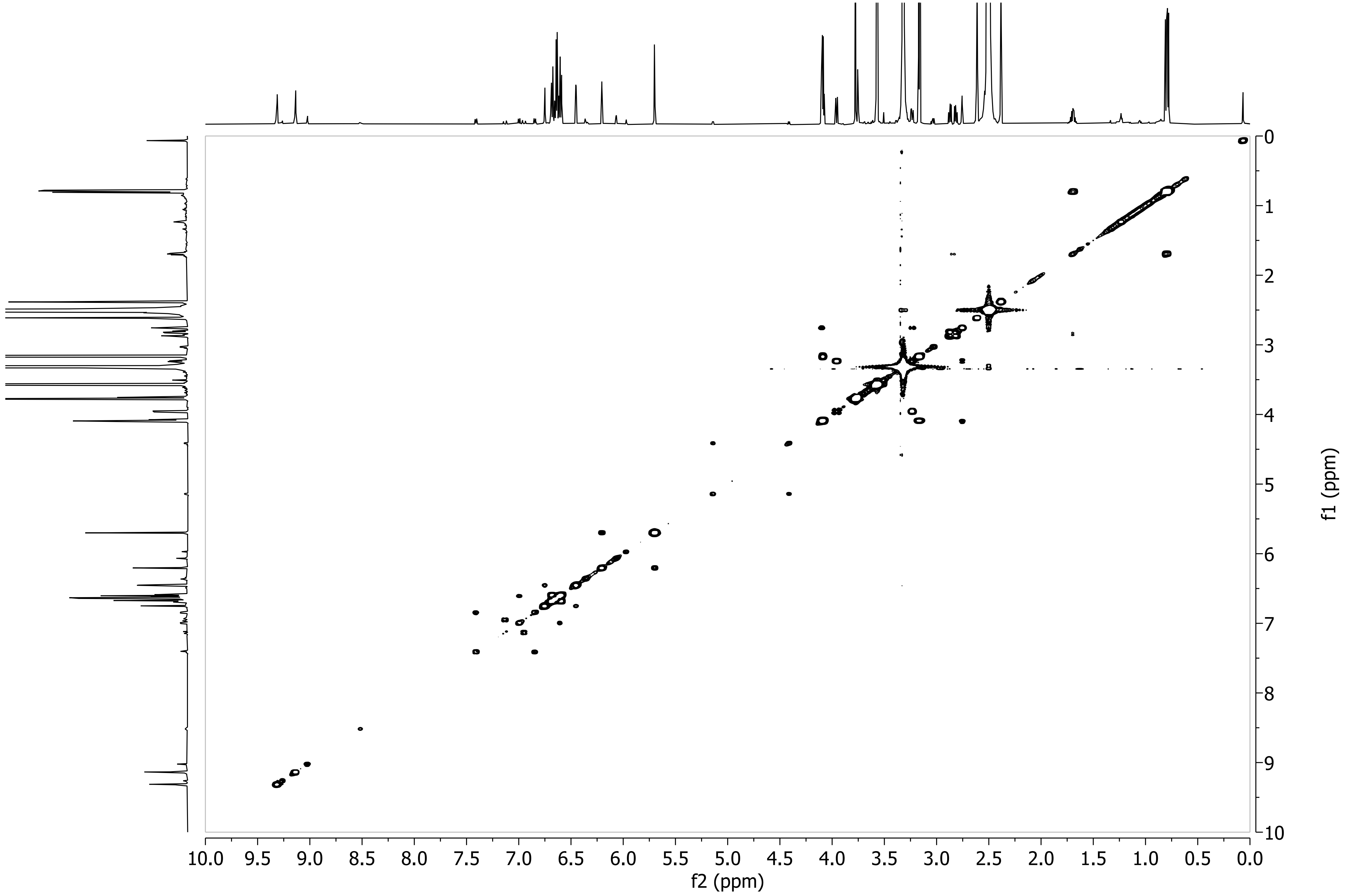

Edited-HSQC NMR spectrum of compound **51** in DMSO-d_6

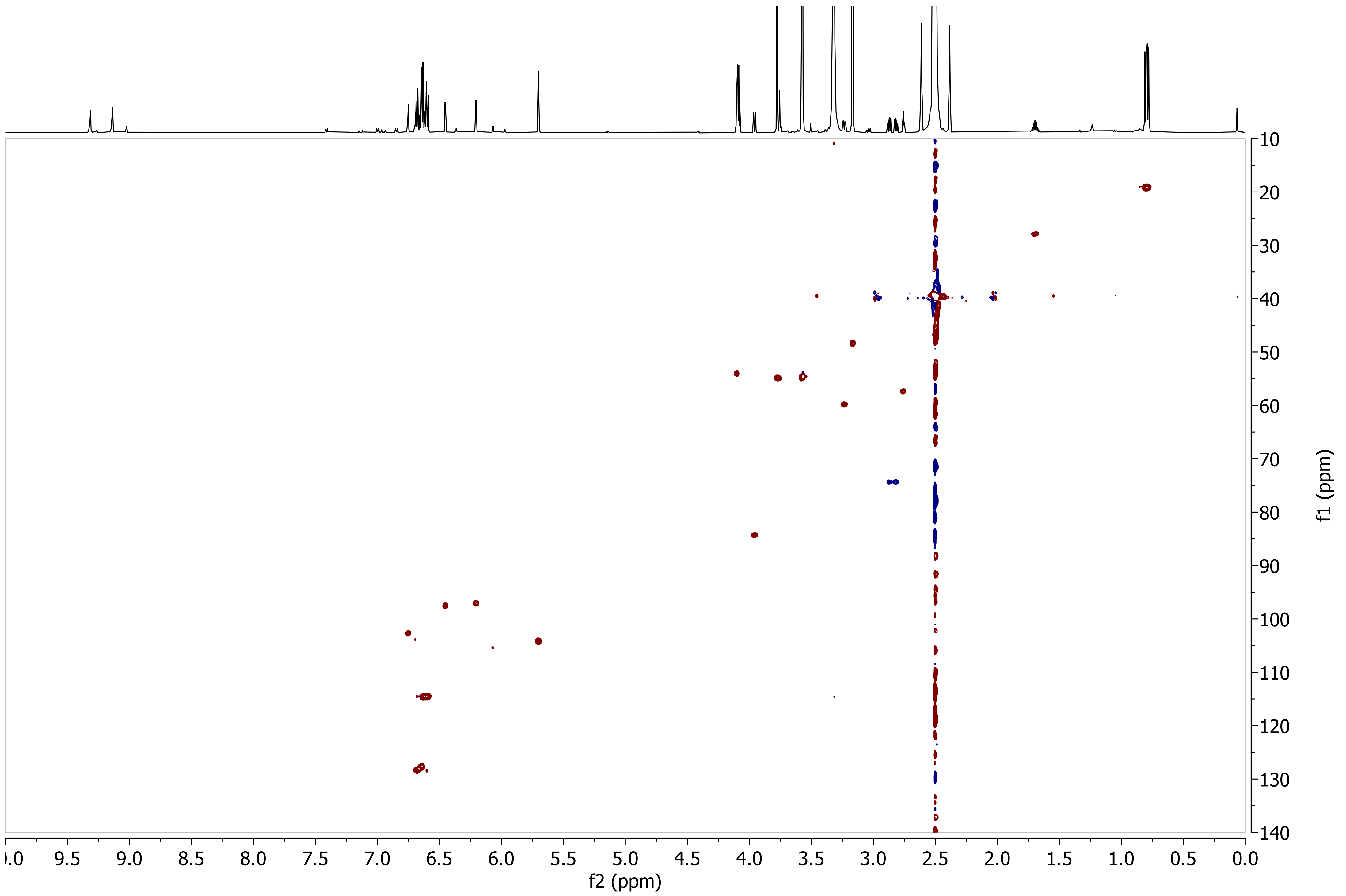

HMBC NMR spectrum of compound **51** in DMSO-d_6

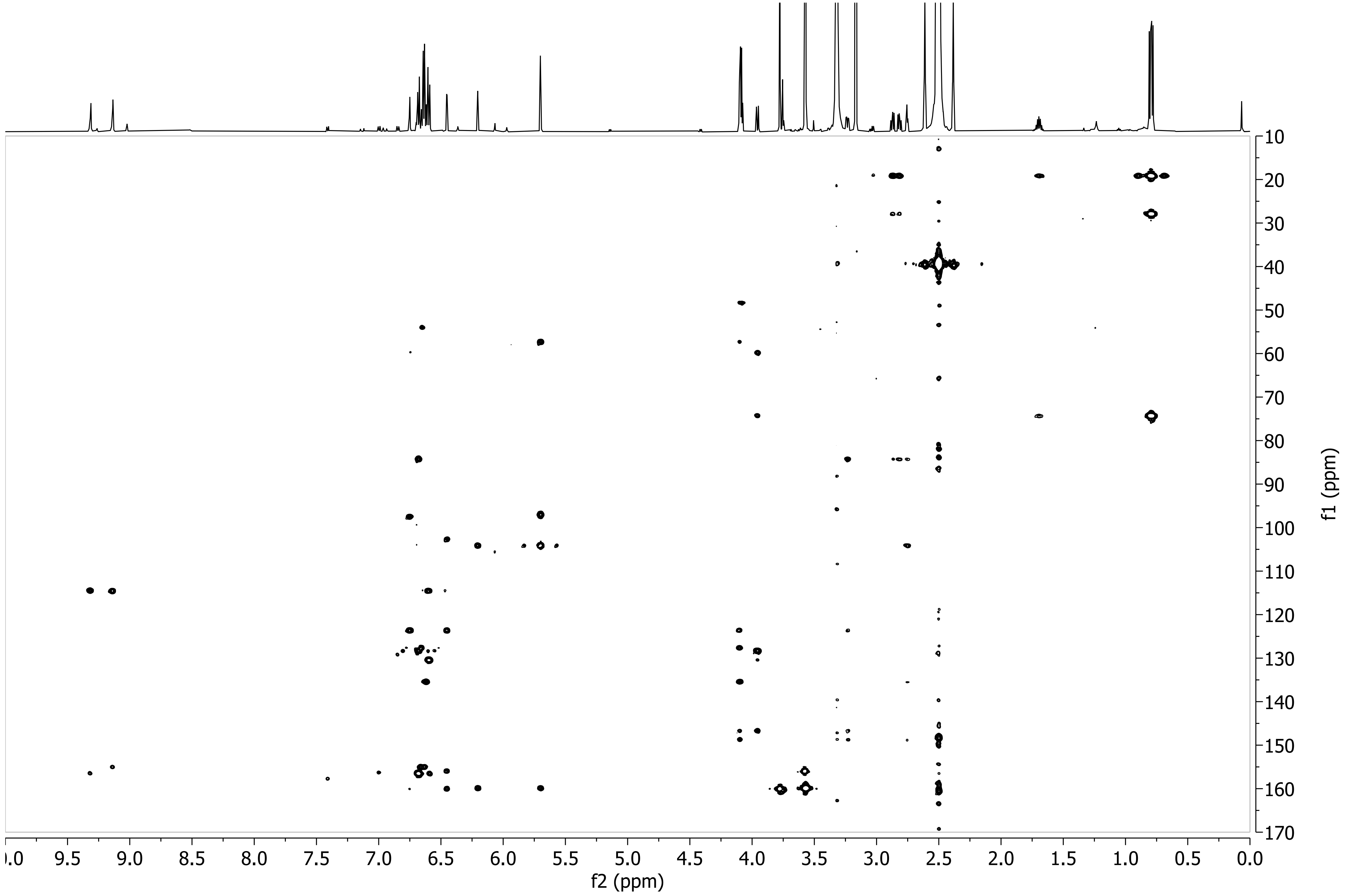

ROESY NMR spectrum of compound **51** in DMSO-d_6

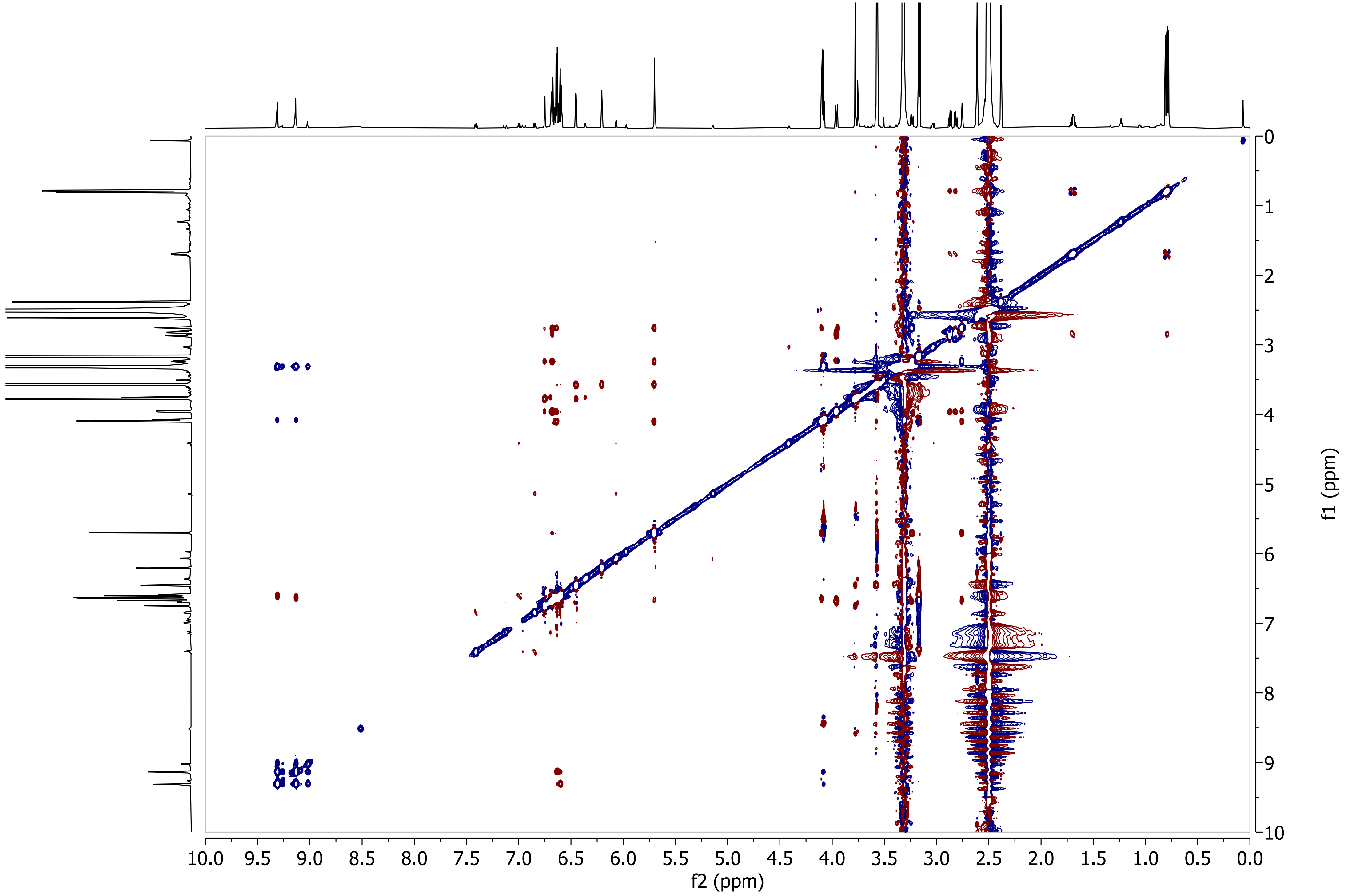

^{1}H NMR spectrum of compound **52** in DMSO-d_6

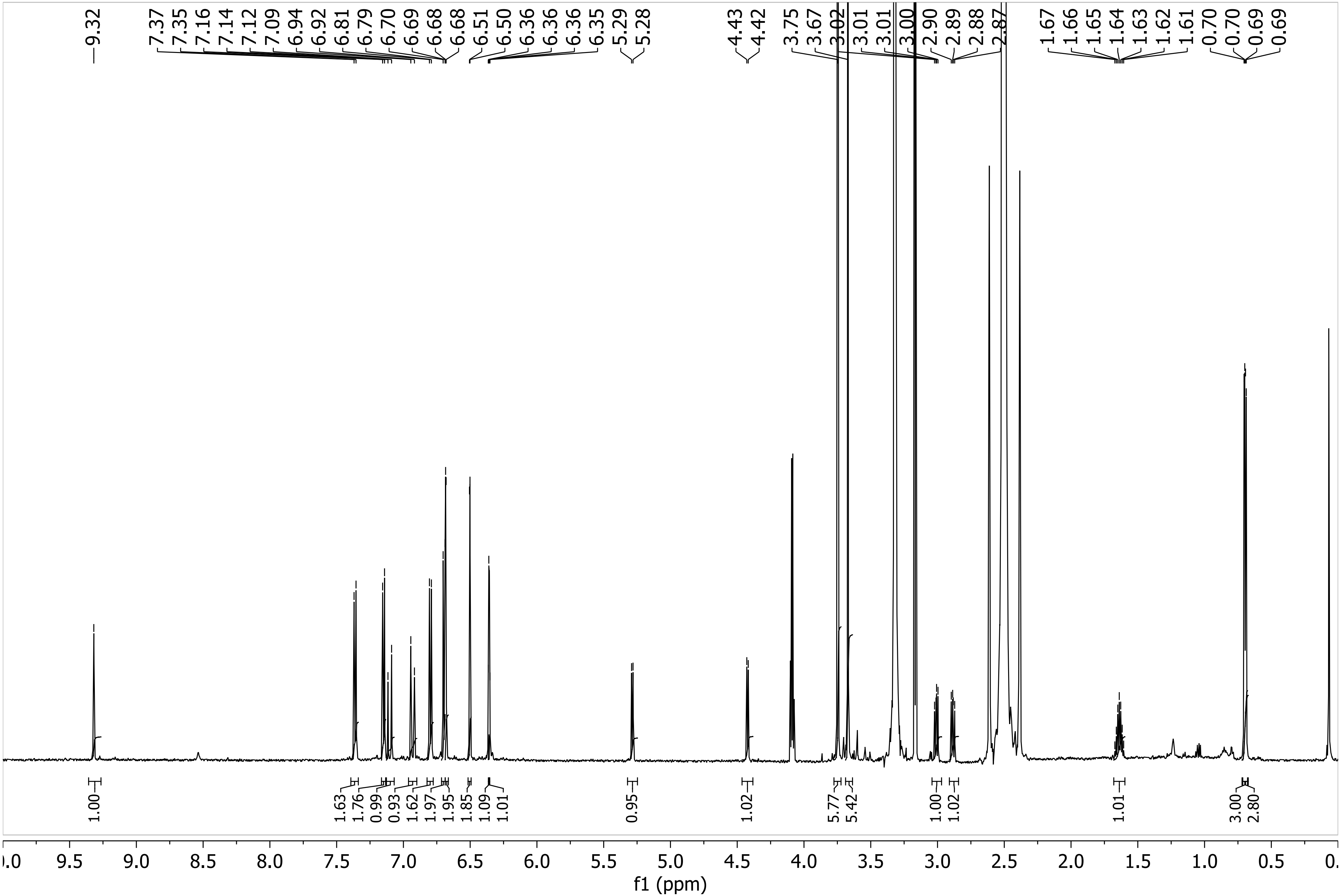

^{1}H NMR spectrum of compound **52** in DMSO-d_6

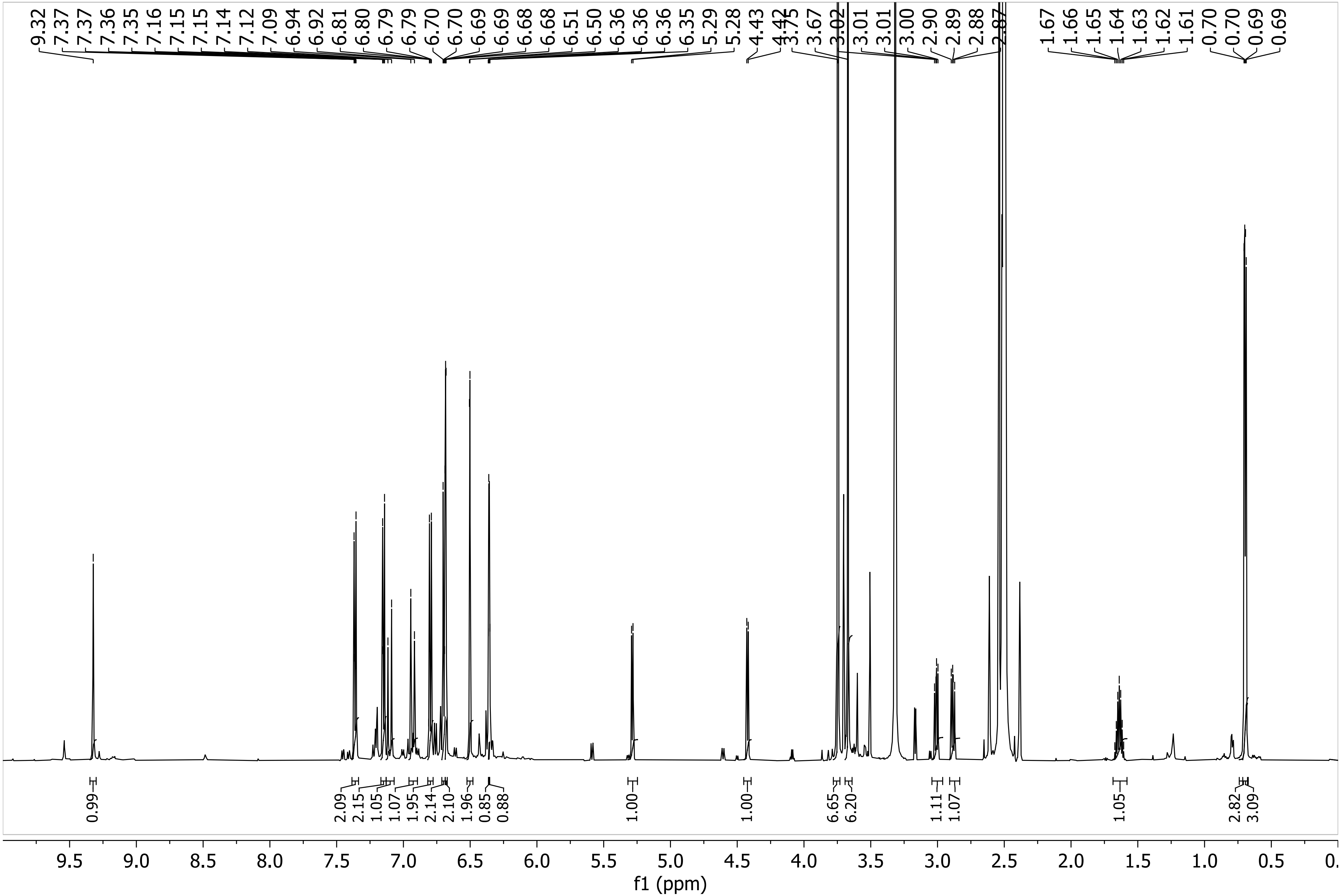

COSY NMR spectrum of compound **52** in DMSO-d_6

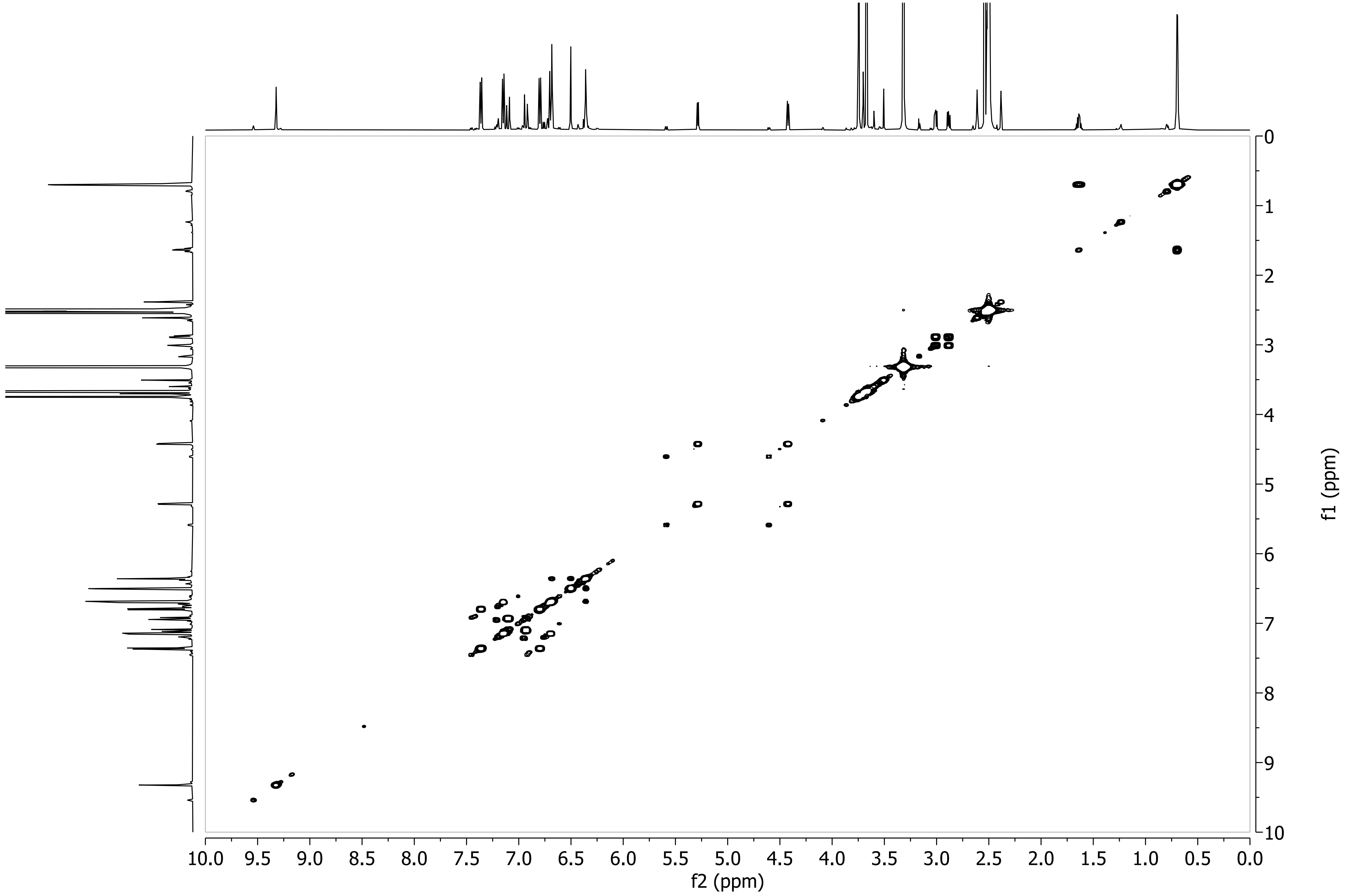

^{13}C-DEPTQ NMR spectrum of compound **52** in DMSO-d_6

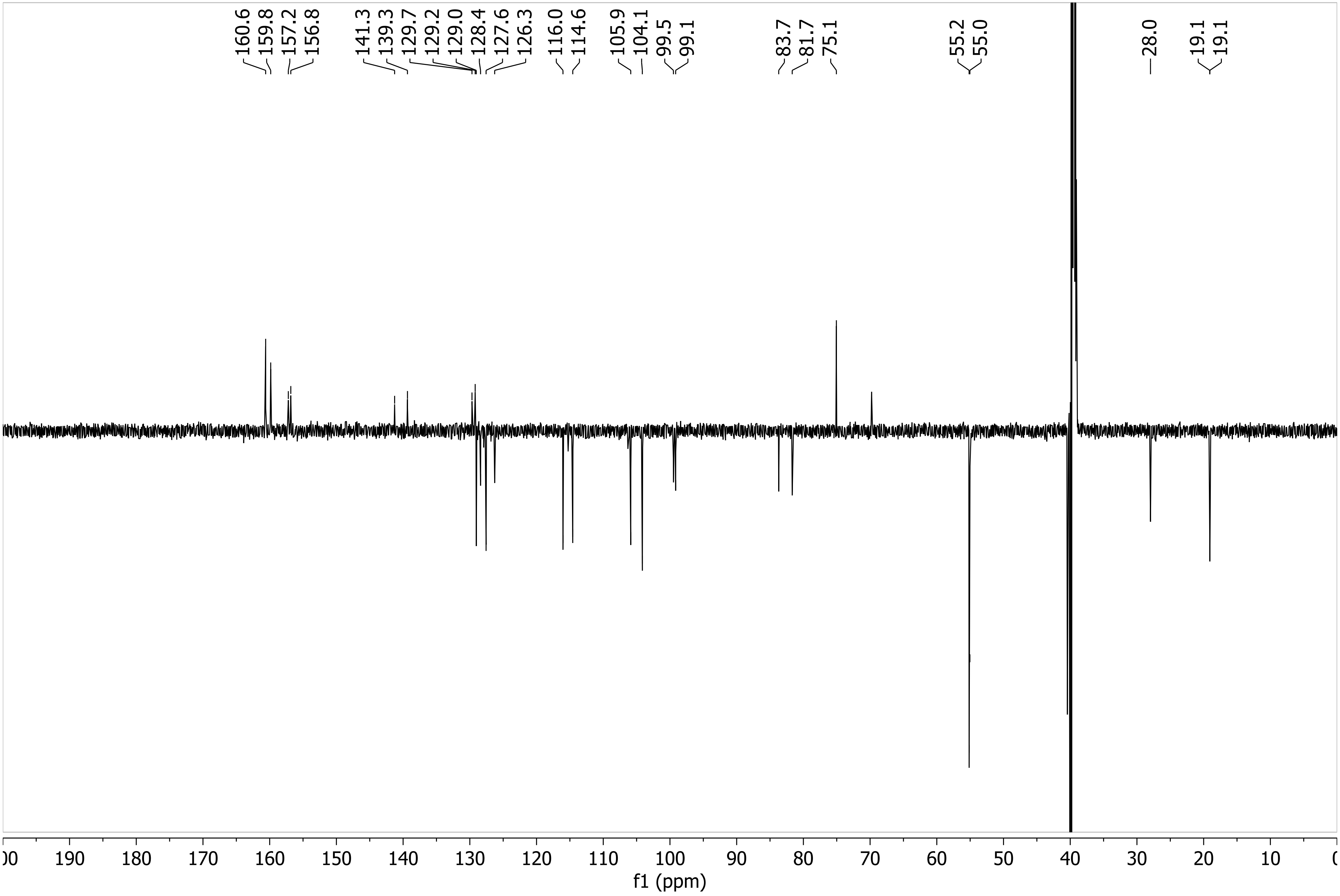

Edited-HSQC NMR spectrum of compound **52** in DMSO-d_6

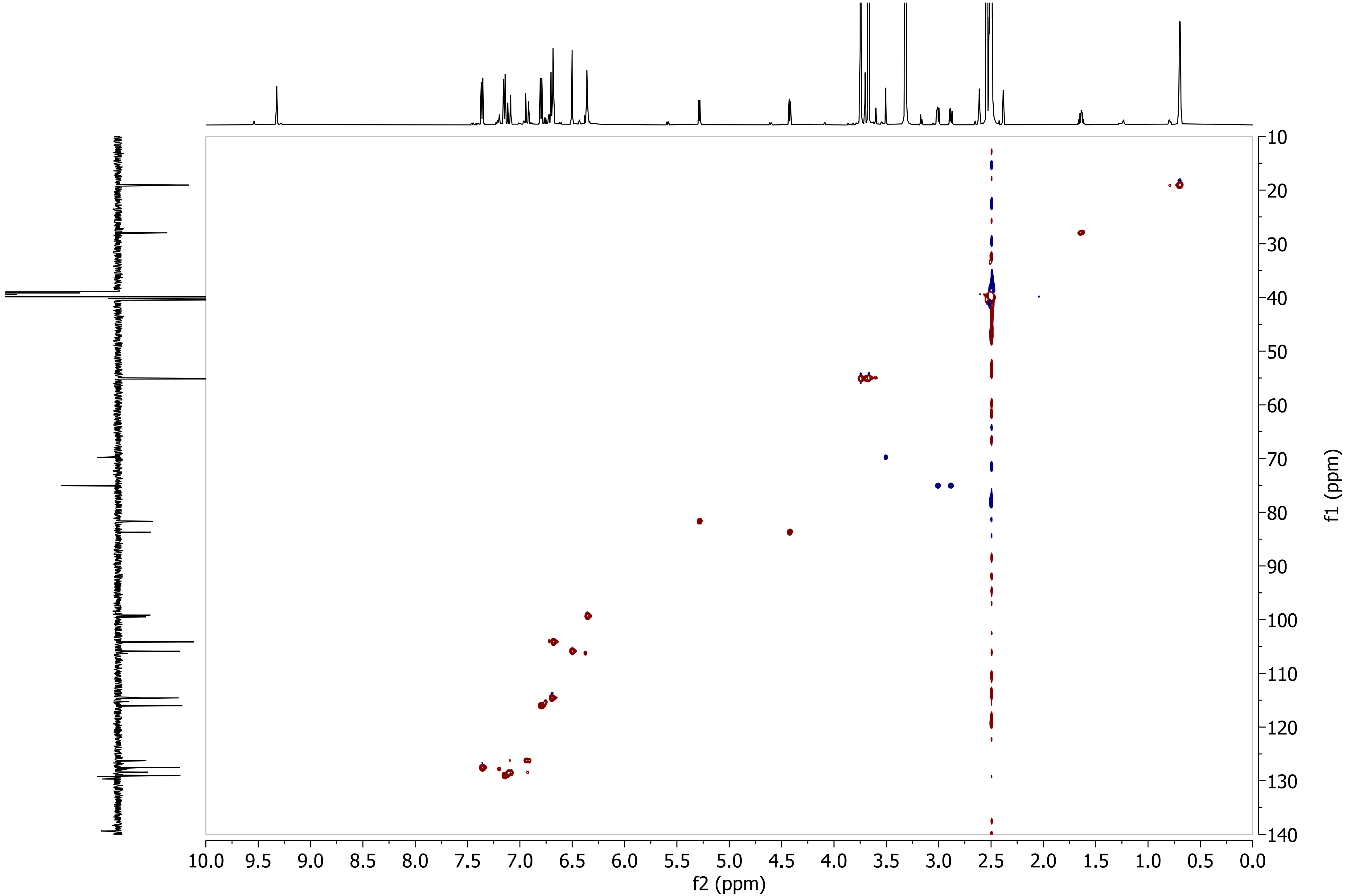

HMBC NMR spectrum of compound **52** in DMSO-d_6

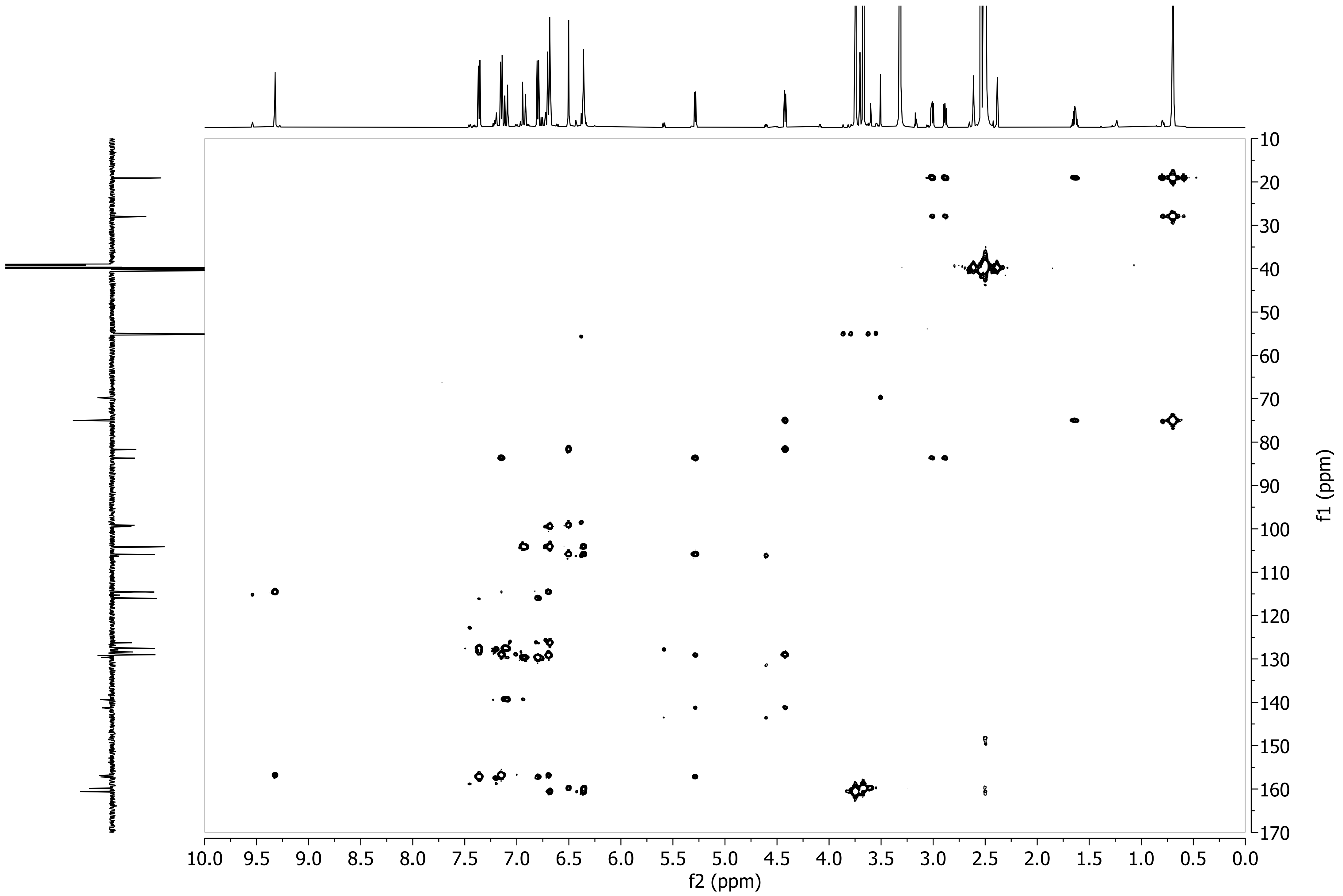

ROESY NMR spectrum of compound **52** in DMSO-d_6

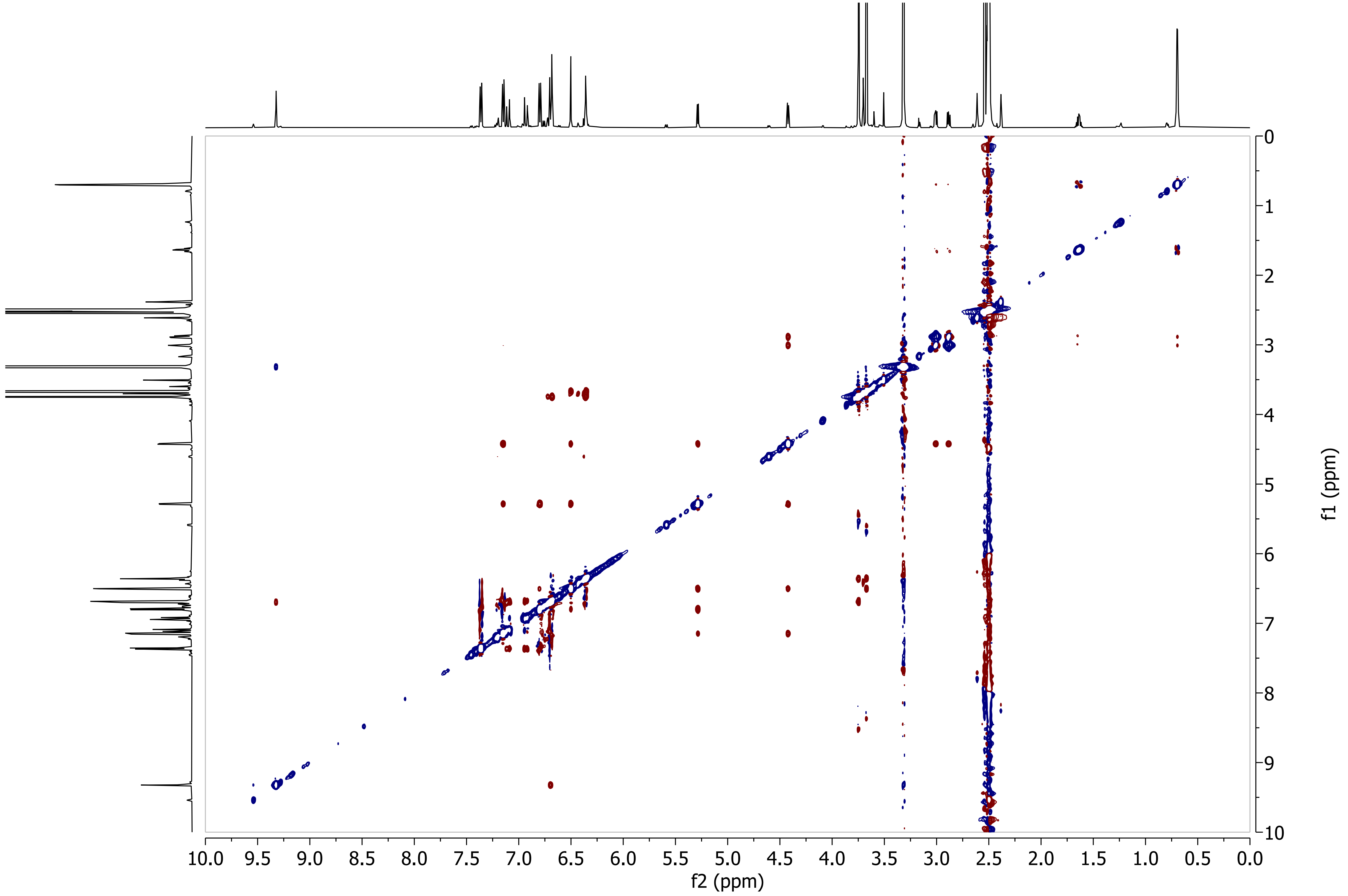

^{1}H NMR spectrum of compound **53** in DMSO-d_6

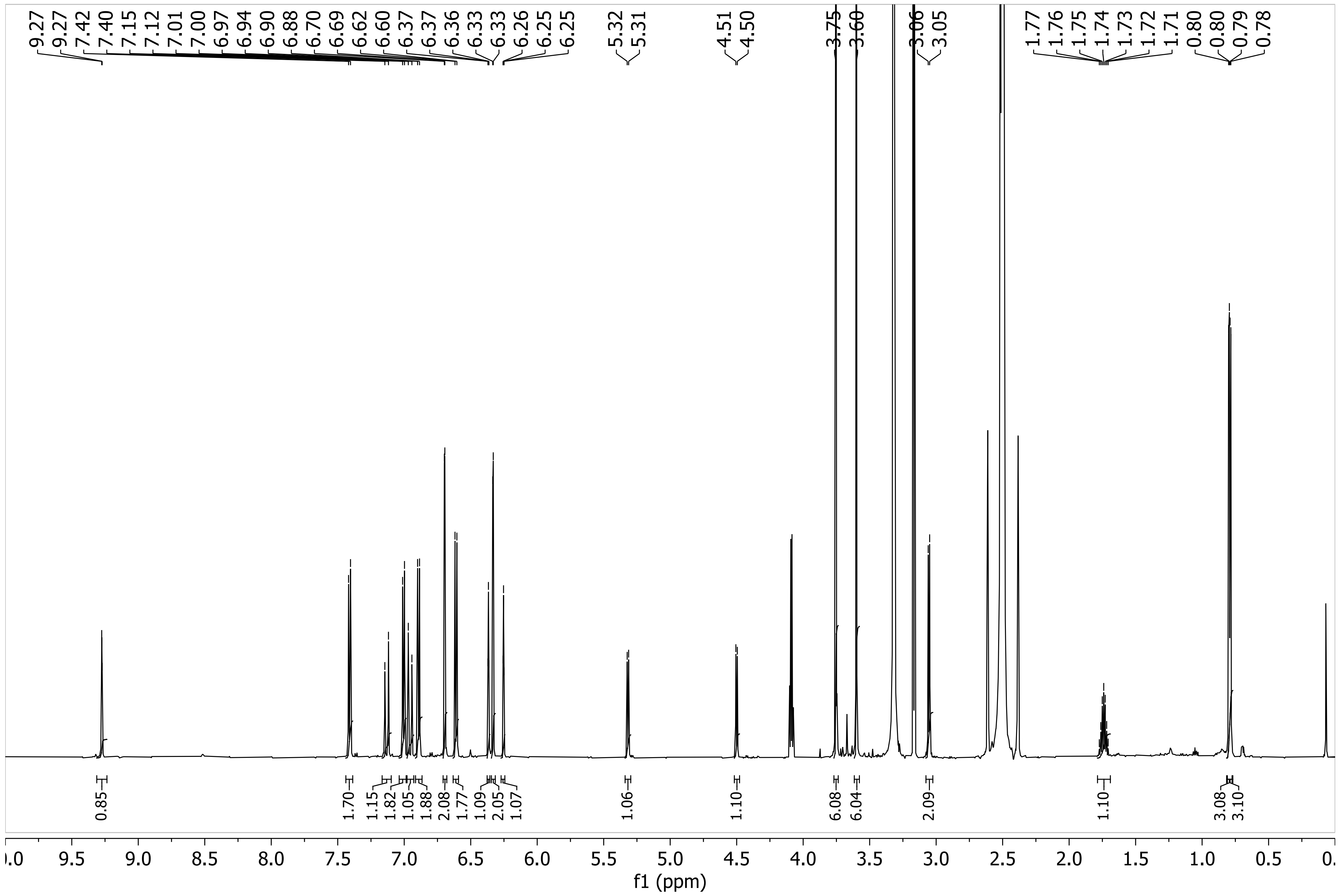

^{1}H NMR spectrum of compound **53** in DMSO-d_6

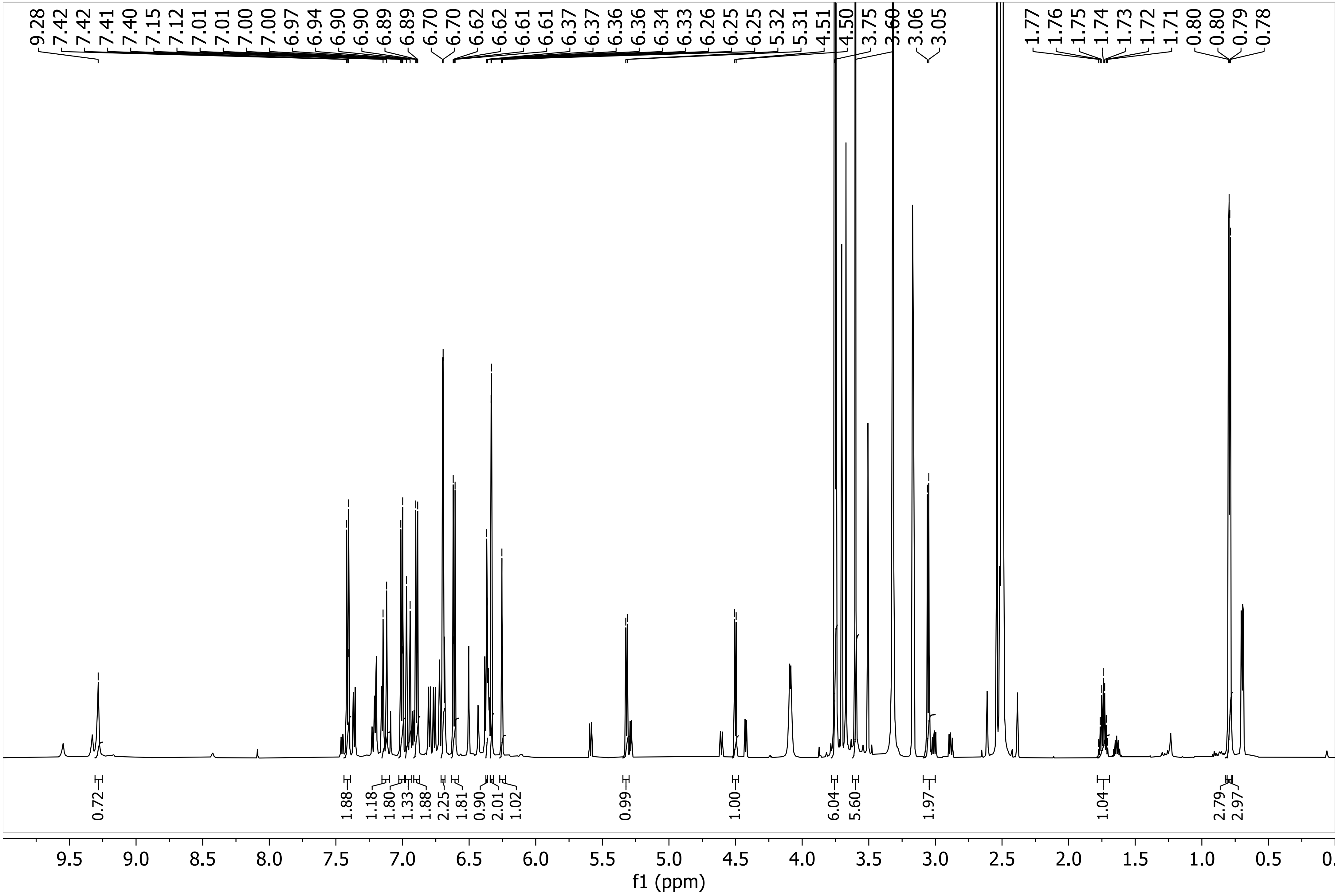

COSY NMR spectrum of compound **53** in DMSO-d_6

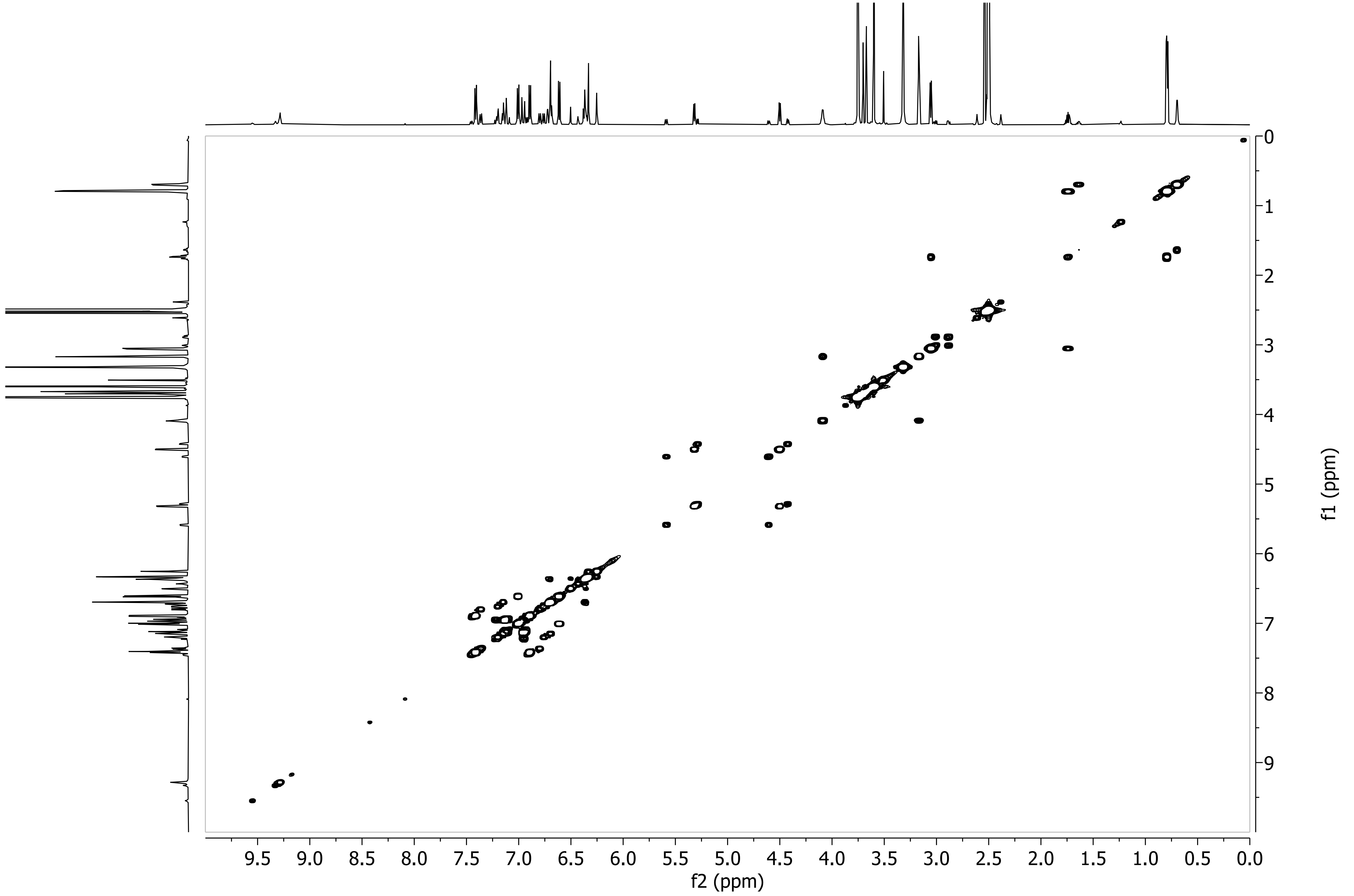

13C-DEPTQ NMR spectrum of compound **53** in DMSO-d_6

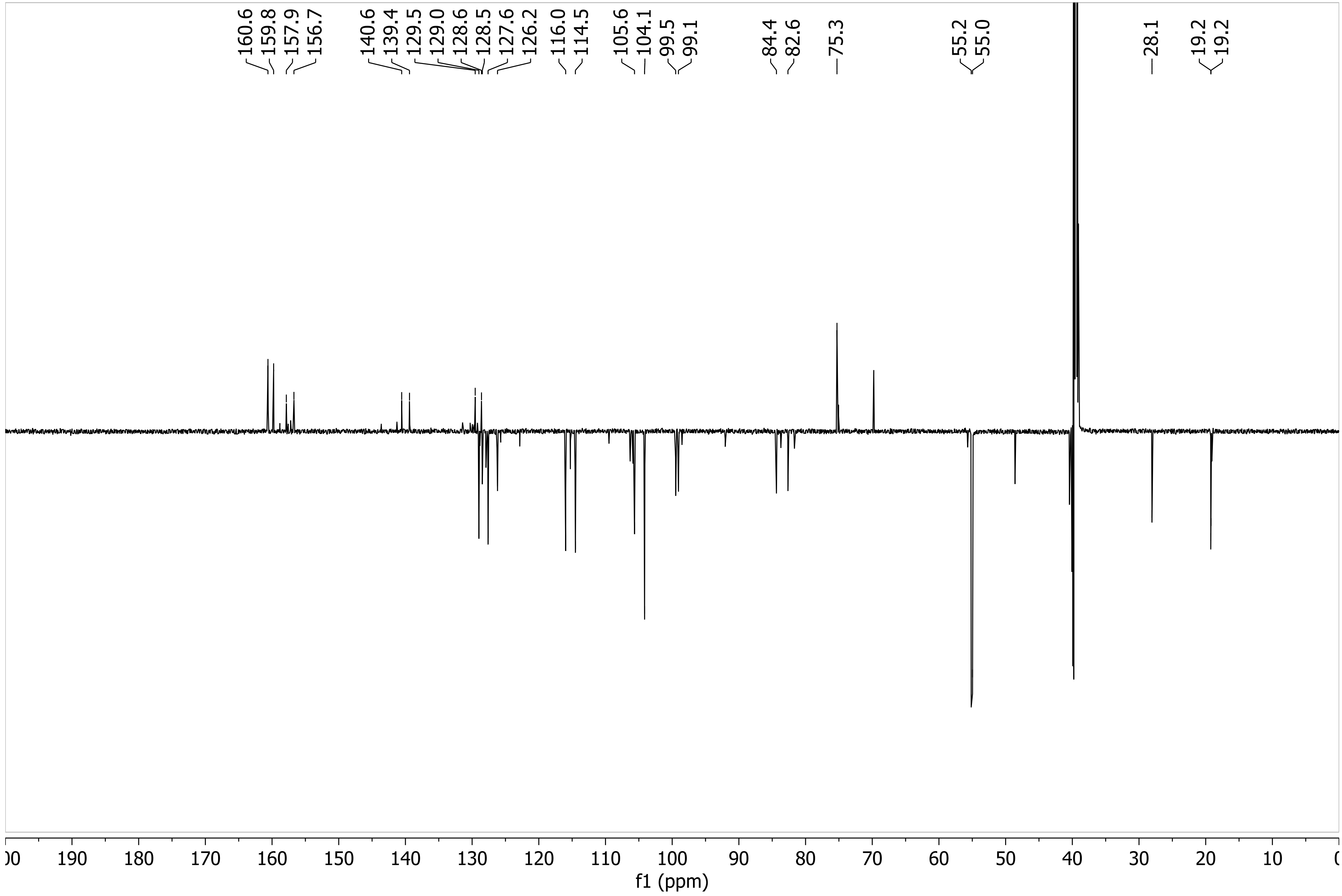

Edited-HSQC NMR spectrum of compound **53** in DMSO-d_6

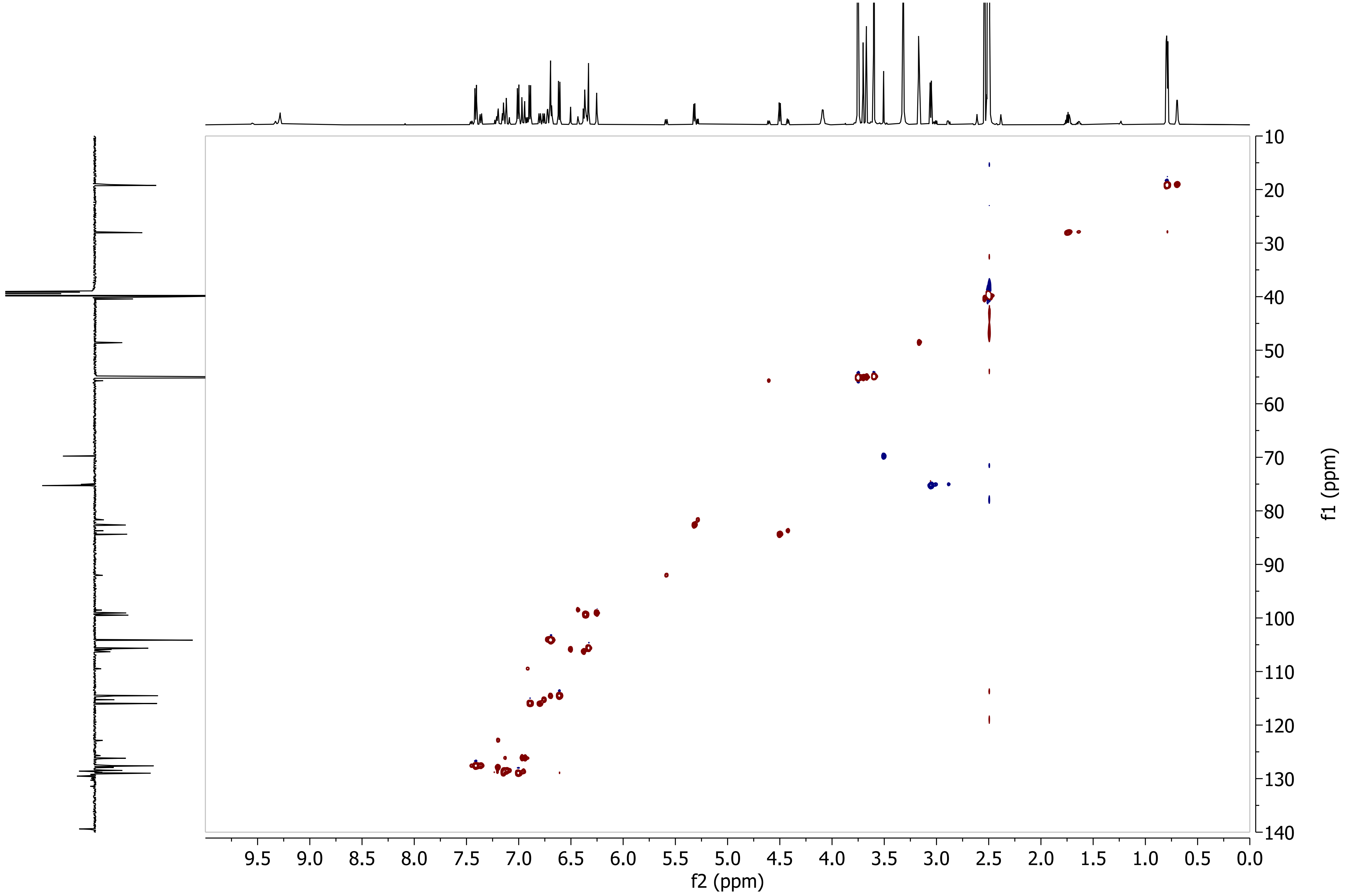

HMBC NMR spectrum of compound **53** in DMSO-d_6

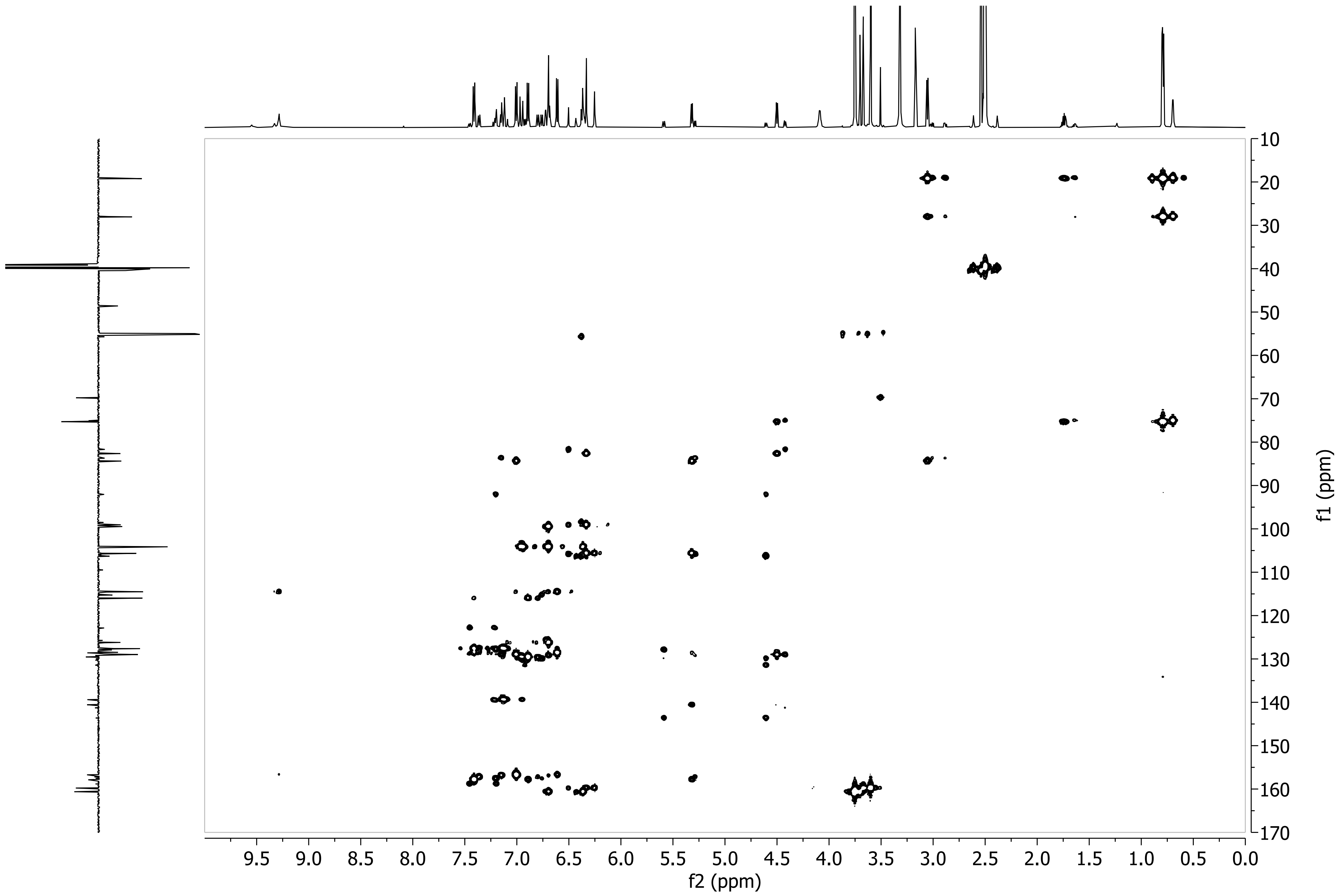

ROESY NMR spectrum of compound **53** in DMSO-d_6

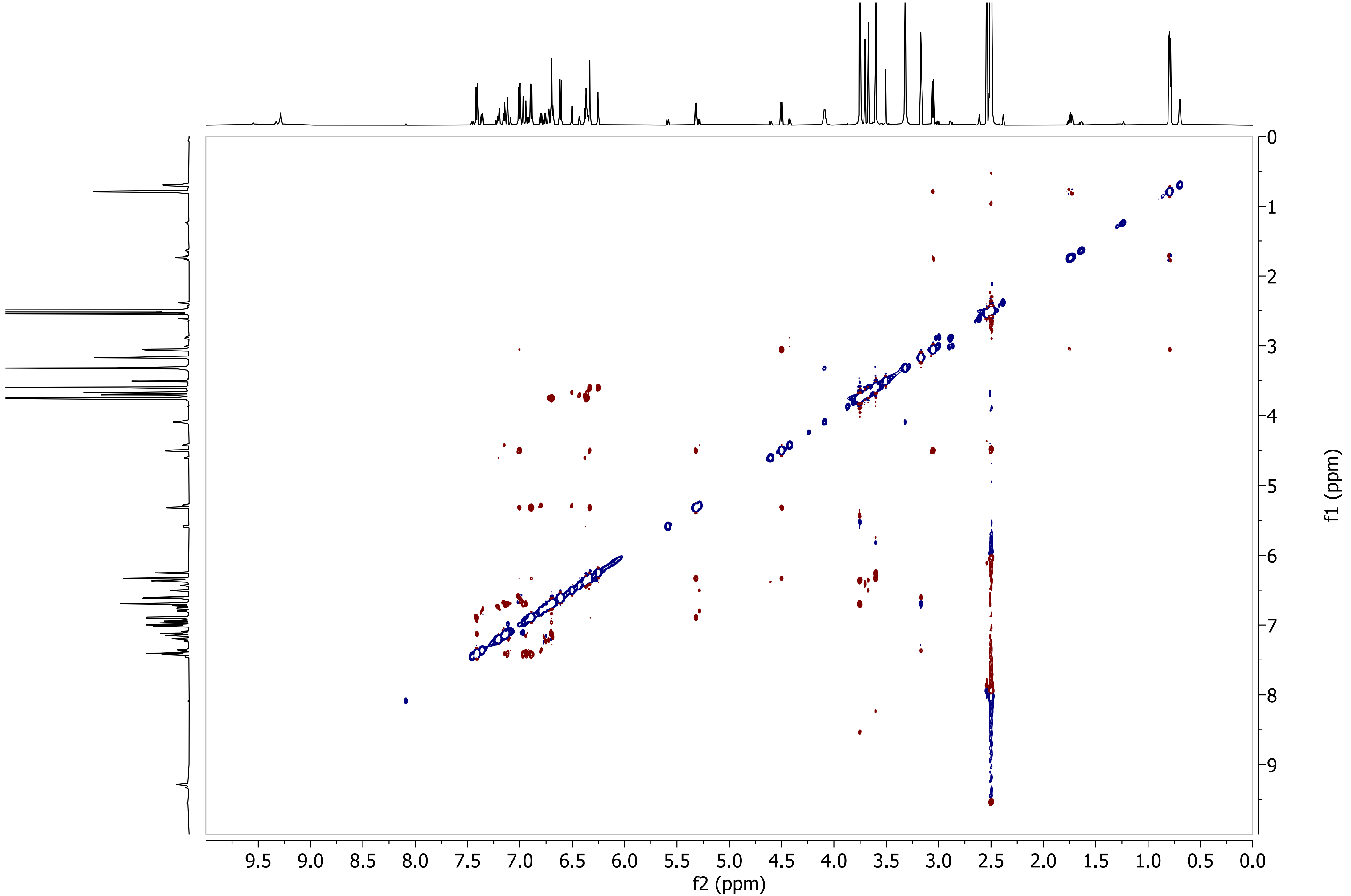

^1H NMR spectrum of compound **54** in DMSO-d_6

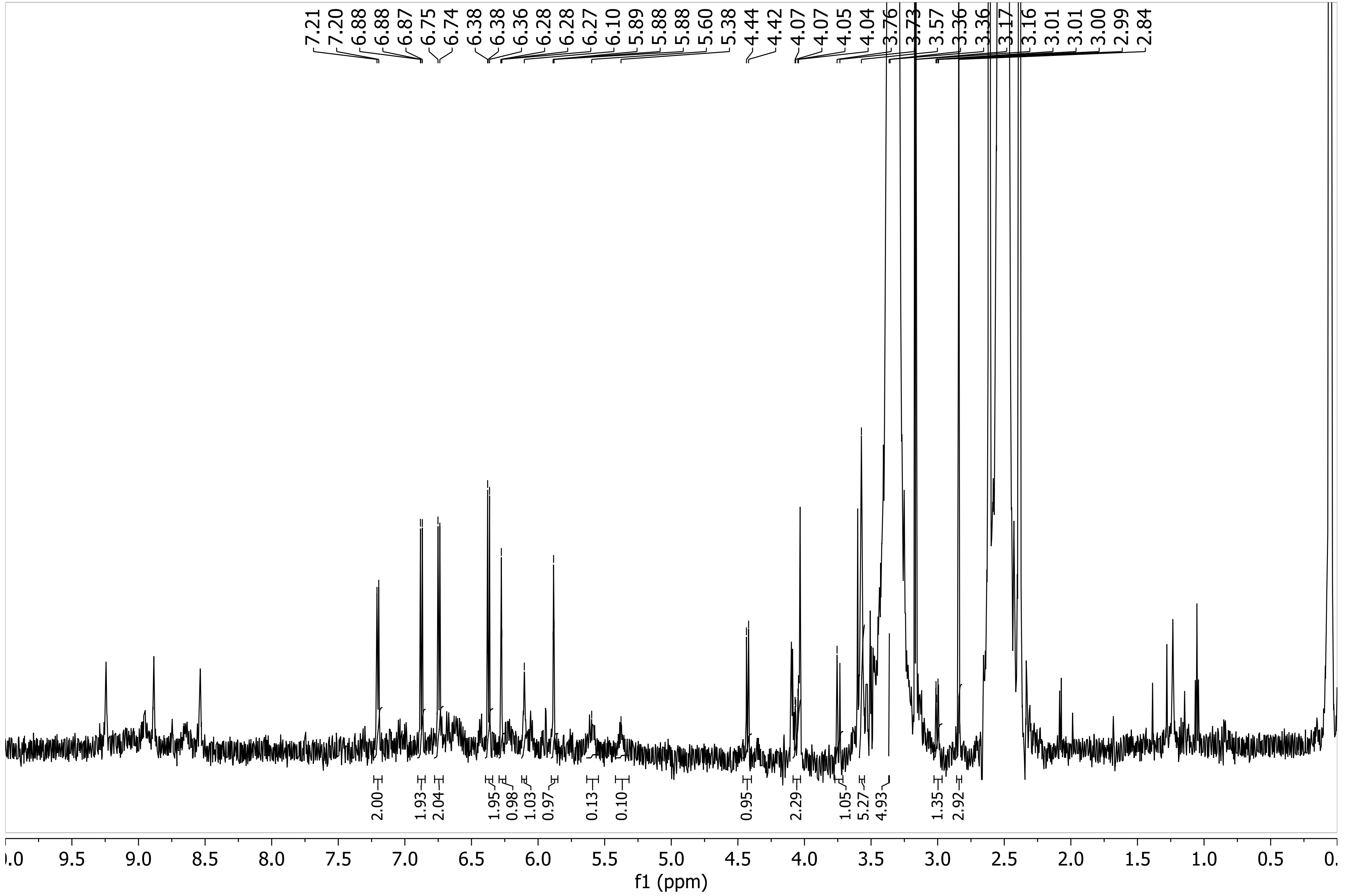

^{1}H NMR spectrum of compound **55** in DMSO-d_6

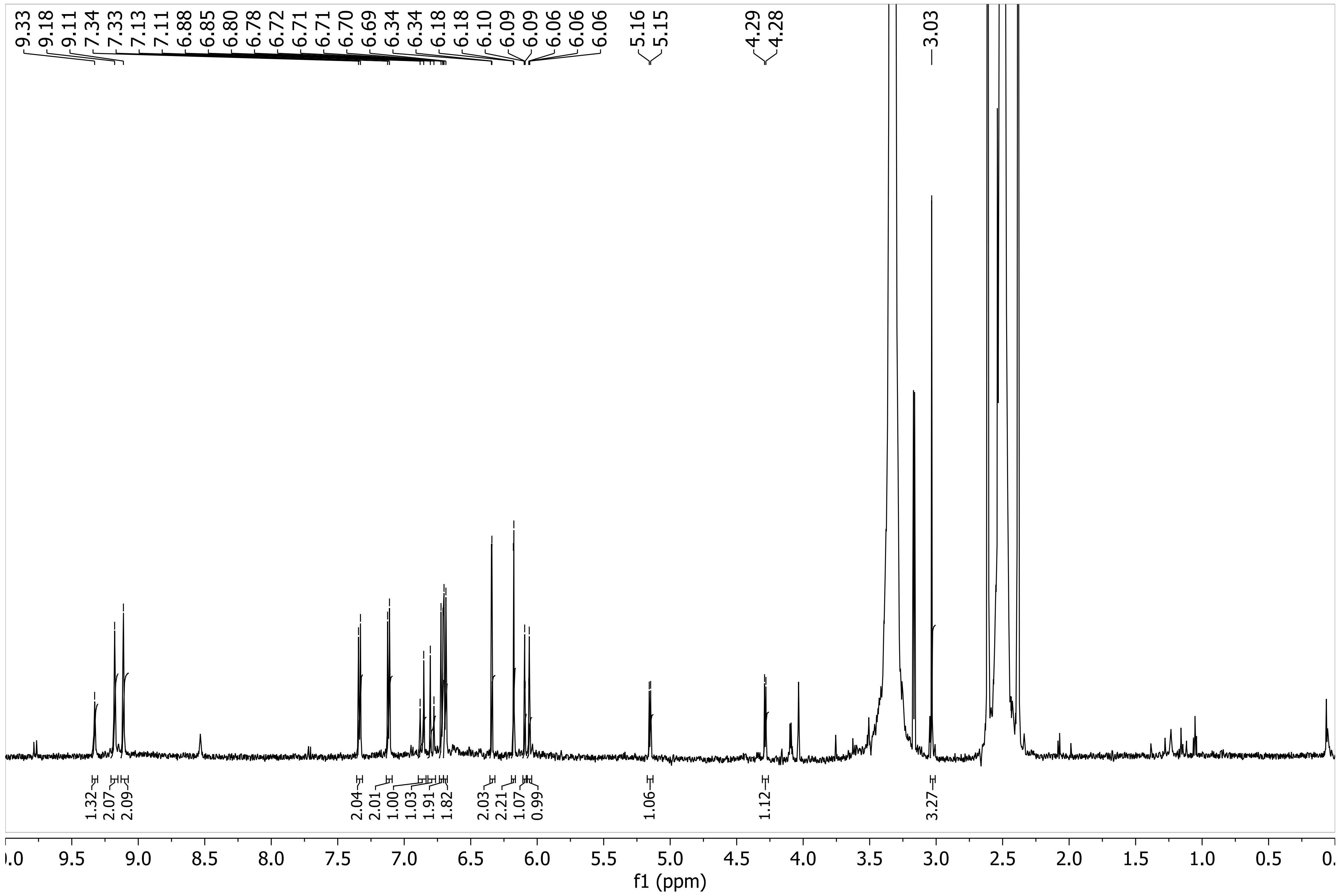

COSY NMR spectrum of compound **55** in DMSO-d_6

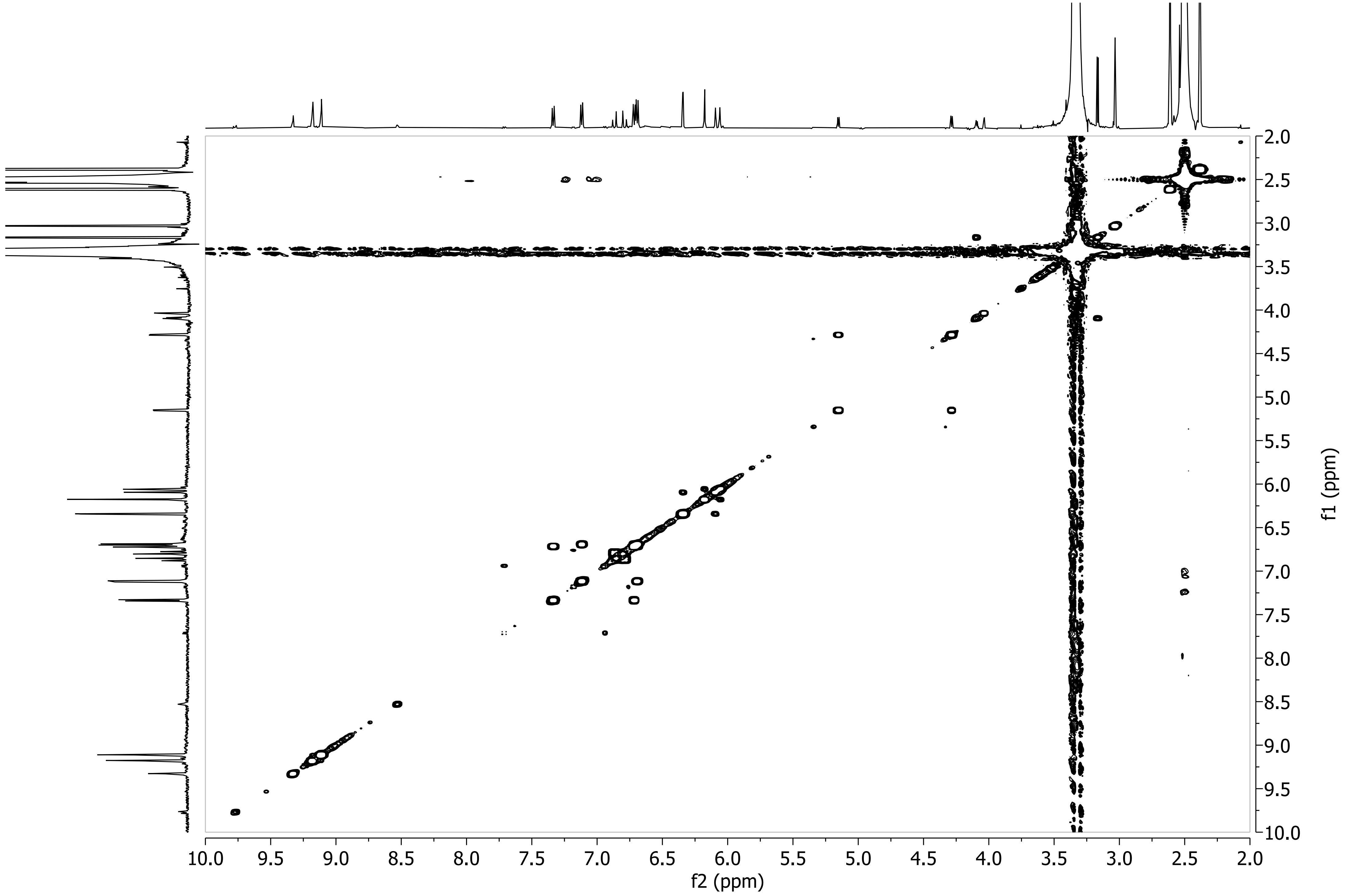

Edited-HSQC NMR spectrum of compound **55** in DMSO-d_6

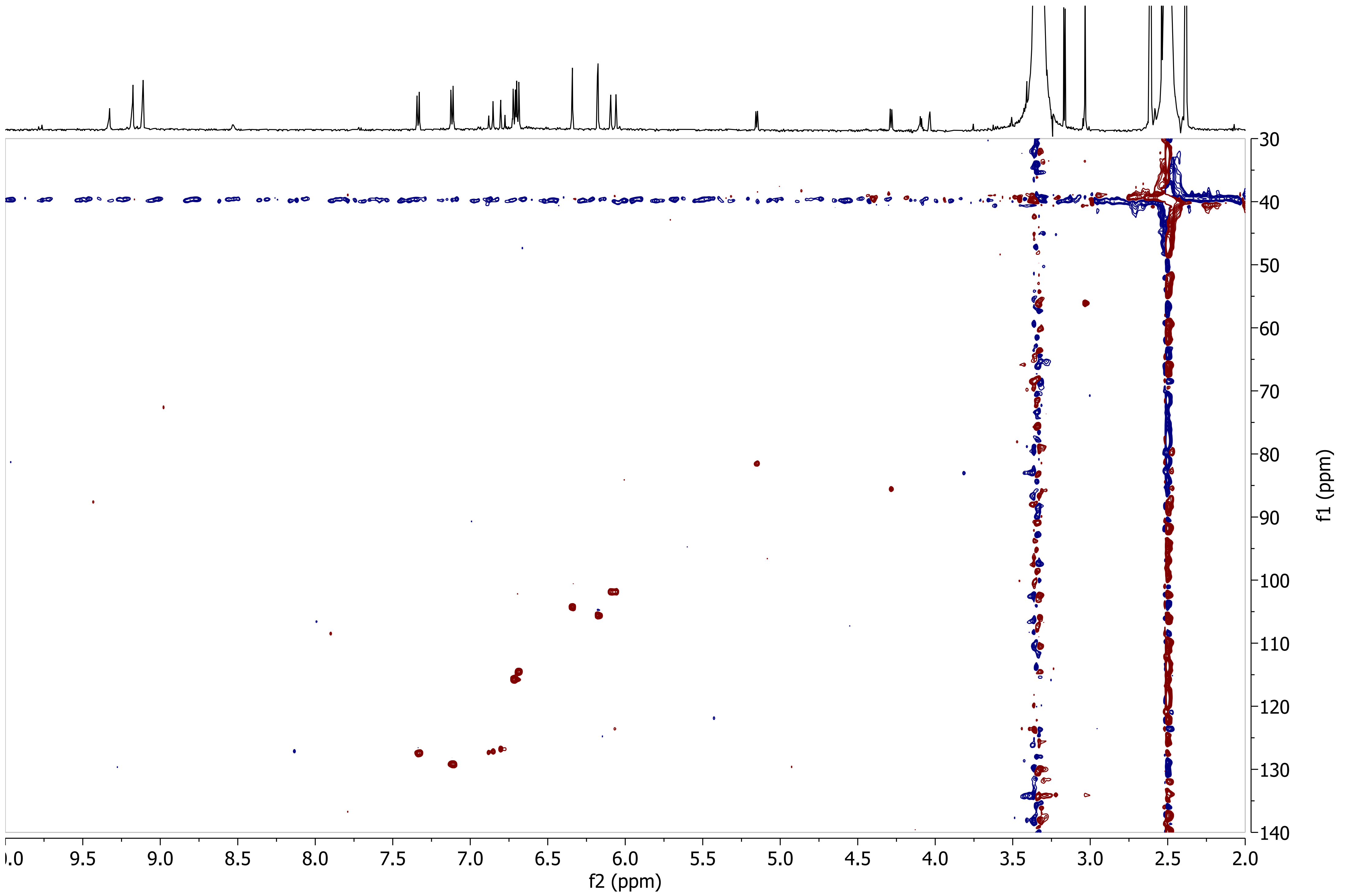

HMBC NMR spectrum of compound **55** in DMSO-d_6

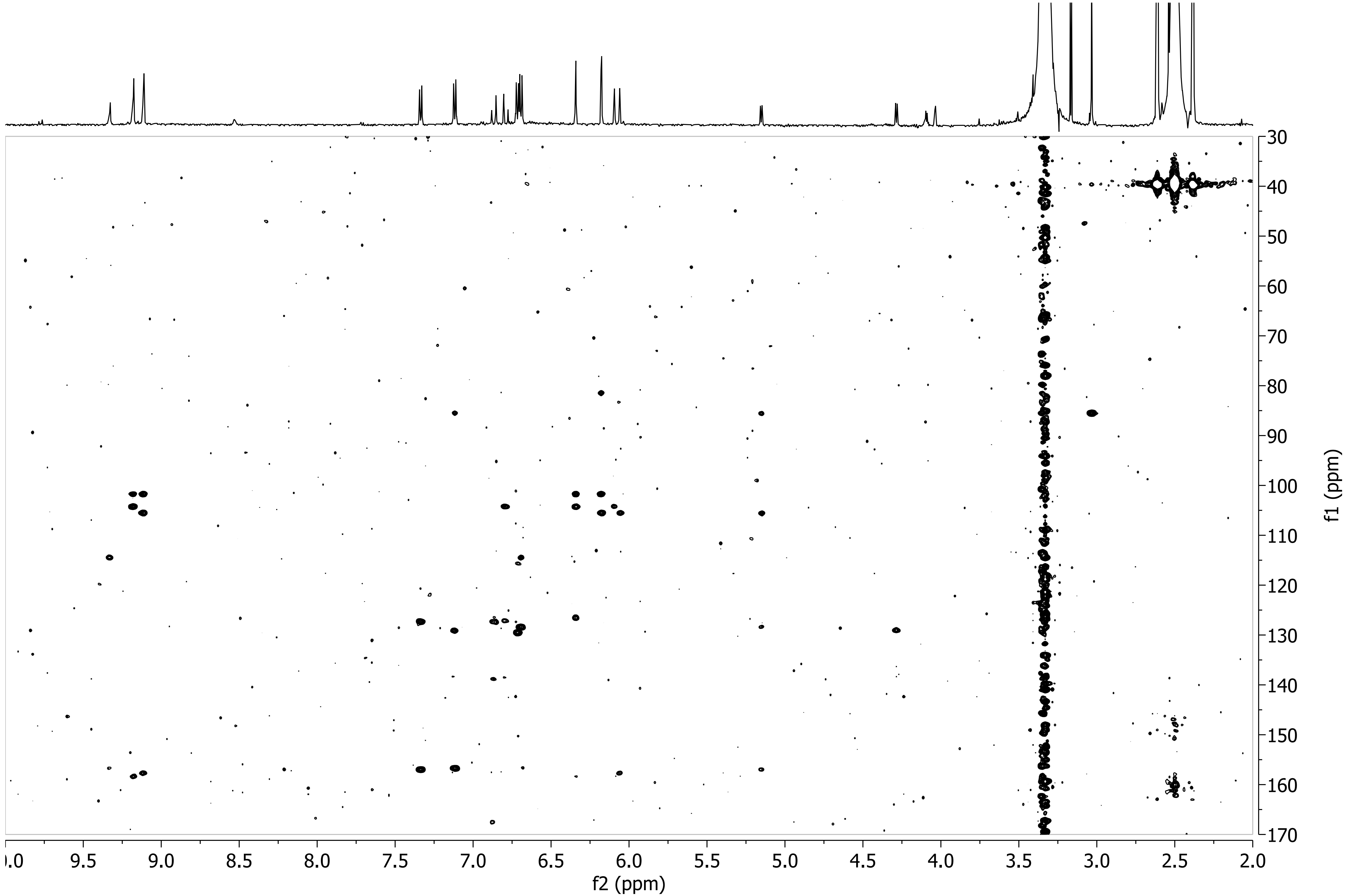

ROESY NMR spectrum of compound **55** in DMSO-d_6

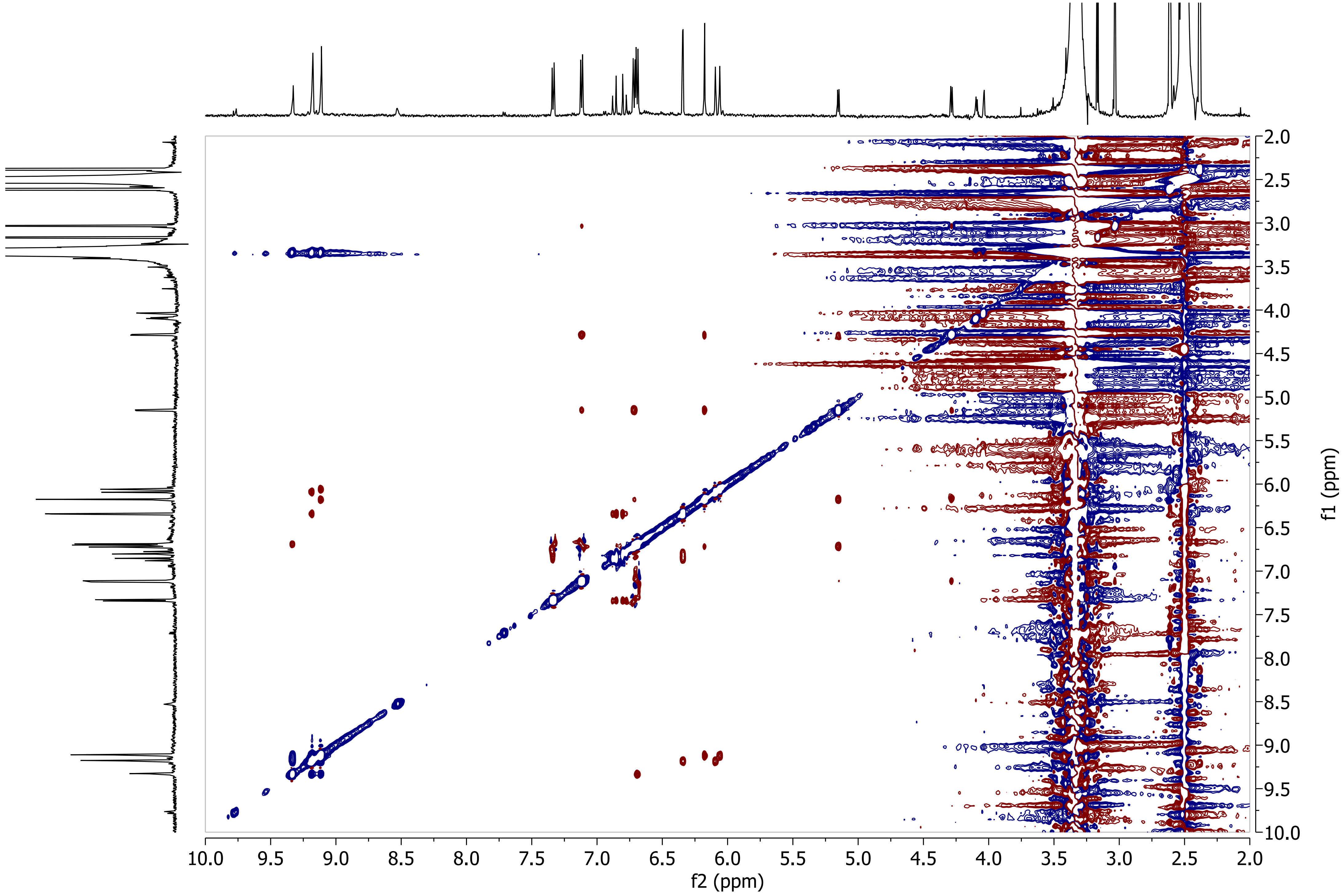

^{1}H NMR spectrum of compound **56** in DMSO-d_6

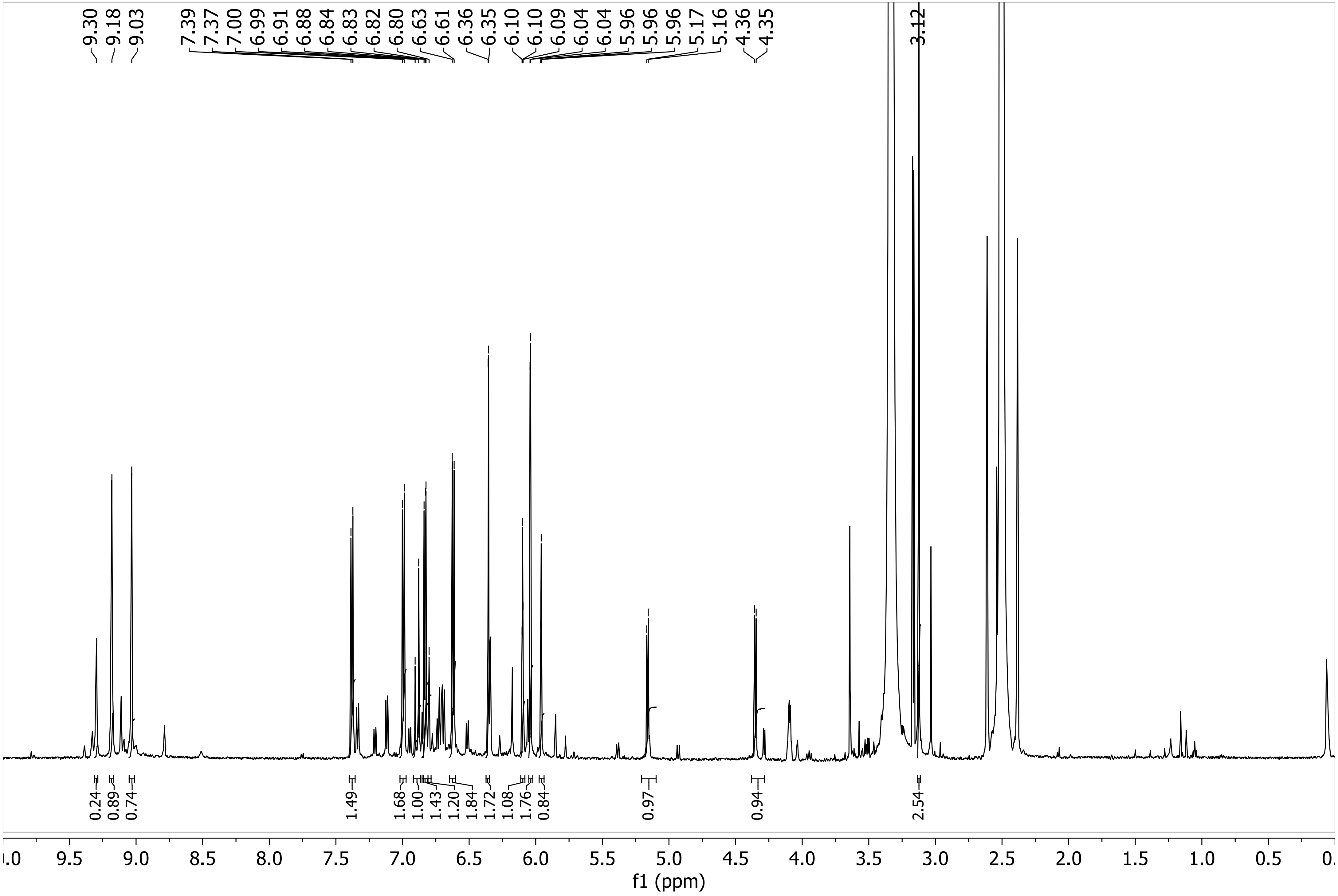

COSY NMR spectrum of compound **56** in DMSO-d_6

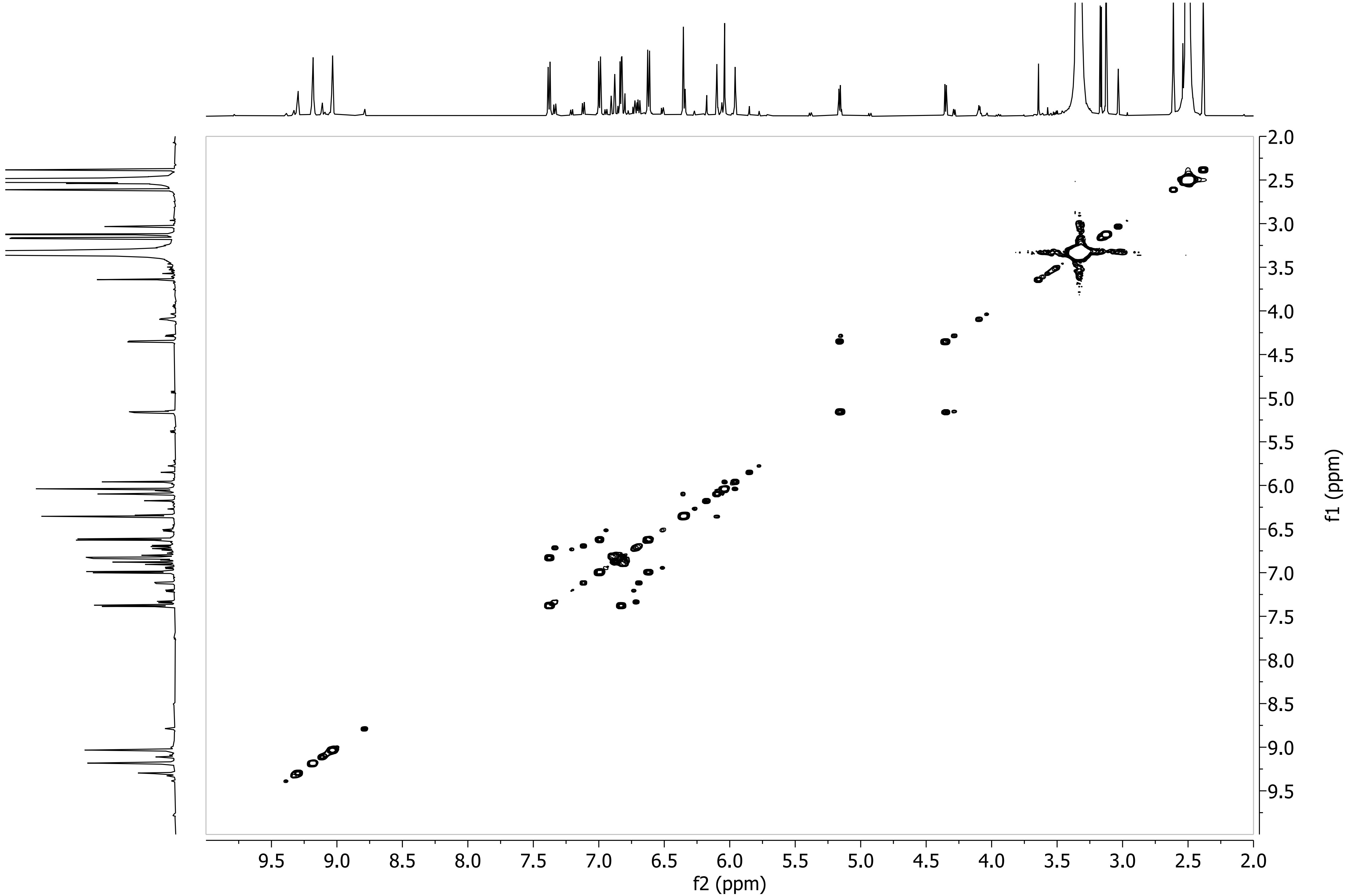

Edited-HSQC NMR spectrum of compound **56** in DMSO-d_6

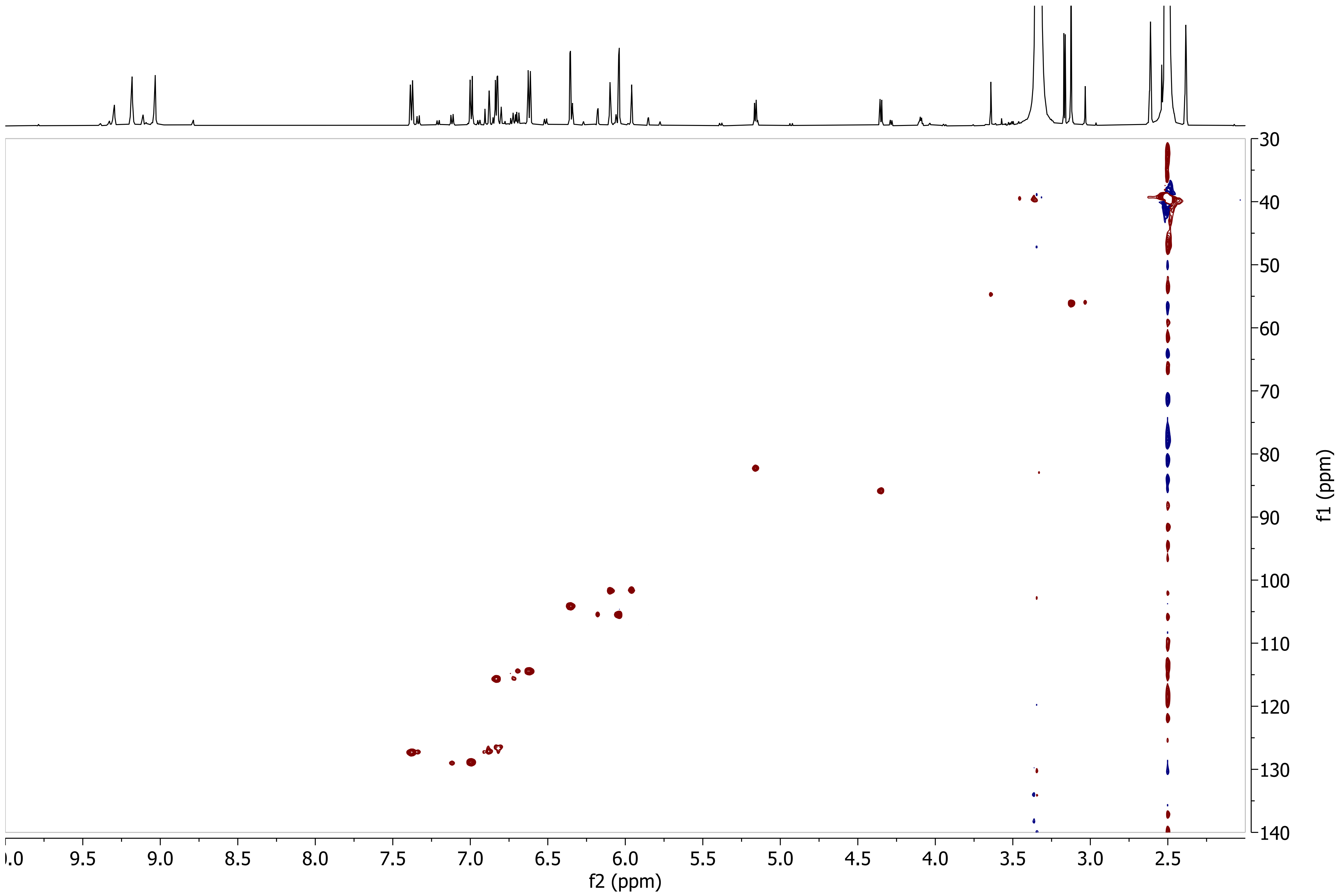

HMBC NMR spectrum of compound **56** in DMSO-d_6

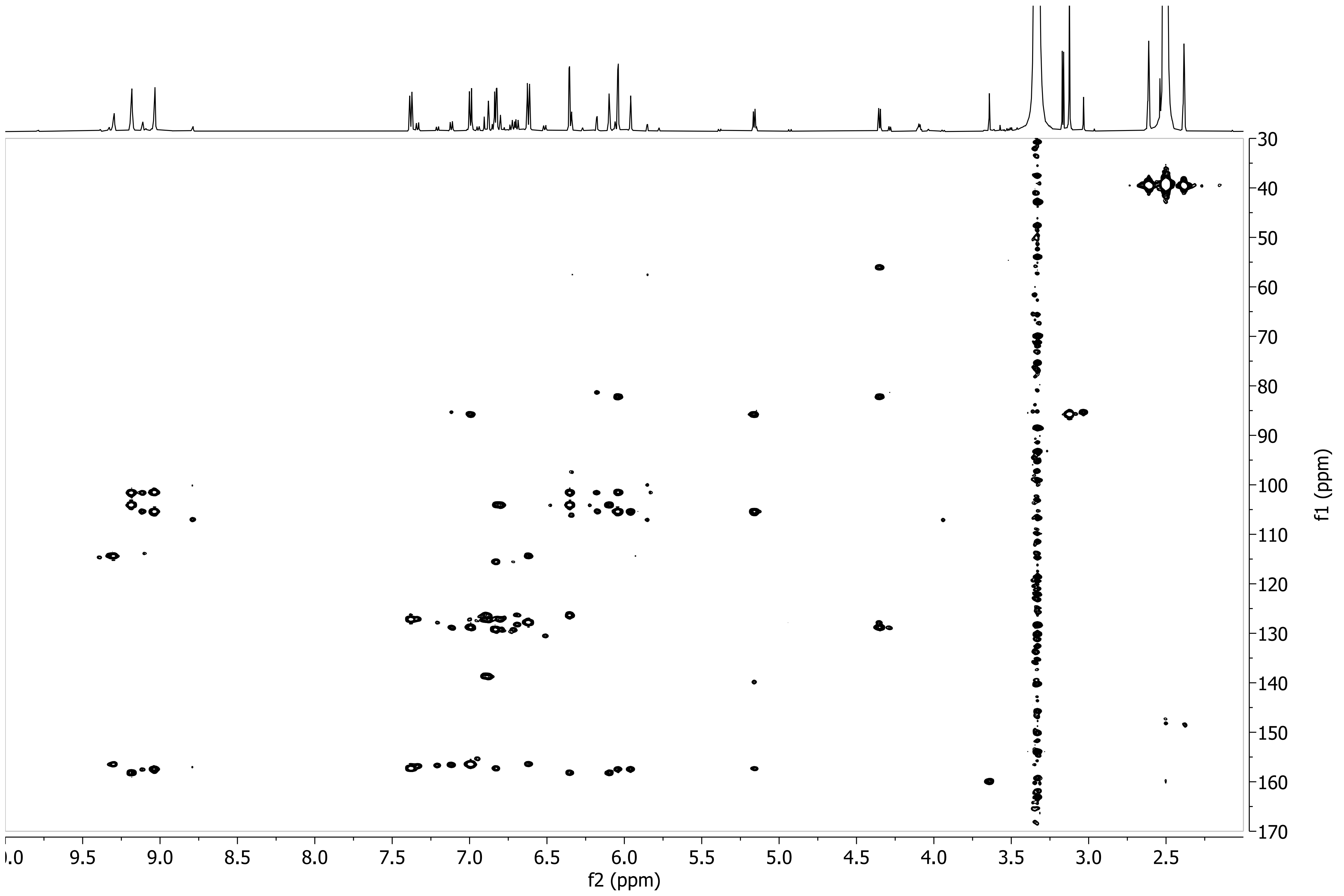

ROESY NMR spectrum of compound **56** in DMSO-d_6

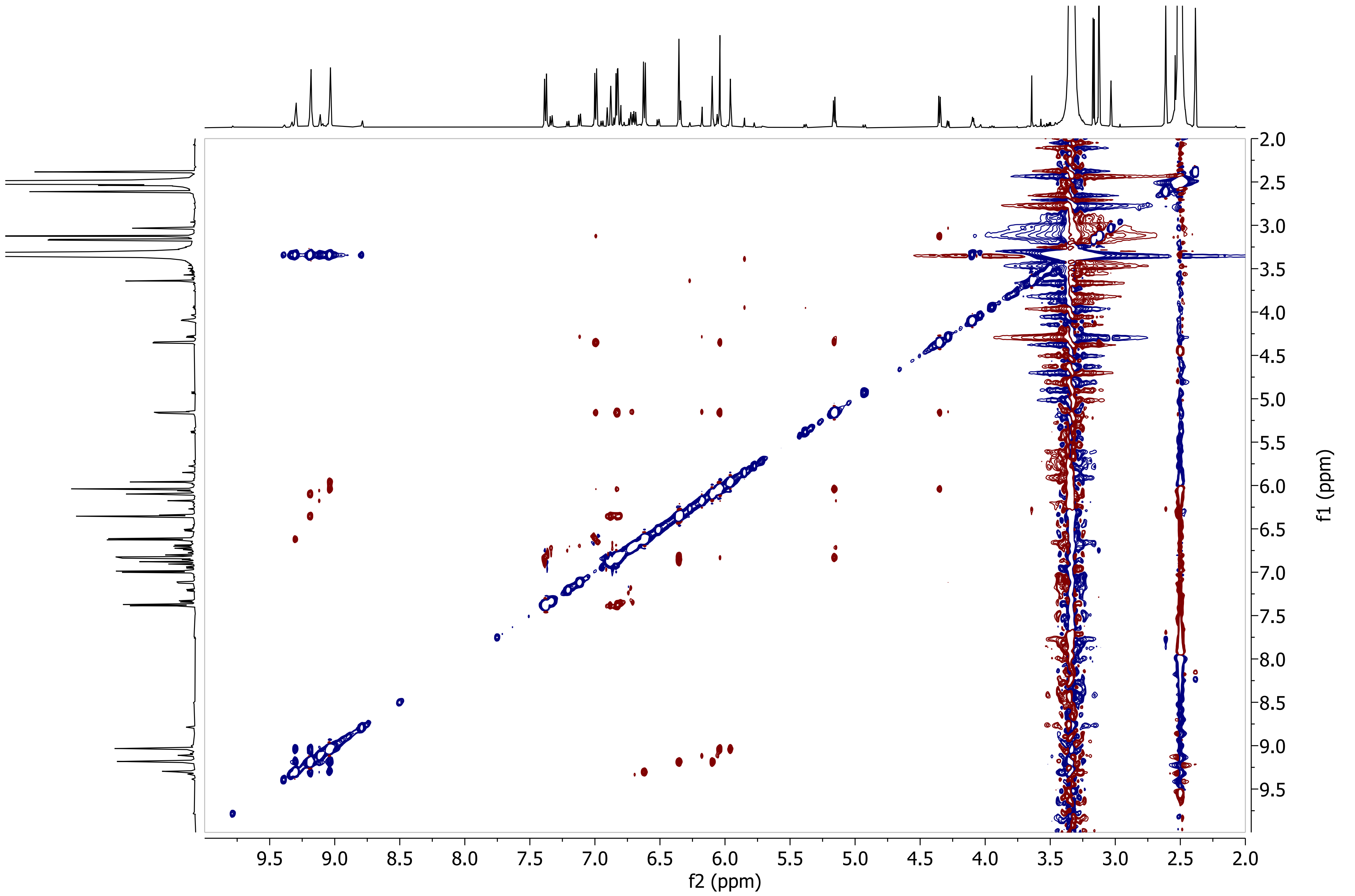

^{1}H NMR spectrum of compound **57** in DMSO-d_6

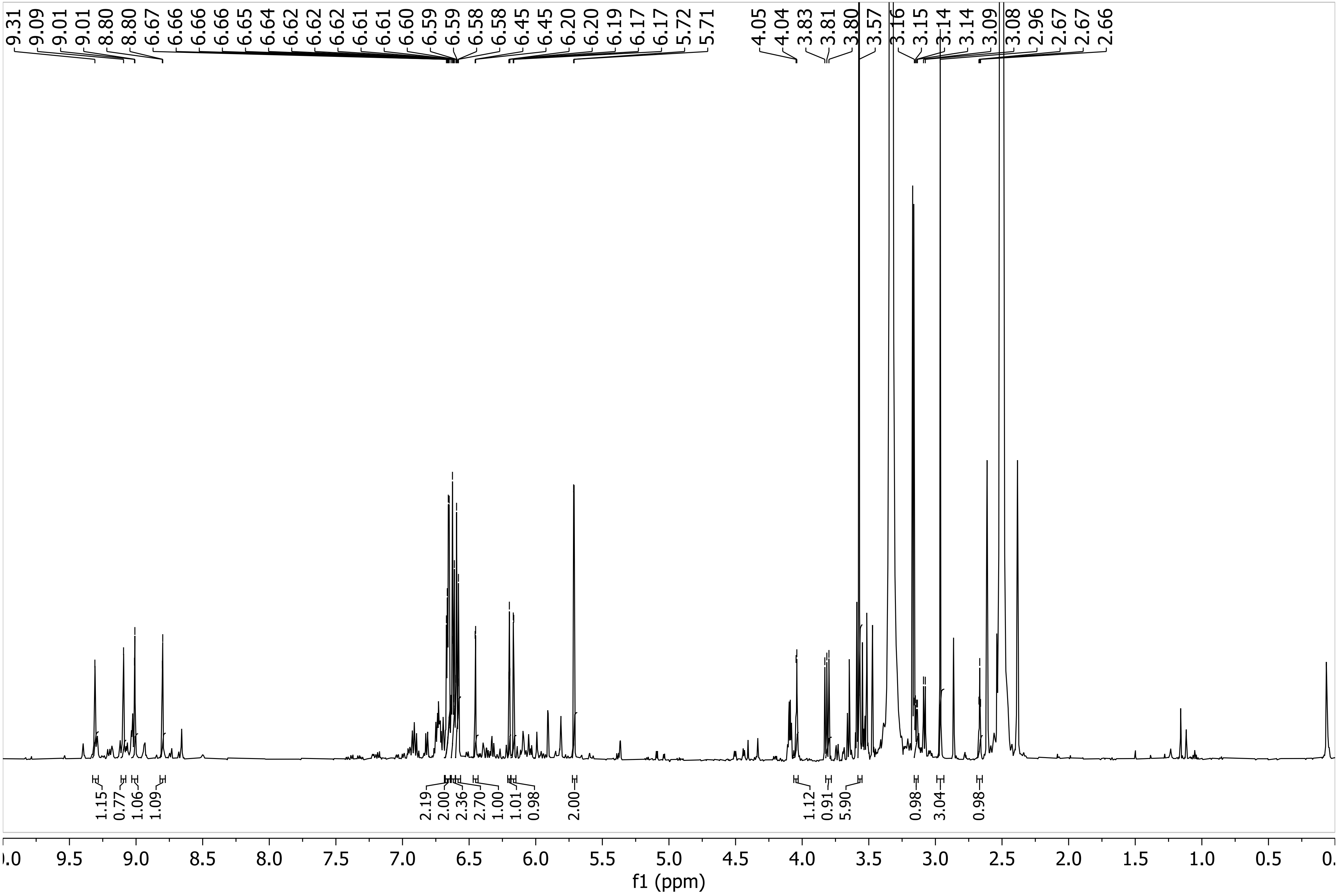

^{1}H NMR spectrum of compound **58** in DMSO-d_6

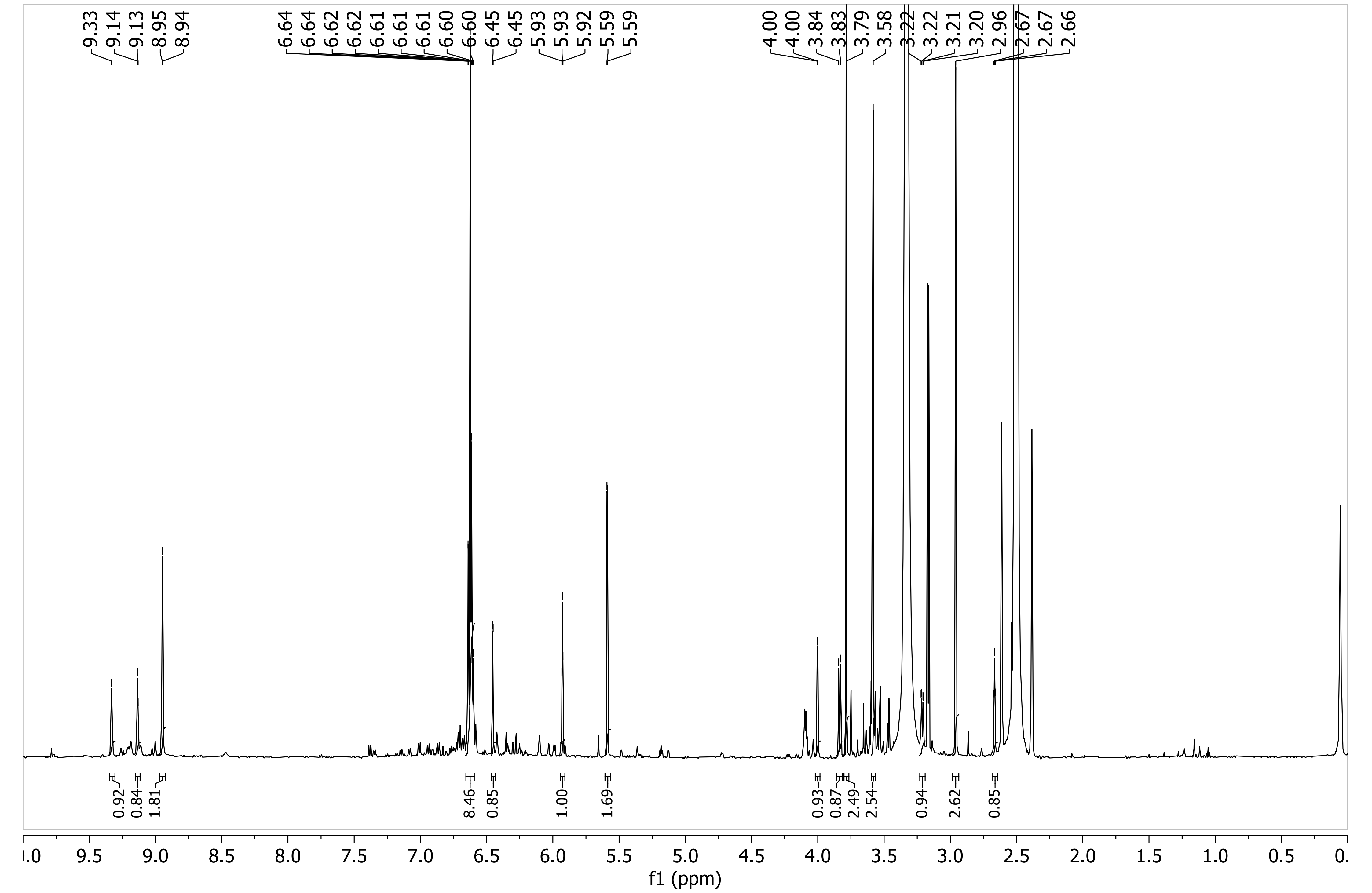

^{1}H NMR spectrum of compound **59** in DMSO-d_6

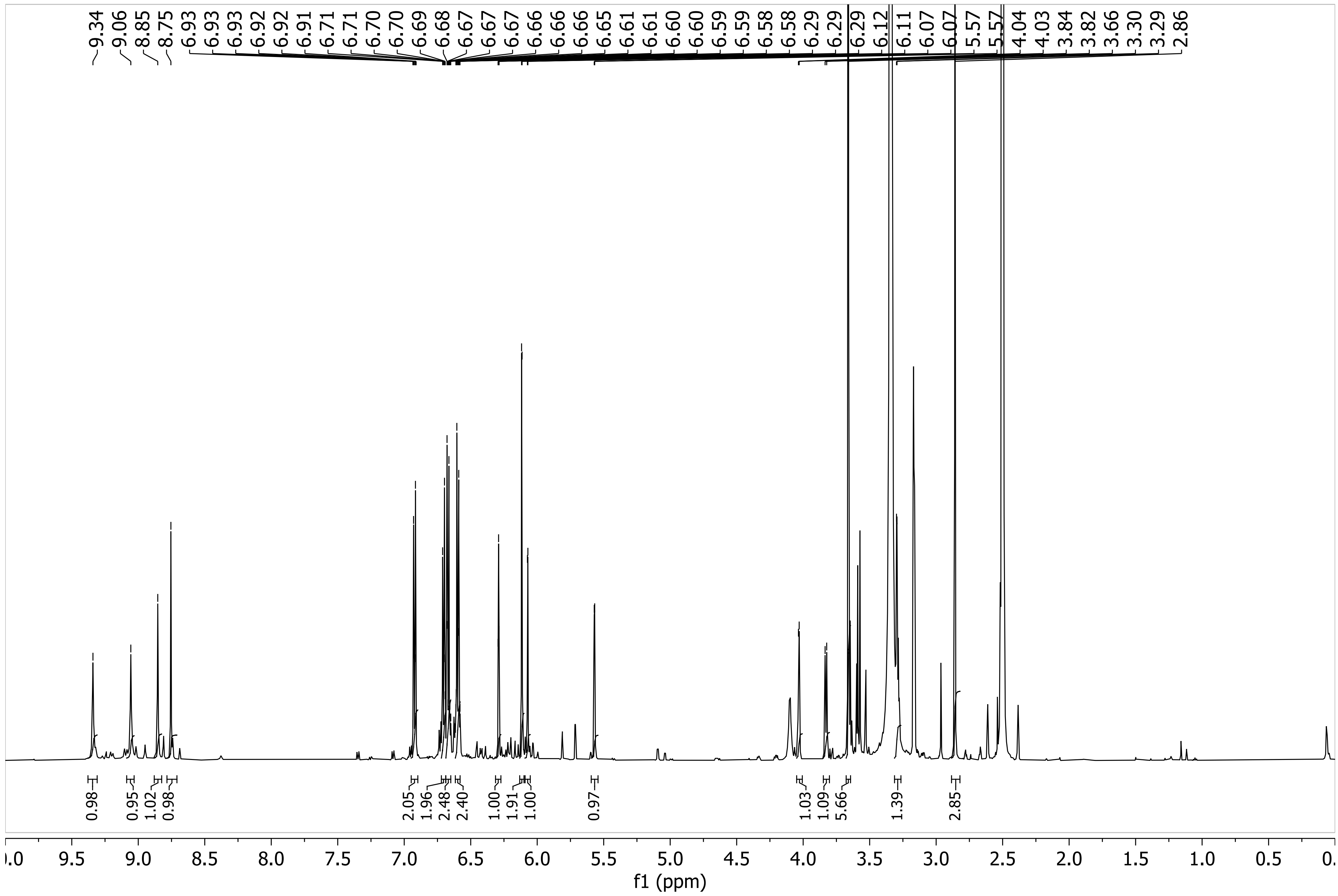

^{1}H NMR spectrum of compound **60** in DMSO-d_6

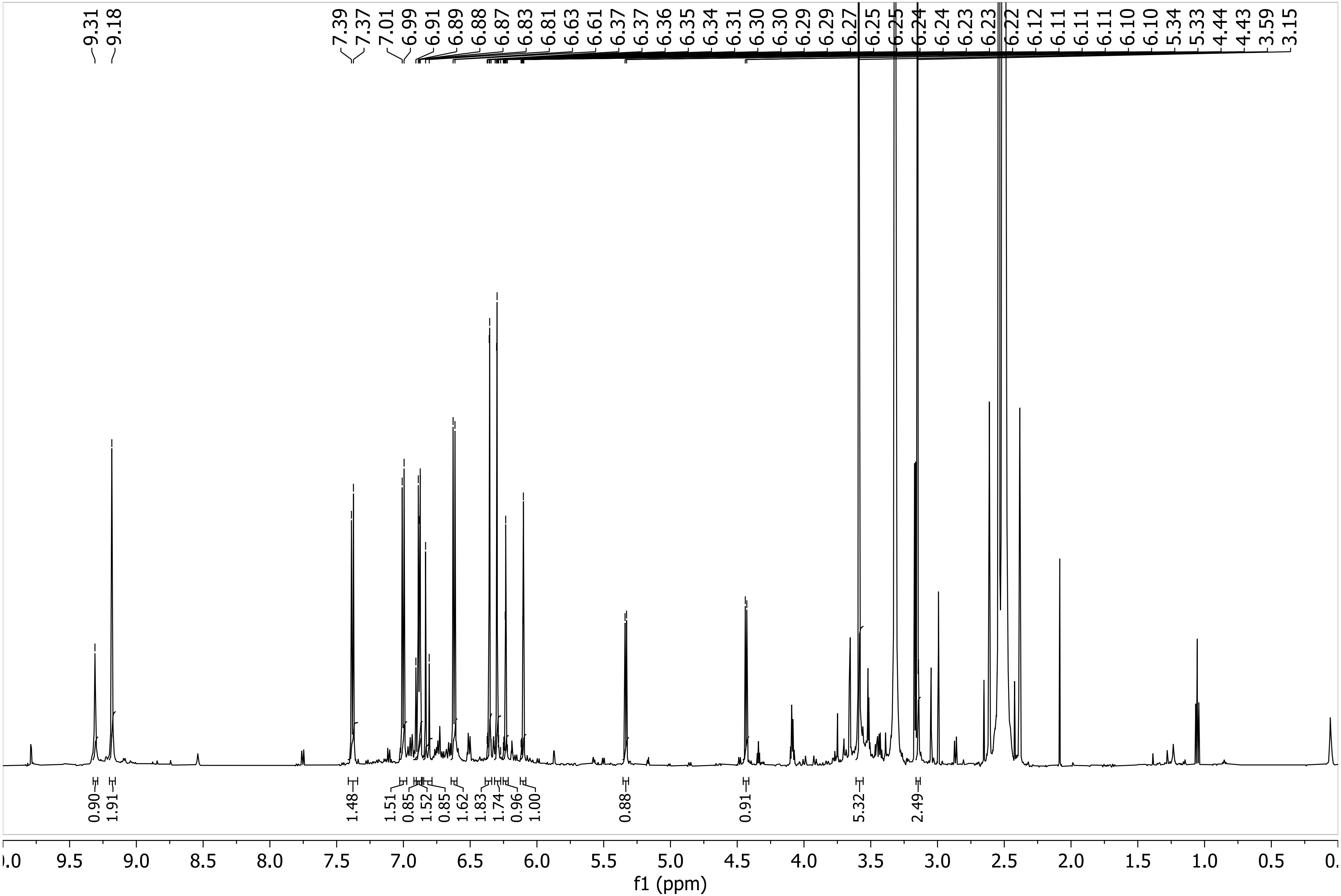

^{1}H NMR spectrum of compound **61** in DMSO-d_6

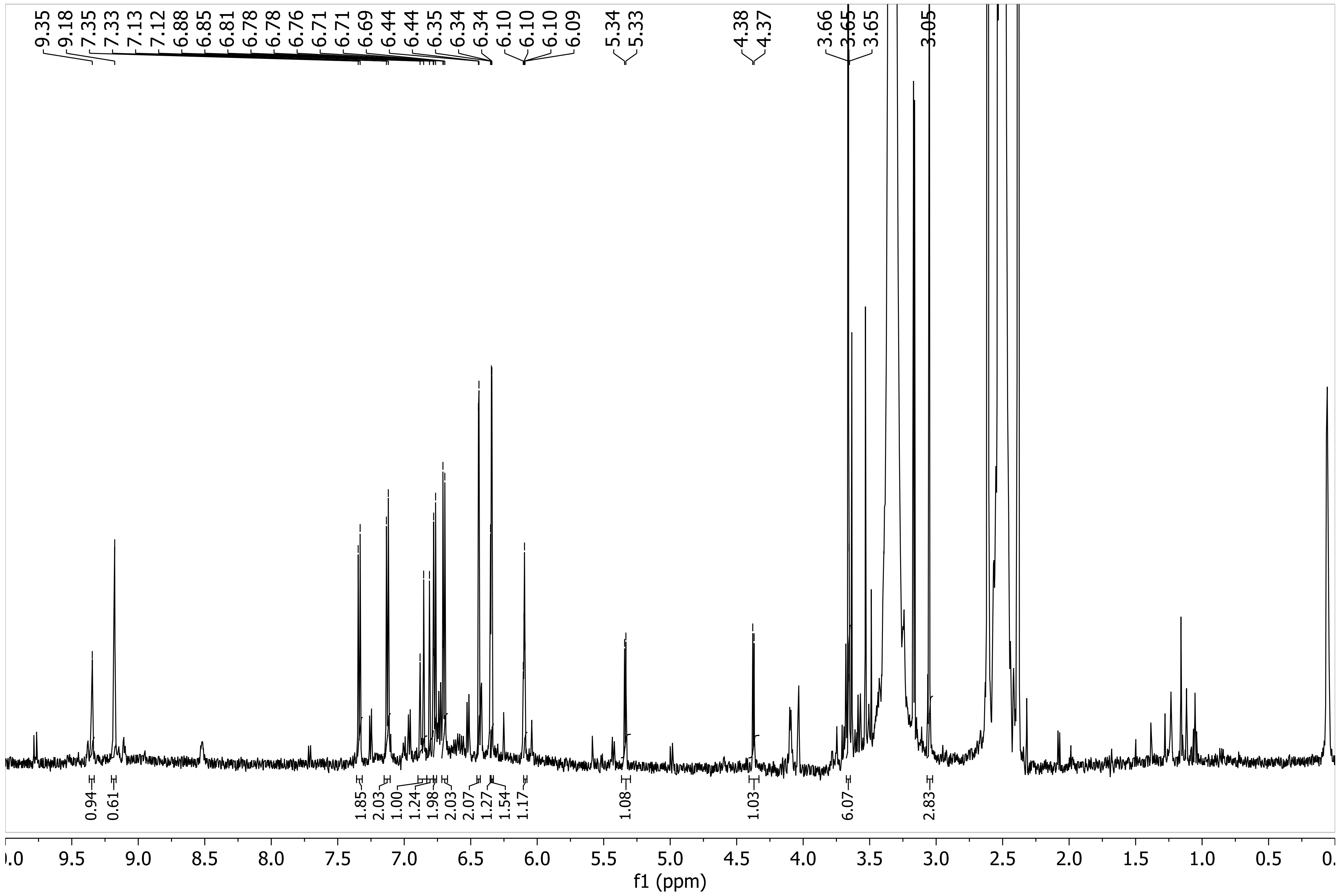

COSY NMR spectrum of compound **61** in DMSO-d_6

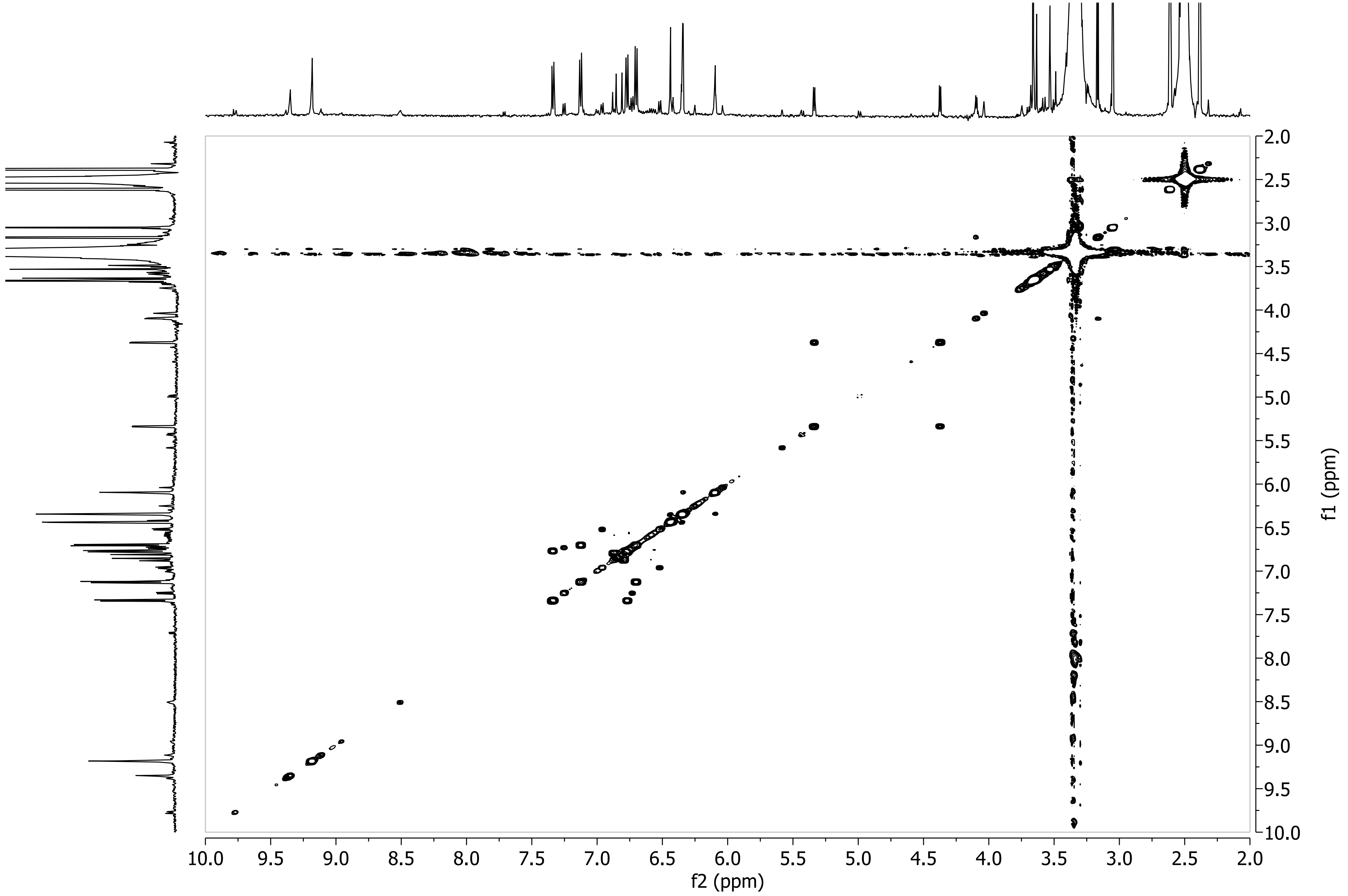

Edited-HSQC NMR spectrum of compound **61** in DMSO-d_6

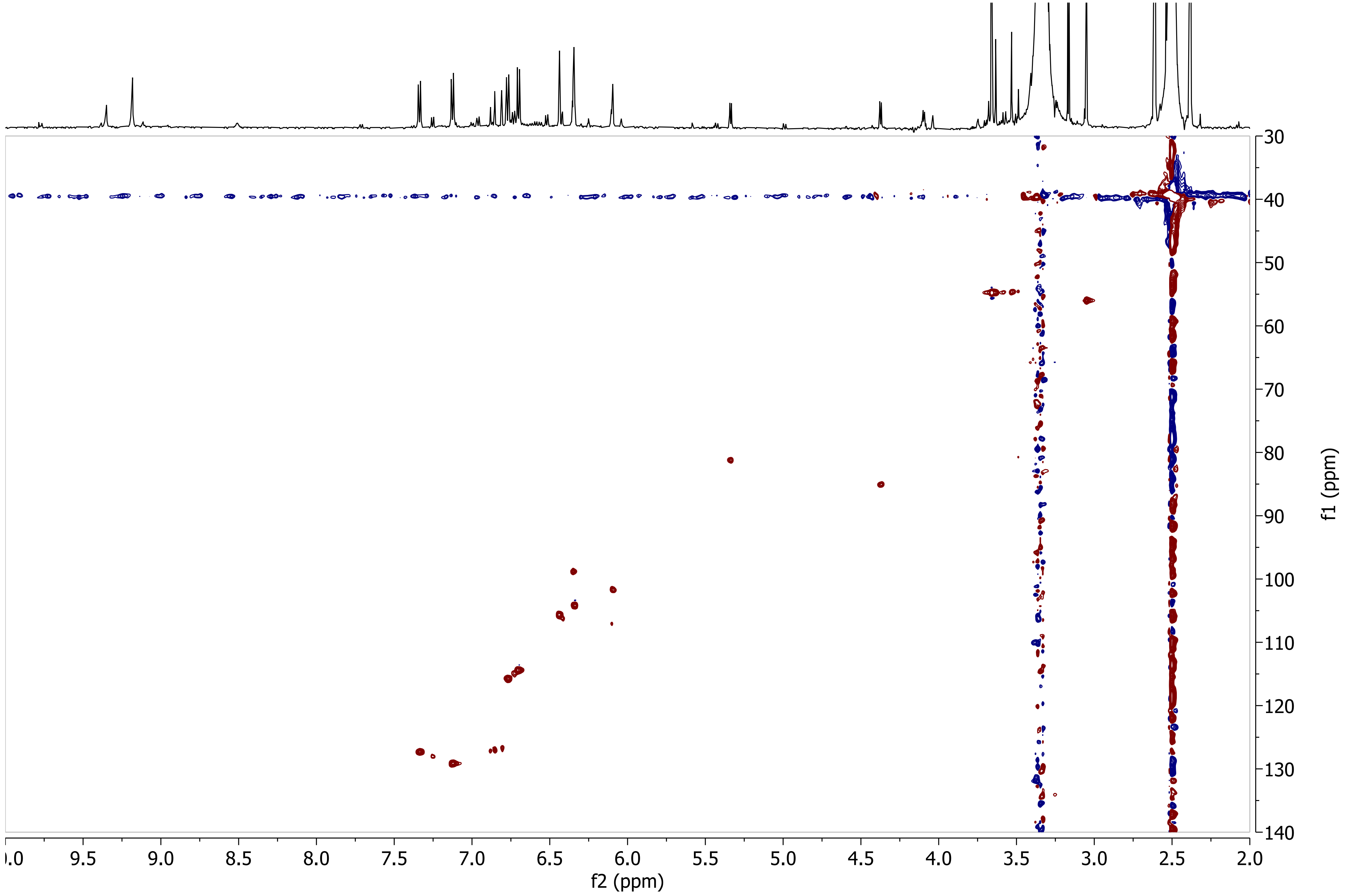

HMBC NMR spectrum of compound **61** in DMSO-d_6

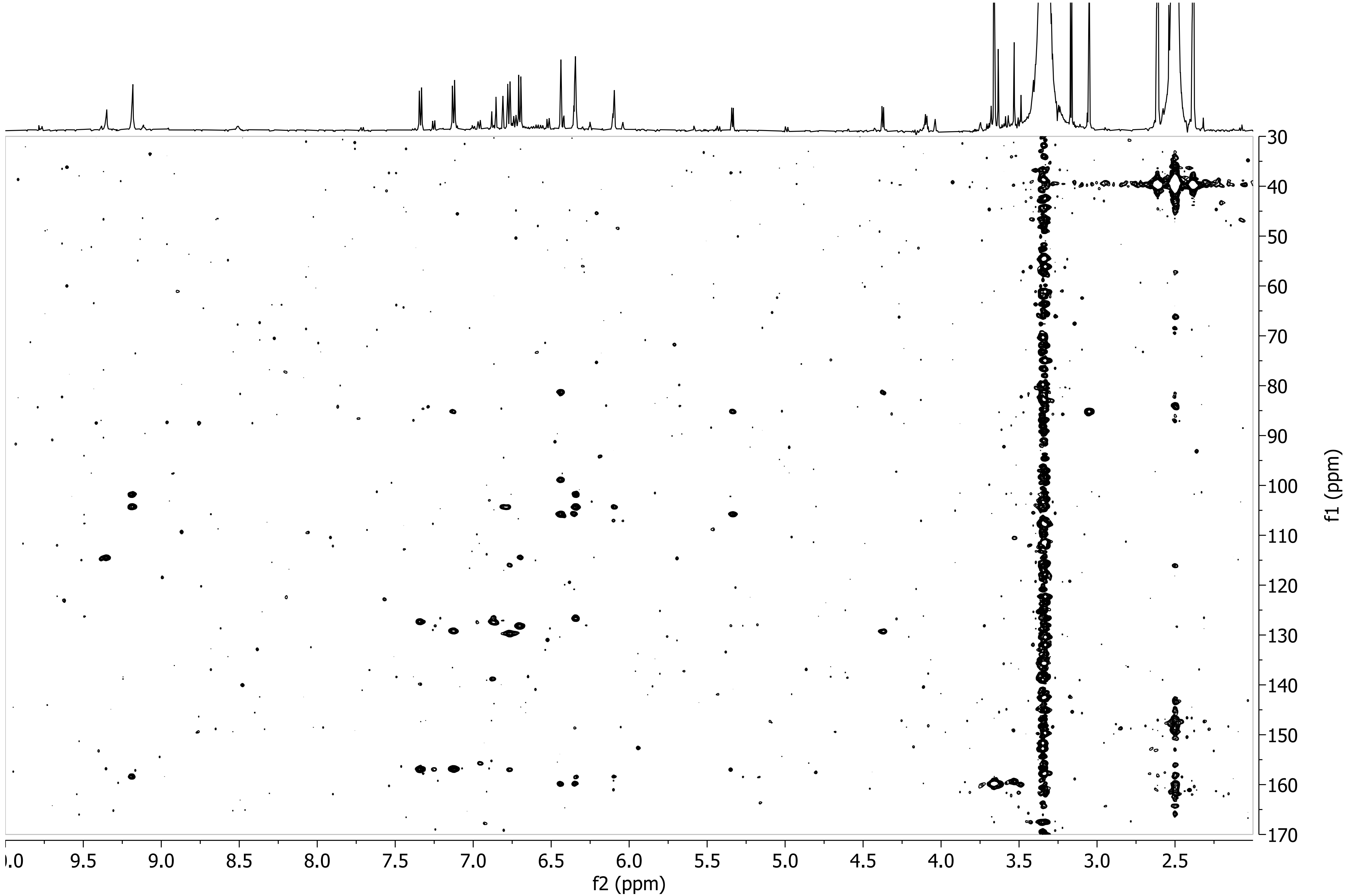

ROESY NMR spectrum of compound **61** in DMSO-d_6

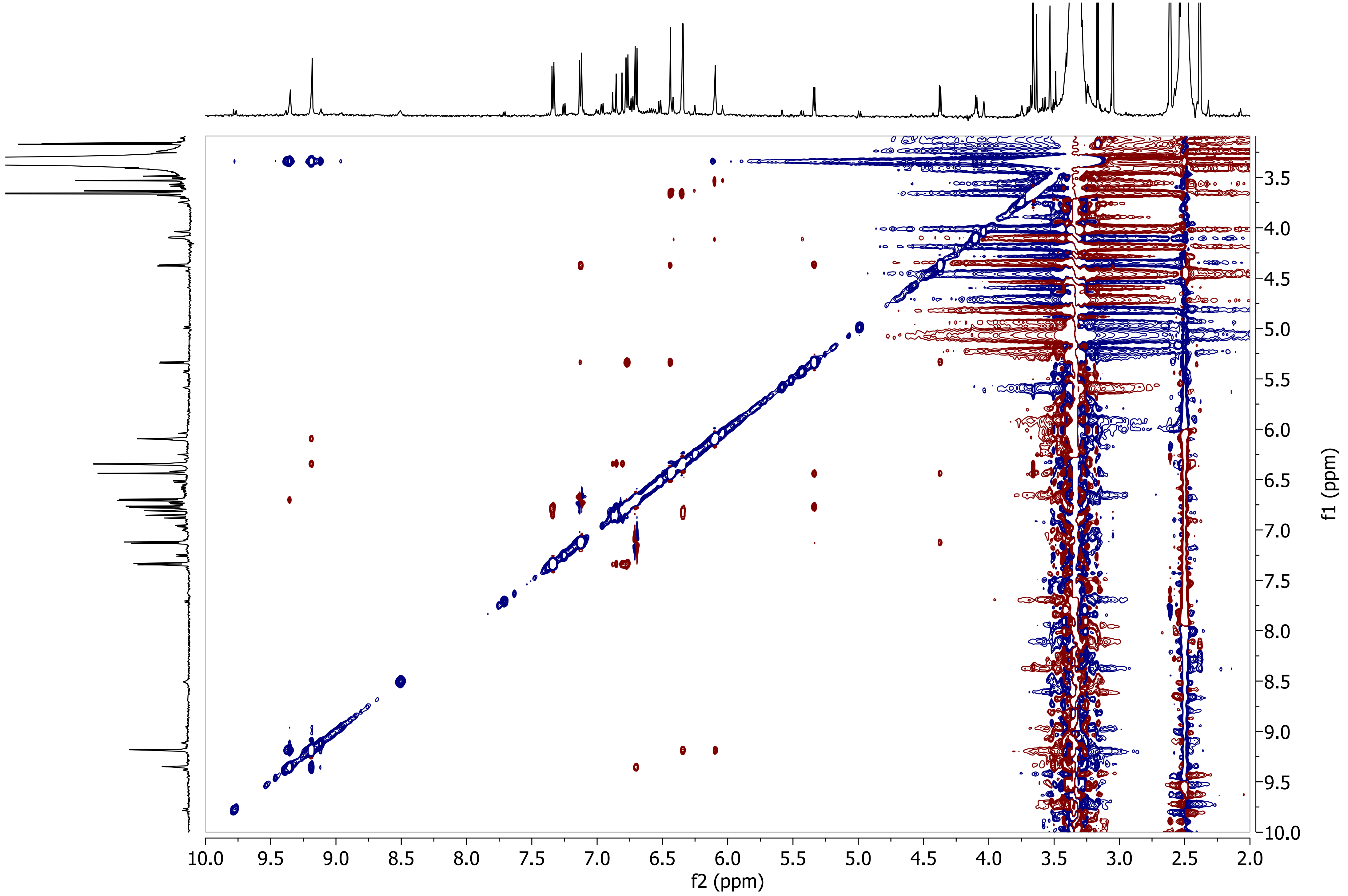

^{1}H NMR spectrum of compound **62** in DMSO-d_6

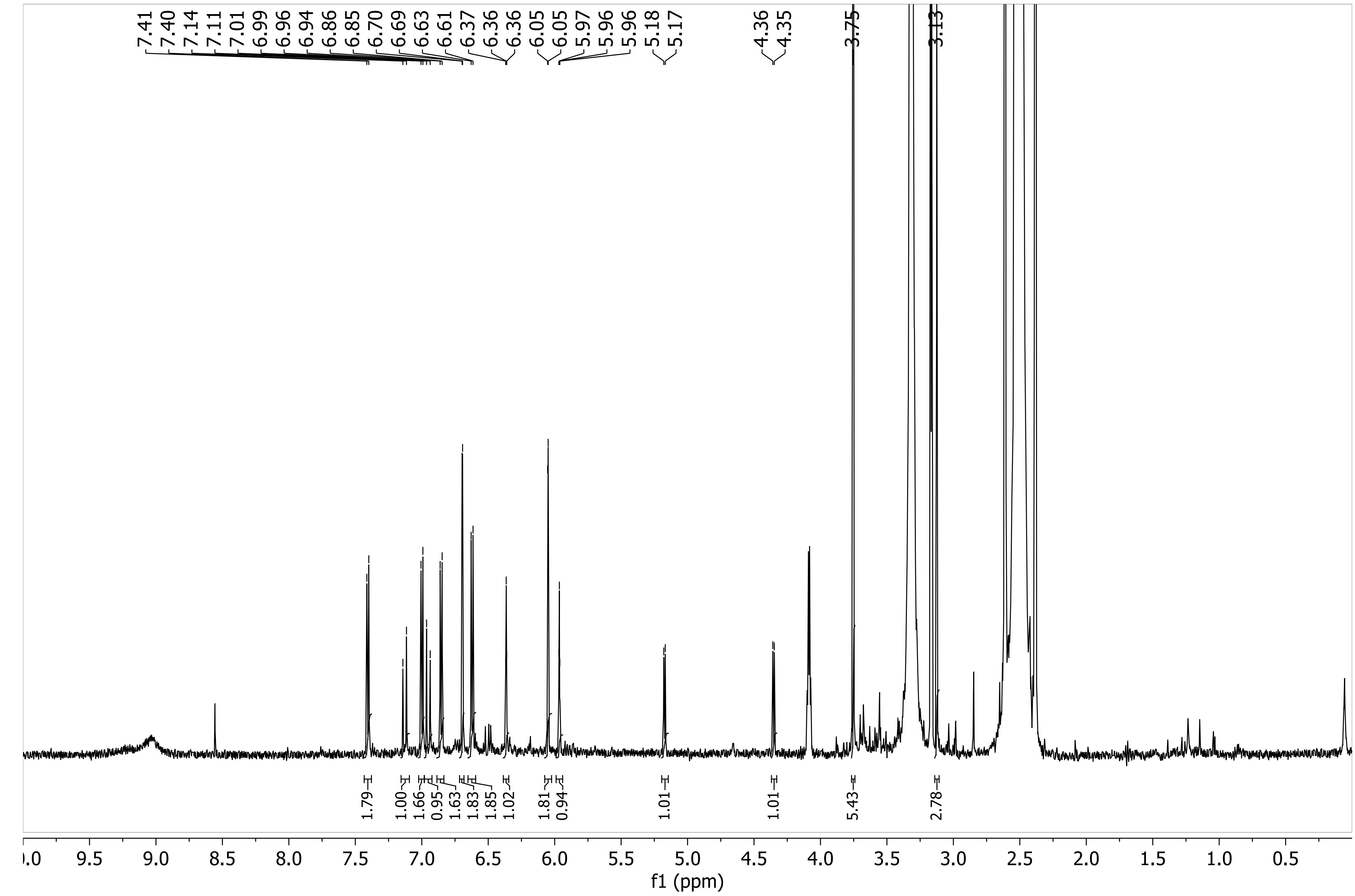

^{1}H NMR spectrum of compound **63** in CD_3OD

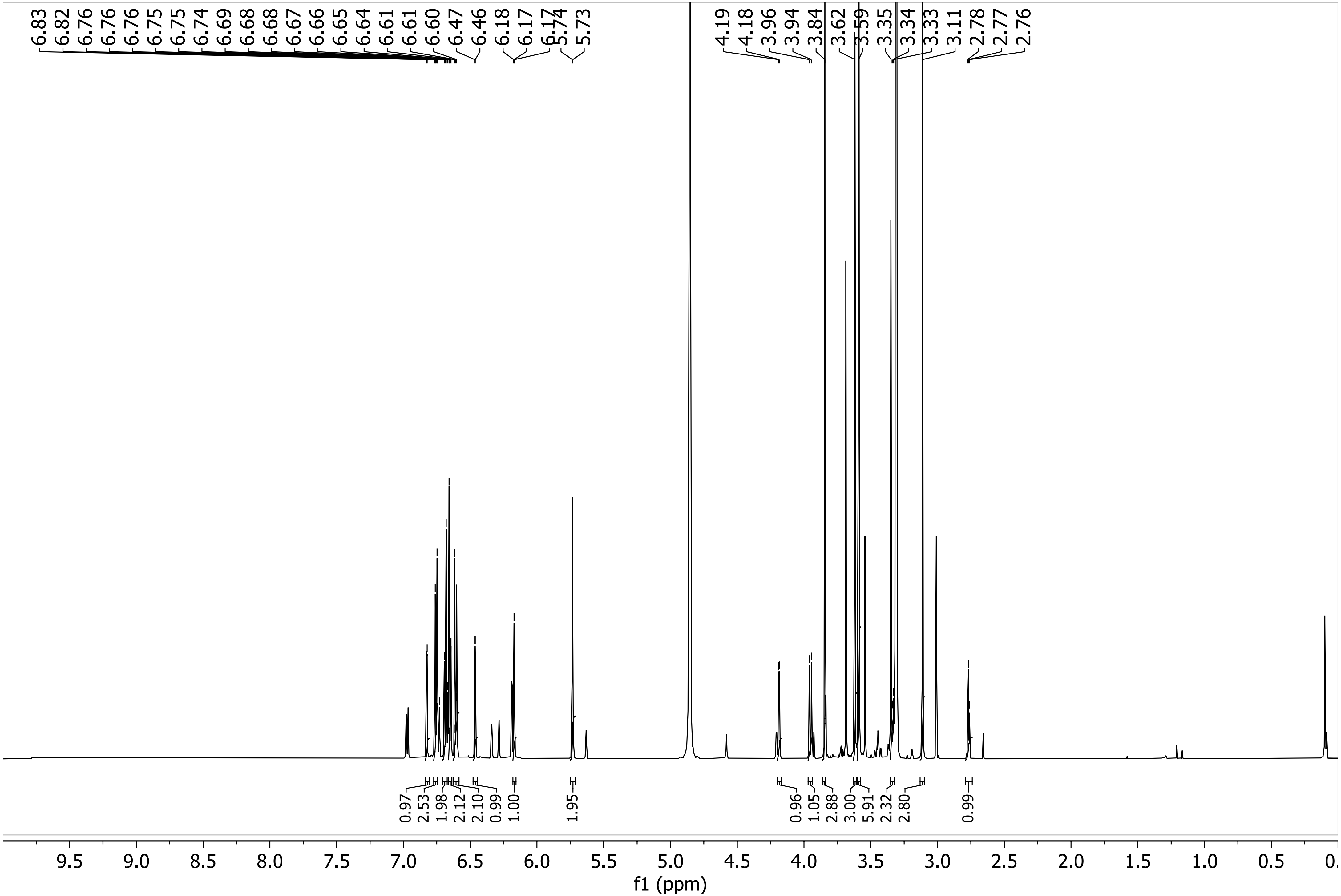

^{1}H NMR spectrum of compound **64** in DMSO-d_6

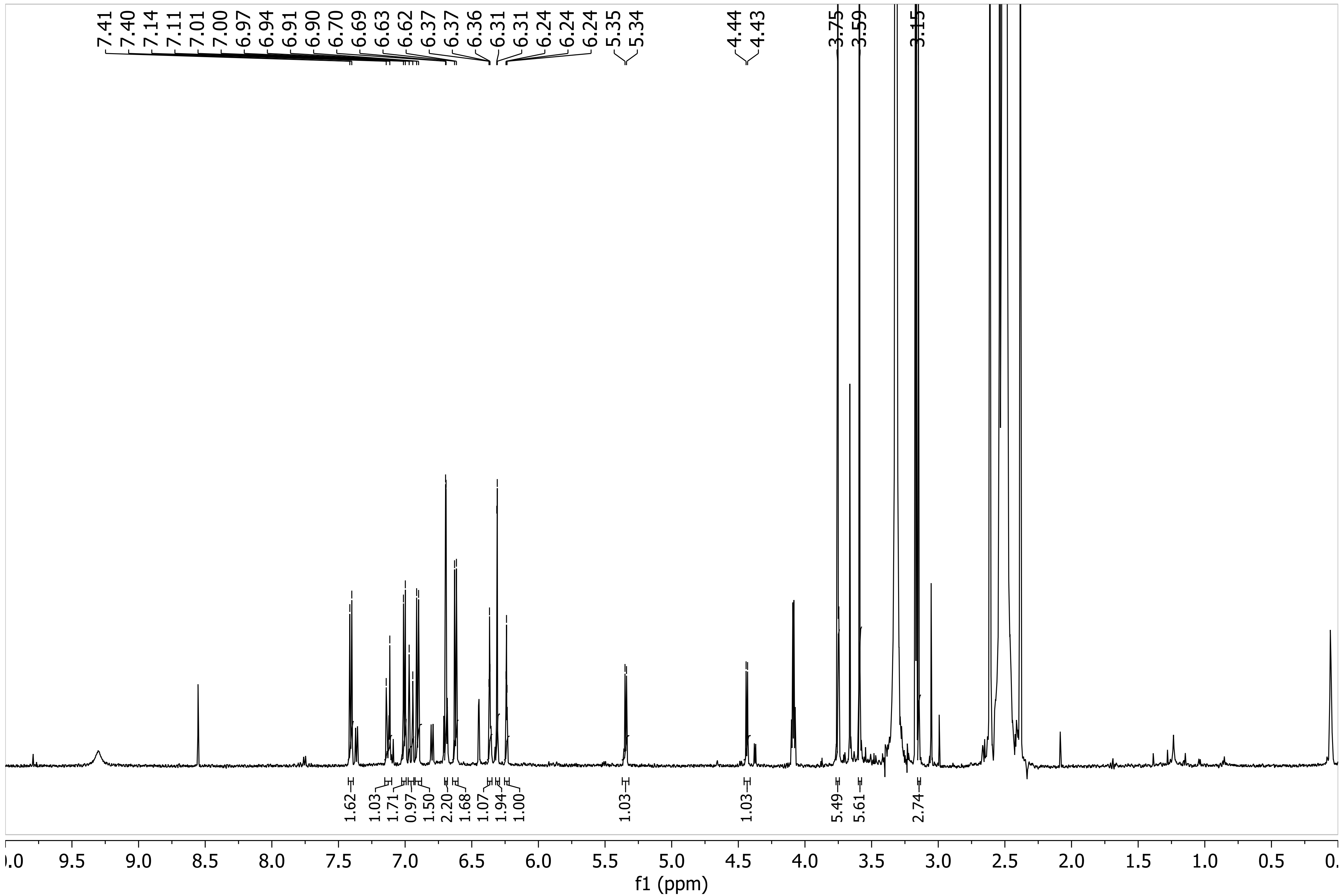

COSY NMR spectrum of compound **64** in DMSO-d_6

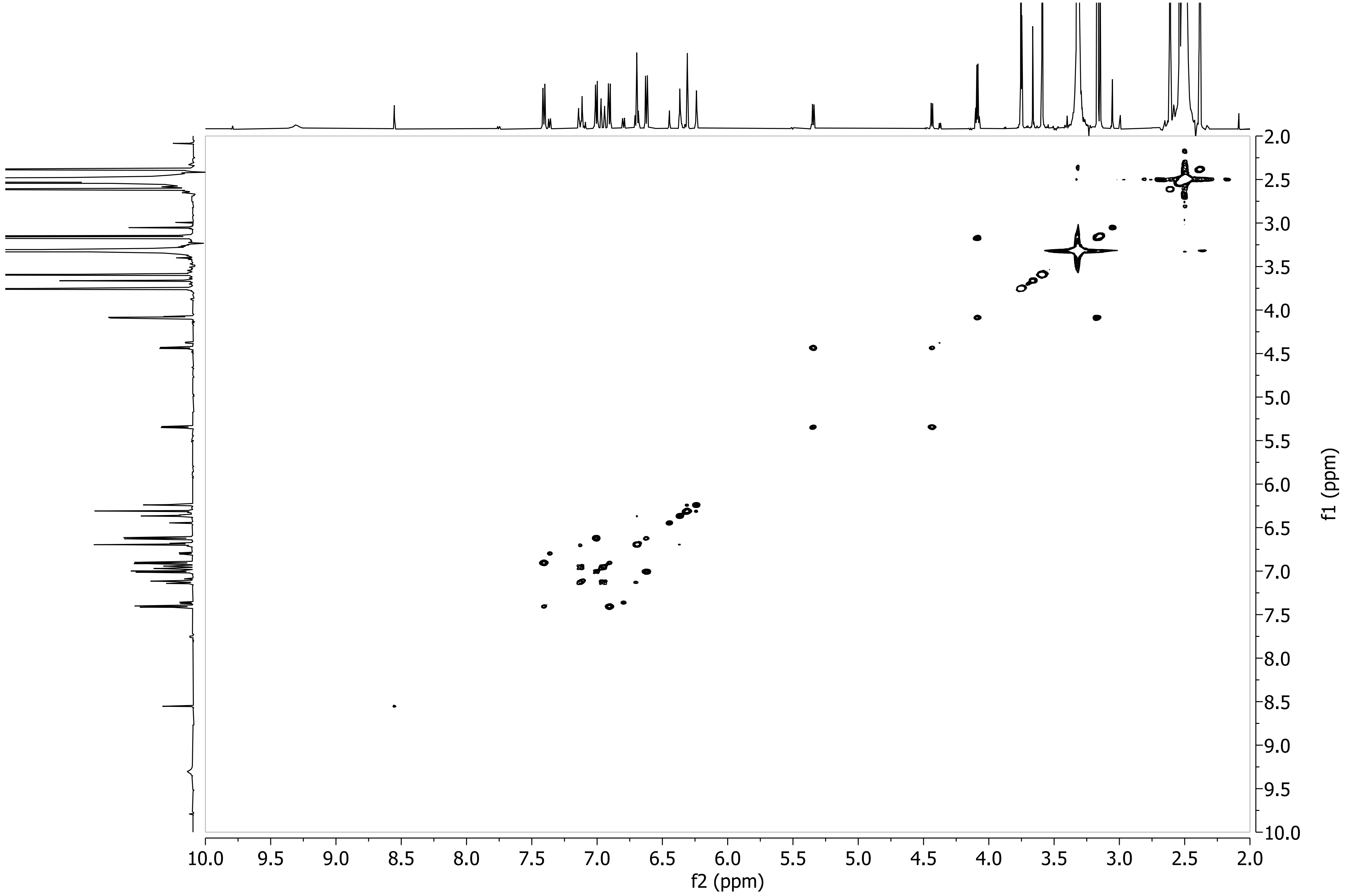

Edited-HSQC NMR spectrum of compound **64** in DMSO-d_6

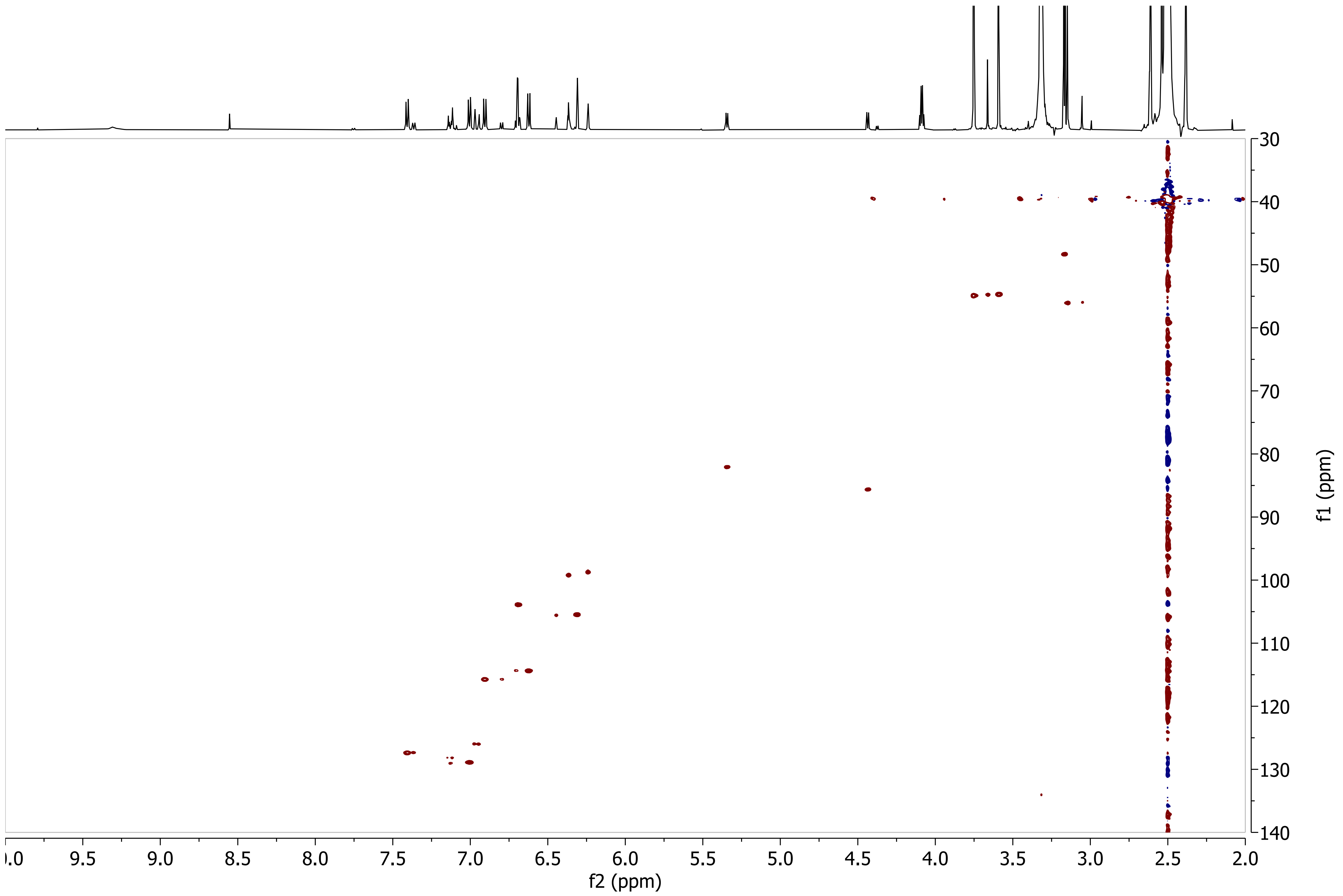

HMBC NMR spectrum of compound **64** in DMSO-d_6

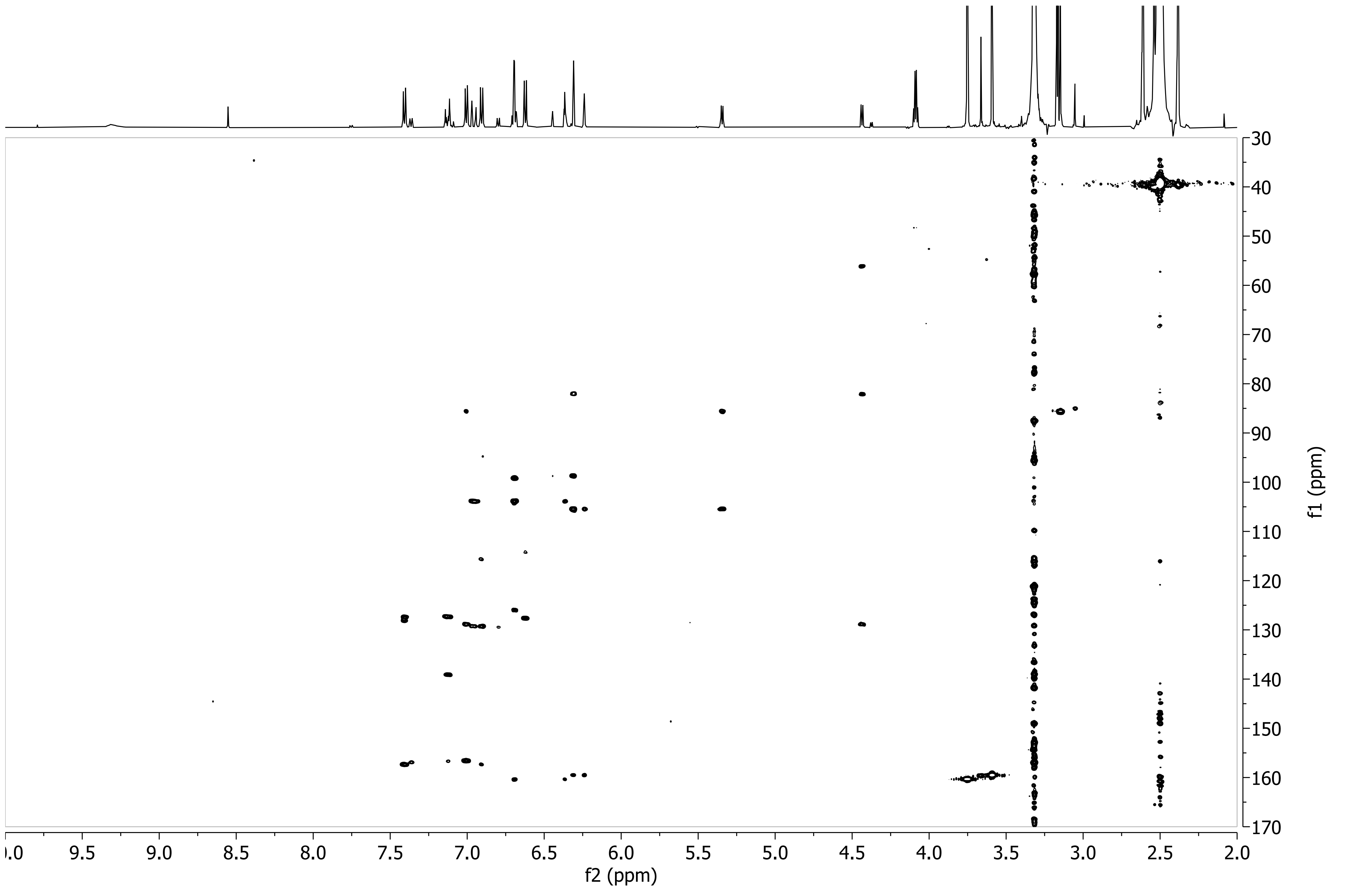

^{1}H NMR spectrum of compound **65** in DMSO-d_6

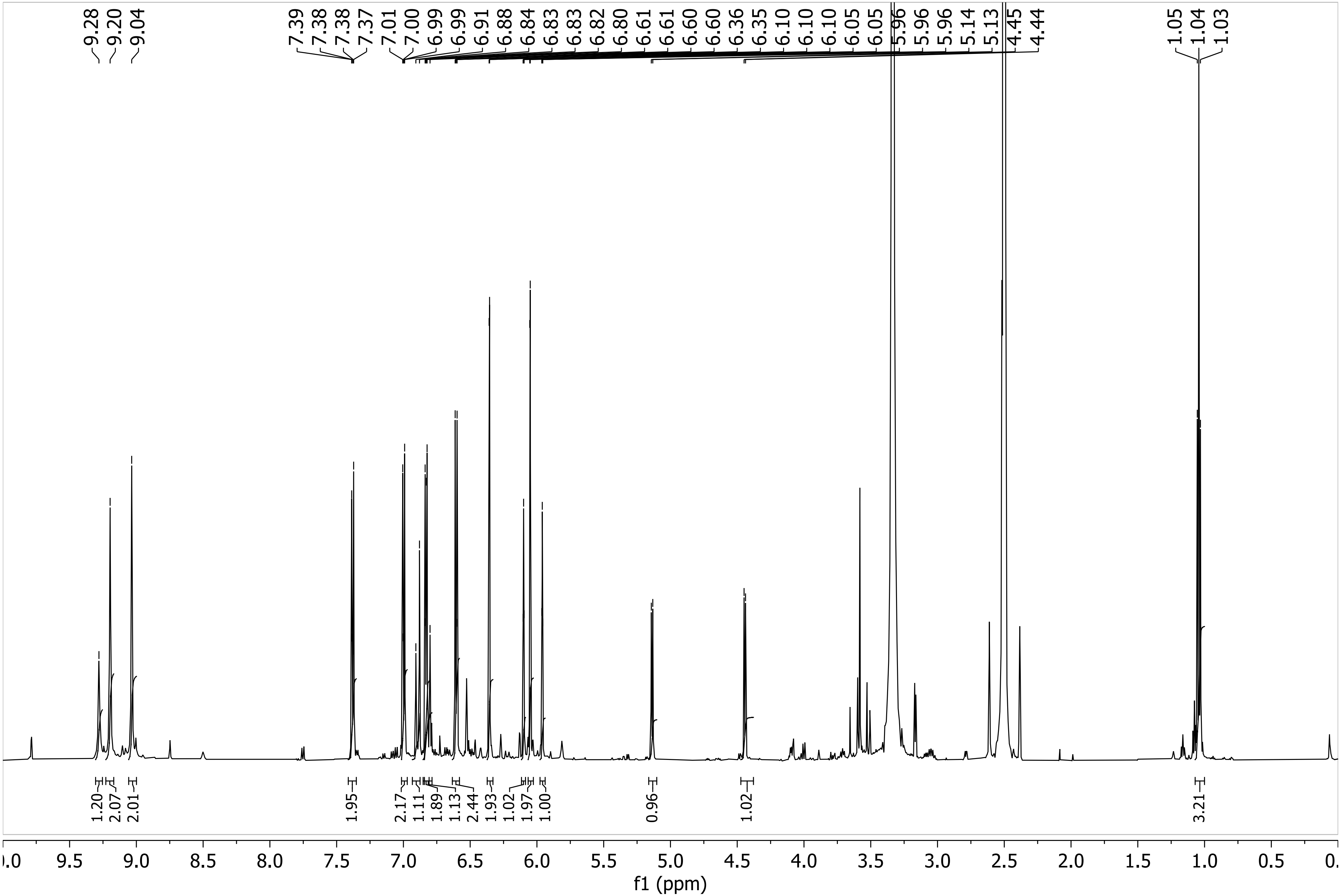

COSY NMR spectrum of compound **65** in DMSO-d_6

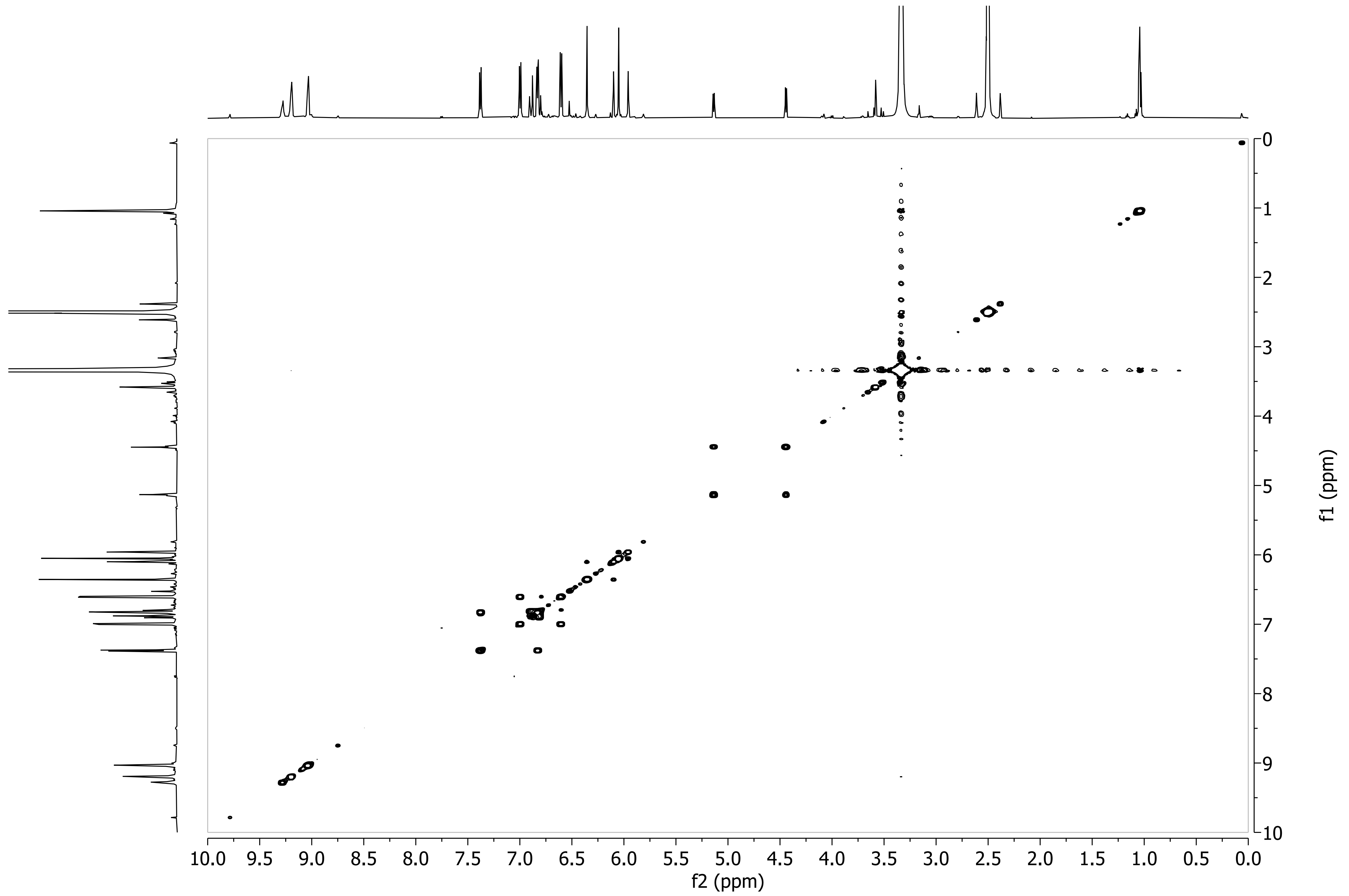

^{13}C-DEPTQ NMR spectrum of compound **65** in DMSO-d_6

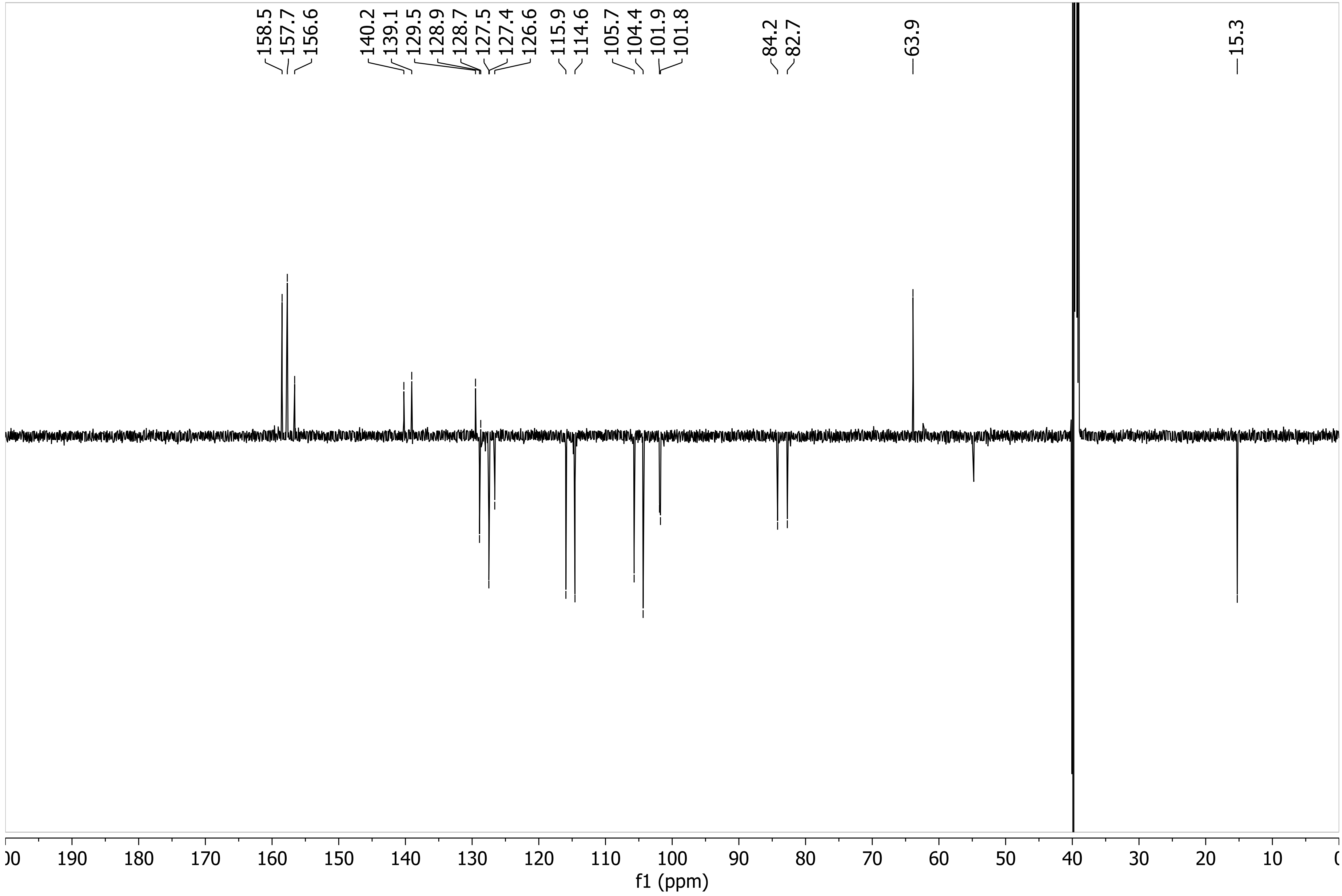

Edited-HSC NMR spectrum of compound **65** in DMSO-d_6

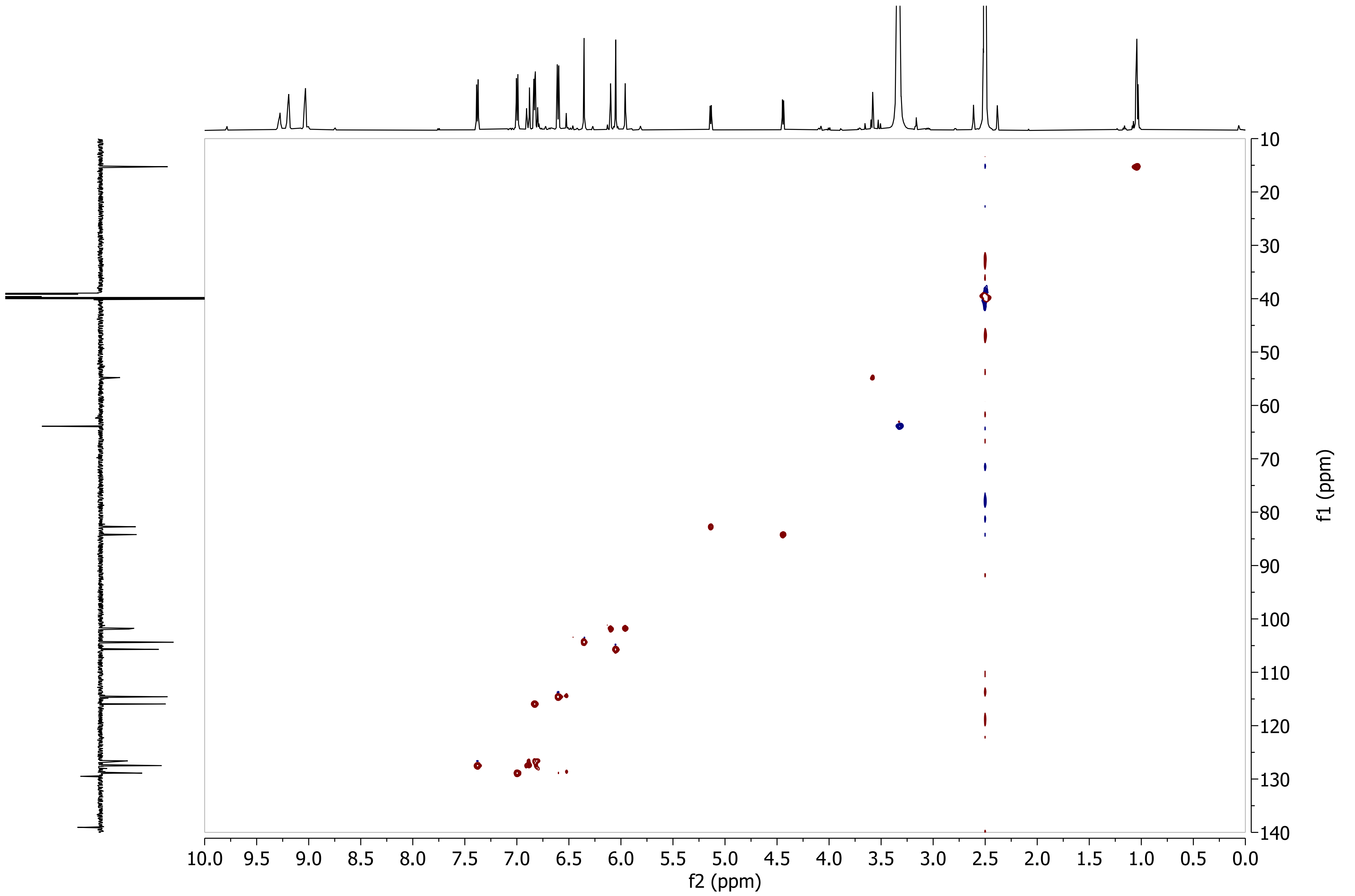

HMBC NMR spectrum of compound **65** in DMSO-d_6

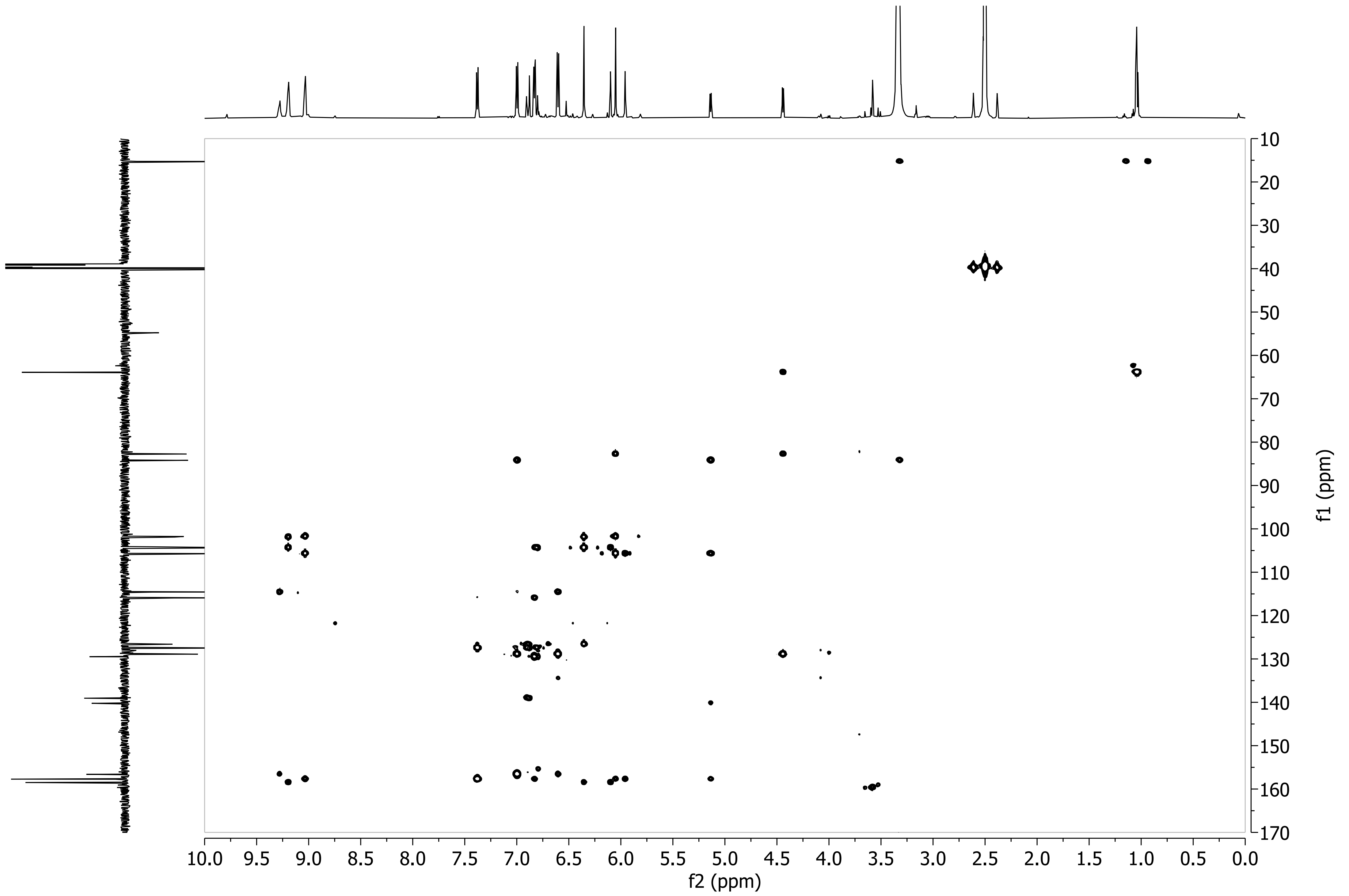

ROESY NMR spectrum of compound **65** in DMSO-d_6

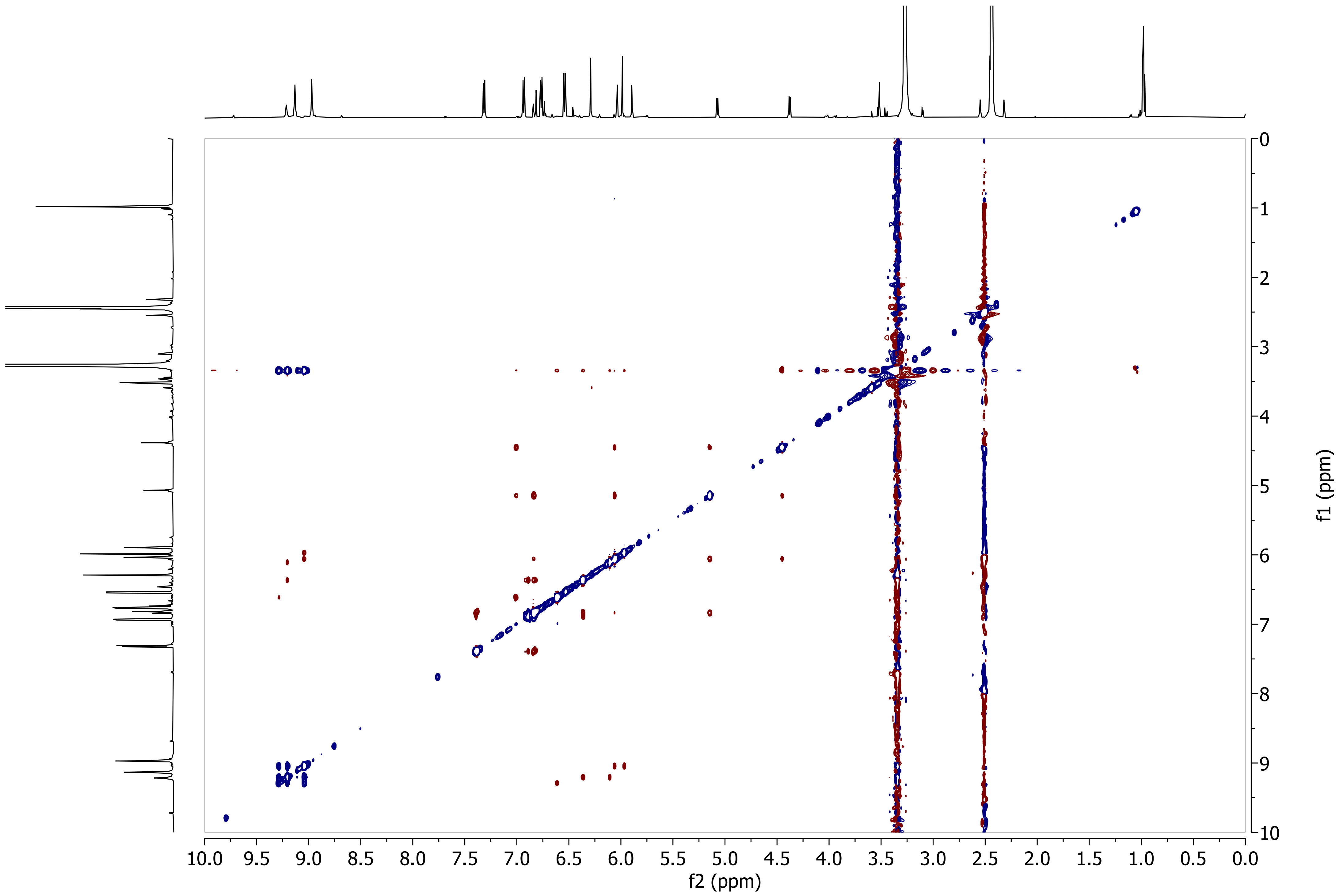

^{1}H NMR spectrum of compound **66** in DMSO-d_6

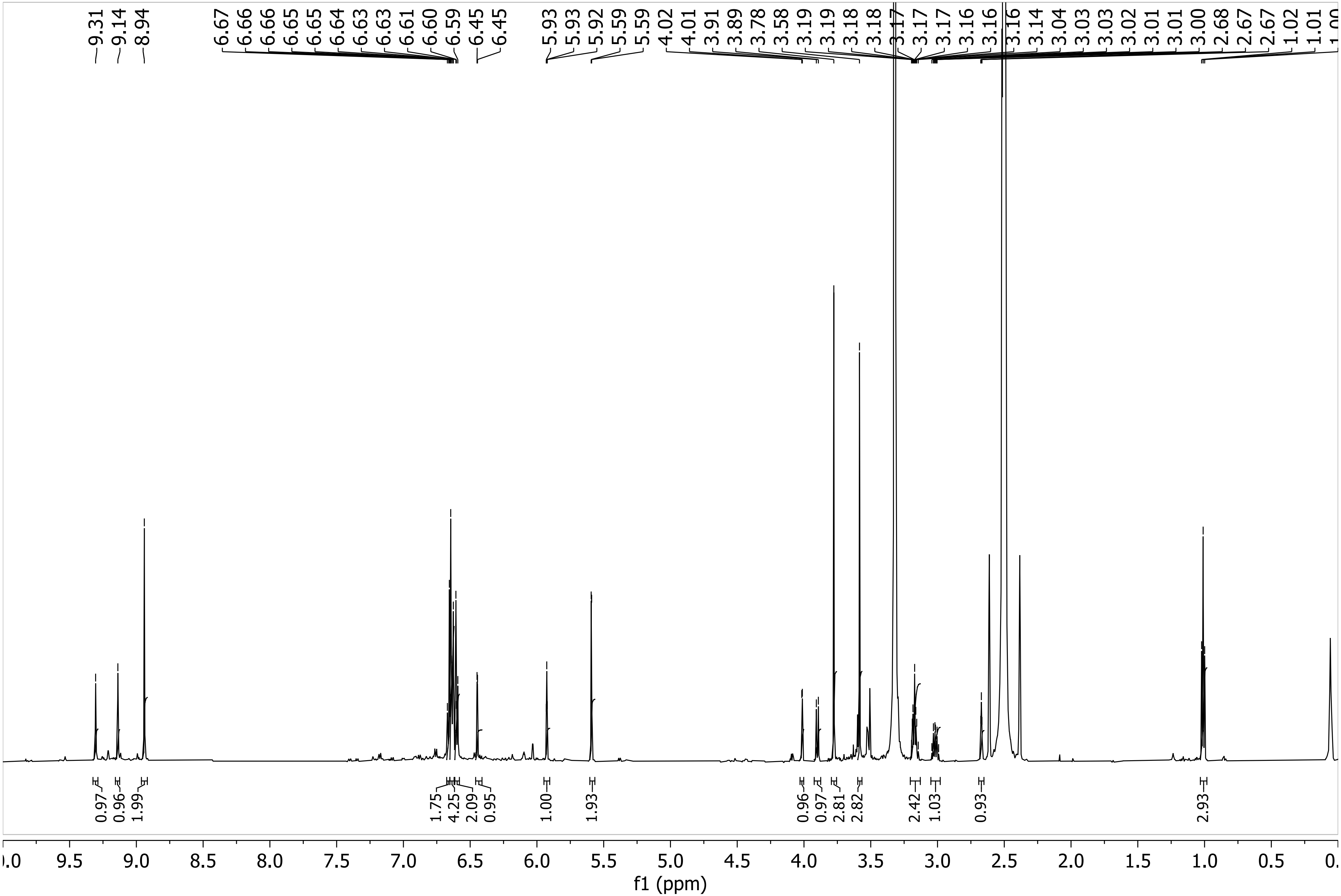

COSY NMR spectrum of compound **66** in DMSO-d_6

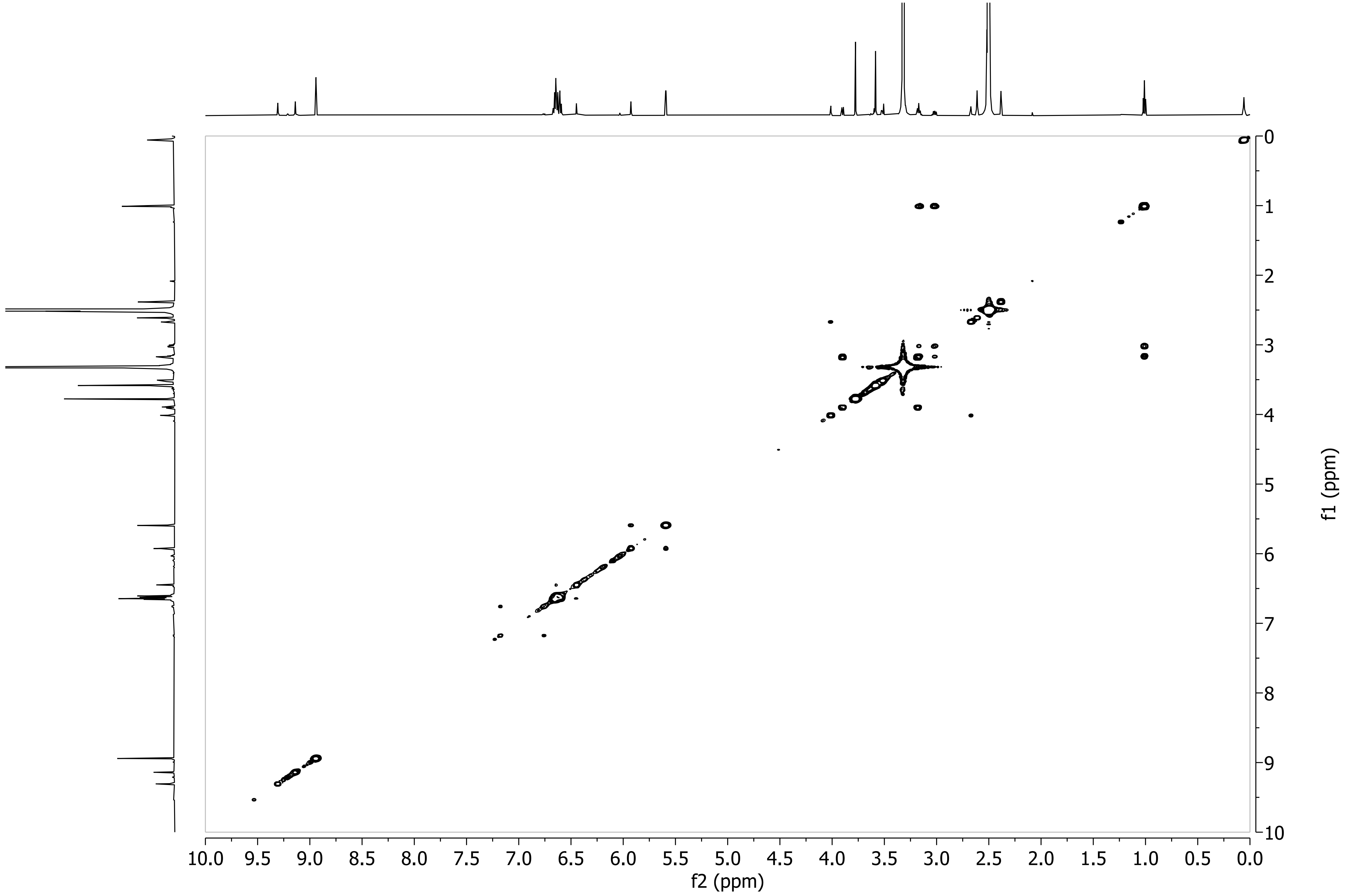

^{13}C-DEPTQ NMR spectrum of compound **66** in DMSO-d_6

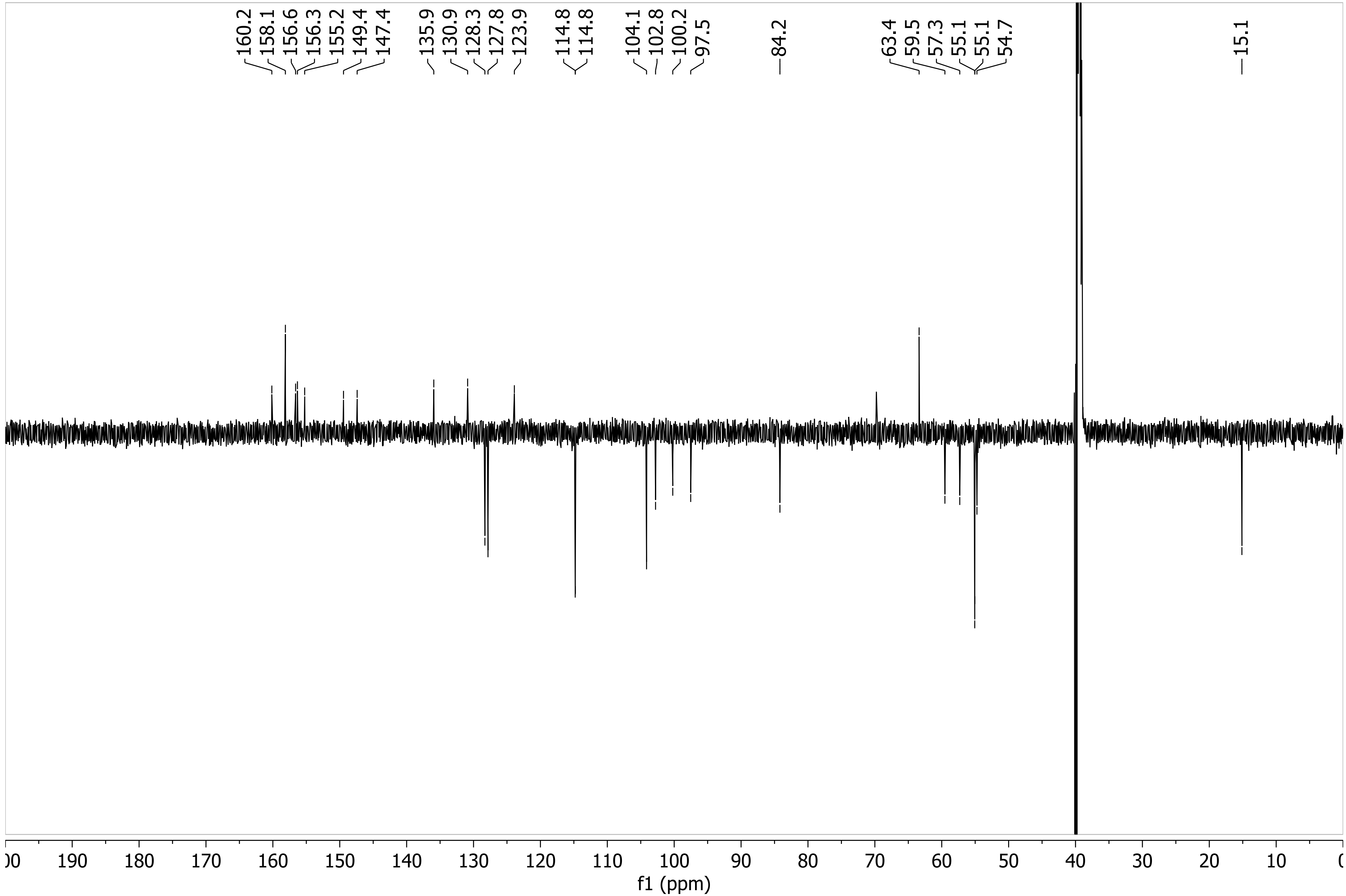

Edited-HSQC NMR spectrum of compound **66** in DMSO-d_6

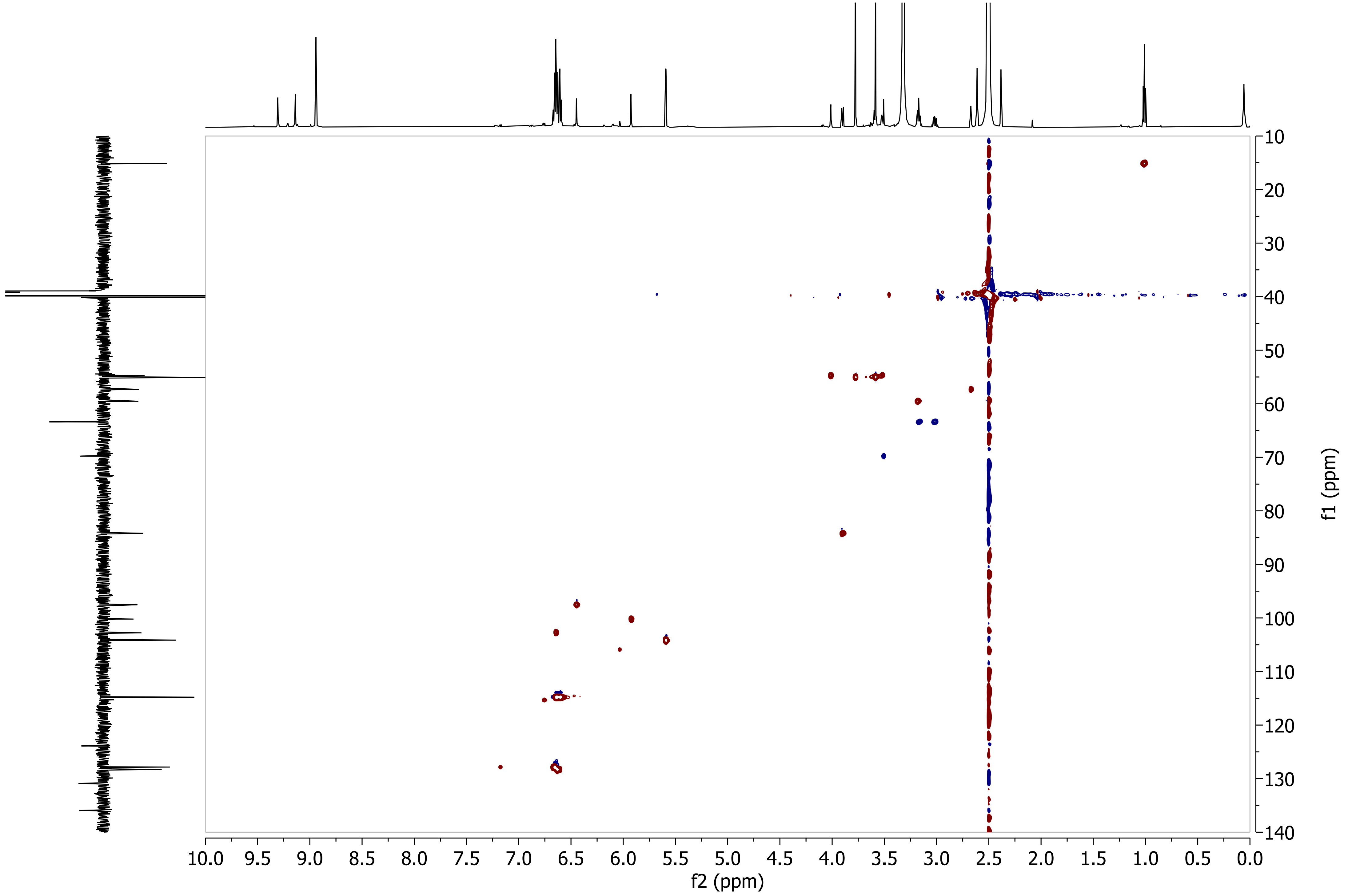

HMBC NMR spectrum of compound **66** in DMSO-d_6

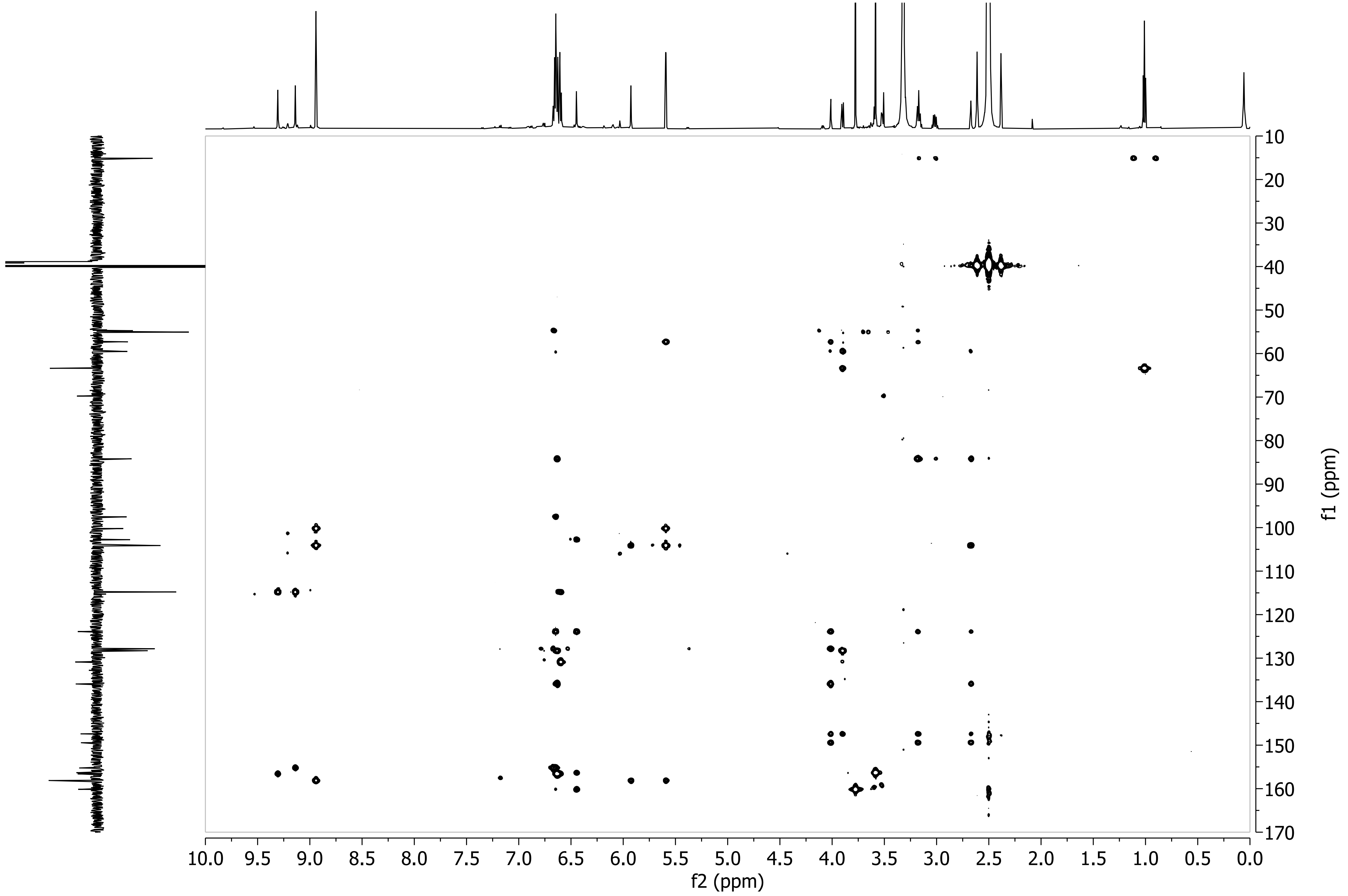

ROESY NMR spectrum of compound **66** in DMSO-d_6

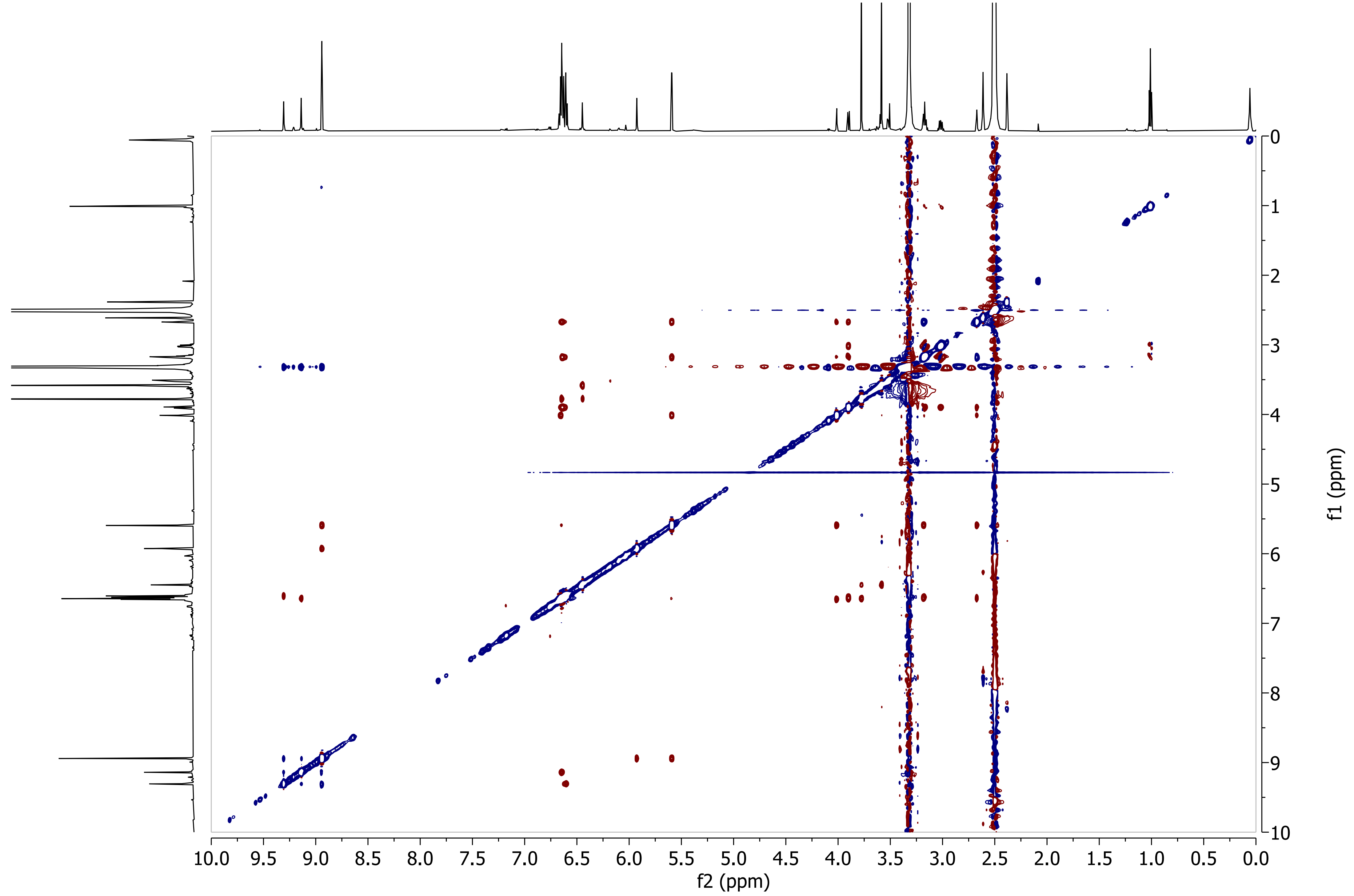

^{1}H NMR spectrum of compound **67** in DMSO-d_6

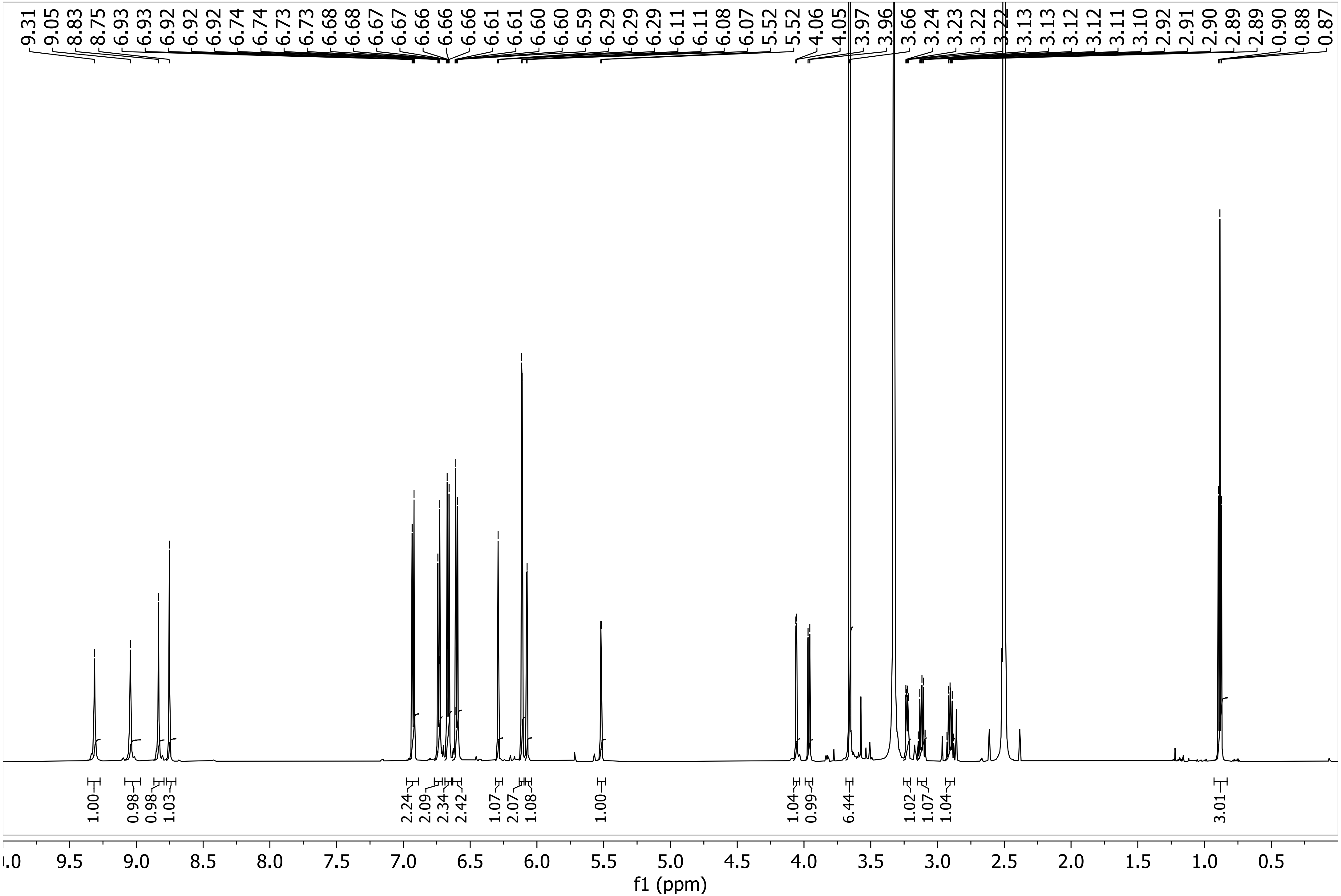

COSY NMR spectrum of compound **67** in DMSO-d_6

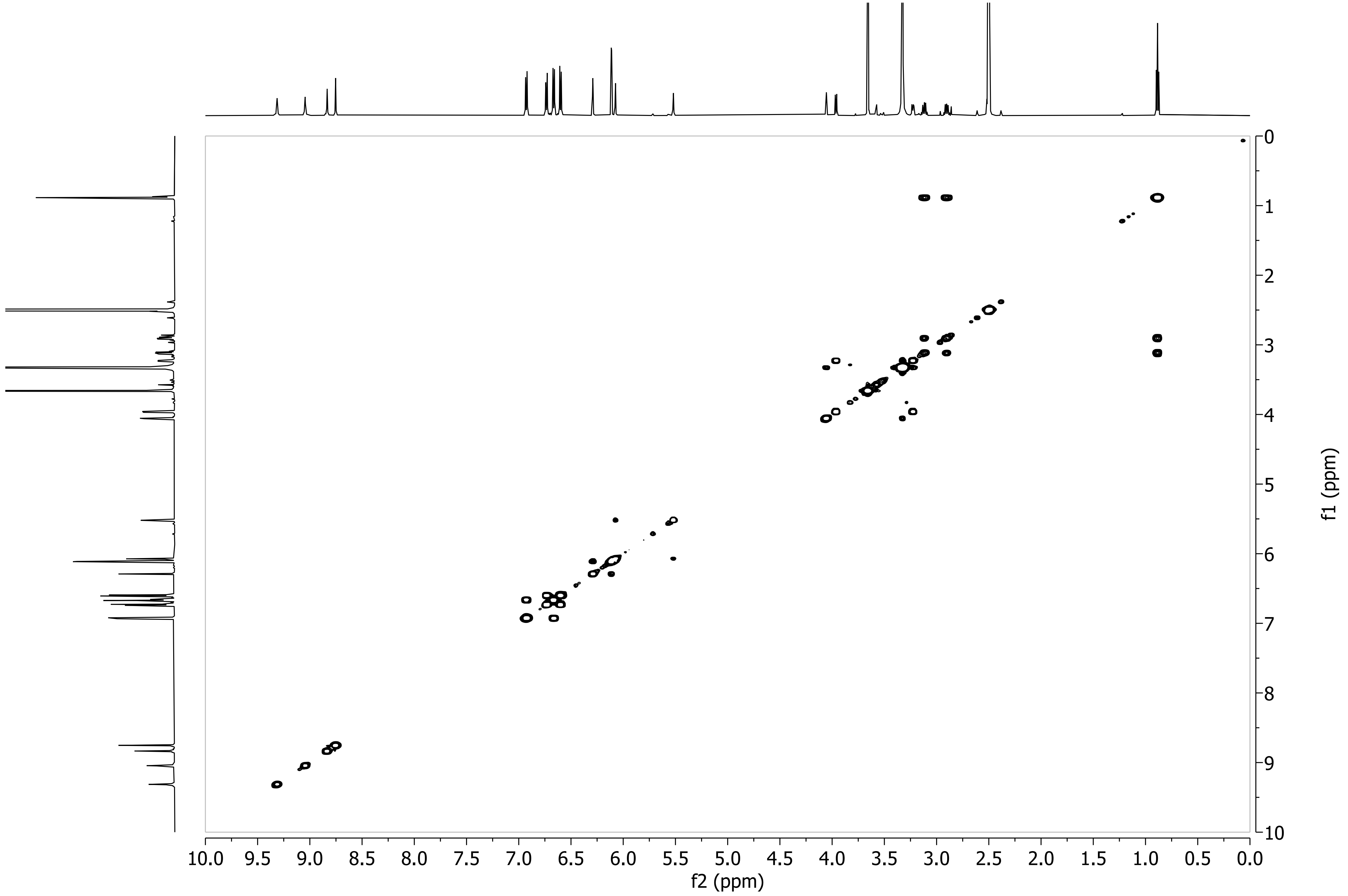

^{13}C-DEPTQ NMR spectrum of compound **67** in DMSO-d_6

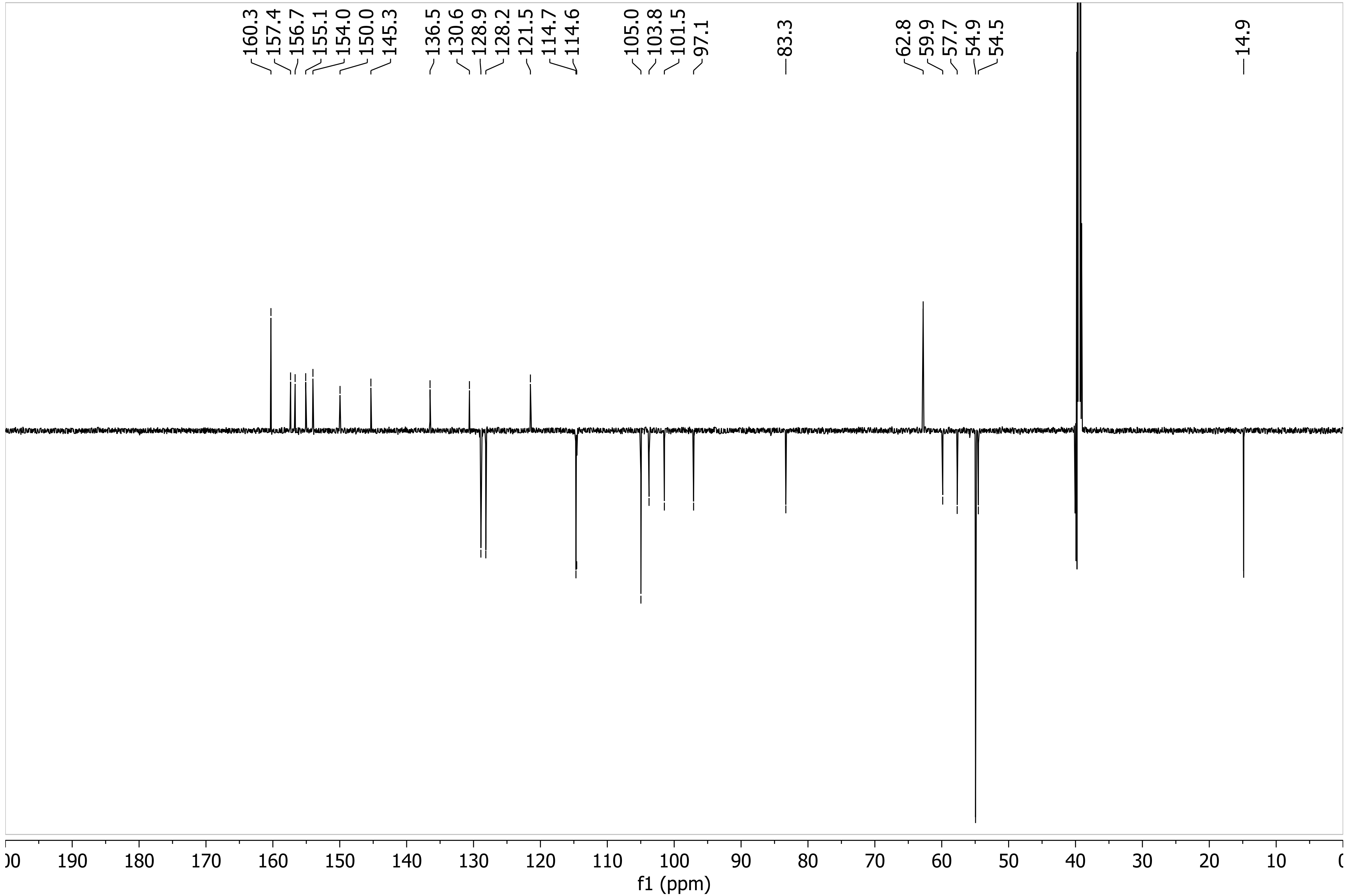

Edited-HSQC NMR spectrum of compound **67** in DMSO-d_6

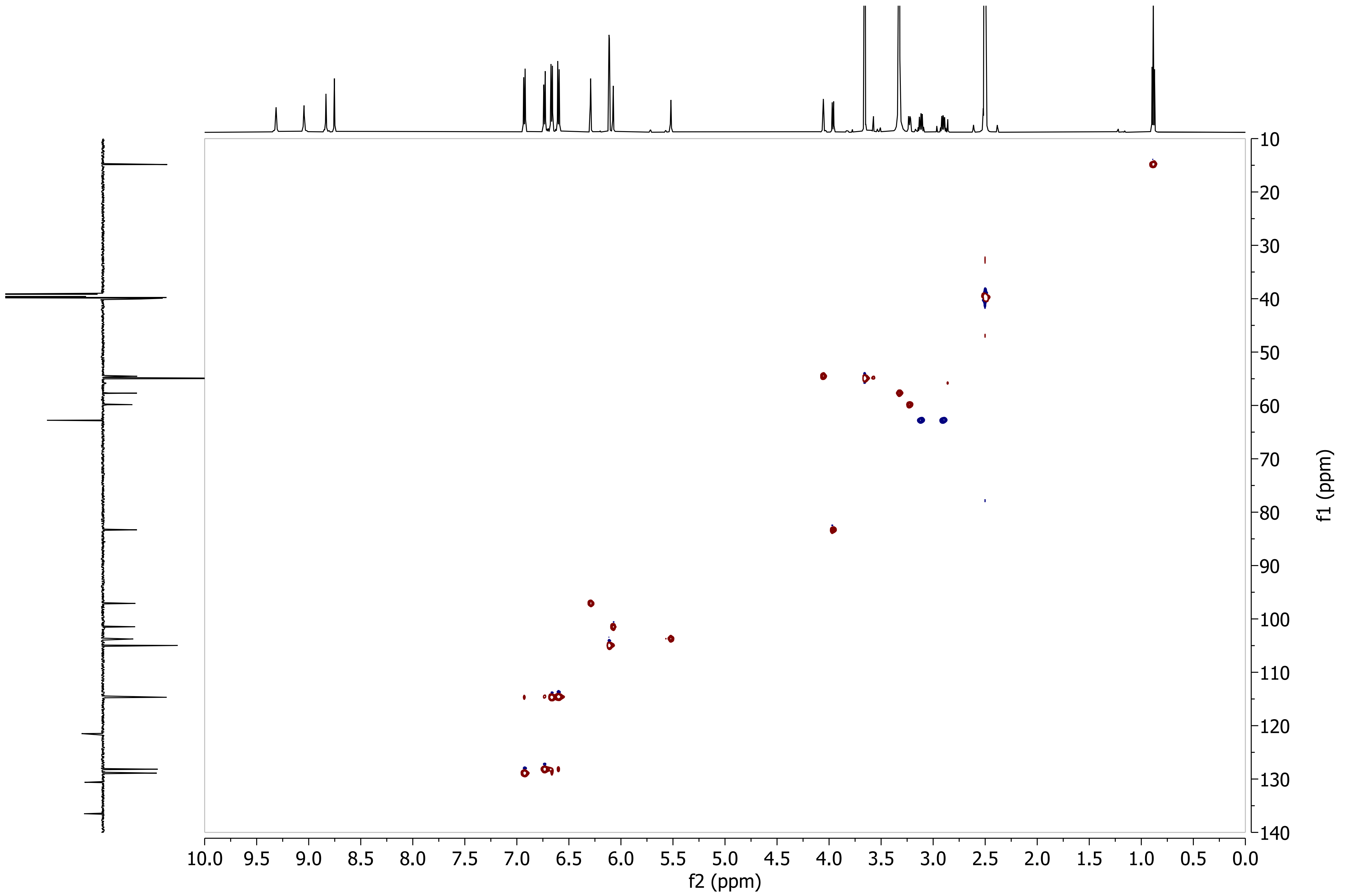

HMBC NMR spectrum of compound **67** in DMSO-d_6

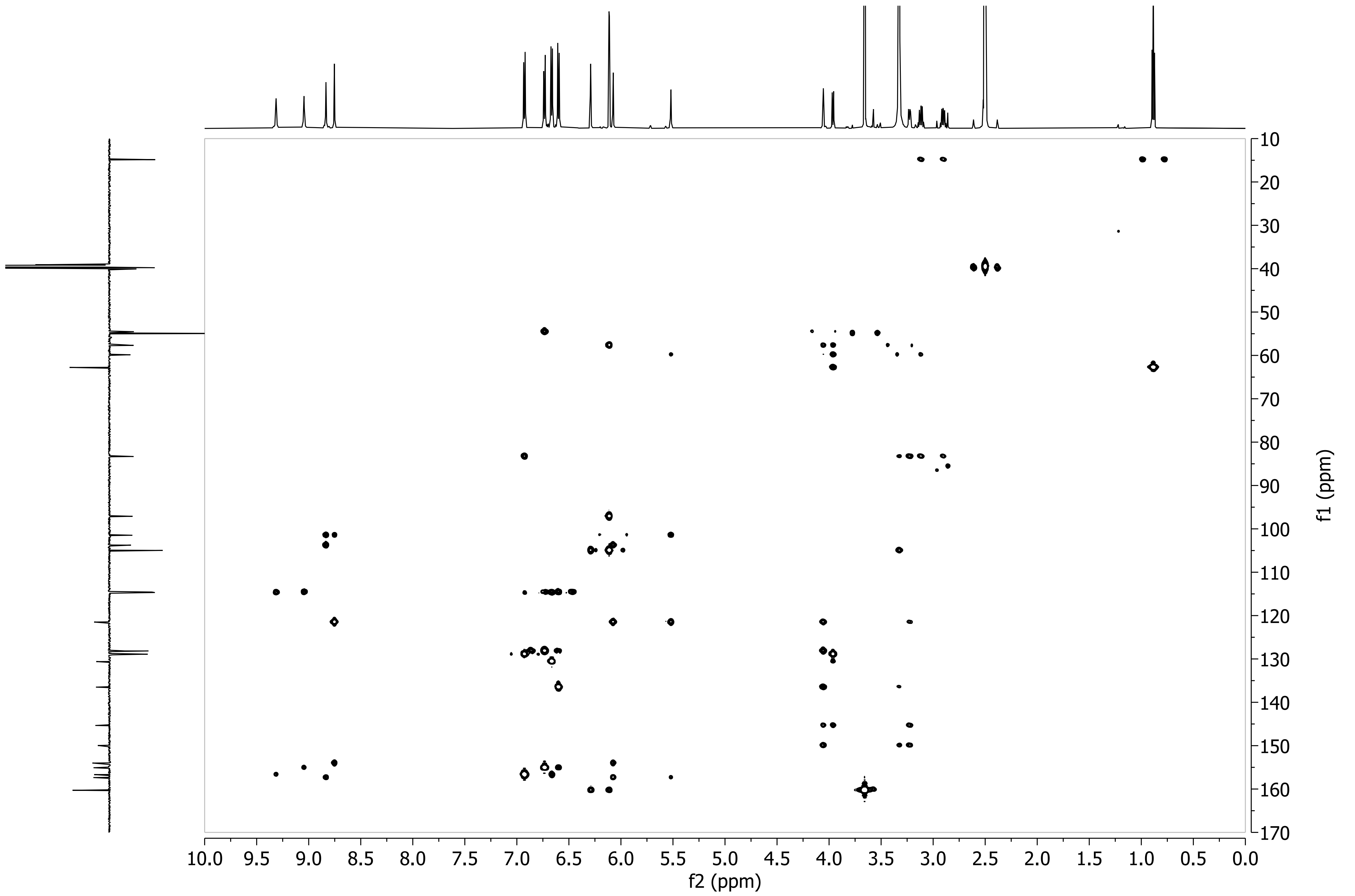

ROESY NMR spectrum of compound **67** in DMSO-d_6

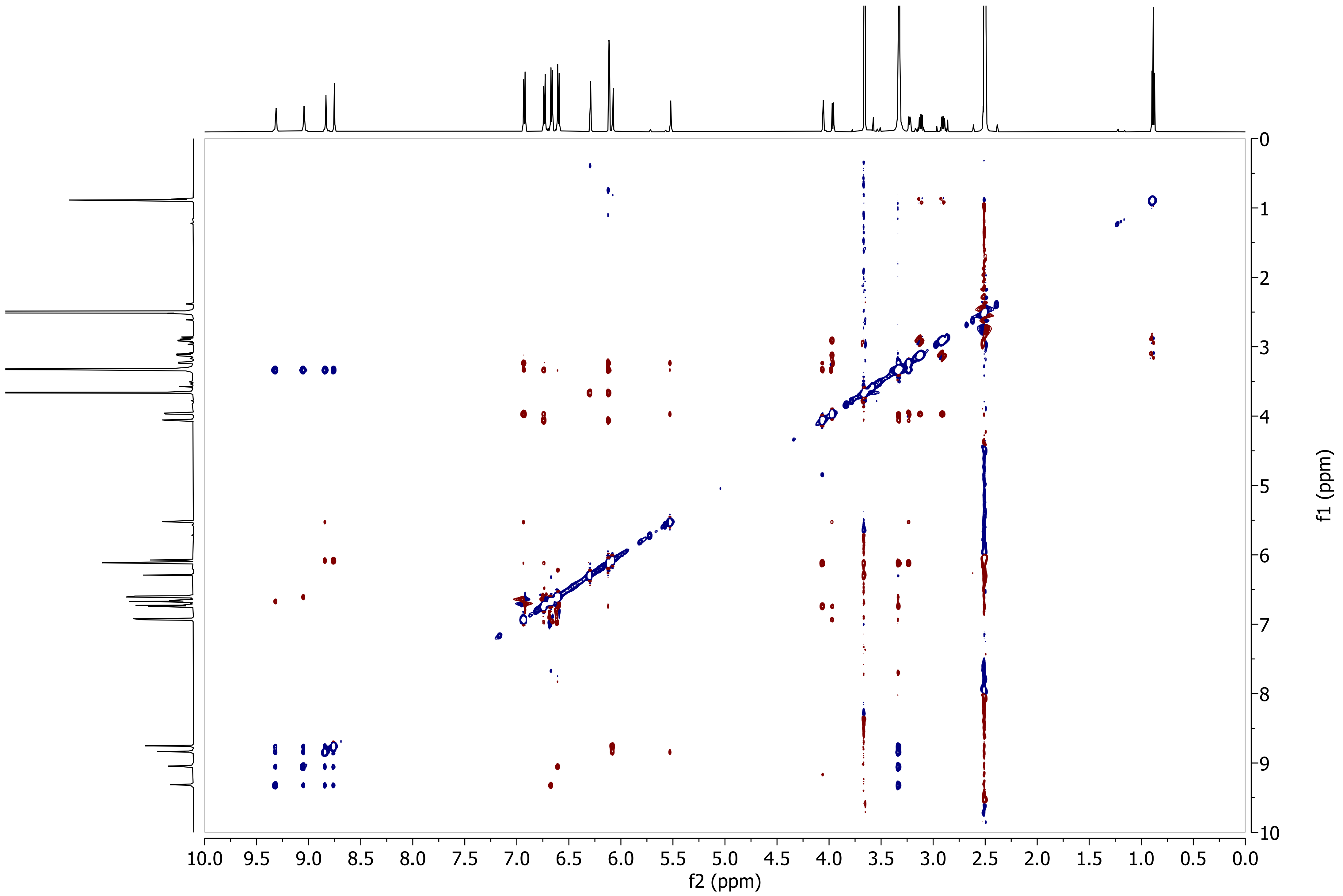

^{1}H NMR spectrum of compound **68** in DMSO-d_6

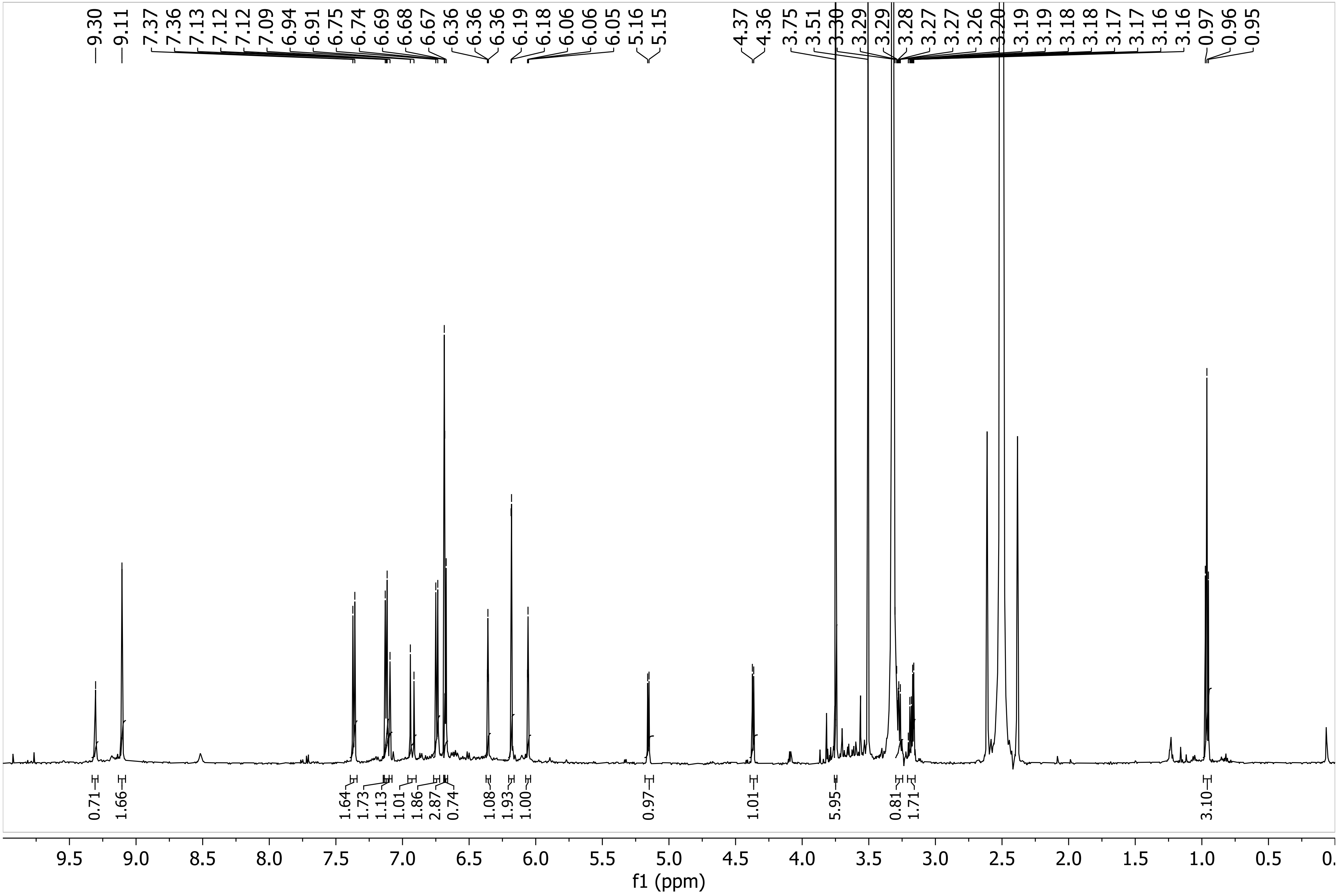

COSY NMR spectrum of compound **68** in DMSO-d_6

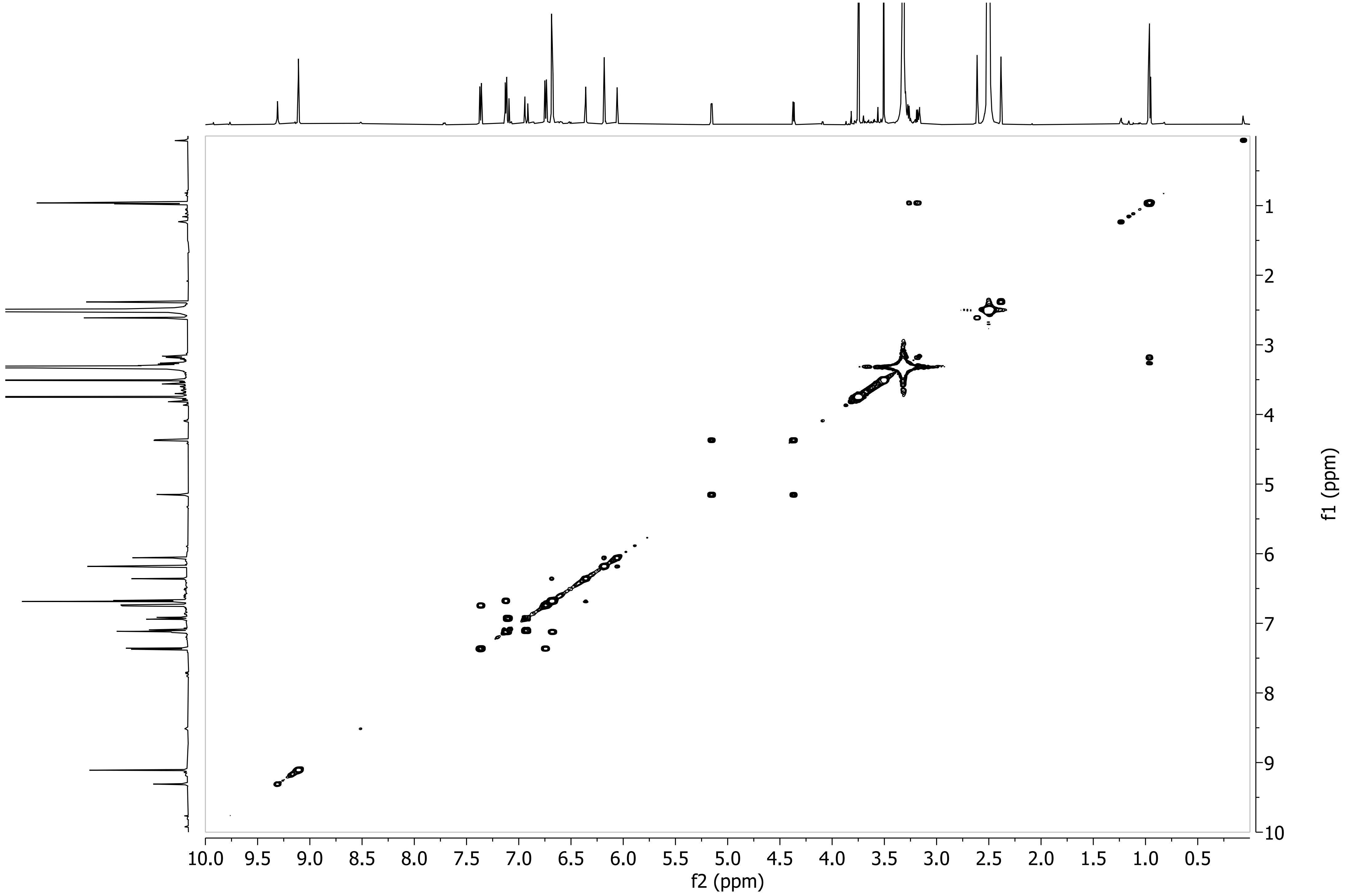

^{13}C-DEPTQ NMR spectrum of compound **68** in DMSO-d_6

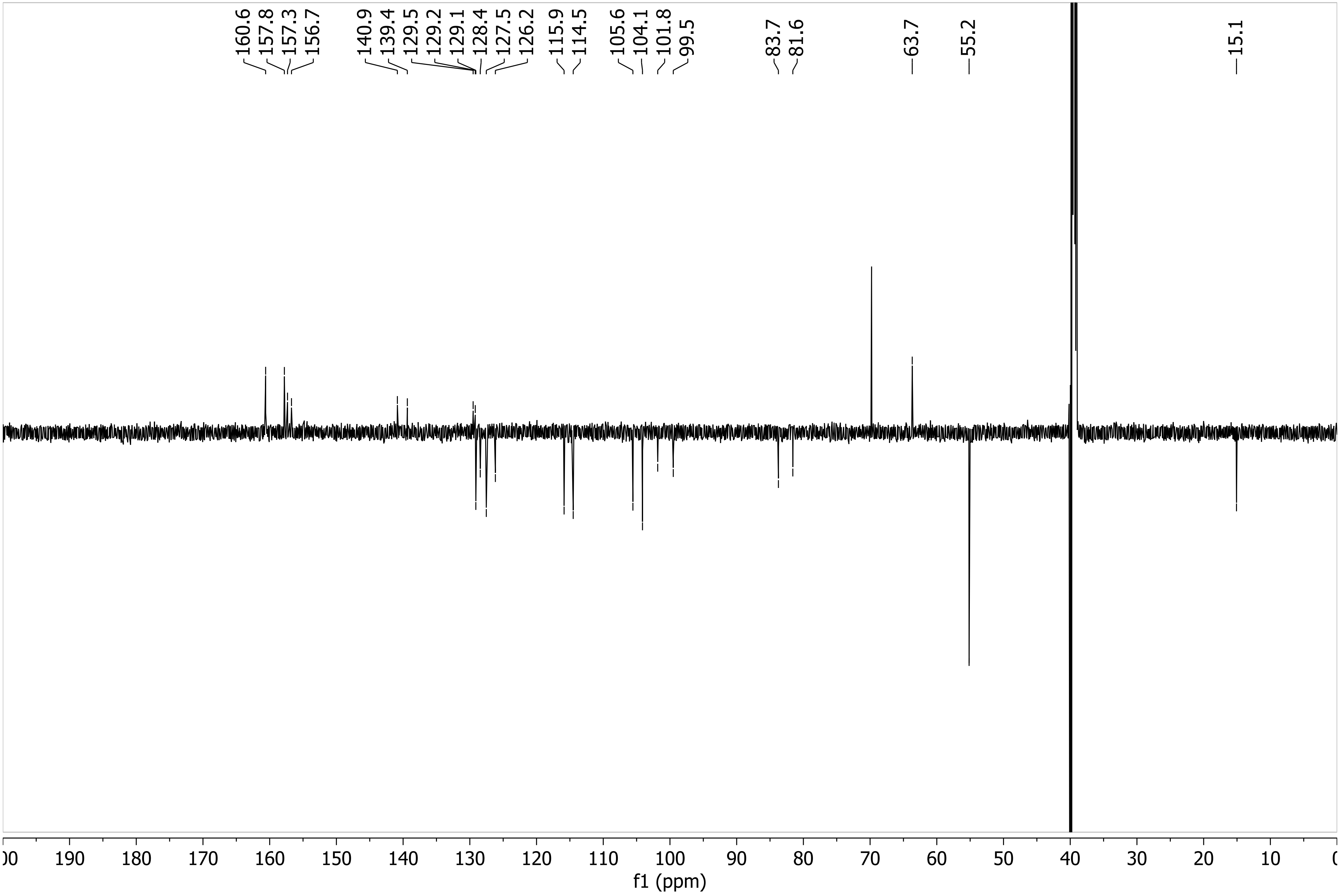

Edited-HSQC NMR spectrum of compound **68** in DMSO-d_6

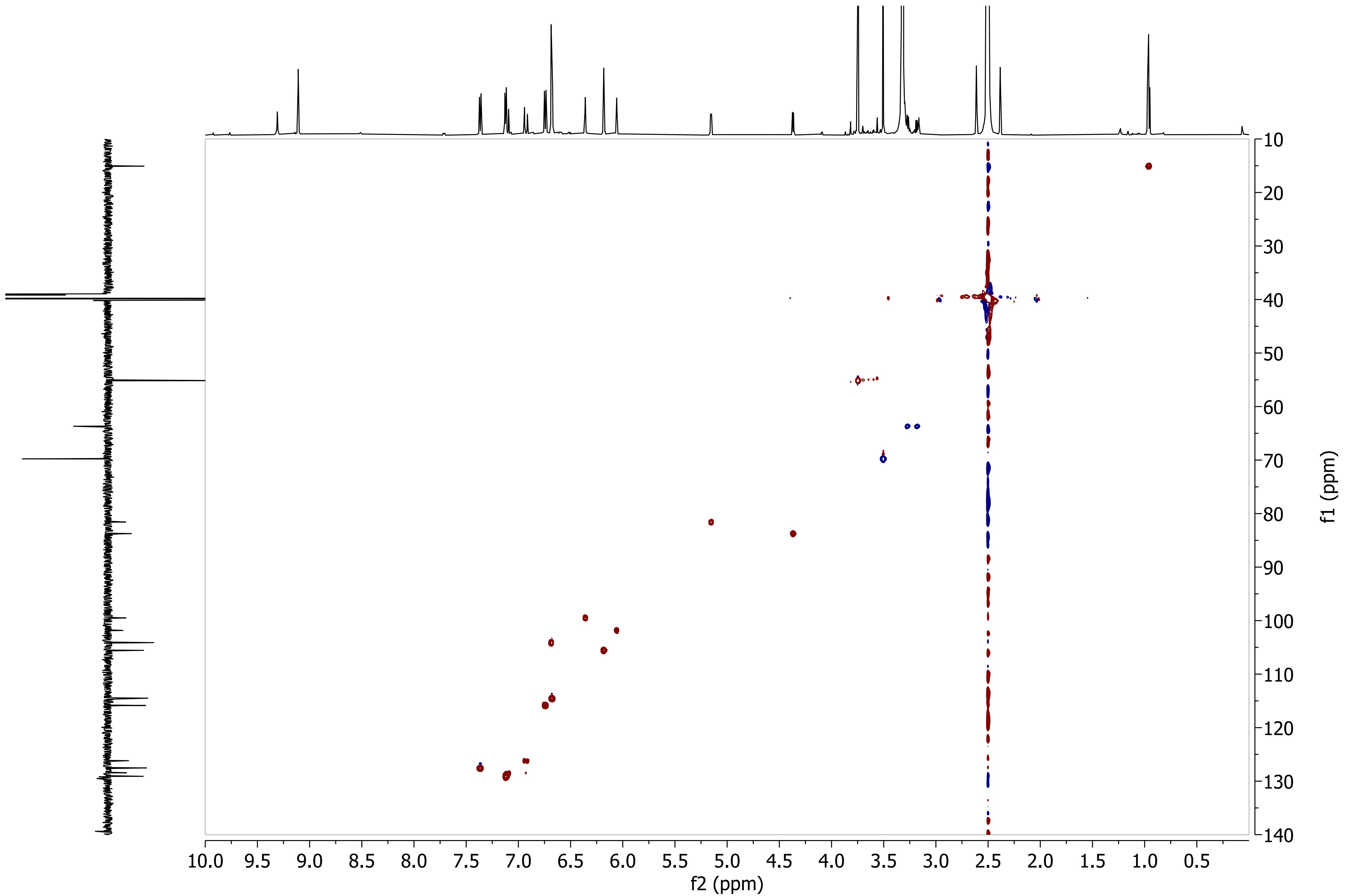

HMBC NMR spectrum of compound **68** in DMSO-d_6

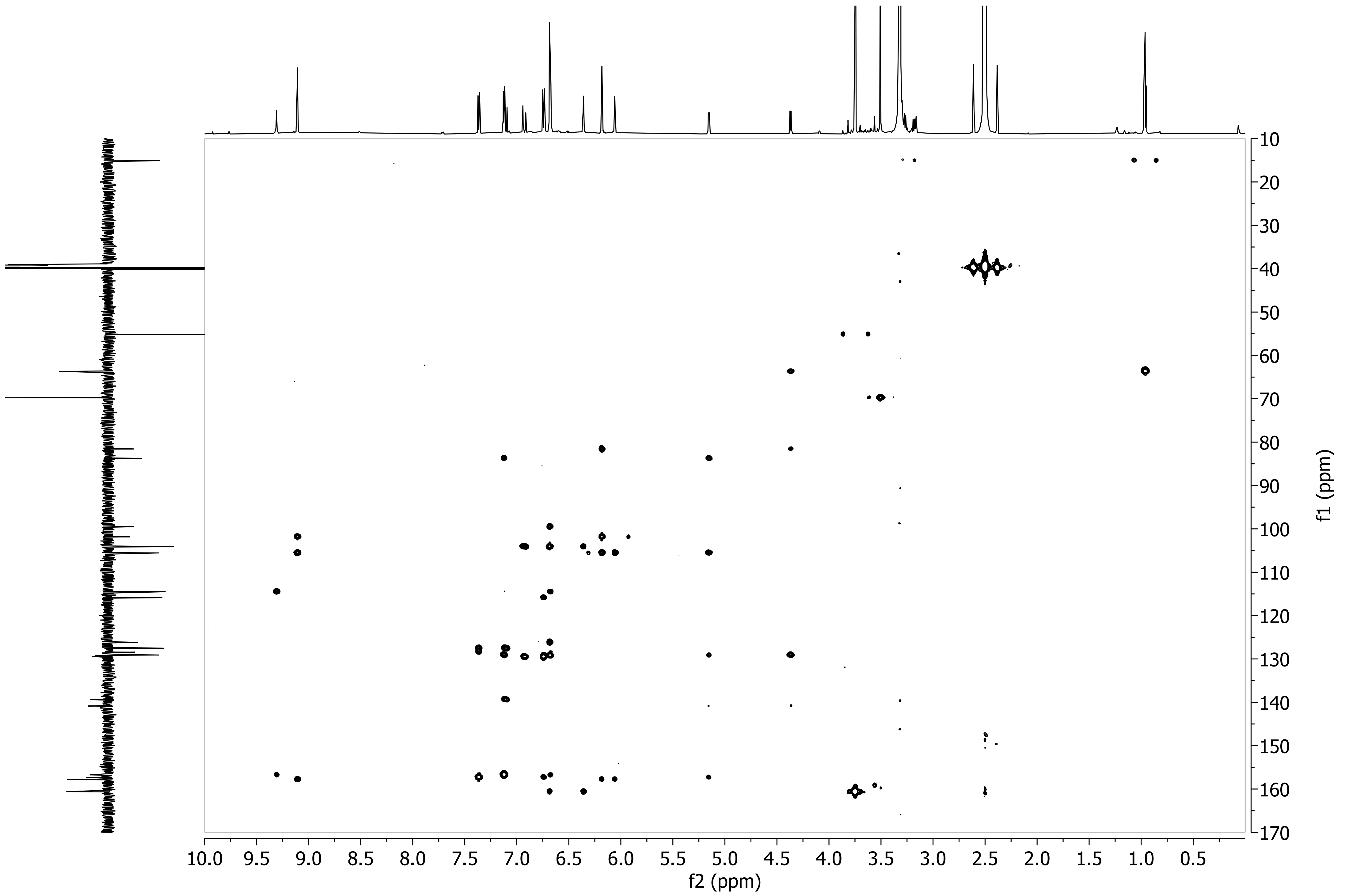

ROESY NMR spectrum of compound **68** in DMSO-d_6

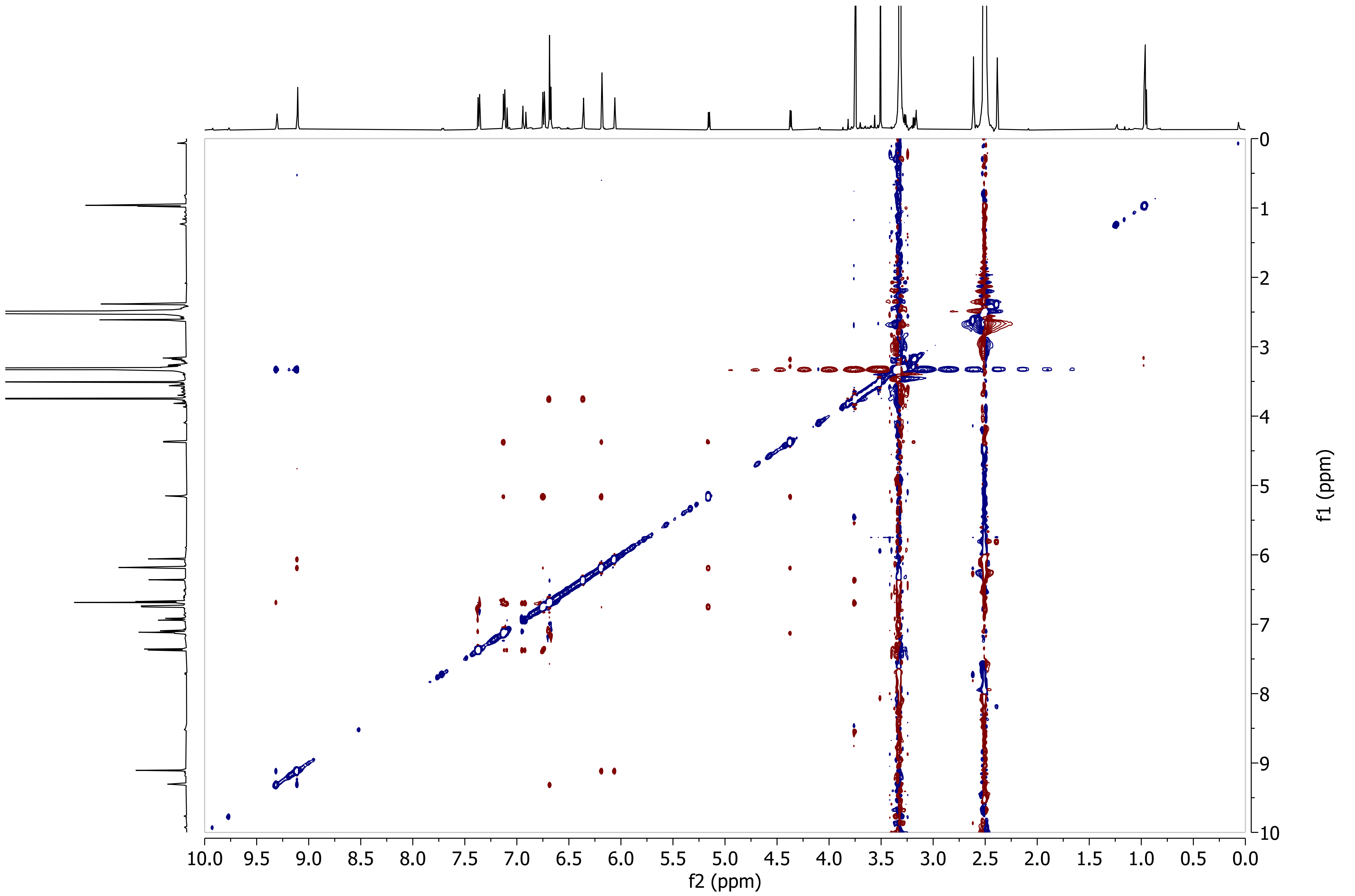

^{1}H NMR spectrum of compound **69** in DMSO-d_6

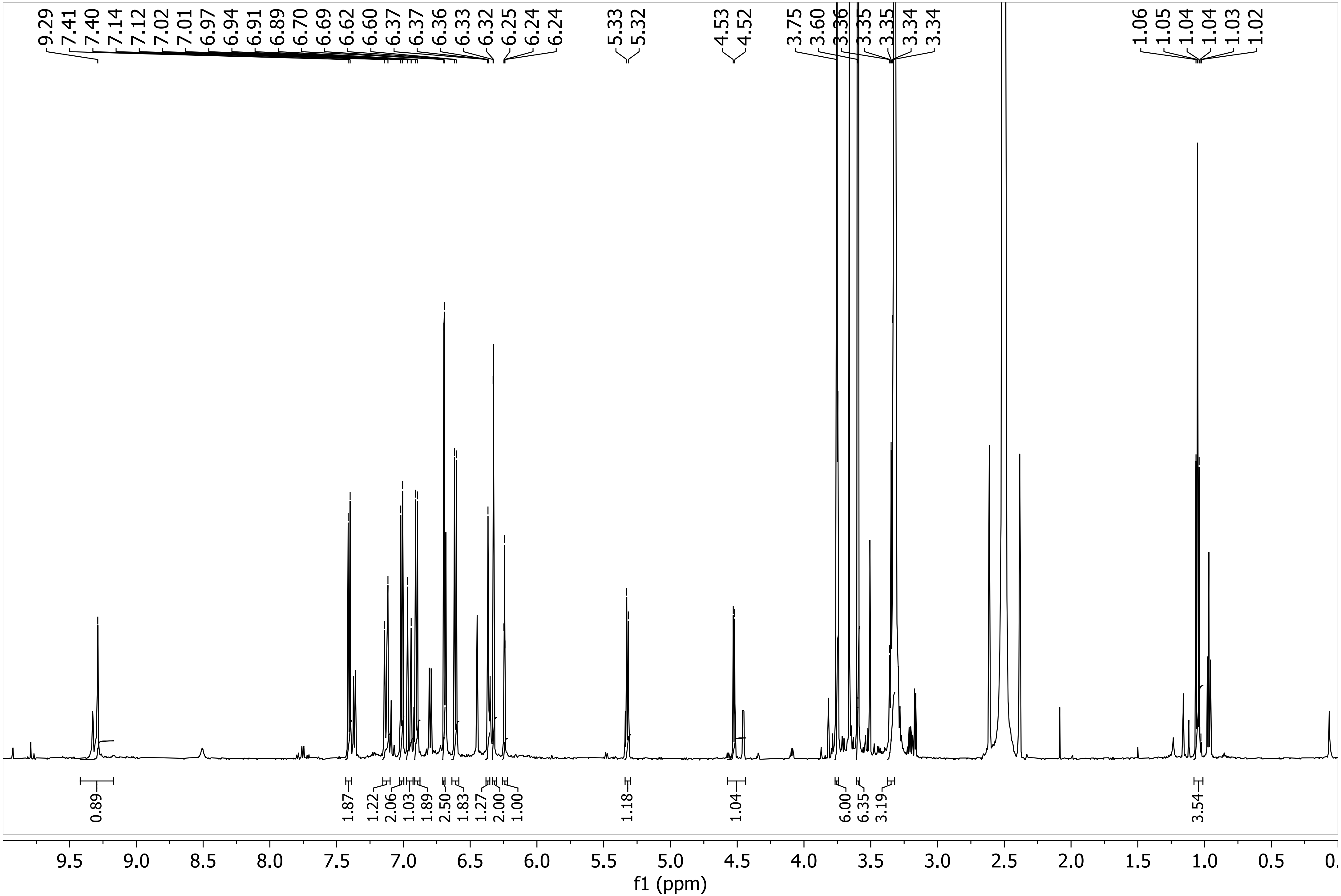

COSY NMR spectrum of compound **69** in DMSO-d_6

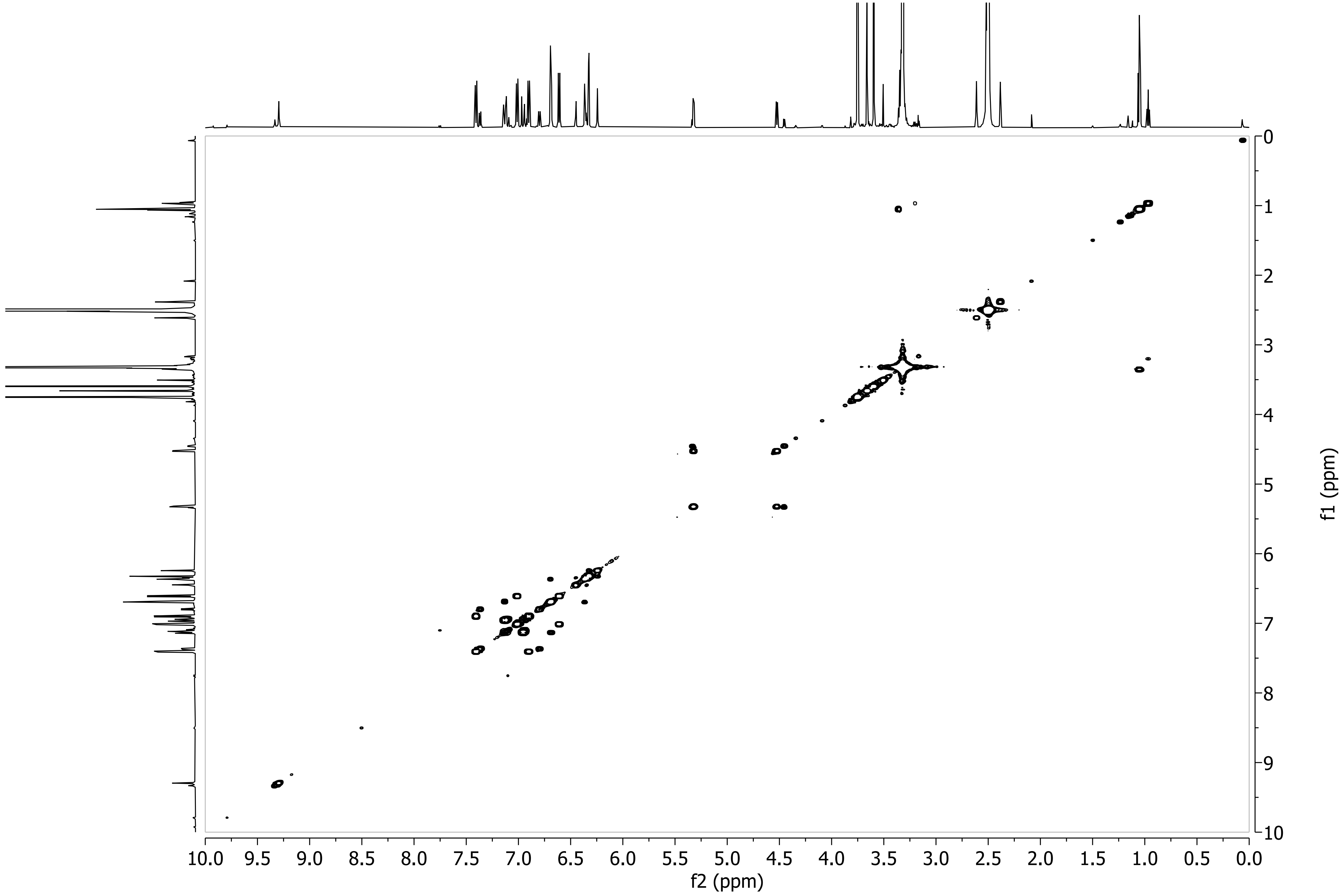

^{13}C-DEPTQ NMR spectrum of compound **69** in DMSO-d_6

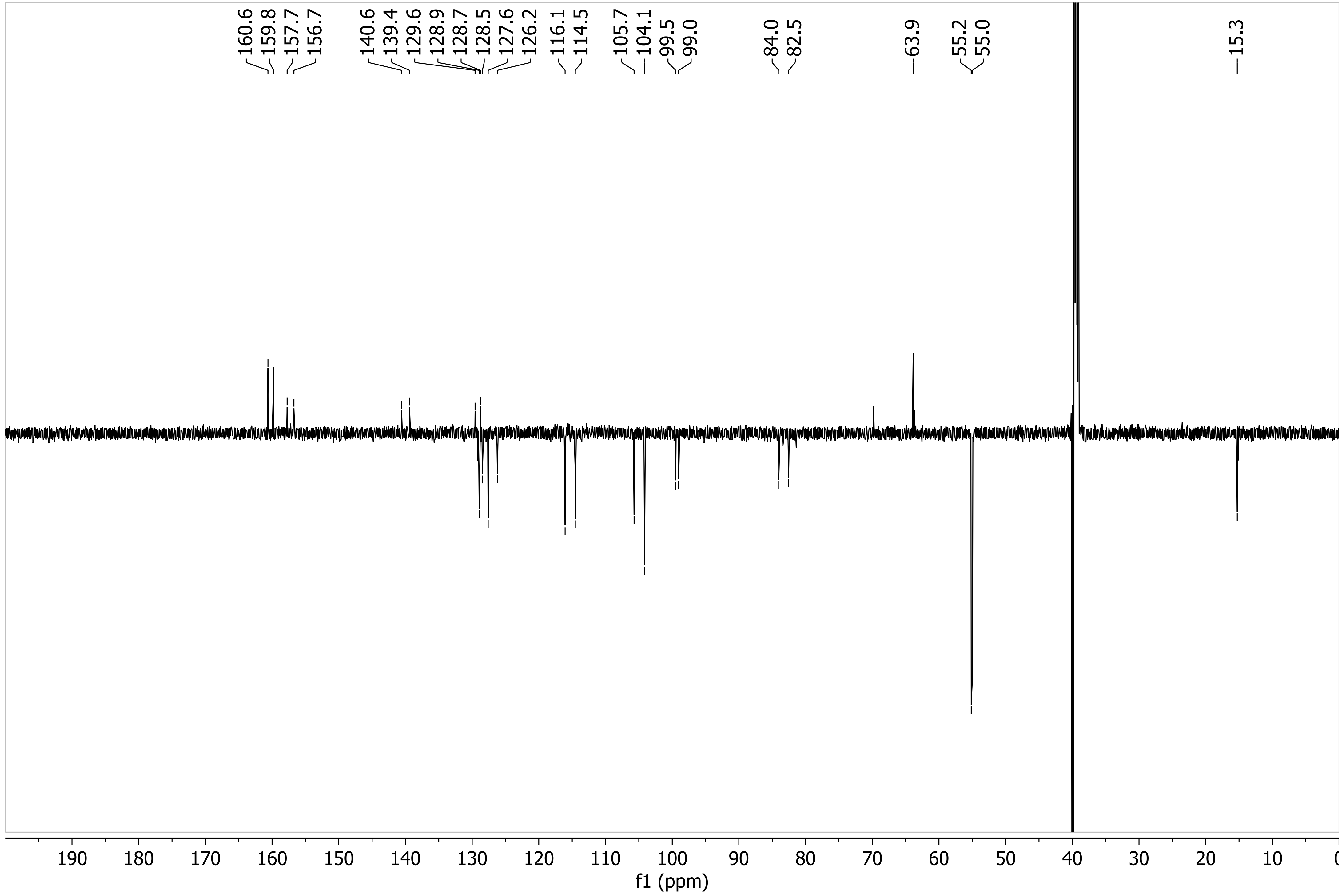

Edited-HSQC NMR spectrum of compound **69** in DMSO-d_6

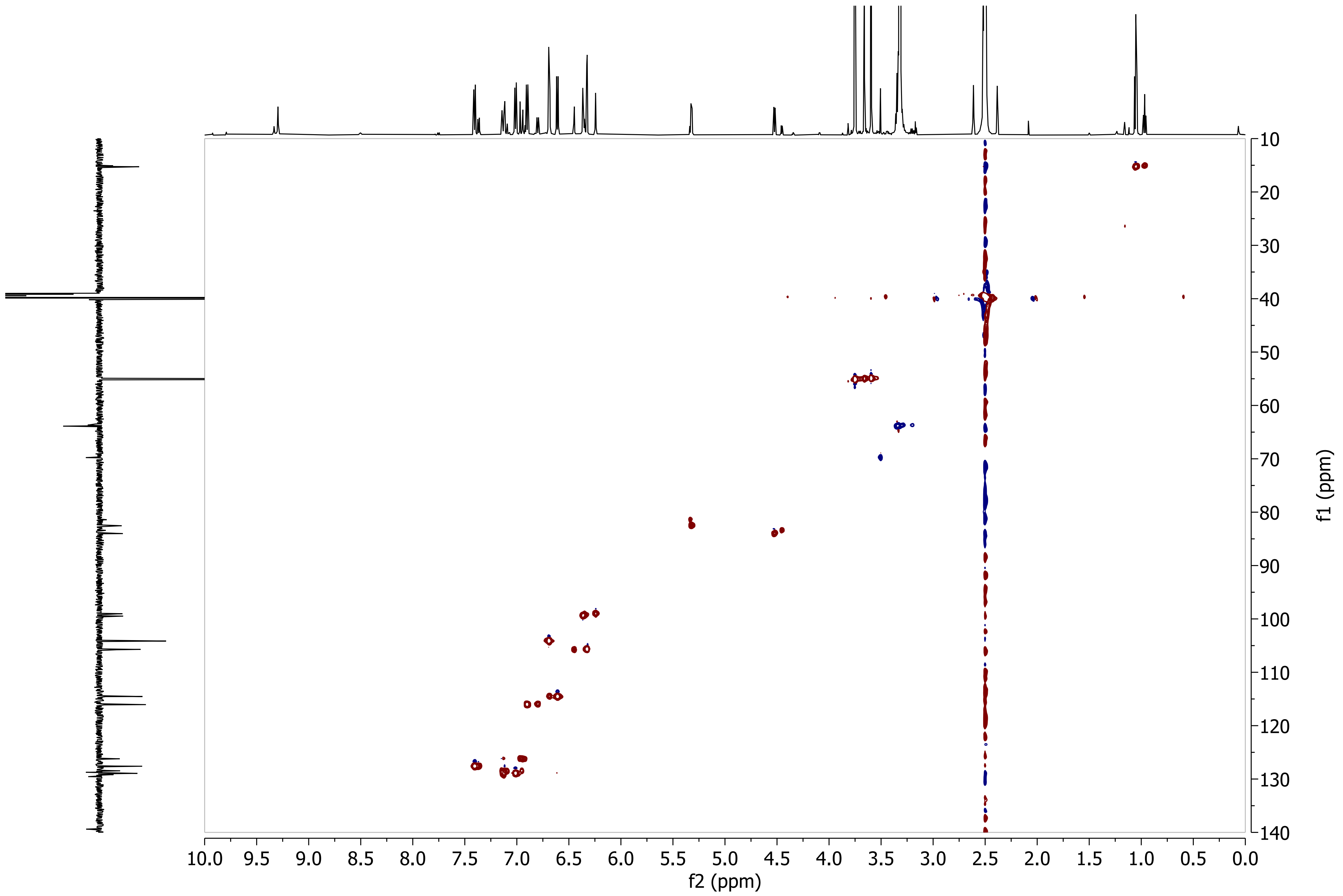

HMBC NMR spectrum of compound **69** in DMSO-d_6

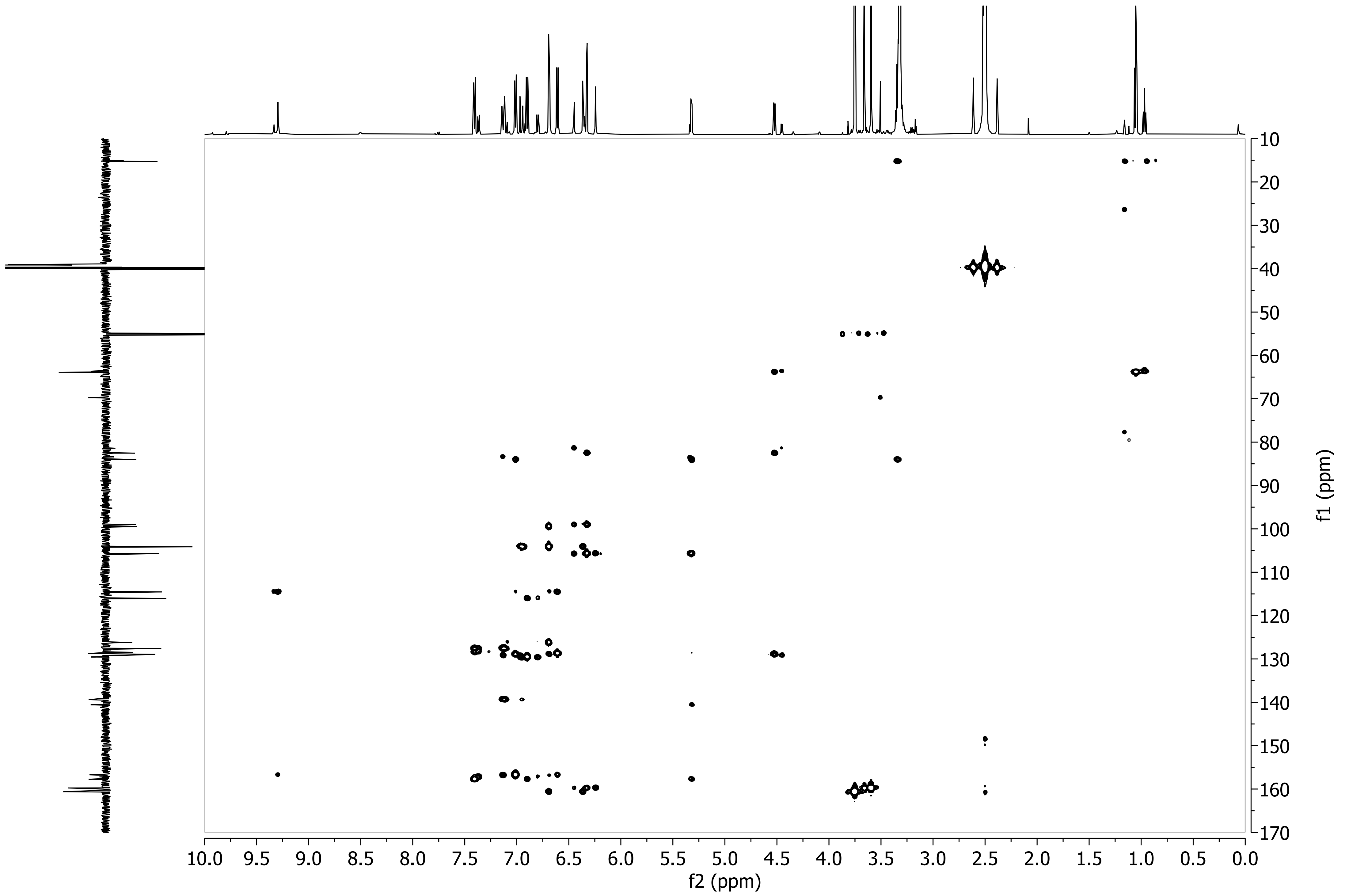

ROESY NMR spectrum of compound **69** in DMSO-d_6

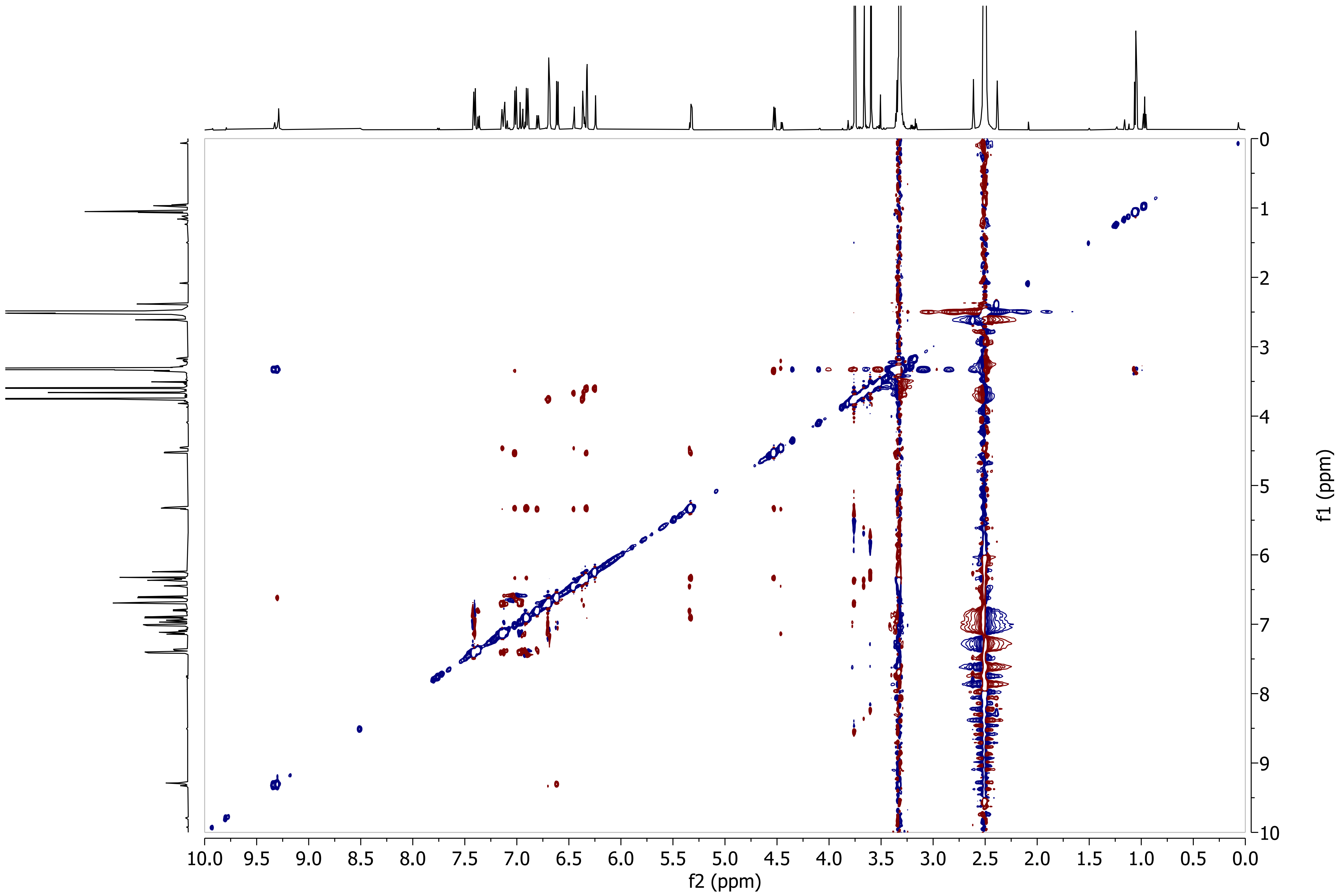

^{1}H NMR spectrum of compound **70** in DMSO-d_6

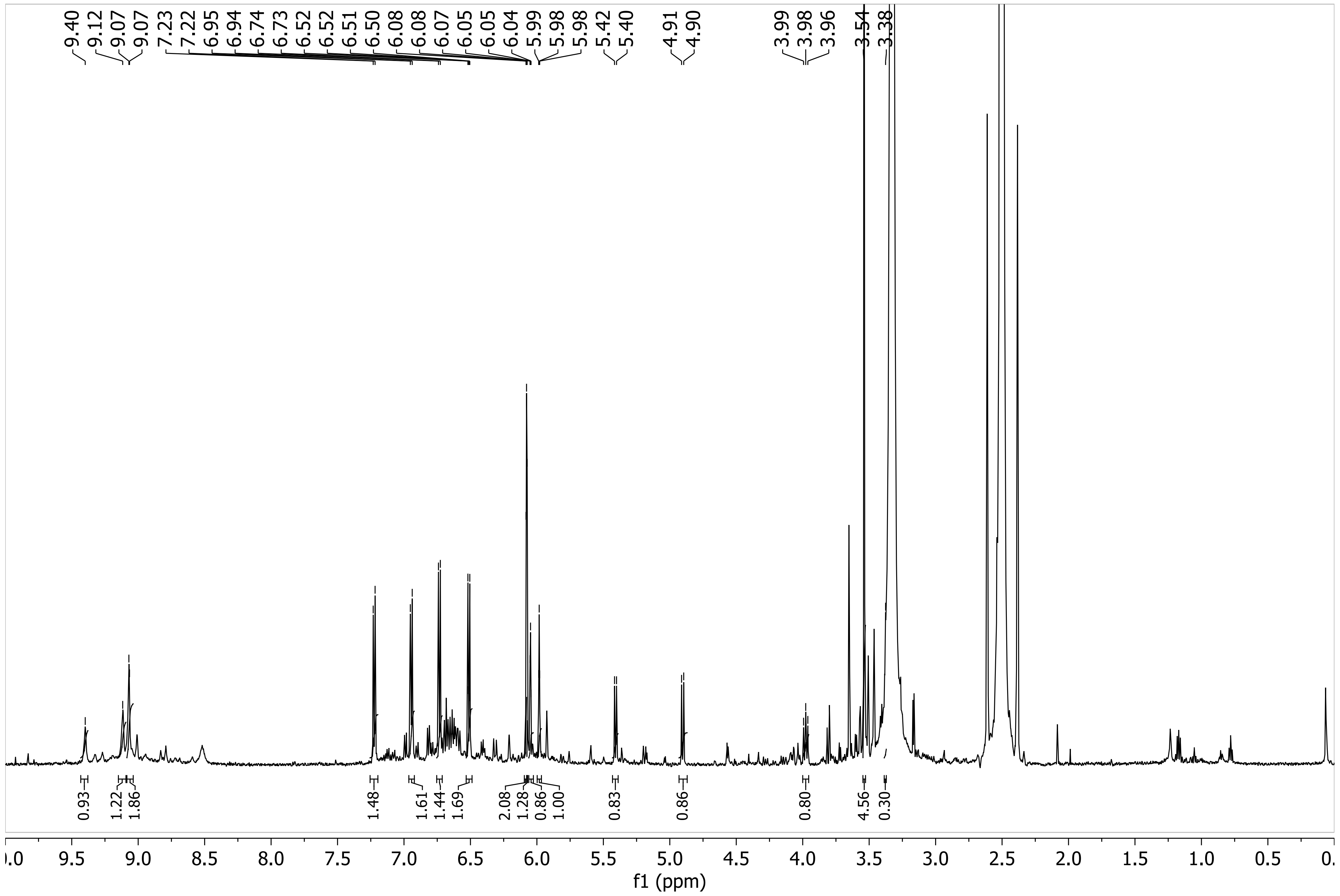

^{1}H NMR spectrum of compound **70** in DMSO-d_6

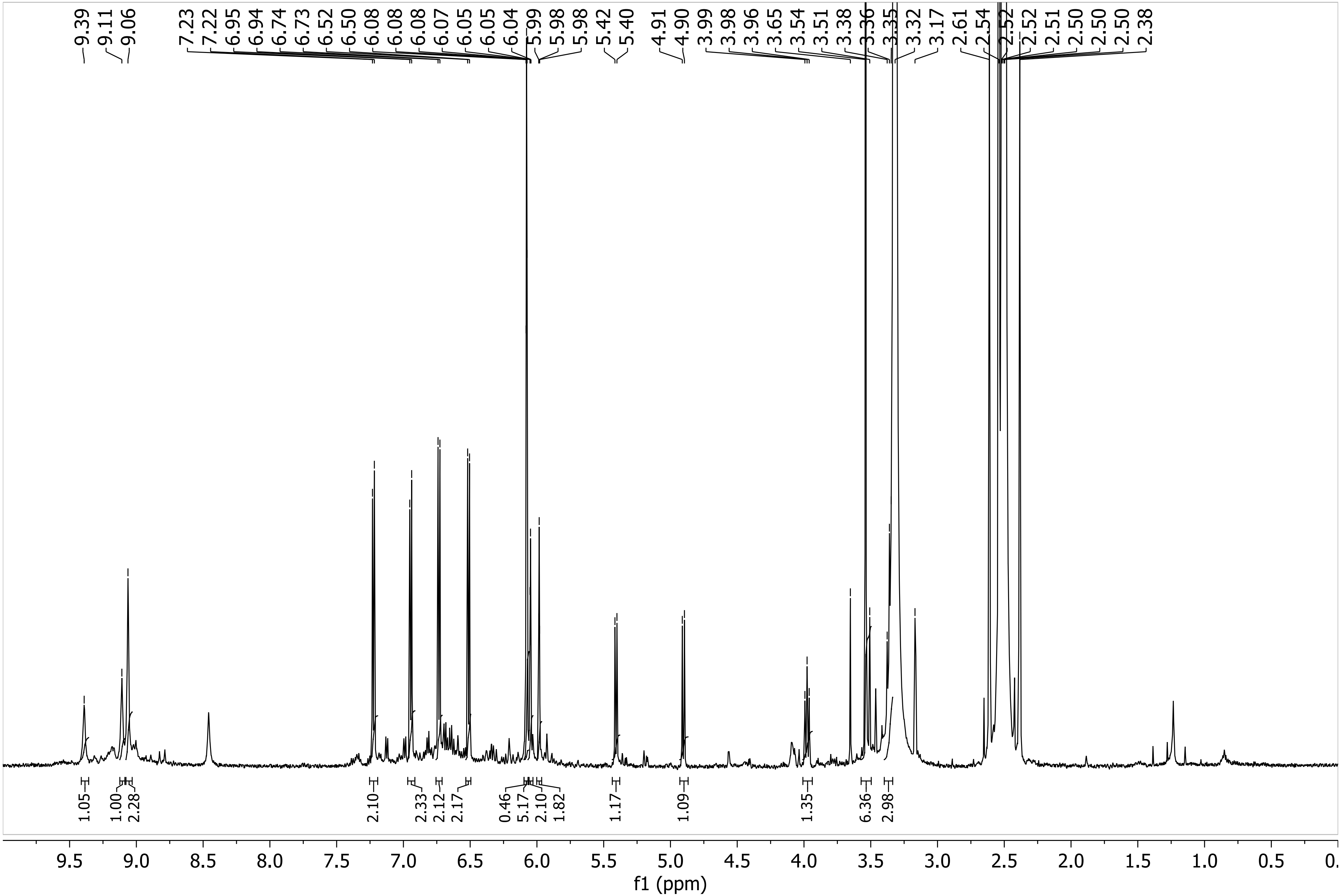

COSY NMR spectrum of compound **70** in DMSO-d_6

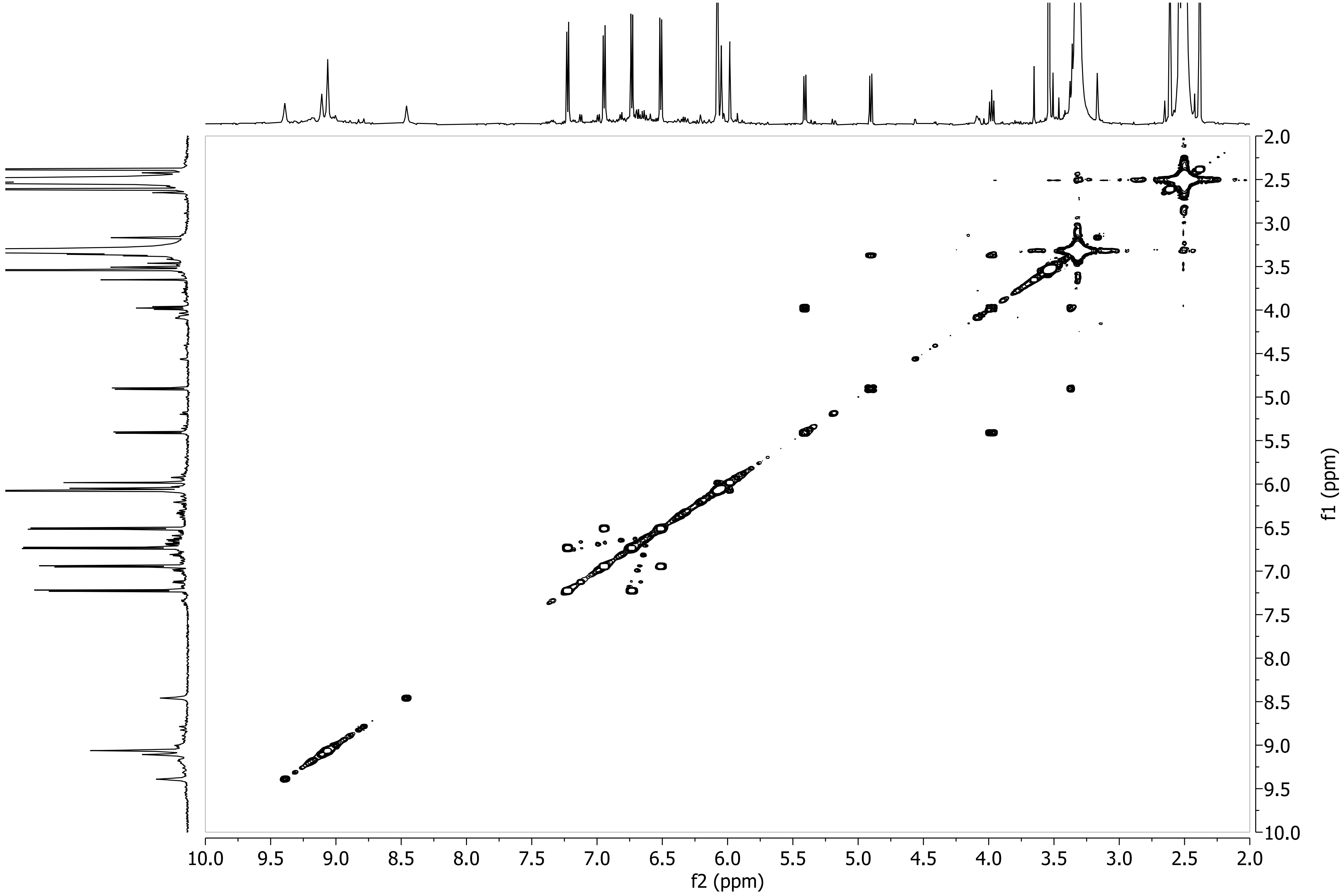

Edited-HSQC NMR spectrum of compound **70** in DMSO-d_6

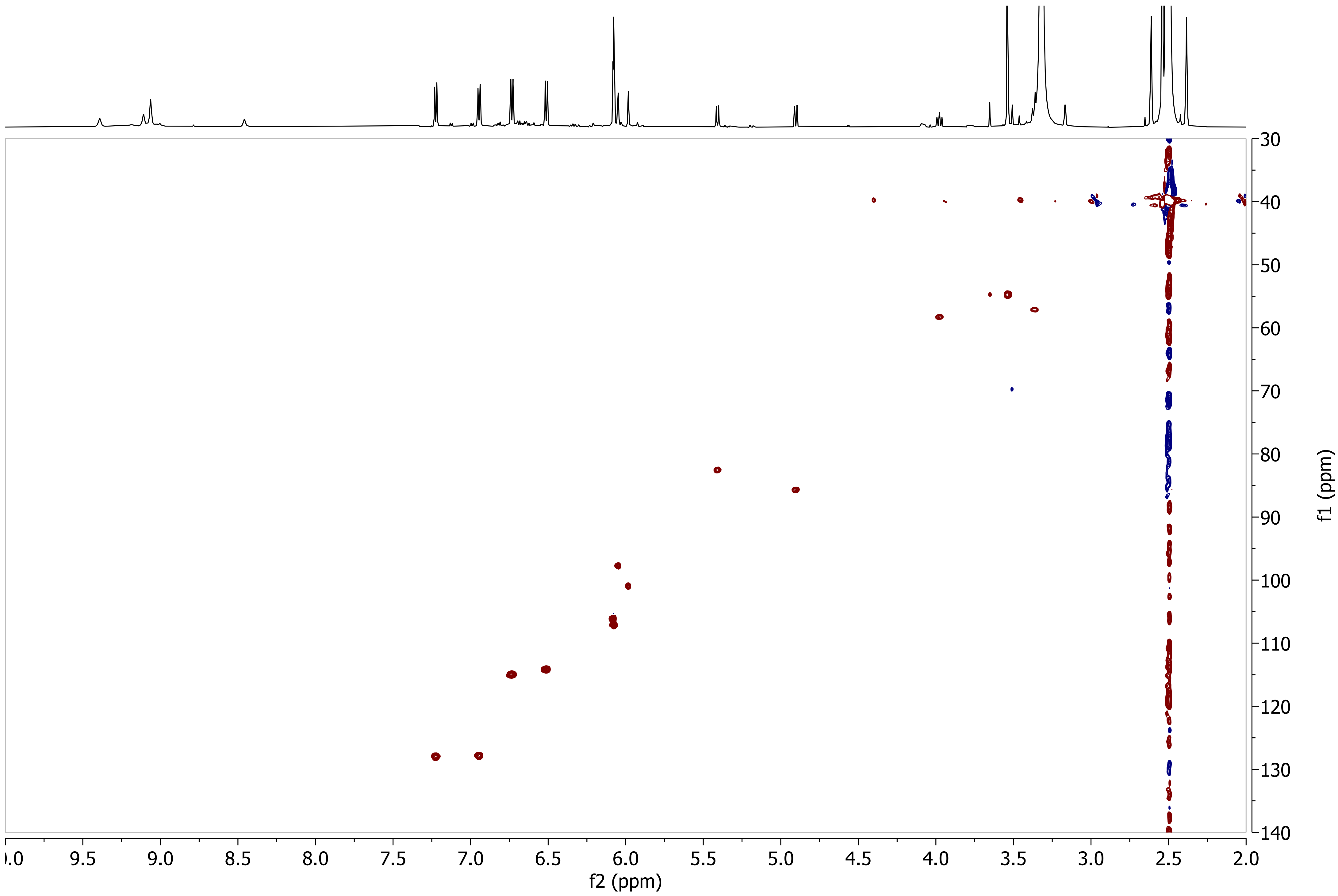

HMBC NMR spectrum of compound **70** in DMSO-d_6

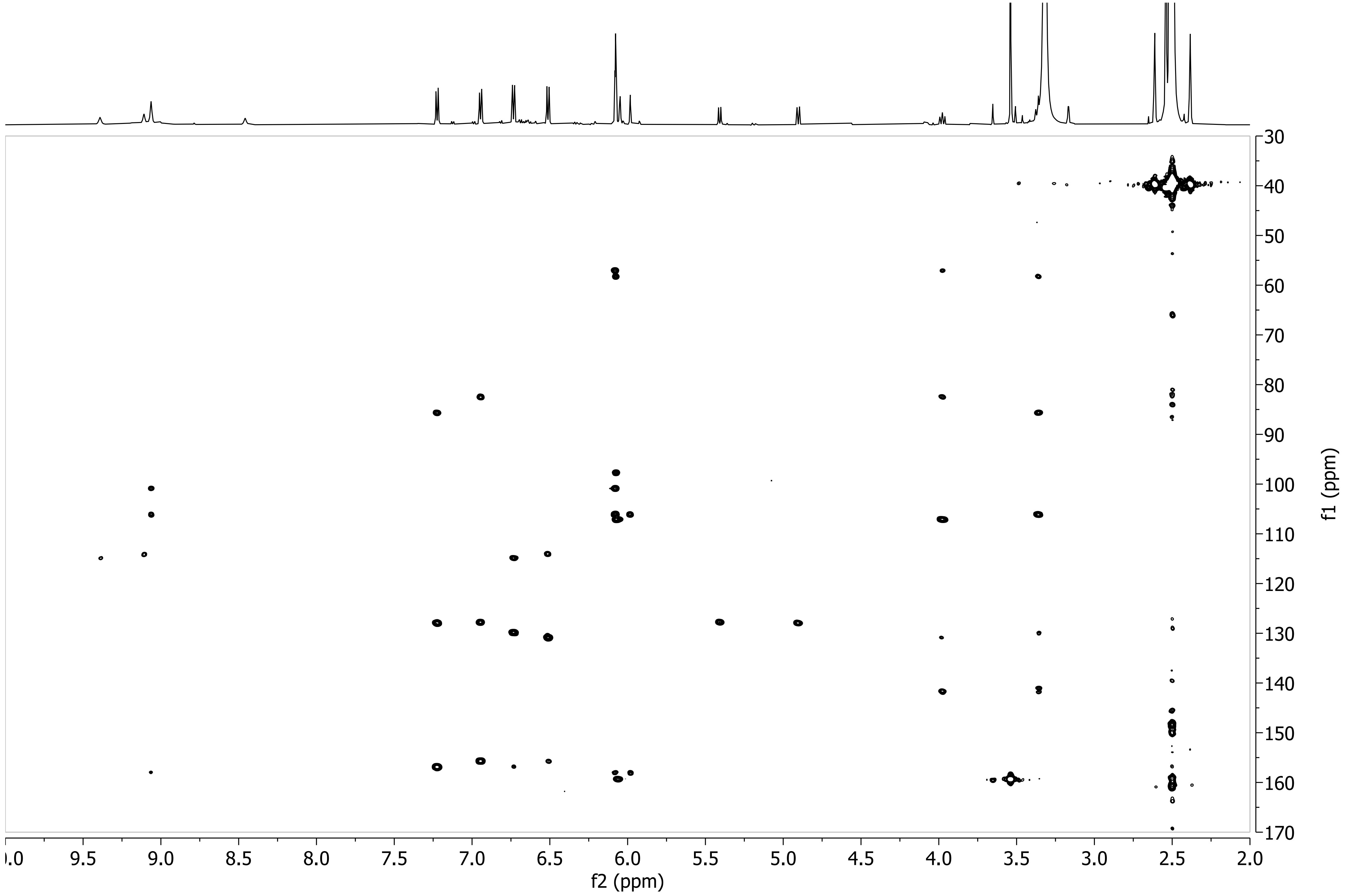

ROESY NMR spectrum of compound **70** in DMSO-d_6

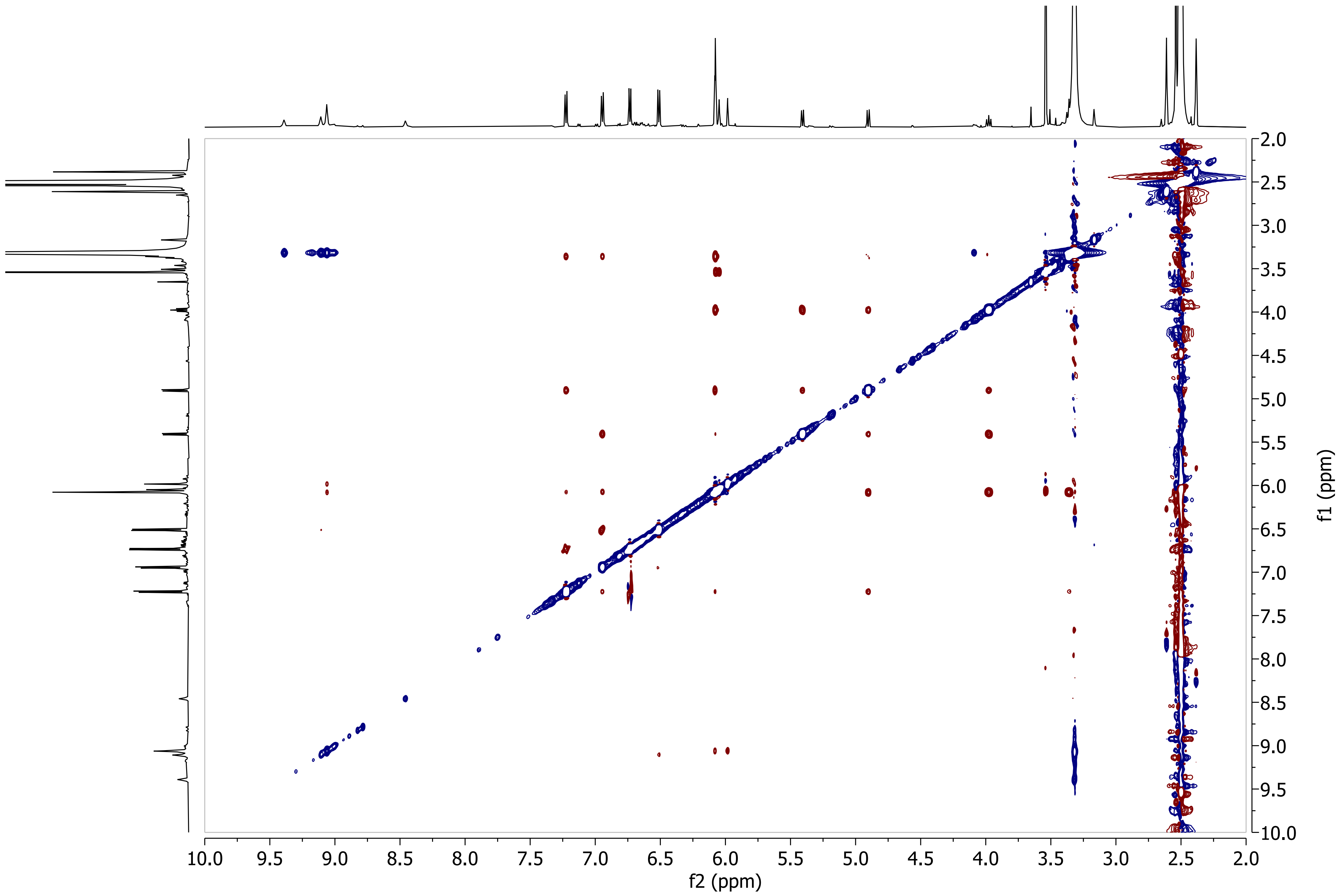

^{1}H NMR spectrum of compound **71** in DMSO-d_6

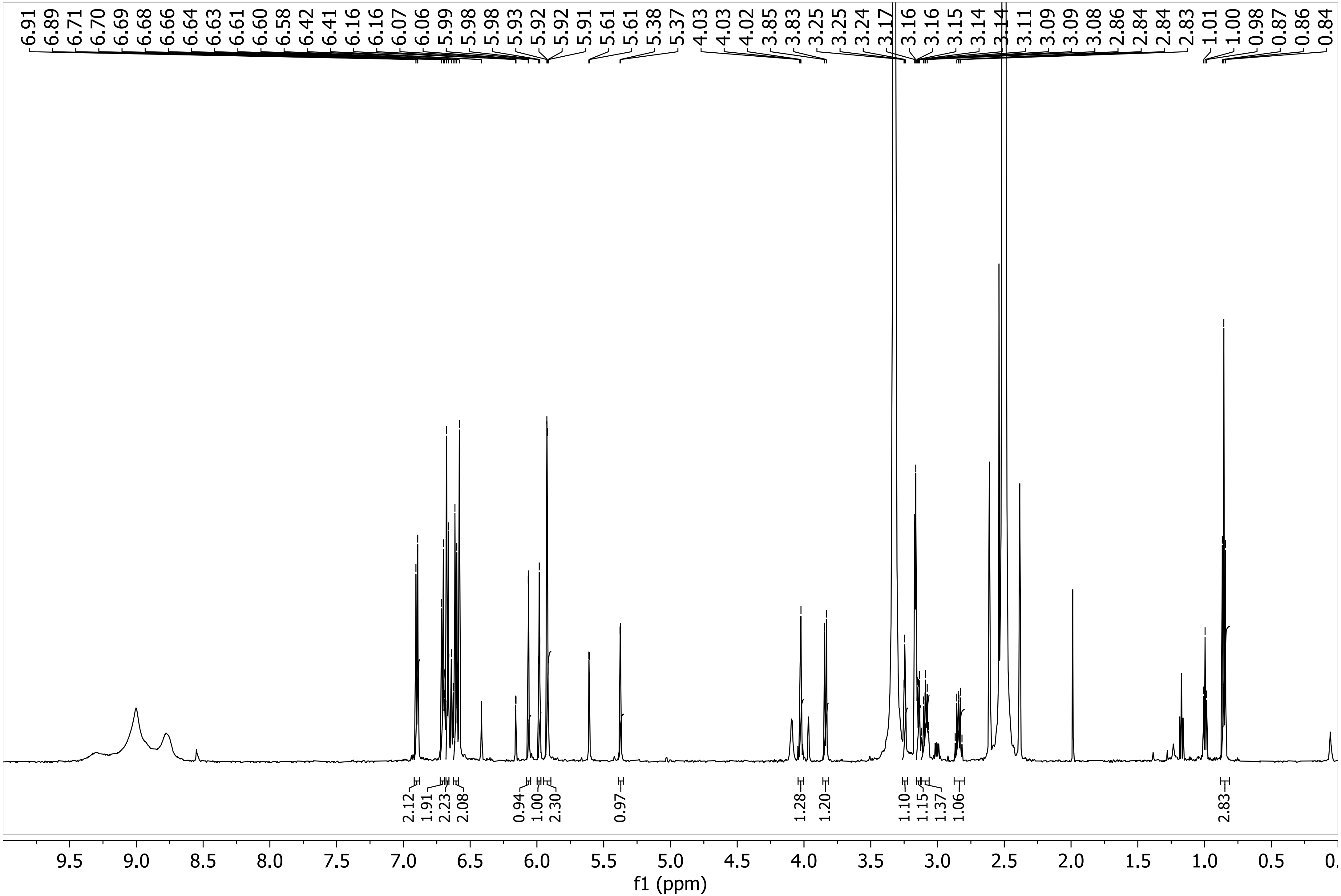

COSY NMR spectrum of compound **71** in DMSO-d_6

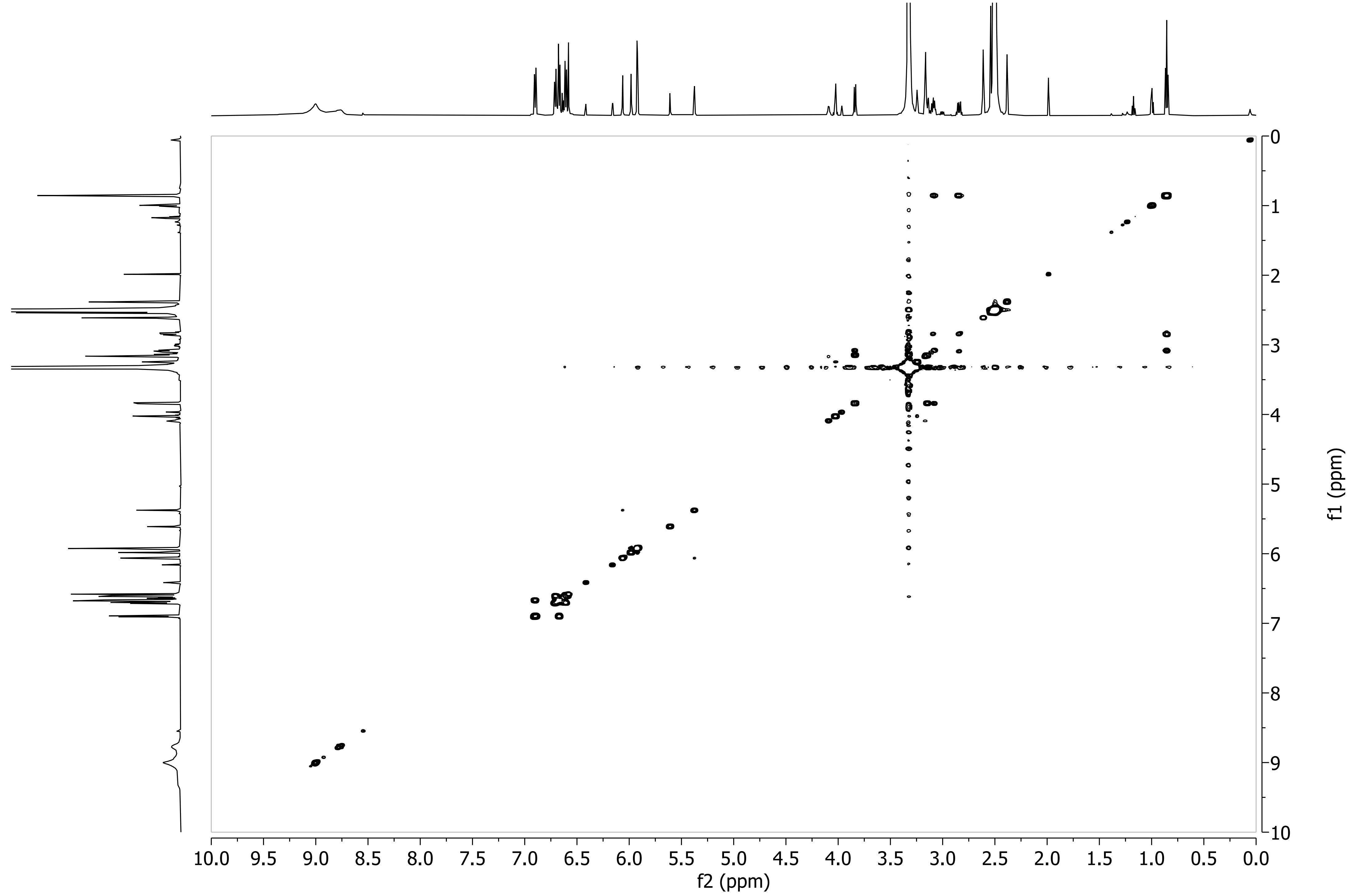

Edited-HSQC NMR spectrum of compound **71** in DMSO-d_6

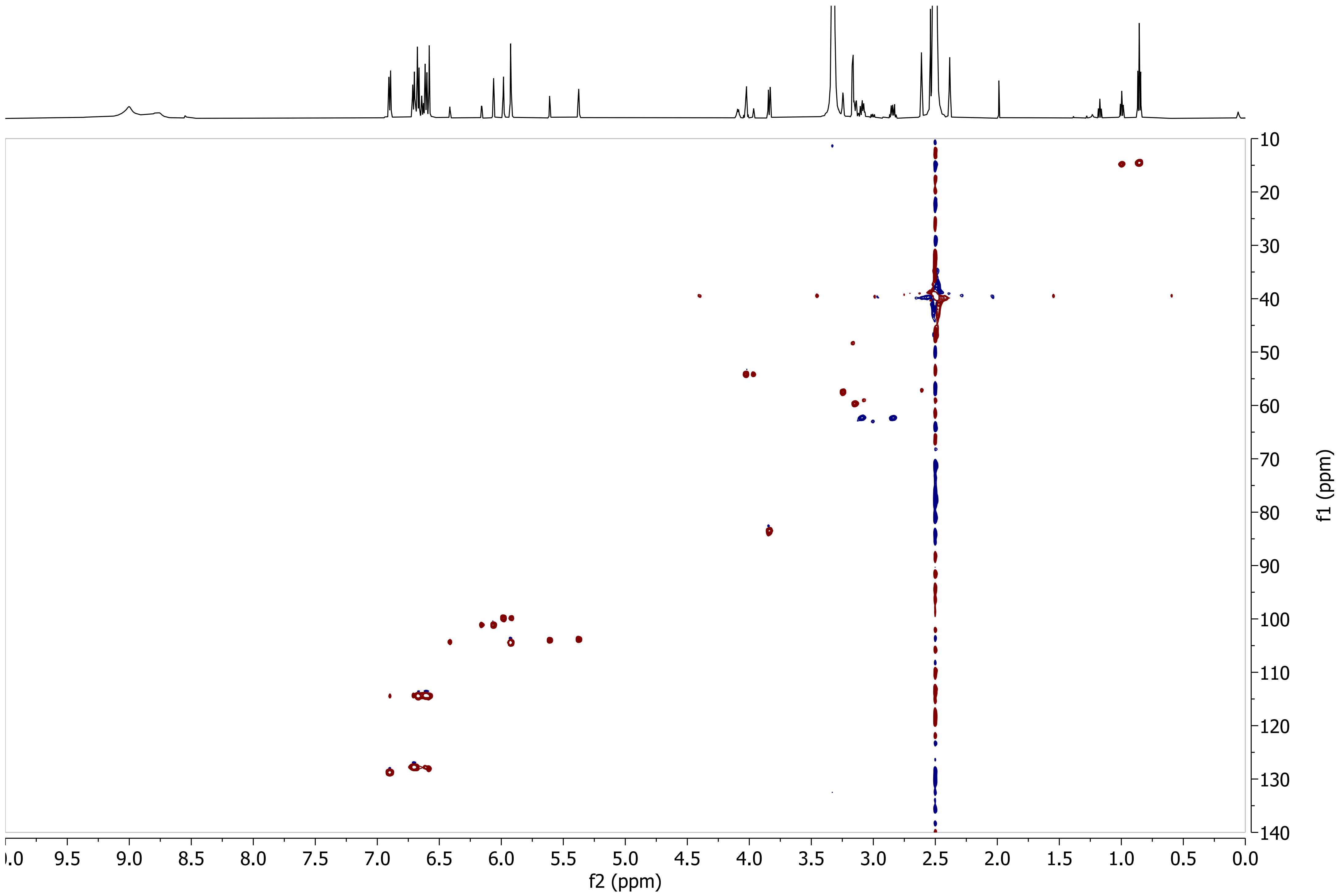

HMBC NMR spectrum of compound **71** in DMSO-d_6

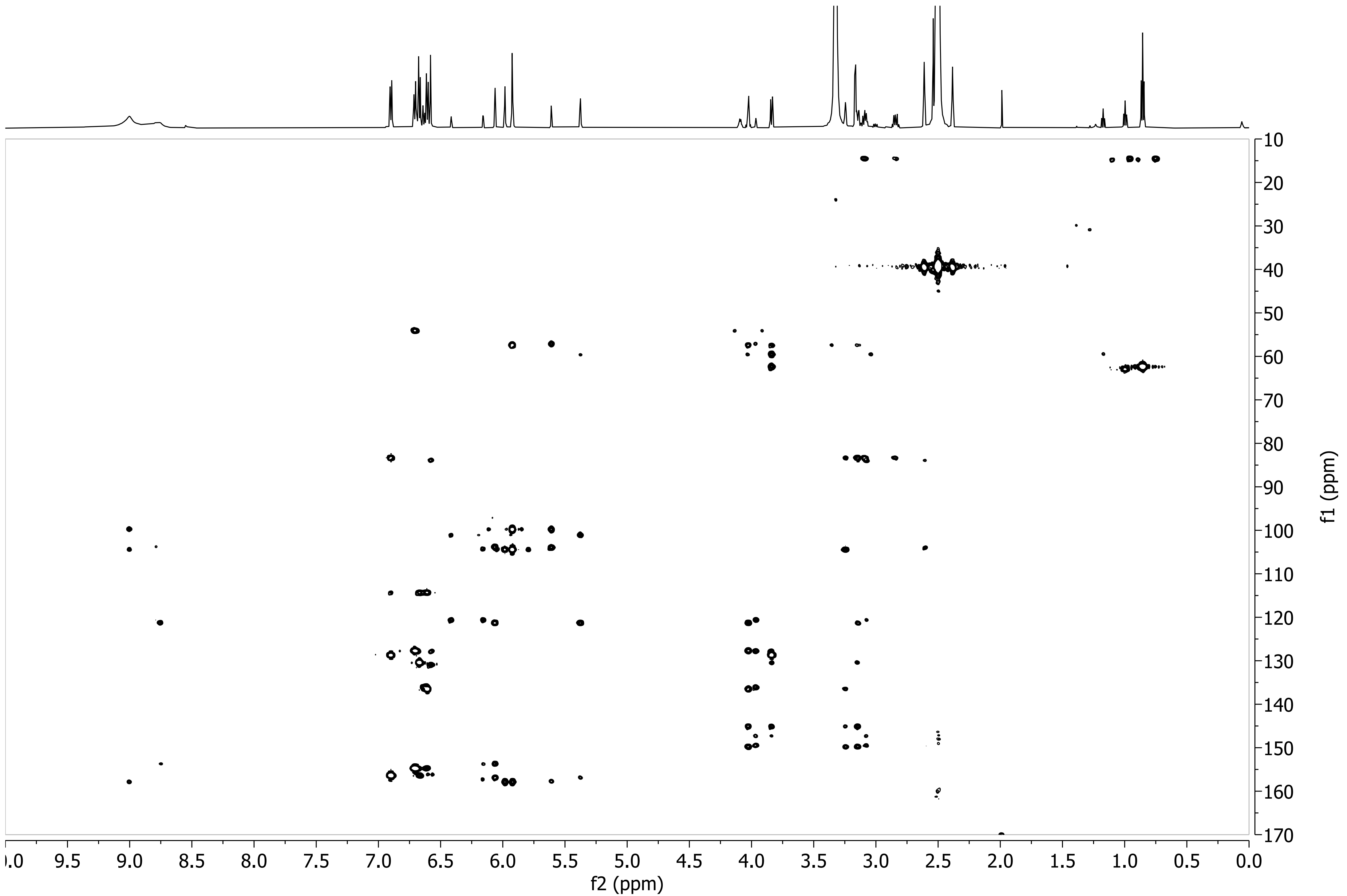

ROESY NMR spectrum of compound **71** in DMSO-d_6

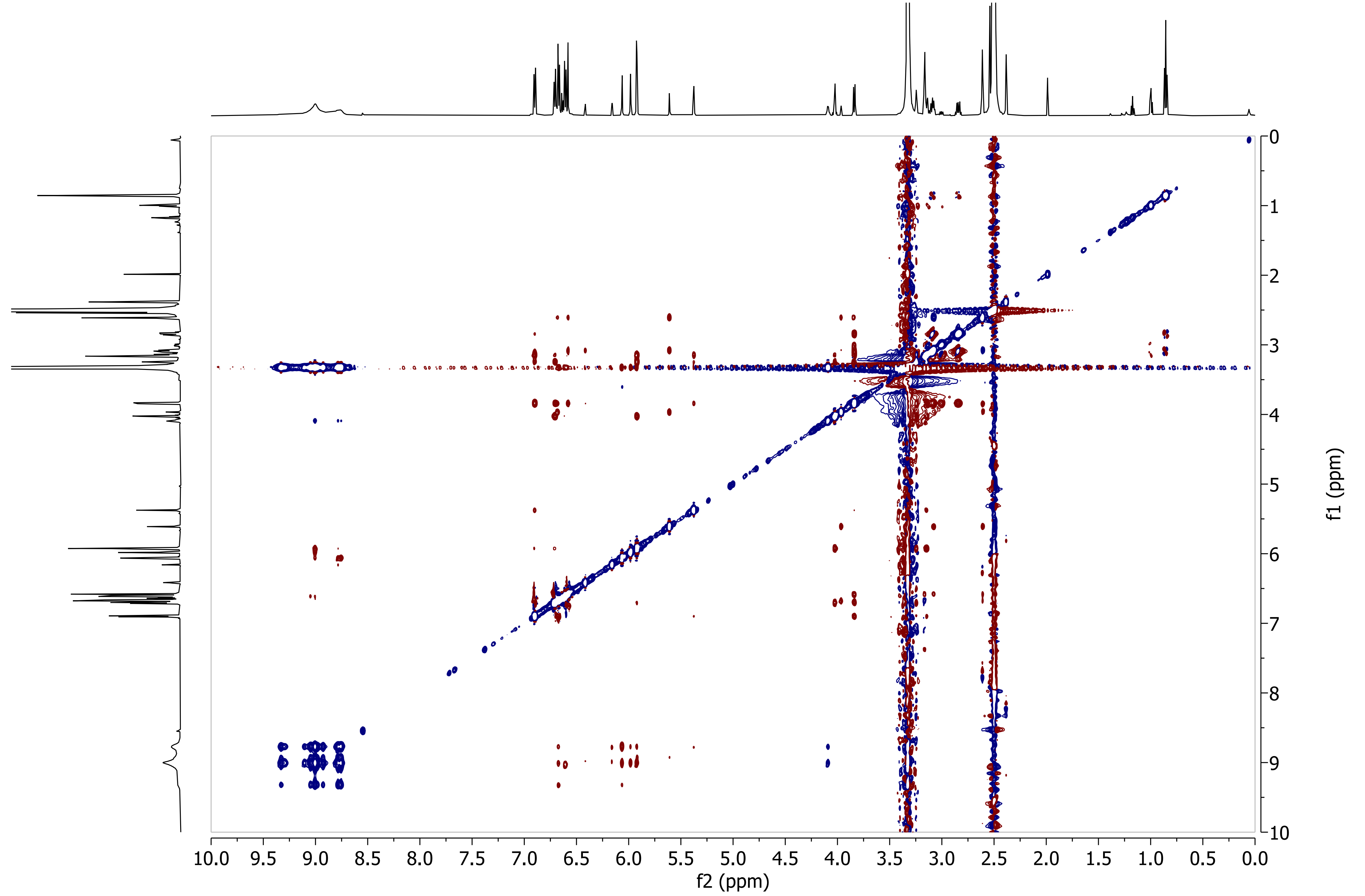

^{1}H NMR spectrum of compound **72** in DMSO-d_6

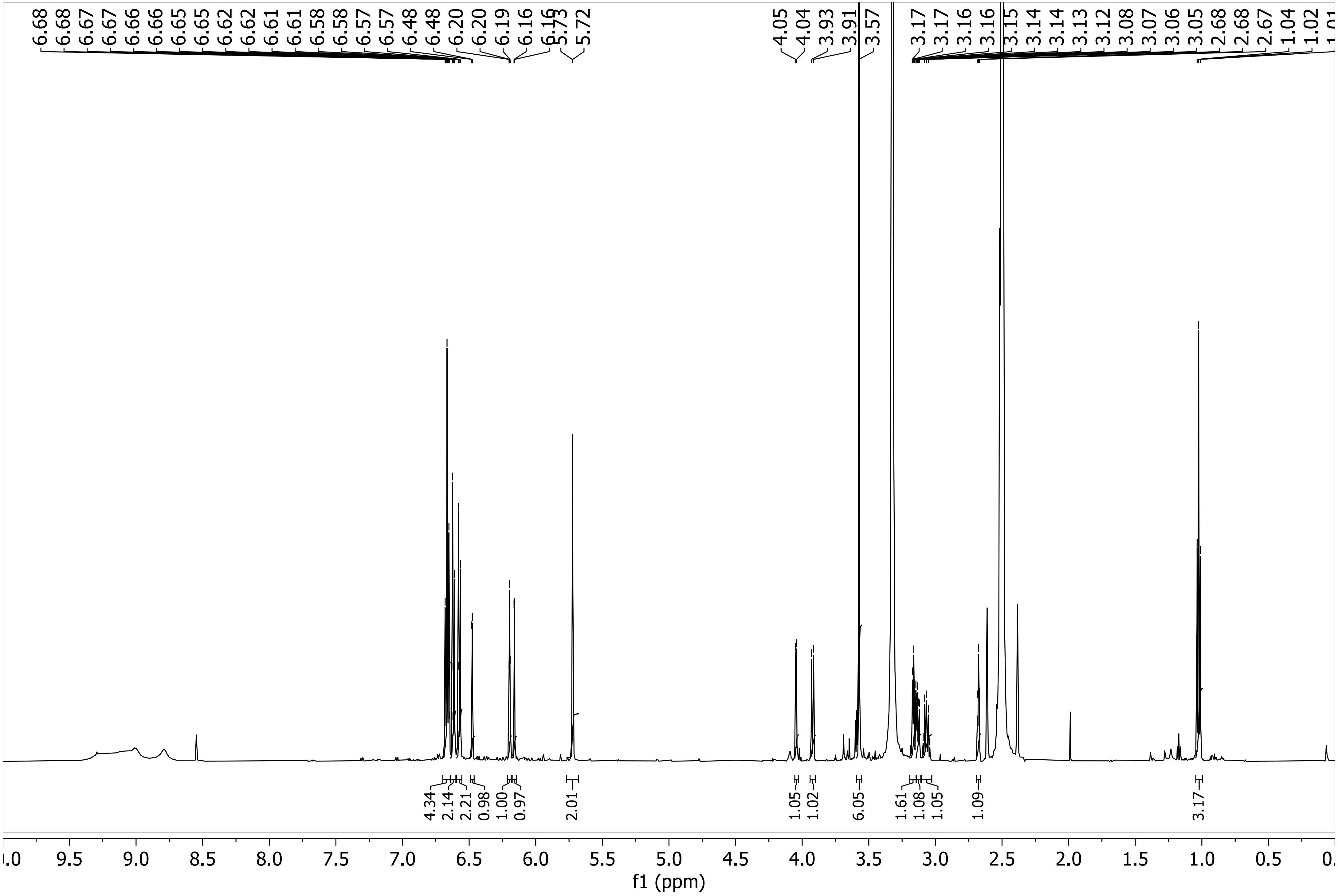

COSY NMR spectrum of compound **72** in DMSO-d_6

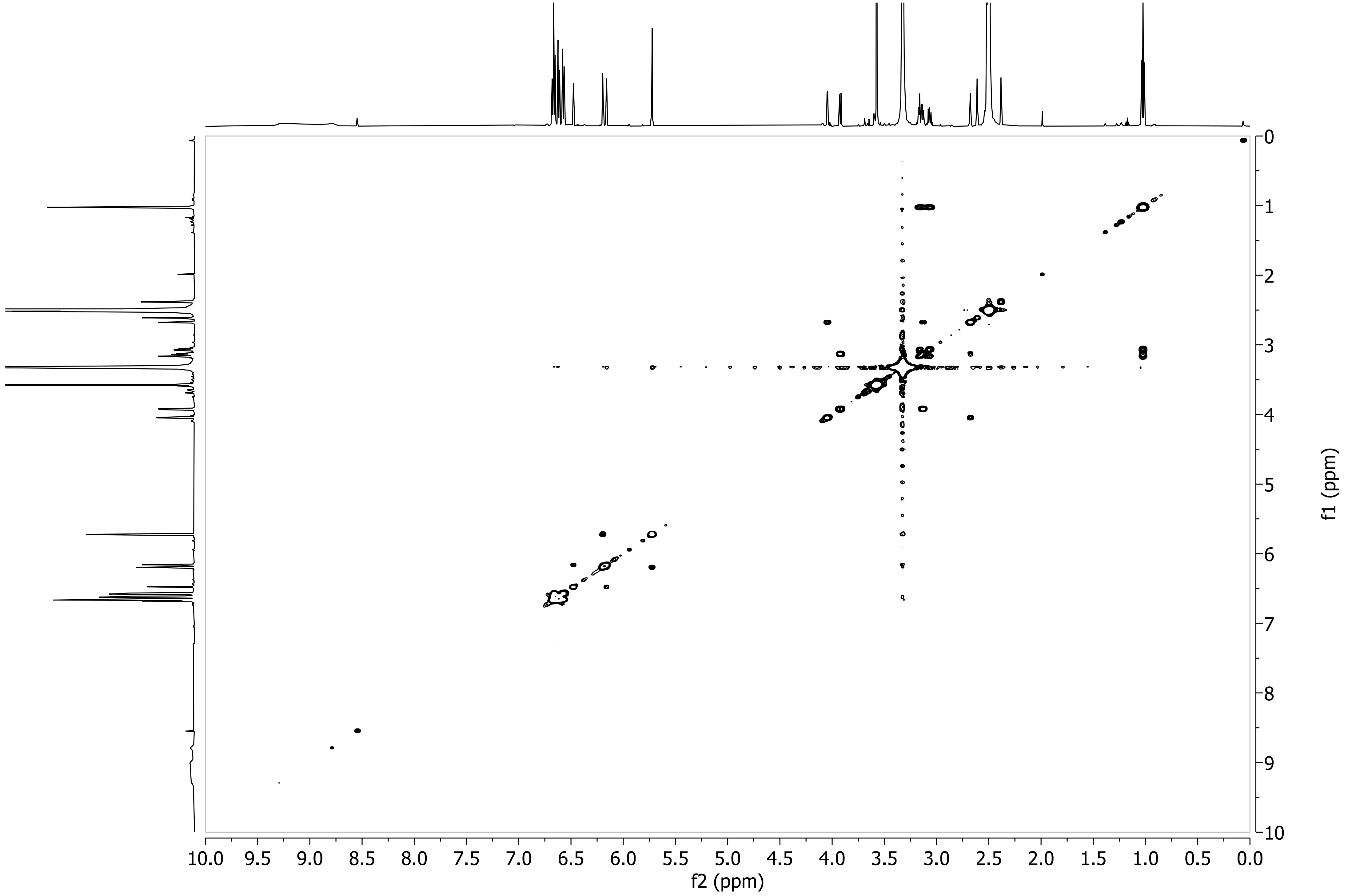

^{13}C-DEPTQ NMR spectrum of compound **72** in DMSO-d_6

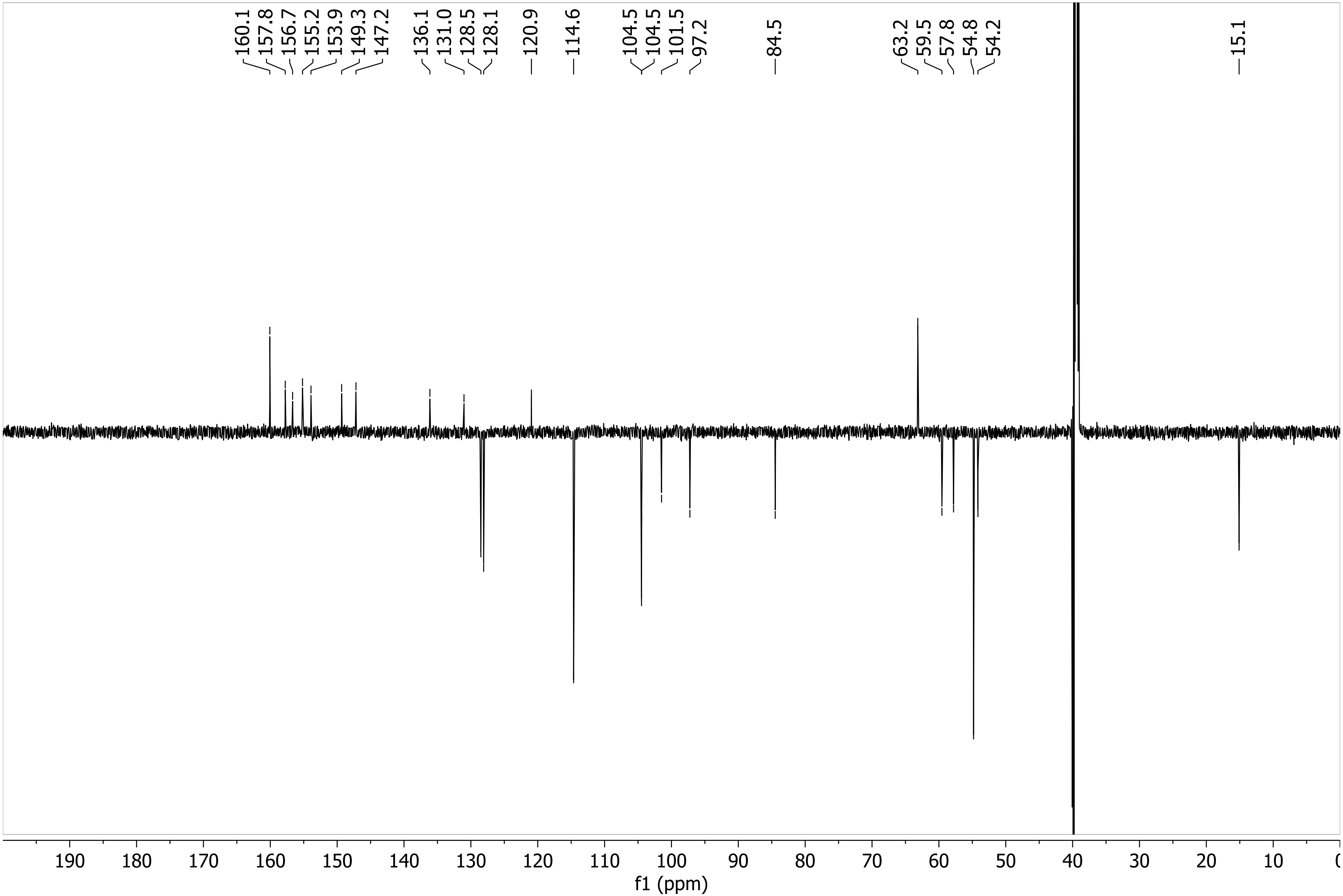

Edited-HSQC NMR spectrum of compound **72** in DMSO-d_6

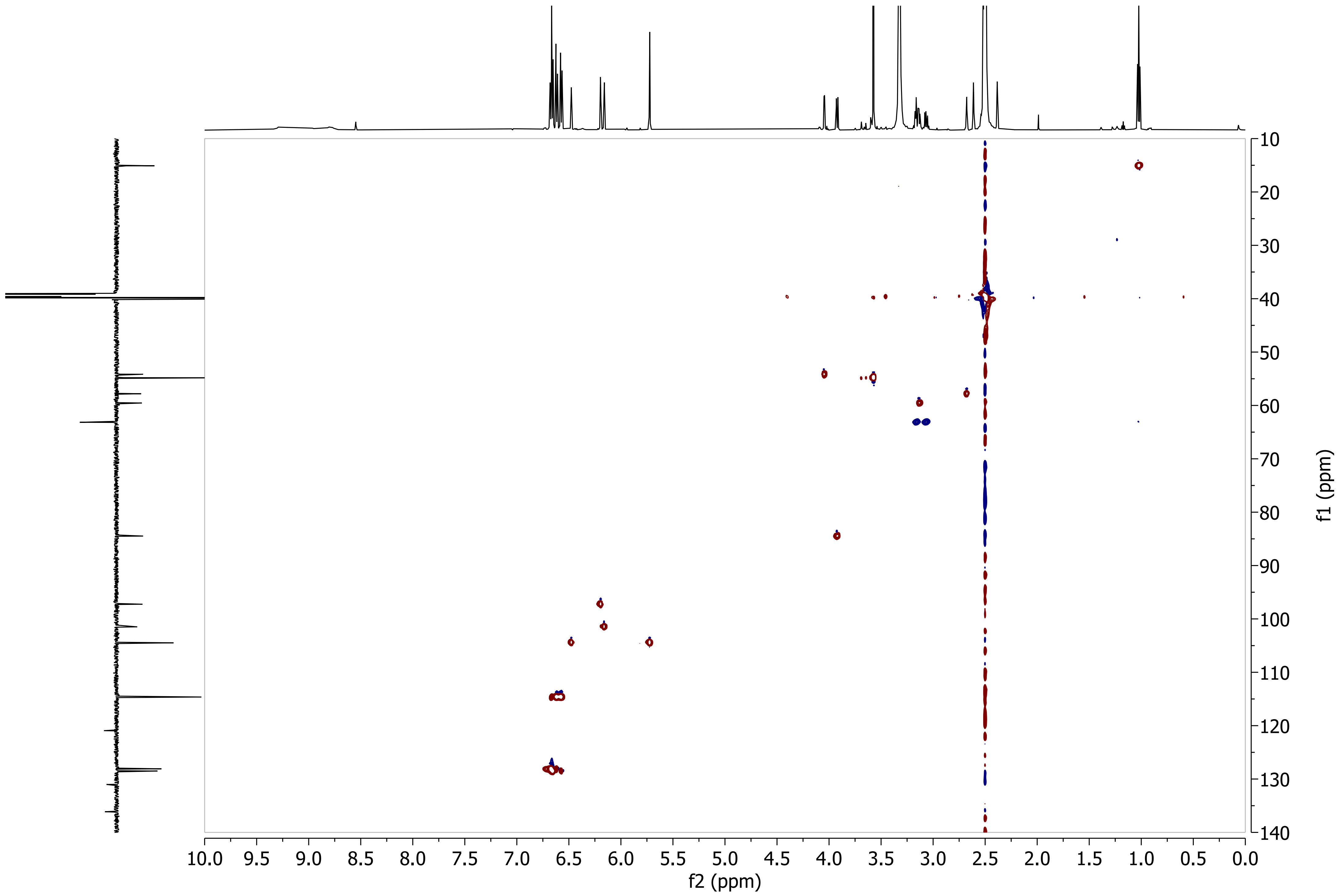

HMBC NMR spectrum of compound **72** in DMSO-d_6

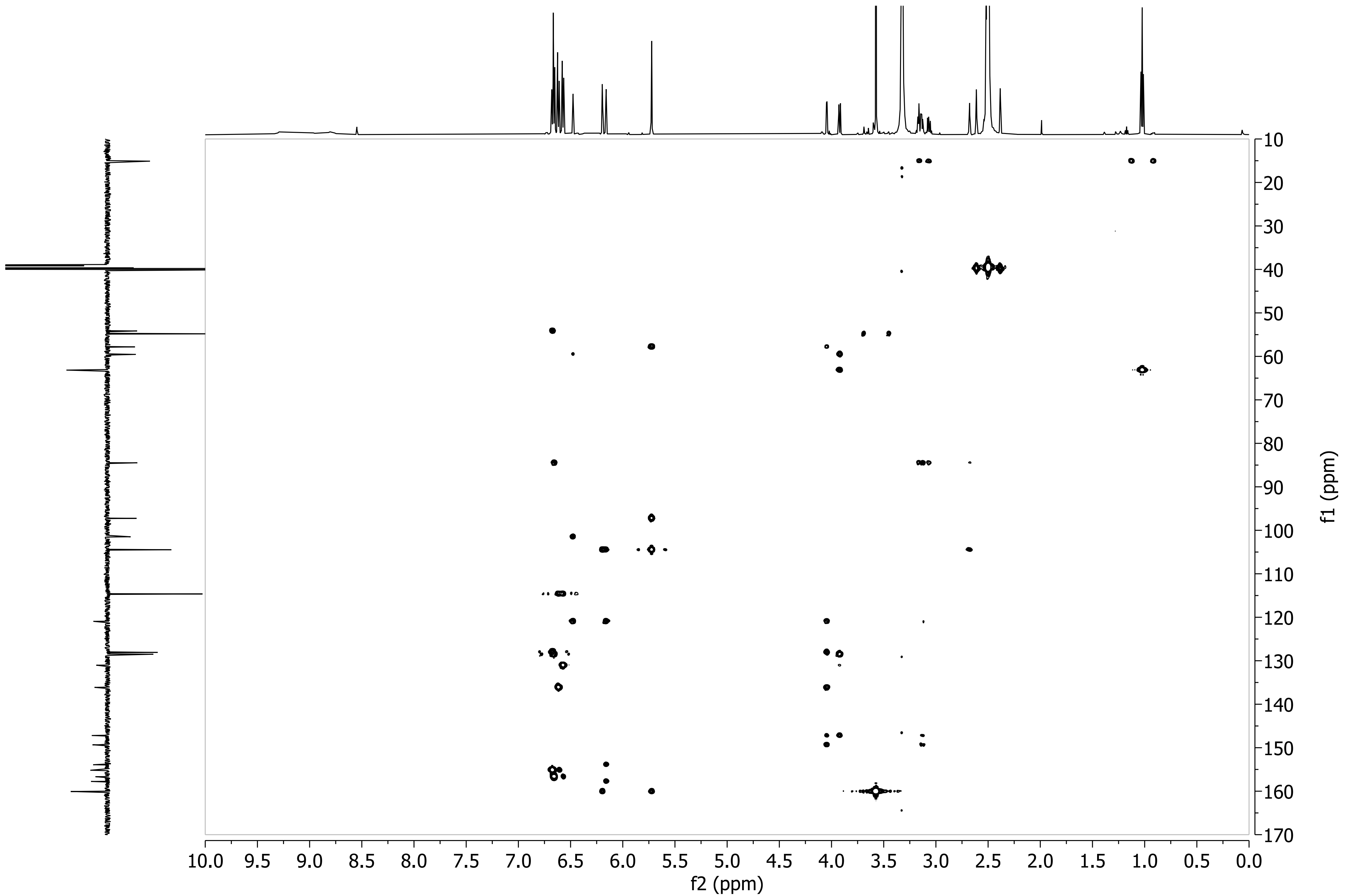

ROESY NMR spectrum of compound **72** in DMSO-d_6

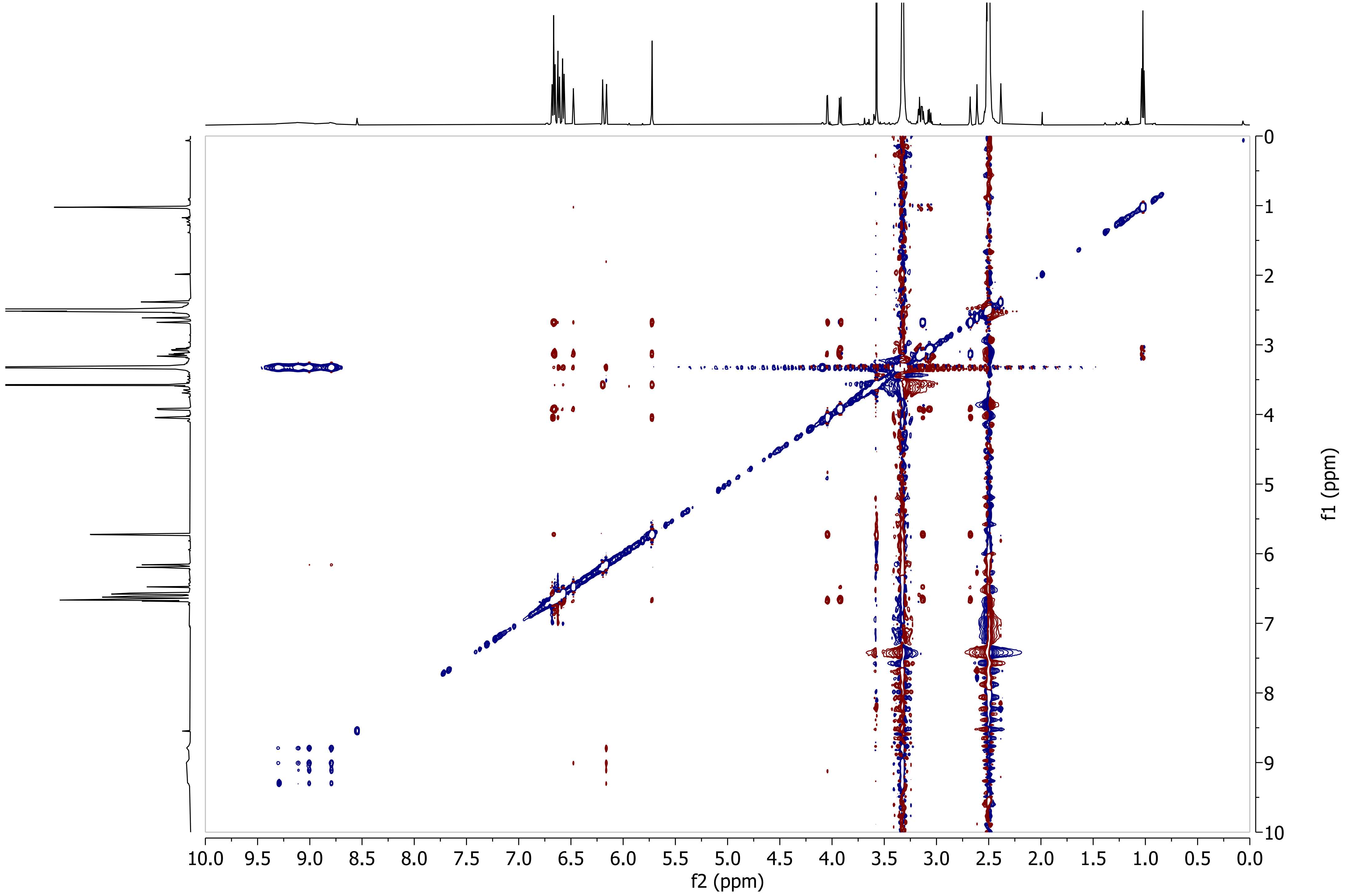

^{1}H NMR spectrum of compound **73** in DMSO-d_6

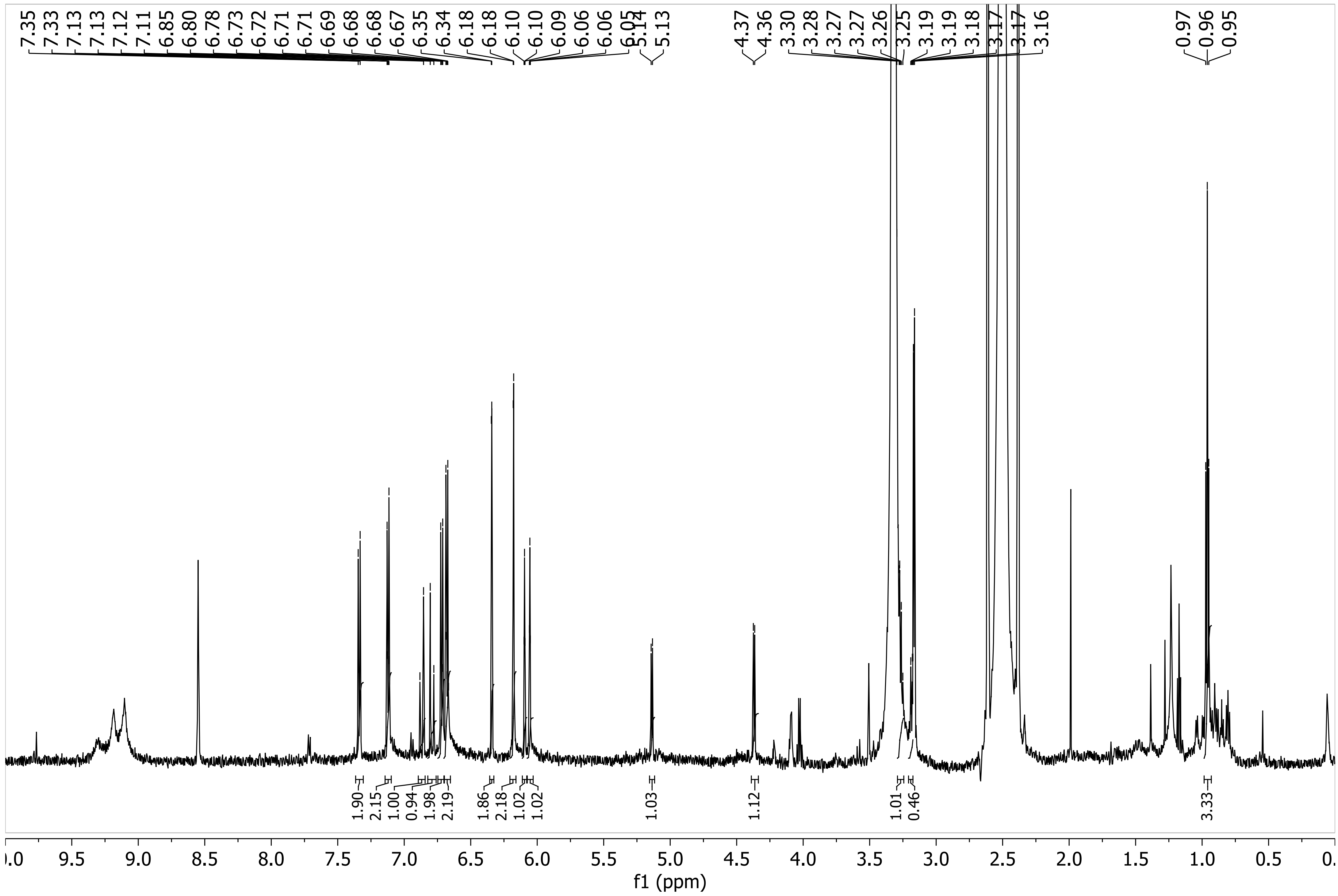

COSY NMR spectrum of compound **73** in DMSO-d_6

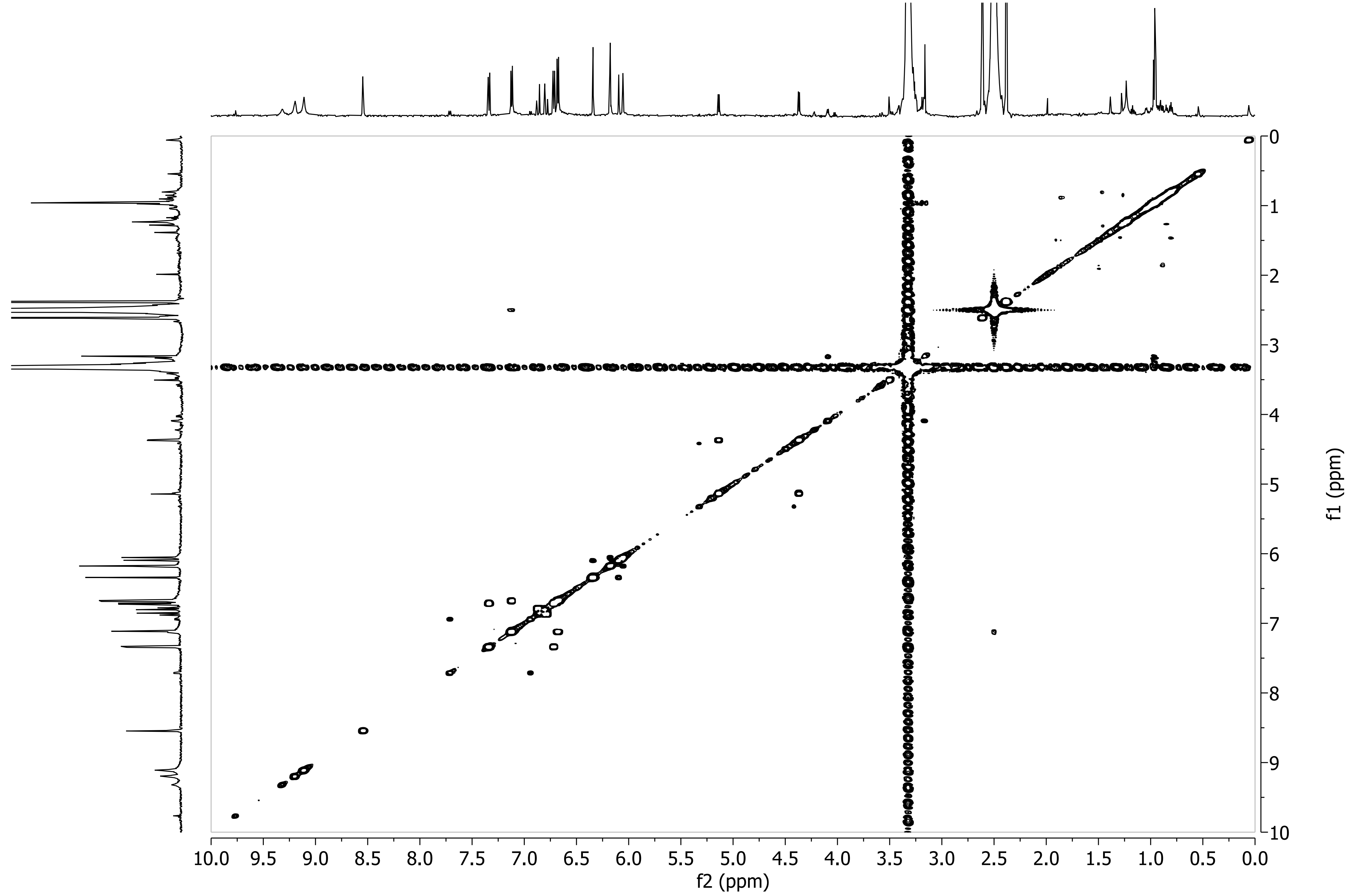

Edited-HSQC NMR spectrum of compound **73** in DMSO-d_6

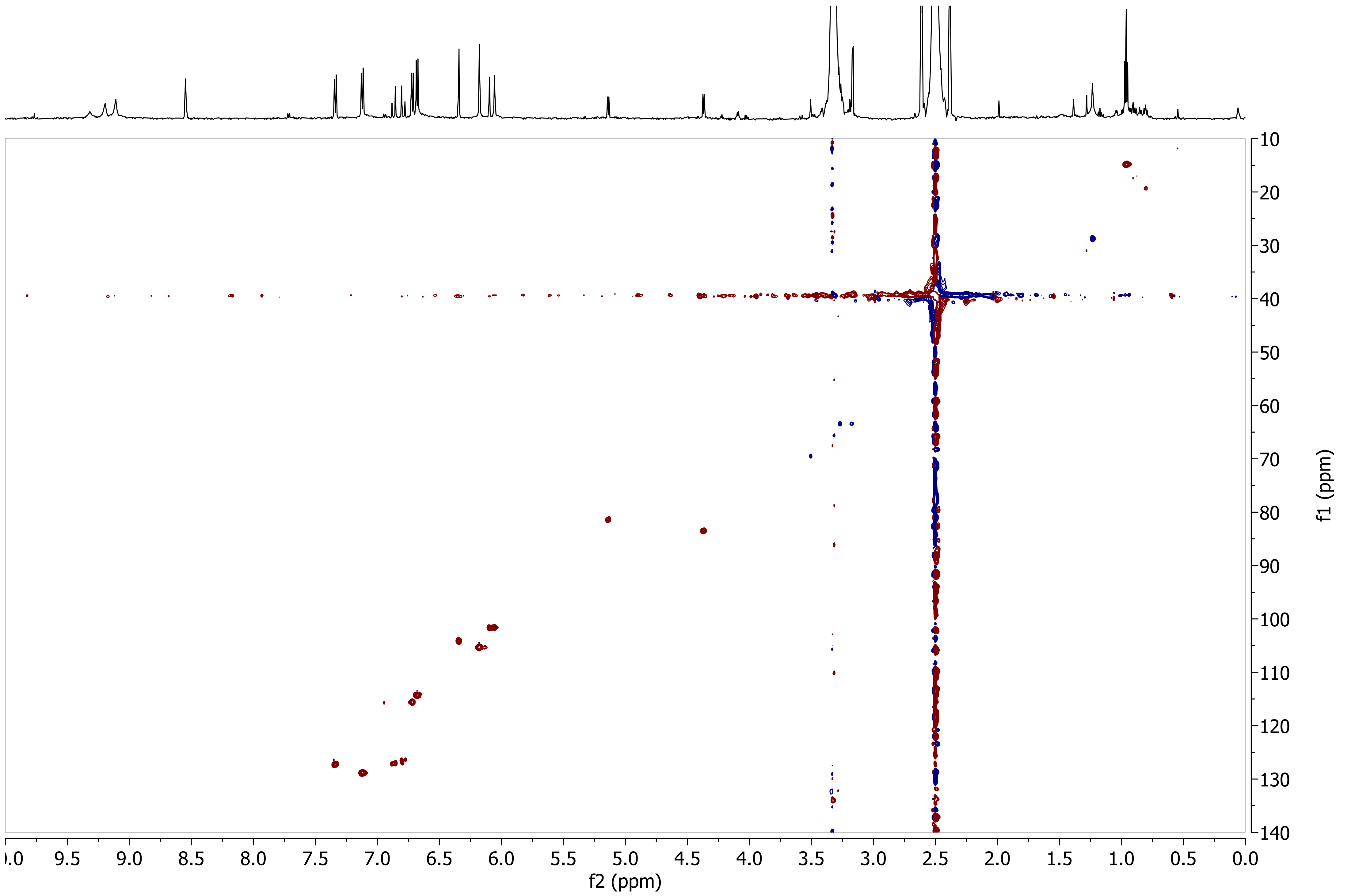

HMBC NMR spectrum of compound **73** in DMSO-d_6

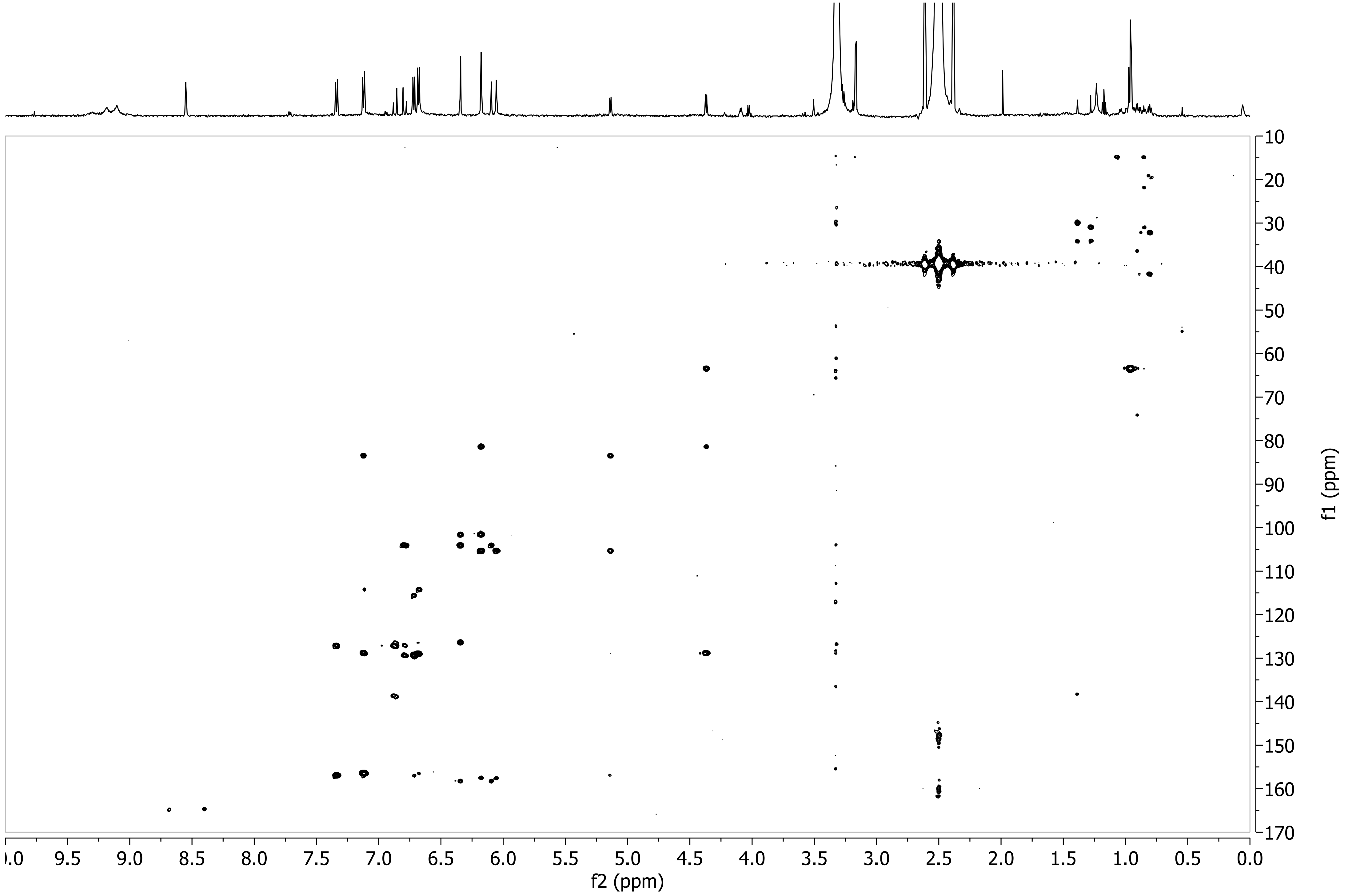

ROESY NMR spectrum of compound **73** in DMSO-d_6

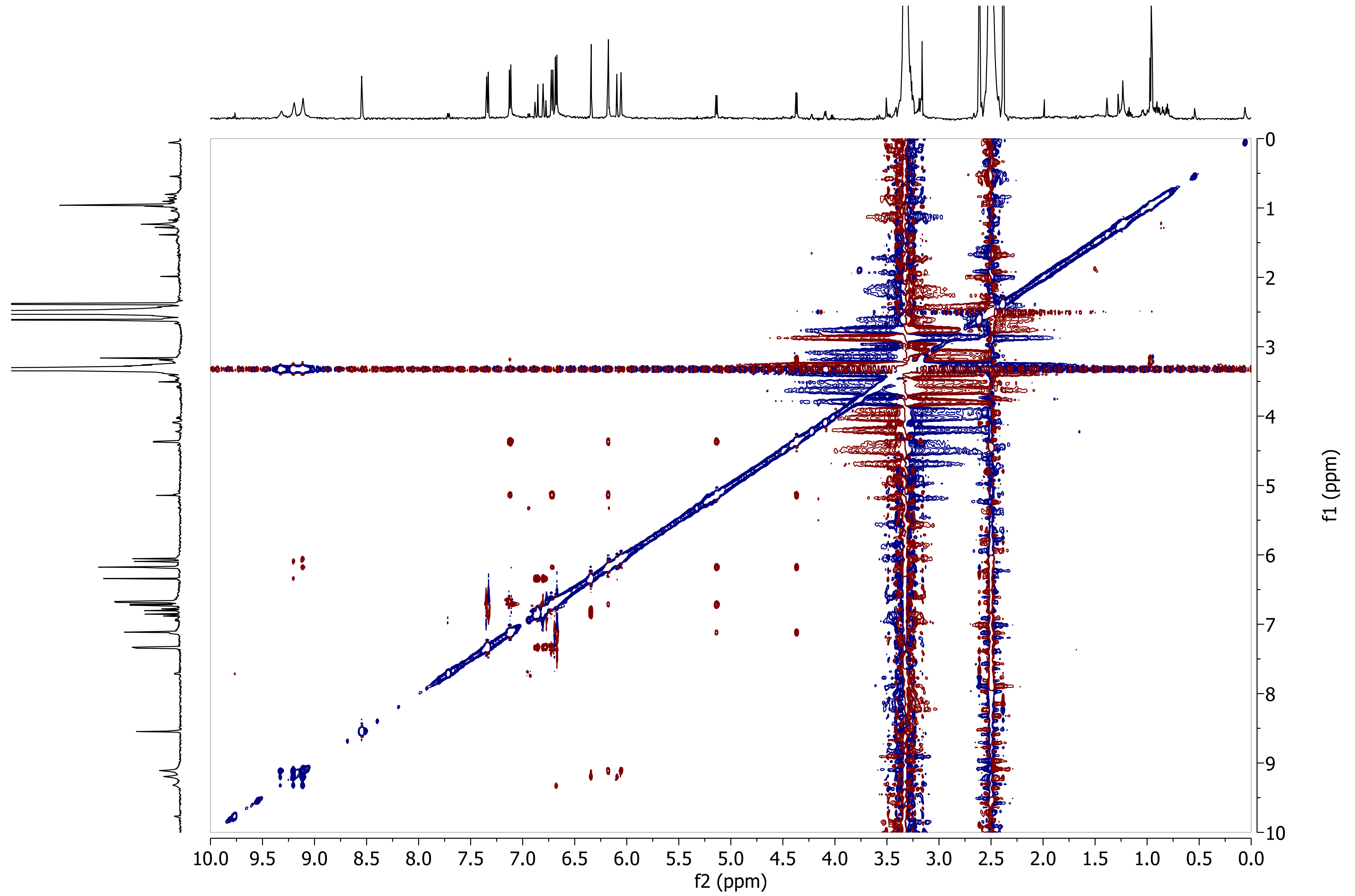

Supplement: Supplementary file 2 [file DataSheet4.pdf]
