# Supplementary material for: Chemoenzymatic Synthesis of Original Stilbene Dimers Possessing Wnt Inhibition Activity in Triple-Negative Breast Cancer Cells Using the Enzymatic Secretome of Botrytis cinerea Pers
Source: Front Chem. 2022 Apr 19;10:881298. doi: 10.3389/fchem.2022.881298 (PMC9062038; doi:10.3389/fchem.2022.881298)

^{1}H NMR spectrum of compound **26** in DMSO-d_6

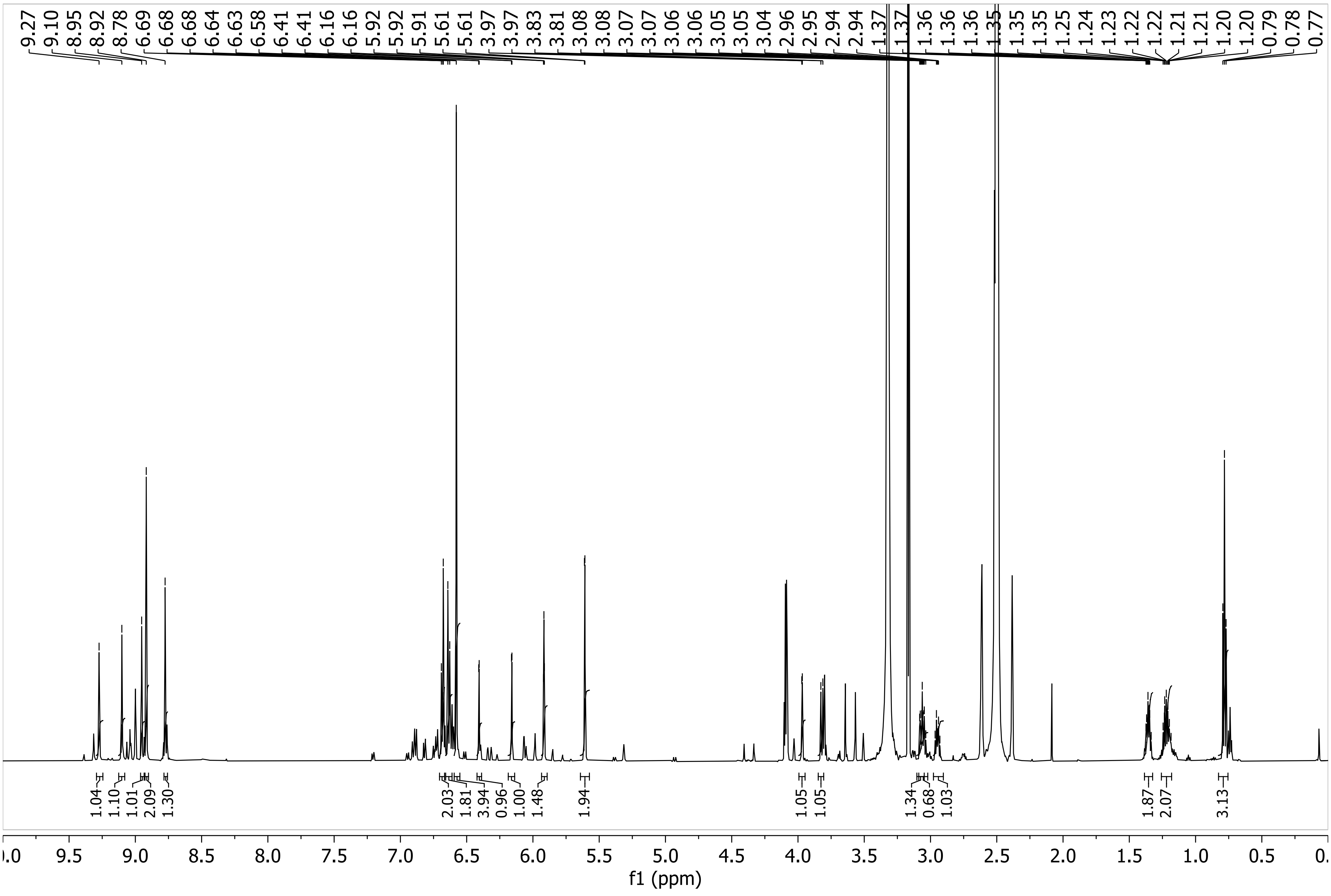

COSY NMR spectrum of compound **26** in DMSO-d_6

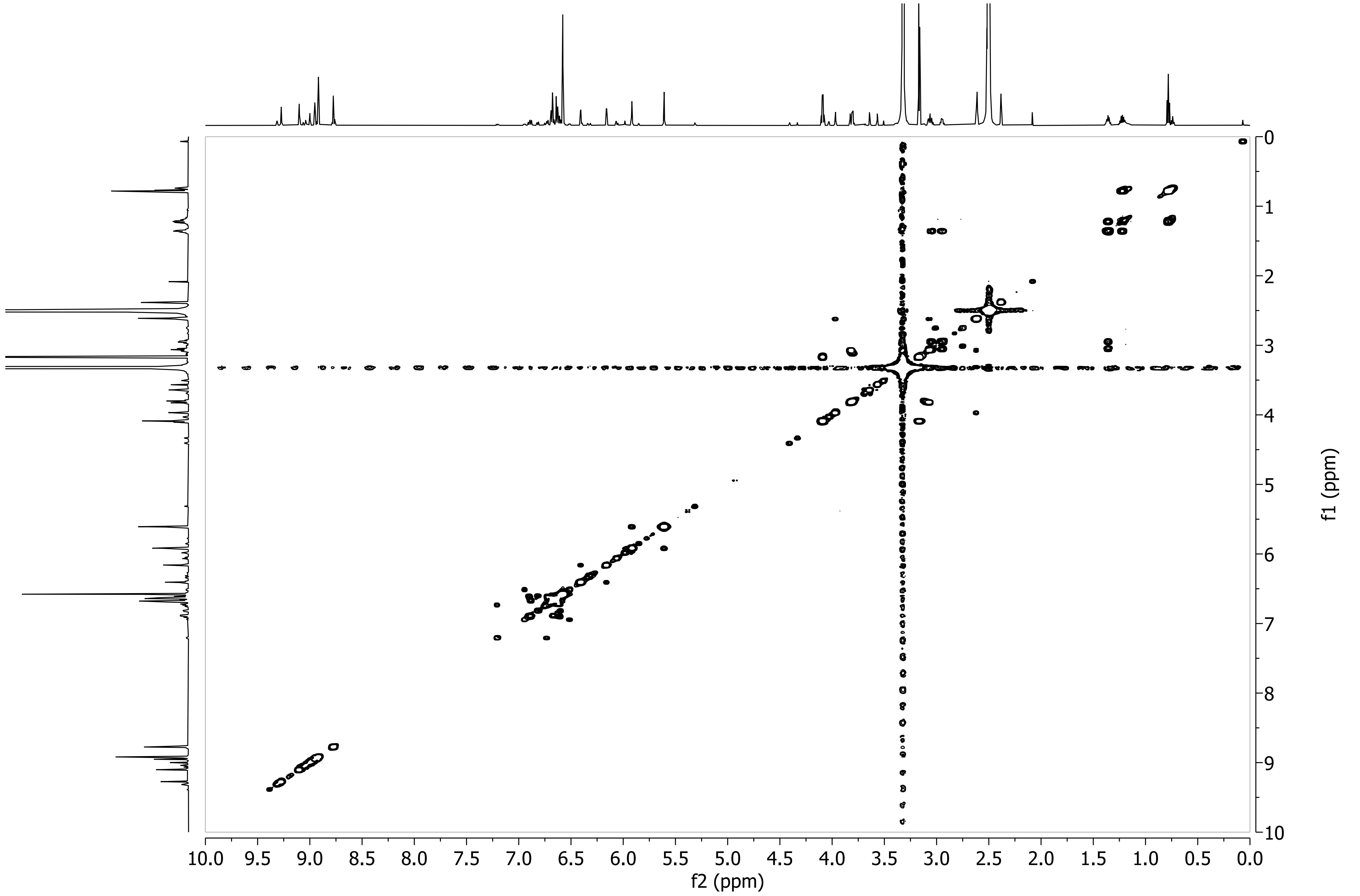

Edited-HSQC NMR spectrum of compound **26** in DMSO-d_6

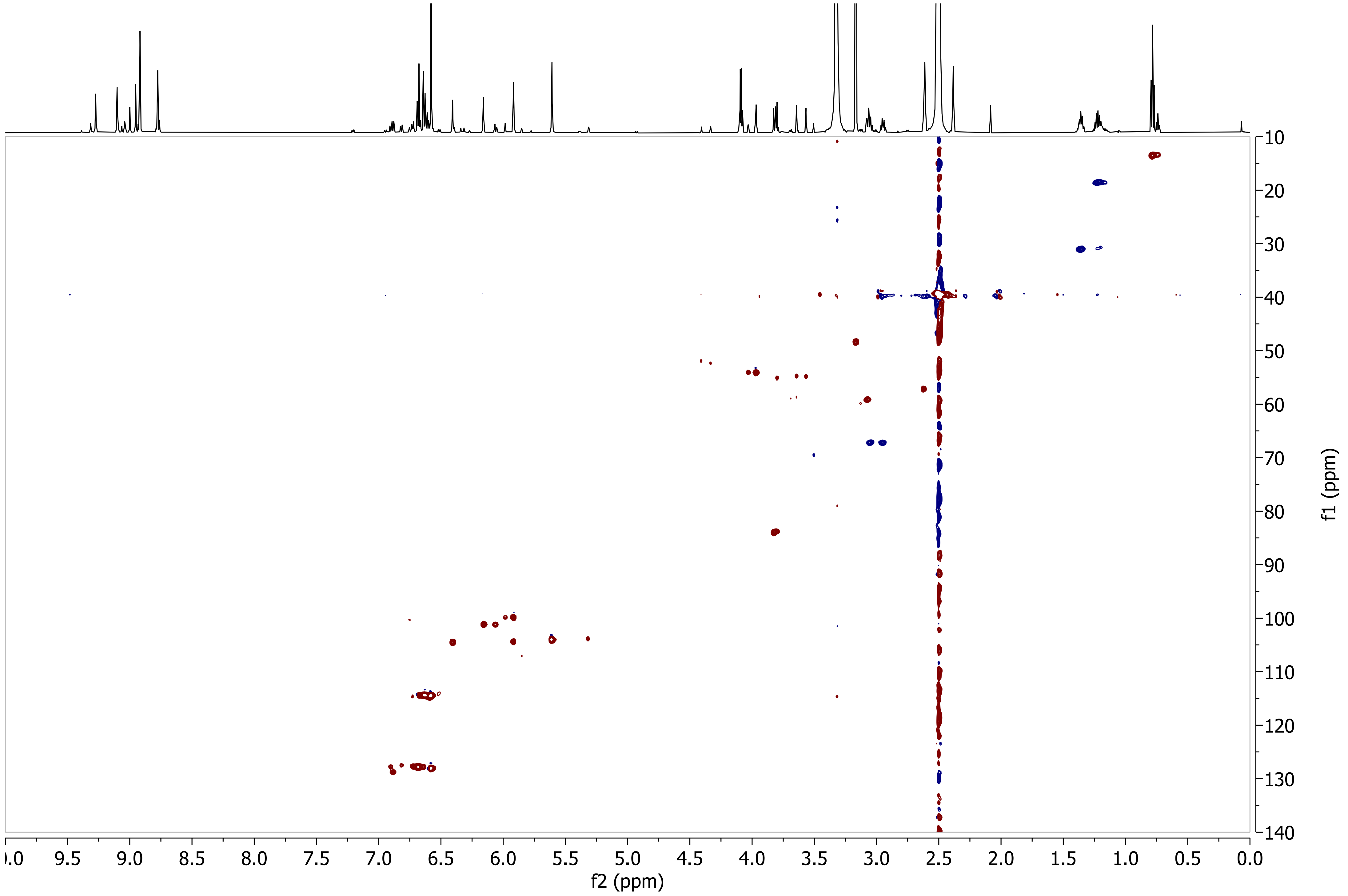

HMBC NMR spectrum of compound **26** in DMSO-d_6

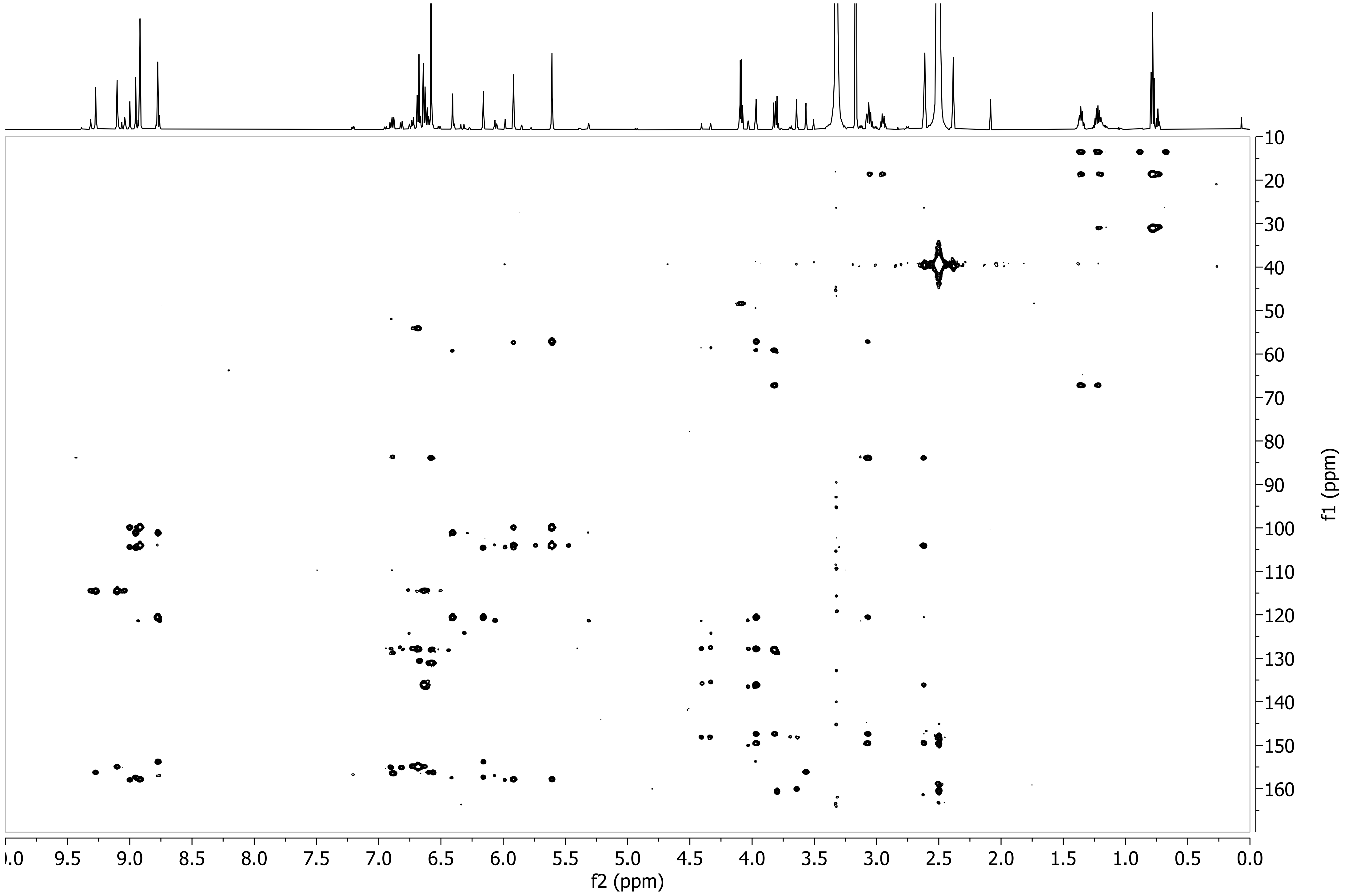

ROESY NMR spectrum of compound **26** in DMSO-d_6

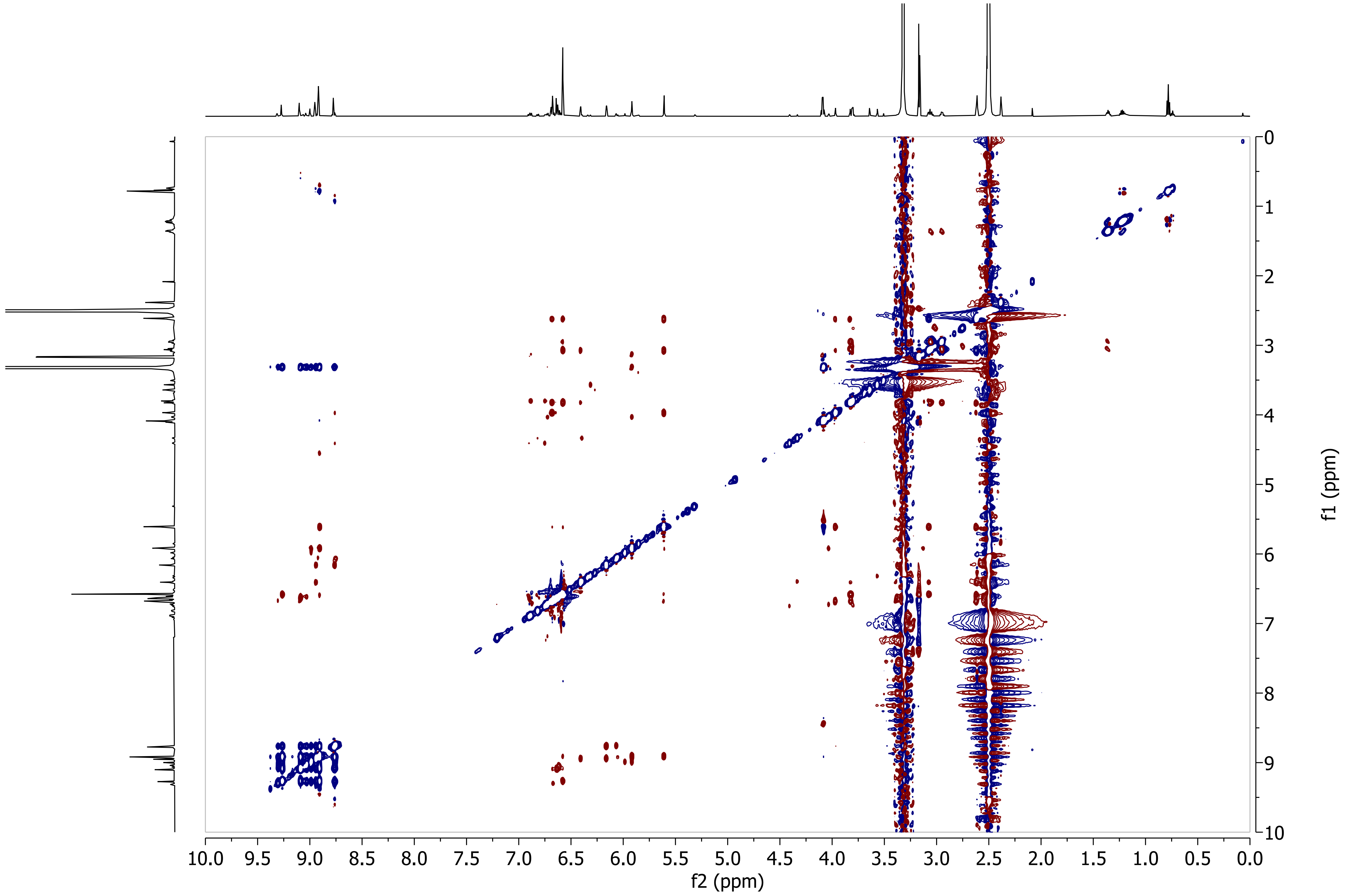

^{1}H NMR spectrum of compound **27** in DMSO-d_6

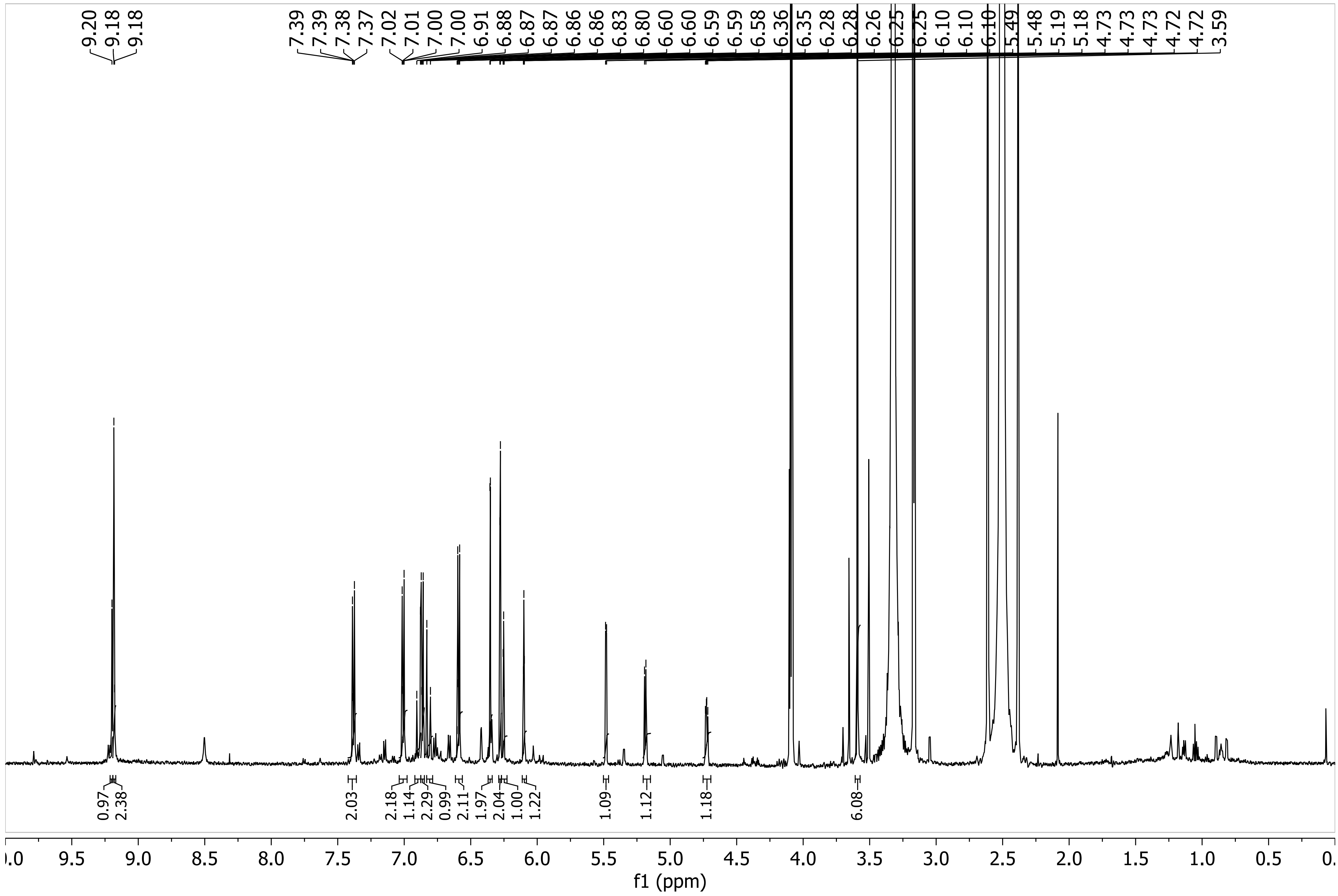

COSY NMR spectrum of compound **27** in DMSO-d_6

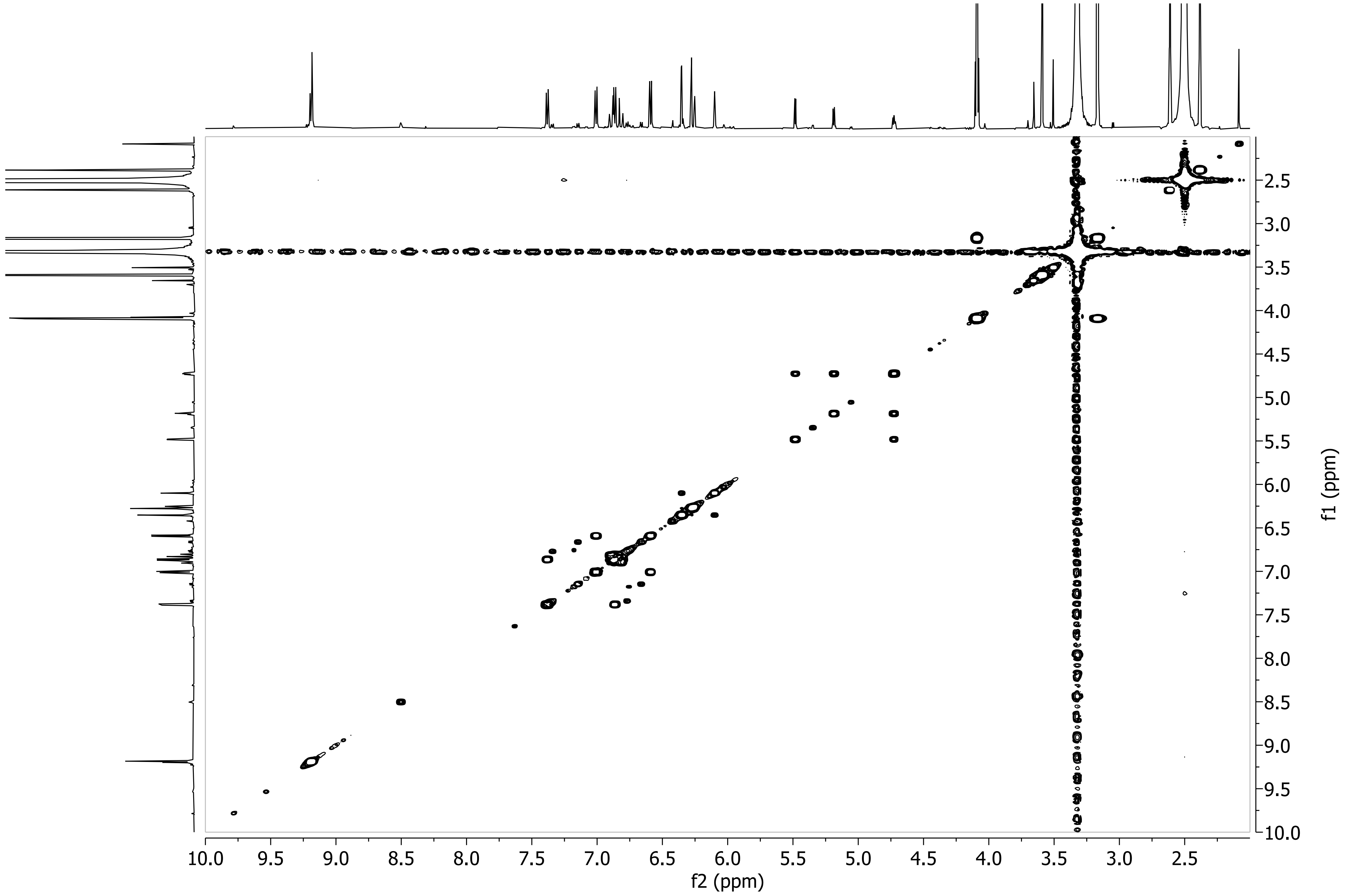

Edited-HSQC NMR spectrum of compound **27** in DMSO-d_6

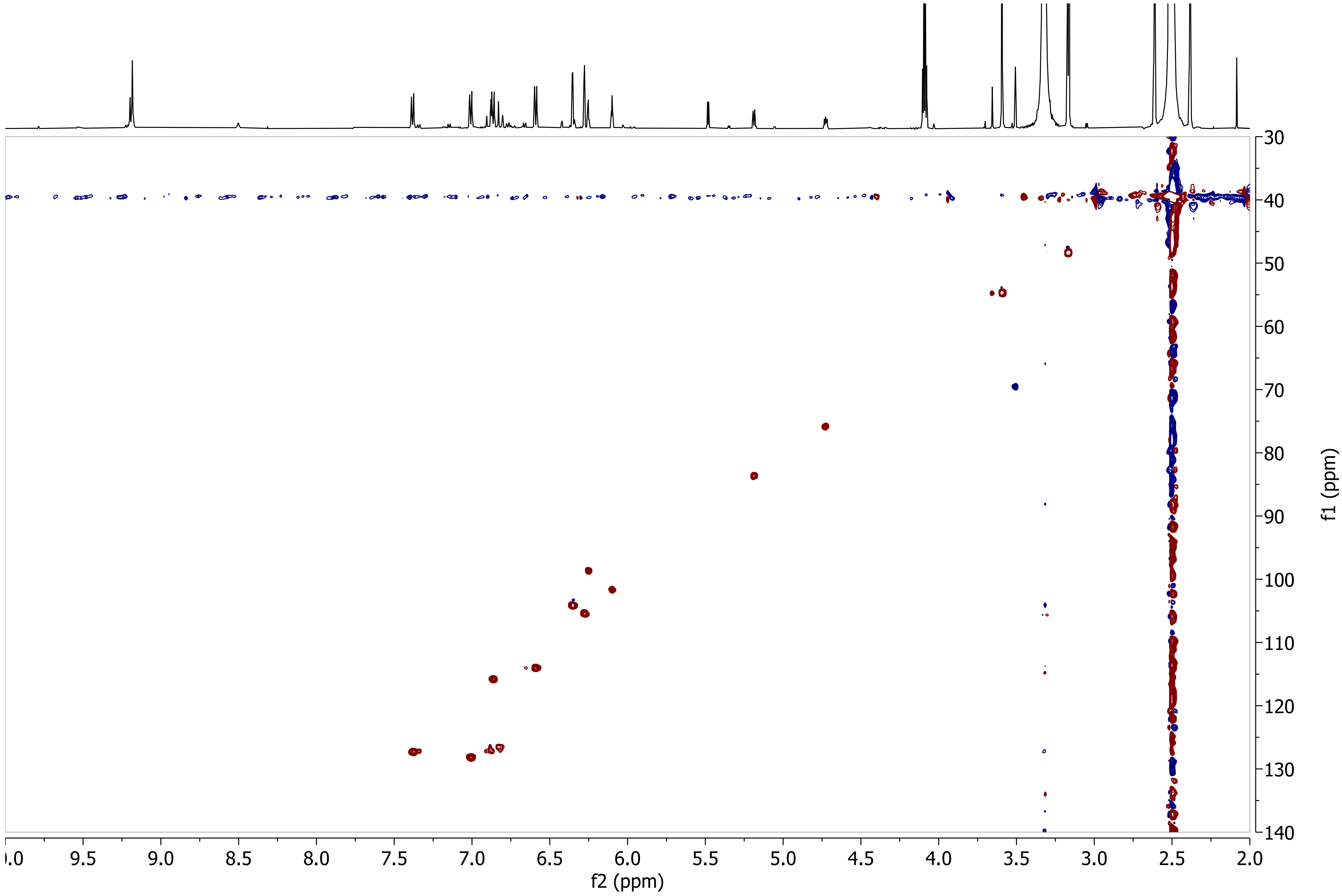

HMBC NMR spectrum of compound **27** in DMSO-d_6

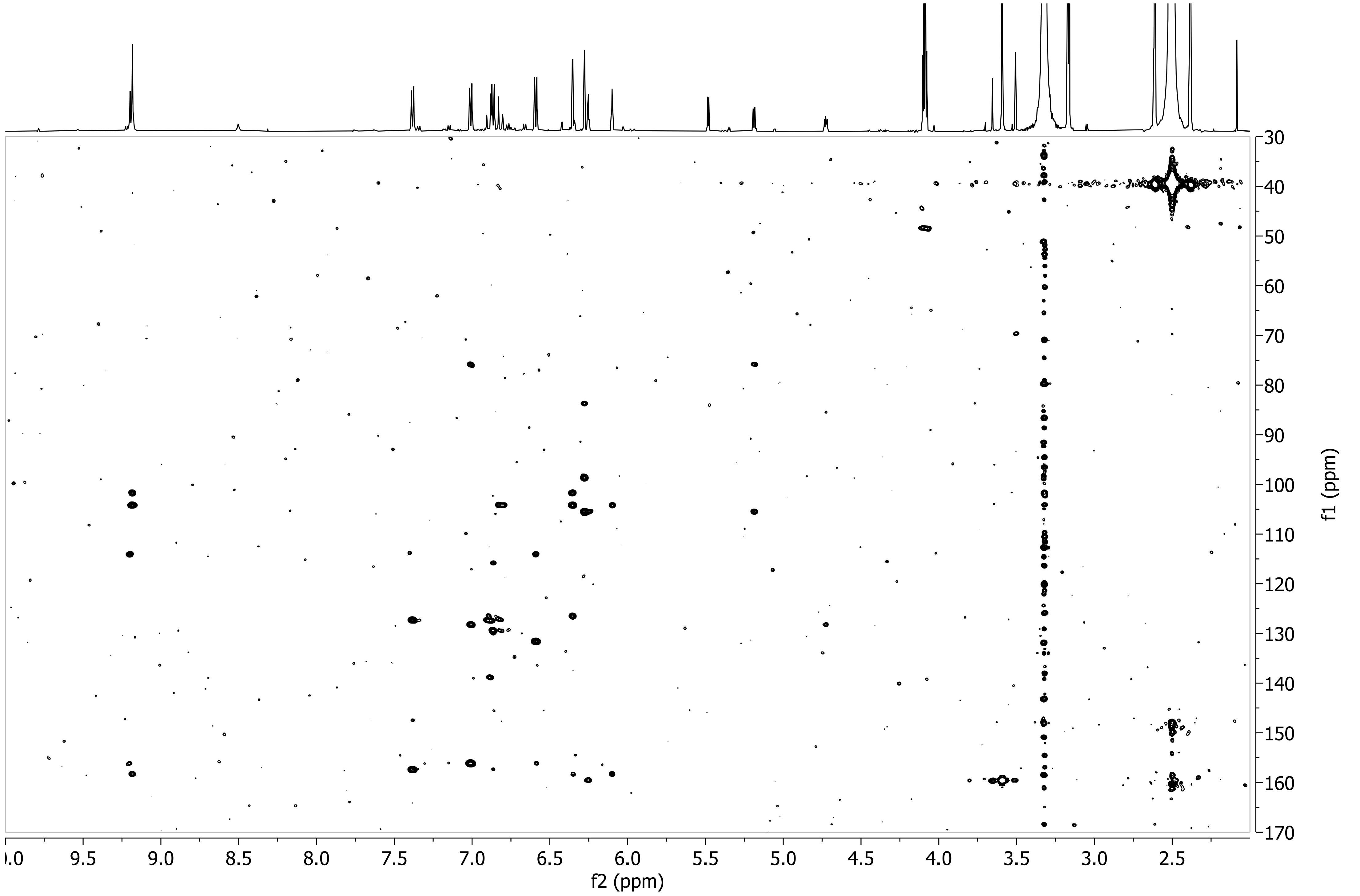

ROESY NMR spectrum of compound **27** in DMSO-d_6

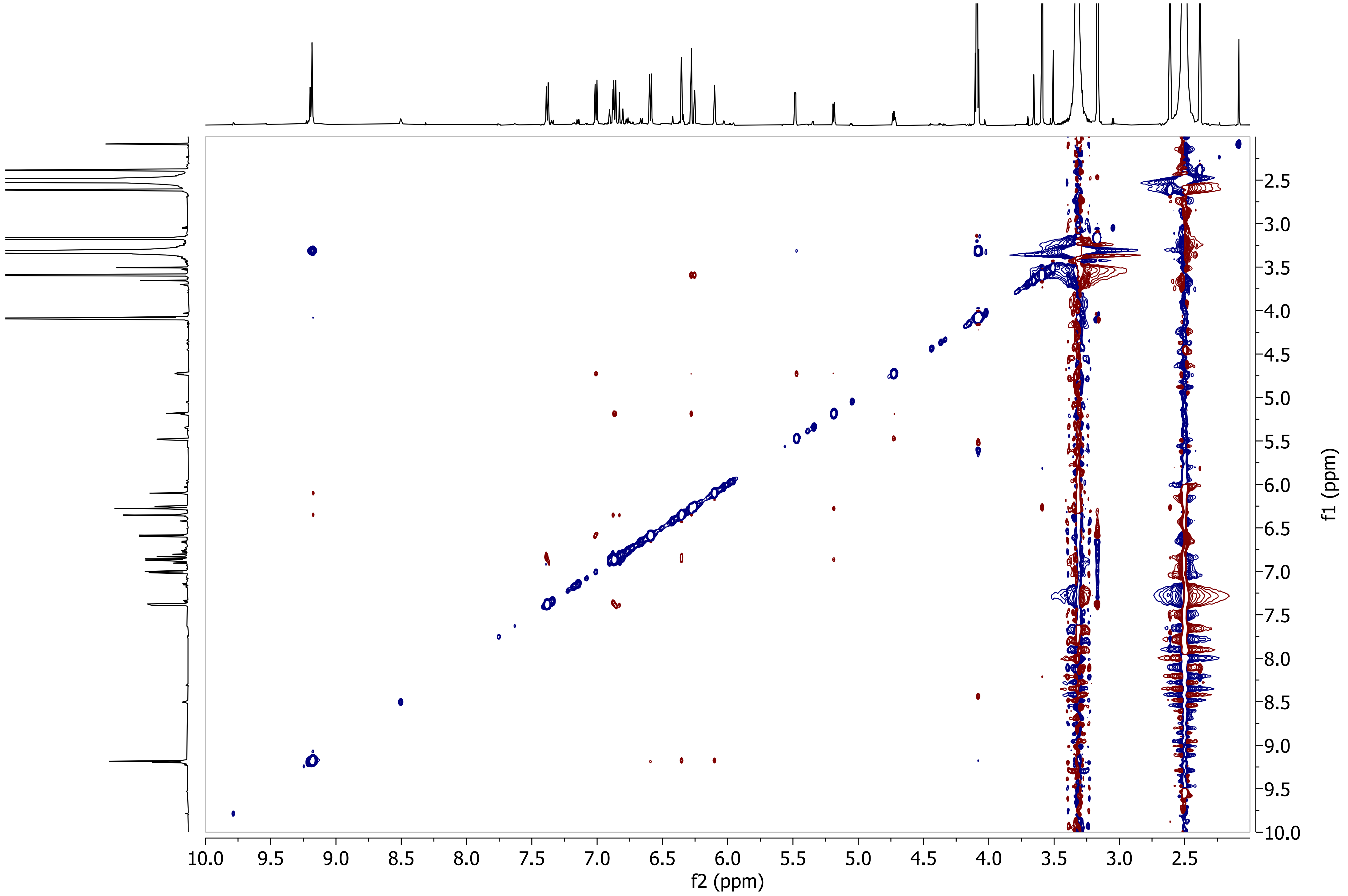

^{1}H NMR spectrum of compound **28** in DMSO-d_6

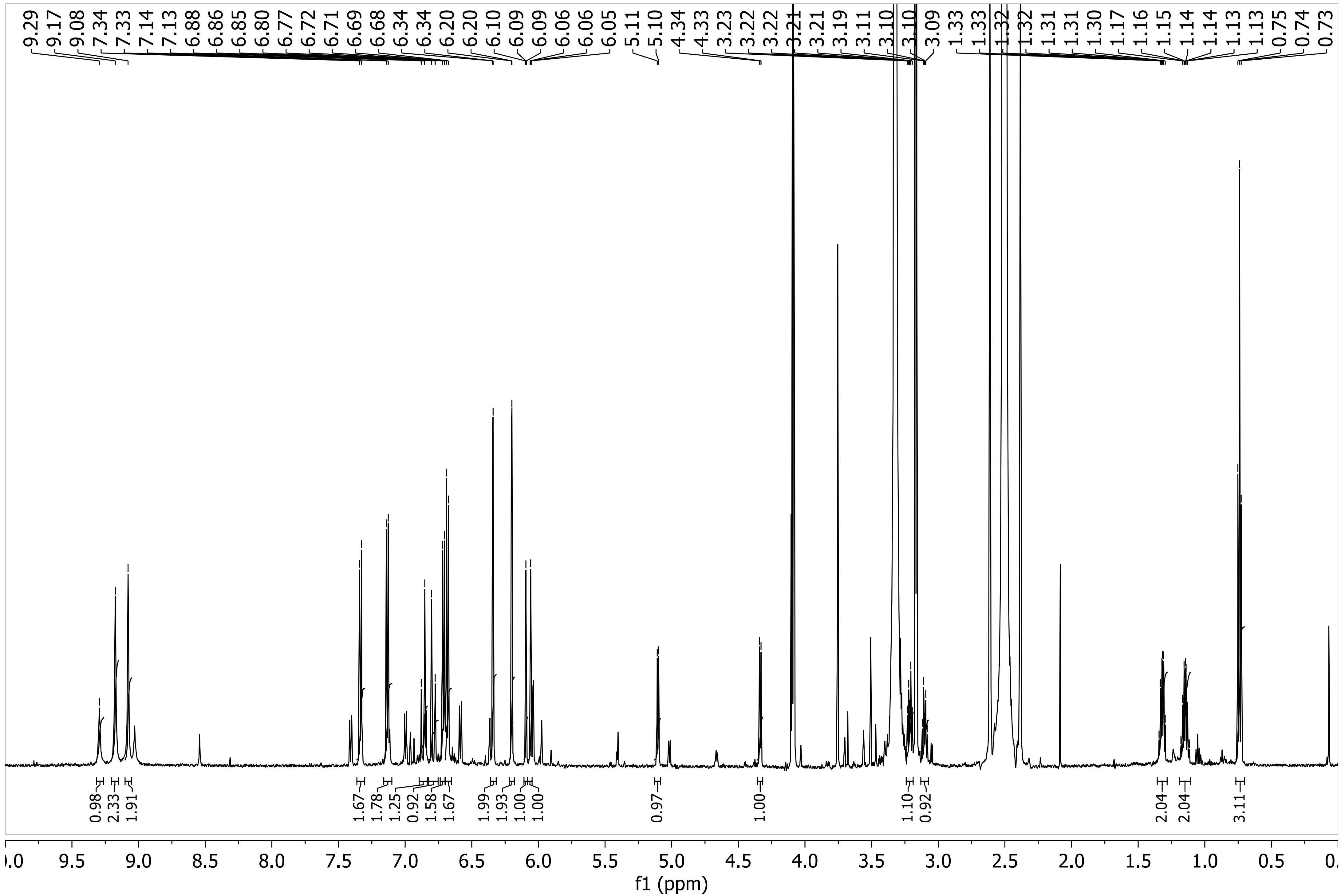

COSY NMR spectrum of compound **28** in DMSO-d_6

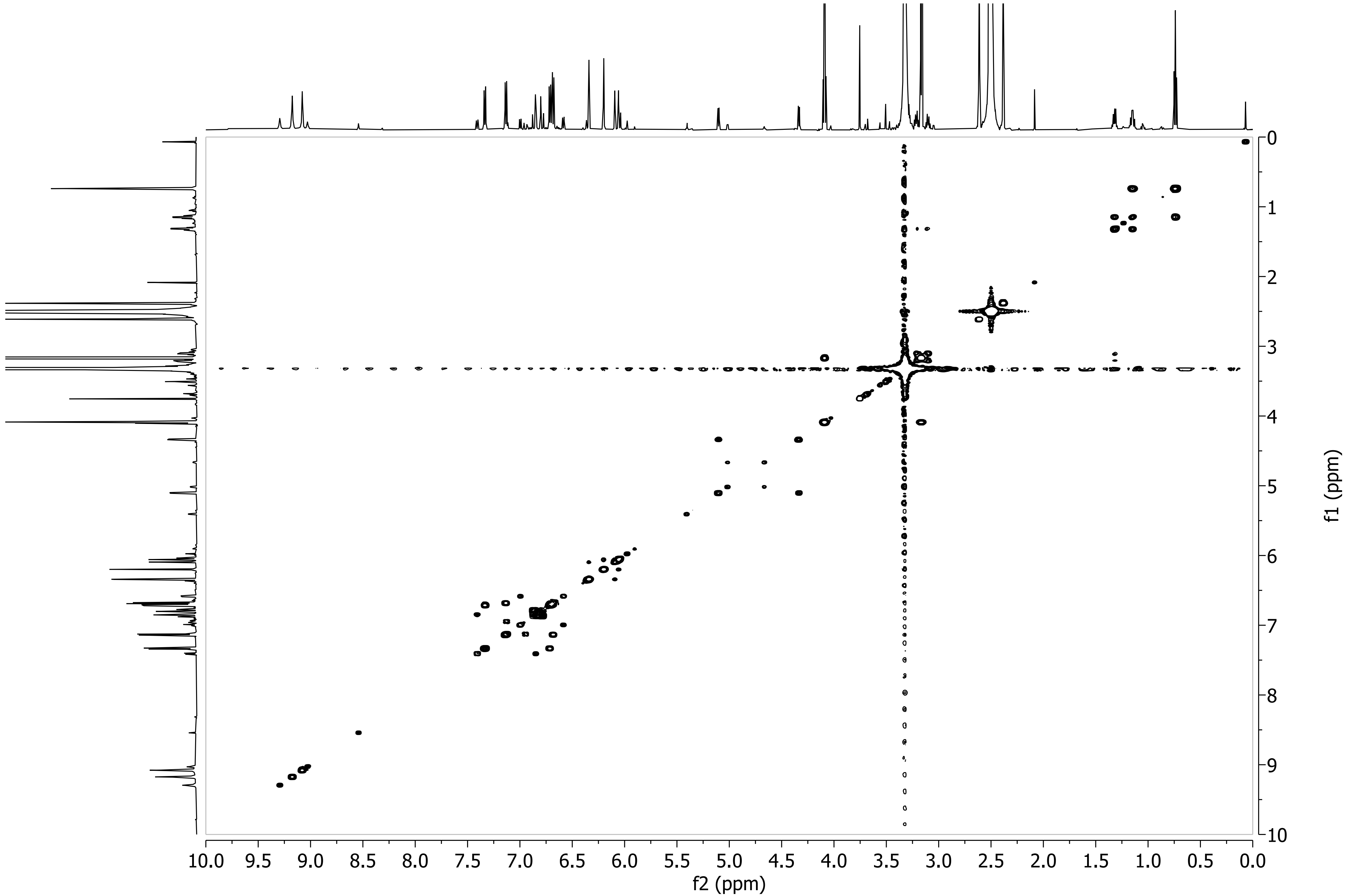

Edited-HSQC NMR spectrum of compound **28** in DMSO-d_6

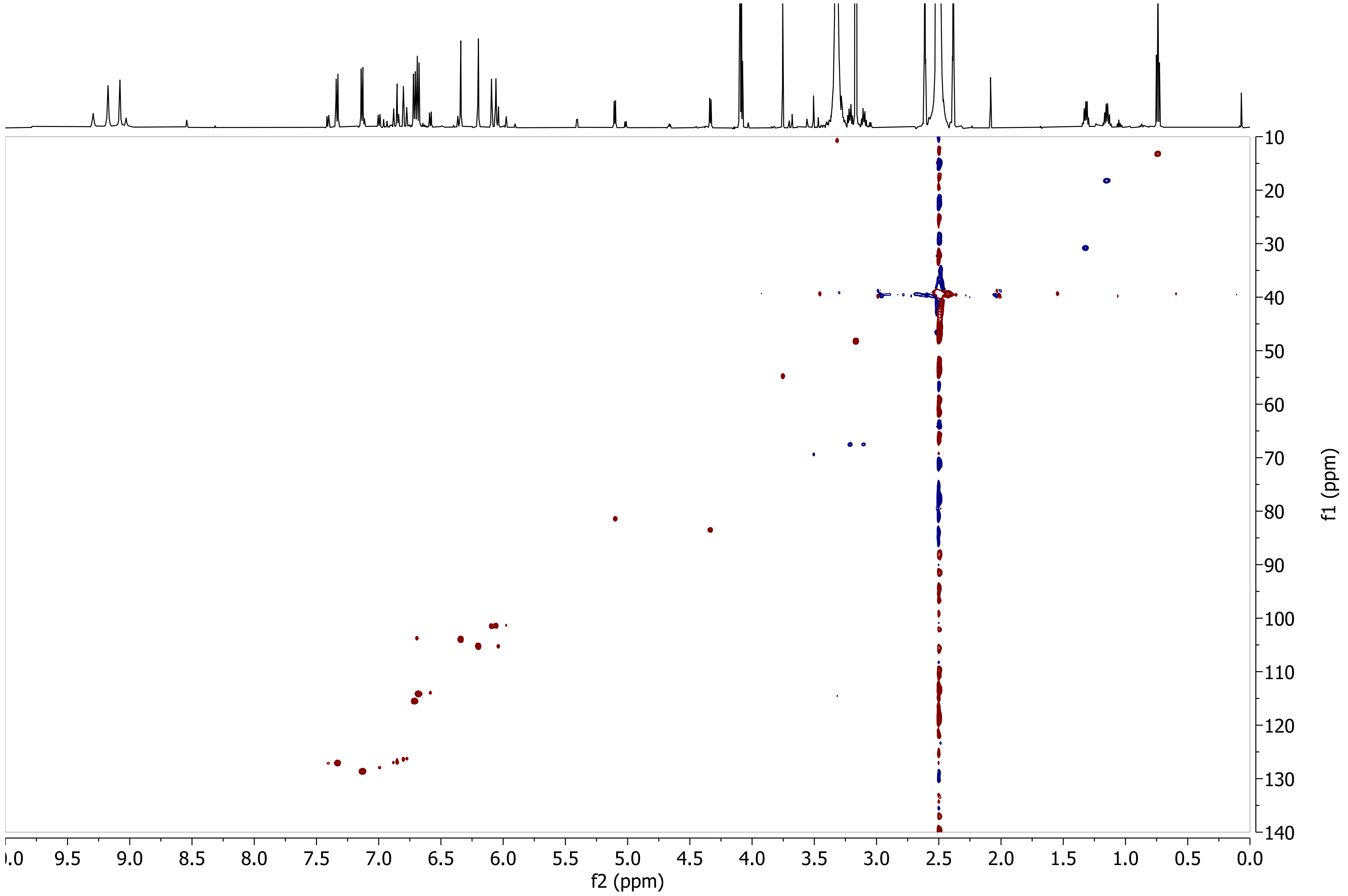

HMBC NMR spectrum of compound **28** in DMSO-d_6

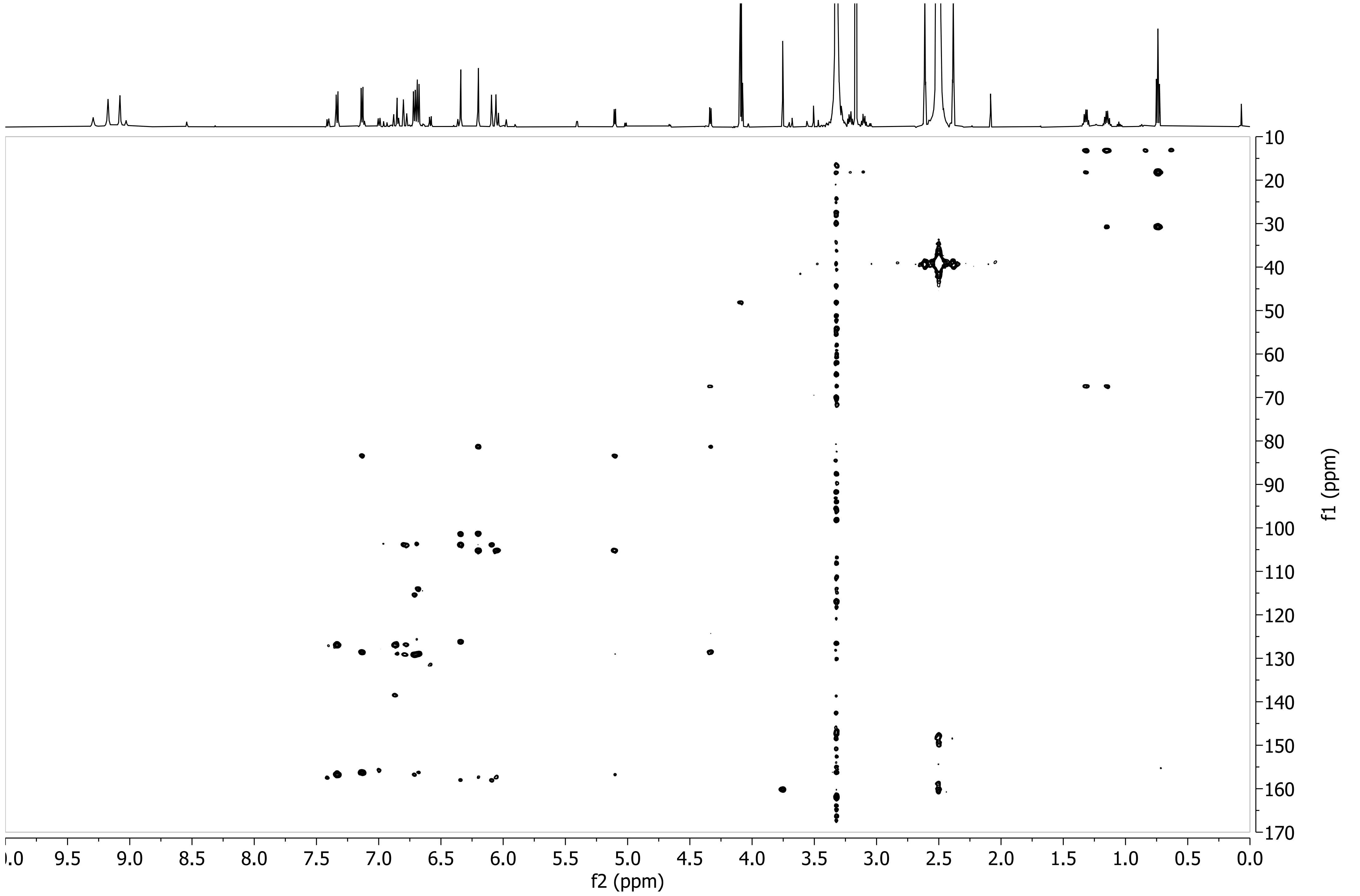

ROESY NMR spectrum of compound **28** in DMSO-d_6

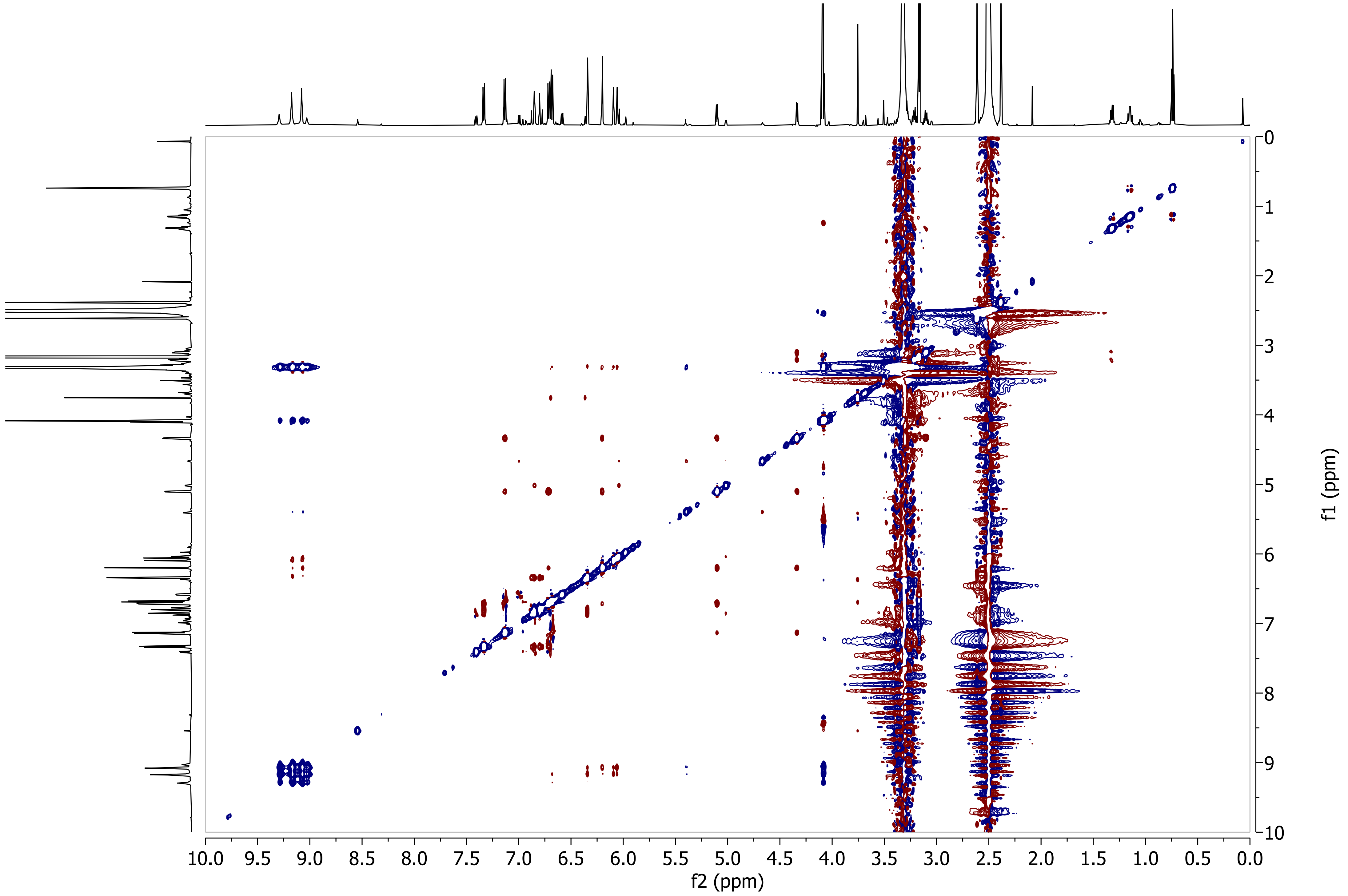

^{1}H NMR spectrum of compound **29** in DMSO-d_6

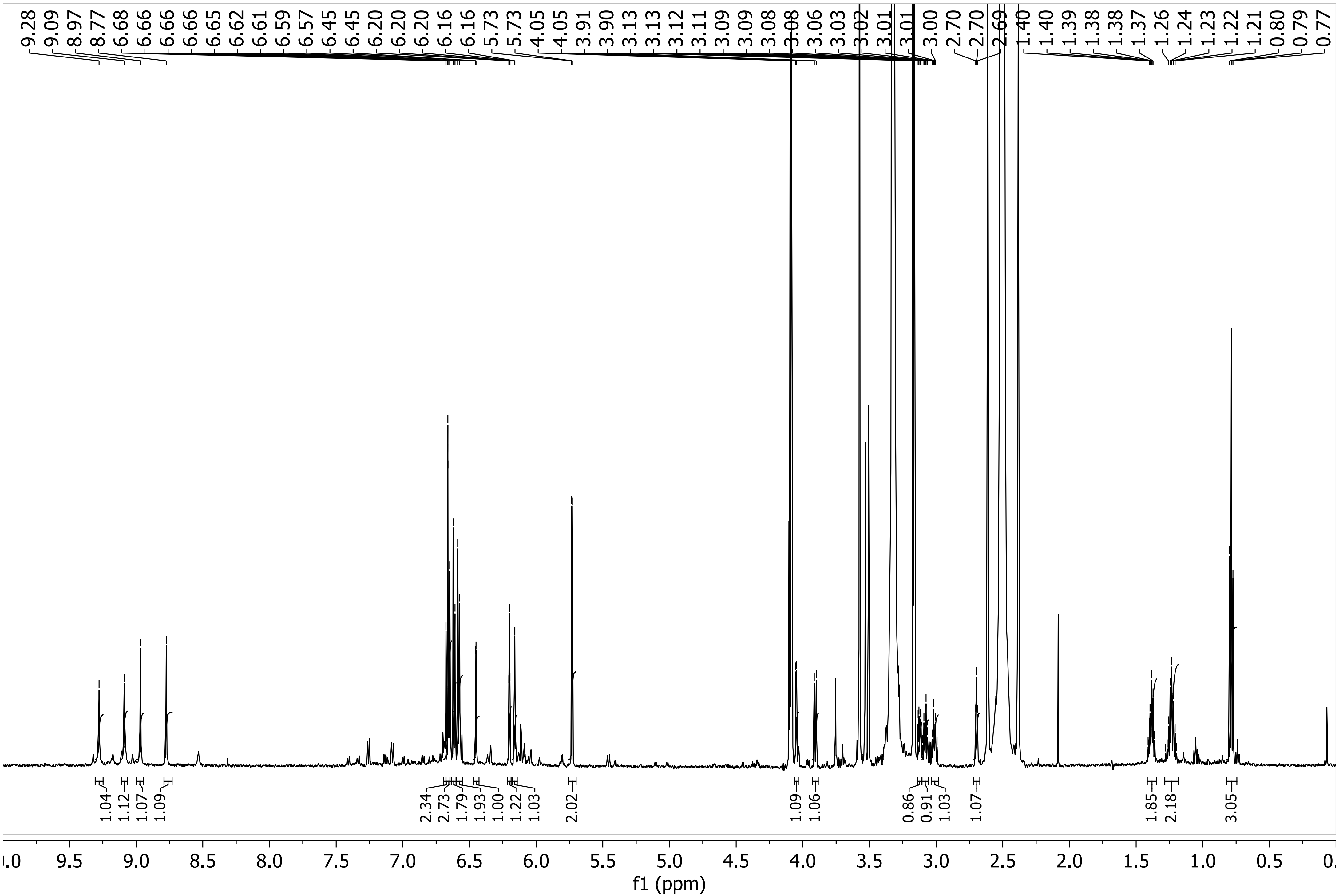

COSY NMR spectrum of compound **29** in DMSO-d_6

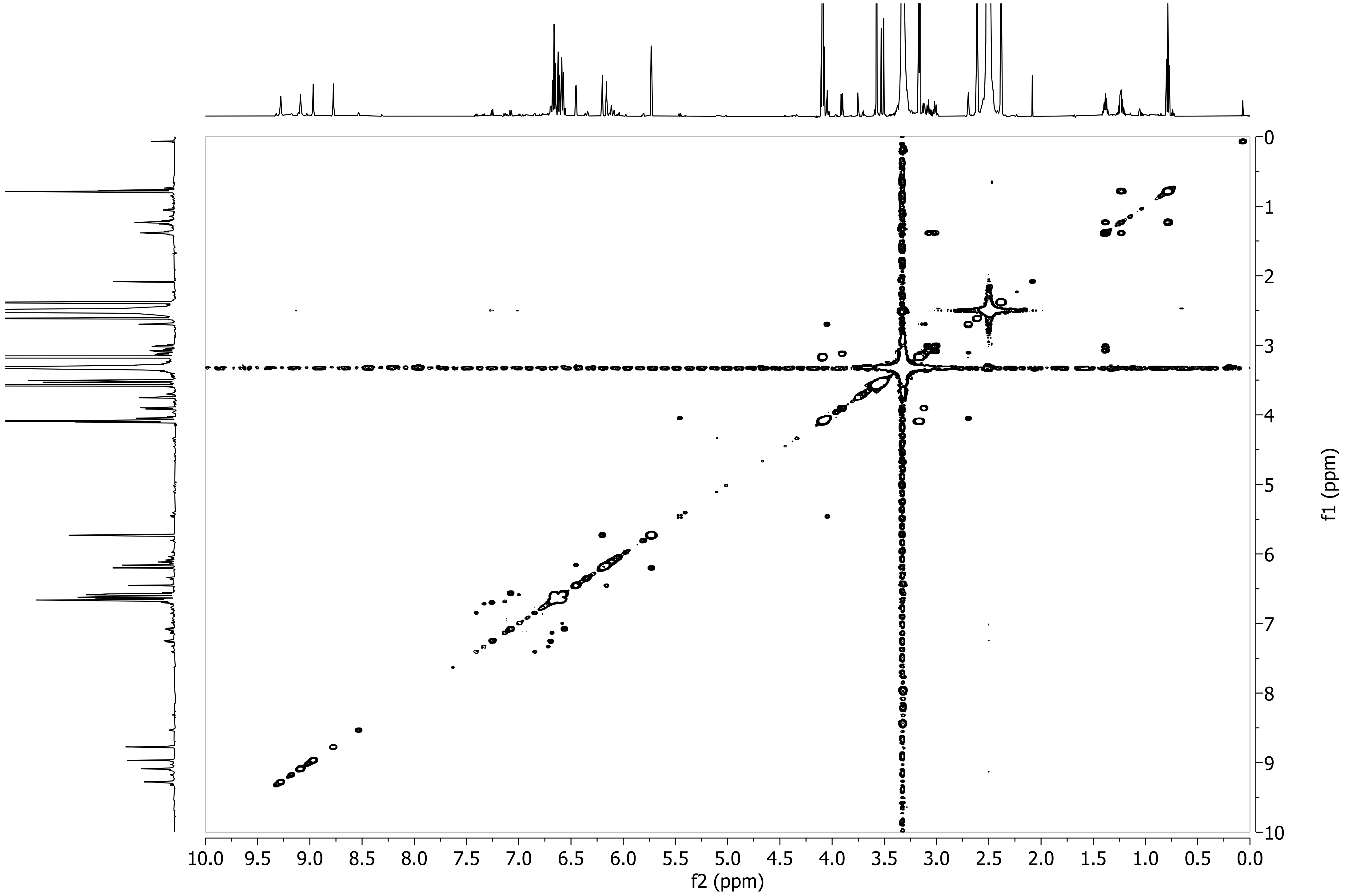

Edited-HSQC NMR spectrum of compound **29** in DMSO-d_6

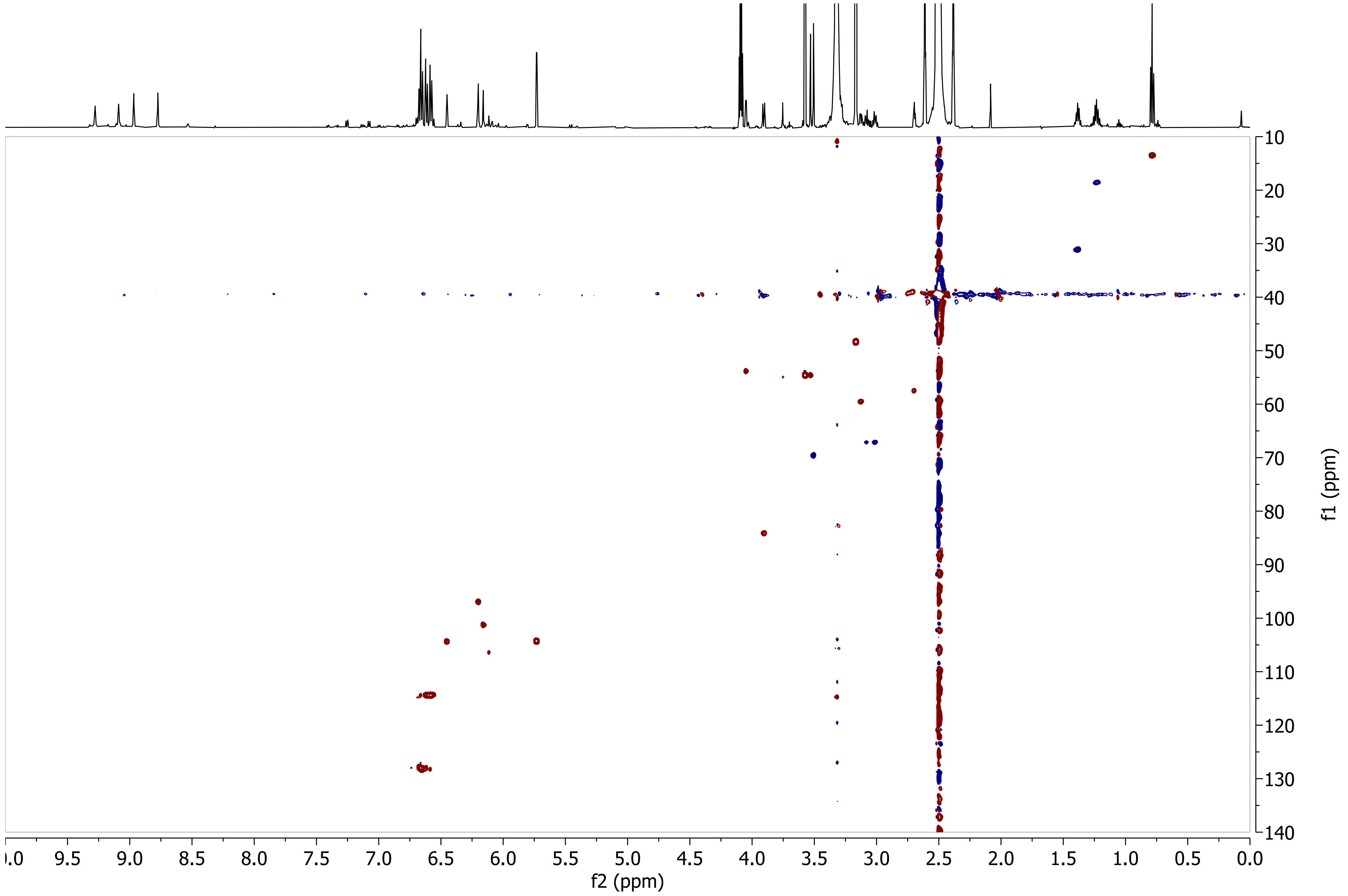

HMBC NMR spectrum of compound **29** in DMSO-d_6

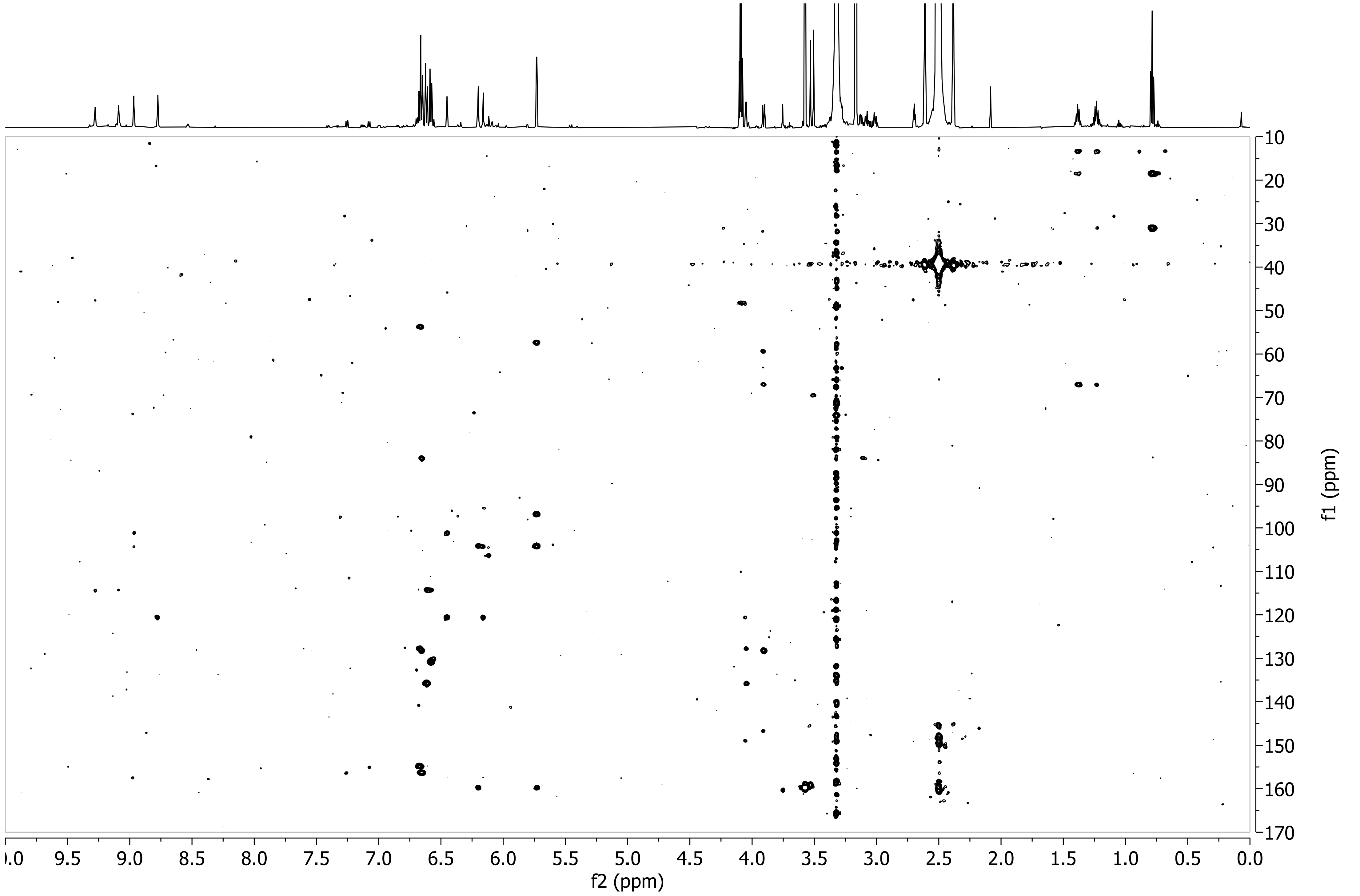

ROESY NMR spectrum of compound **29** in DMSO-d_6

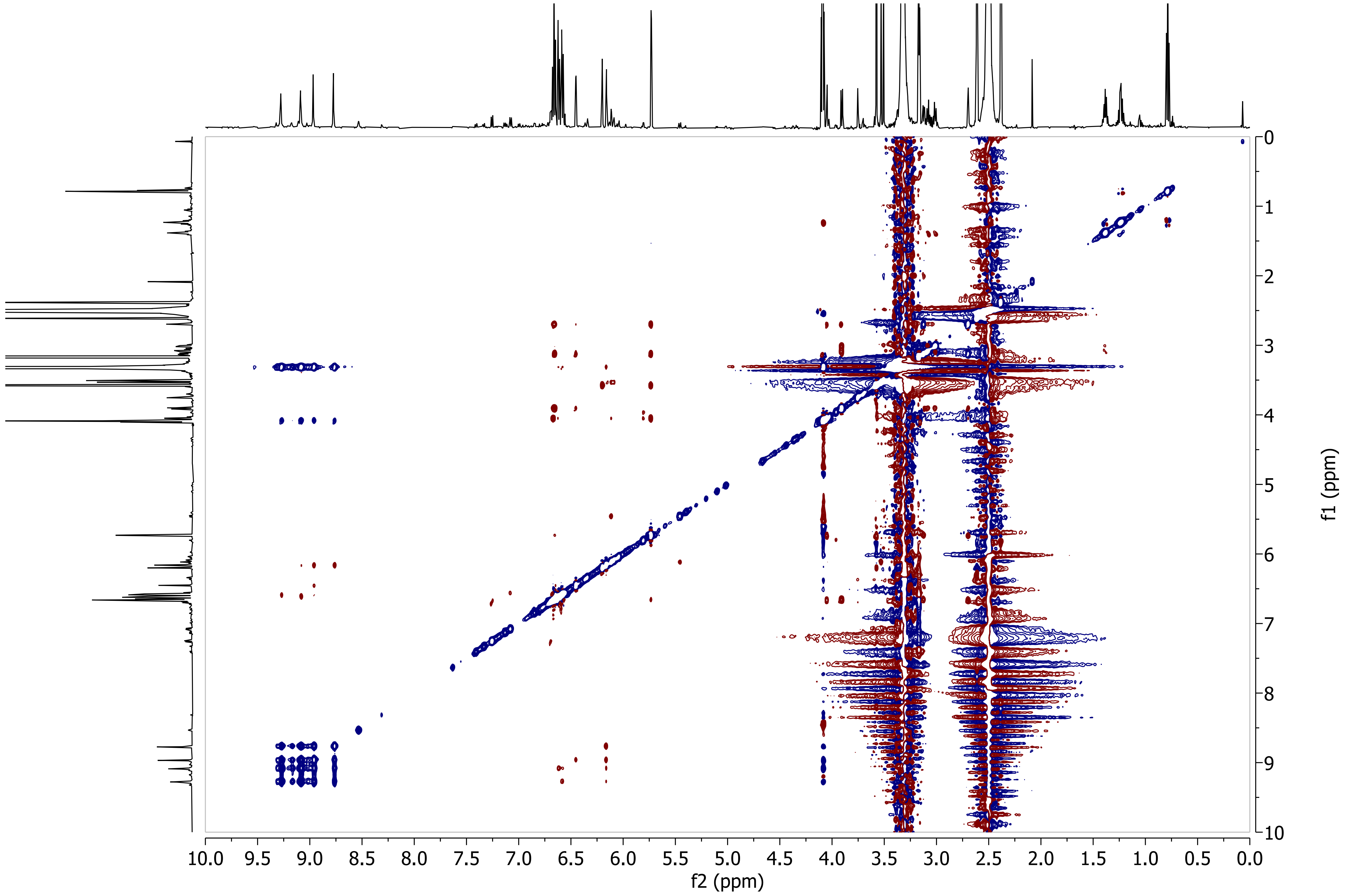

^{1}H NMR spectrum of compound **30** in DMSO-d_6

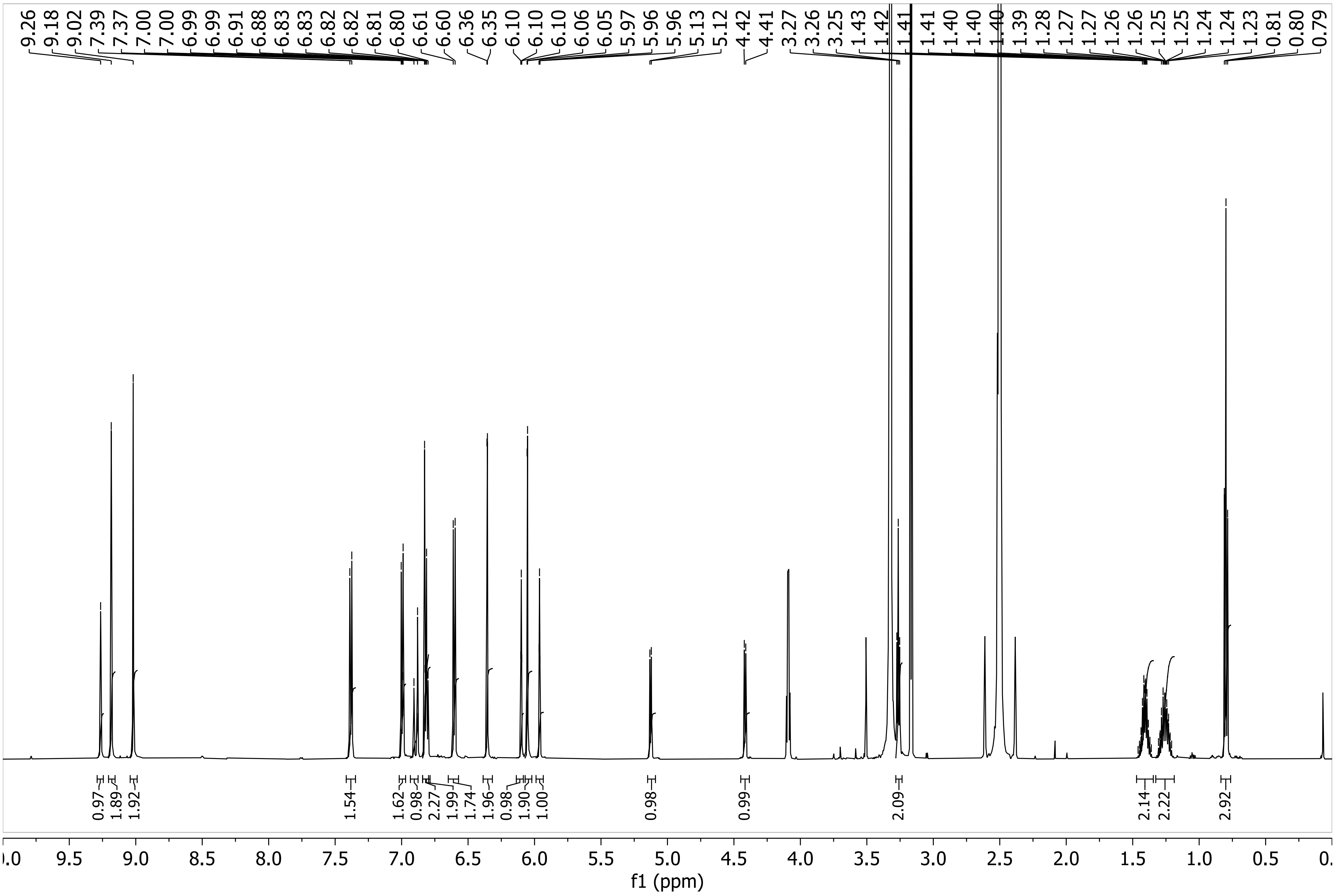

COSY NMR spectrum of compound **30** in DMSO-d_6

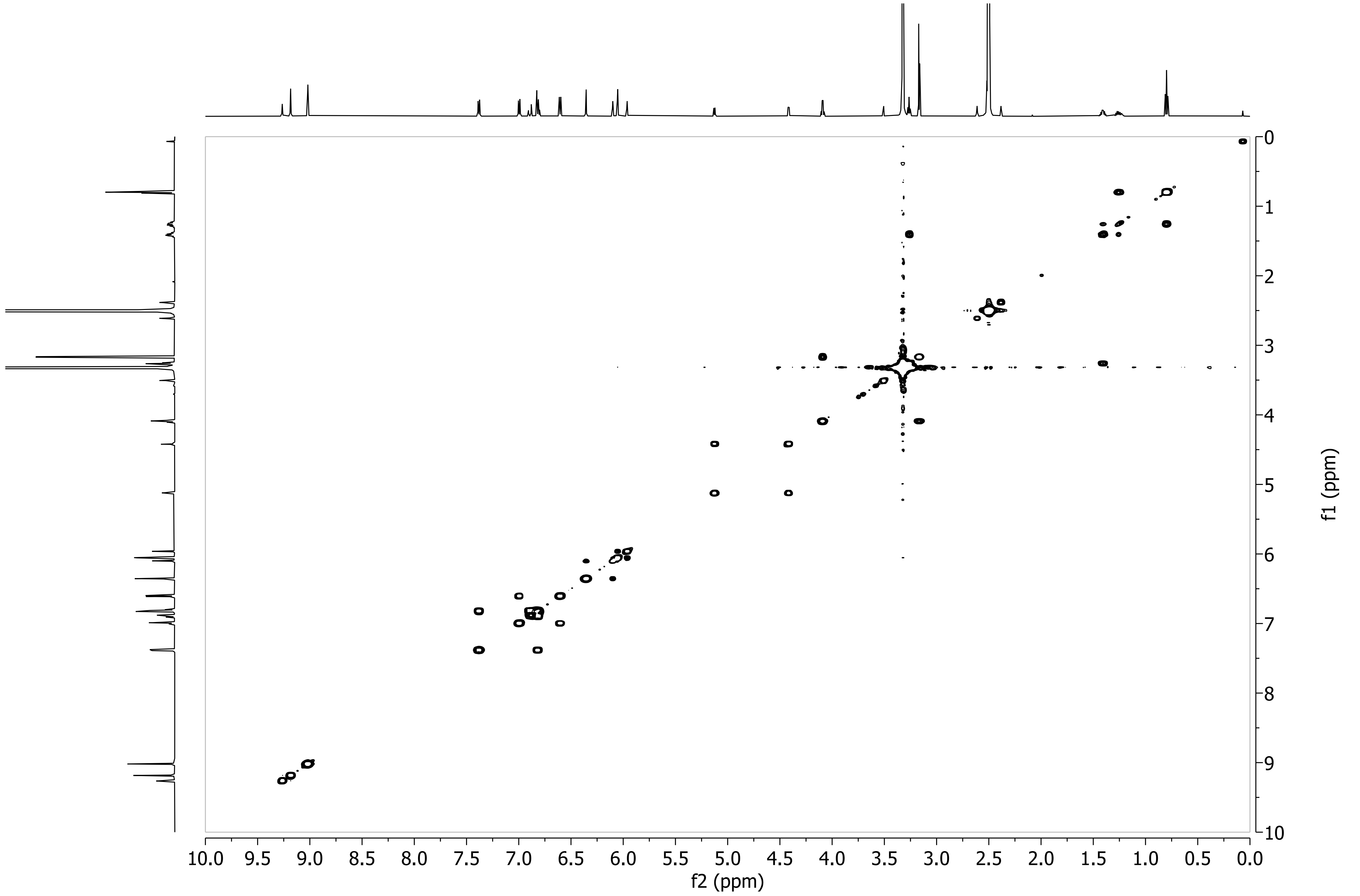

^{13}C-DEPTQ NMR spectrum of compound **30** in DMSO-d_6

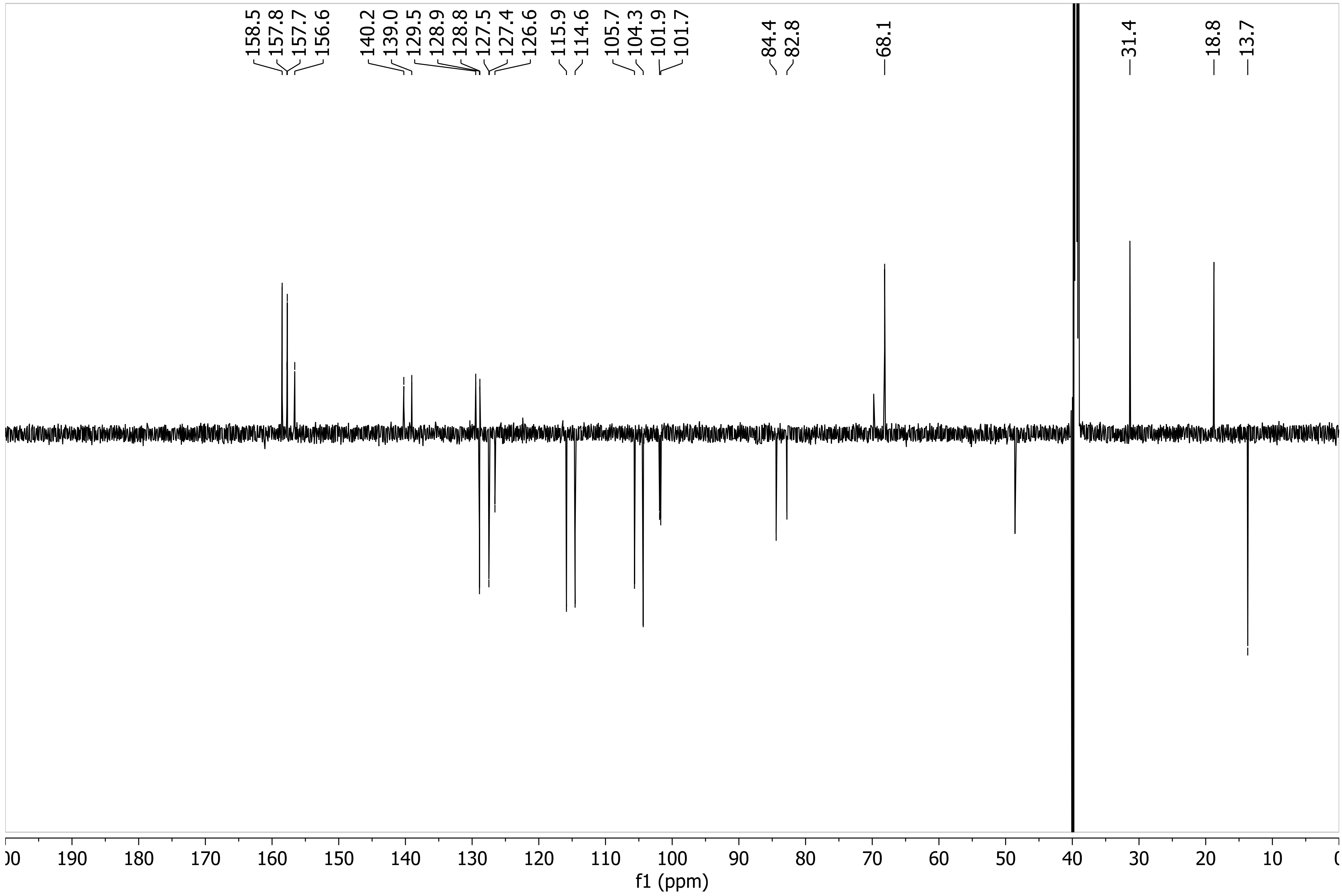

Edited-HSQC NMR spectrum of compound **30** in DMSO-d_6

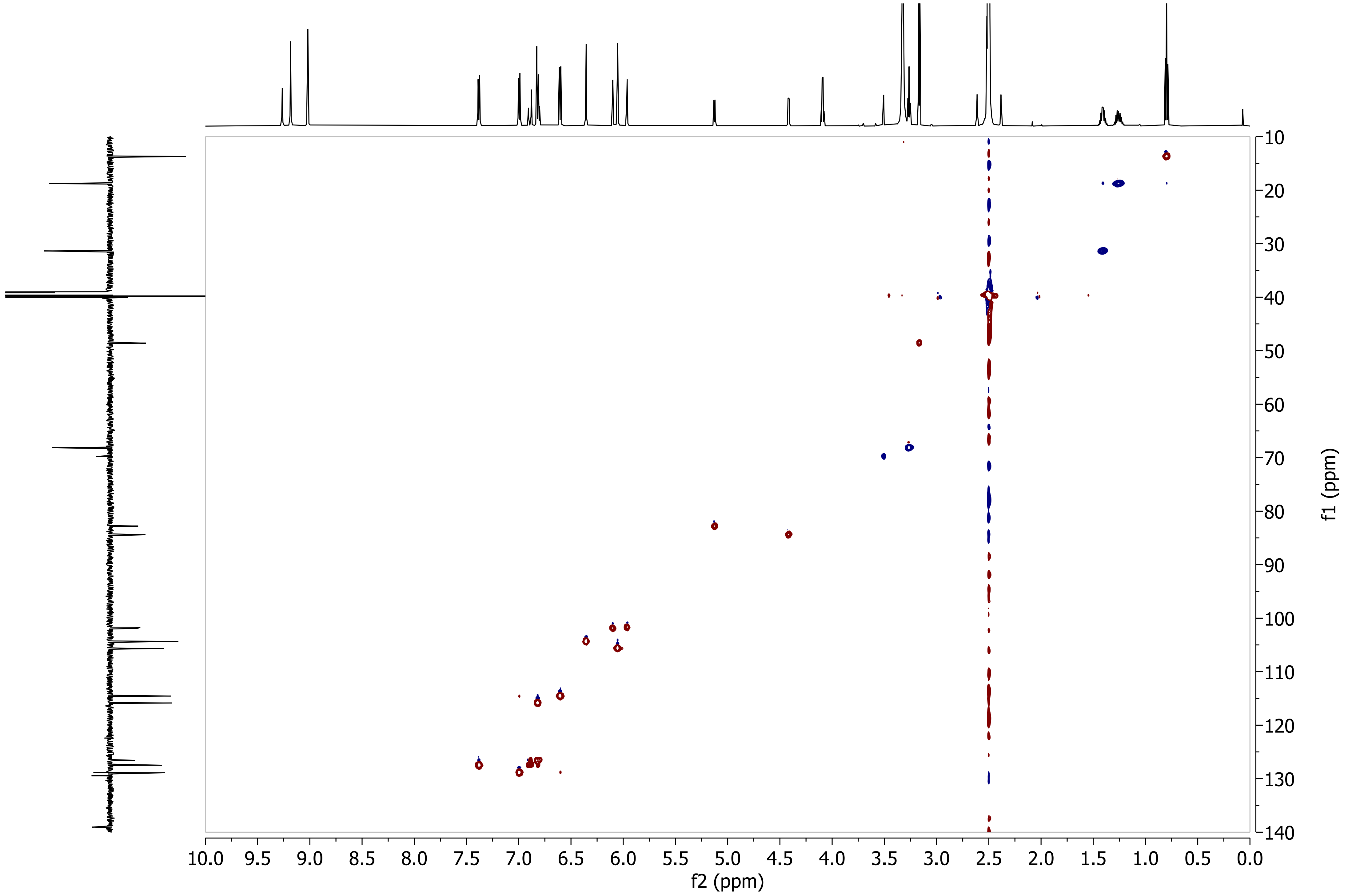

HMBC NMR spectrum of compound **30** in DMSO-d_6

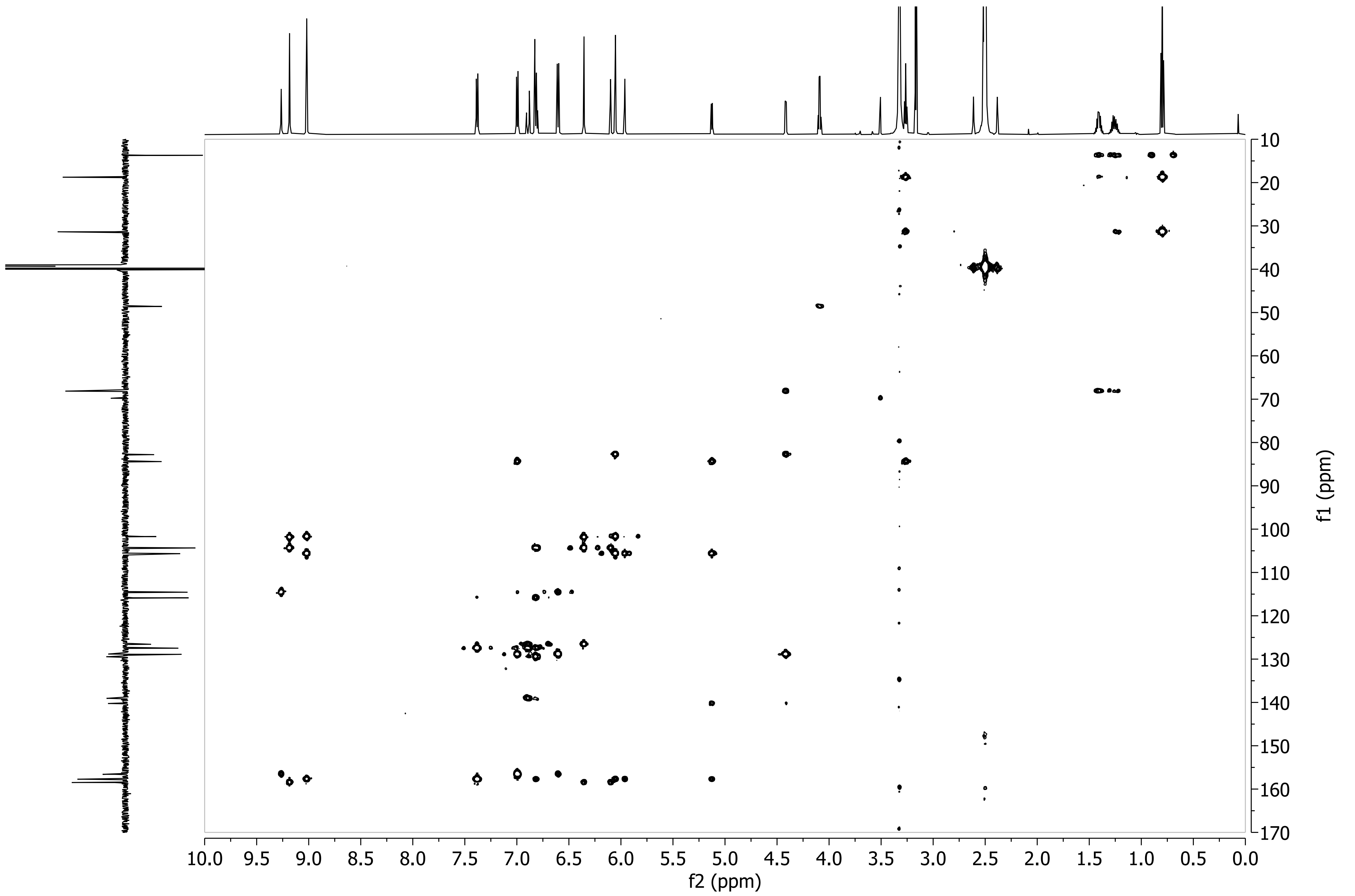

ROESY NMR spectrum of compound **30** in DMSO-d_6

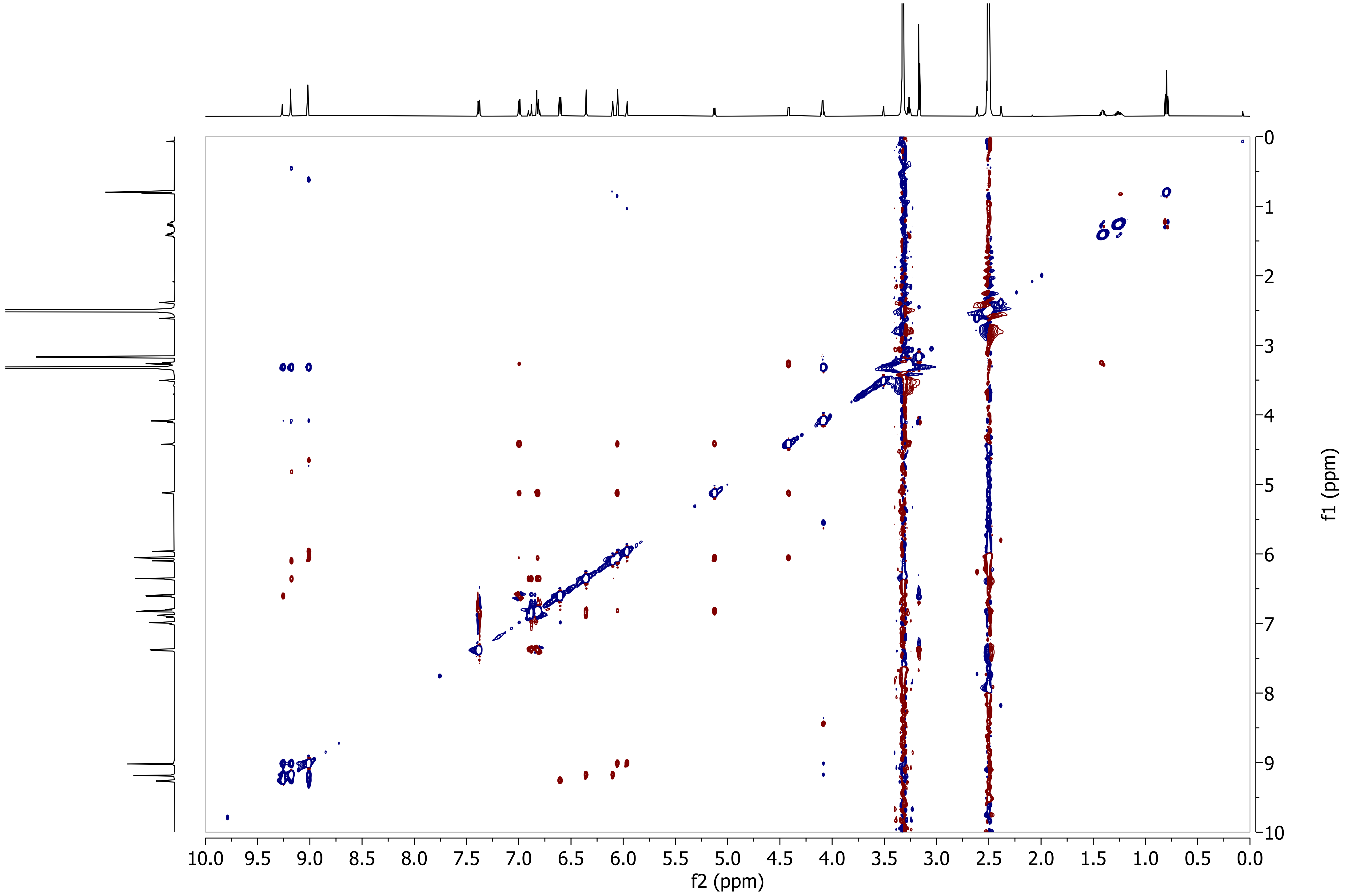

^{1}H NMR spectrum of compound **31** in DMSO-d_6

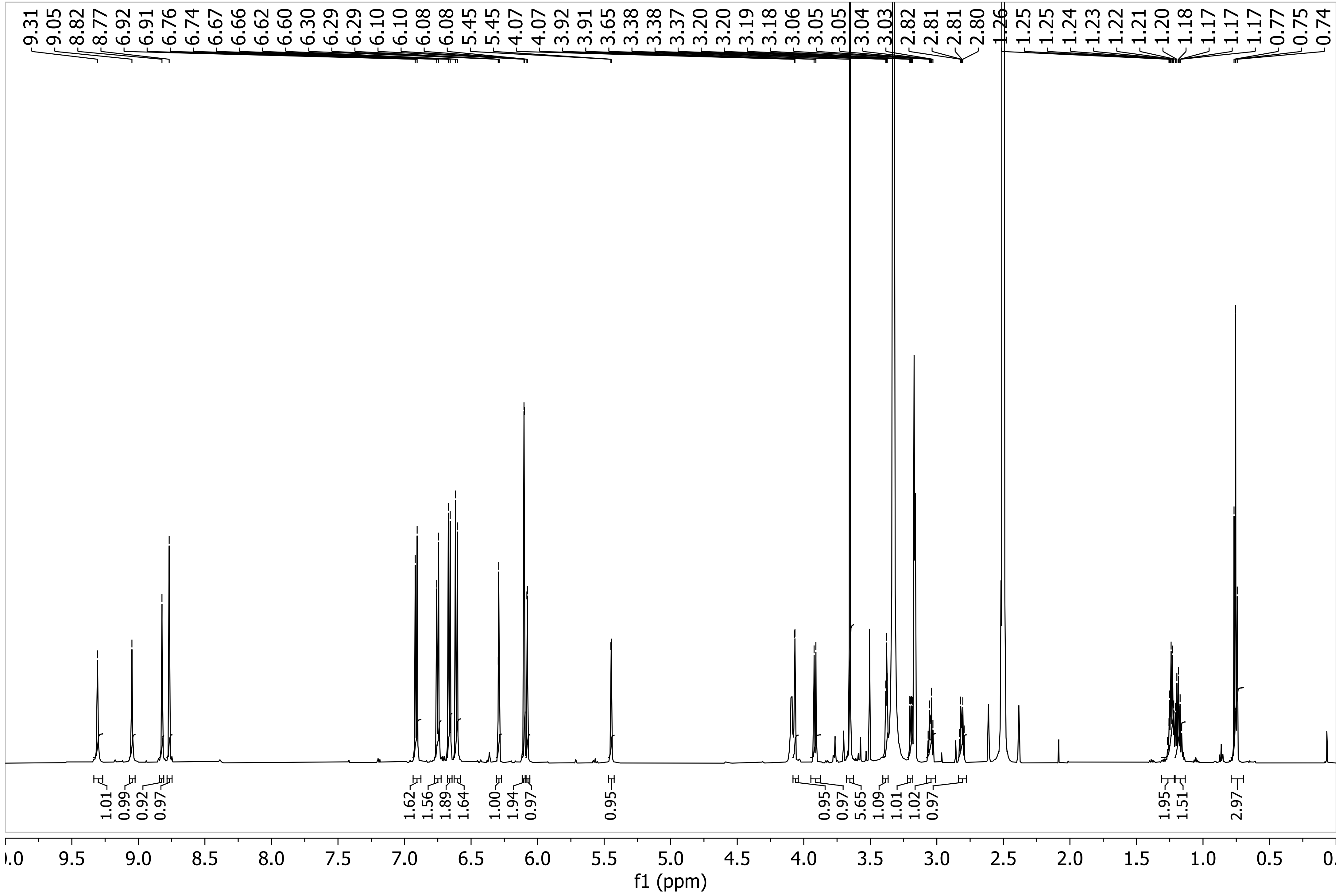

COSY NMR spectrum of compound **31** in DMSO-d_6

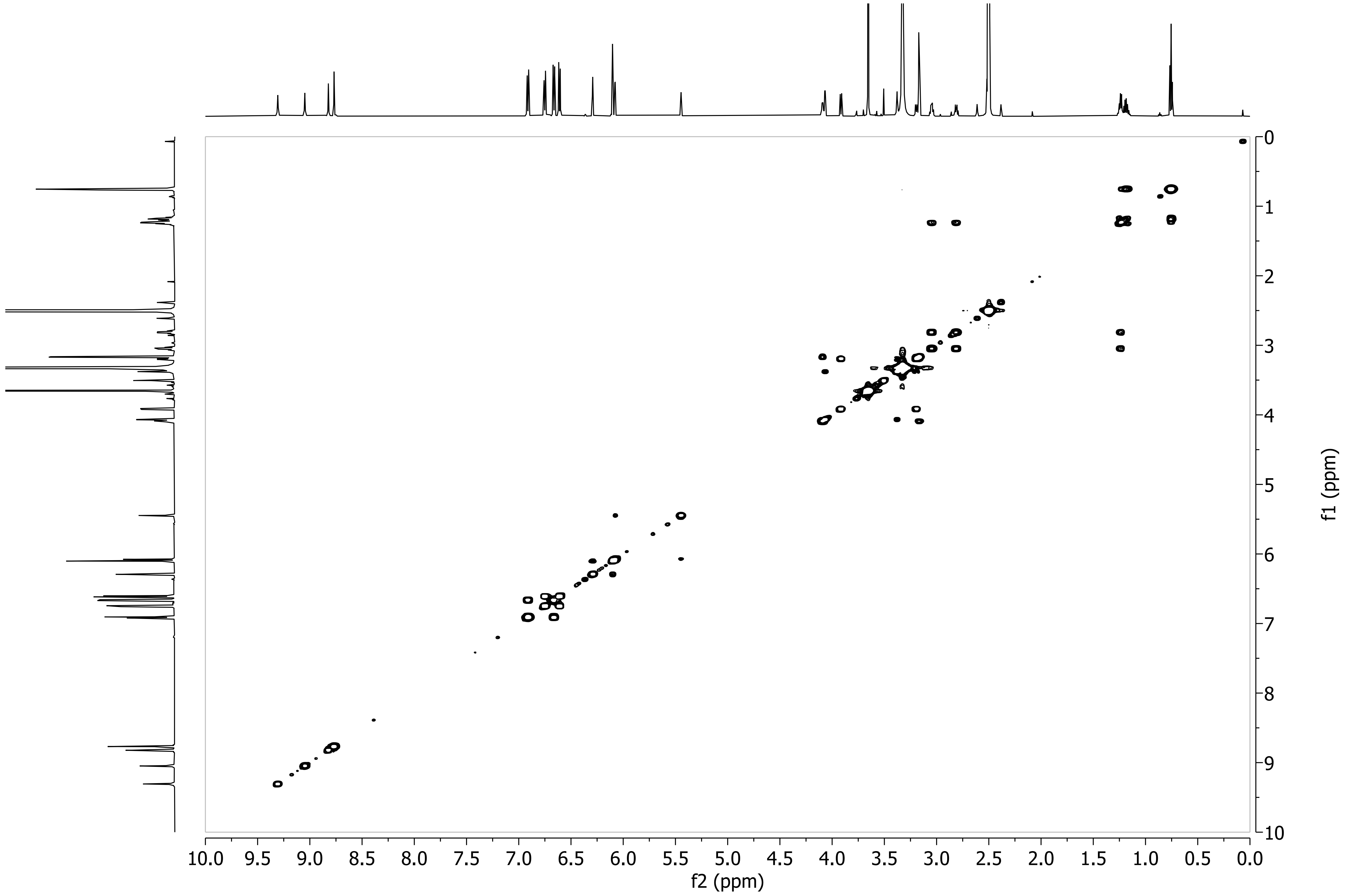

^{13}C-DEPTQ NMR spectrum of compound **31** in DMSO-d_6

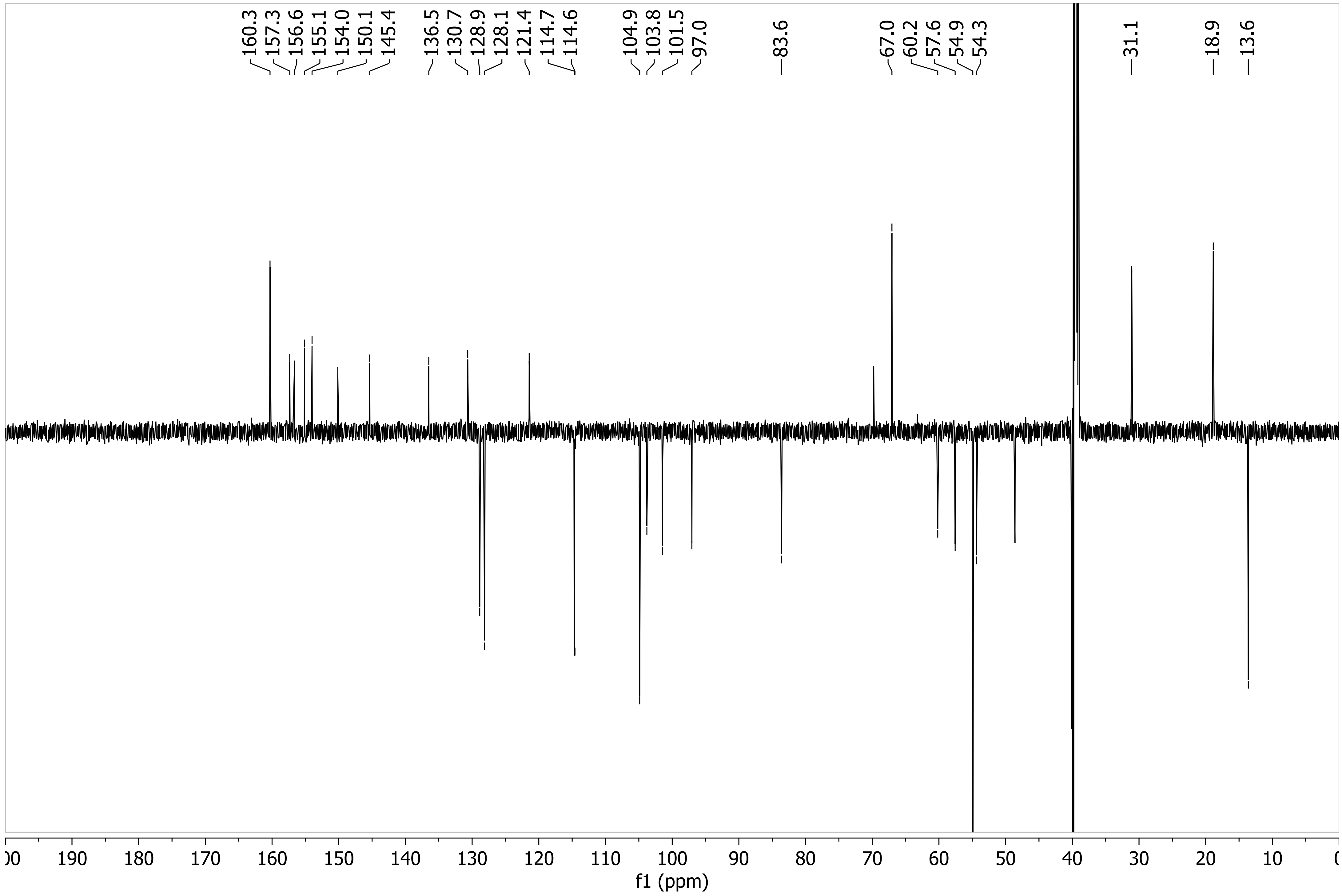

Edited-HSQC NMR spectrum of compound **31** in DMSO-d_6

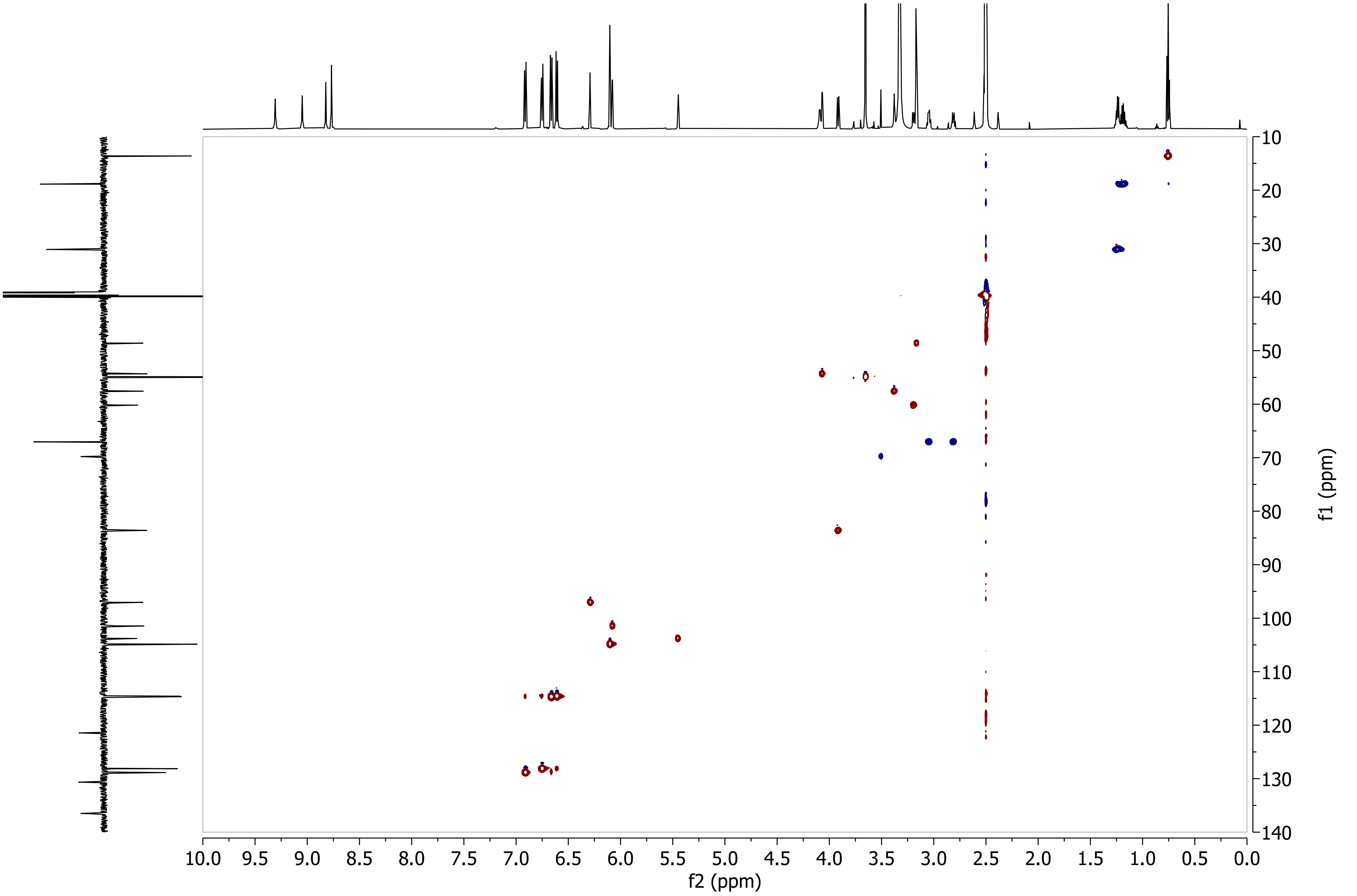

HMBC NMR spectrum of compound **31** in DMSO-d_6

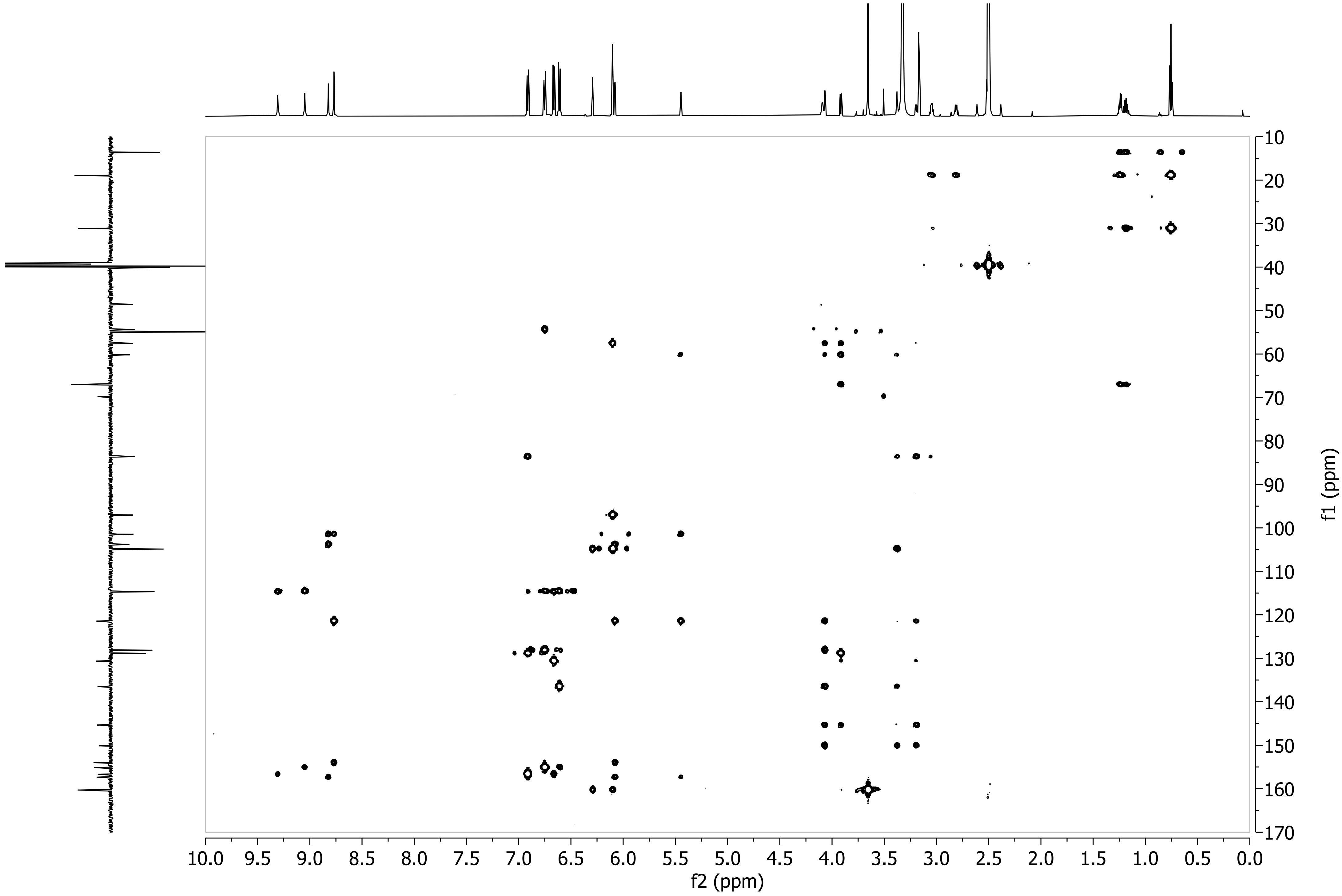

ROESY NMR spectrum of compound **31** in DMSO-d_6

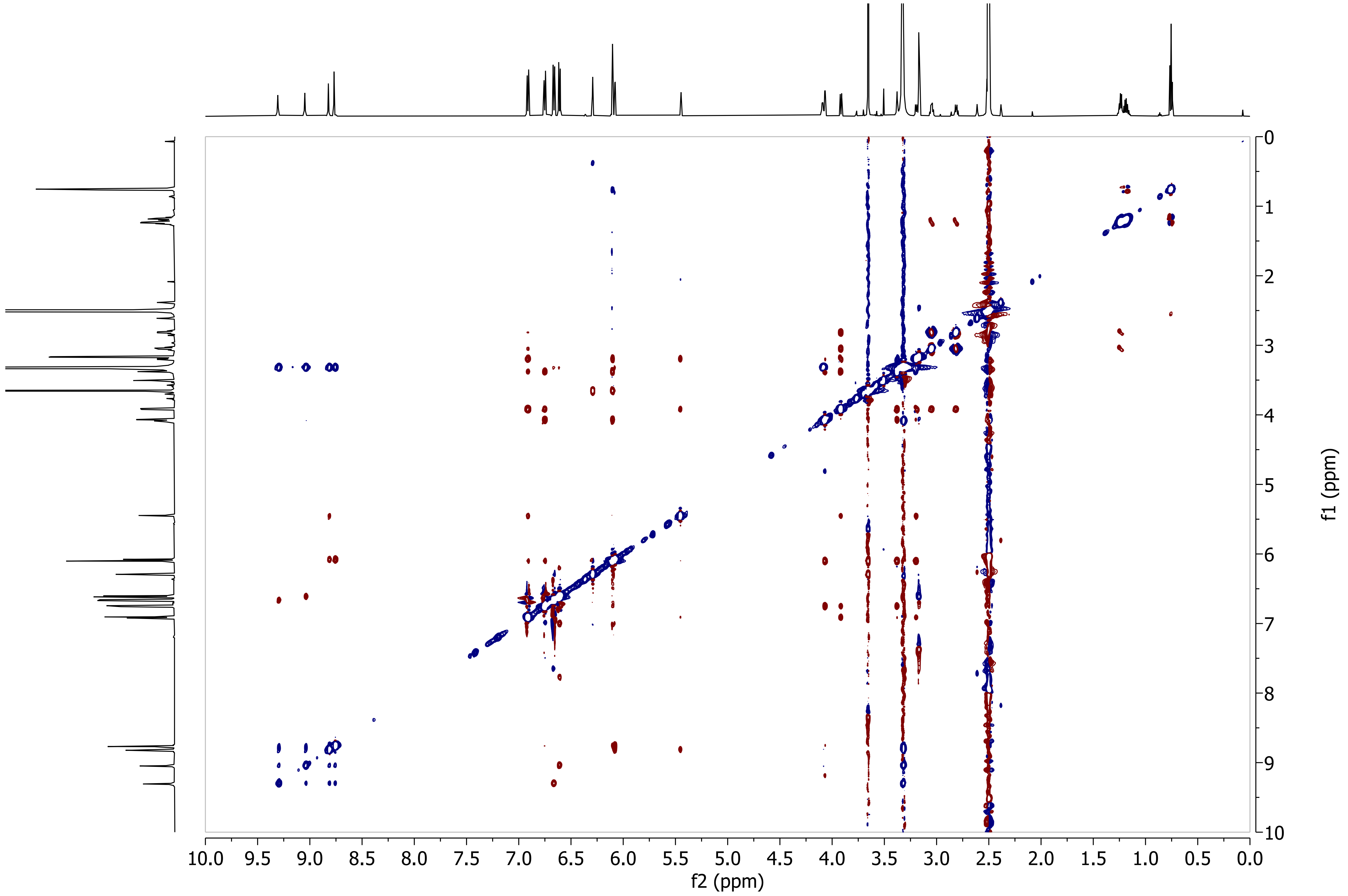

^{1}H NMR spectrum of compound **32** in DMSO-d_6

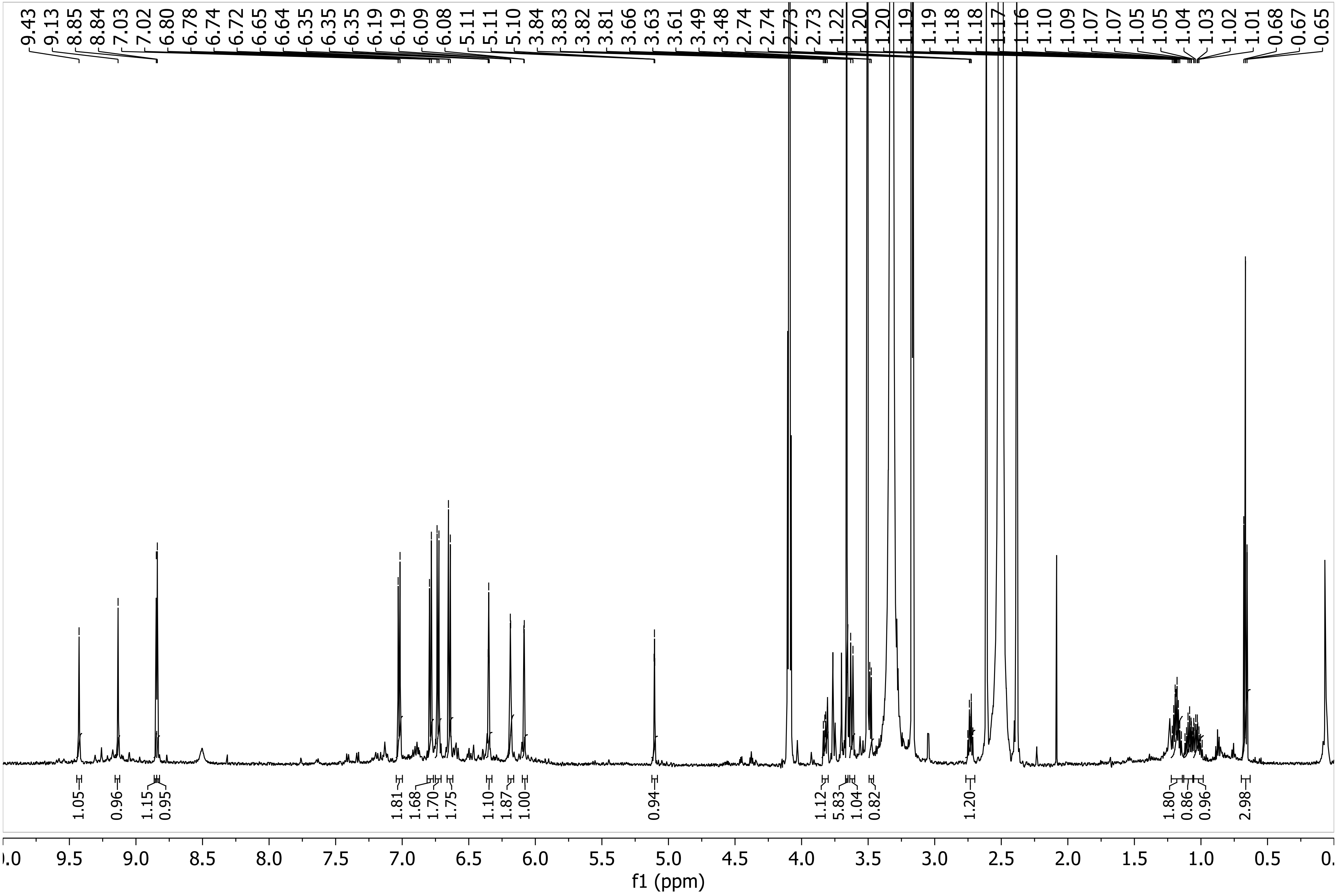

COSY NMR spectrum of compound **32** in DMSO-d_6

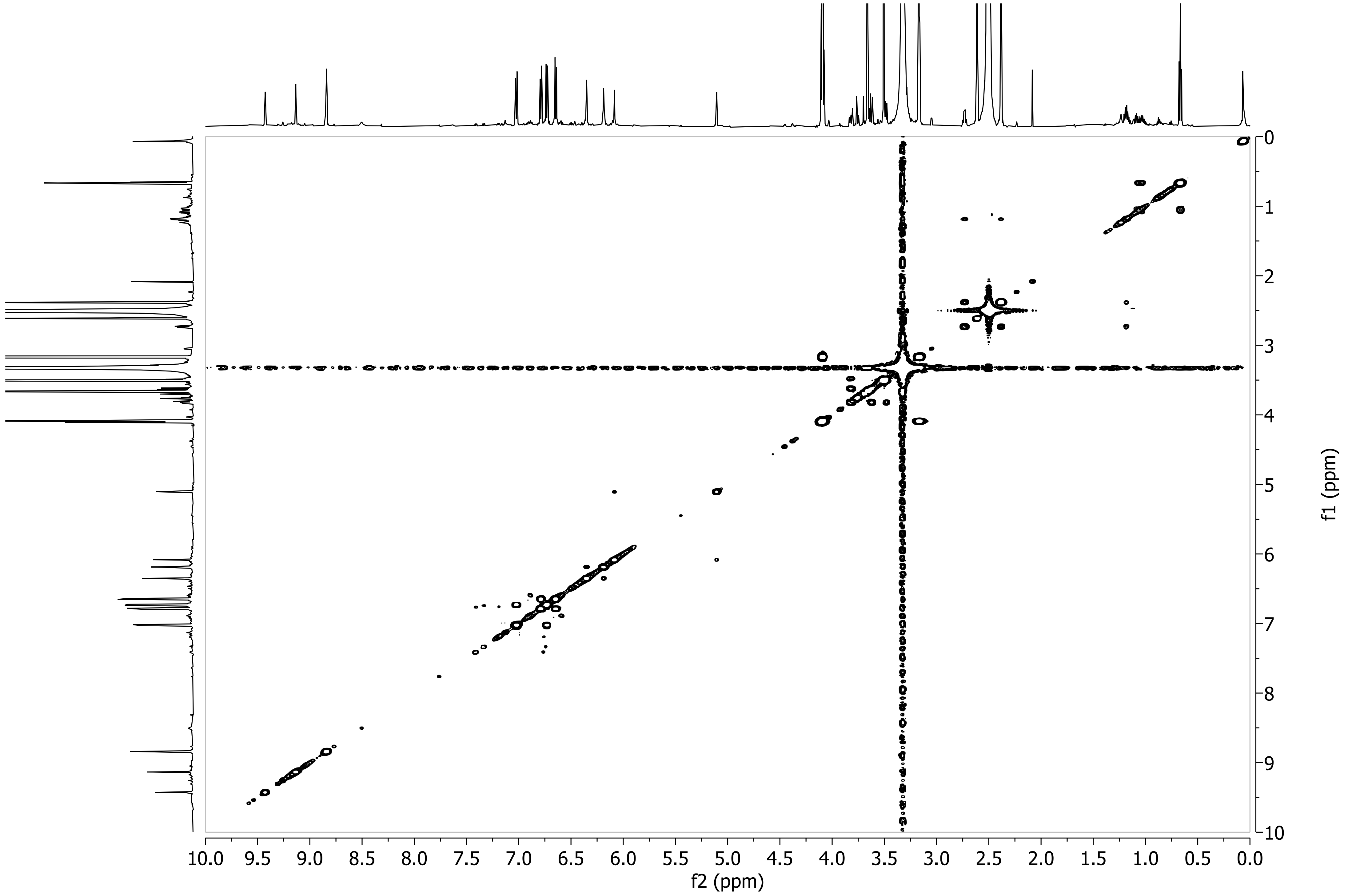

Edited-HSQC NMR spectrum of compound **32** in DMSO-d_6

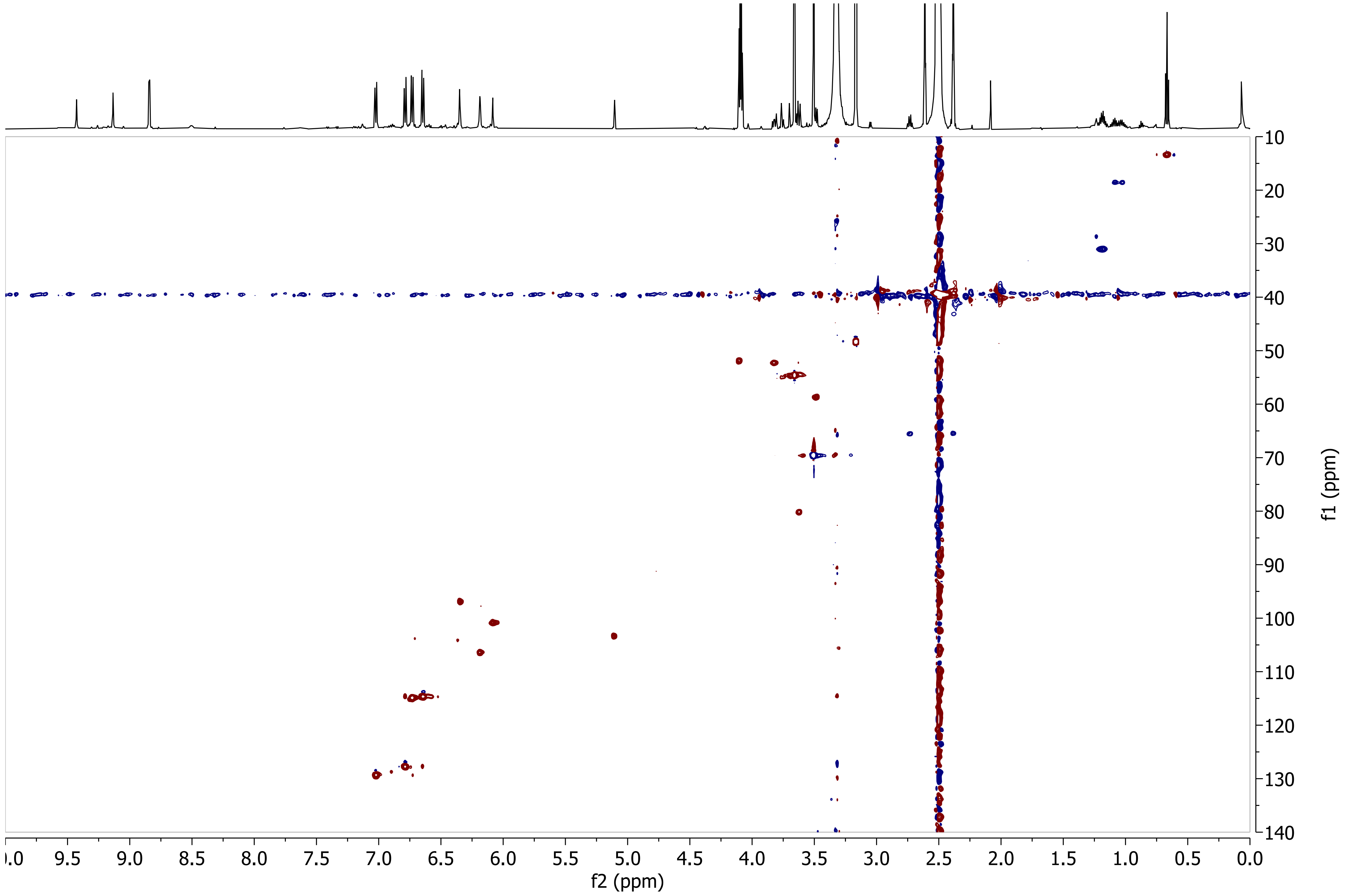

HMBC NMR spectrum of compound **32** in DMSO-d_6

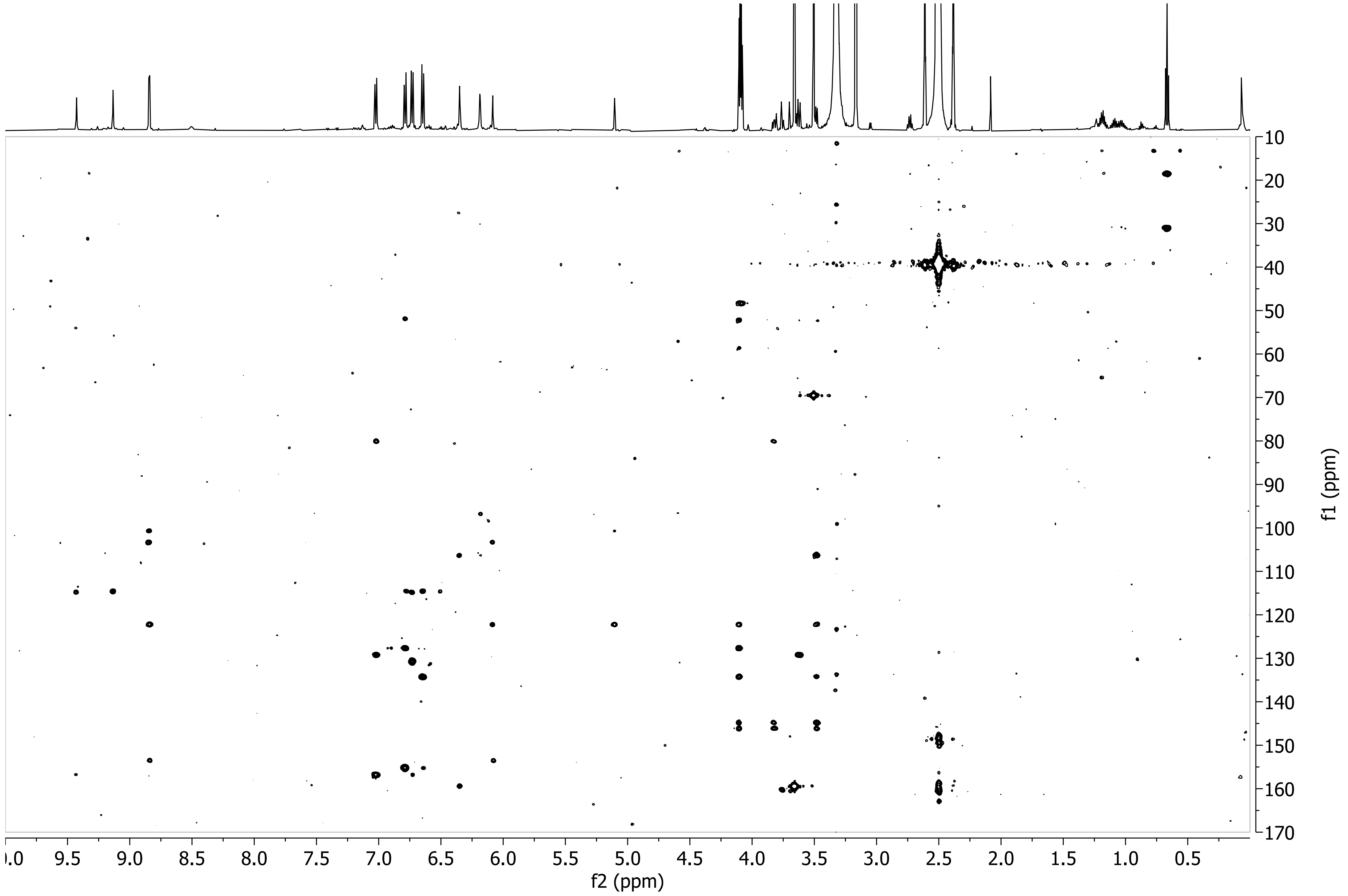

ROESY NMR spectrum of compound **32** in DMSO-d_6

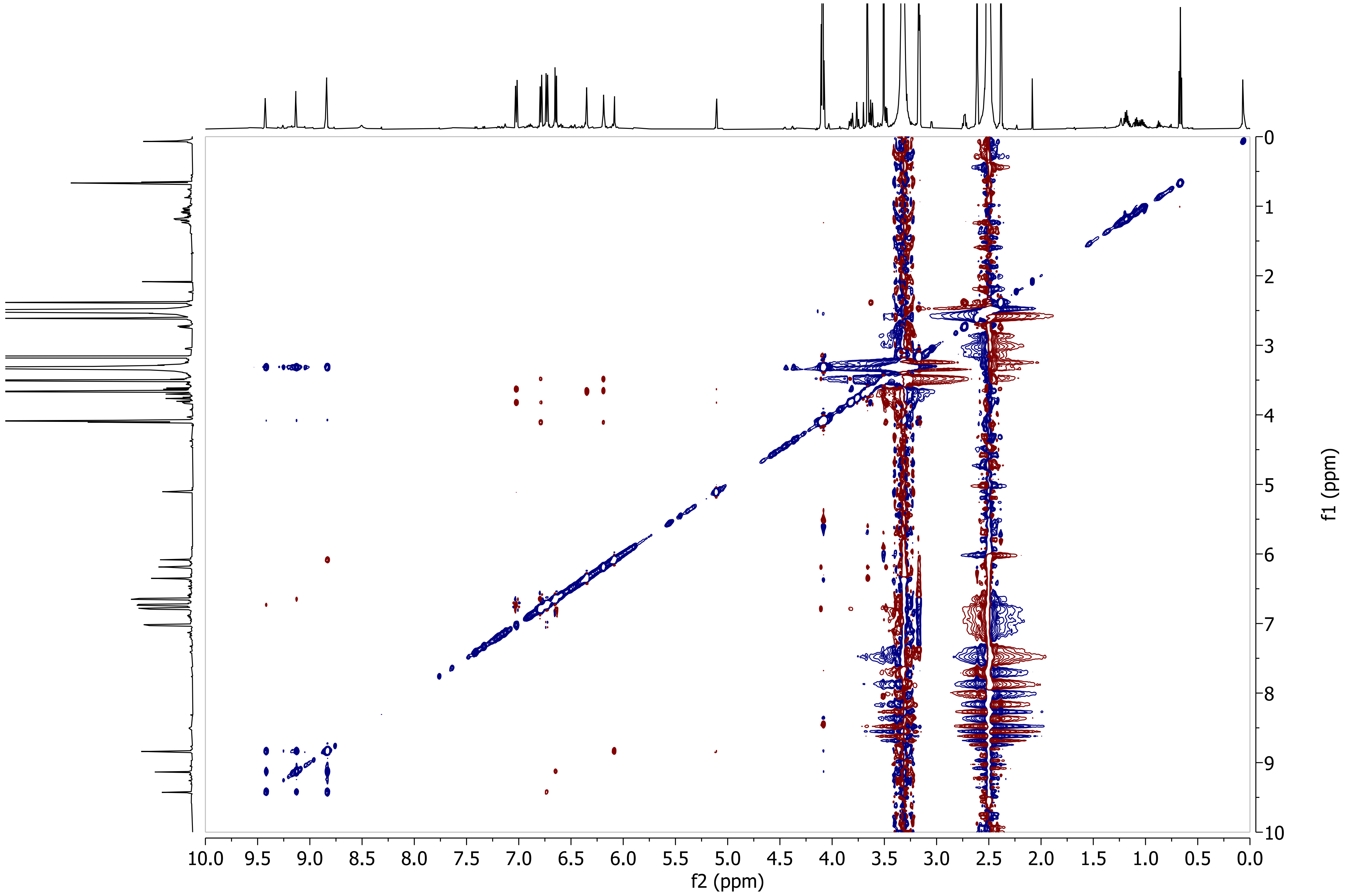

^{1}H NMR spectrum of compound **33** in DMSO-d_6

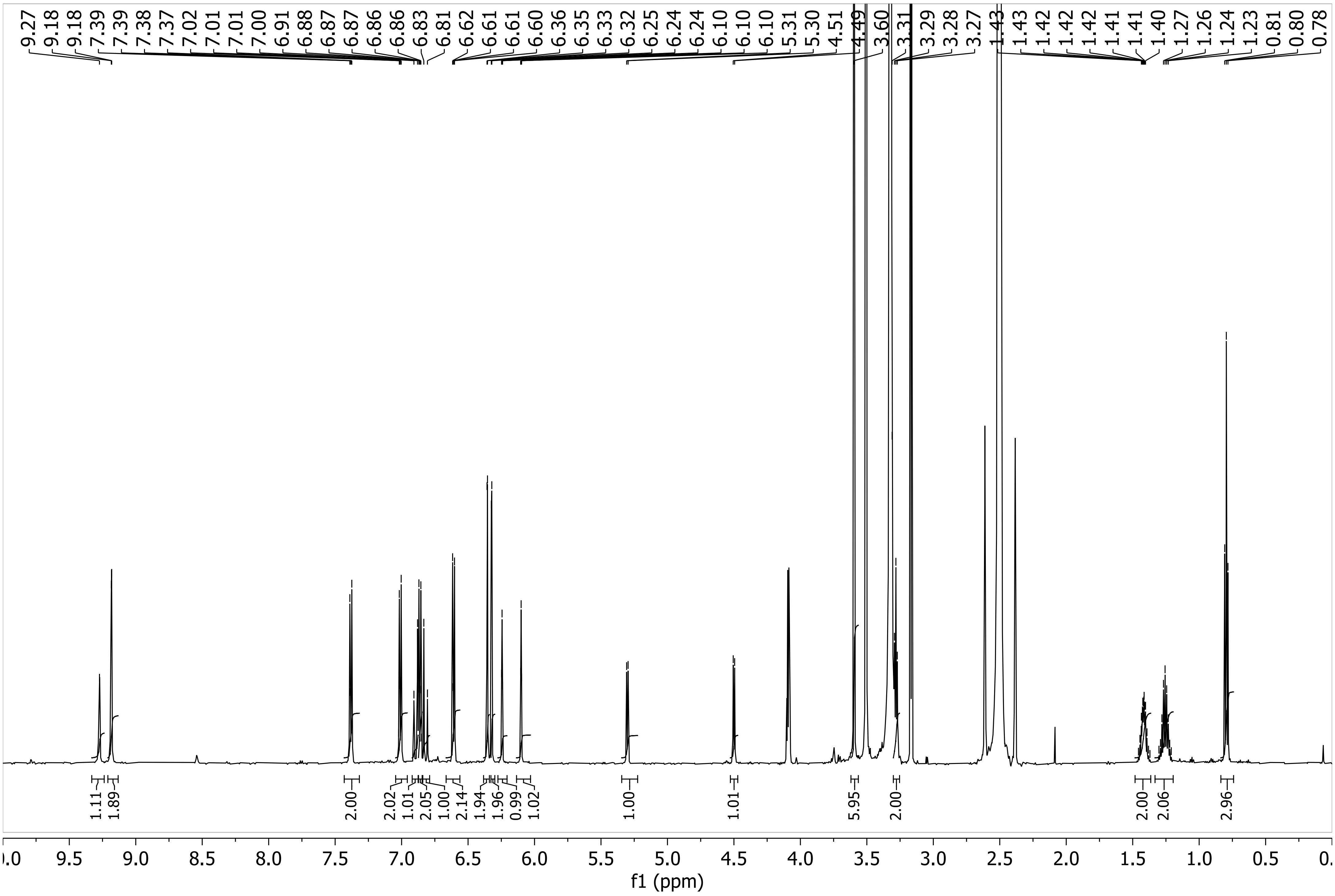

COSY NMR spectrum of compound **33** in DMSO-d_6

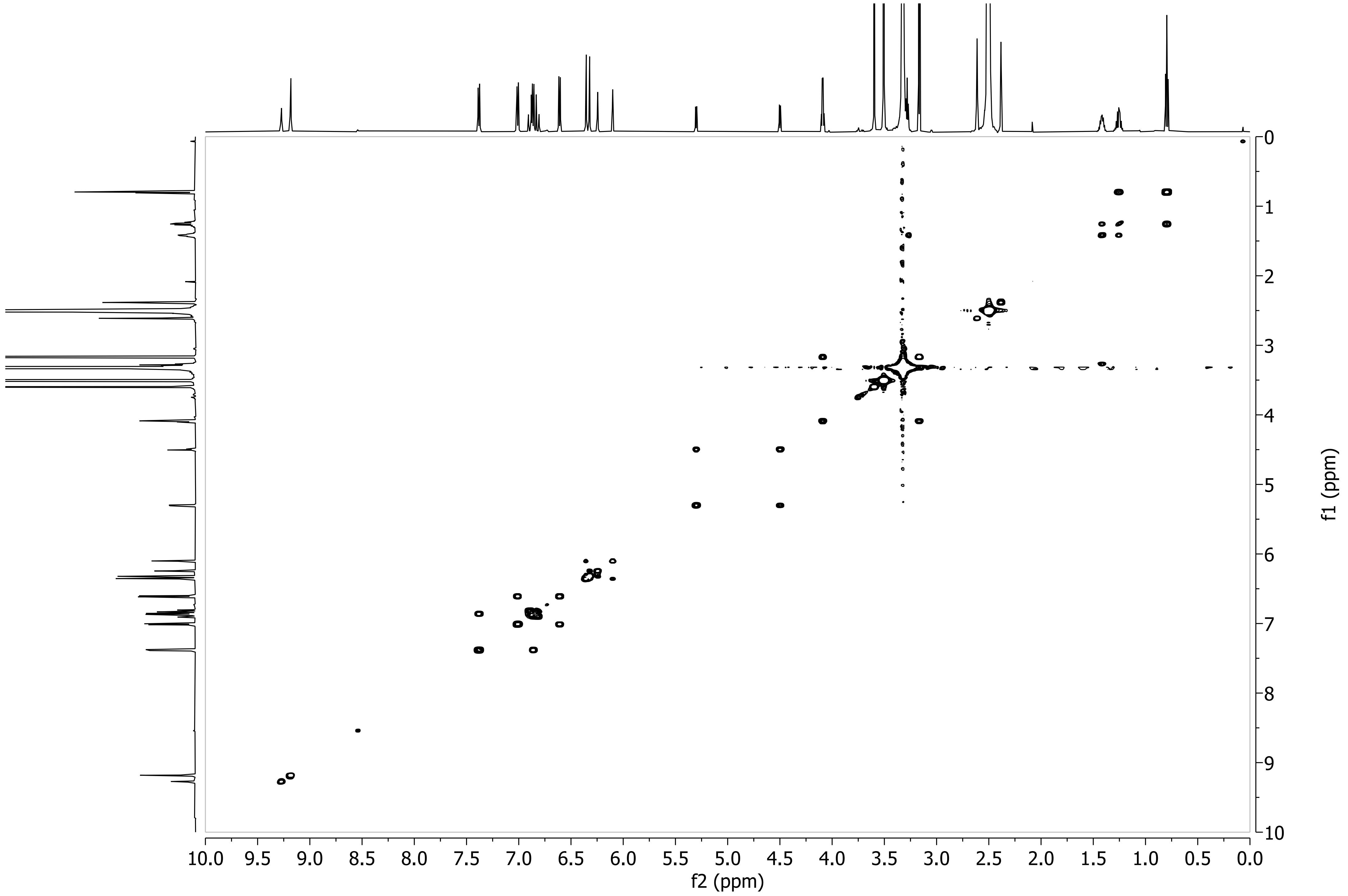

Edited-HSQC NMR spectrum of compound **33** in DMSO-d_6

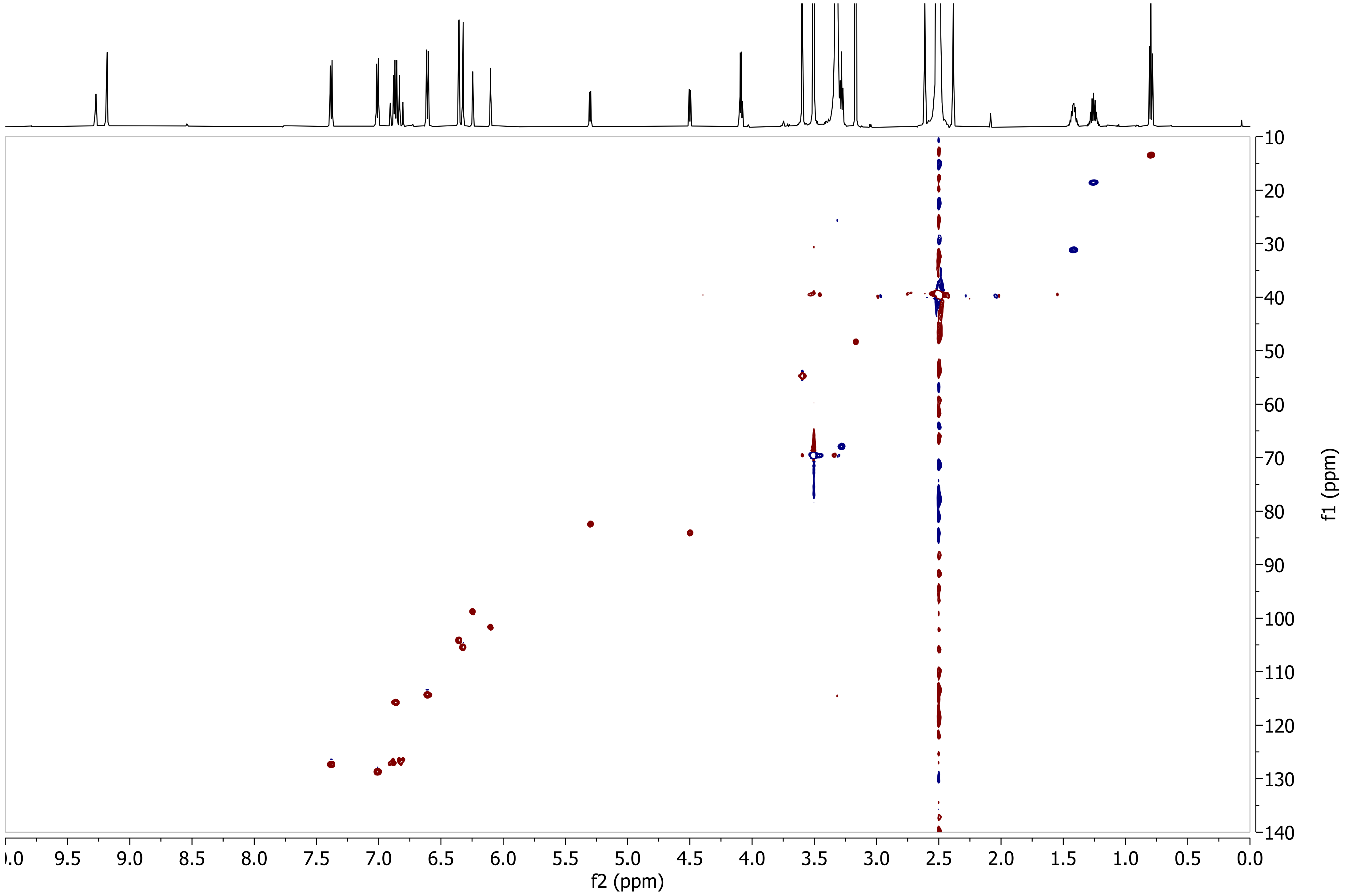

HMBC NMR spectrum of compound **33** in DMSO-d_6

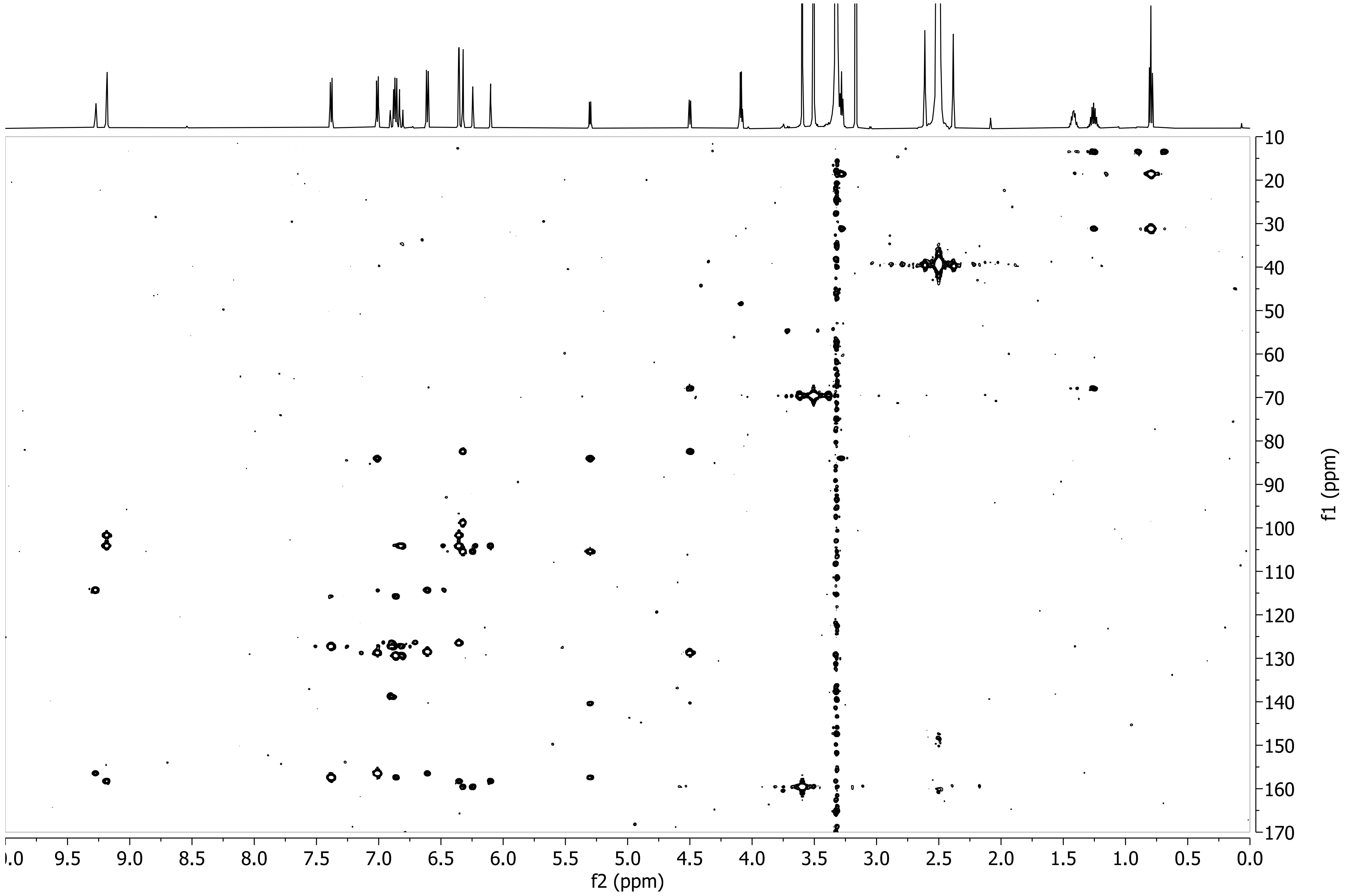

ROESY NMR spectrum of compound **33** in DMSO-d_6

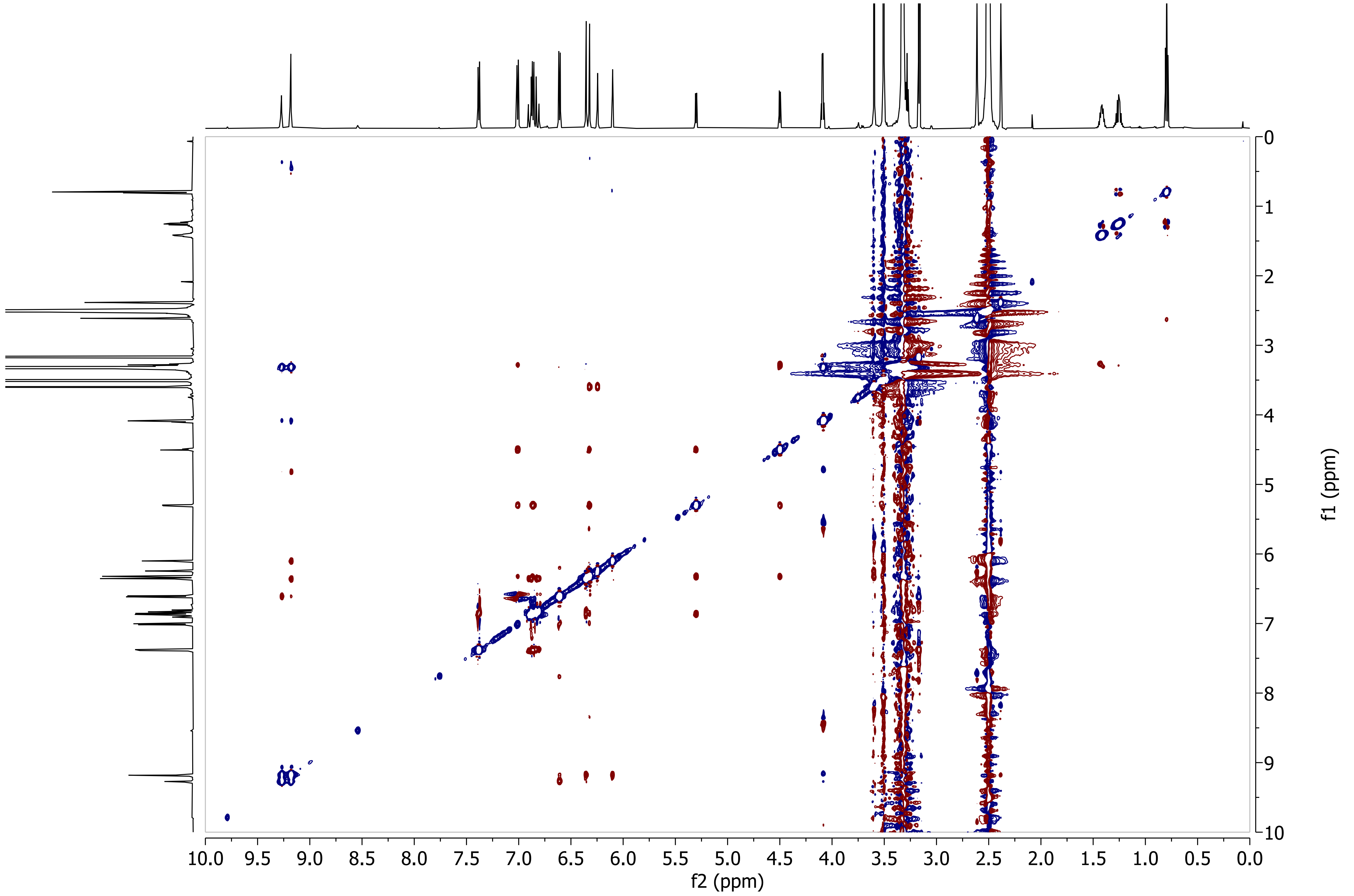

^{1}H NMR spectrum of compound **34** in DMSO-d_6

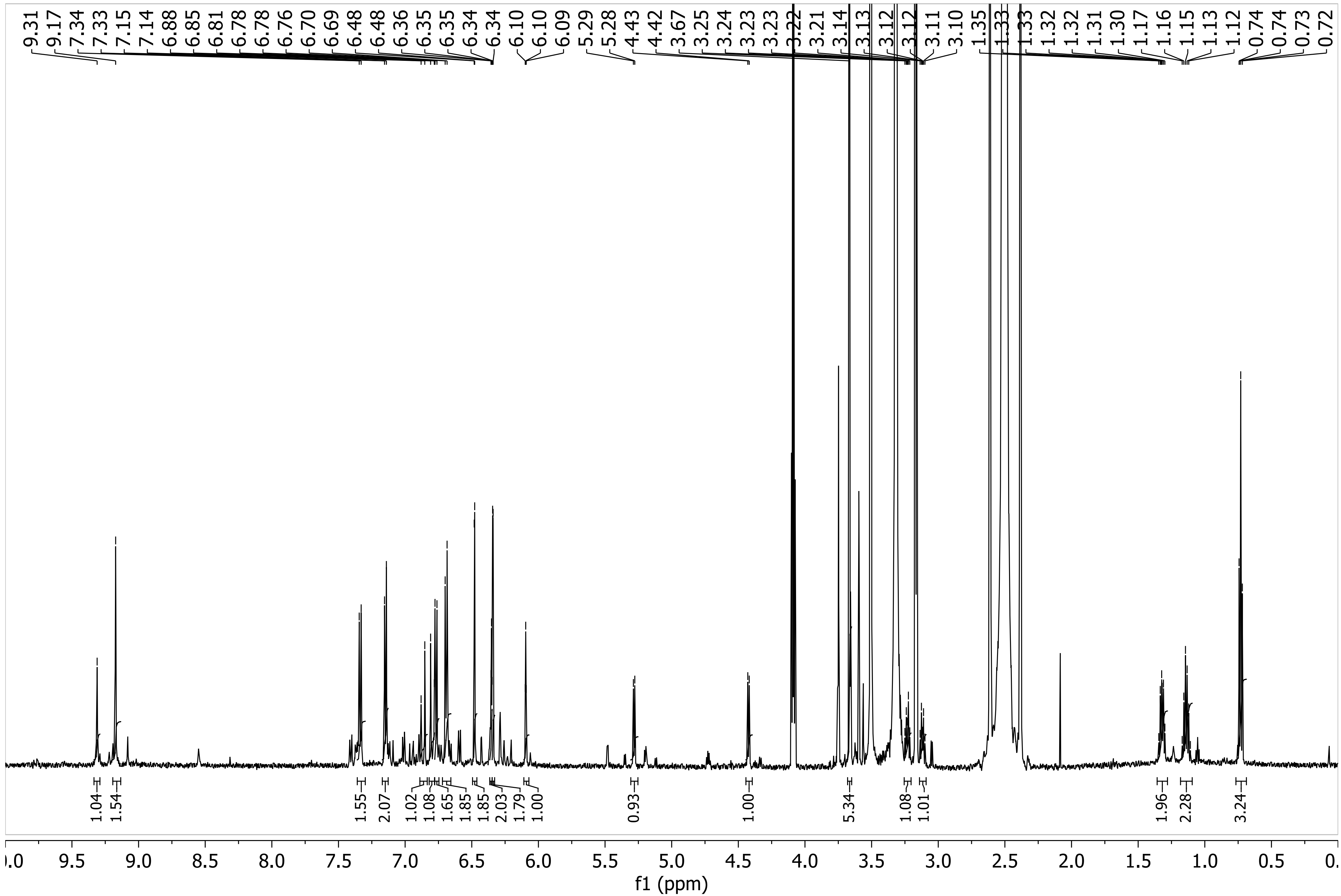

COSY NMR spectrum of compound **34** in DMSO-d_6

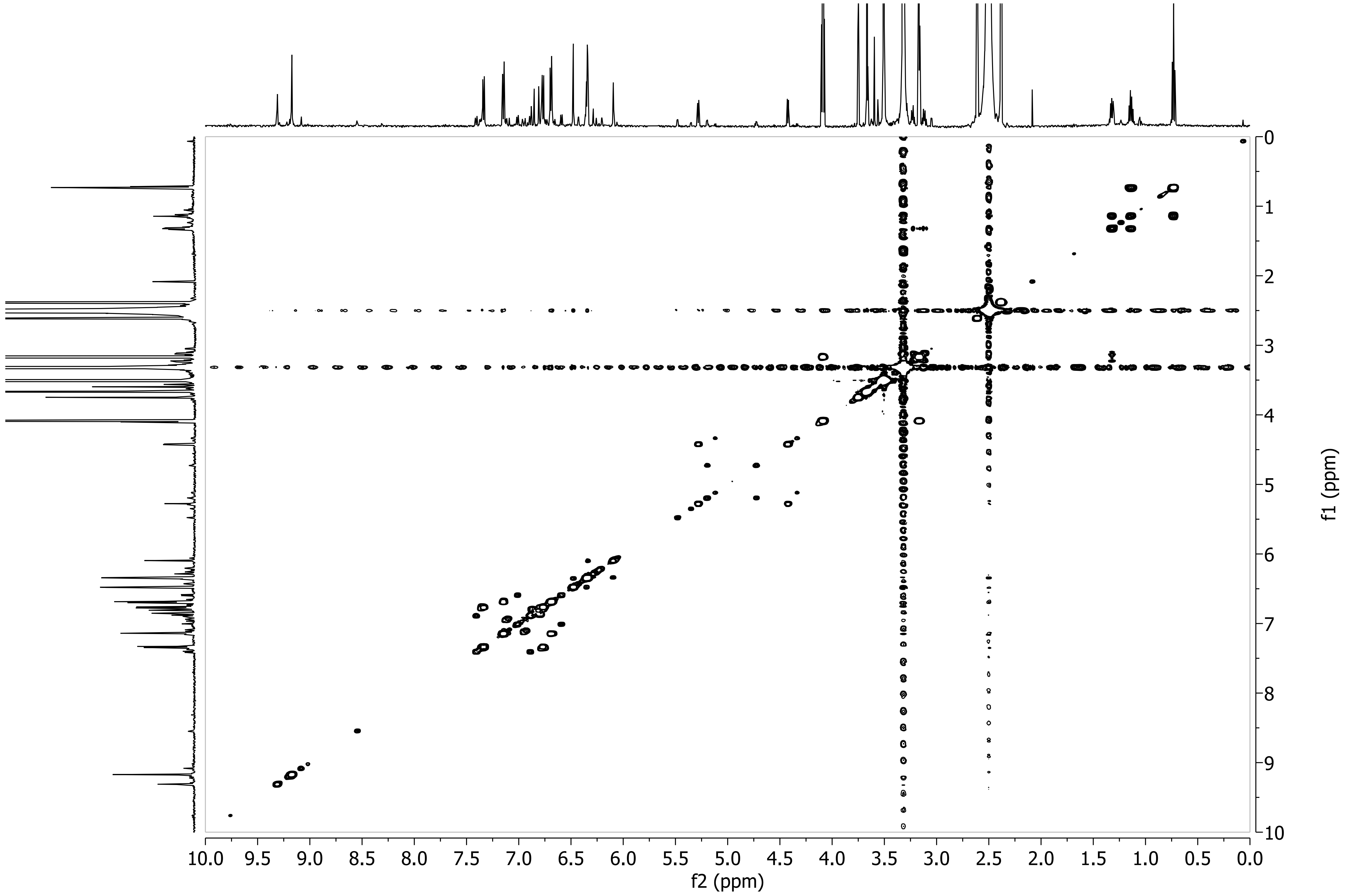

ROESY NMR spectrum of compound **34** in DMSO-d_6

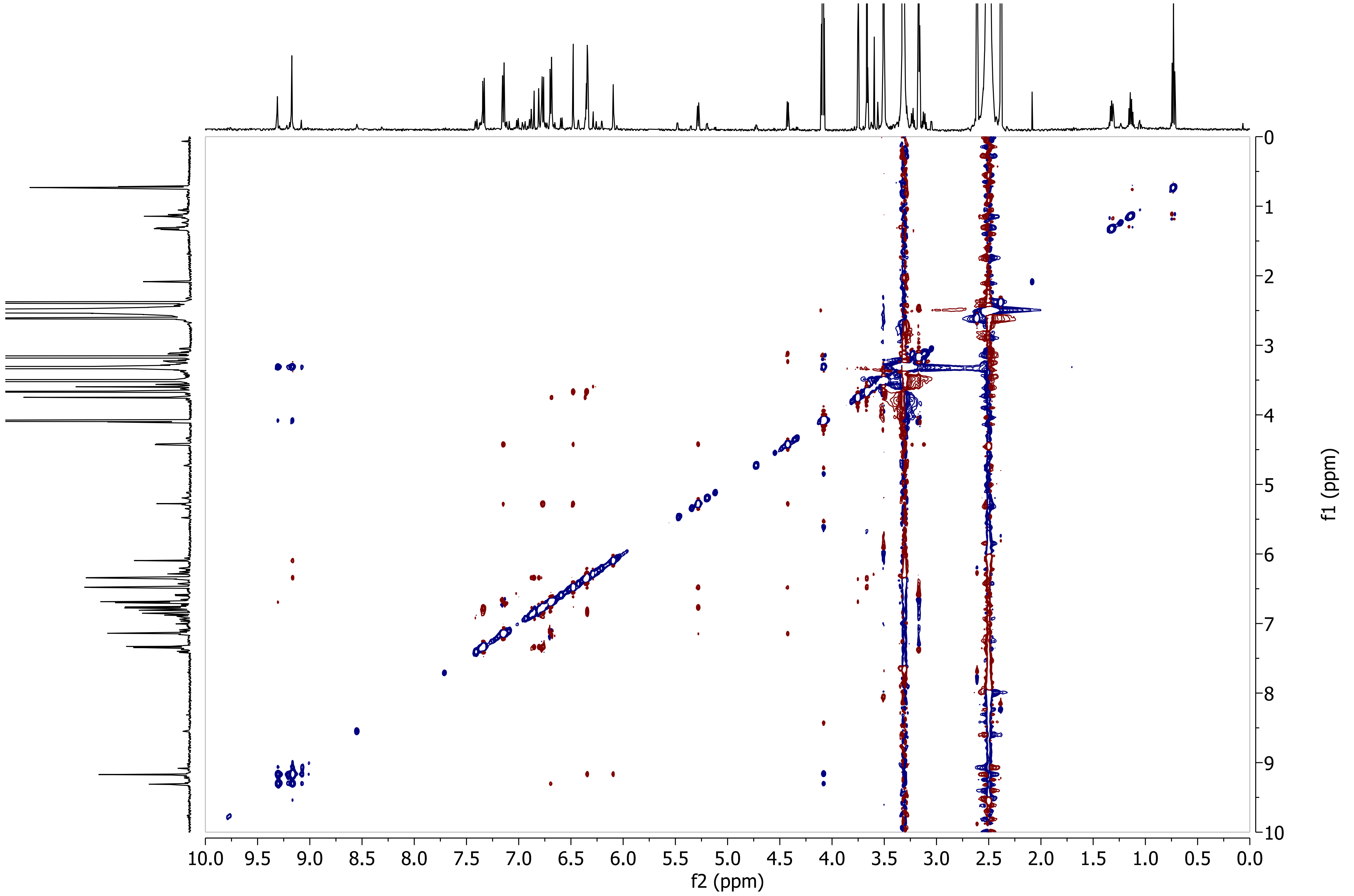

^{1}H NMR spectrum of compound **35** in DMSO-d_6

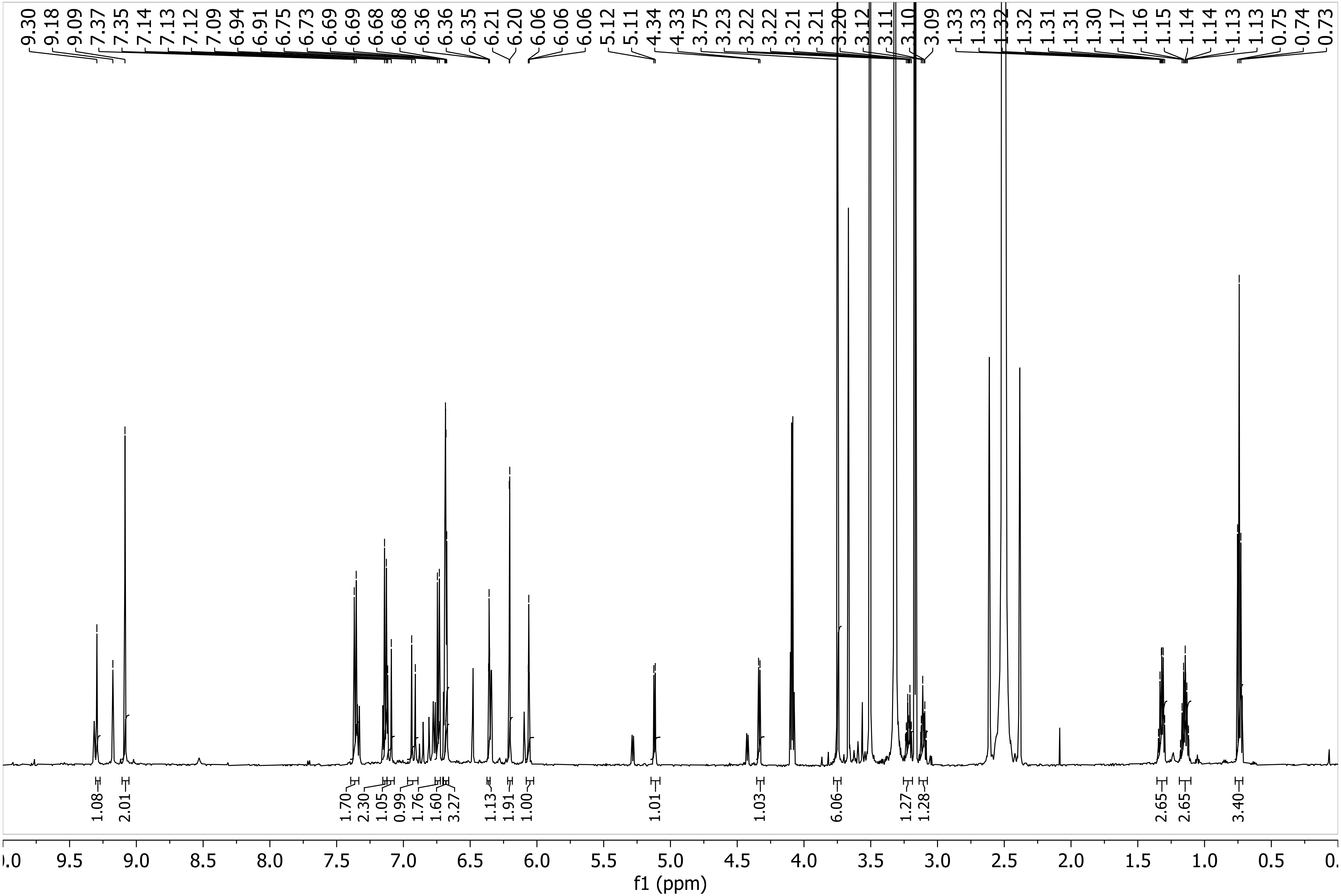

COSY NMR spectrum of compound **35** in DMSO-d_6

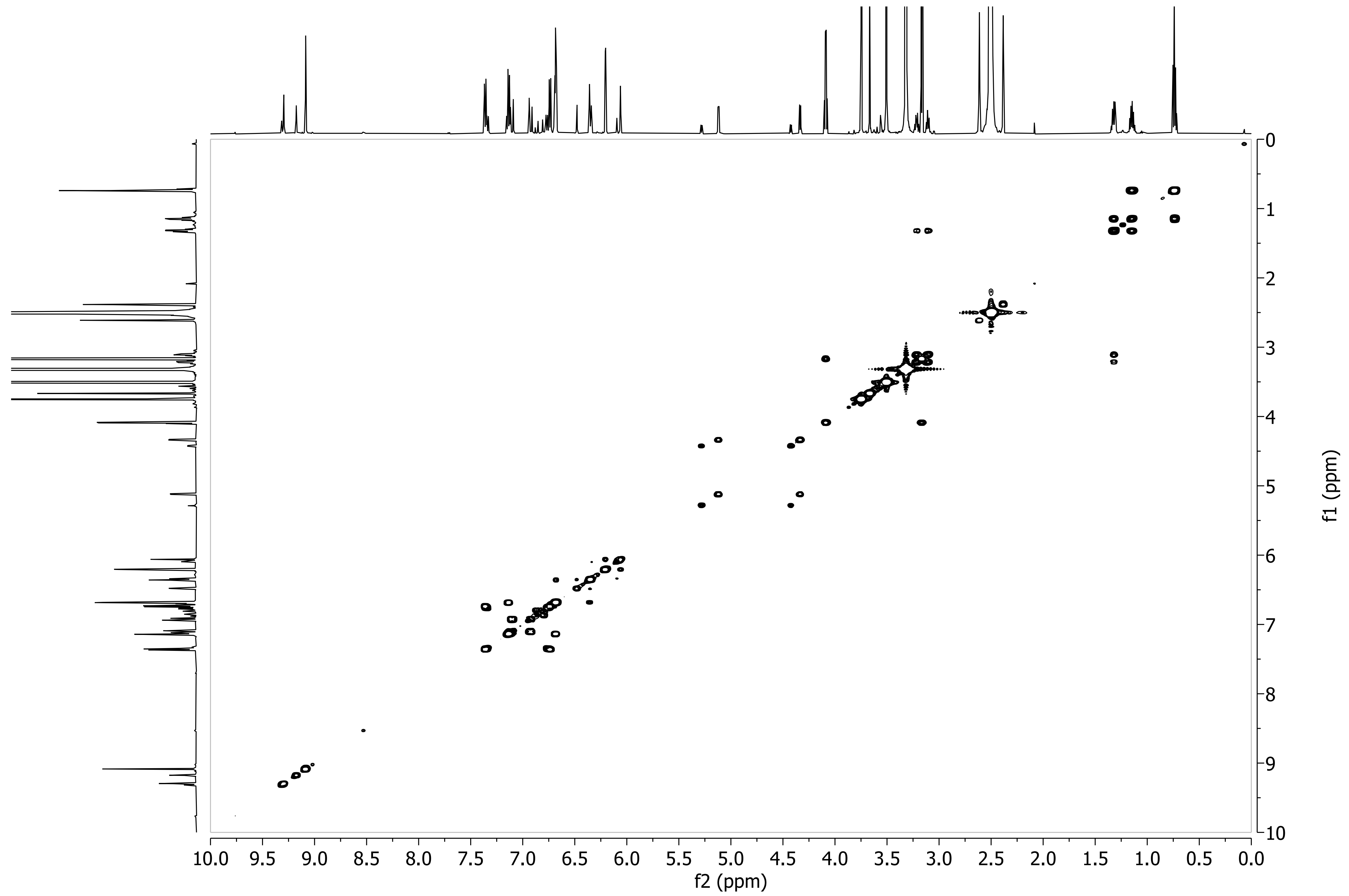

Edited-HSQC NMR spectrum of compound **35** in DMSO-d_6

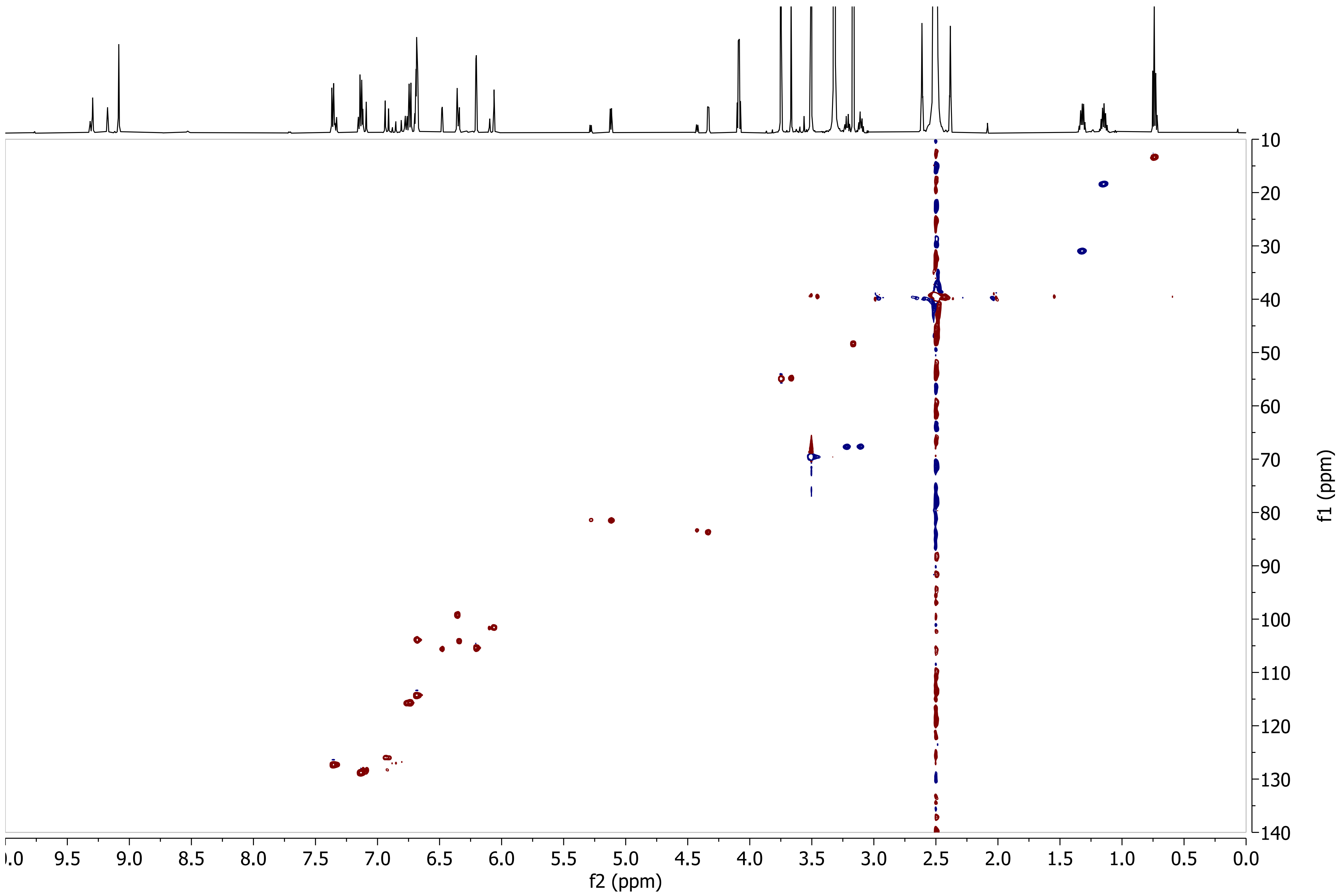

HMBC NMR spectrum of compound **35** in DMSO-d_6

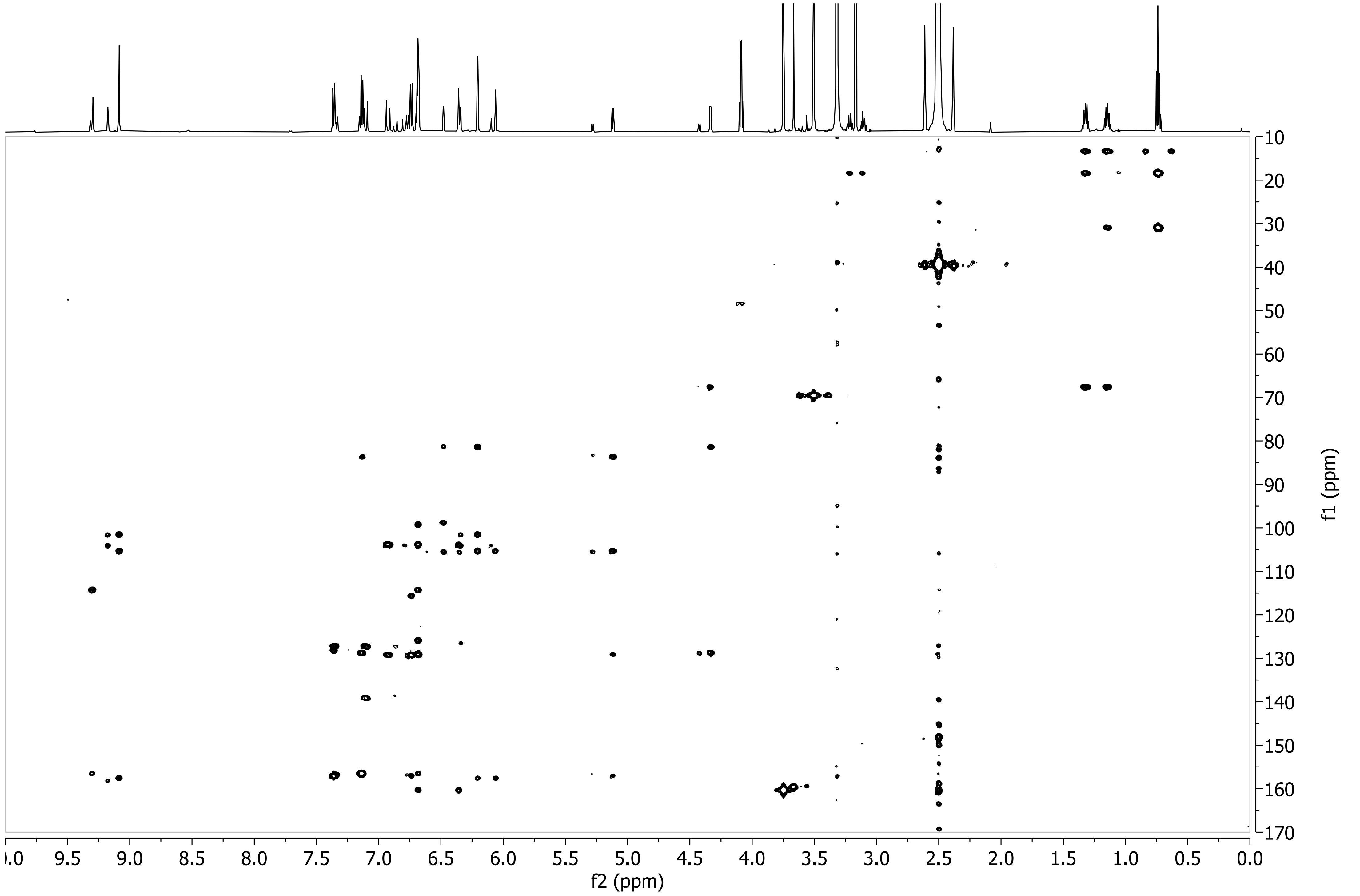

ROESY NMR spectrum of compound **35** in DMSO-d_6

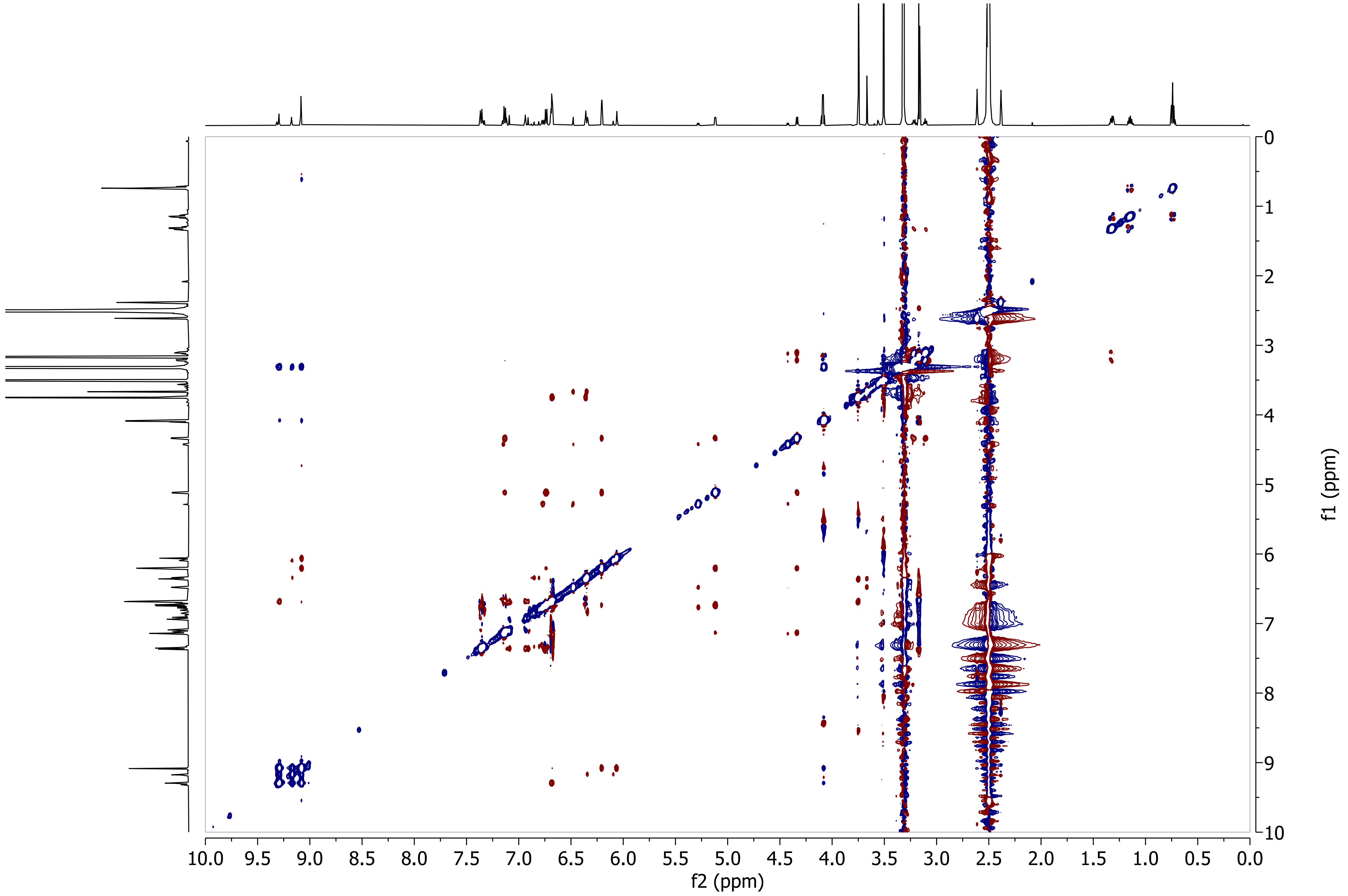

^{1}H NMR spectrum of compound **36** in DMSO-d_6

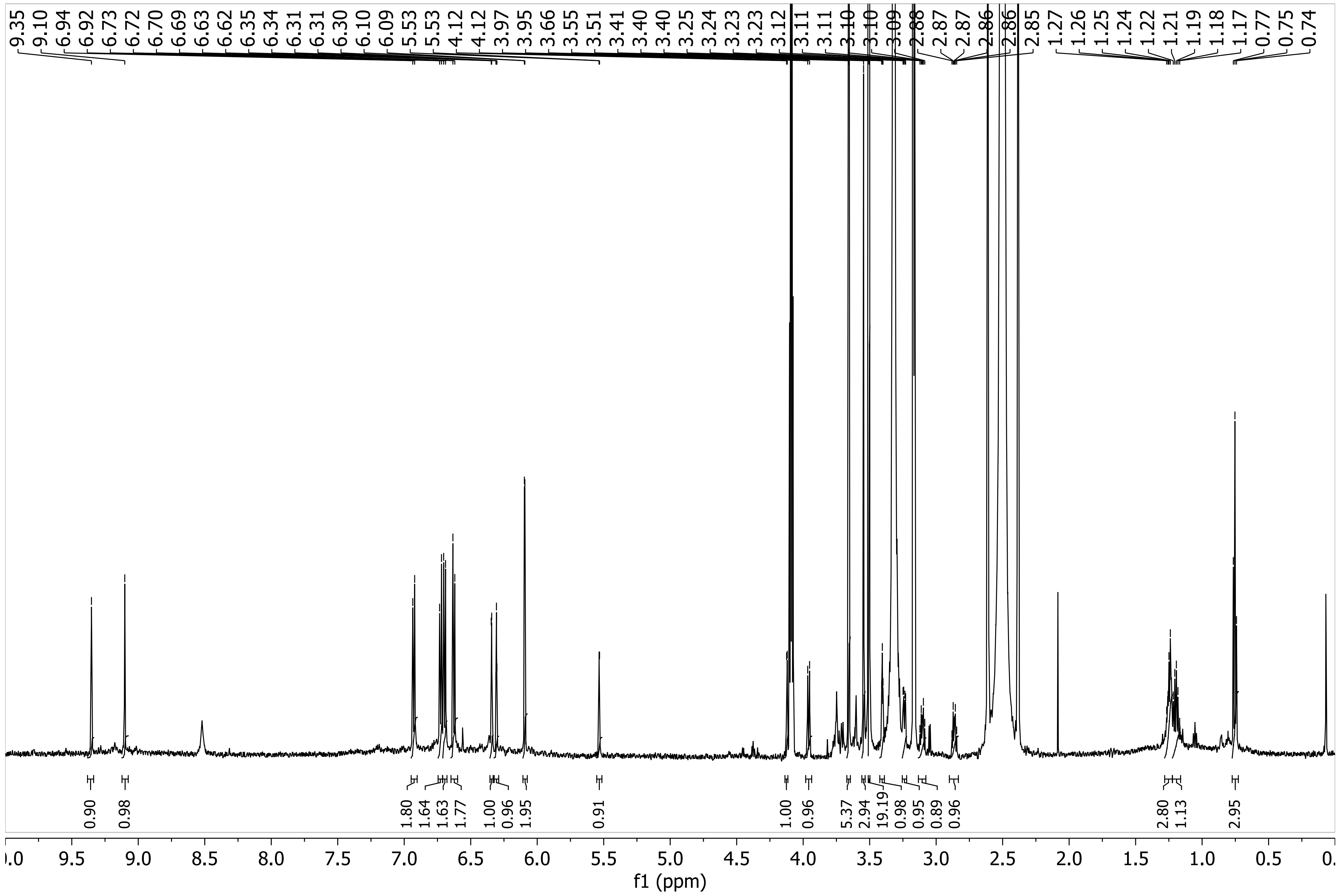

COSY NMR spectrum of compound **36** in DMSO-d_6

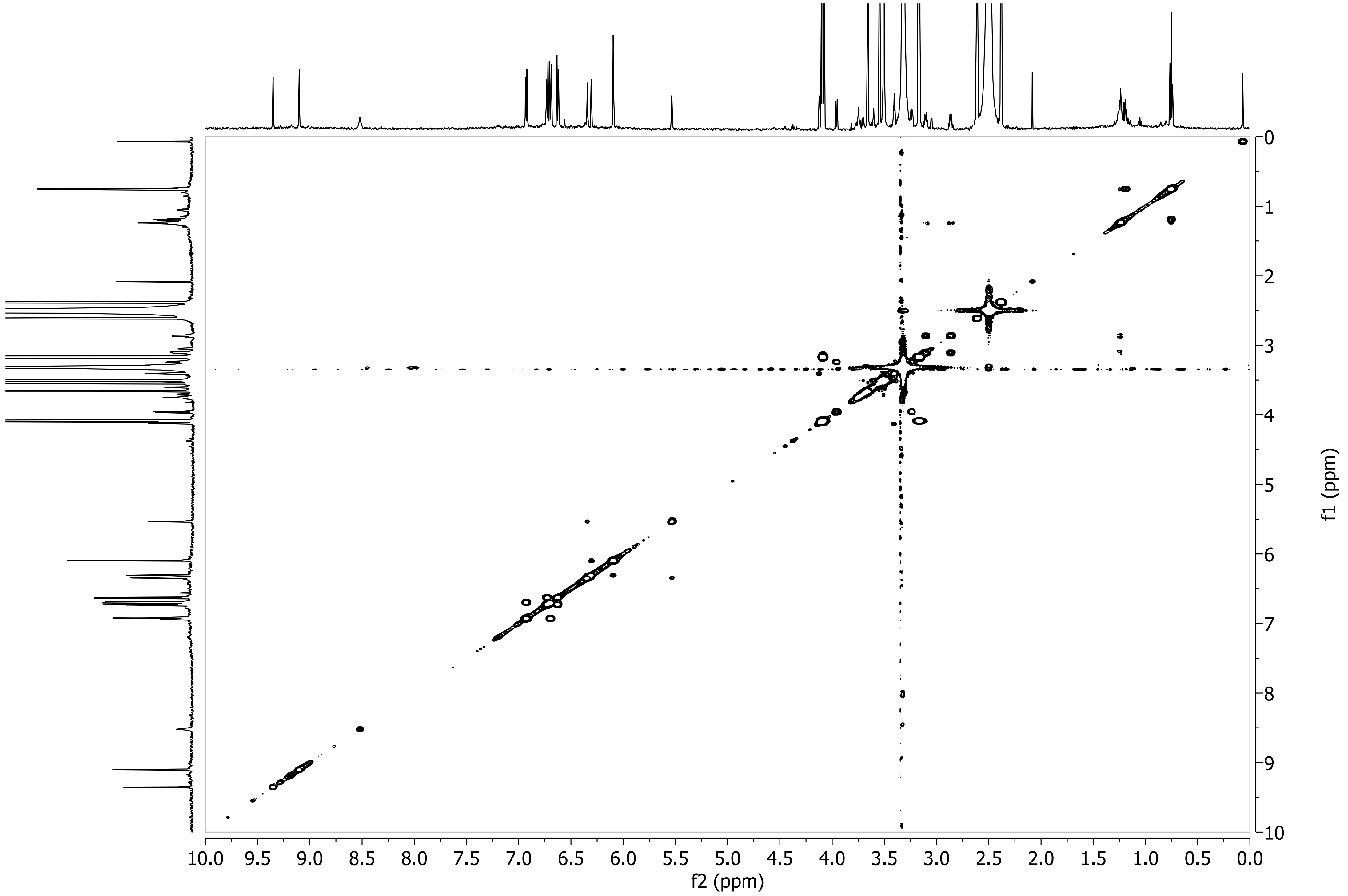

Edited-HSQC NMR spectrum of compound **36** in DMSO-d_6

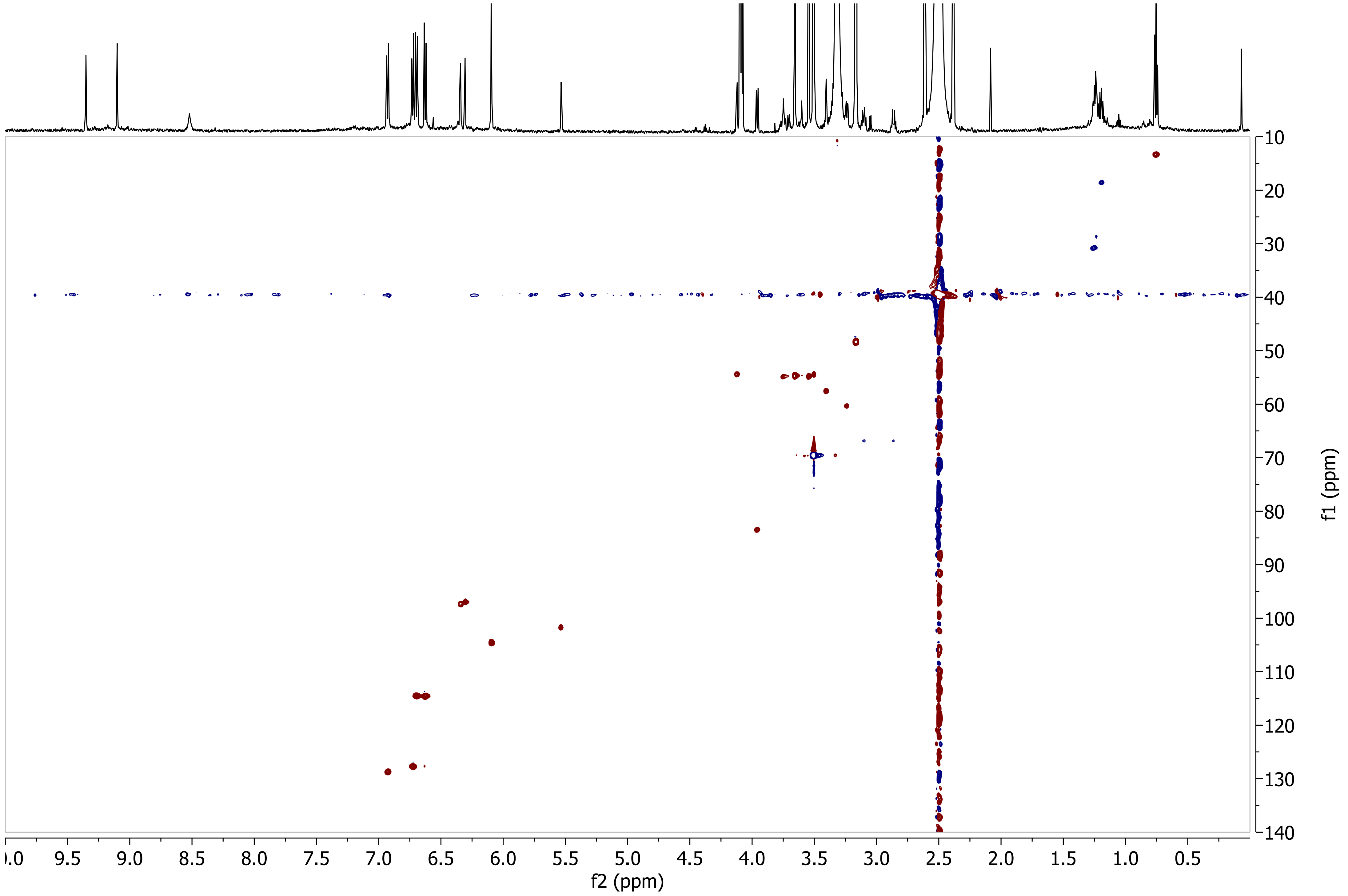

ROESY NMR spectrum of compound **36** in DMSO-d_6

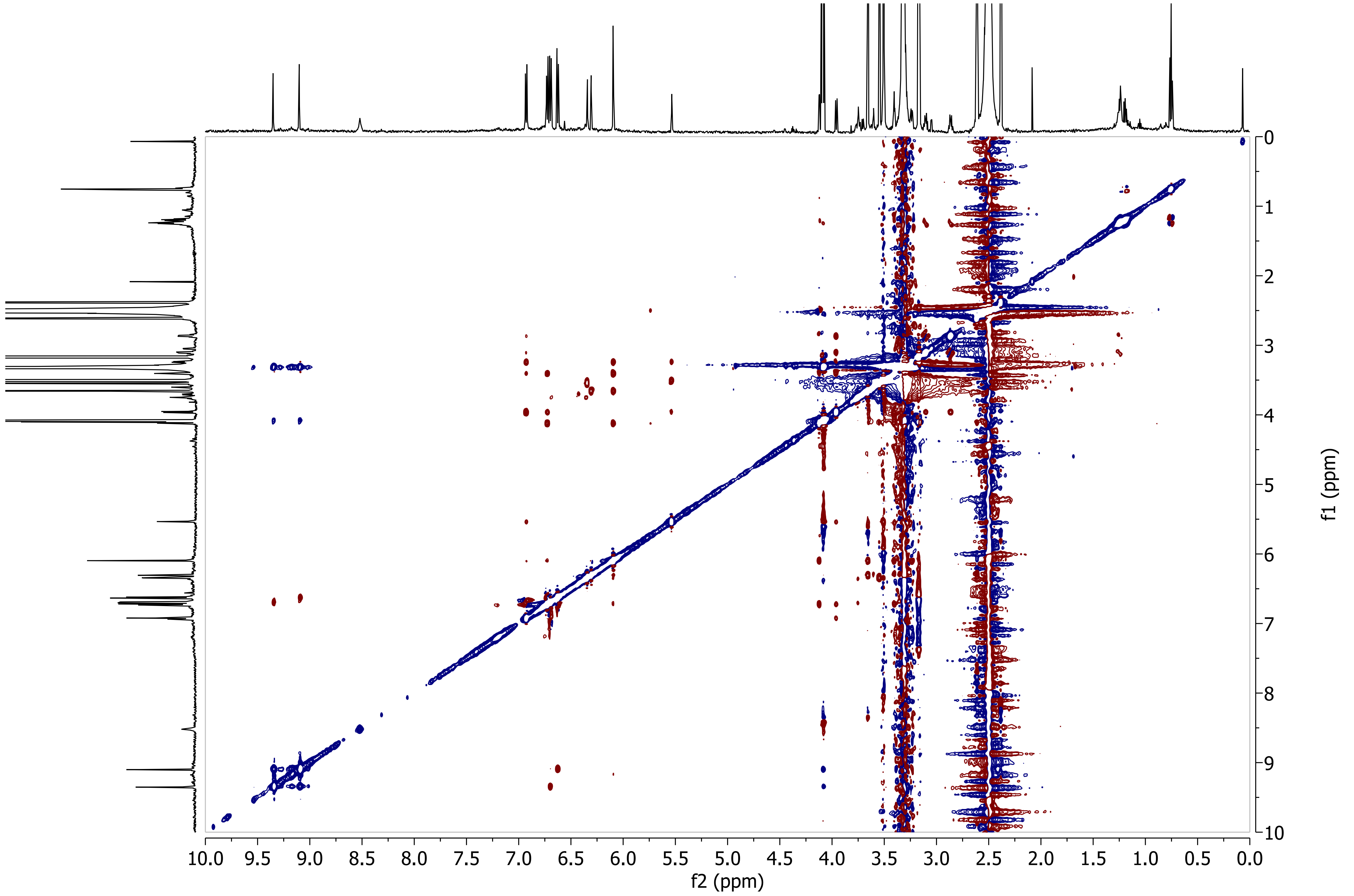

^{1}H NMR spectrum of compound **37** in DMSO-d_6

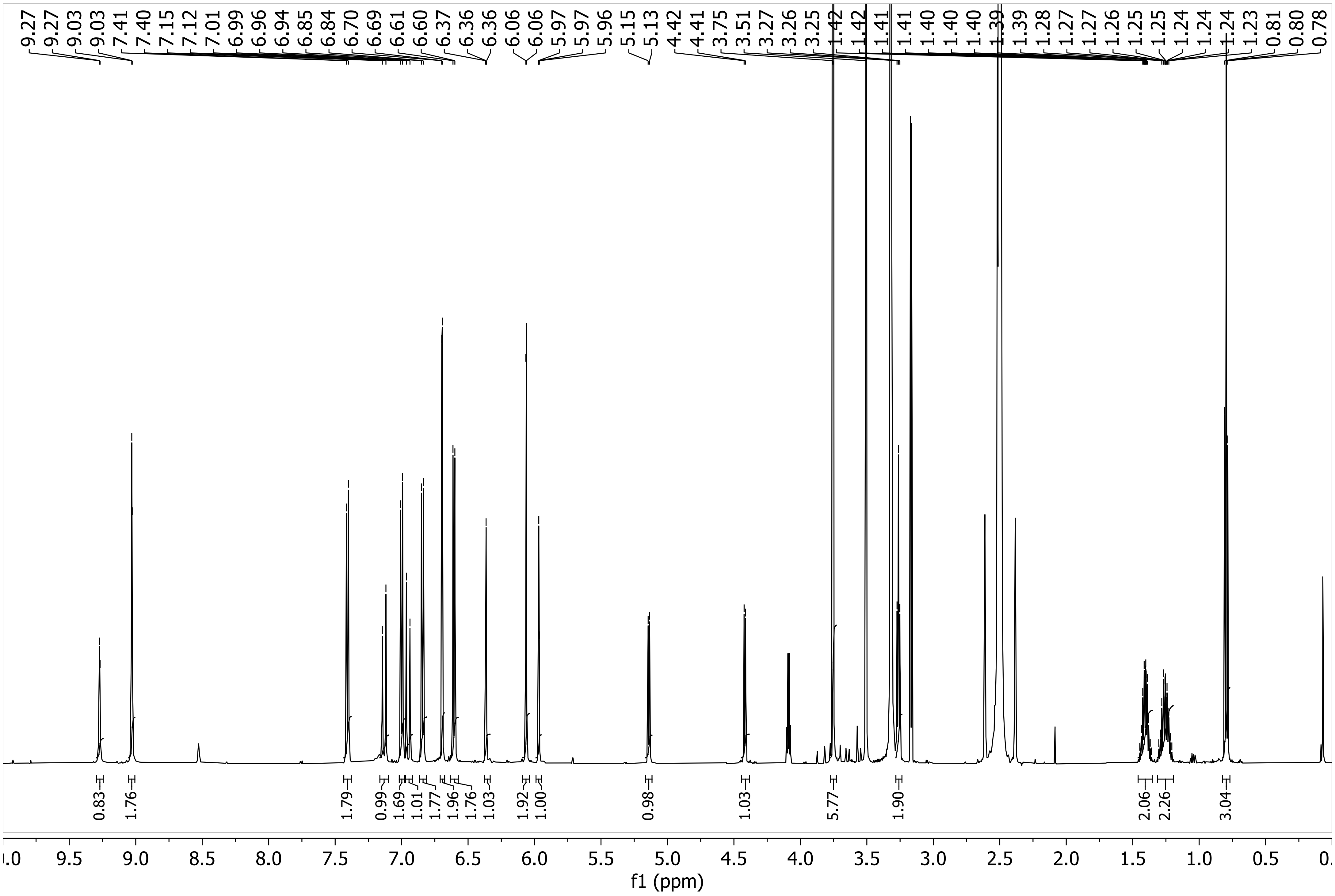

COSY NMR spectrum of compound **37** in DMSO-d_6

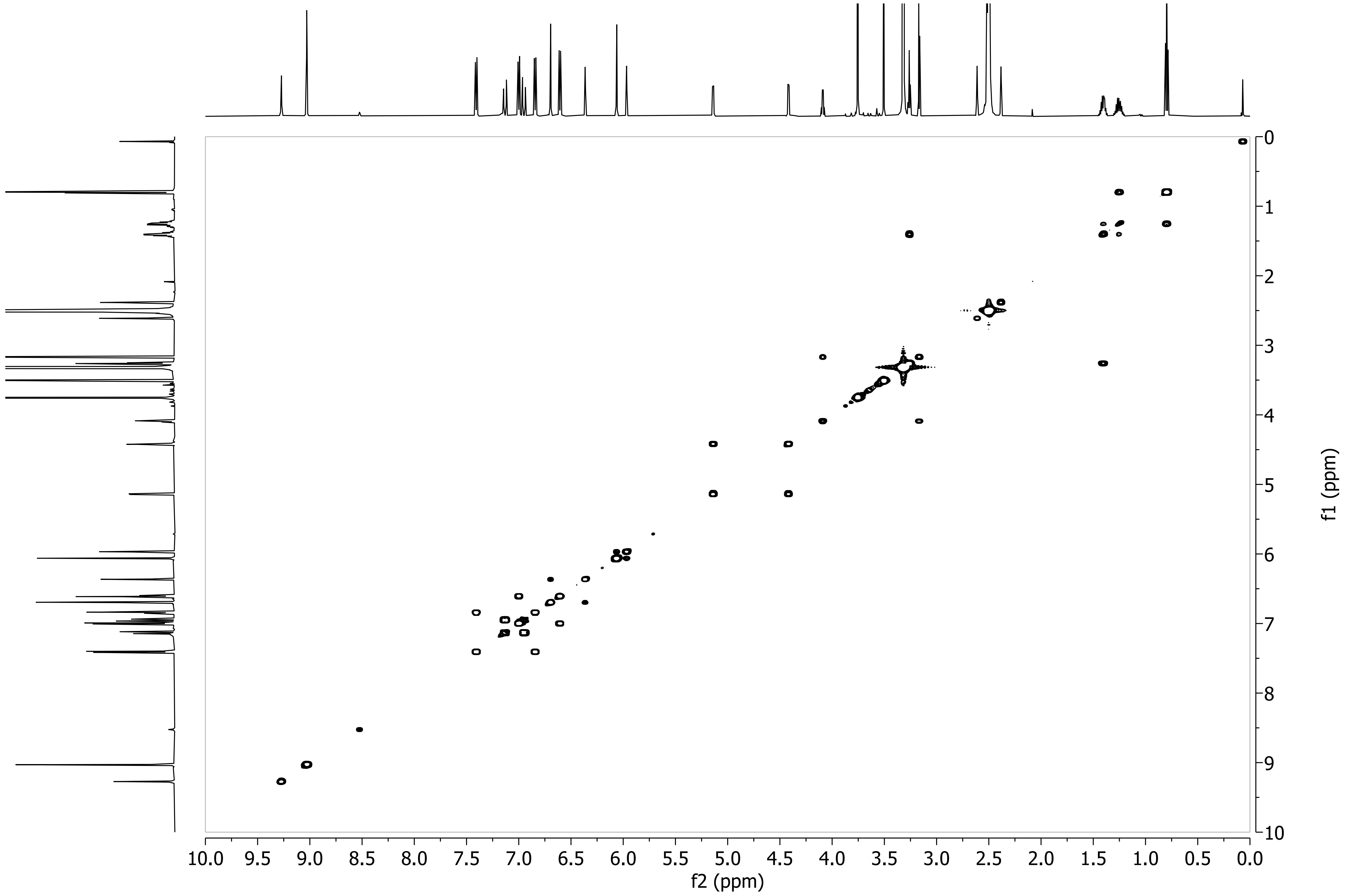

Edited-HSQC NMR spectrum of compound **37** in DMSO-d_6

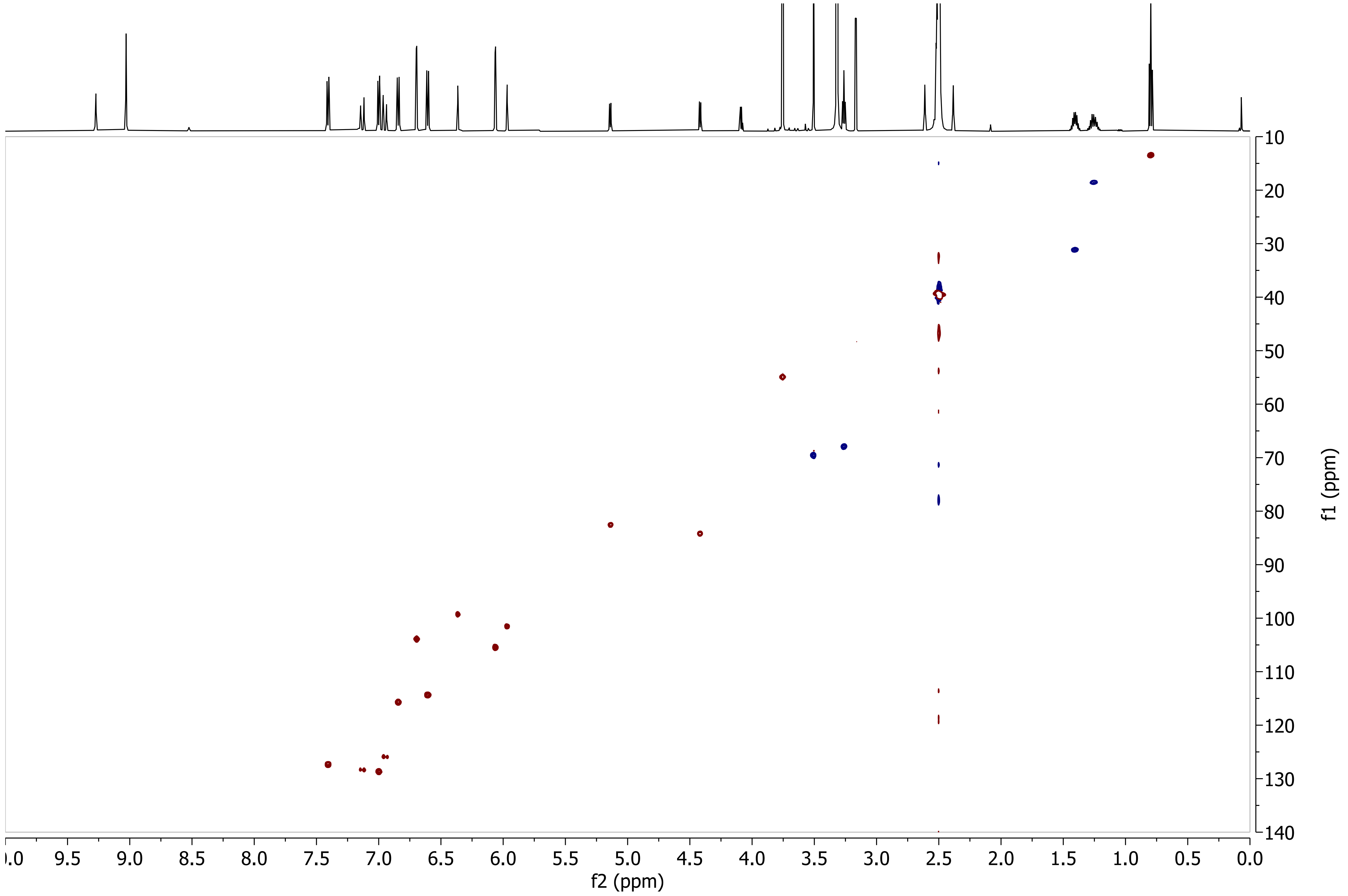

HMBC NMR spectrum of compound **37** in DMSO-d_6

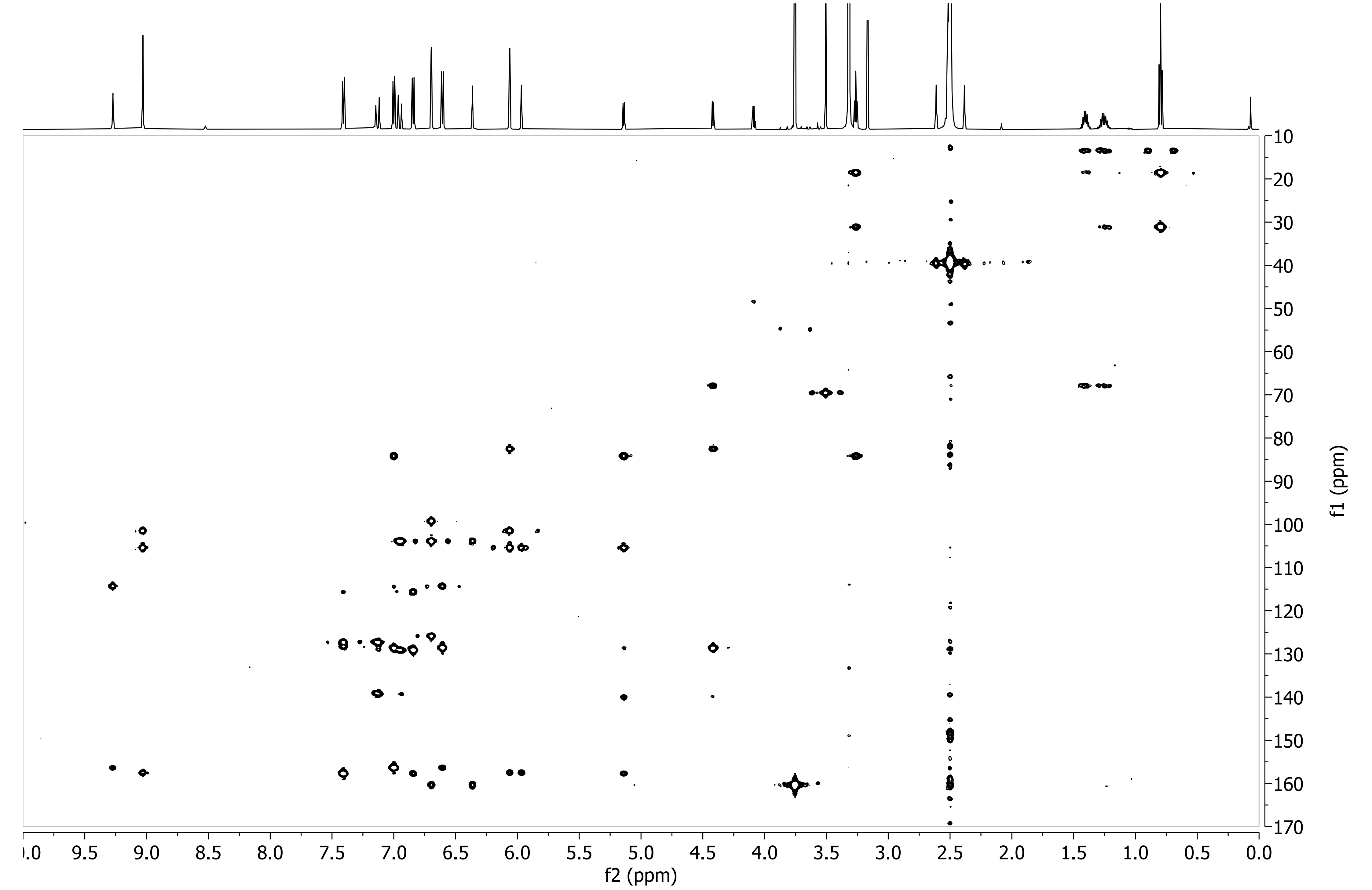

ROESY NMR spectrum of compound **37** in DMSO-d_6

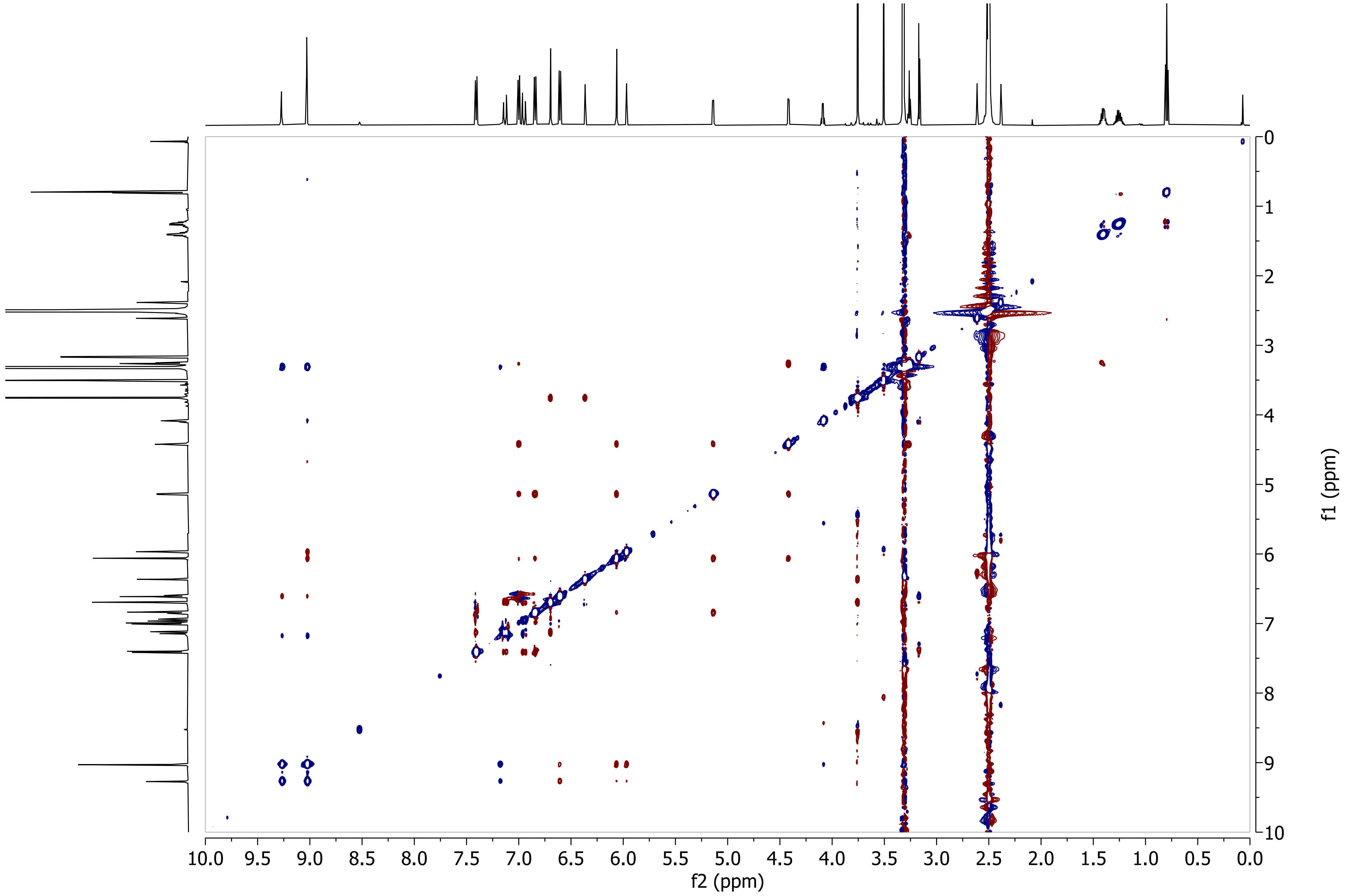

^{1}H NMR spectrum of compound **38** in DMSO-d_6

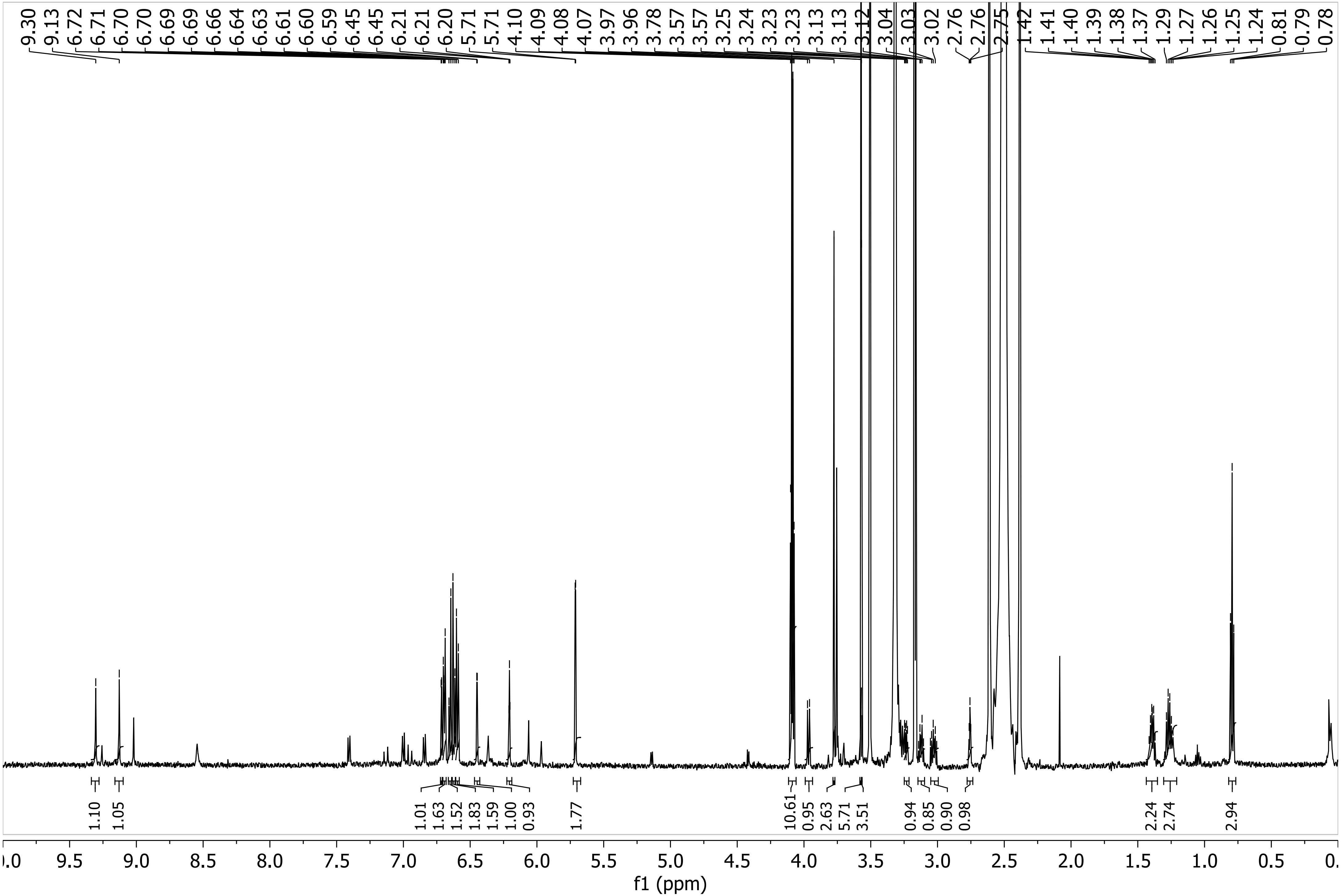

COSY NMR spectrum of compound **38** in DMSO-d_6

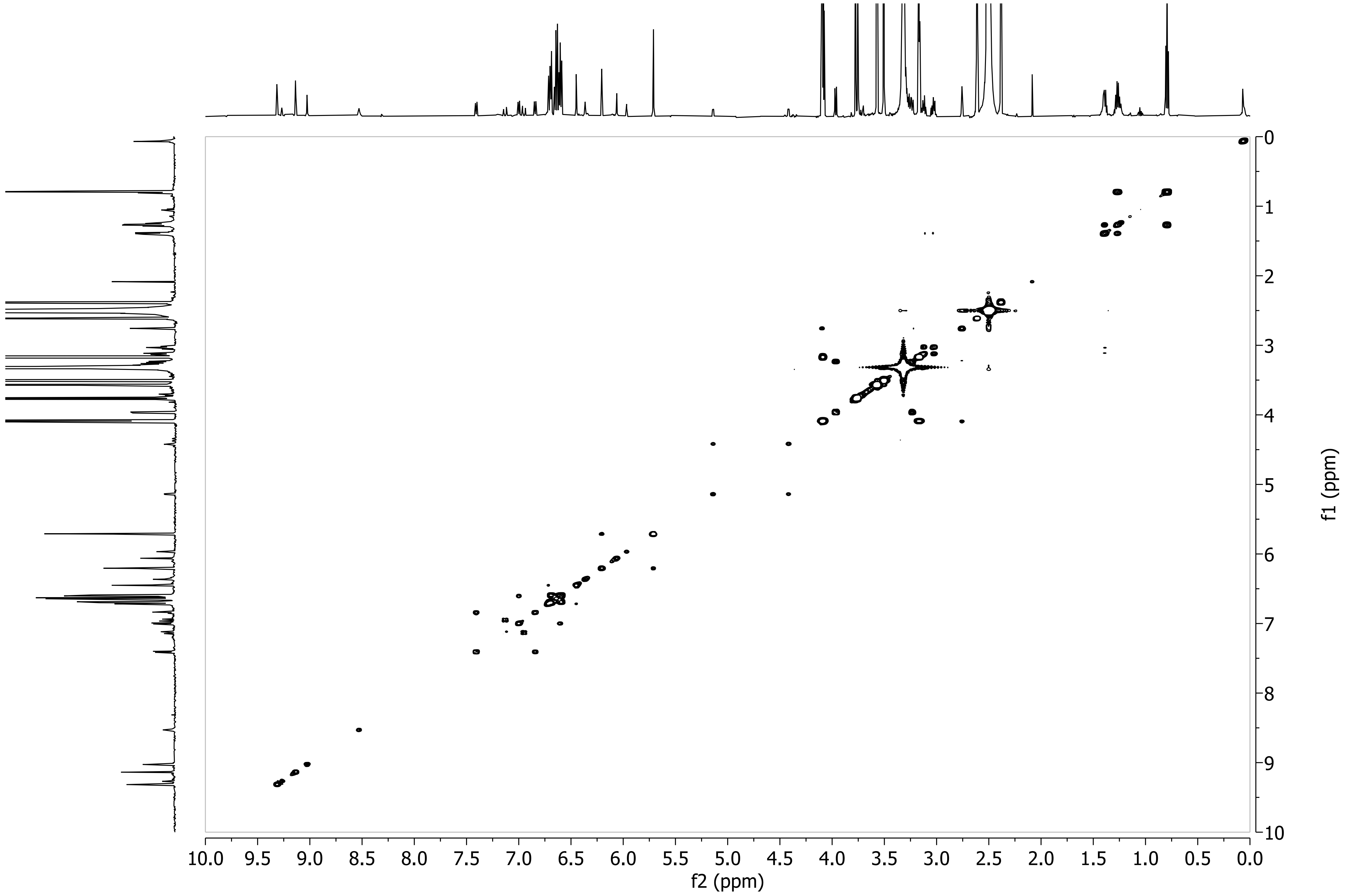

Edited-HSQC NMR spectrum of compound **38** in DMSO-d_6

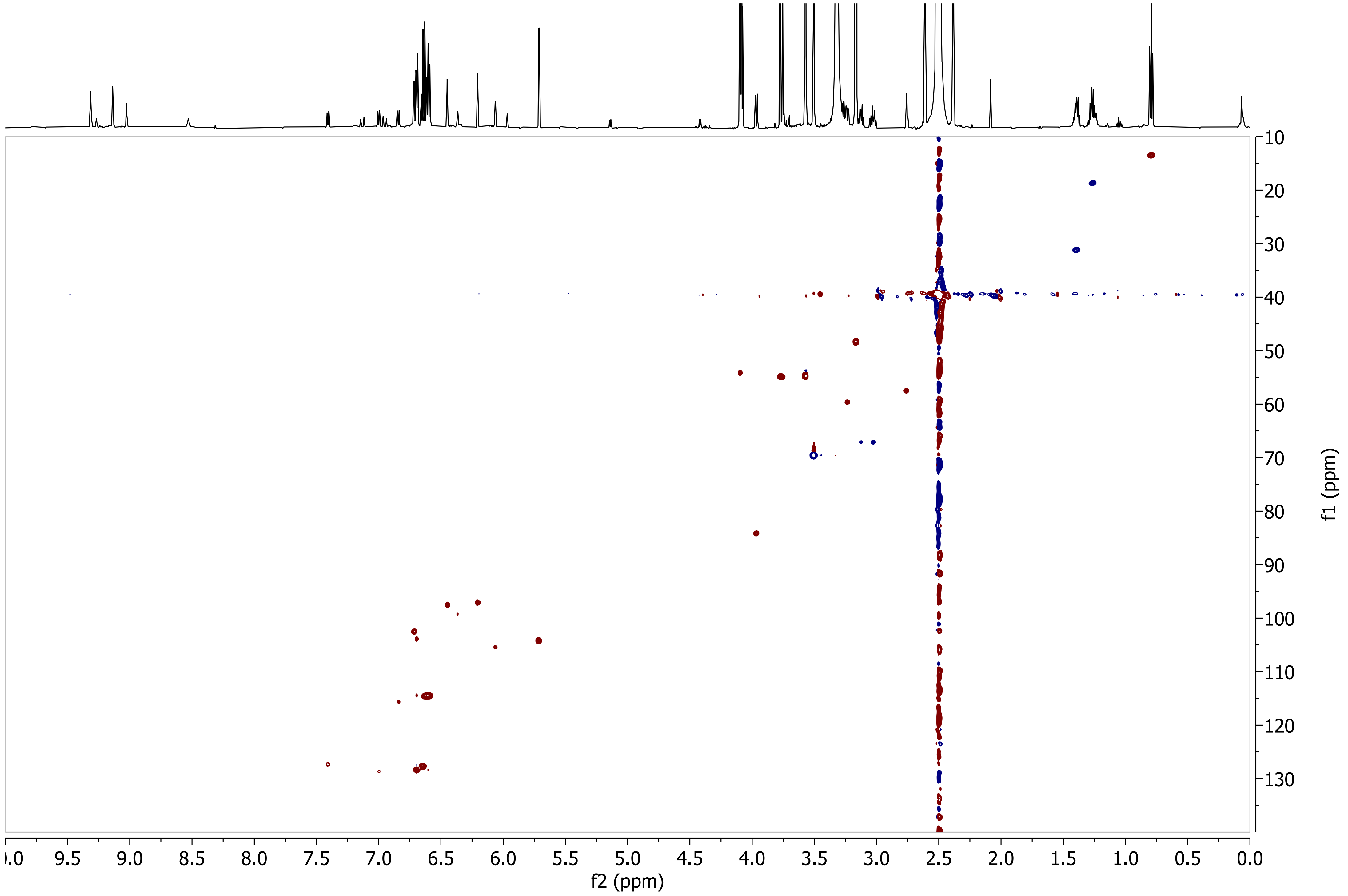

ROESY NMR spectrum of compound **38** in DMSO-d_6

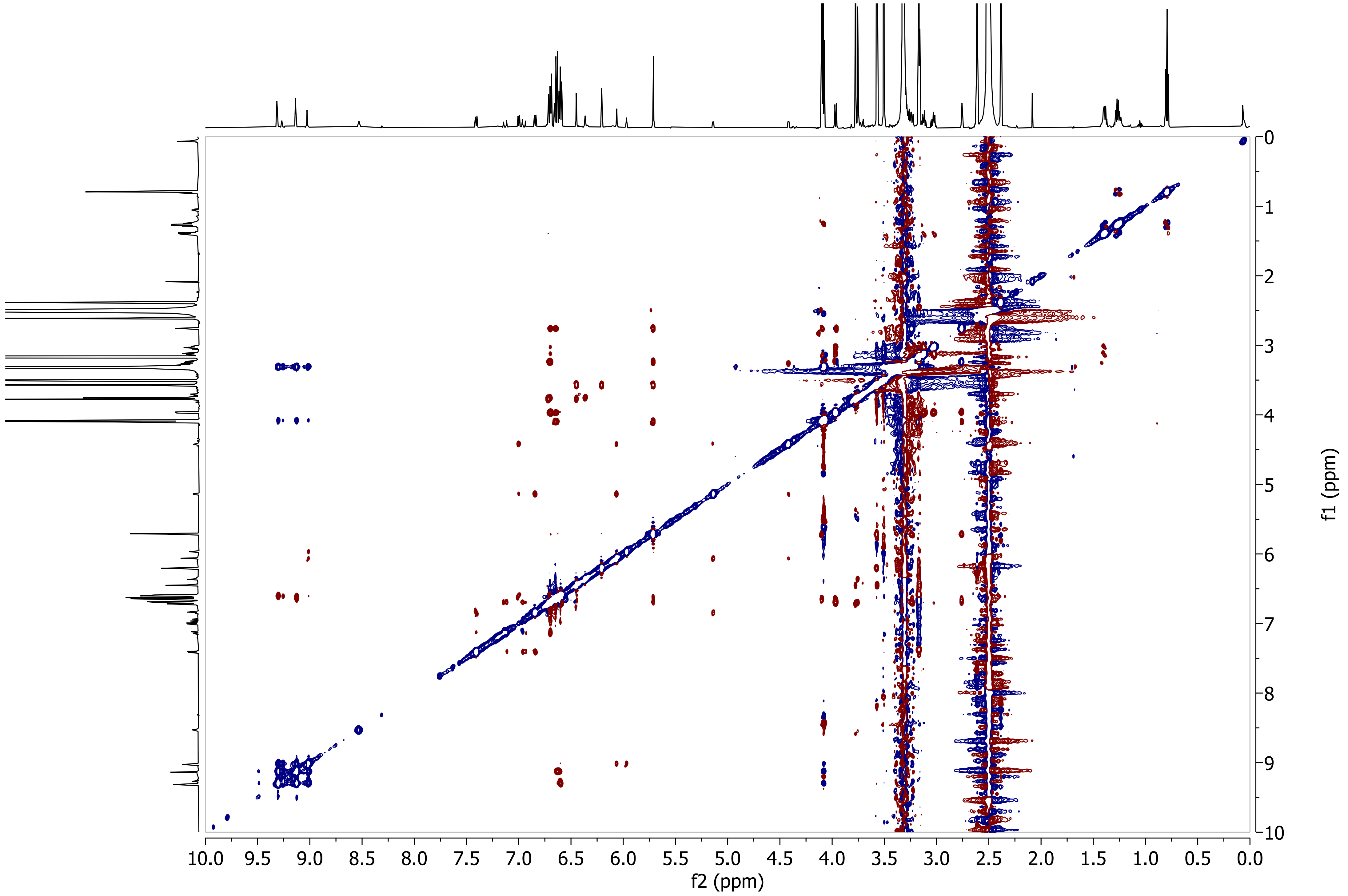

^{1}H NMR spectrum of compound **39** in DMSO-d_6

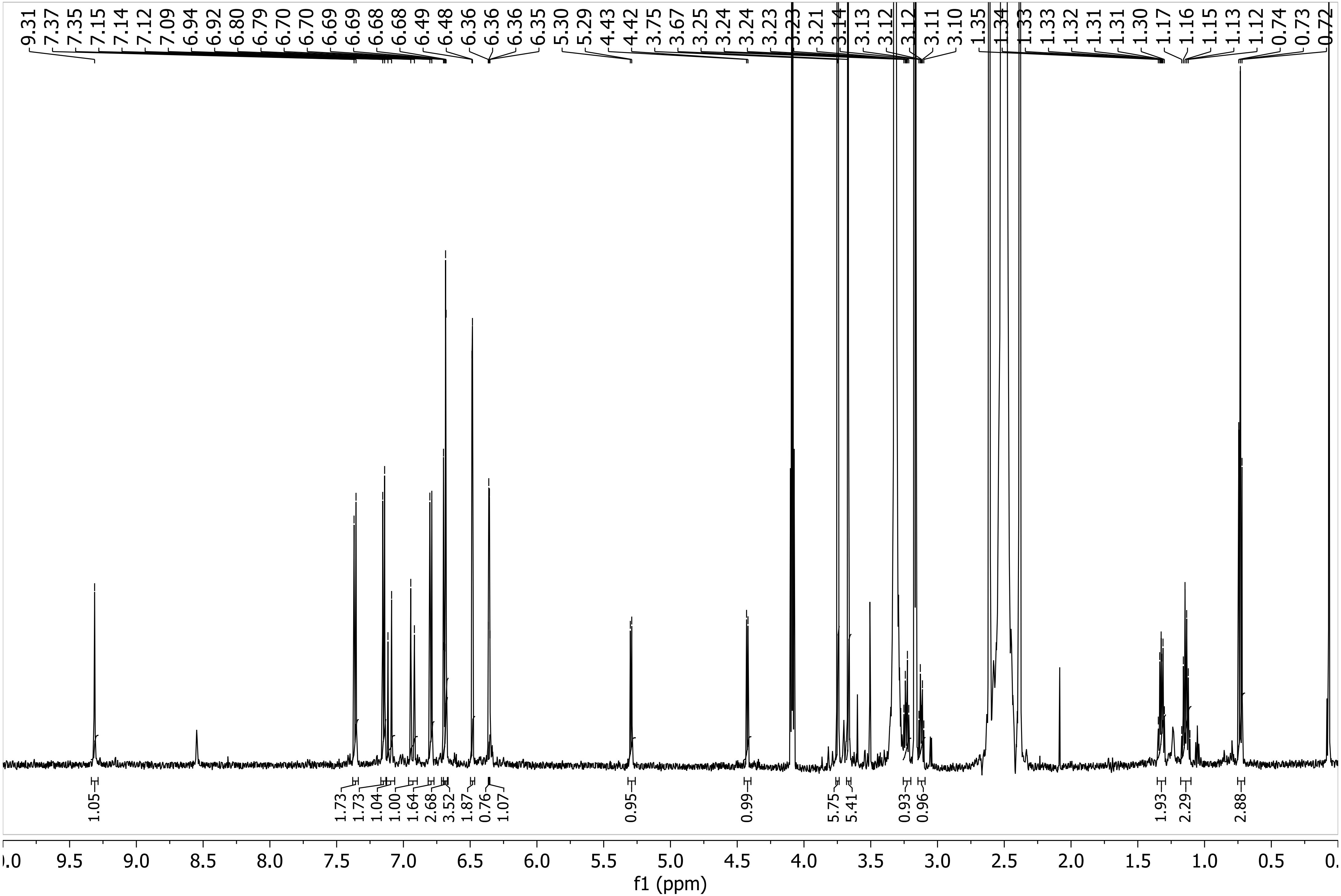

COSY NMR spectrum of compound **39** in DMSO-d_6

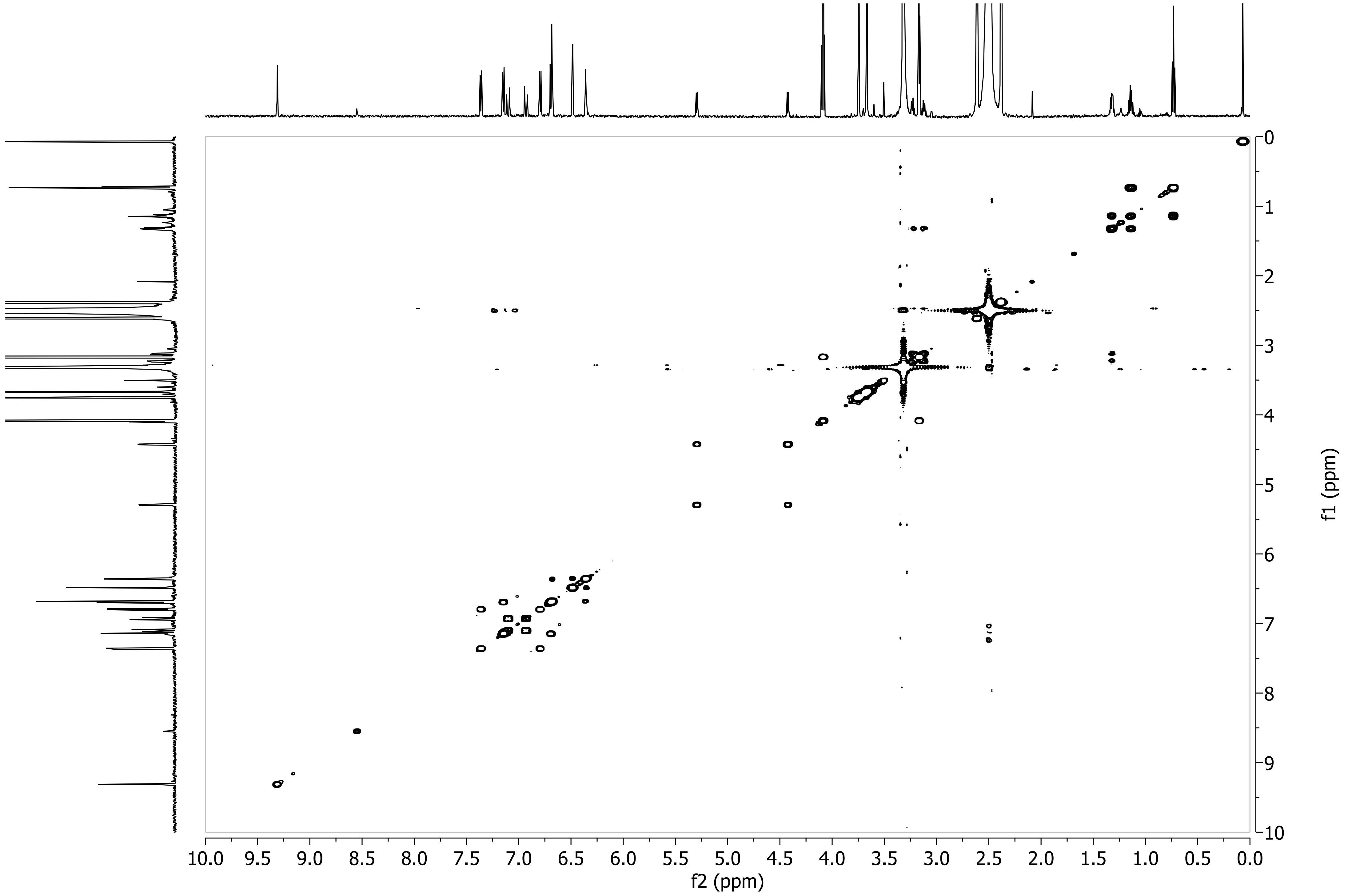

Edited-HSQC NMR spectrum of compound **39** in DMSO-d_6

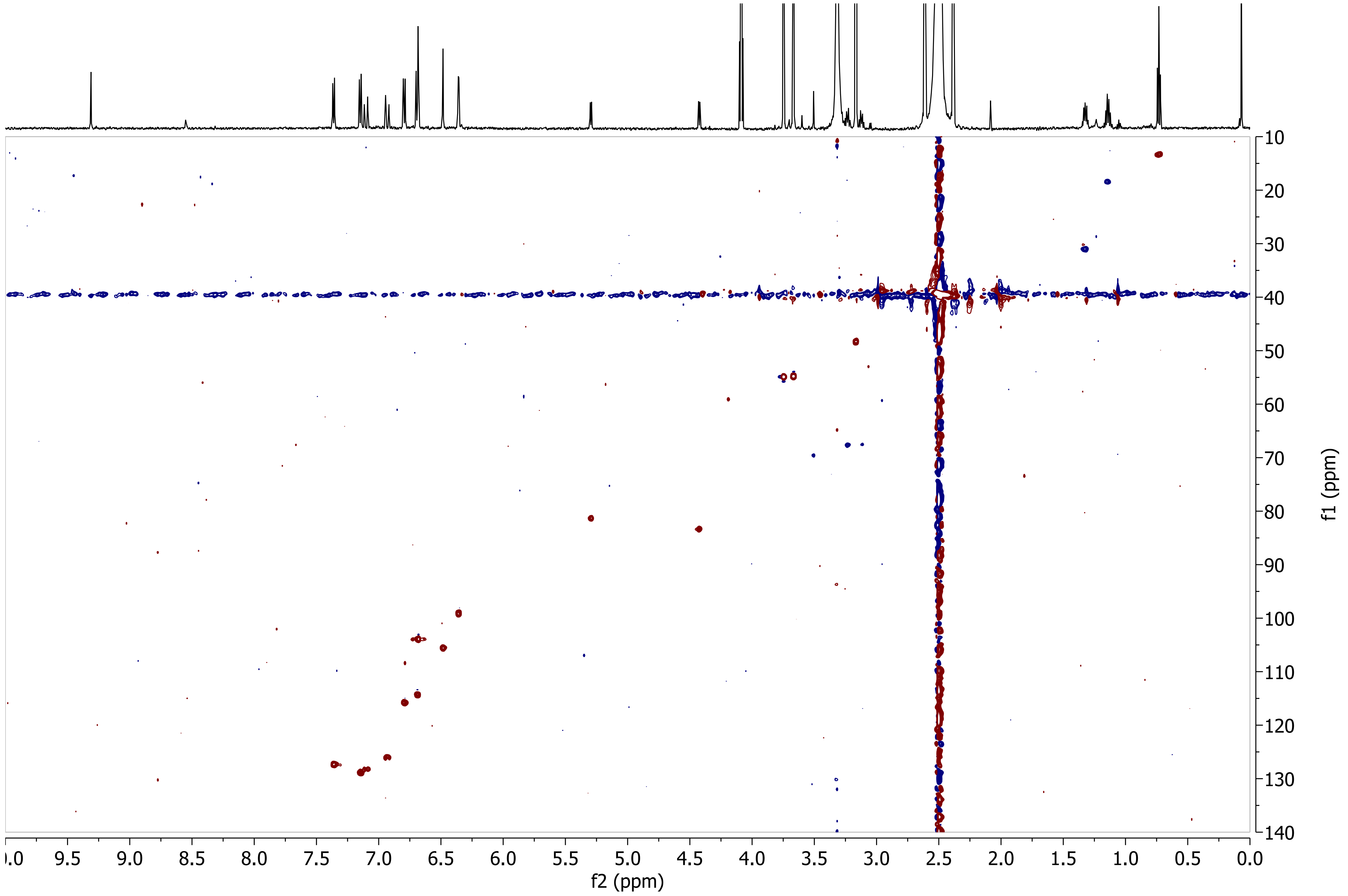

ROESY NMR spectrum of compound **39** in DMSO-d_6

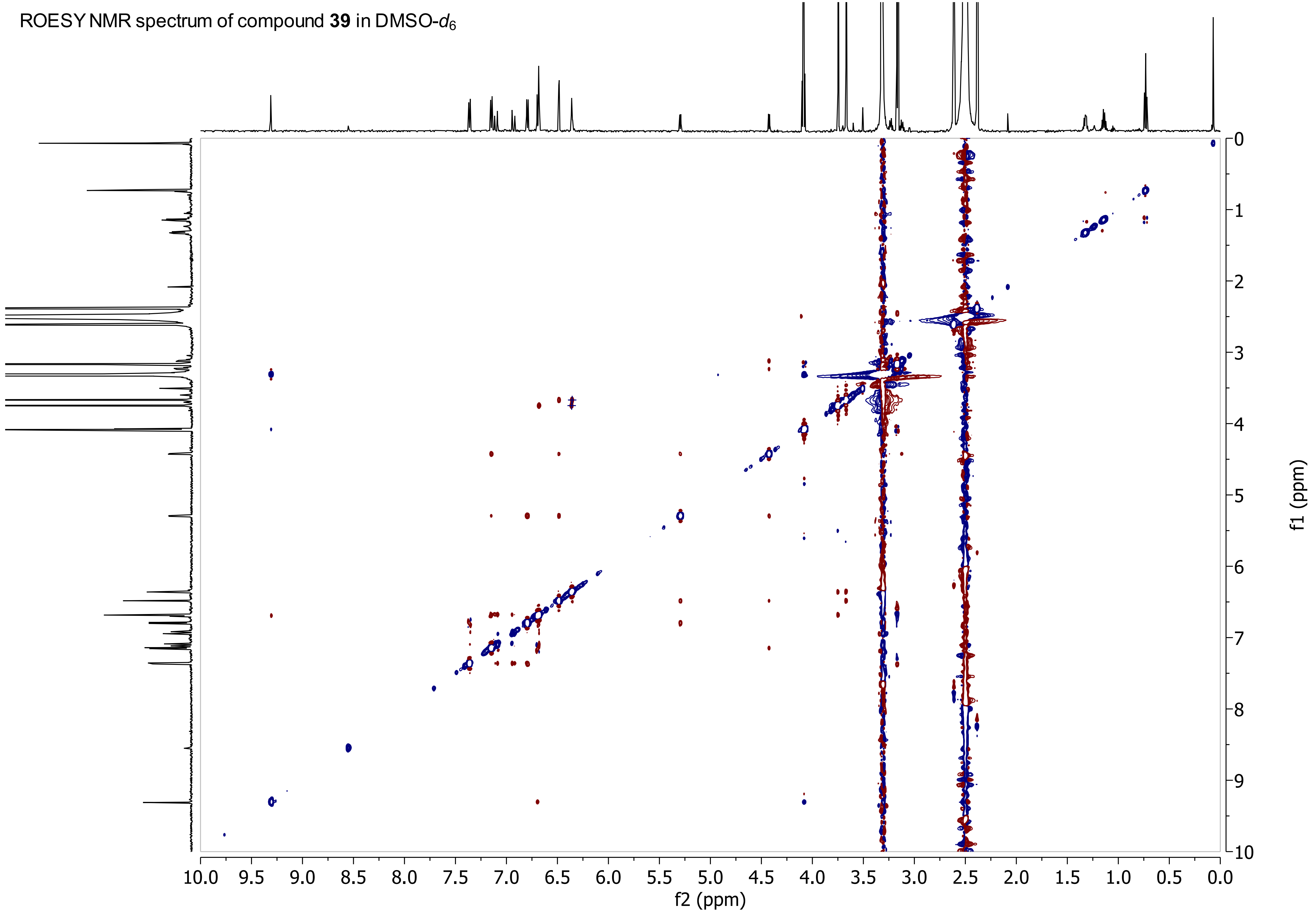

^{1}H NMR spectrum of compound **40** in DMSO-d_6

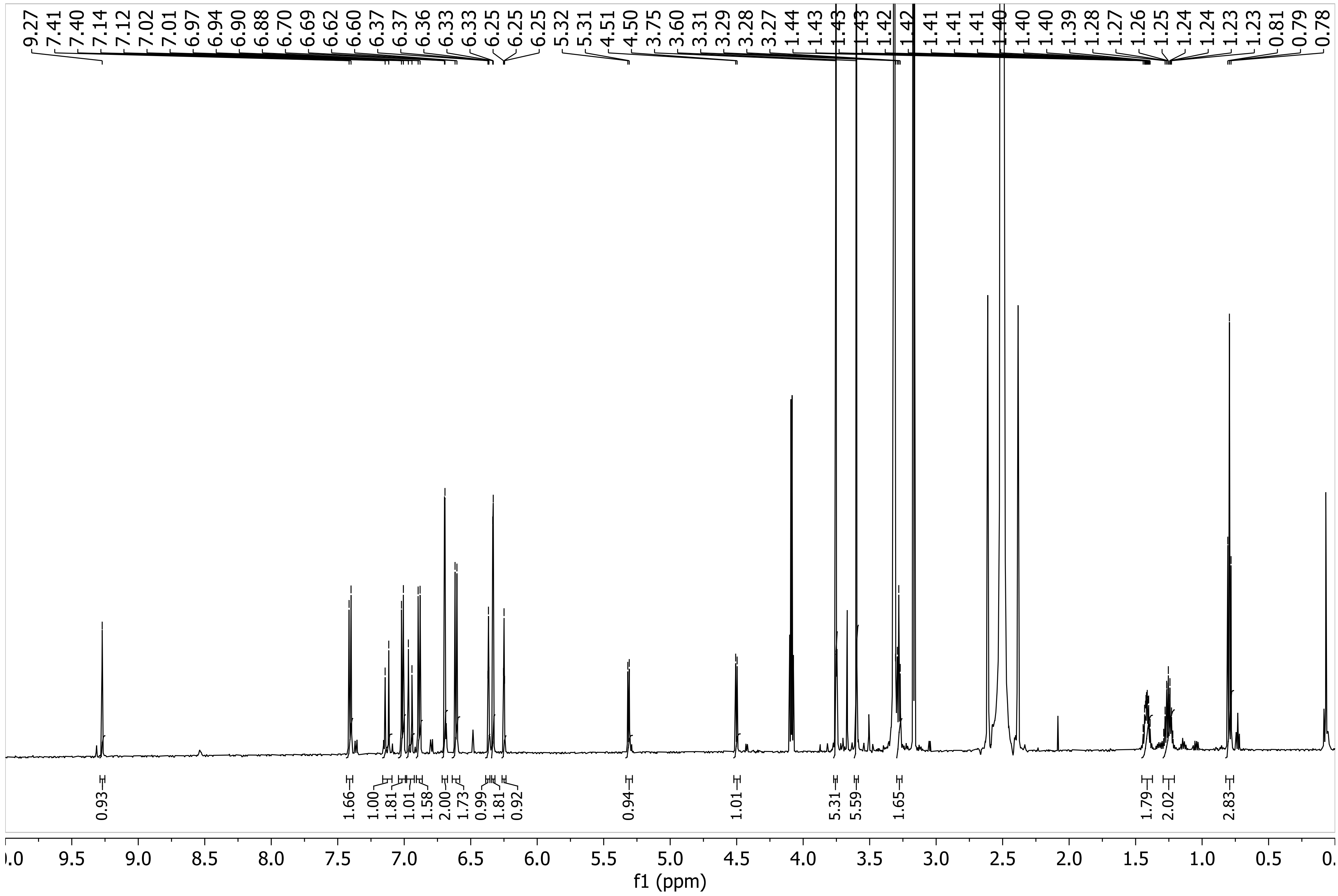

COSY NMR spectrum of compound **40** in DMSO-d_6

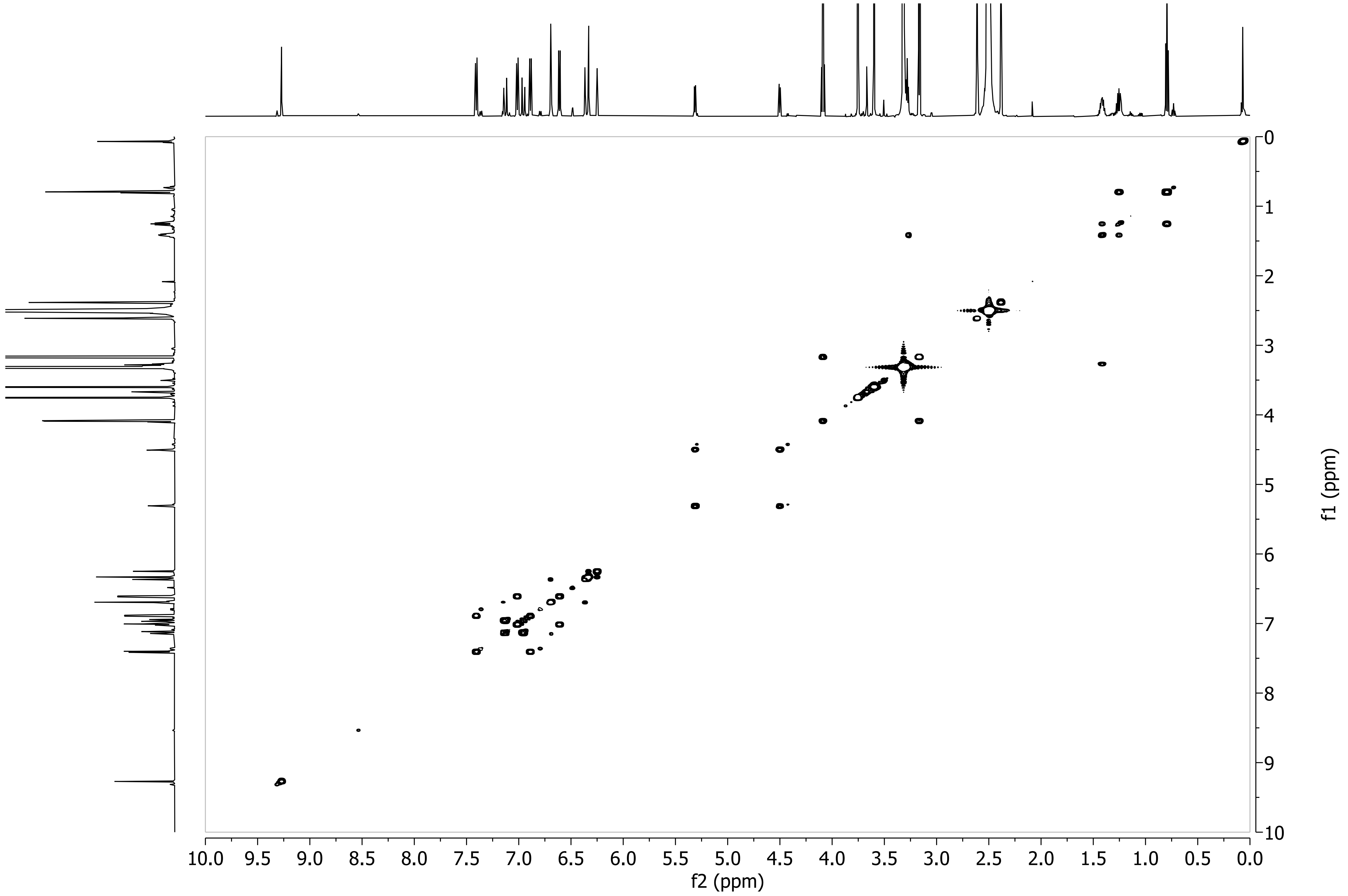

Edited-HSQC NMR spectrum of compound **40** in DMSO-d_6

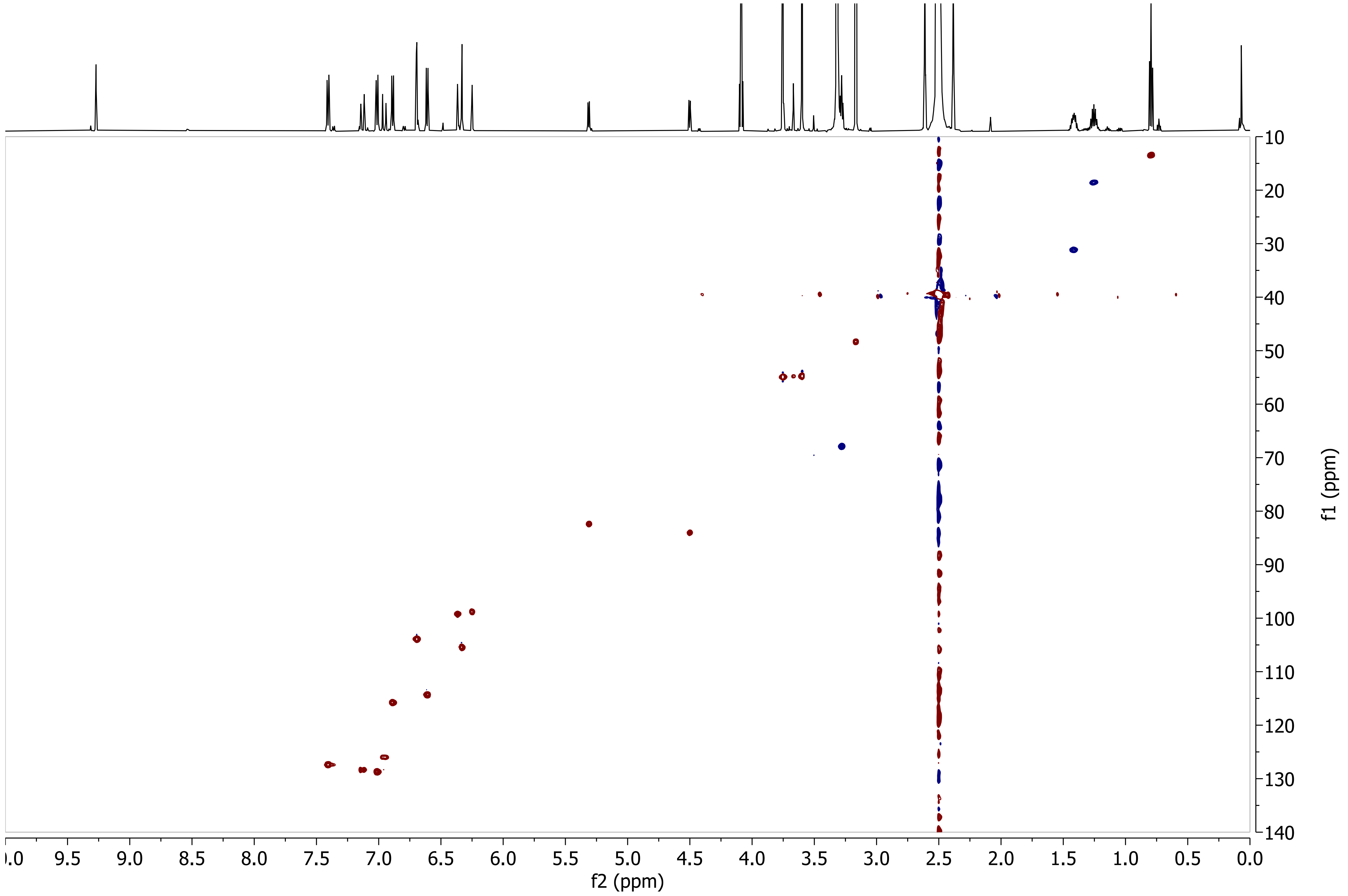

HMBC NMR spectrum of compound **40** in DMSO-d_6

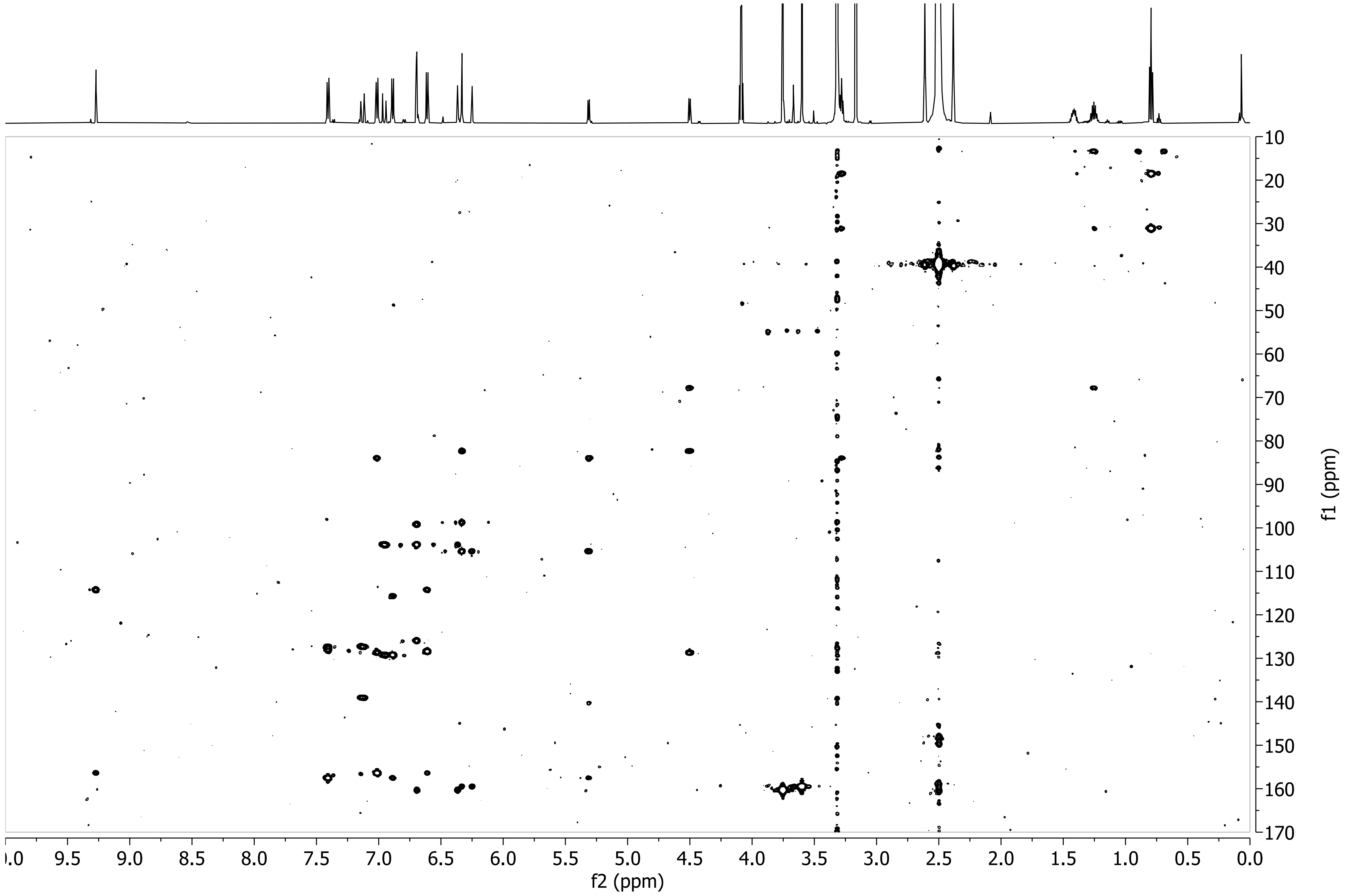

ROESY NMR spectrum of compound **40** in DMSO-d_6

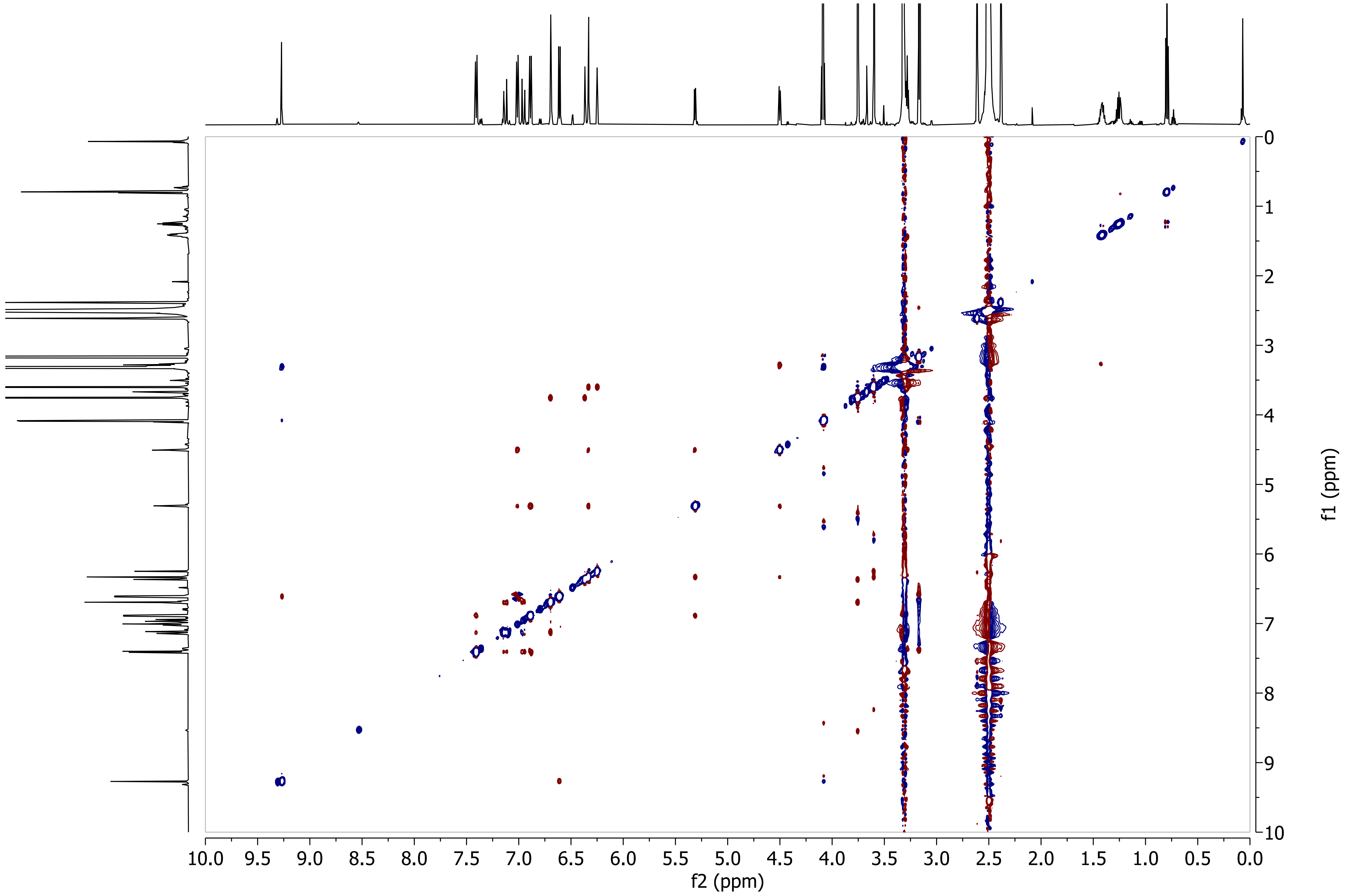

^{1}H NMR spectrum of compound **41** in DMSO-d_6

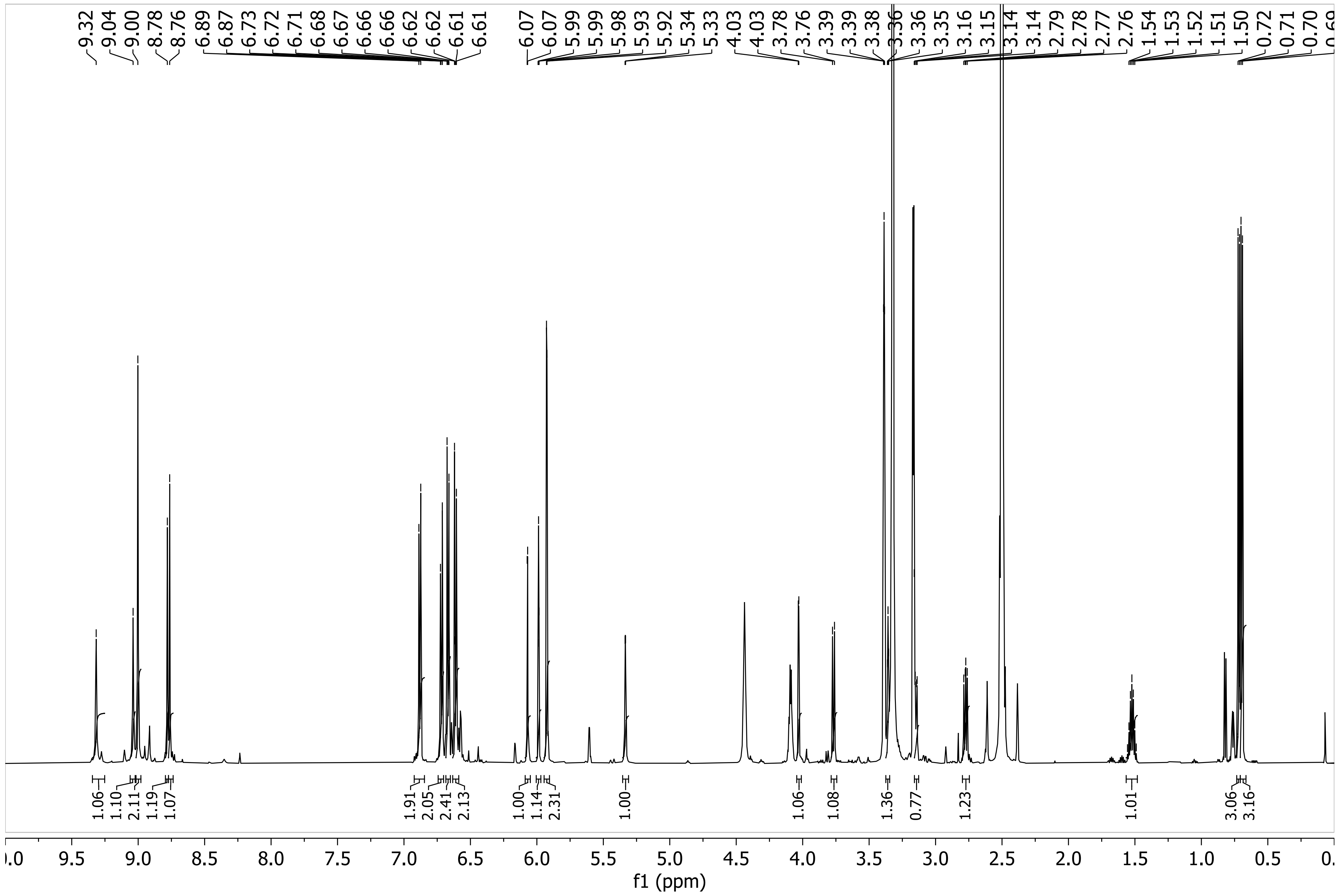

COSY NMR spectrum of compound **41** in DMSO-d_6

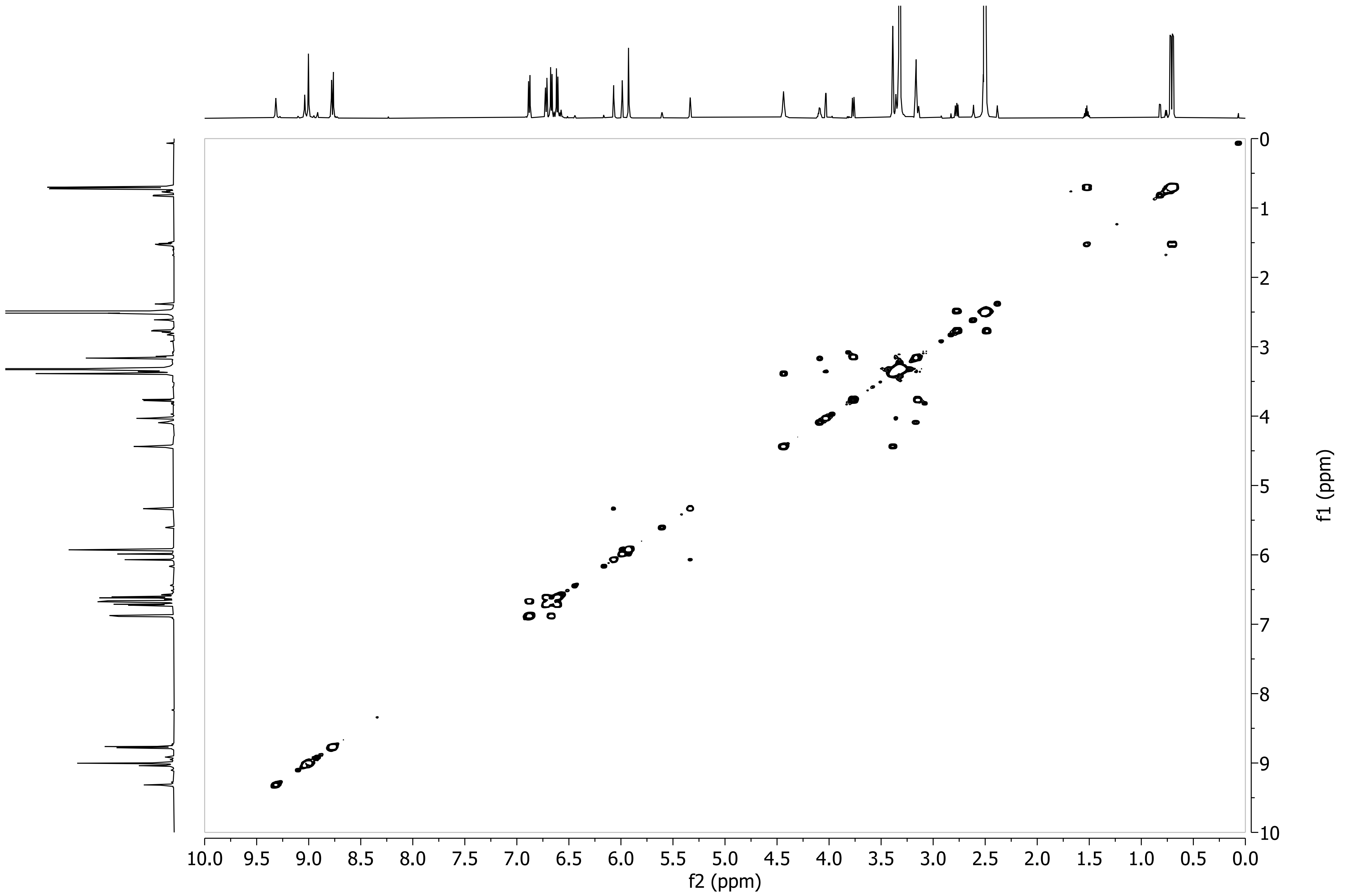

^{13}C-DEPTQ NMR spectrum of compound **41** in DMSO-d_6

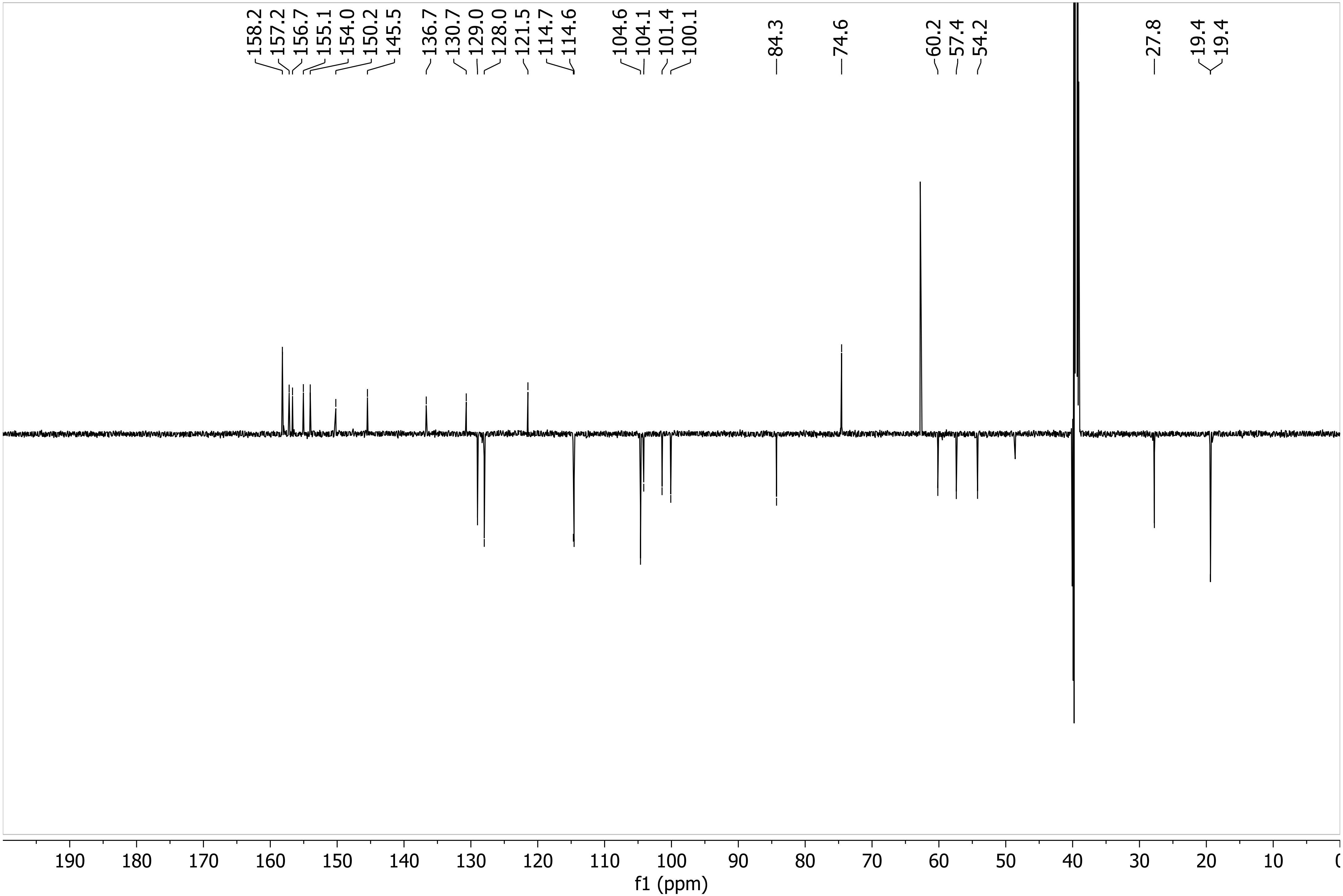

Edited-HSQC NMR spectrum of compound **41** in DMSO-d_6

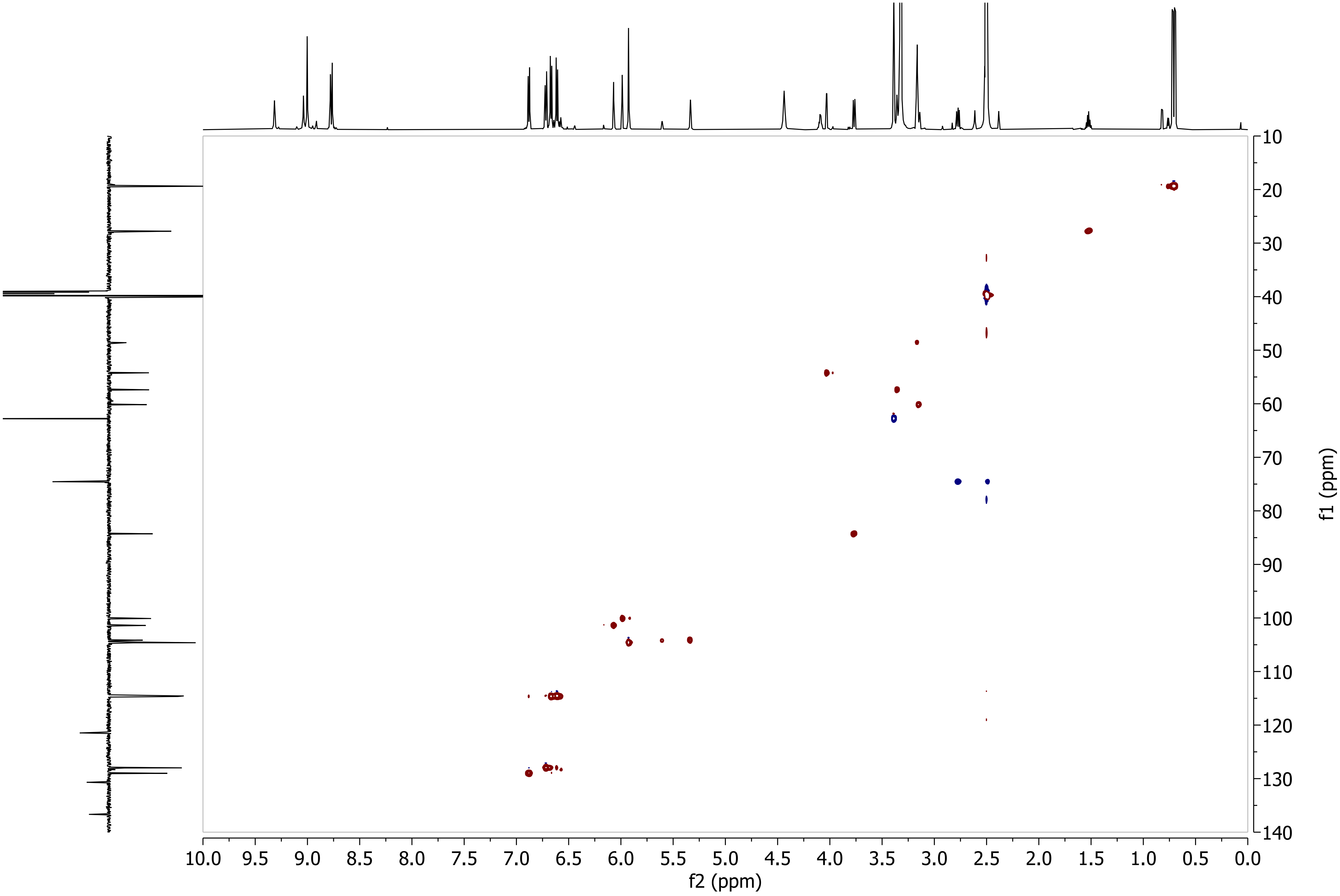

HMBC NMR spectrum of compound **41** in DMSO-d_6

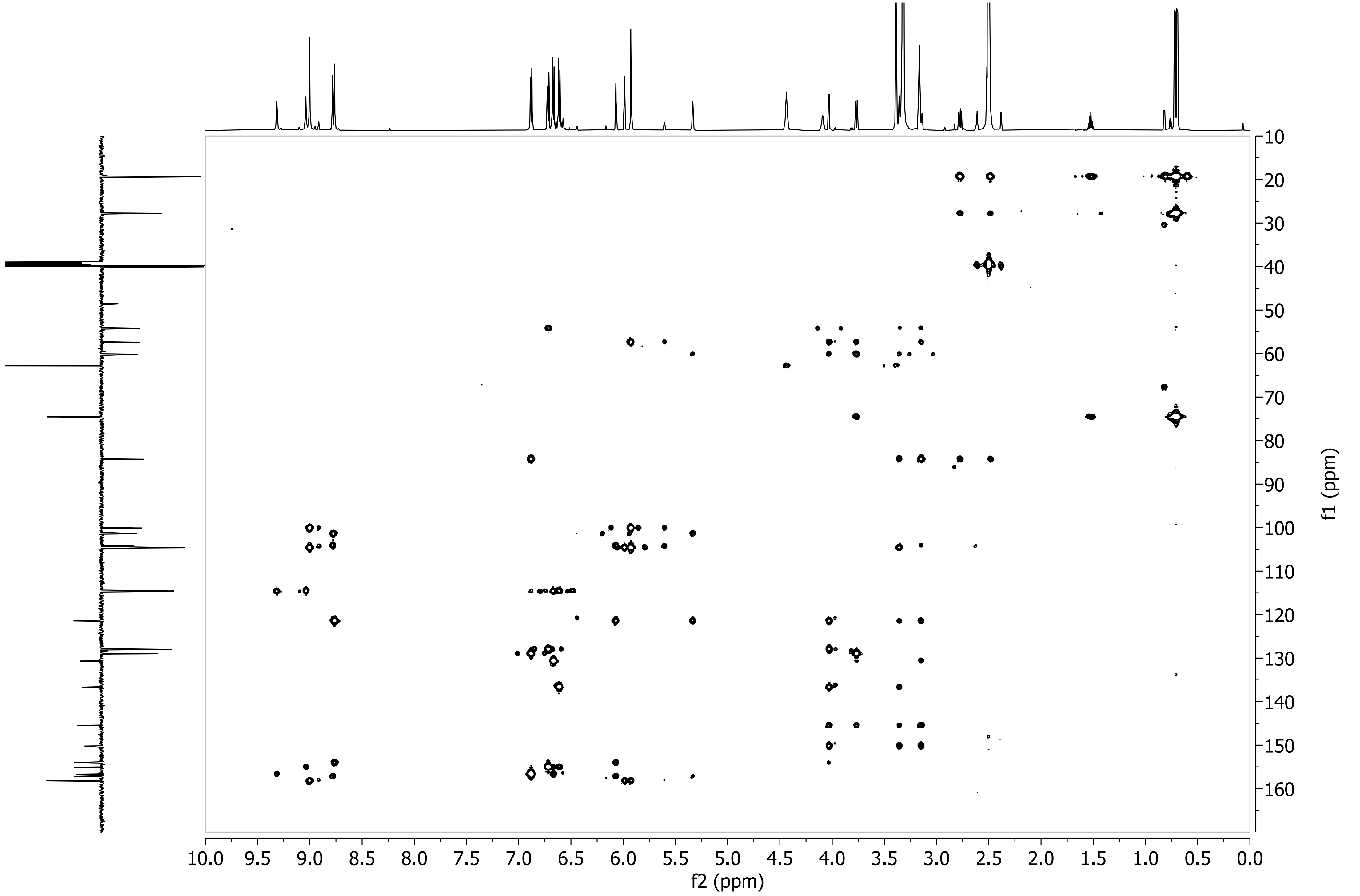

ROESY NMR spectrum of compound **41** in DMSO-d_6

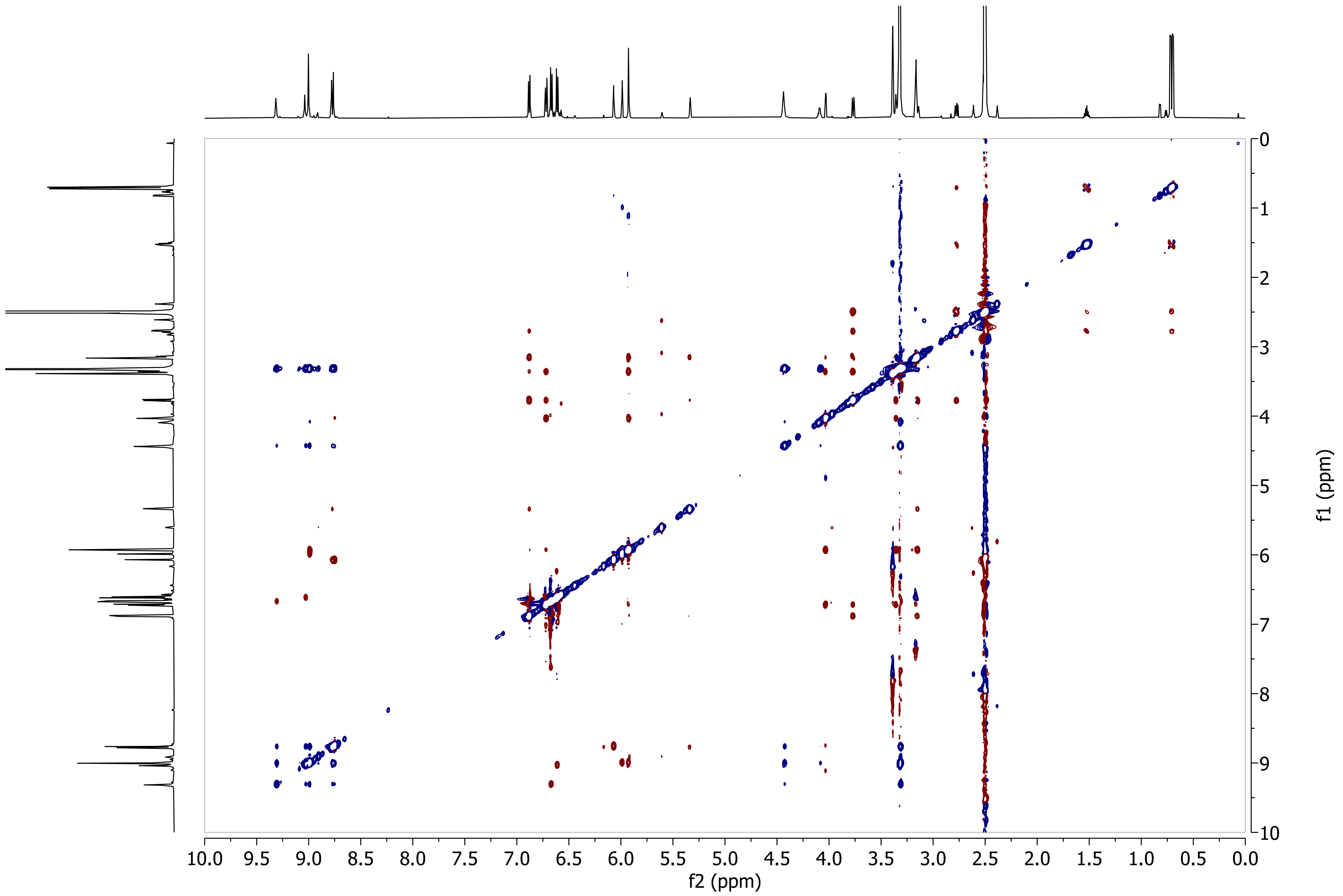

^{1}H NMR spectrum of compound **42** in DMSO-d_6

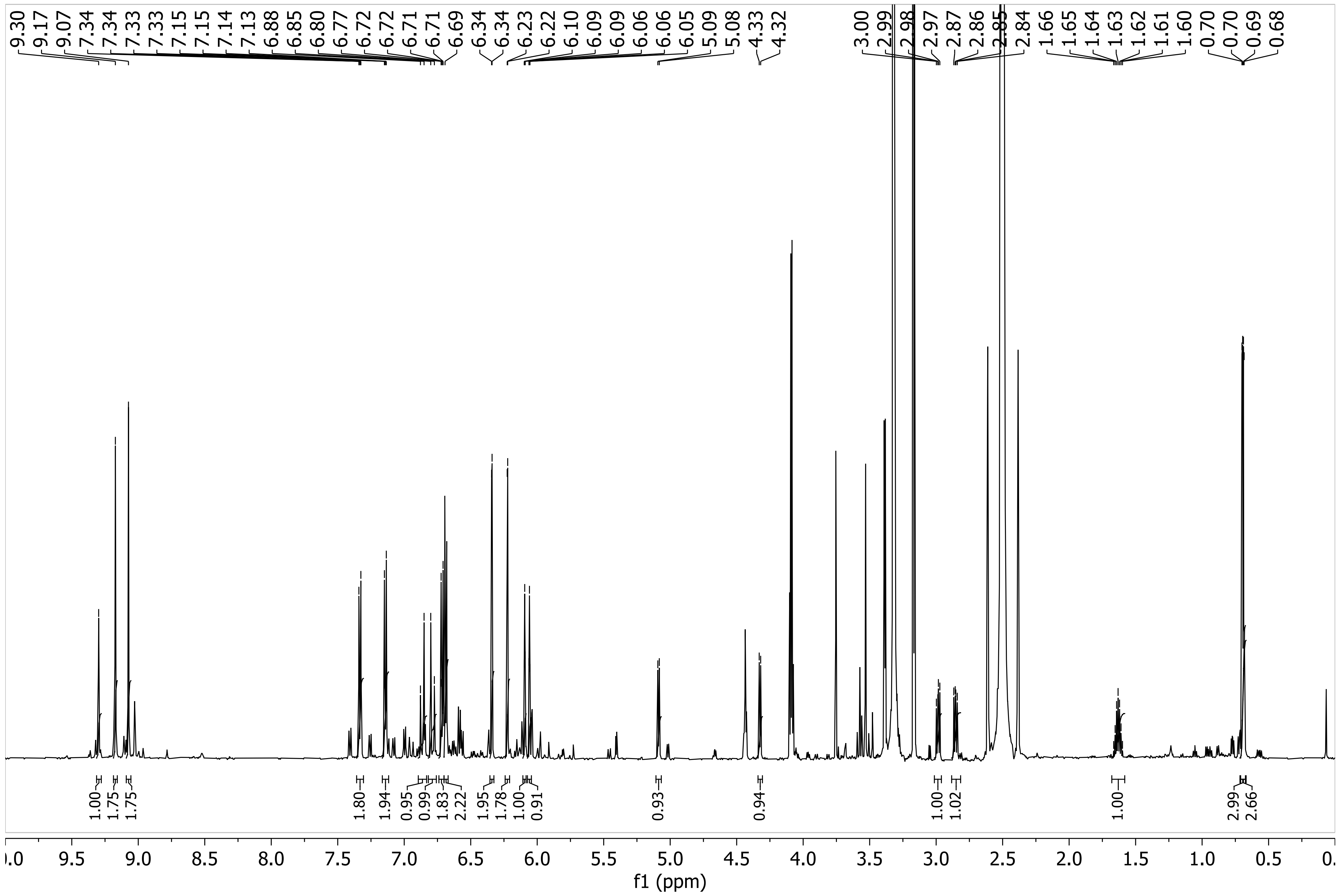

^{1}H NMR spectrum of compound **42** in DMSO-d_6

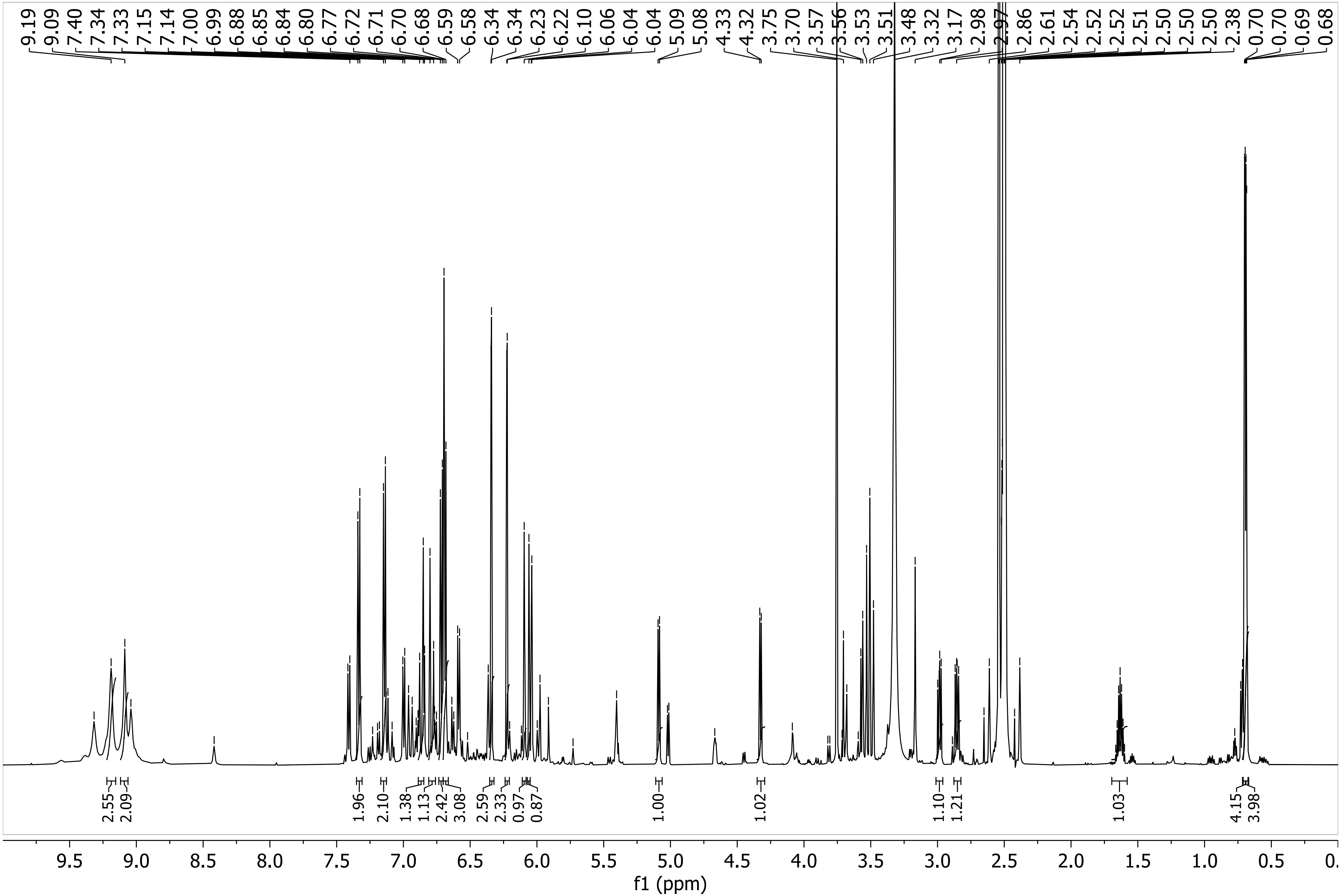

COSY NMR spectrum of compound **42** in DMSO-d_6

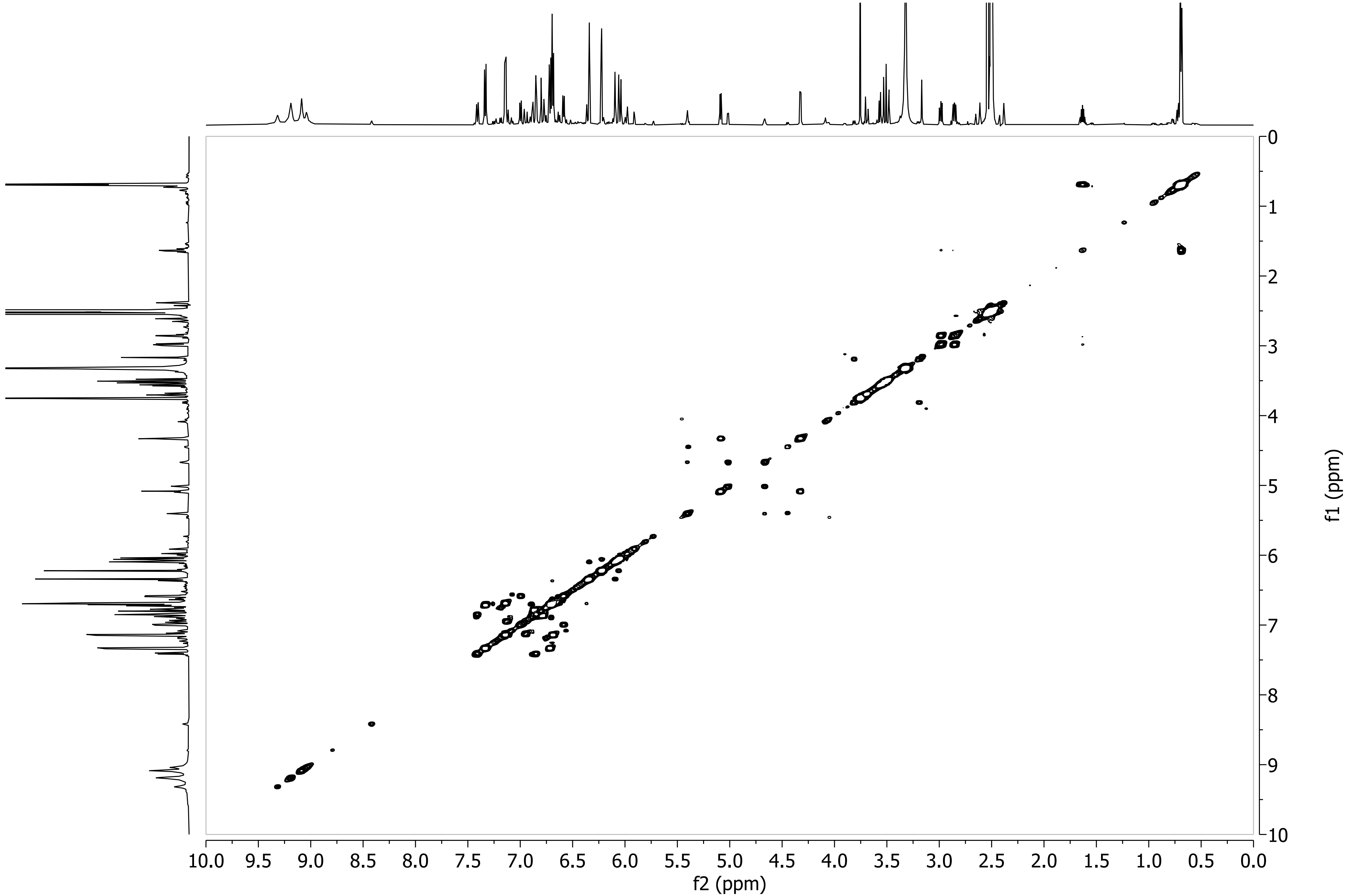

^{13}C-DEPTQ NMR spectrum of compound **42** in DMSO-d_6

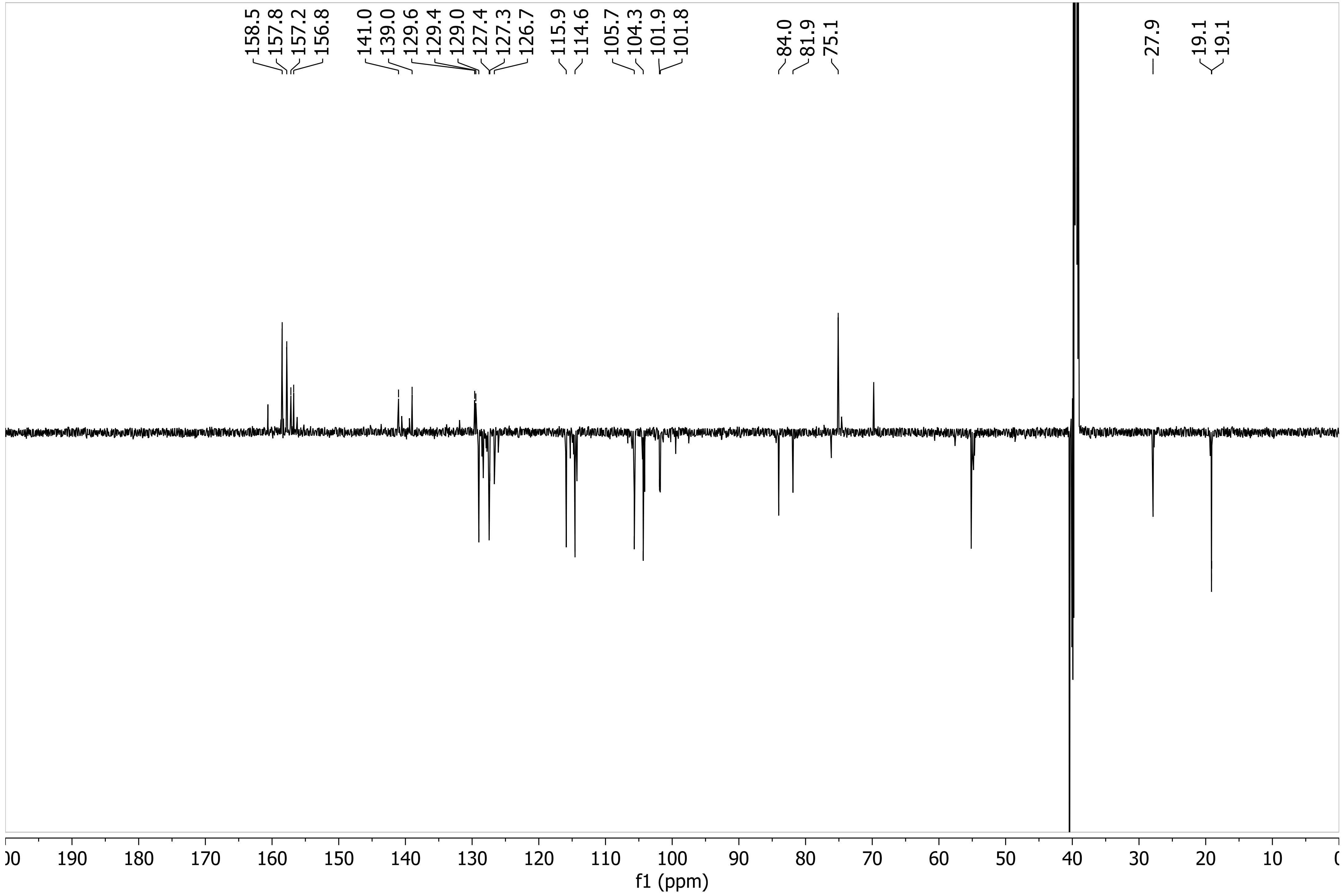

Edited-HSQC NMR spectrum of compound **42** in DMSO-d_6

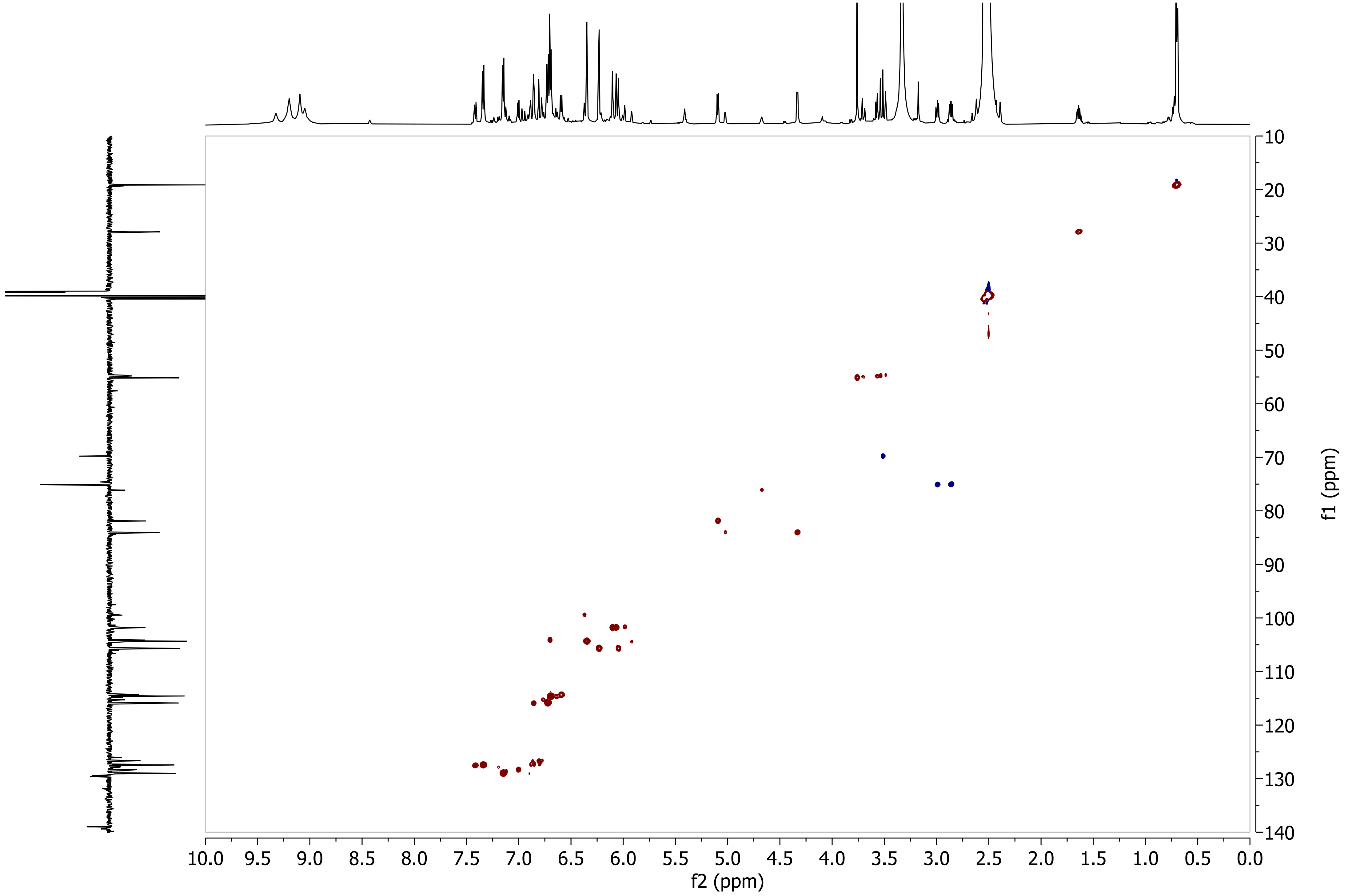

HMBC NMR spectrum of compound **42** in DMSO-d_6

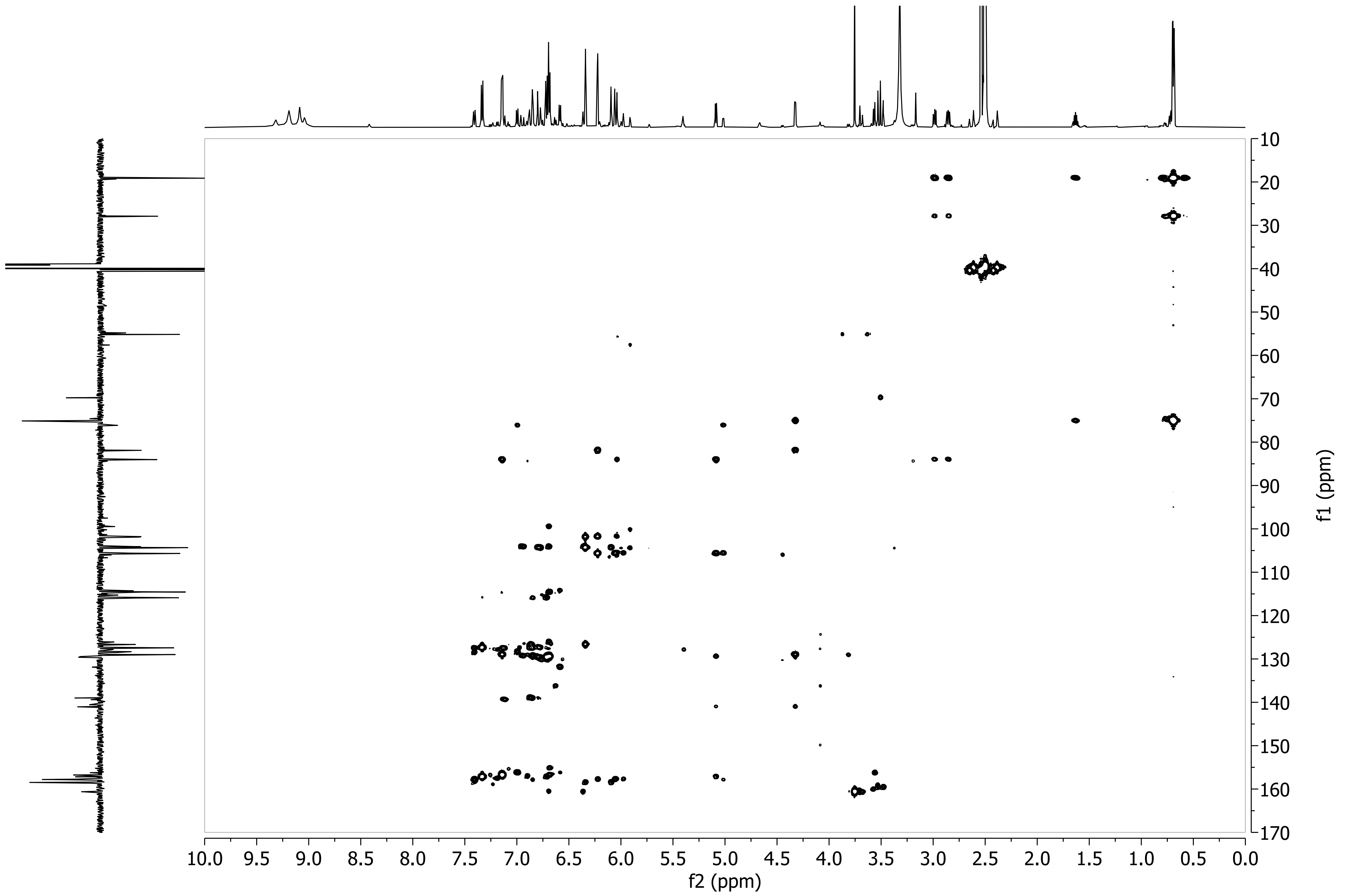

ROESY NMR spectrum of compound **42** in DMSO-d_6

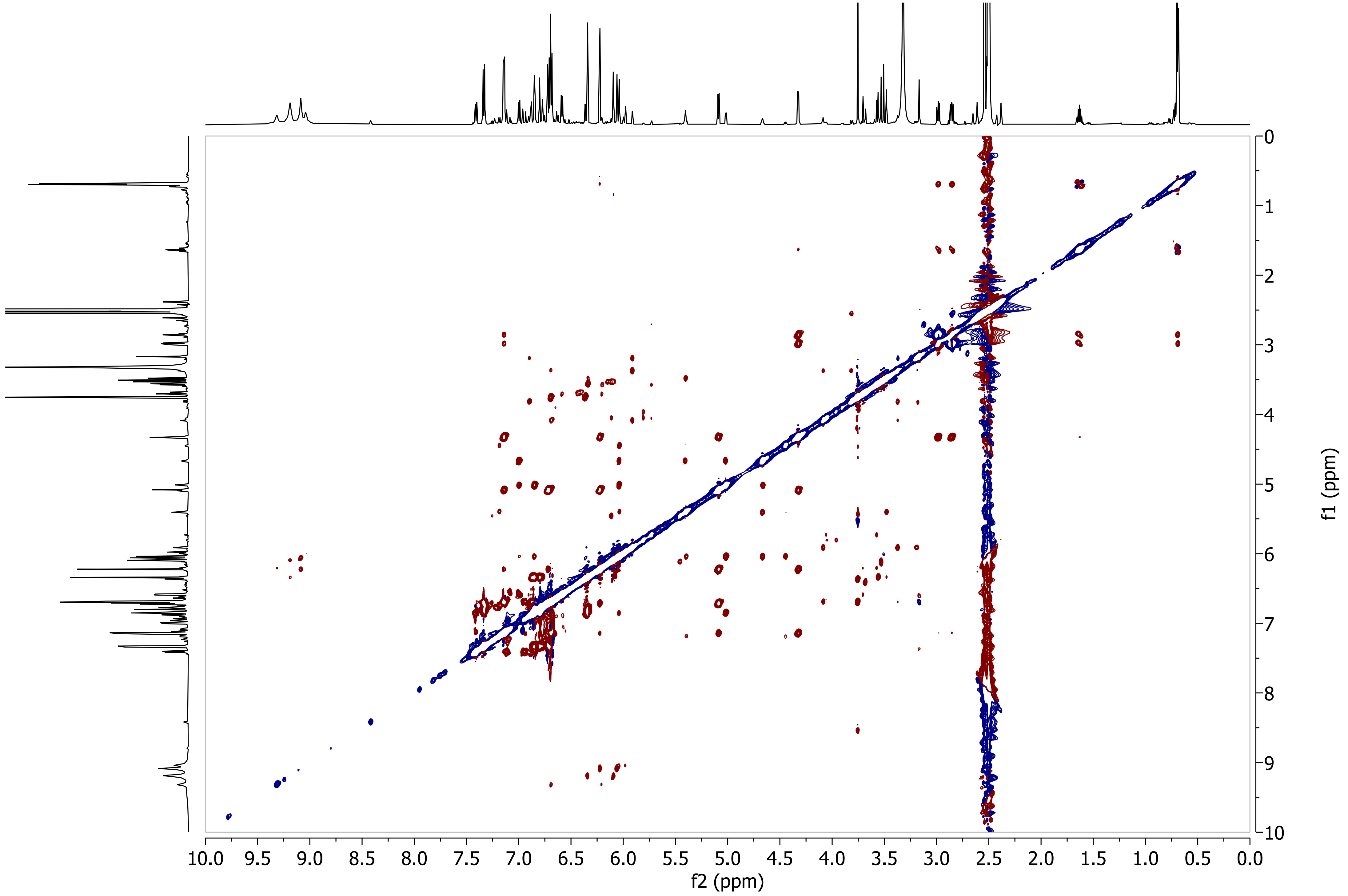

^{1}H NMR spectrum of compound **43** in DMSO-d_6

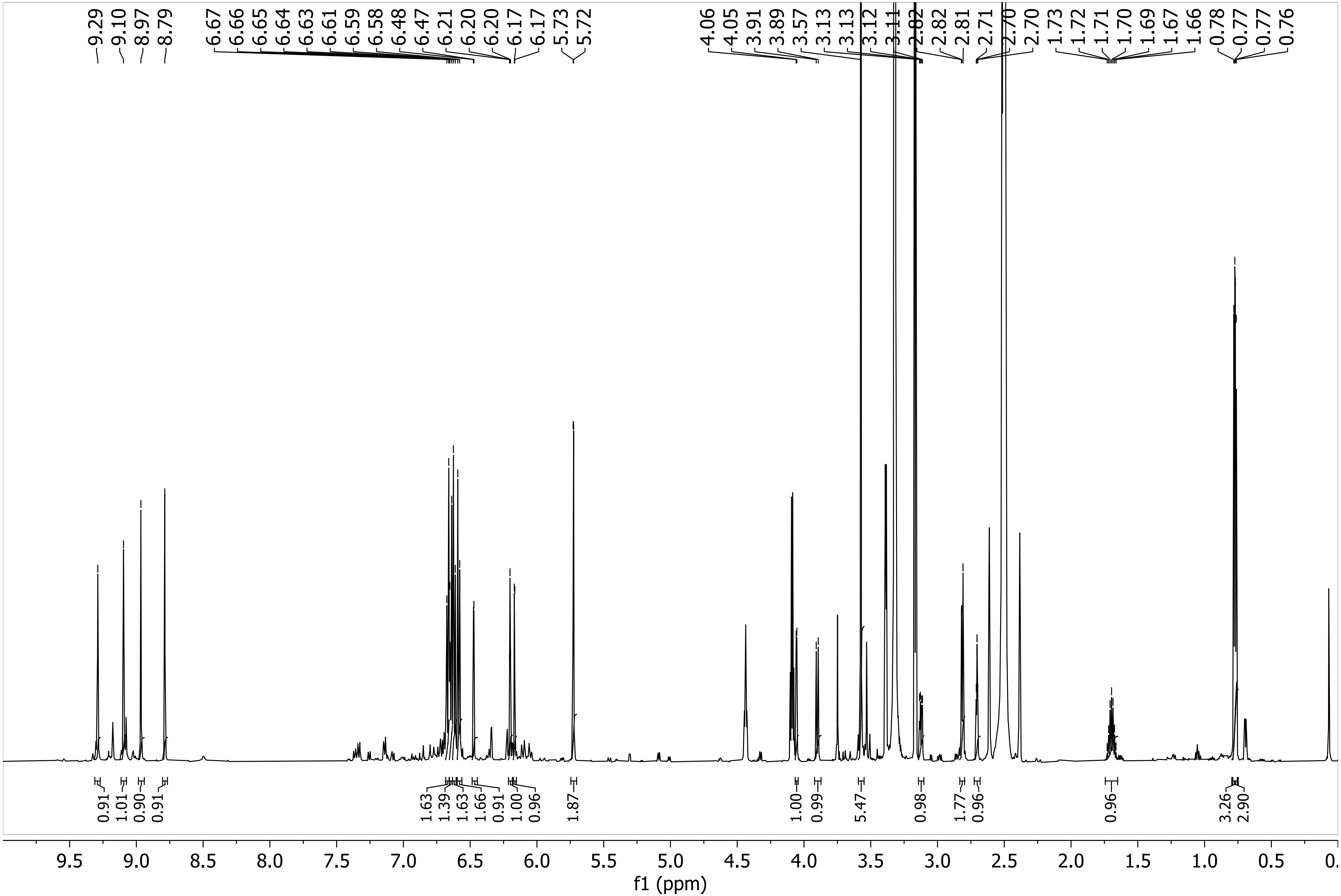

COSY NMR spectrum of compound **43** in DMSO-d_6

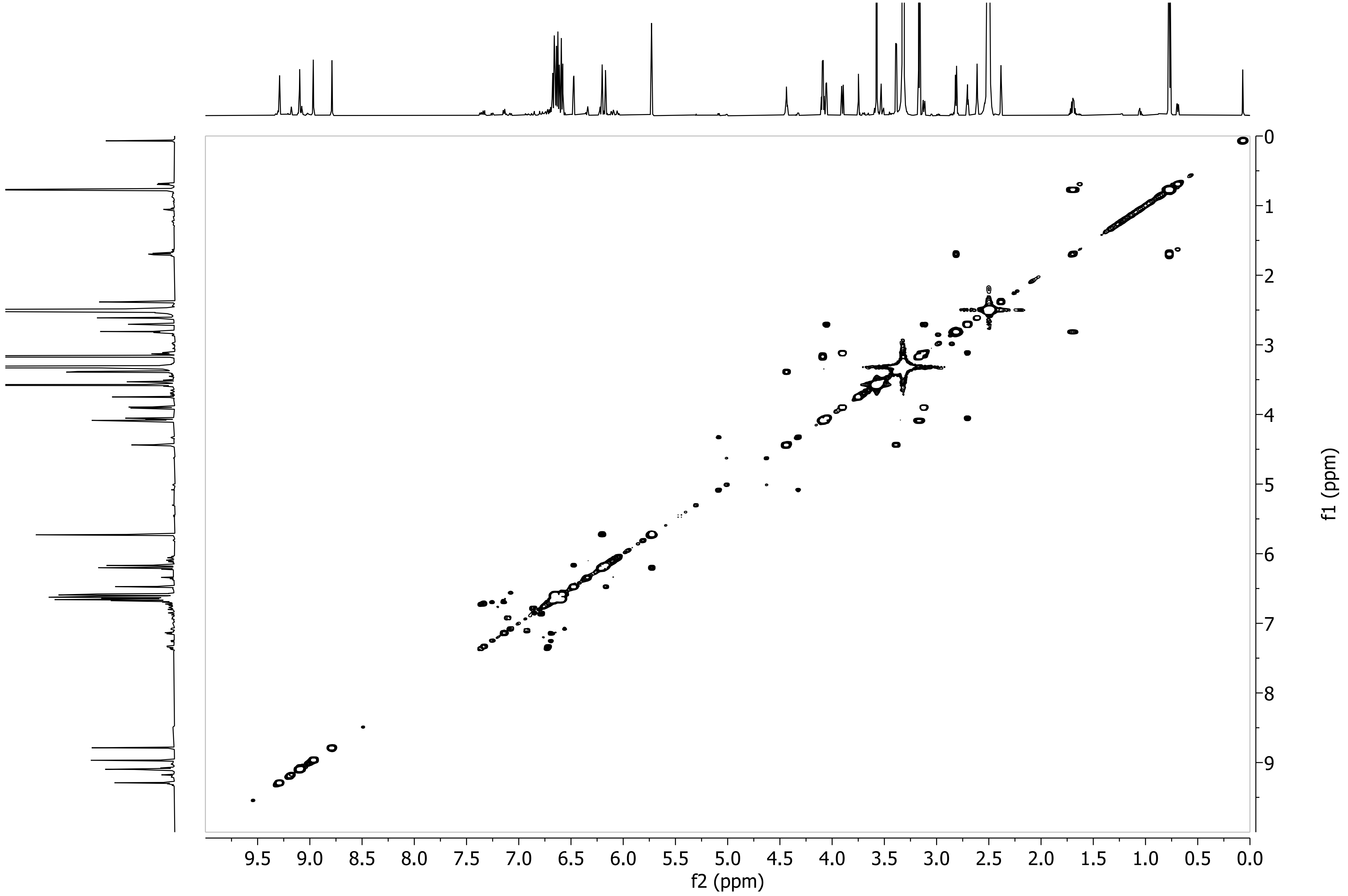

Edited-HSQC NMR spectrum of compound **43** in DMSO-d_6

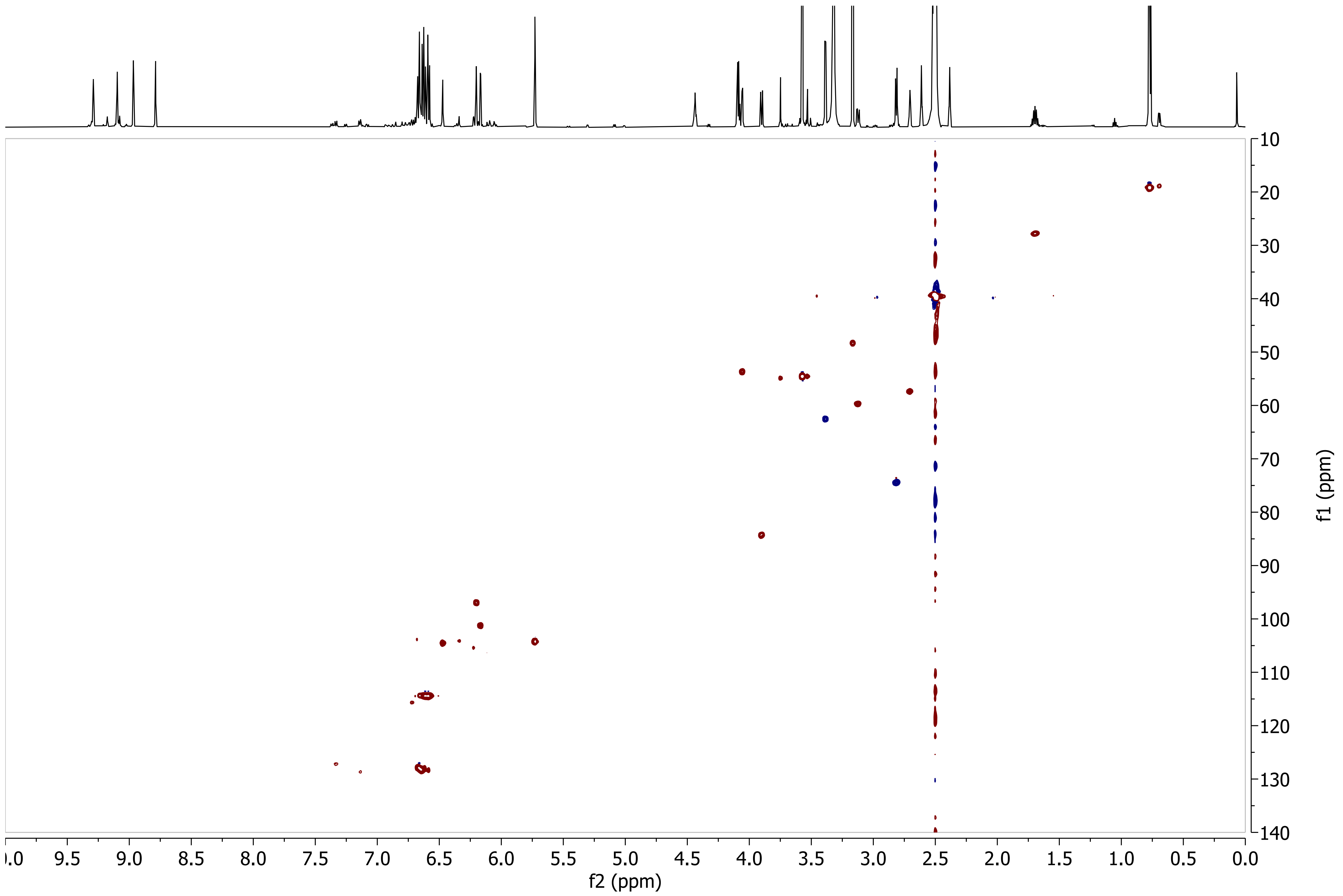

HMBC NMR spectrum of compound **43** in DMSO-d_6

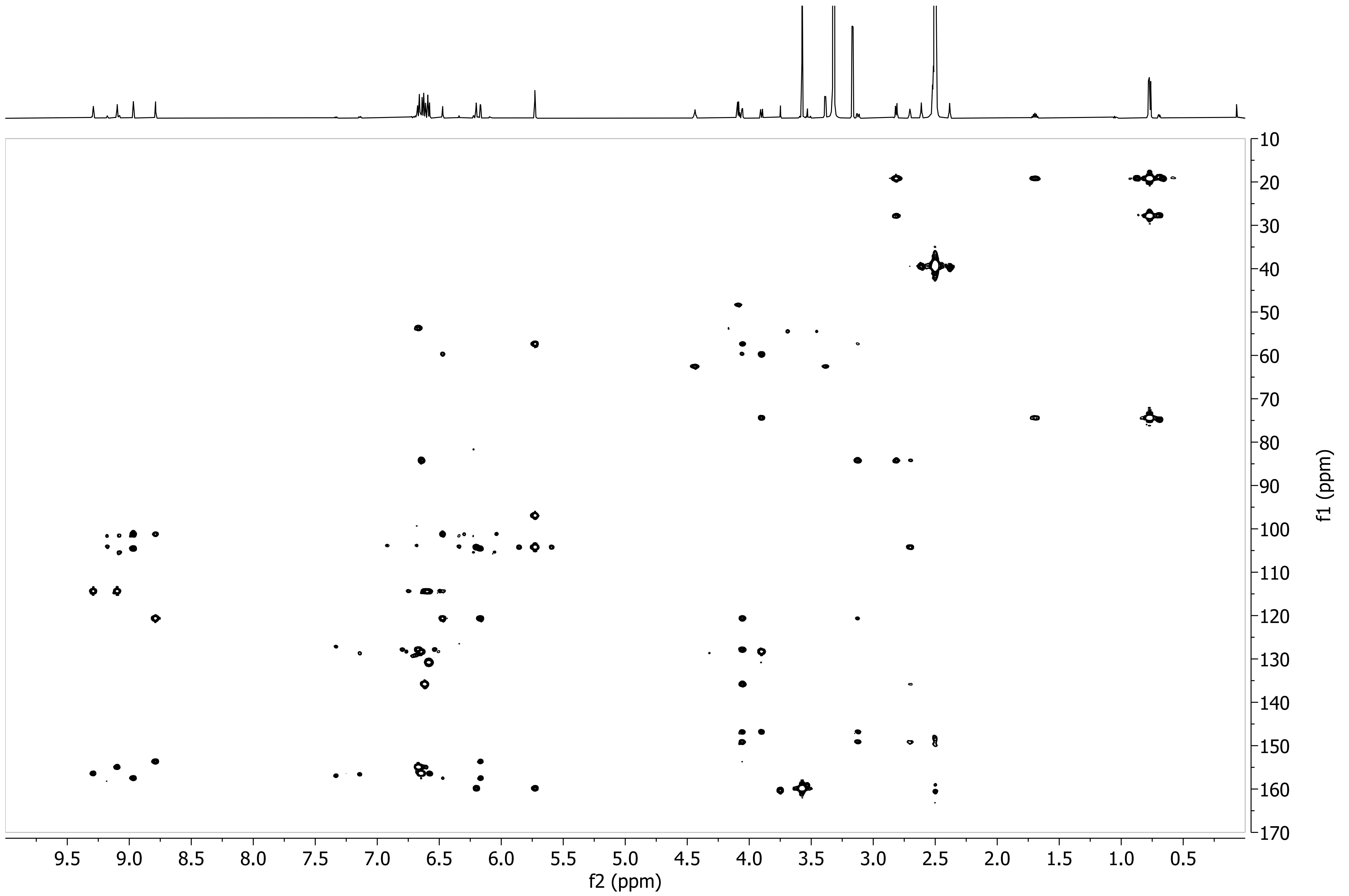

ROESY NMR spectrum of compound **43** in DMSO-d_6

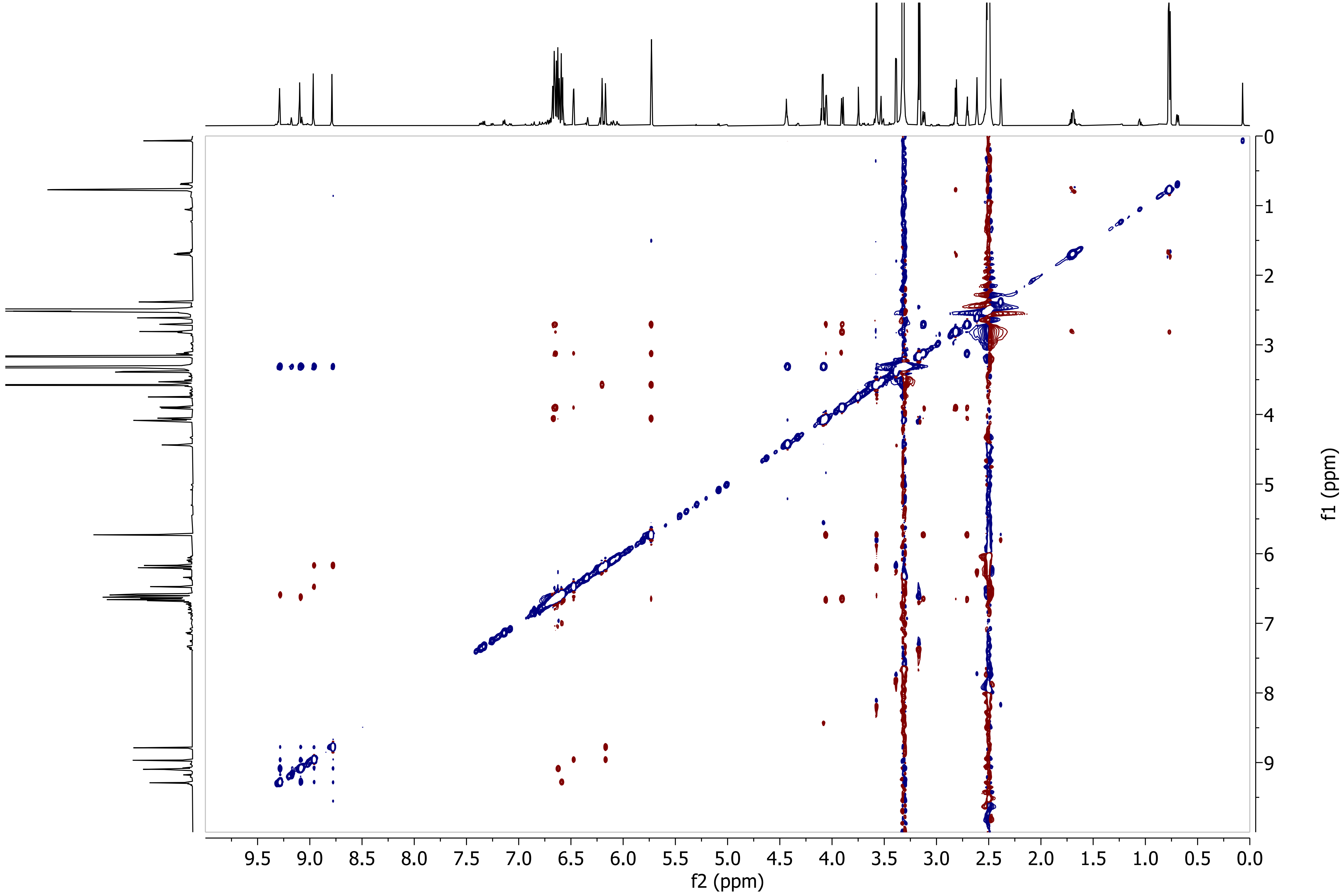

^{1}H NMR spectrum of compound **44** in DMSO-d_6

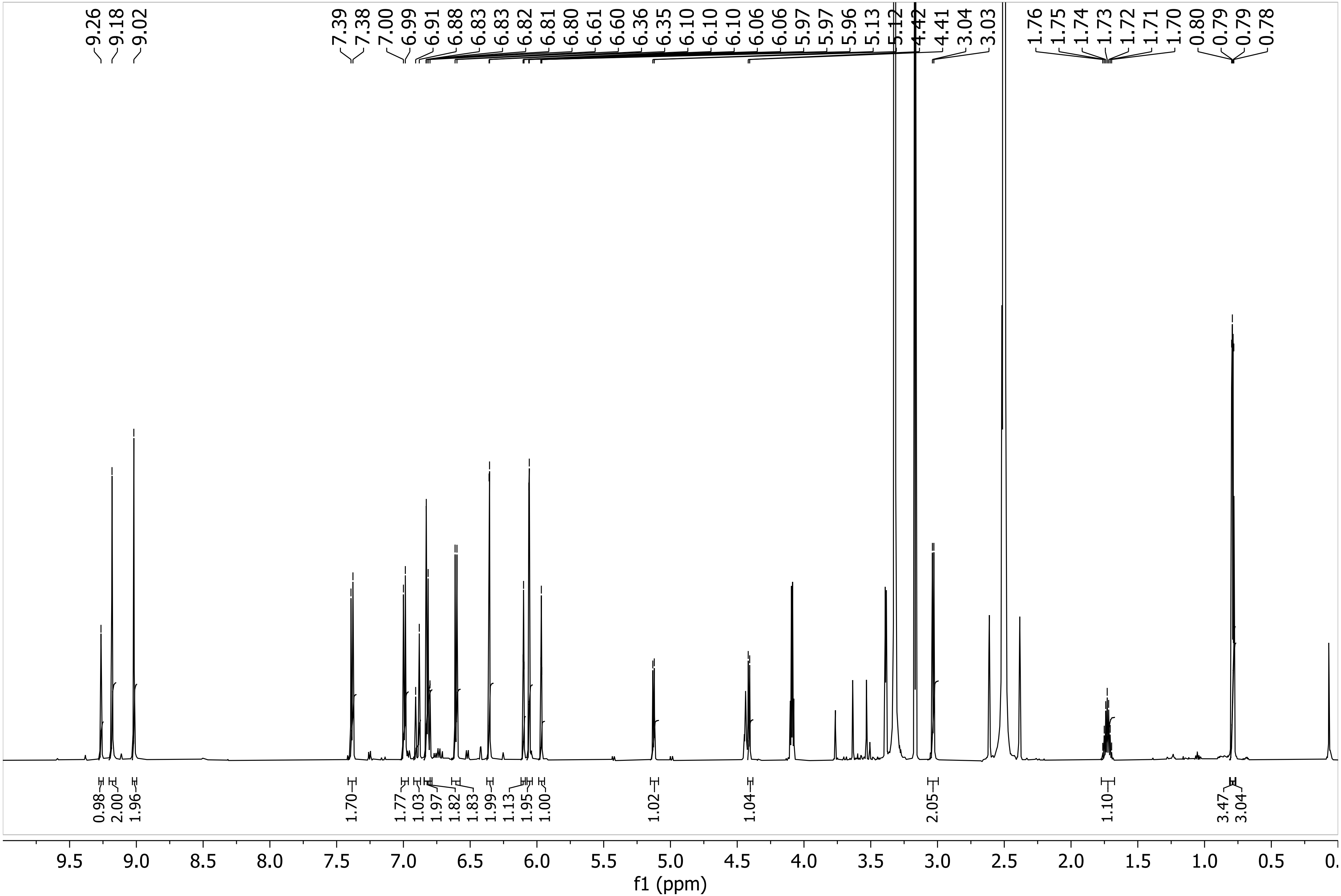

^{1}H NMR spectrum of compound **44** in DMSO-d_6

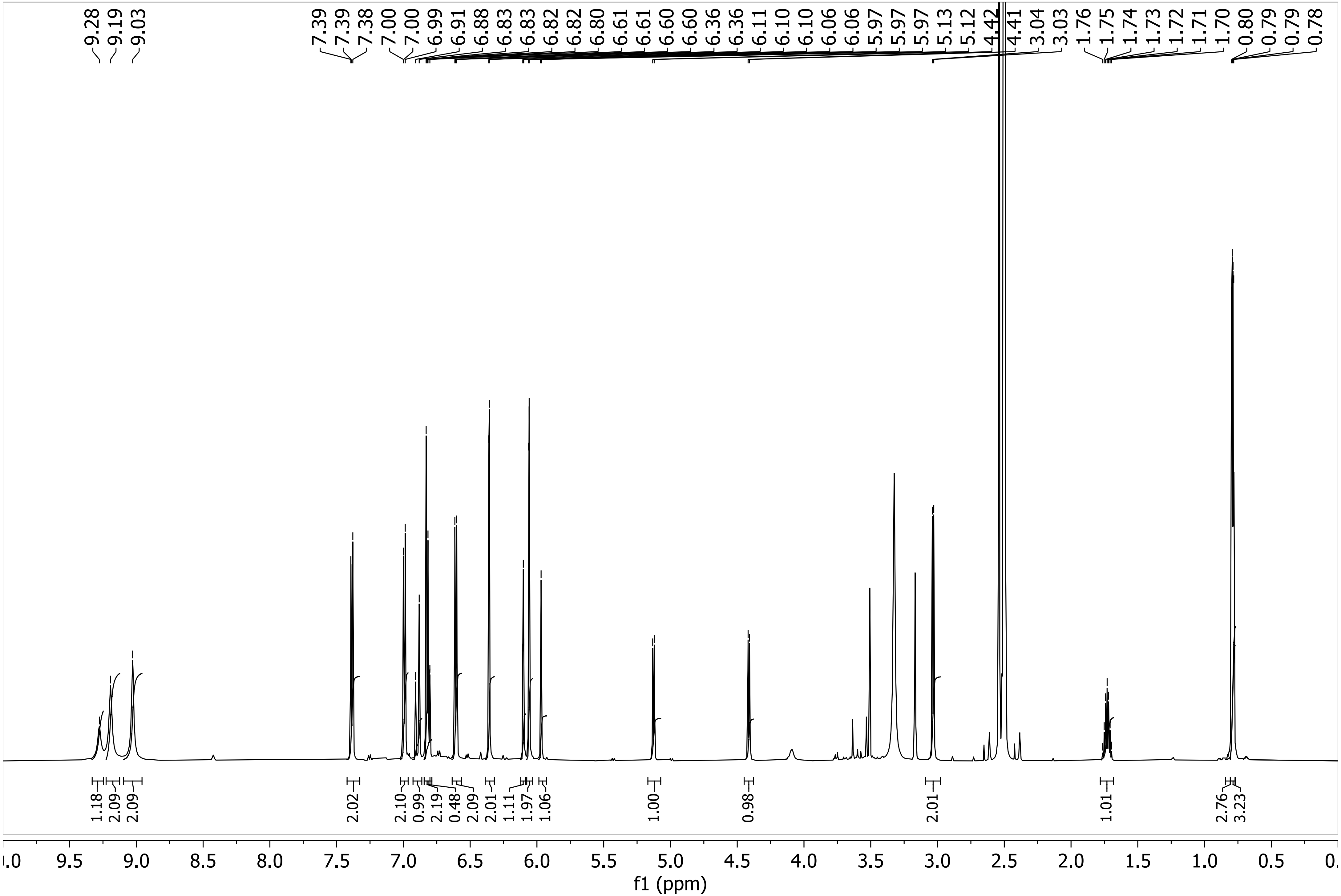

COSY NMR spectrum of compound **44** in DMSO-d_6

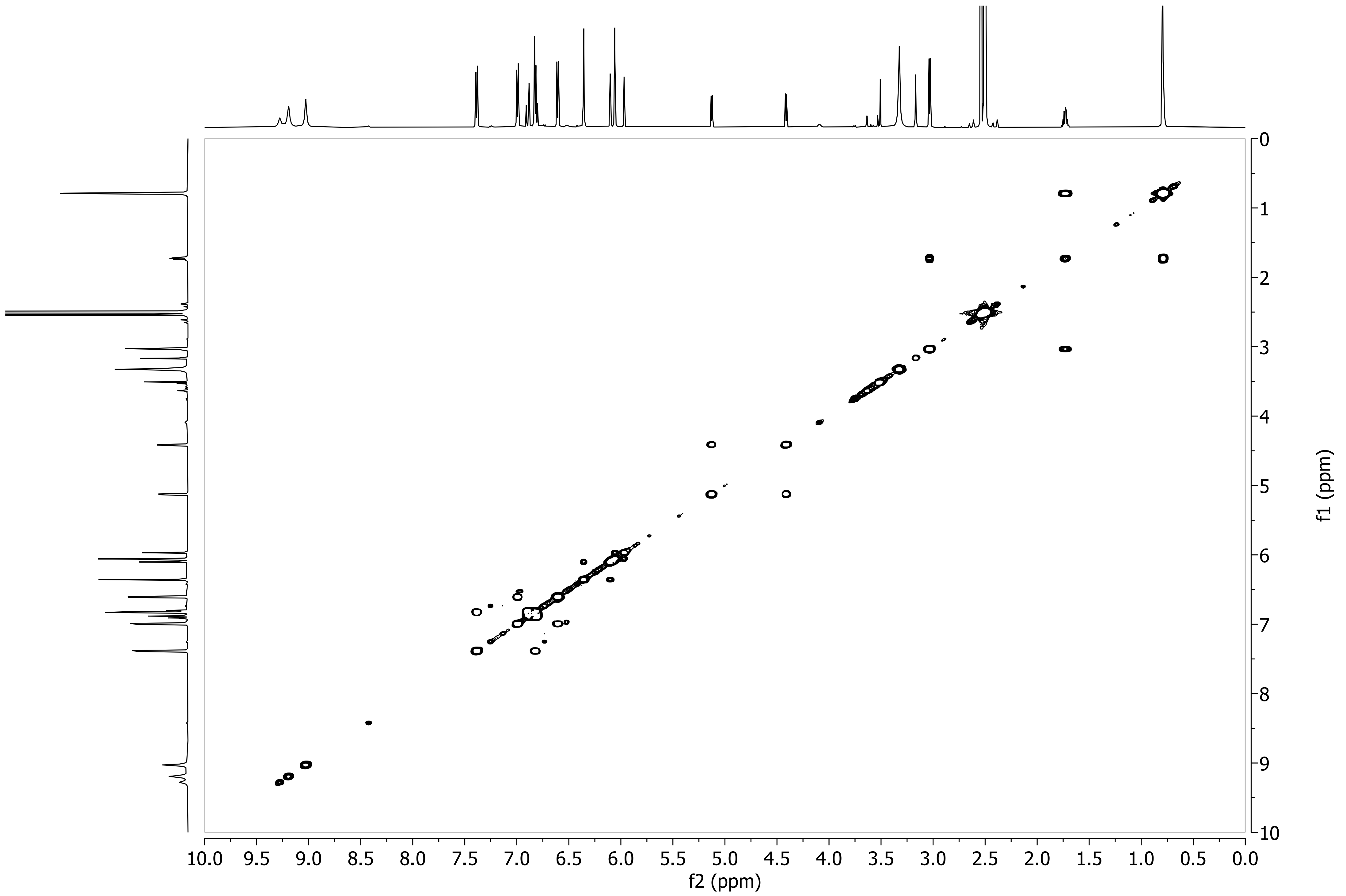

^{13}C-DEPTQ NMR spectrum of compound **44** in DMSO-d_6

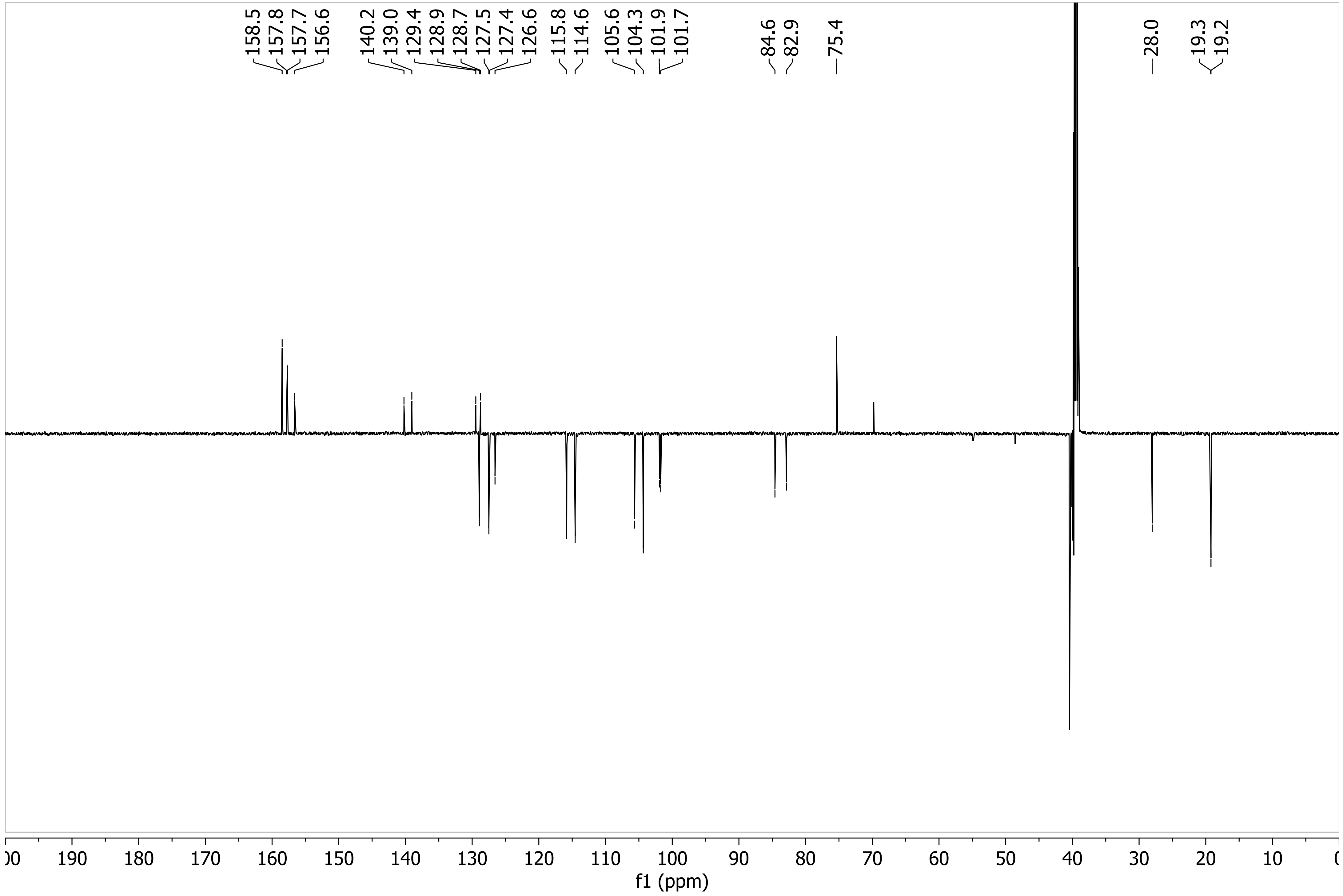

Edited-HSQC NMR spectrum of compound **44** in DMSO-d_6

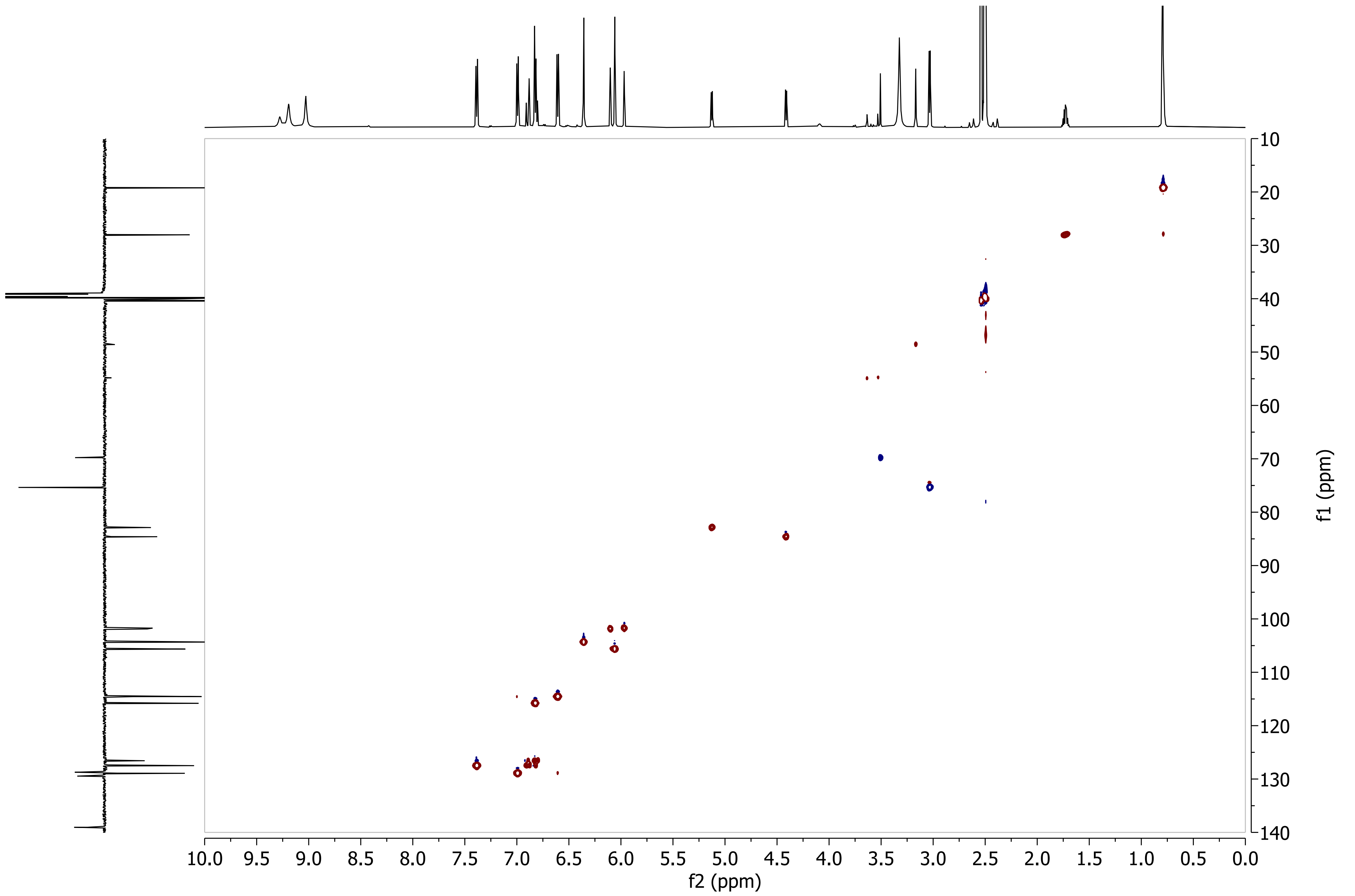

HMBC NMR spectrum of compound **44** in DMSO-d_6

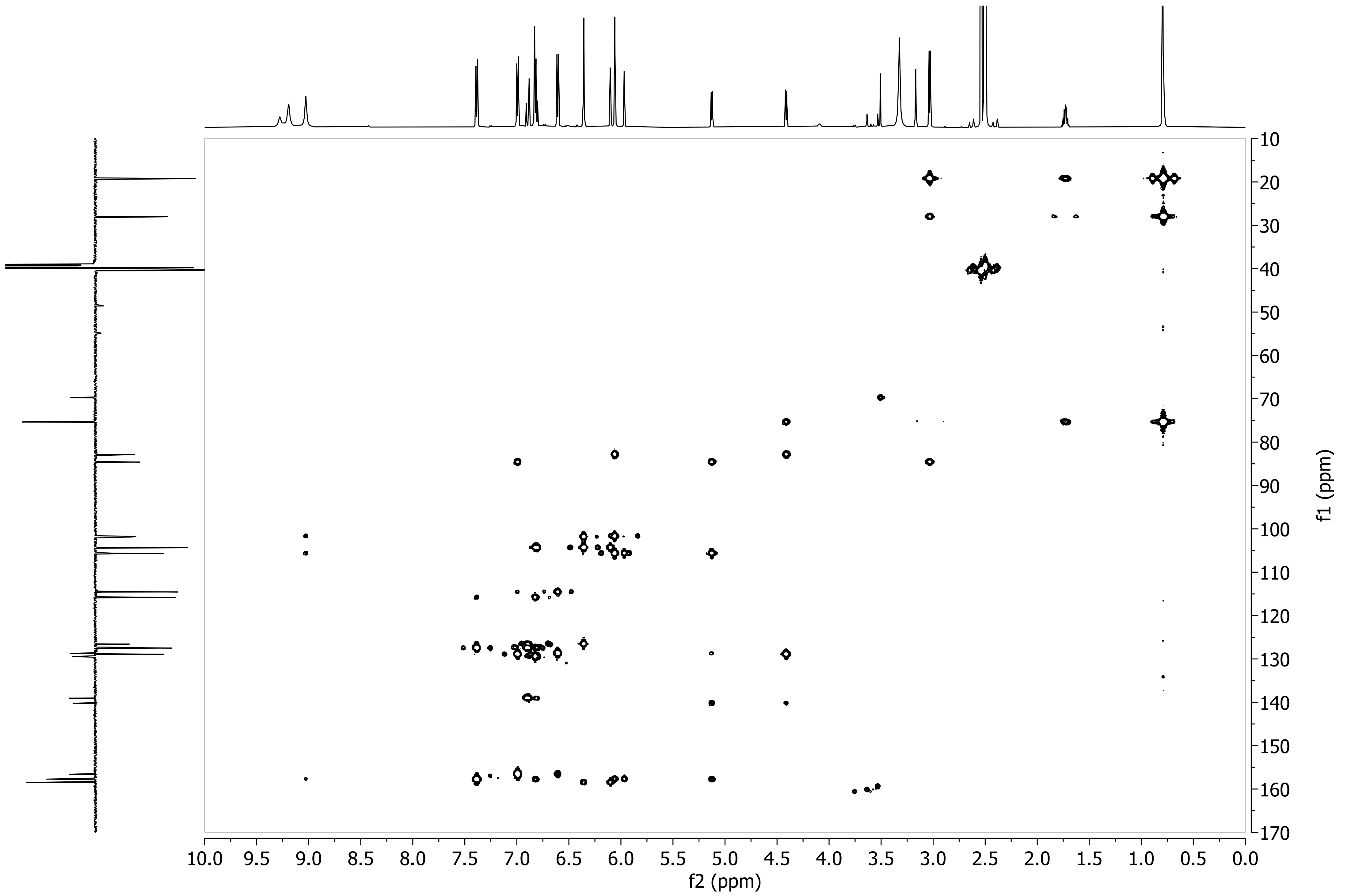

ROESY NMR spectrum of compound **44** in DMSO-d_6

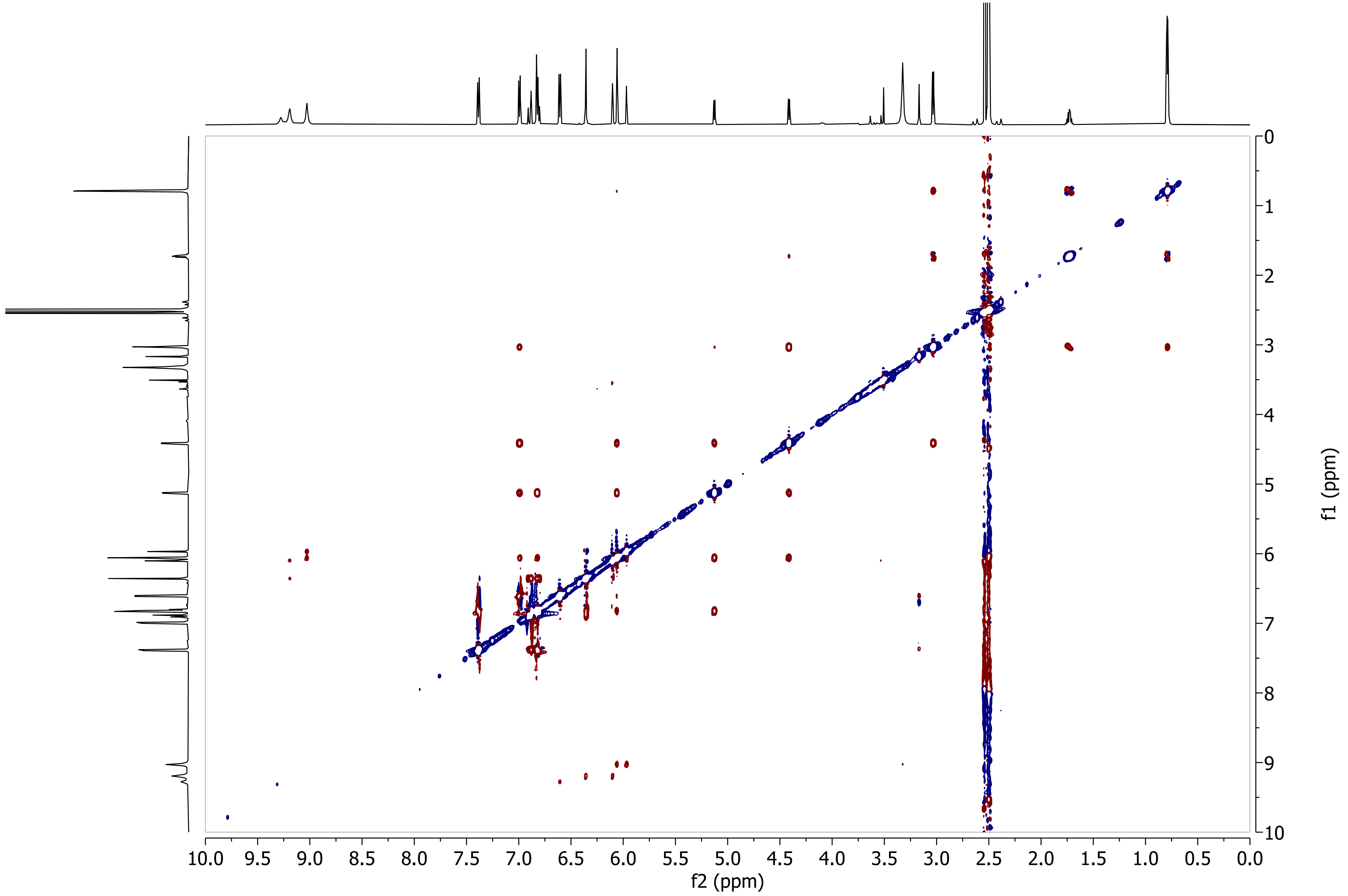

^{1}H NMR spectrum of compound **45** in DMSO-d_6

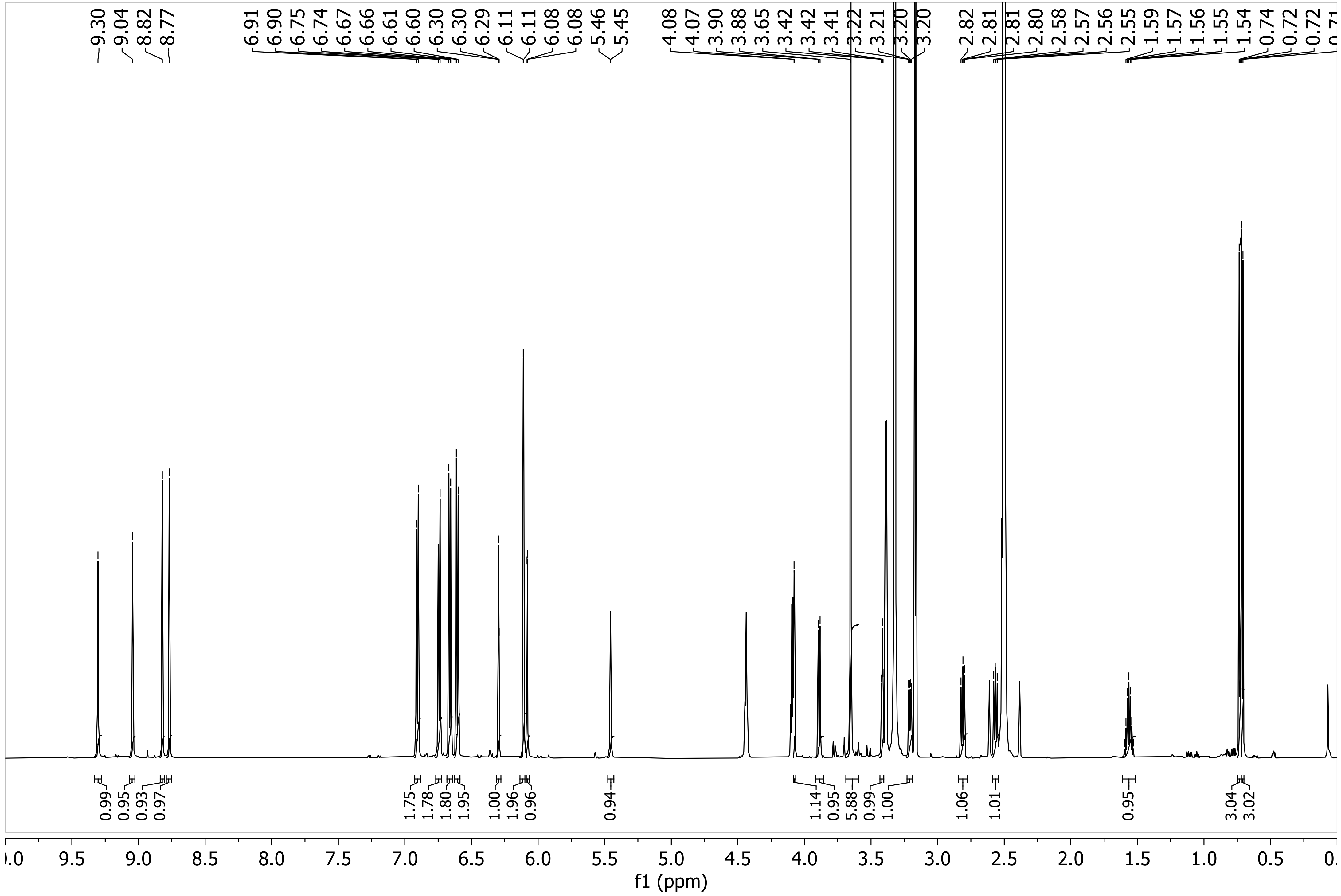

^{1}H NMR spectrum of compound **45** in DMSO-d_6

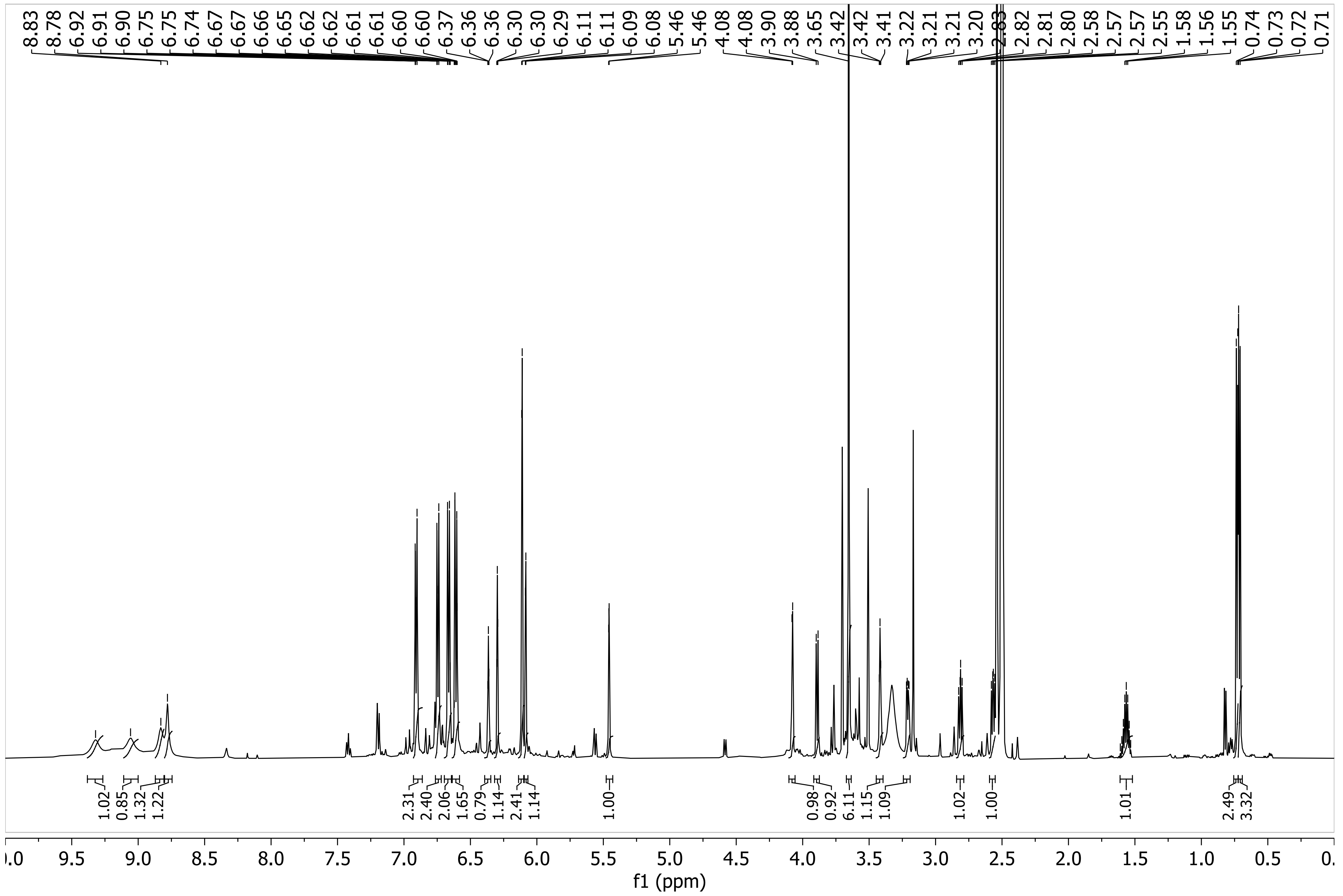

COSY NMR spectrum of compound **45** in DMSO-d_6

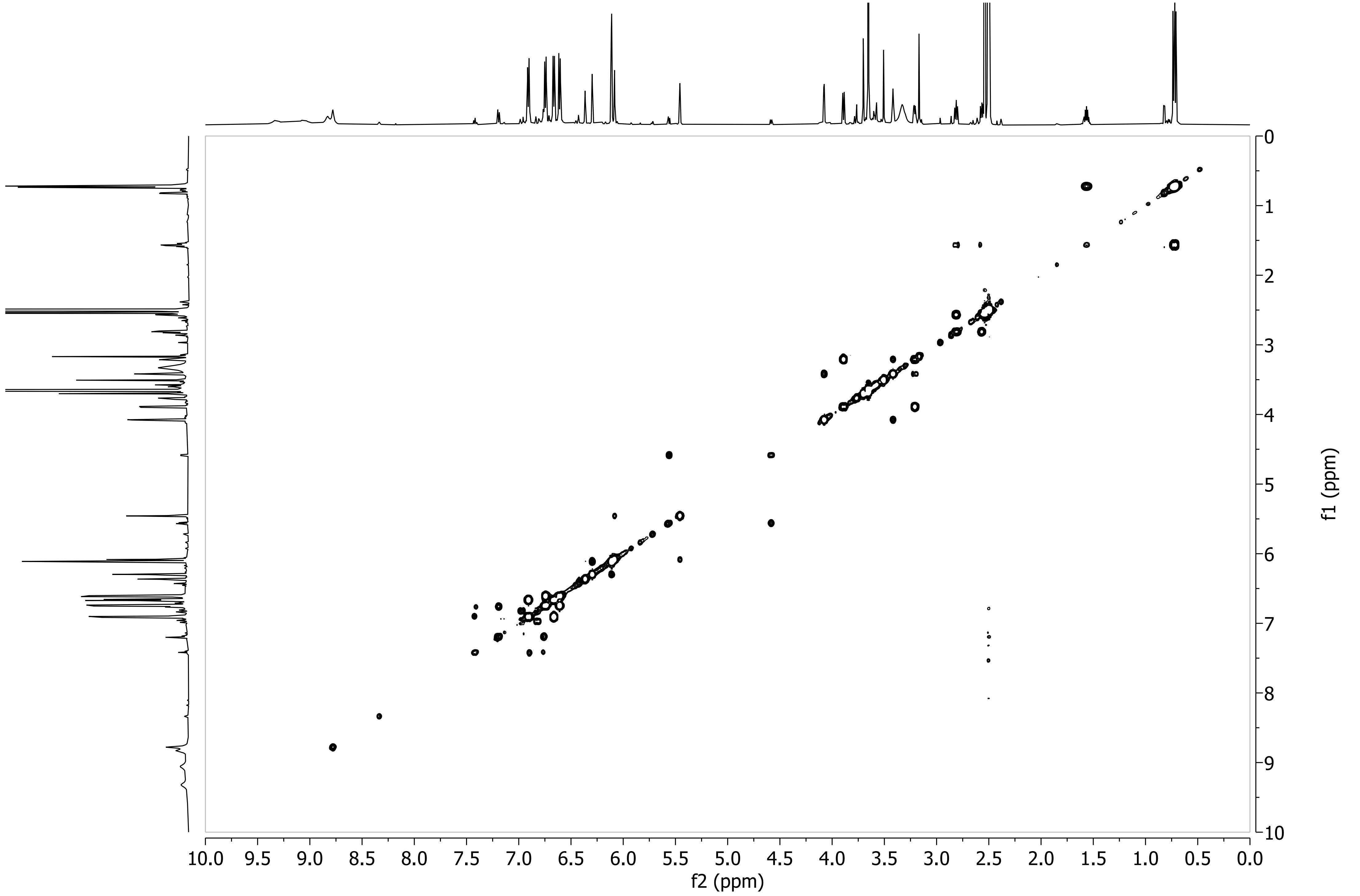

^{13}C-DEPTQ NMR spectrum of compound **45** in DMSO-d_6

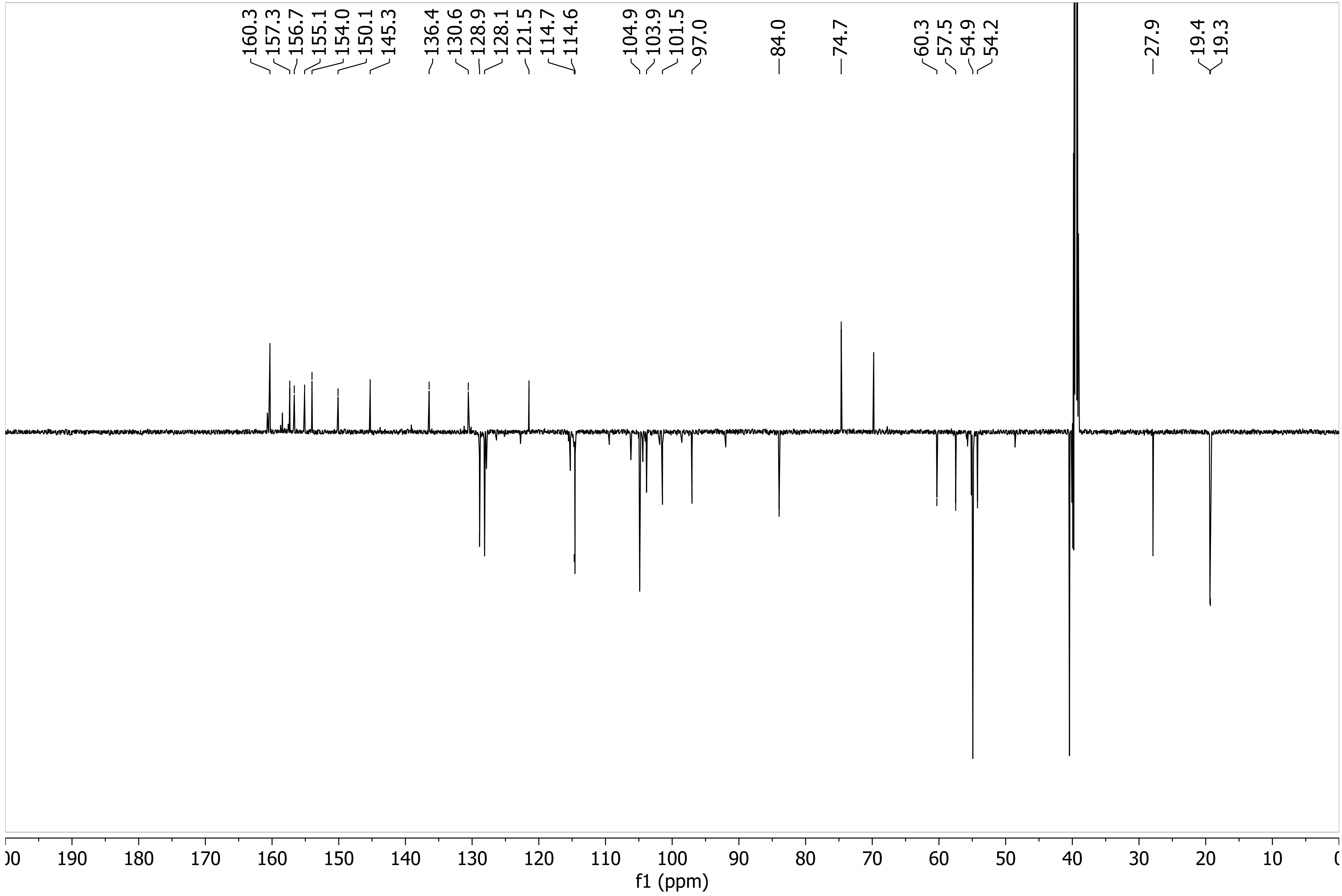

Edited-HSQC NMR spectrum of compound **45** in DMSO-d_6

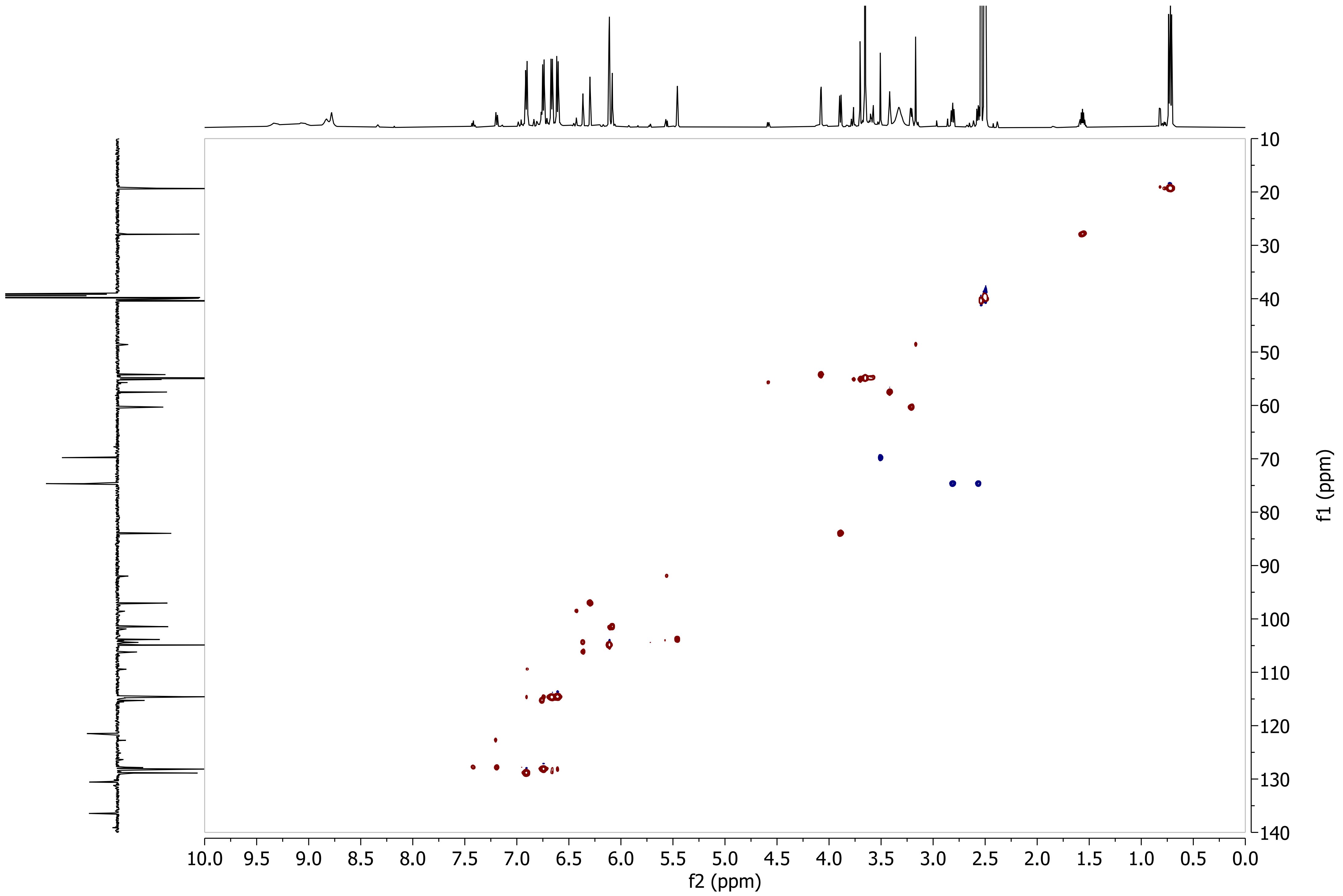

HMBC NMR spectrum of compound **45** in DMSO-d_6

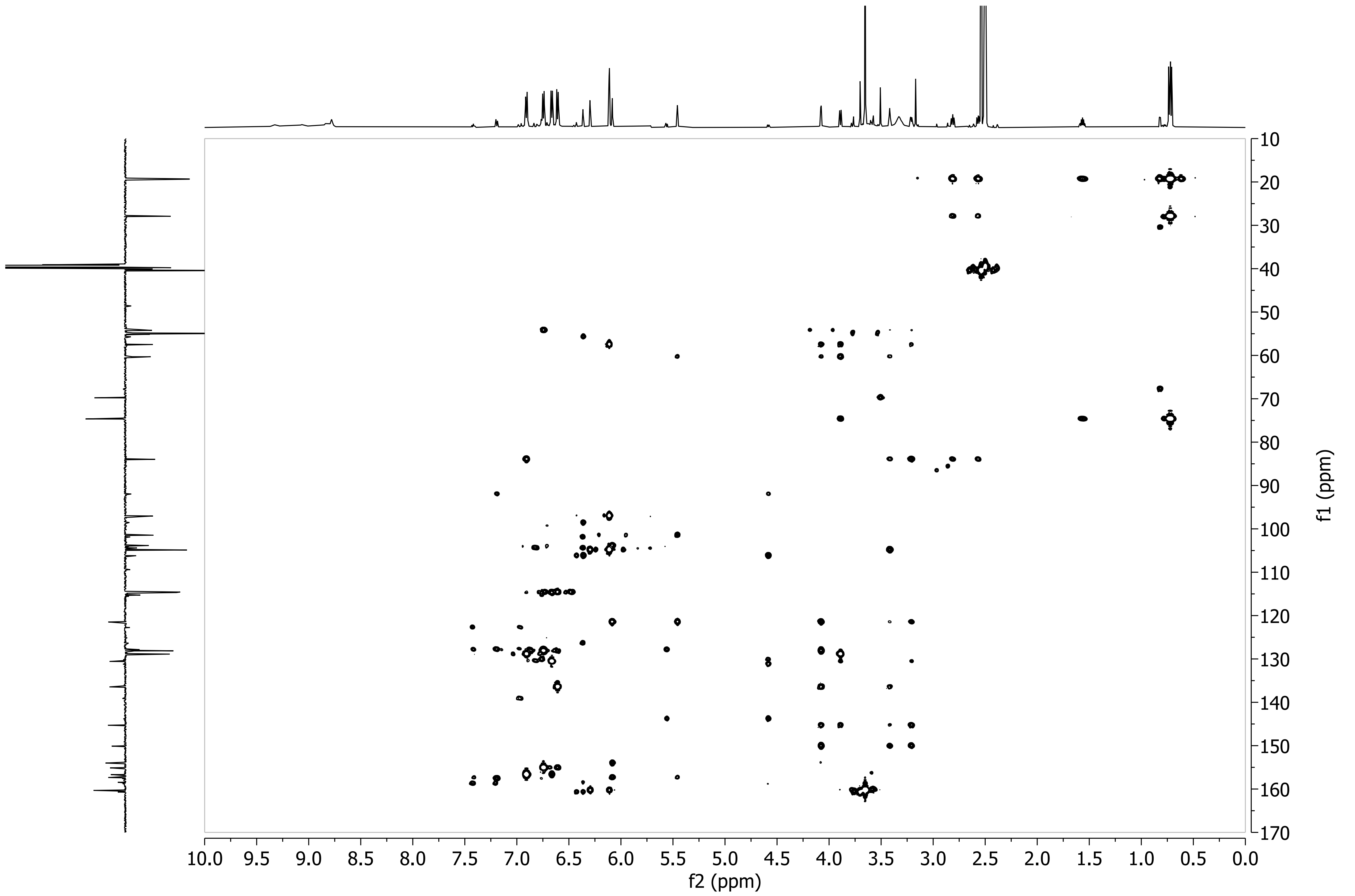

ROESY NMR spectrum of compound **45** in DMSO-d_6

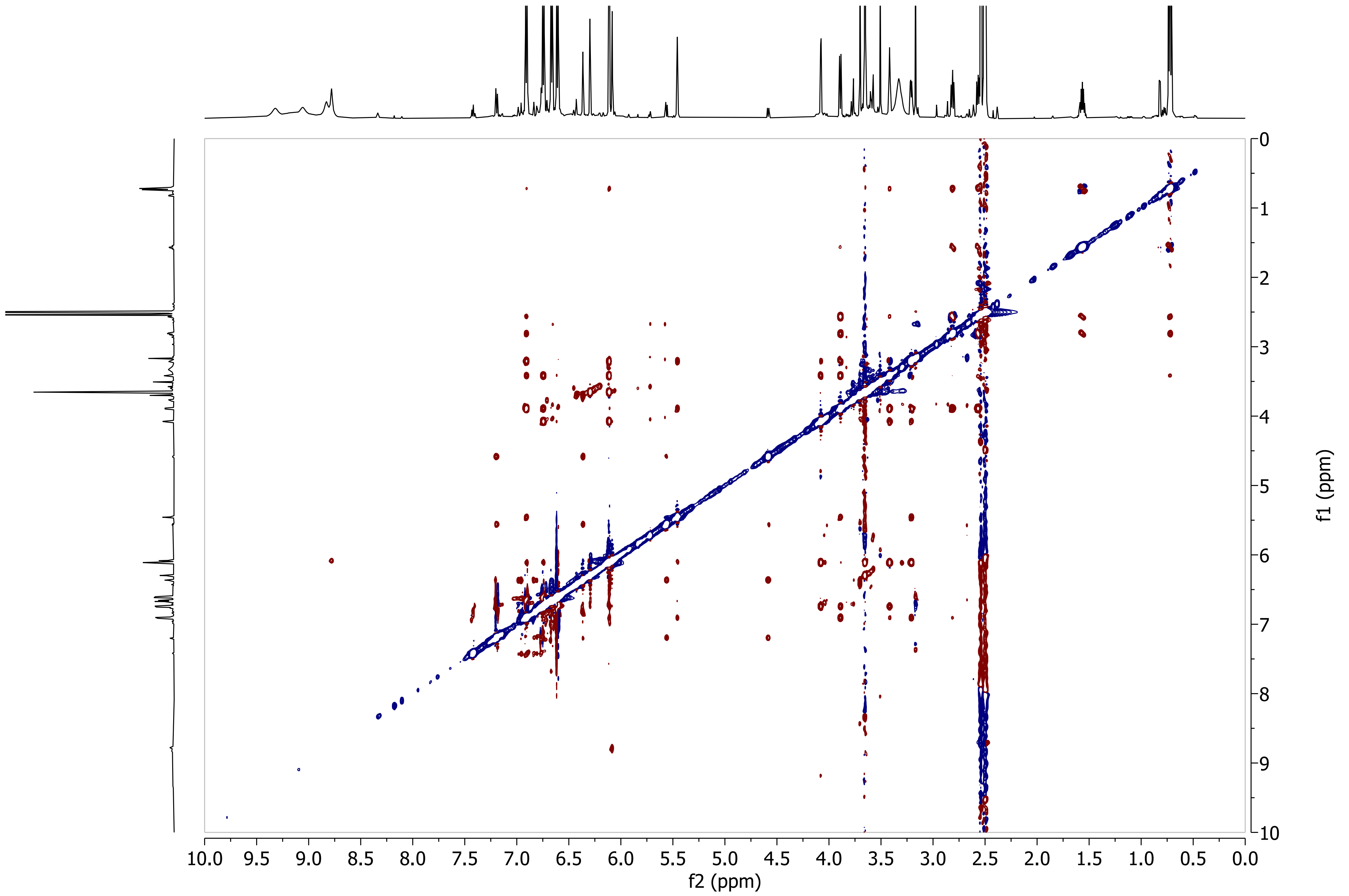

^{1}H NMR spectrum of compound **46** in DMSO-d_6

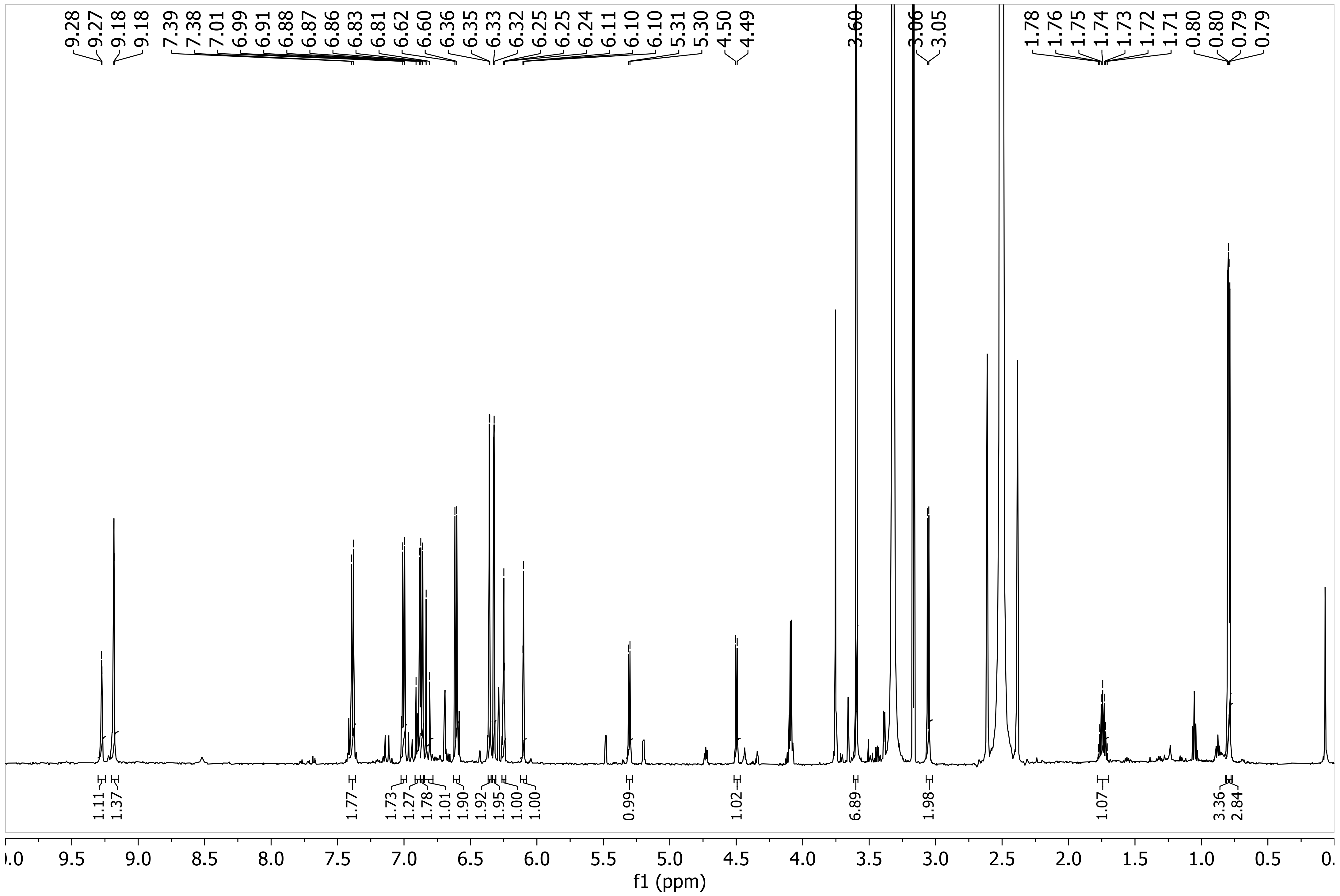

^{1}H NMR spectrum of compound **46** in DMSO-d_6

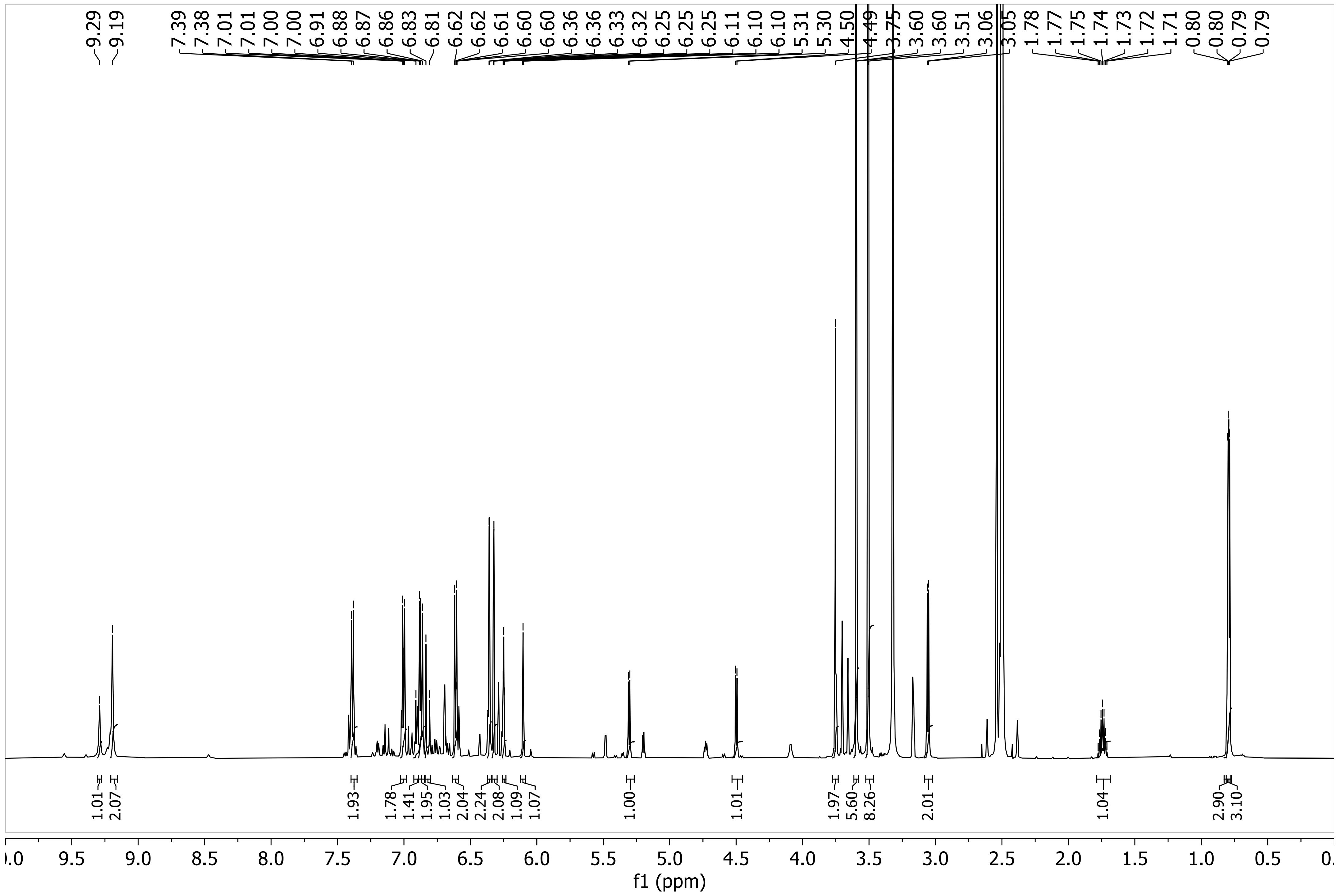

COSY NMR spectrum of compound **46** in DMSO-d_6

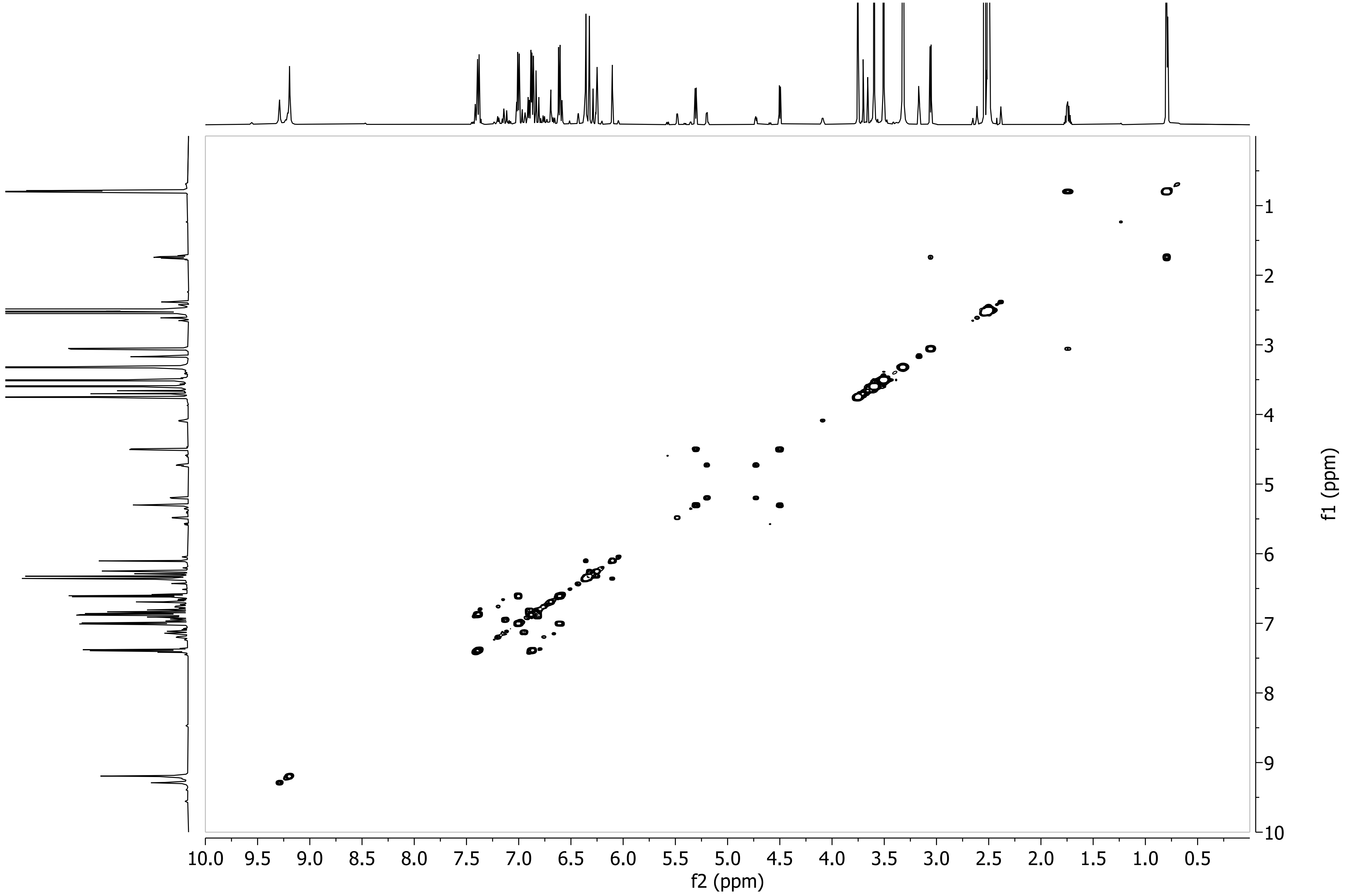

^{13}C-DEPTQ NMR spectrum of compound **46** in DMSO-d_6

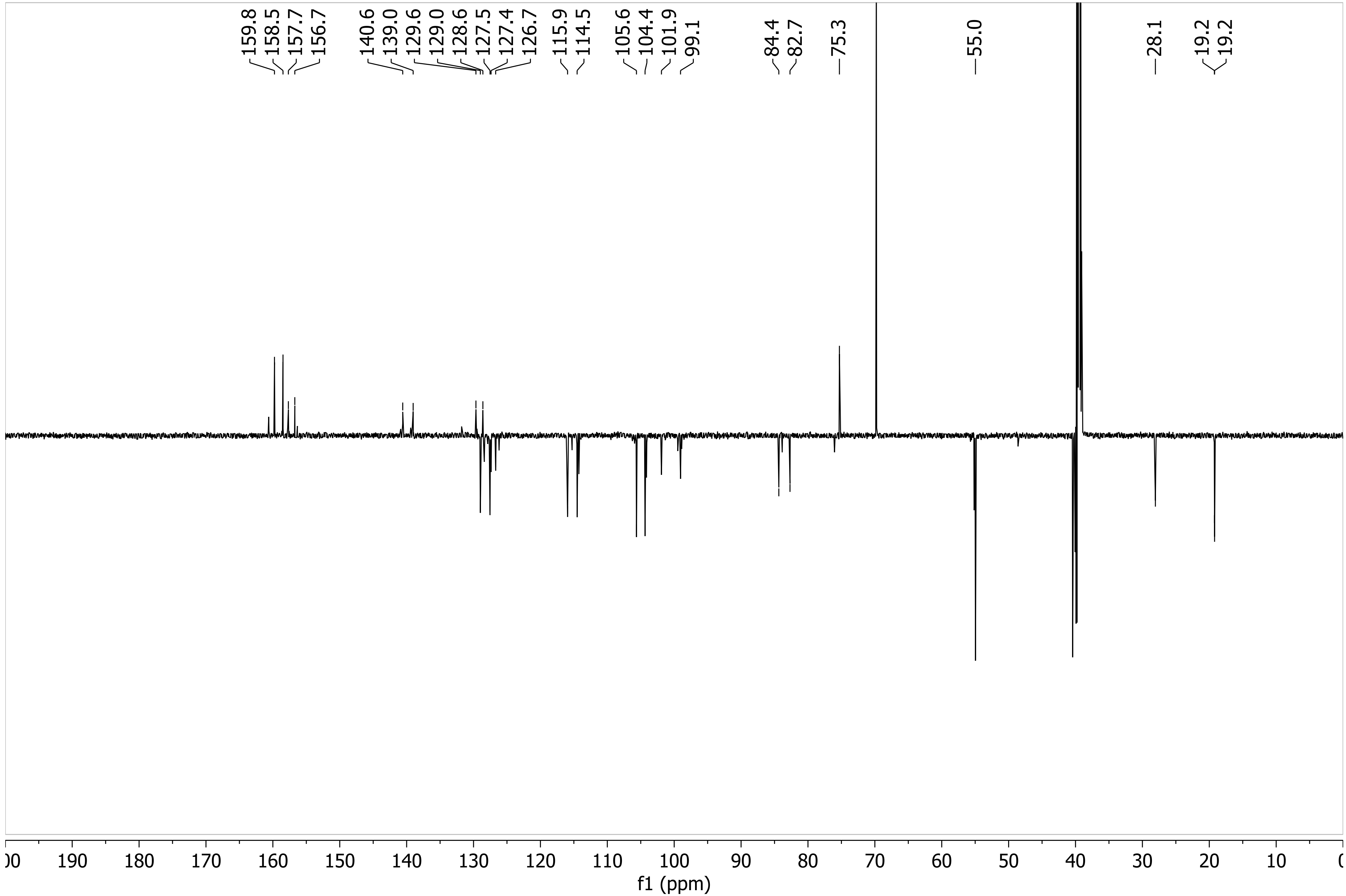

Edited-HSQC NMR spectrum of compound **46** in DMSO-d_6

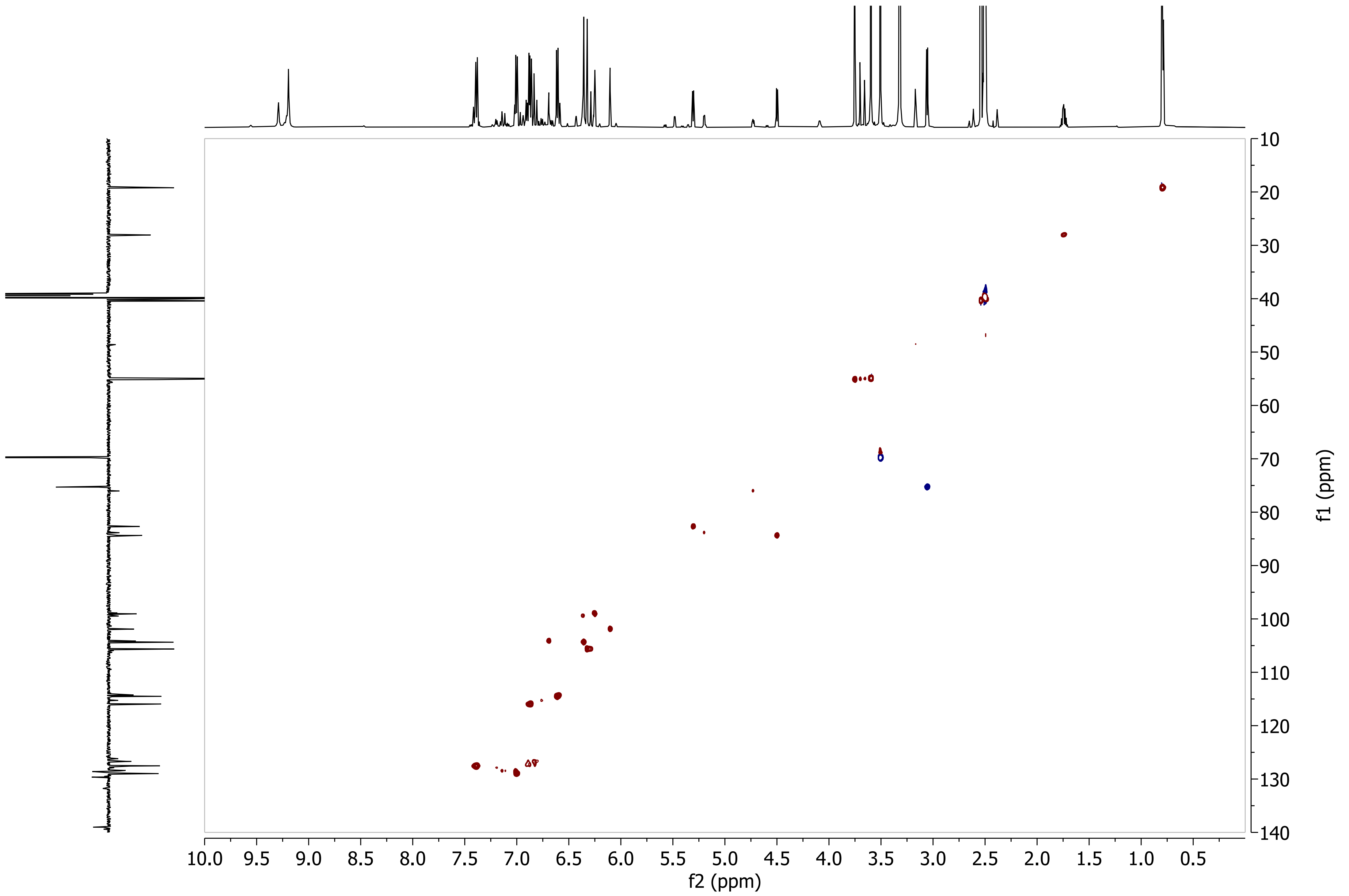

HMBC NMR spectrum of compound **46** in DMSO-d_6

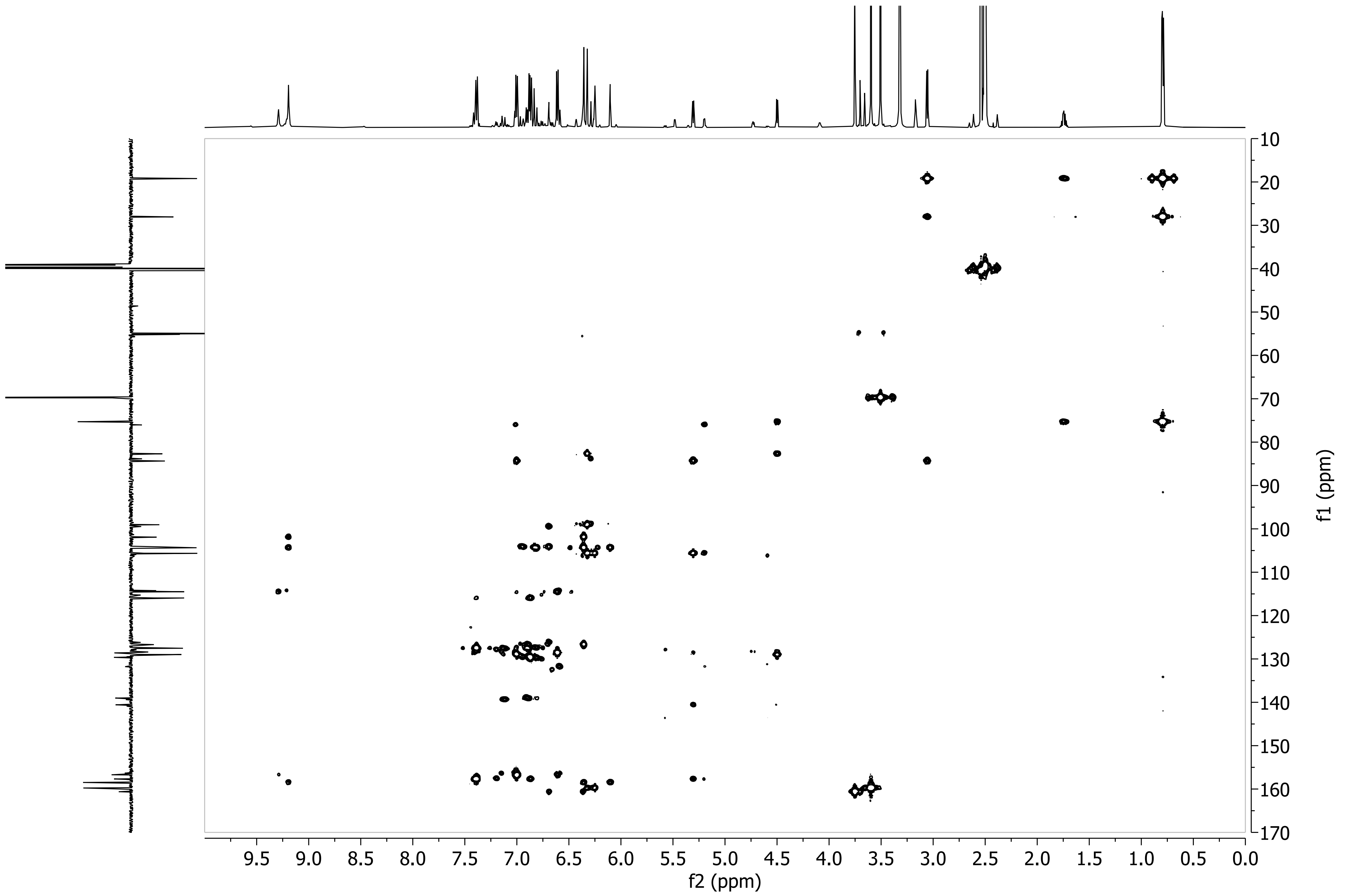

ROESY NMR spectrum of compound **46** in DMSO-d_6

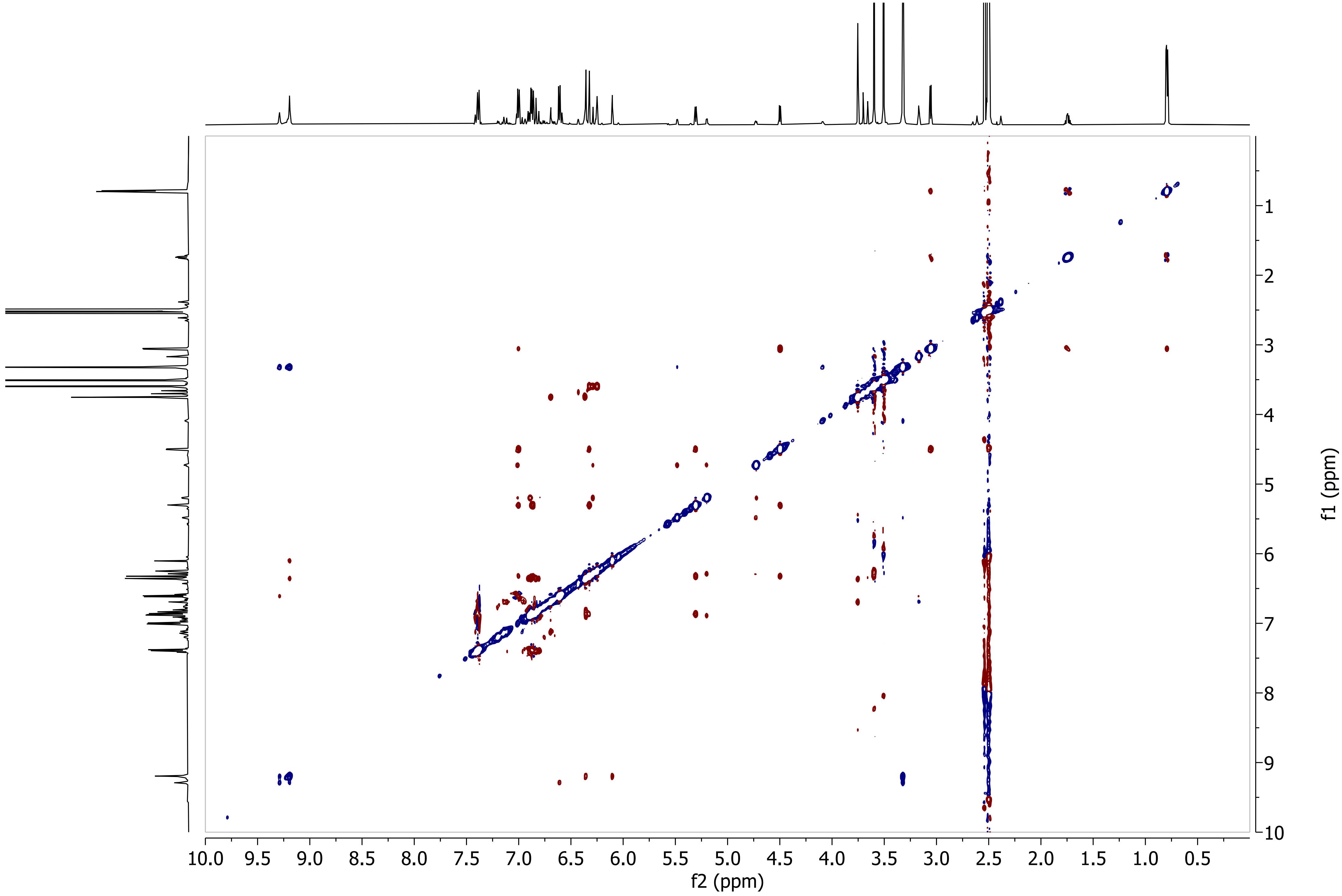

^{1}H NMR spectrum of compound **47** in DMSO-d_6

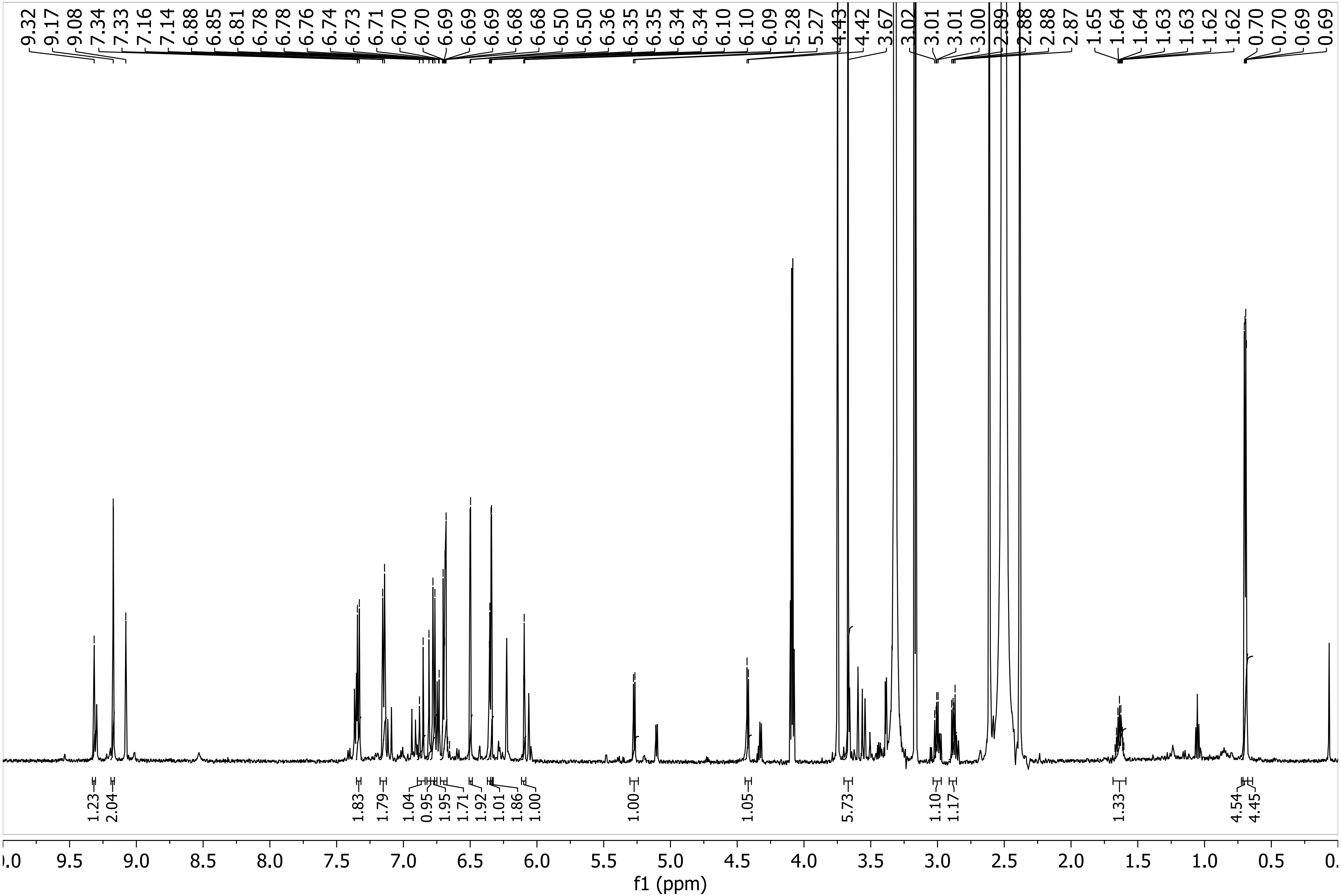

COSY NMR spectrum of compound **47** in DMSO-d_6

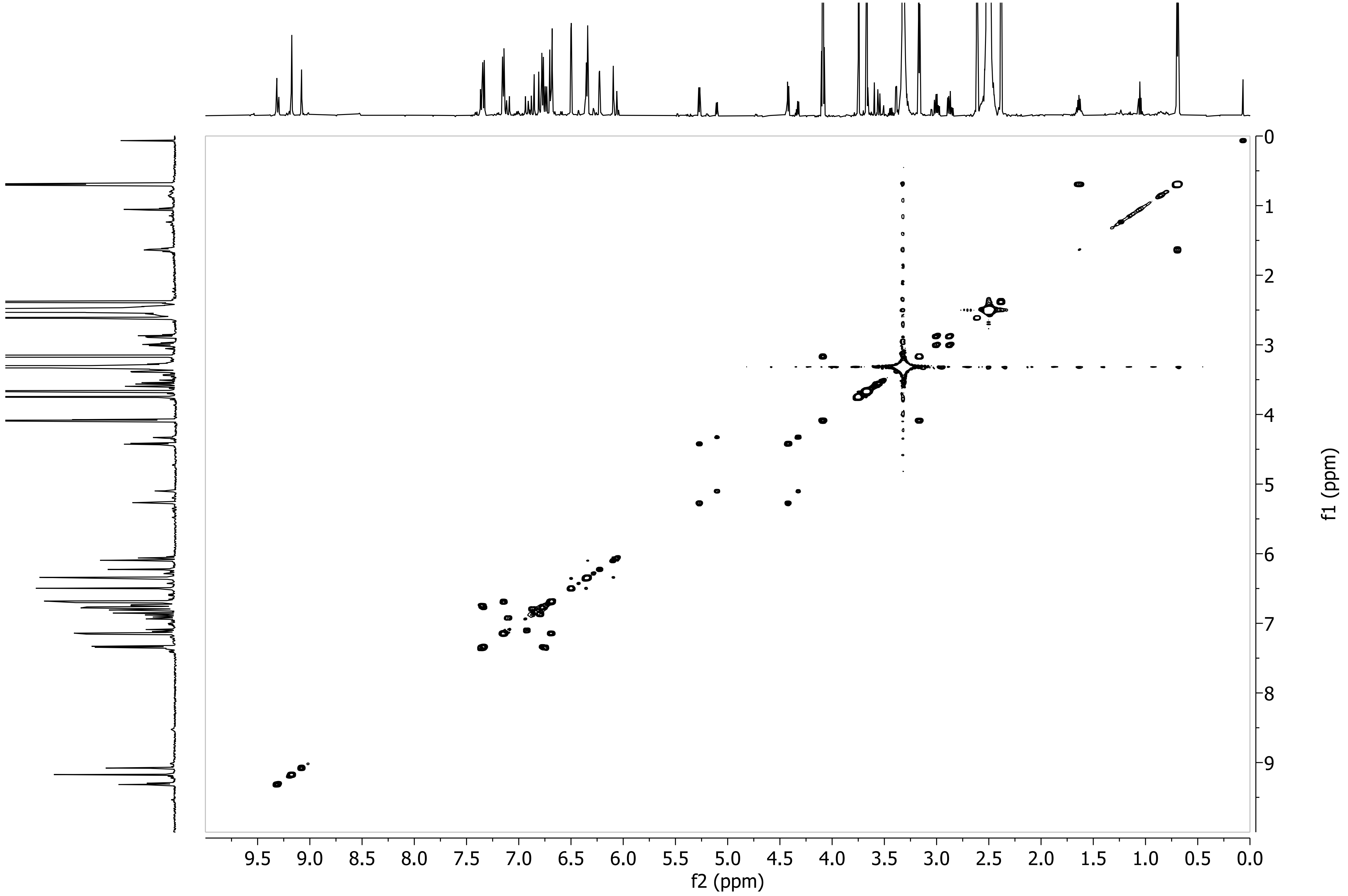

Edited-HSQC NMR spectrum of compound **47** in DMSO-d_6

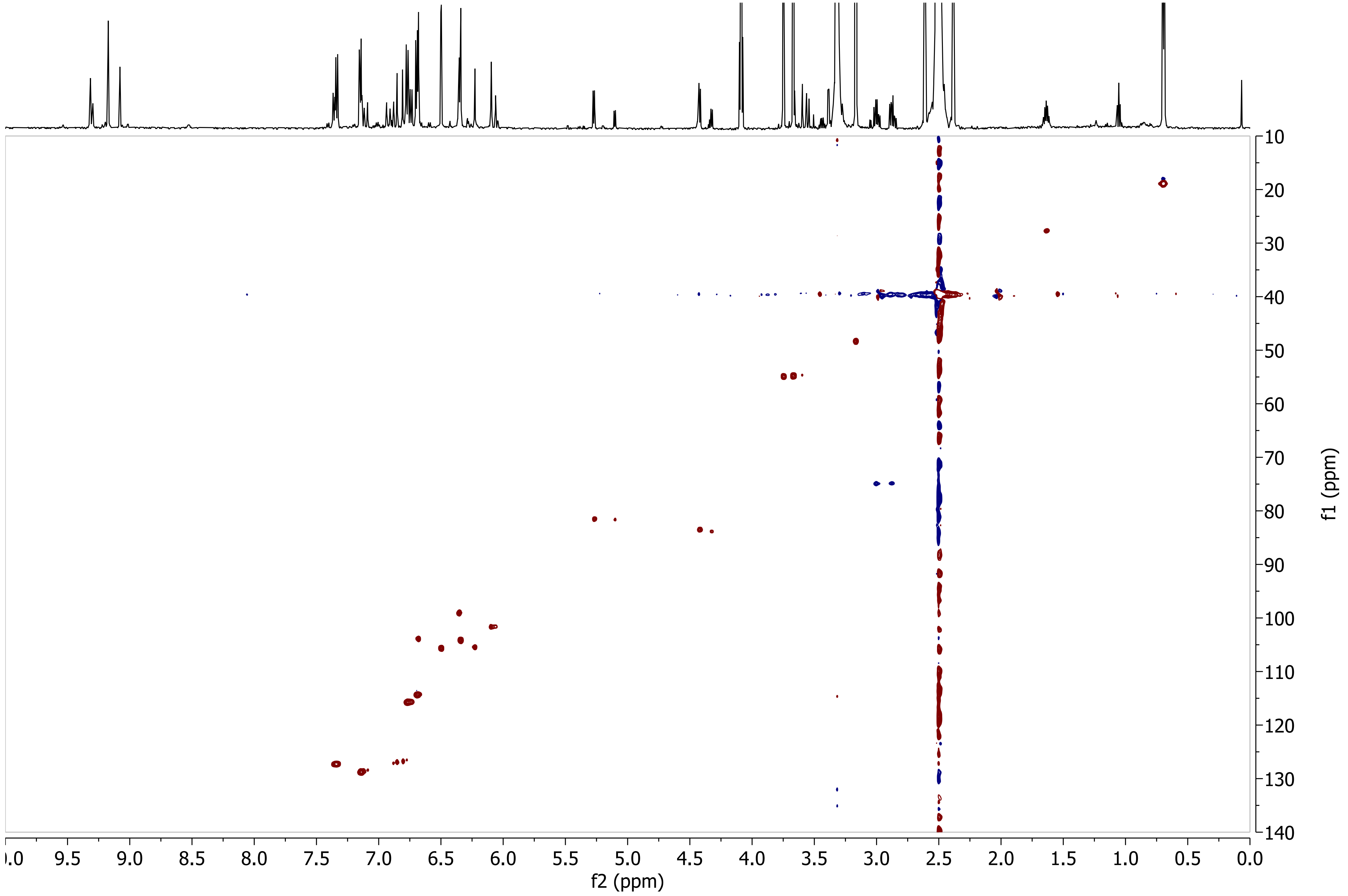

HMBC NMR spectrum of compound **47** in DMSO-d_6

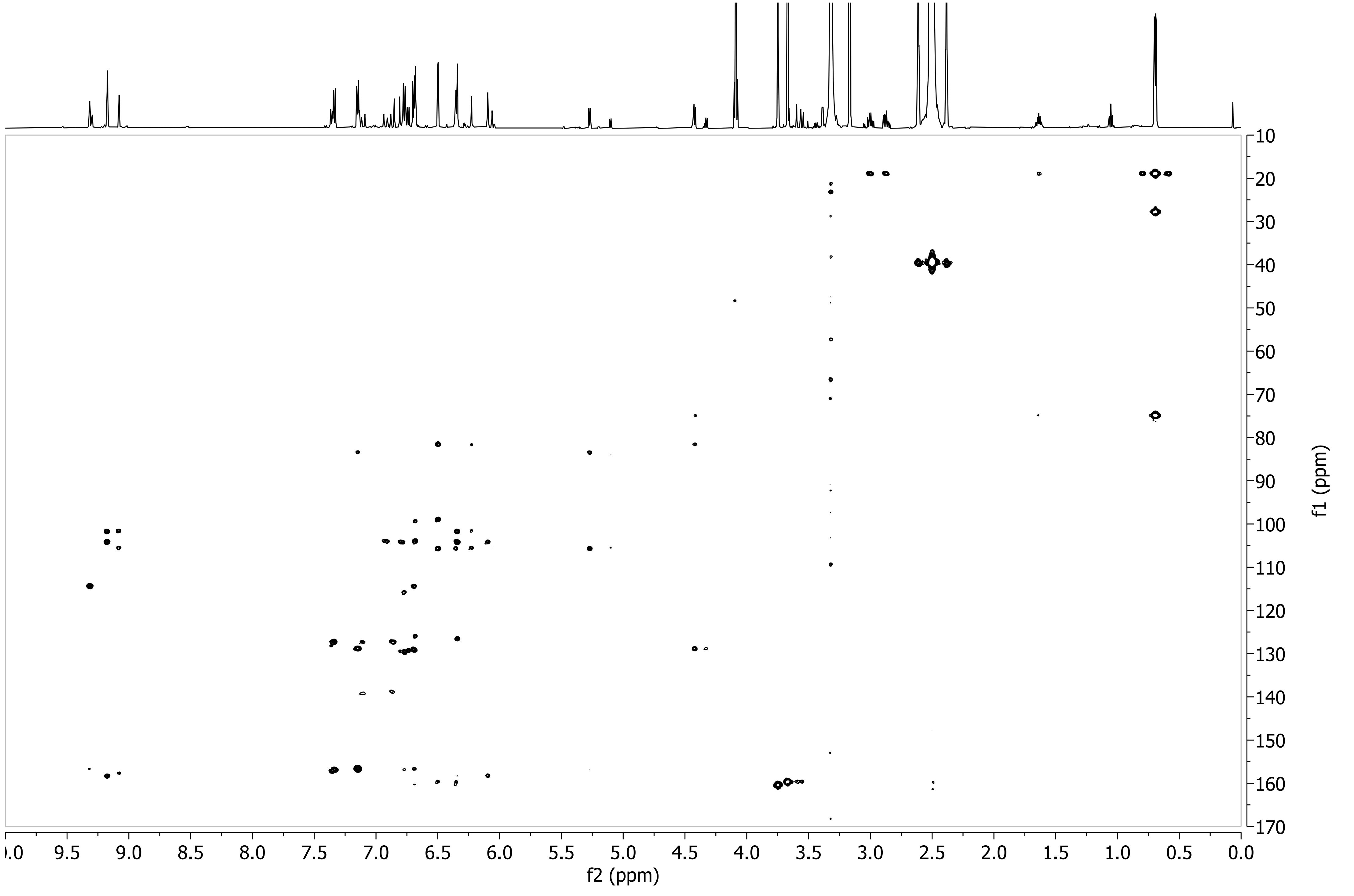

ROESY NMR spectrum of compound **47** in DMSO-d_6

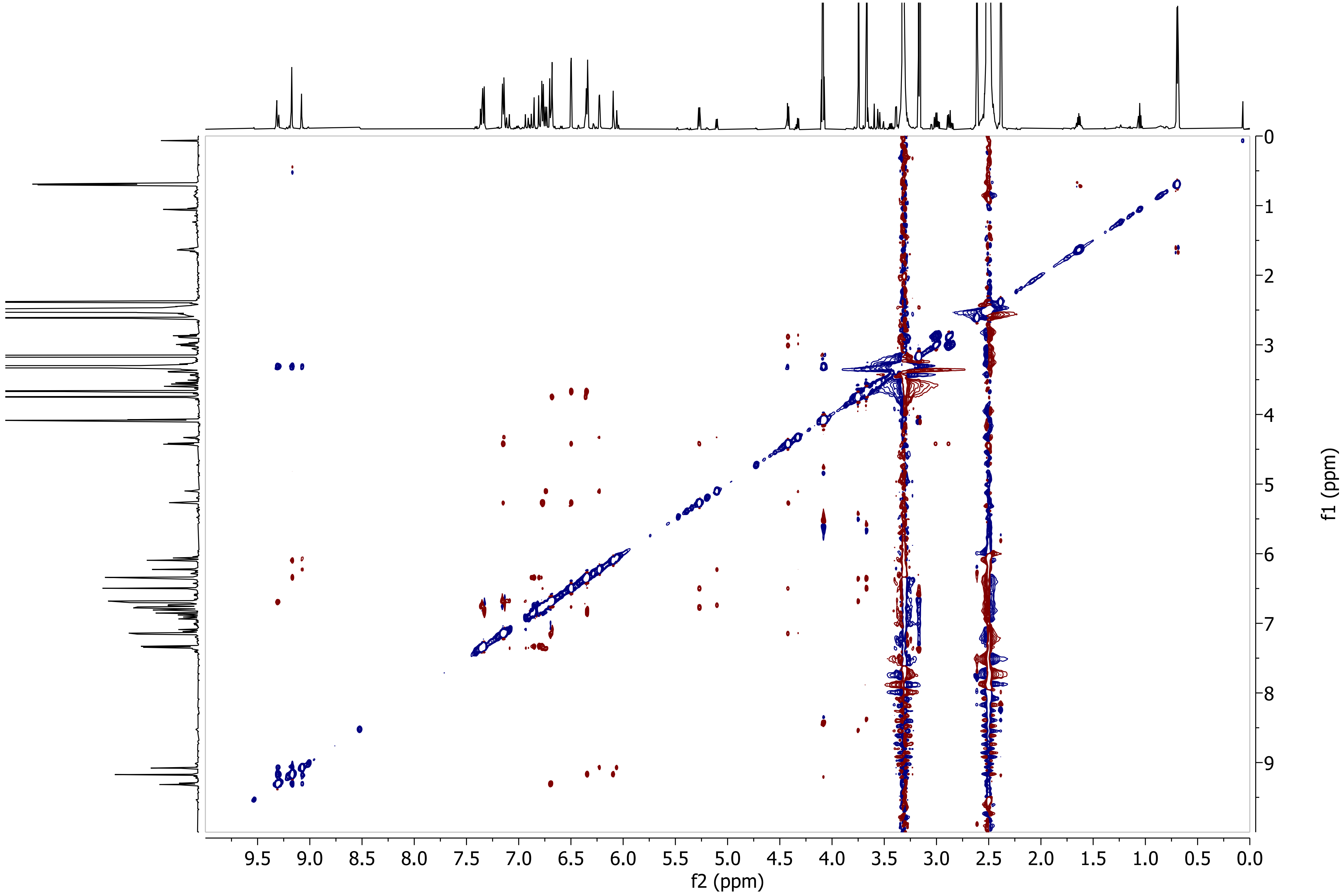

Supplement: Supplementary file 3 [file DataSheet3.pdf]
