# Supplementary material for: Chemoenzymatic Synthesis of Original Stilbene Dimers Possessing Wnt Inhibition Activity in Triple-Negative Breast Cancer Cells Using the Enzymatic Secretome of Botrytis cinerea Pers
Source: Front Chem. 2022 Apr 19;10:881298. doi: 10.3389/fchem.2022.881298 (PMC9062038; doi:10.3389/fchem.2022.881298)
Supplement: Supplementary file 4 [file DataSheet1.docx]

Supplementary Material


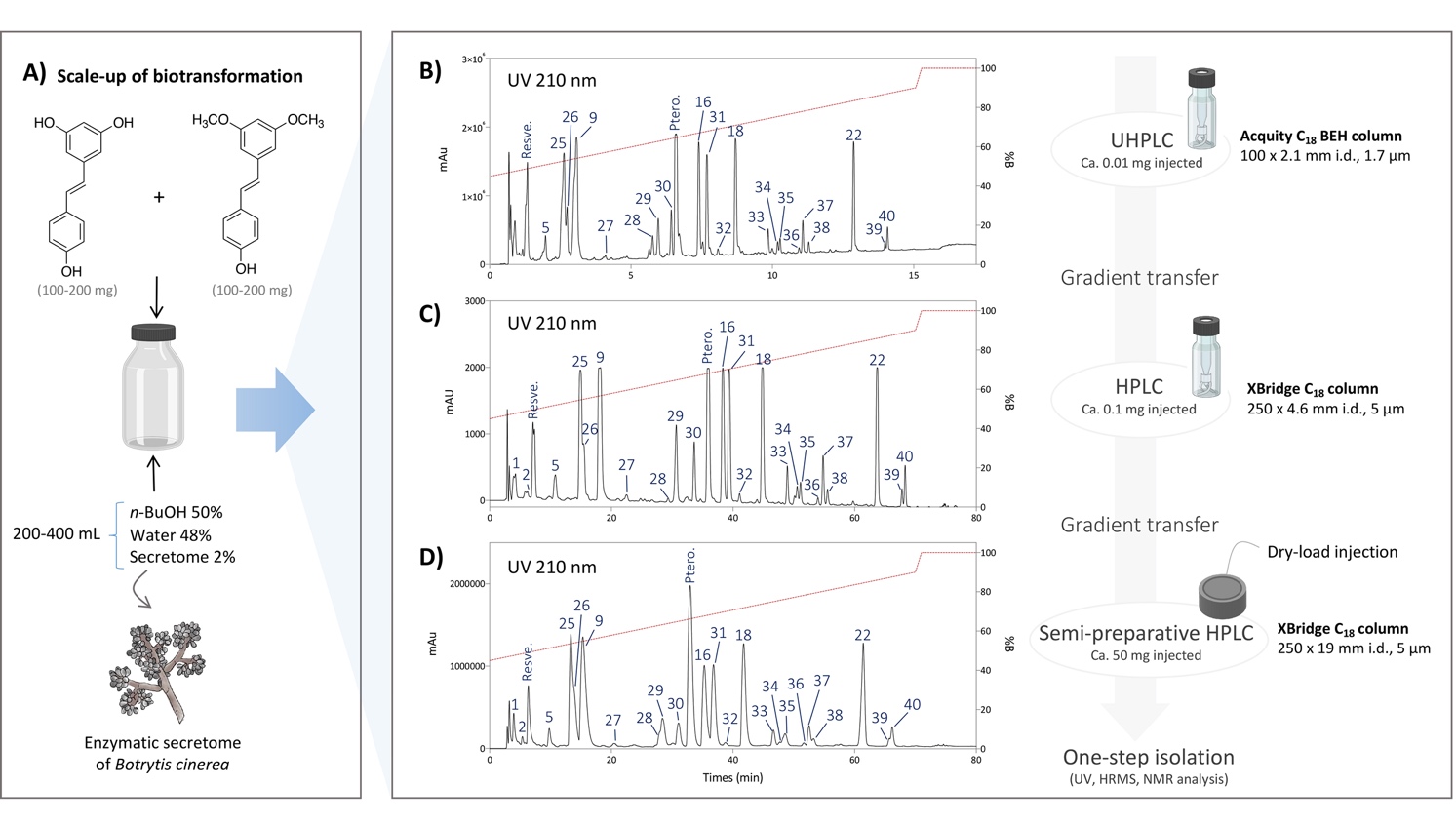


**Supplementary Figure 1.** A) Scale-up conditions of the biotransformation reaction of resveratrol and pterostilbene with the secretome of *Botrytis cinerea* with 50% of *n*-BuOH, 2% of enzymatic secretome and 48% of water (total volume 200-400 mL). B) Metabolite profile of the reaction after 24h using UHPLC-PDA with optimized chromatographic conditions. C) Analytical HPLC-PDA chromatogram after gradient transfer of the UHPLC-optimized conditions. D) Semi-preparative HPLC-PDA chromatogram after gradient transfer of the UHPLC-optimized conditions.


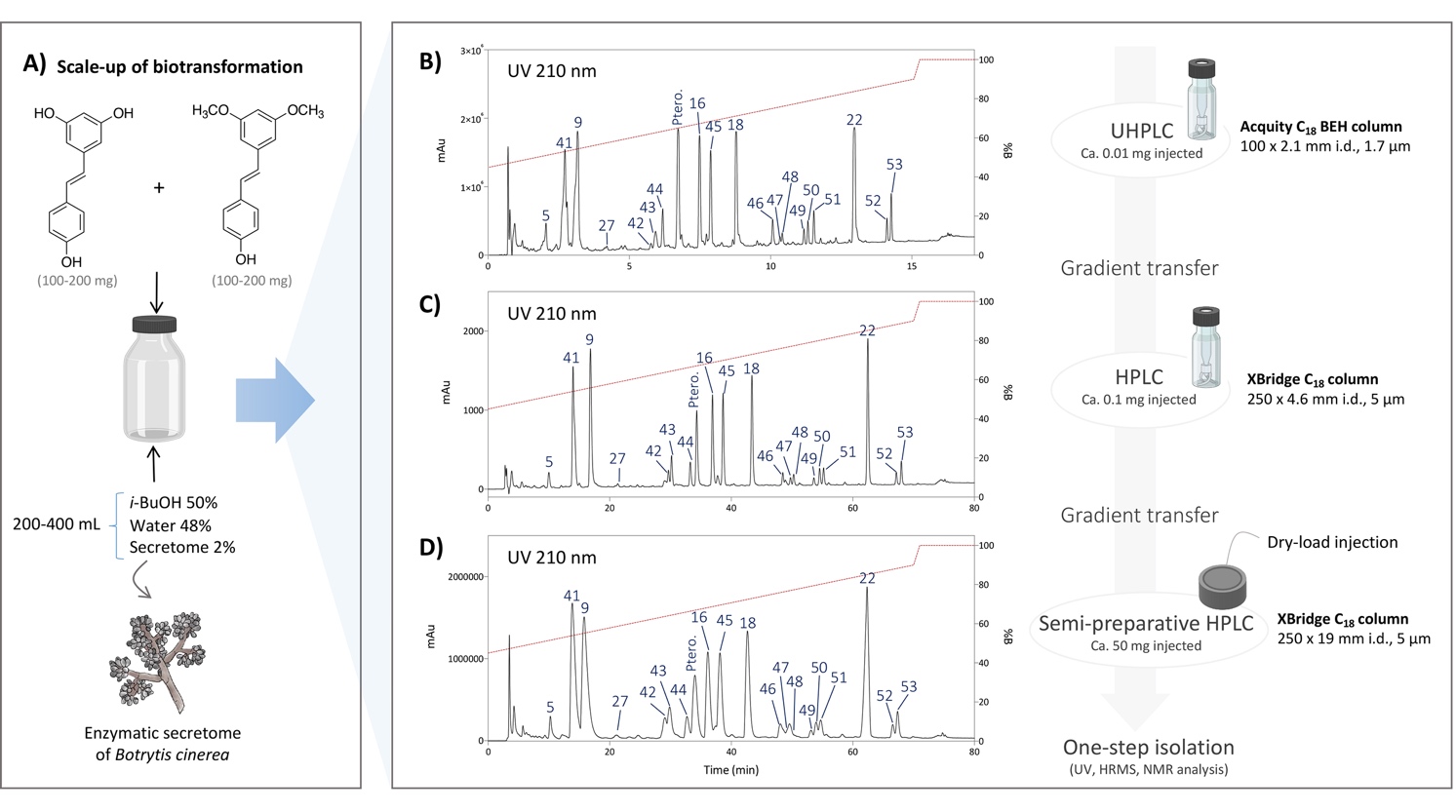


**Supplementary Figure 2.** A) Scale-up conditions of the biotransformation reaction of resveratrol and pterostilbene with the secretome of *Botrytis cinerea* with 50% of *i*-BuOH, 2% of enzymatic secretome and 48% of water (total volume 200-400 mL). B) Metabolite profile of the reaction after 24h using UHPLC-PDA with optimized chromatographic conditions. C) Analytical HPLC-PDA chromatogram after gradient transfer of the UHPLC-optimized conditions. D) Semi-preparative HPLC-PDA chromatogram after gradient transfer of the UHPLC-optimized conditions.


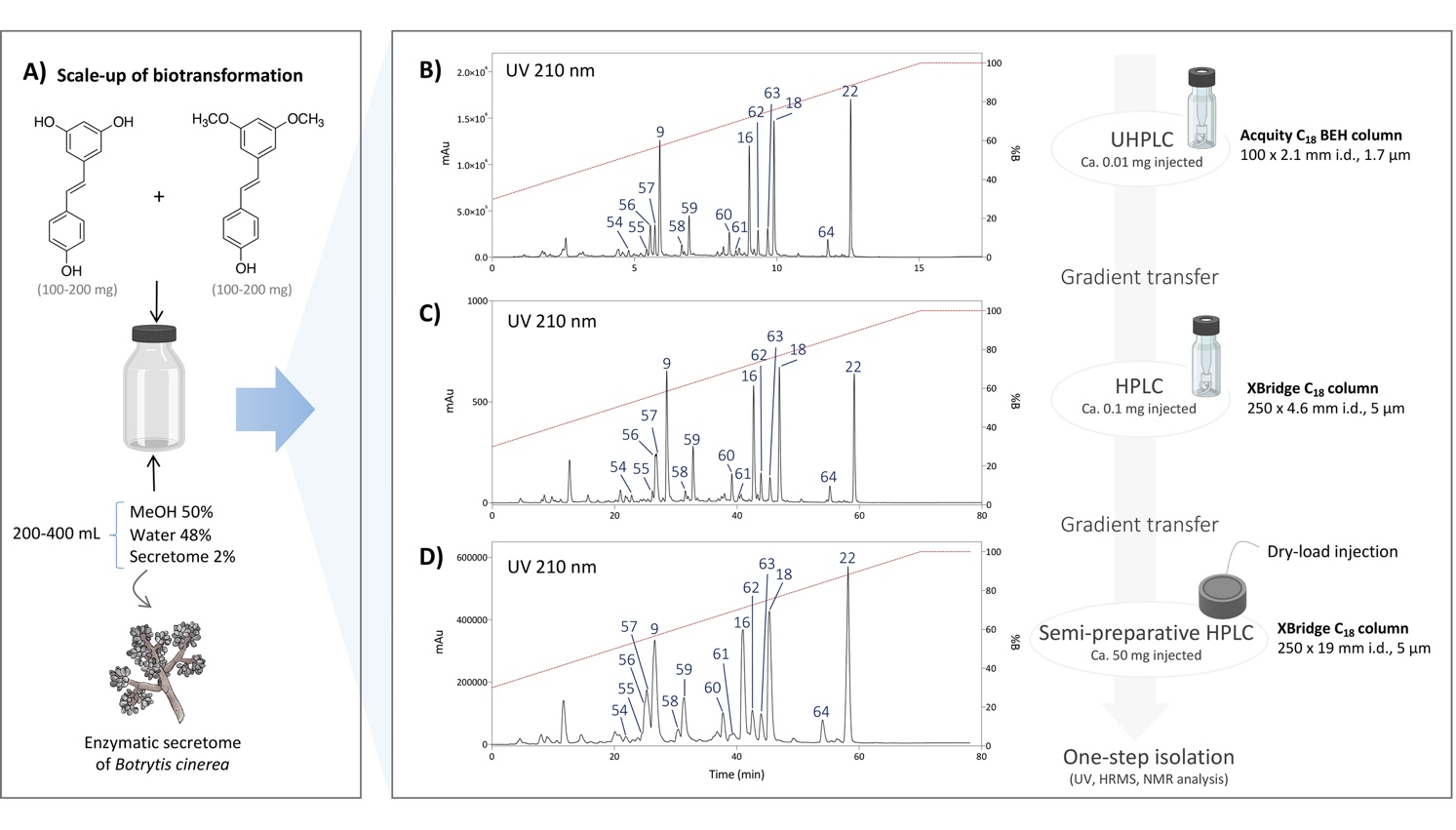


**Supplementary Figure 3.** A) Scale-up conditions of the biotransformation reaction of resveratrol and pterostilbene with the secretome of *Botrytis cinerea* with 50% of MeOH, 2% of enzymatic secretome and 48% of water (total volume 200-400 mL). B) Metabolite profile of the reaction after 24h using UHPLC-PDA with optimized chromatographic conditions. C) Analytical HPLC-PDA chromatogram after gradient transfer of the UHPLC-optimized conditions. D) Semi-preparative HPLC-PDA chromatogram after gradient transfer of the UHPLC-optimized conditions.


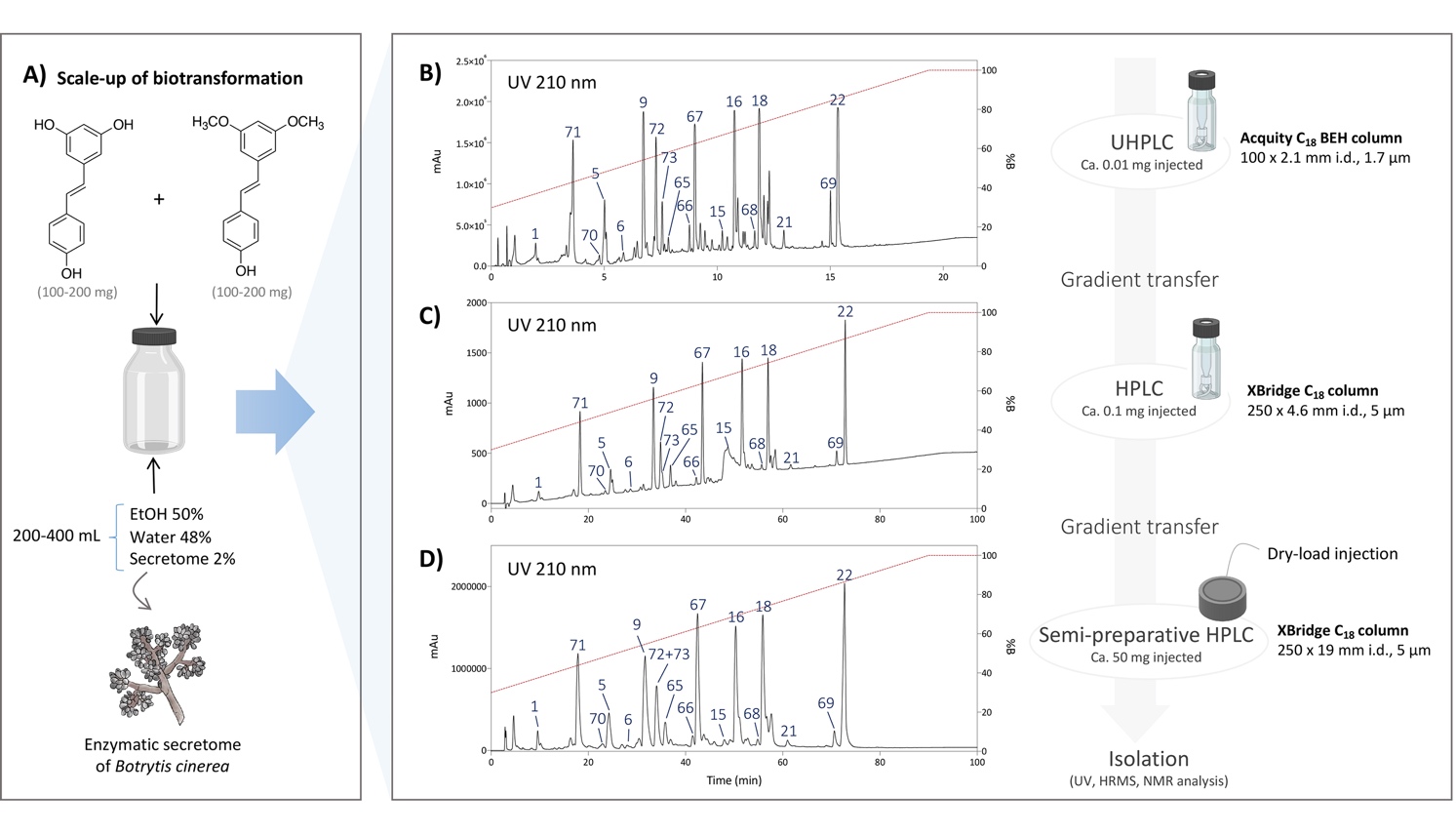


**Supplementary Figure 4.** A) Scale-up conditions of the biotransformation reaction of resveratrol and pterostilbene with the secretome of *Botrytis cinerea* with 50% of EtOH, 2% of enzymatic secretome and 48% of water (total volume 200-400 mL). B) Metabolite profile of the reaction after 24h using UHPLC-PDA with optimized chromatographic conditions. C) Analytical HPLC-PDA chromatogram after gradient transfer of the UHPLC-optimized conditions. D) Semi-preparative HPLC-PDA chromatogram after gradient transfer of the UHPLC-optimized conditions.


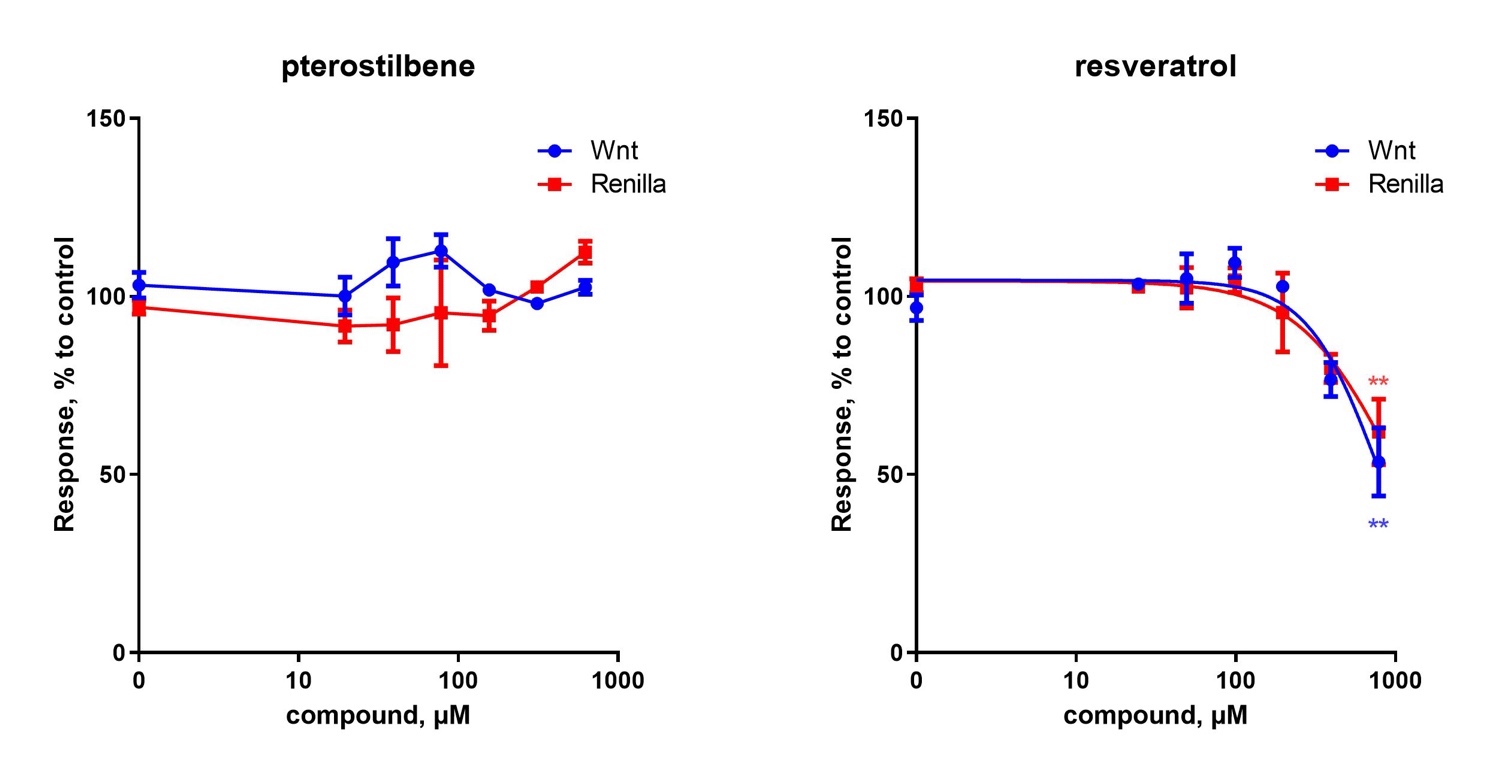


**Supplementary Figure 5.** Neither pterostilbene (left panel), nor resveratrol (right panel) are capable of specific inhibition of Wnt signaling in BT-20 TNBC cell line. While pterostilbene is completely inactive, resveratrol shows some unspecific toxicity with IC_50_ approaching 1mM. Statistical significance for both compounds was assessed by one-way ANOVA followed by multiple comparisons with DMSO-treated values, p values are shown as **p<0.01


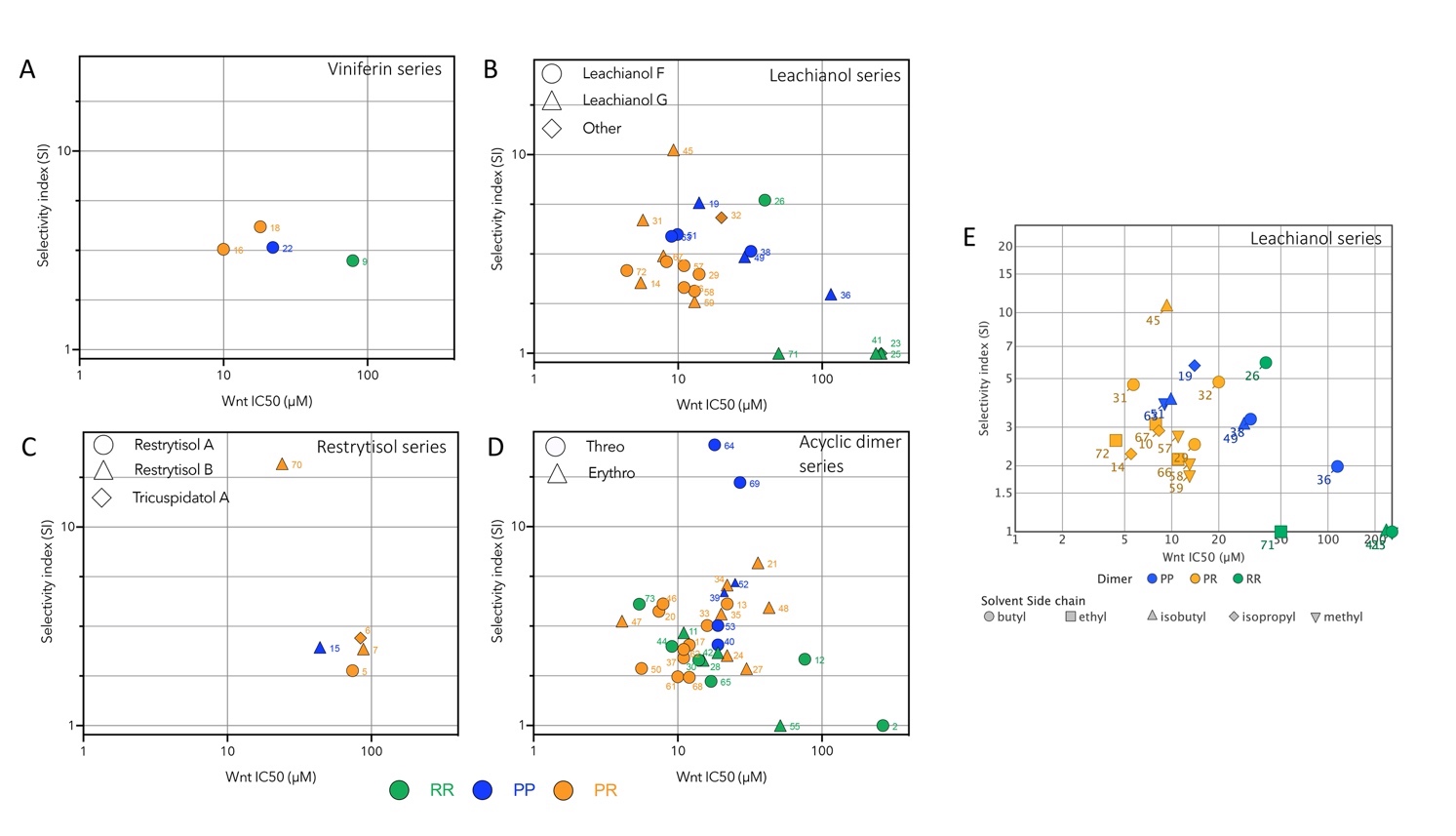


**Supplementary Figure 6.** Relations between derivative scaffolds and anti-Wnt activity, subplots by scaffold type. (A) viniferin series. (B) leachianol series. (C) restrytisol series. (D) acyclic dimer series. (E) Influence of the solvent side chain in the case of the leachianol series.


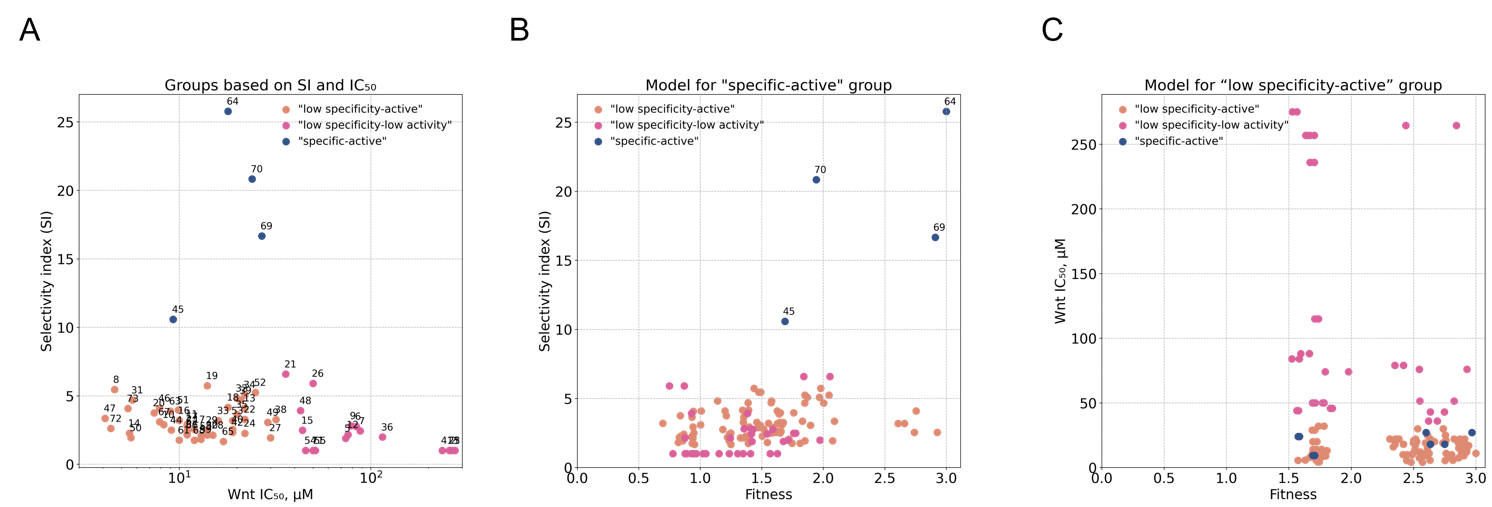


**Supplementary Figure 7.** (A) Custom groups based on the IC_50_ and SI. (B) Performance for the “specific-active” group model. (C) Performance for the “low specificity-active” group model. As the IC50 does not correlate with the fitness, this model can only be used as a descriptive model.


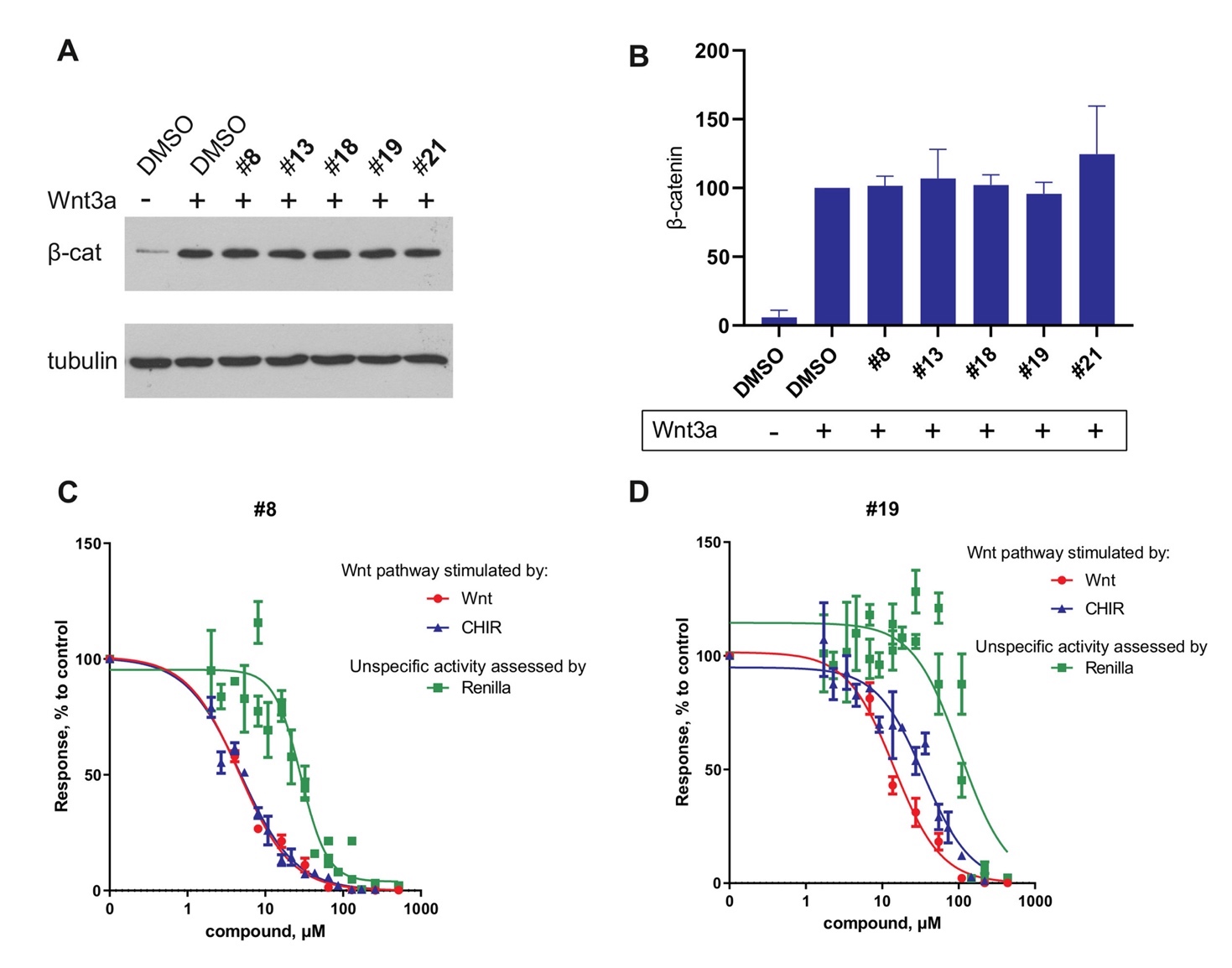


**Supplementary Figure 8.** The compounds target in Wnt pathway is below the level of beta-catenin accumulation. (A, B) Representative Western blot and quantification demonstrating absence of effect of any compound on β-catenin accumulation stimulated by Wnt3a. (C, D) Compounds **8** and **19** are suppressing CHIR99021 (“CHIR”)-induced response in Wnt signaling with the same potency as Wnt3a-induced signal.


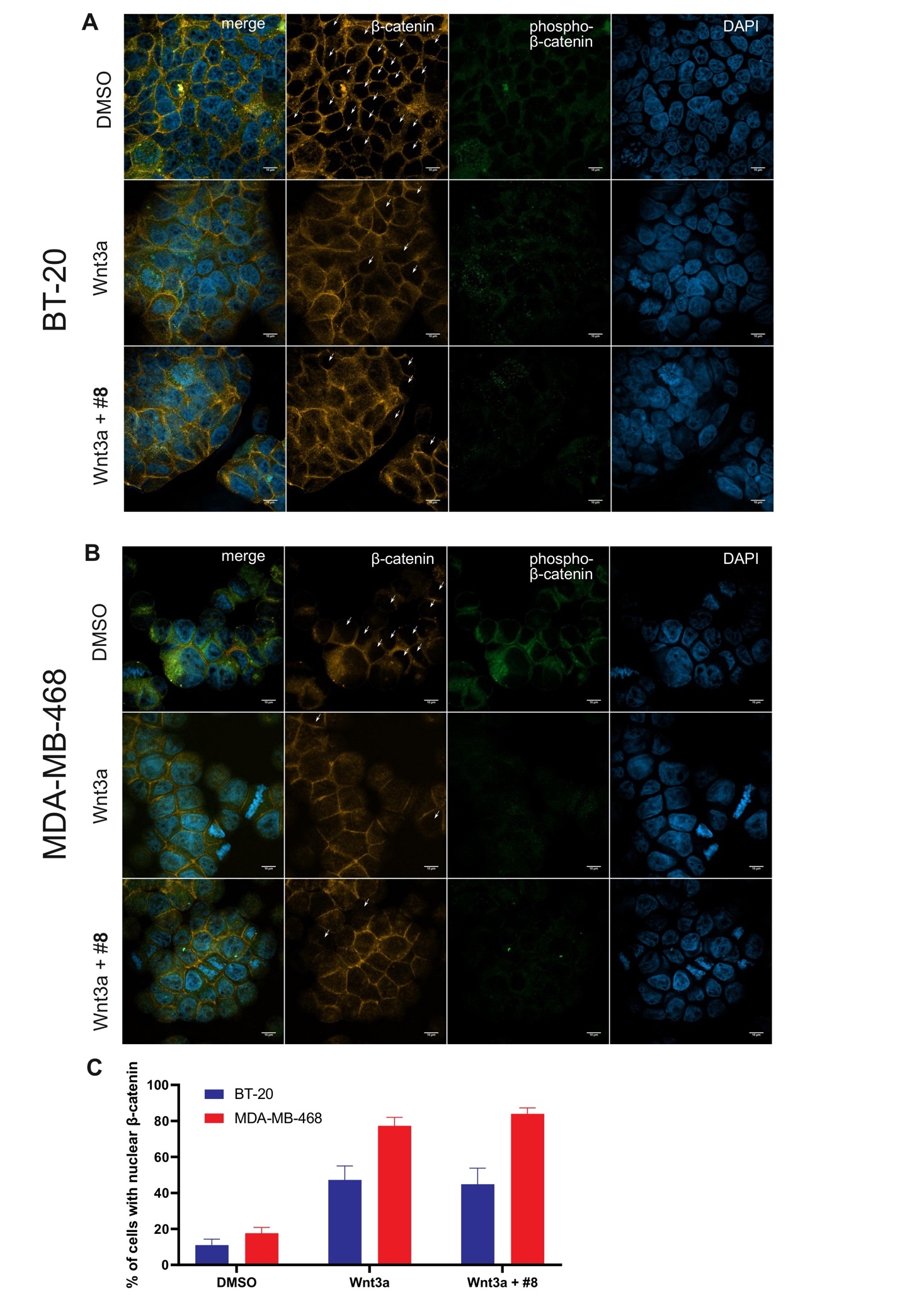


**Supplementary Figure 9.** Compound **8** does not affect Wnt3a-mediated translocation or promote phosphorylation of β-catenin. Stainings against β-catenin and phospho-β-catenin were made in (A) BT-20 cells and (B) MDA-MB-468 cells and quantified on (C). In either line a number of cells positive for nuclear β-catenin remained the same. In MDA-MB-468 cells Wnt3a treatment additionally results in strong and visible drop in phospho-β-catenin, which is not reversed by compound treatment. The cells which do not present cytoplasmic/nuclear β-catenin are marked with white arrows on corresponding stainings for both cell lines.

|  |  |  | | |  | |  |  |  | |  | |  | |  | |  | | | |  |  |  | | |
| --- | --- | --- | --- | --- | --- | --- | --- | --- | --- | --- | --- | --- | --- | --- | --- | --- | --- | --- | --- | --- | --- | --- | --- | --- | --- |
| **Co-solvent** | **UHPLC** (100 x 2.1 mm i.d., 1.7 µm) | | | | | | | **HPLC** (250 x 4.6 mm i.d., 5 µm) | | | | | | | | | | **Semi-preparative HPLC** (250 x 19 mm i.d., 5 µm) | | | | | |  |  |
|  | Flow-rate (mL/min) | | %A | %B | | Time (min) | | Flow-rate (mL/min) | | %A | | | | %B | | Time (min) | | Flow-rate (mL/min) | %A | %B | Time (min) | | |  |  |
| *i*-PrOH | 0.4 | | 60 | 40 | | 0 | | 1 | | 60 | | 40 | | | | 0 | | 17 | 60 | 40 | 0 | | |  |  |
|  |  |  | 10 | 90 | | 15.07 | |  |  | 10 | | 90 | | | | 70 | |  | 10 | 90 | 70 | | |  |  |
|  |  |  | 0 | 100 | | 15.28 | |  |  | 0 | | 100 | | | | 71 | |  | 0 | 100 | 71 | | |  |  |
|  |  |  | 0 | 100 | | 19.40 | |  |  | 0 | | 100 | | | | 90 | |  | 0 | 100 | 90 | | |  |  |
| *n*-BuOH and *i*-BuOH | 0.4 | | 55 | 45 | | 0 | | 1 | | 55 | | 45 | | | | 0 | | 17 | 55 | 45 | 0 | | |  |  |
|  |  |  | 10 | 90 | | 15.07 | |  |  | 10 | | 90 | | | | 70 | |  | 10 | 90 | 70 | | |  |  |
|  |  |  | 0 | 100 | | 15.28 | |  |  | 0 | | 100 | | | | 71 | |  | 0 | 100 | 71 | | |  |  |
|  |  |  | 0 | 100 | | 19.40 | |  |  | 0 | | 100 | | | | 90 | |  | 0 | 100 | 90 | | |  |  |
| EtOH | 0.4 | | 70 | 30 | | 0 | | 1 | | 70 | | 30 | | | | 0 | | 17 | 70 | 30 | 0 | | |  |  |
|  |  |  | 0 | 100 | | 19.40 | |  |  | 0 | | 100 | | | | 90 | |  | 0 | 100 | 90 | | |  |  |
|  |  |  | 0 | 100 | | 22 | |  |  | 0 | | 100 | | | | 100 | |  | 0 | 100 | 100 | | |  |  |
| MeOH | 0.4 | | 70 | 30 | | 0 | | 1 | | 70 | | 30 | | | | 0 | | 17 | 70 | 30 | 0 | | |  |  |
|  |  |  | 0 | 100 | | 15.07 | |  |  | 0 | | 100 | | | | 70 | |  | 0 | 100 | 70 | | |  |  |
|  |  |  | 0 | 100 | | 17.21 | |  |  | 0 | | 100 | | | | 80 | |  | 0 | 100 | 80 | | |  |  |
|  |  | |  |  | |  | |  | |  | |  | | | |  | |  |  |  |  | | |  |  |
|  |  | |  |  | |  | |  | |  | |  | | | |  | |  |  |  |  | | |  |  |

**Supplementary Table 1.** Chromatographic methods optimized at the UHPLC scale and geometrically transferred to the analytical HPLC and semi-preparative HPLC scale.
